# Boron-Based Inhibitors of the NLRP3 Inflammasome

**DOI:** 10.1016/j.chembiol.2017.08.011

**Published:** 2017-11-16

**Authors:** Alex G. Baldwin, Jack Rivers-Auty, Michael J.D. Daniels, Claire S. White, Carl H. Schwalbe, Tom Schilling, Halah Hammadi, Panichakorn Jaiyong, Nicholas G. Spencer, Hazel England, Nadia M. Luheshi, Manikandan Kadirvel, Catherine B. Lawrence, Nancy J. Rothwell, Michael K. Harte, Richard A. Bryce, Stuart M. Allan, Claudia Eder, Sally Freeman, David Brough

**Affiliations:** 1 Division of Pharmacy and Optometry, School of Health Sciences, Faculty of Biology, Medicine and Health, Manchester Academic Health Science Centre, University of Manchester, Stopford Building, Oxford Road, Manchester M13 9PT, UK; 2 Division of Neuroscience and Experimental Psychology, School of Biological Sciences, Faculty of Biology, Medicine and Health, Manchester Academic Health Science Centre, University of Manchester, AV Hill Building, Oxford Road, Manchester M13 9PT, UK; 3 Aston Pharmacy School, School of Life & Health Sciences, Aston University, Aston Triangle, Birmingham B4 7ET, UK; 4 St. George's, University of London, Institute for Infection and Immunity, Cranmer Terrace, London SW17 0RE, UK; 5 MedImmune Ltd., Aaron Klug Building, Granta Park, Cambridge CB21 6GH, UK

**Keywords:** boron, inflammation, NLRP3 inflammasome, inhibitor, interleukin-1

## Abstract

NLRP3 is a receptor important for host responses to infection, yet is also known to contribute to devastating diseases such as Alzheimer's disease, diabetes, atherosclerosis, and others, making inhibitors for NLRP3 sought after. One of the inhibitors currently in use is 2-aminoethoxy diphenylborinate (2APB). Unfortunately, in addition to inhibiting NLRP3, 2APB also displays non-selective effects on cellular Ca^2+^ homeostasis. Here, we use 2APB as a chemical scaffold to build a series of inhibitors, the NBC series, which inhibit the NLRP3 inflammasome *in vitro* and *in vivo* without affecting Ca^2+^ homeostasis. The core chemical insight of this work is that the oxazaborine ring is a critical feature of the NBC series, and the main biological insight the use of NBC inhibitors led to was that NLRP3 inflammasome activation was independent of Ca^2+^. The NBC compounds represent useful tools to dissect NLRP3 function, and may lead to oxazaborine ring-containing therapeutics.

## Highlights

• New inhibitors of the NLRP3 inflammasome are described
• NLRP3 inflammasome activation is independent of Ca^2+^
• These new inflammasome inhibitors are effective *in vivo*
• The inhibitors described may lead to the development of new drugs

## Graphical Abstract

**Figure:**
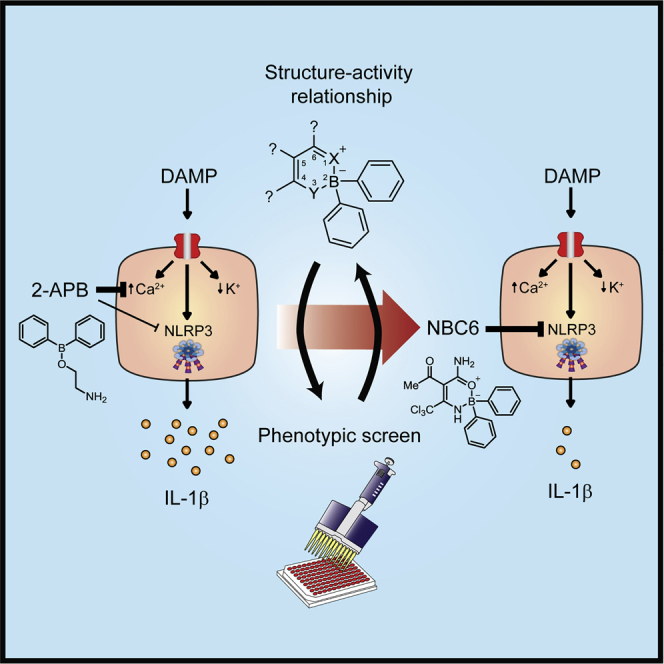

## Teaser

The NLRP3 inflammasome is known to contribute to damaging inflammation during disease. Baldwin et al. describe a new boron-containing series of inflammasome inhibitors, which may lead to the development of new anti-inflammatory molecules and allow further interrogation of inflammatory mechanisms.

## Introduction

Inflammation, which contributes to almost all known non-infectious diseases, is triggered by infection or injury sensed by pattern recognition receptors (PRRs) on inflammatory cells. Soluble PRRs have received particular attention due to their ability to form molecular complexes known as inflammasomes, which facilitate the release of inflammatory cytokines such as interleukin-1β (IL-1β), an important aspect of the inflammatory response. Inflammasomes are formed following the activation of cytosolic PRRs, of which the NOD-like receptor (NLR) family, pyrin domain-containing protein 3 (NLRP3), is the best characterized. The NLRP3 inflammasome is formed when NLRP3, described mainly in macrophages and monocytes, senses the presence of pathogen-associated molecular patterns (PAMPs) or damage-associated molecular patterns (DAMPs). Upon its activation, NLRP3 binds to the adapter protein ASC (apoptosis-associated speck-like protein containing a caspase activation and recruitment domain), which in turn recruits pro-caspase-1 to form an inflammasome complex. This results in the activation of caspase-1, which in turn cleaves pro-forms of the pro-inflammatory cytokines IL-1β and IL-18, causing their activation and facilitating their release from the cell (Latz et al., 2013).

NLRP3-dependent cytokine release is implicated in the development of several important diseases (McGettrick and O'Neill, 2013, Heneka et al., 2015) and may represent a pharmacological target for the treatment of inflammatory disease (Coll et al., 2015, Daniels et al., 2016). Signaling mechanisms regulating the activation of NLRP3 remain to be fully characterized. One signaling mechanism proposed to regulate the activation of the NLRP3 inflammasome is an increase in intracellular calcium ([Ca^2+^]_i_) (Horng, 2014). Many reports suggesting an involvement of Ca^2+^ in inflammasome activation have used the Ca^2+^-signaling inhibitor 2-aminoethoxy diphenylborinate (2APB, **1**) (Lee et al., 2012, Murakami et al., 2012, Compan et al., 2012, Rossol et al., 2012).

2APB is a cell-permeable small-molecule inhibitor of Ca^2+^ homeostasis with multiple targets including inositol 1,4,5-trisphosphate (InsP_3_)-dependent Ca^2+^ release, store-operated Ca^2+^ entry, and potentially also Ca^2+^ pumps and mitochondria, where effects are described as use-dependent and poorly reversible (Peppiatt et al., 2003). 2APB is also a poorly selective TRP (transient receptor potential) channel blocker (Schaefer, 2014). However, recent evidence suggests that the effects of 2APB on inflammasome activation may be independent of an effect on Ca^2+^ (Katsnelson et al., 2015). The utility of 2APB as an inhibitor of NLRP3, however, is limited by its non-selective effects on cellular Ca^2+^ homeostasis. Our aim was to develop new and potent inflammasome inhibitors based on the scaffold of 2APB but with reduced non-specific effects on Ca^2+^ homeostasis. We describe NBC6 (and its analogs) as completely new and potent inhibitors of the NLRP3 inflammasome that act independently of Ca^2+^.

## Results

### Inhibitory Effects of 2APB Require Boron

**Figure 1:**
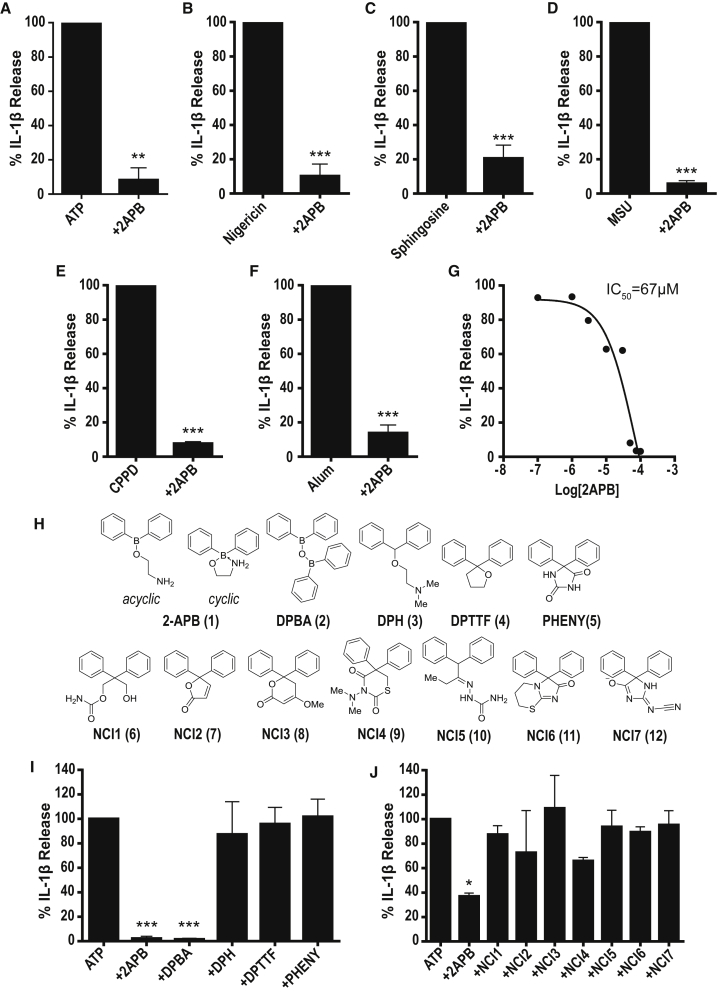
Establishing the Importance of Boron in 2APB for NLRP3 Inflammasome Inhibition (A–G) Mouse peritoneal macrophages were primed with bacterial endotoxin (lipopolysaccharide [LPS], 1 μg mL^−1^, 2 hr) and then stimulated with vehicle (0.5% DMSO) or 2APB (75 μM) before stimulation with ATP (5 mM, 20 min) (A), nigericin (20 μM, 15 min) (B), sphingosine (20 μM, 1 hr) (C), monosodium urate crystals (MSU; 250 μg mL^−1^, 1 hr) (D), calcium pyrophosphate dehydrate crystals (CPPD; 250 μg mL^−1^, 1 hr) (E), or aluminum hydroxide (Alum; 250 μg mL^−1^, 1 hr) (F). The half-maximal inhibitory concentration (IC_50_) for the effects of 2APB on IL-1β release induced by ATP was established using a 3-parameter logistical sigmoidal model (G). (H) Chemical structures of B-containing compounds 2APB (**1**) and DPBA (**2**) and C-containing 2APB analogs (**3–12**). (I and J) Mouse peritoneal macrophages were primed as before and stimulated with vehicle (0.5% DMSO) or inhibitor (**1–5**, 75 μM) before stimulation with ATP (5 mM, 20 min) (I). Mouse BMDMs were primed with LPS (1 μg mL^−1^, 4 hr) and incubated with vehicle (0.5% DMSO) or molecules (NCI1–7, **6–12**, 40 μM) for 15 min before ATP stimulation (5 mM, 1 hr) (J). In all cases supernatants were analyzed by ELISA. Data are presented as mean percentage of IL-1β release versus vehicle (DMSO) control + SEM (n = 3–9). *p < 0.05, **p < 0.01, ***p < 0.001, significant difference from 100% IL-1β release (Holm-Sidak corrected one-sample t test).

To establish that 2APB was a robust NLRP3 inflammasome inhibitor, mouse peritoneal macrophages were primed with LPS and then stimulated with a range of NLRP3 inflammasome-activating DAMPs. After LPS cells received pre-treatment with 2APB, which was then present for the duration of DAMP stimulation, 2APB inhibited the release of IL-1β in response to NLRP3 inflammasome activators ATP, nigericin, sphingosine, monosodium urate crystals (MSU), calcium pyrophosphate dihydrate crystals (CPPD), or aluminum hydroxide (Alum) (Figures 1A–1F), consistent with previous work reporting 2APB as an inhibitor of the NLRP3 inflammasome (Lee et al., 2012, Murakami et al., 2012, Compan et al., 2012, Rossol et al., 2012, Katsnelson et al., 2015). To identify the pharmacophore of 2APB responsible for the inhibition of IL-1β processing and release, we screened a small library of 2APB analogs based on previously published data investigating the pharmacophore responsible for the effects of 2APB on store-operated Ca^2+^ entry (Dobrydneva and Blackmore, 2001). The acyclic structure of 2APB (**1**) is shown in Figure 1H, although in reality the ethanolamine coordinates to the boron (B) atom to give a 5-membered ring cyclic structure. Also shown is the analog diphenylborinic anhydride (DPBA, **2**, the dimerized dehydrated form of diphenylborinic acid), diphenhydramine (DPH, **3**, a carbon analog of acyclic 2APB in which there is no possibility of a closed ring form), 2,2-diphenyltetrahydrofuran (DPTTF, **4**, which contains no B), and phenytoin (PHENY, **5**, which is similar to 2APB in that it has two phenyl groups attached to a heterocyclic ring, but again does not contain B). To test the effects of these analogs, we primed mouse peritoneal macrophages with LPS and then pre-treated with drug before stimulation with ATP. The drug was also present throughout the ATP stimulation. The effects of the drugs were normalized to ATP-induced IL-1β release in the absence of any drug (Figure 1I). The only analog to inhibit IL-1β release, in addition to 2APB, was the B-containing DPBA, with the other analogs having no effect (Figure 1I). The half-maximal inhibitory concentration (IC_50_) for the effects of 2APB on IL-1β release was 67 μM (Figure 1G).

These data suggest the B atom is essential for the inhibitory effects of 2APB on IL-1β release. To further test the requirement for B, we used computational similarity searching using ROCS (rapid overlay of chemical structures) (Grant et al., 1996) and Tanimoto scoring (ShapeTanimoto and ColorTanimoto, for shape and chemical similarity, respectively), to identify diverse commercially available carbon analogs of 2APB for screening. Several of the top ranked hits, selected on shape and pharmacophore match (from a library of ∼2 million non-B-containing compounds from the ZINC “LeadsNow” database, zinc.docking.org), were sourced via the repository of the NIH's National Cancer Institute (NCI) Developmental Therapeutics Program and were screened against ATP-induced IL-1β release using primary mouse bone marrow-derived macrophages (BMDMs). BMDMs were primed with LPS and incubated with vehicle (0.5% DMSO) or molecules (NCI1–7, **6–12**, Figure 1H) at 40 μM (to allow any enhanced inhibitory activity to be observed) for 15 min before ATP stimulation. The effects of the molecules were normalized to ATP-induced IL-1β release (Figure 1J). None of the carbon analogs were as effective as 2APB at this concentration, suggesting that the B atom is important for the inhibitory activity of 2APB.

### Refinement of the Structure-Activity Relationship

**Figure 2:**
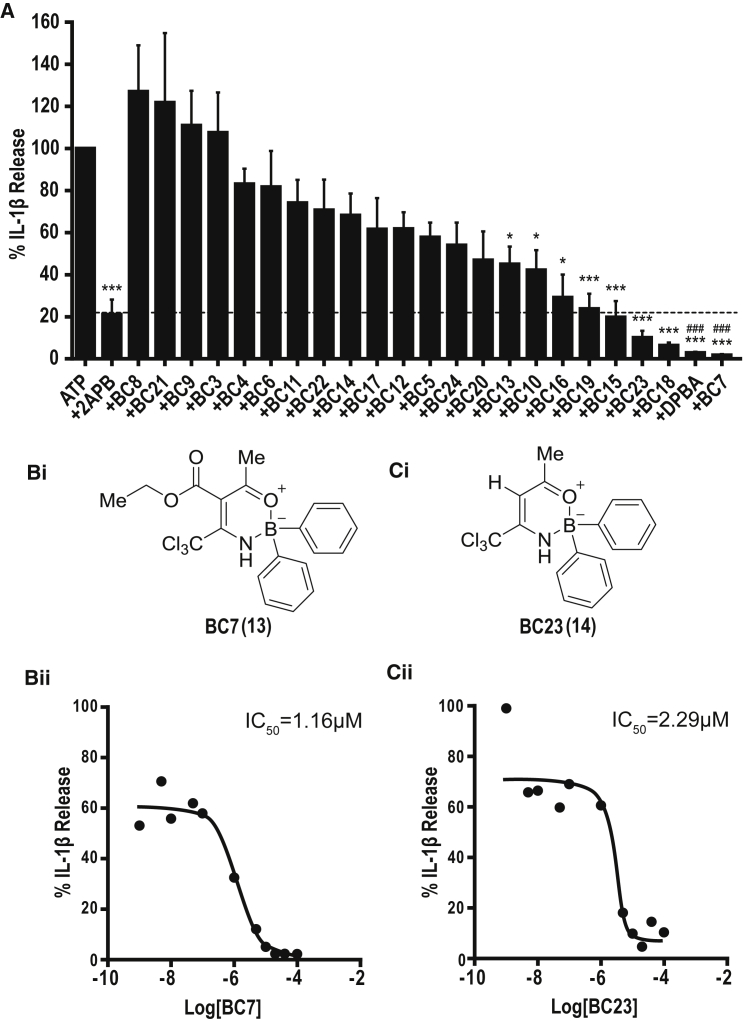
Identification of an Oxazaborine Ring in an Improved Pharmacophore Primary mouse BMDMs were primed with LPS (1 μg mL^−1^, 4 hr) and incubated with vehicle (0.5% DMSO) or molecules (BC1–24, Figure S1) at 40 μM for 15 min before ATP stimulation (5 mM, 1 hr). The effects of the molecules on IL-1β release were measured by ELISA and normalized to ATP-induced IL-1β release in the absence of any inhibitor (A). The chemical structures (i) and half-maximal inhibitory concentration curves (IC_50_, ii) for BC7 (B) and BC23 (C) are also presented using a 3-parameter logistical sigmoidal model. Data are presented as mean percentage of IL-1β release versus vehicle (DMSO) control + SEM of at least 3 experiments. *p < 0.05, ***p < 0.001, significant difference from 100% IL-1β release (Holm-Sidak corrected one-sample t test). ^###^p < 0.001, significant improvement from 2APB treatment (Holm-Sidak corrected post hoc comparison).

Given the apparent dependence on B, we screened a diverse library of commercially available B-containing compounds identified using SciFinder Scholar (called the BC series [for boron compound]) that shared some features and properties with 2APB (Figure S1). The BC molecules were screened against ATP-induced IL-1β release using primary mouse BMDMs as described above. Cells were primed with LPS and incubated with vehicle or molecules (BC1–24, Figure S1) before ATP stimulation. The effects of the molecules on IL-1β release were normalized to ATP-induced IL-1β release in the absence of any inhibitor (Figure 2A). Through this approach we identified analogs that were orders of magnitude more potent than 2APB at inhibiting IL-1β release (e.g., 2APB, IC_50_ = 67 μM; BC7 (**13**), IC_50_ = 1.2 μM; BC23 (**14**), IC_50_ = 2.3 μM; Figures 2B and 2C). Our preliminary qualitative structure-activity relationship (SAR) analysis identified the importance of the diarylborinic acid motif and an oxazaborine ring, with conformationally restricted analogs showing enhanced activity (Figure 2).

**Figure 3:**
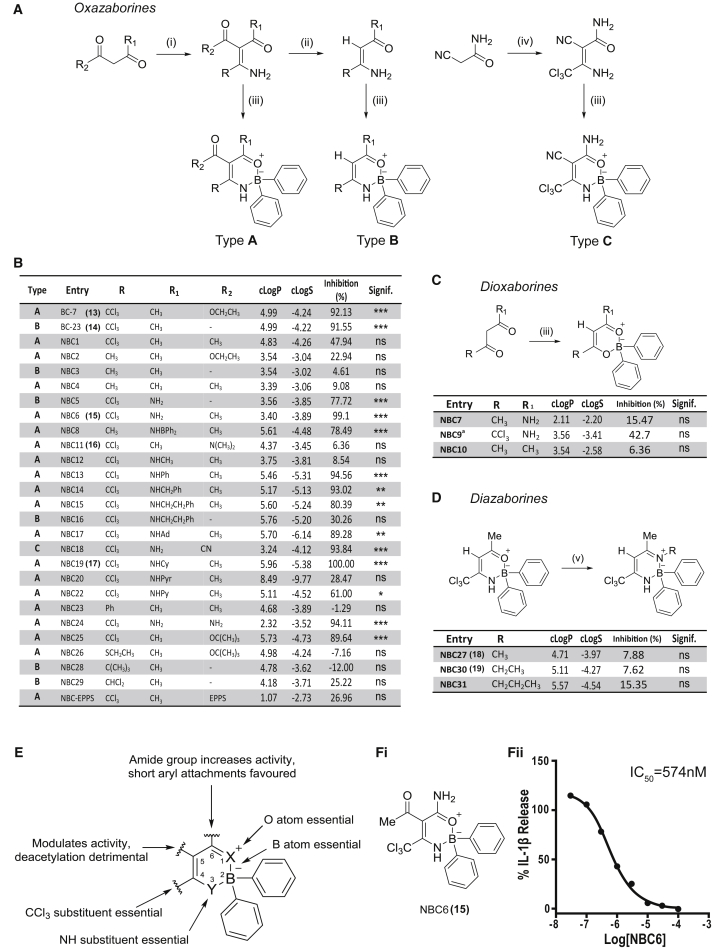
Refinement of the Structure-Activity Relationship (A) Pathway for oxazaborine syntheses. The method for the synthesis of the oxazaborine compounds are described as types A, B, and C. (i) RCN, Zn(acac)_2_/SnCl_4_, dichloromethane/toluene, room temperature to 80°C, 3–16 hr; (ii) K_2_CO_3(sat)_, EtOH, room temperature, 24 hr; (iii) DPBA, tetrahydrofuran (THF), 50°C, 16 hr; (iv) Cl_3_CCN, NaOAc, EtOH, room temperature, 16 hr (A). (B–D) Table of oxazaborines synthesized with structure type (A, B or C) identified (B). Ad, adamantyl; Cy, cyclohexyl; Pyr, pyrene; Py, pyridinyl; EPPS, 4-(2-hydroxyethyl)piperazine-1-propanesulfonic acid. Also shown in (B) to (D) is the percentage of inhibition of IL-1β release from LPS and nigericin-treated THP-1 cells with 10 μM inhibitor and the calculated cLogP and cLogS values for each compound. (C) Pathway for dioxaborine syntheses. (iii) DPBA, THF, 50°C, 16 hr. ^a^NBC9 was isolated as a by-product during NBC5 synthesis. (D) Pathway for diazaborine synthesis. (v) RNH_2_, THF, 50°C, 24 hr. (E) Summary of SAR analysis of NBCs. (F) Half-maximal inhibitory concentration curve (IC_50_) for NBC6 (Fi) is presented using a 3-parameter logistical sigmoidal model (n = 6) (Fii). *p < 0.05, **p < 0.01, ***p < 0.001, significant difference from 100% IL-1β release (Holm-Sidak corrected one-sample t test), n = 4. ns, not significant.

We then modified aspects of our lead BC molecules to improve activity and solubility, in addition to identifying the pharmacophore. Notably we modified the groups at each position of the oxazaborine ring (Figures 3A–3D), with atom numbering of the oxazaborine ring as shown in Figure 3E. A series of dioxa-, oxaza-, and diazaborines (novel boron compounds [NBC]1–31) based on the structures of BC7/23 were synthesized (for full details see Methods S1). In brief, 1,3-dicarbonyls were reacted with acetonitrile derivatives in the presence of a metal catalyst (Zn(acac)_2_ or SnCl_4_) to yield acetylated enaminones, by adapting previously reported methods (Veronese et al., 1986, Singh and Lesher, 1978). These intermediates were readily deacetylated by treating with K_2_CO_3_ (Veronese et al., 1986). Subsequent borylation of these enaminones using DPBA afforded the corresponding oxazaborine NBC molecules using a method similar to that previously described (Vasil'ev et al., 1992, Dorokhov et al., 1995) (Figure 3A). The synthesis of oxazaborine NBC18 used similar chemistry, except that the starting material was cyanoacetamide (Figure 3A). Dioxaborines were synthesized by directly borylating 1,3-dicarbonyls (Bally et al., 1965) (Figure 3C). Reaction of BC23 with a range of alkyl amines yielded diazaborines (Figure 3D) adapted from Vasil'ev et al. (2013). Reaction of BC23 with ammonia did not give the expected diazaborine product, and only the dechlorinated compound containing a CHCl_2_ group (NBC29) was isolated. cLogP and cLogS calculations were performed for BC7, BC23, NBC1–31, and NBC-EPPS (Figure 3B), and demonstrate that a number of potent NBC molecules (NBC6, 18, 24) have improved physicochemical properties compared with the original lead compounds BC7 and BC23. Furthermore, an experimental LogS value for our lead analog NBC6 was found to be −1.63 in MeOH (9.7 mg mL^−1^) (data not shown). These data suggest that the NBC molecules are sparingly soluble in aqueous solution.

**Figure 4:**
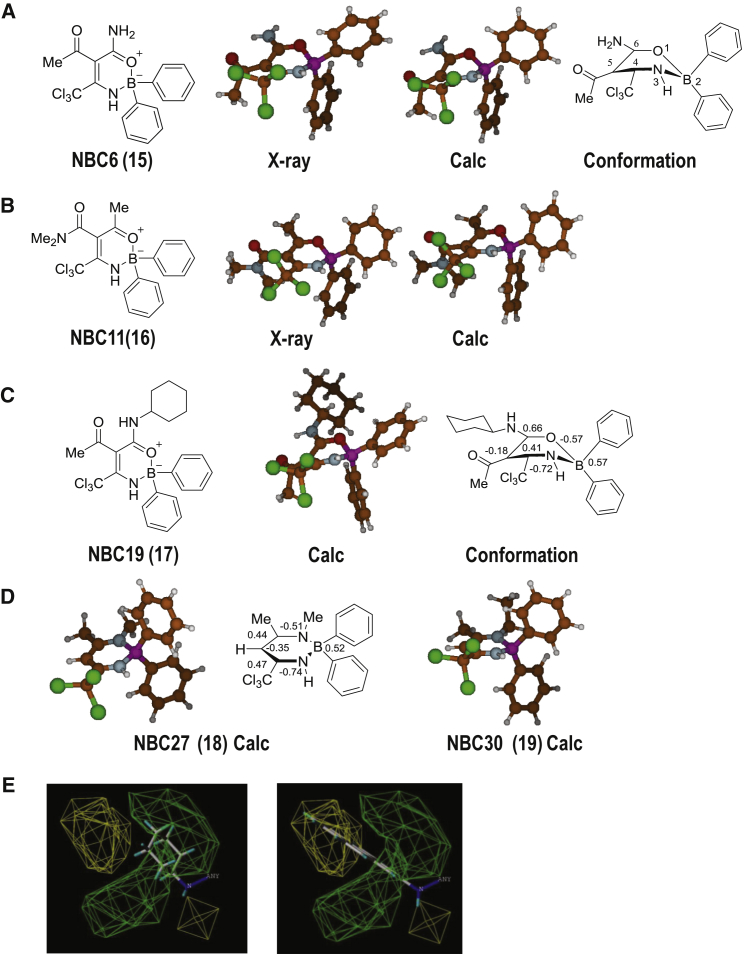
X-Ray Crystallography and Computational Modeling of NBCs (A and B) Crystal (X-ray) and predicted structure (Calc) of NBC6 (**15**) (A) and NBC11 (**16**) (B), calculated at the M06-L/6-31G* level of theory. The ring is boat-like in conformation (atom numbering shown in A). (C) Computed structure and Mulliken partial charges on ring atoms of oxazaborine NBC19 (**17**). (D) Computed structures of NBC27 (**18**) and NBC30 (**19**) illustrating the planarity of the diazaborine ring and Mulliken partial charges of NBC27 and NBC30. (E) Steric field arising from topomer CoMFA of 24 oxazaborine compounds, superimposed on structures of (left) NBC19 and (right) NBC20.

To screen the NBC series we used the human monocytic THP-1 cell line, since these cells would allow a higher throughput compared with the primary cells used above. Cells were primed with LPS and then treated with vehicle or NBC molecule (NBC1–31) at 10 μM for 15 min before activation of the inflammasome and IL-1β release with nigericin. The NBC molecule was present throughout nigericin stimulation. The effects of the molecules on IL-1β release were normalized to nigericin-induced IL-1β release in the absence of any inhibitor (Figure 3B). These data showed some additional features of the SAR (Figures 3B and 3E). Any substitution of the -CCl_3_ group at position 4 (Figure 3E) was detrimental to bioactivity: its electronic character was important for inhibition of IL-1β release, confirmed by the substantially reduced activities of the isosteric ^t^Bu (NBC28) and phenyl (NBC23) analogs. A *bis*-oxazaborine chelate (NBC8) was the only analog lacking the CCl_3_ group that showed good bioactivity, which may be attributed to the extra BPh_2_ group in the structure. Modifications of the oxazaborine ring (O-B-N bonding) to a dioxaborine (O-B-O) or diazaborine (N-B-N) ring structure was also detrimental to bioactivity. An oxazaborine ring structure was required, as the NBC6 enaminone intermediate prior to borylation (NBC6i, without B) was inactive (not shown). Modifications at the 5-position were generally tolerated, and typically molecules containing a carbonyl group-containing moiety showed enhanced bioactivity (Figures 3B and 3E). However, the ester NBC-EPPS analog containing the buffer 4-(2-hydroxyethyl)-1-piperazinepropanesulfonic acid (EPPS) was less active due to the substituent chain length either being too long or too hydrophilic. Deacetylation at position 5 (e.g., NBC6 cf. NBC5) leads to a loss in bioactivity (Figure 3E), so an acetyl group was preferentially retained in this position. Changing the methyl group at position 6 modulated bioactivity (Figure 3E), with substitution to the primary amide analog noticeably enhancing inflammasome inhibition (NBC6, **15**) (Figure 3B). Changing the primary amide to a secondary amide improved bioactivity as long as the substituents were hydrophobic (alkyl or aromatic) and the chain length of the substituent was not too short (R_1_ = Me, NBC12) or too bulky (R_1_ = pyrene, NBC22). Full substitution of the primary amide (NBC6) to the *N*,*N*-dimethyl tertiary amide derivative (NBC11, **16**) lost bioactivity, potentially due to a change in chelation (see X-ray in Figures 4A and 4B). It was interesting to note that secondary amide derivatives containing either phenyl (NBC13) or cyclohexyl (NBC19, **17**) substitutions are both active, showing that the ring can be either unsaturated or saturated. In summary, an oxazaborine scaffold and a CCl_3_ group on the 4-position was required to inhibit IL-1β release (Figure 3E). From the screen of NBC molecules, NBC6 was most potent and more drug-like compared with leads BC7/23, and further analysis revealed increased potency with an IC_50_ of 574 nM (Figure 3F).

### X-Ray Crystallography and Computational Modeling of NBCs

When borylating the enaminone intermediates using DPBA, the oxazaborine product could adopt a number of different B chelate structures. For example, NBC6 and NBC11 could chelate to B through the NH of the enamine and either the amide or ketone C=O. To determine the structures of the chelates in the solid phase, crystals of NBC6 and NBC11 were grown in *n*-hexane/toluene (1:1) and X-ray crystallographic analysis was undertaken. The *R* factors obtained for NBC6 and NBC11 were 4.08% and 2.83%, respectively. The B atom lies out of the ring plane in a boat-envelope conformation in both oxazaborine structures, whereas the other atoms in the heterocycle are planar and are involved in a π-electron conjugated system (Figure 4A) as previously reported for other oxazaborines (Josefíka et al., 2012, Mikyseka et al., 2017). NBC6 is chelated to B through O/N chelation of the amide C=O and enamine NH (Figure 4A), whereas NBC11 is chelated to B through O/N chelation of the ketone C=O and enamine NH (Figure 4B). Thus full substitution of the primary amide (NBC6) to an *N*,*N*-dimethyl tertiary amide (NBC11) has induced a change in B chelation, which could explain the large difference in observed bioactivity between these two oxazaborines (Figure 3B).

Computational modeling was applied to further investigate the impact of the shape and electronic properties of the NBCs on the bioactivity and observed SAR. All of the compounds were energy optimized at the M06-L/6-31G* level of quantum mechanics (Zhao and Truhlar, 2006). Consistent with the X-ray crystal structures, computational modeling predicted that the rings in the oxazaborines adopted boat-envelope conformations (NBC6 [Figure 4A] and NBC11 [Figure 4B]). Indeed, the agreement in ring pucker of the 6-membered ring between calculation and the X-ray structures was very good: for NBC6, the mean unsigned ring pucker torsion <ν> differs by 2.1° while for NBC11 the difference is 5.7° (Table S1). There was good agreement with experiment in the use of M06 functionals to model coordinate B-N bond lengths and bond enthalpies of methyl-substituted aminoboranes (Janesko, 2010). For NBC6 and NBC11, the average experimental pucker <ν> was 25.4° and 21.1°, respectively (Table S1). This indicates significant deviation from planarity; the greatest pucker for both rings centered around the B atom, with a ν_5_ angle of −45.4° and −37.3°, respectively (Table S1). Computed bond distances within the ring also reproduce the experiment well, with a maximum deviation from the crystallographic values of 0.02 Å for NBC6 and 0.01 Å for NBC11 (Table S1). The largest difference in ring bond distance between NBC6 and NBC11 is found for the C_5_-C_6_ bond, with predicted and experimental distances agreeing on a 0.05-Å larger bond distance in NBC6 (Table S1). In contrast, energy optimization of the NBC diazaborine compounds, containing the N-B-N motif, predict at the M06-L/6-31G* level a planar diazaborine ring (e.g., NBC27 [**18**], and NBC30 [**19**], Figure 4D). The planarity is reflected by a mean unsigned pucker angle <ν> of 3.3° and 11.4° for NBC27 and NBC30, respectively (Table S1), distinctly lower than the values for NBC oxaborines, which exceed 20°. Furthermore, the N-B-N angle in these compounds are 112.5° and 111.2° for NBC27 and NBC30, respectively, compared with O-B-N angles of 102.6° and 104.0° in the NBC6 and NBC11 X-ray structures (θ, Table S1). These geometric features reflect the more aromatic character of the diazaborine systems.

Further insight into electronic distribution was obtained from Mulliken population analysis: firstly we note that the B atom is predicted to possess a positive partial charge in all of the NBC compounds analyzed (Figures 4C and 4D; Table S1). The charge on the B atom is on average 0.59 *e* for the 24 O-B-N compounds, 0.60 *e* for 3 O-B-O compounds, and somewhat less for the 3 N-B-N compounds, with an average value of 0.51 *e*. This reflects the low Pauling electronegativity of the B atom, reported as 2.04 compared with values of 3.04 for N and 3.44 for O (Allen, 1989). Thus, although we traditionally represent 4-coordinate B atoms with a formal negative charge (e.g., Figure 3), quantum chemical analysis predicts that the B atom in the heterocycle of these NBC compounds possesses a partial positive charge. The magnitude of the partial atomic charge on carbon C_6_, q(C_6_) is higher for NBC6 and NBC19 (>0.6 *e*) compared with less active compounds NBC11, NBC27, and NBC30 (<0.5 *e*, Table S1). For oxazaborines, a higher value of q(C_6_) appears to be due to the presence of an amido substituent at C_6_. The correlation of q(C_6_) and observed activity of the compounds is somewhat modest, with a correlation coefficient *r*^2^ of 0.5 (a similar correlation is found for the C_5_-C_6_ bond distance); this reflects the influence of other factors, in particular steric constraints on substituents. 3D-QSAR was performed using the topomer comparative molecular field analysis (CoMFA) method (Cramer, 2003), based on oxazaborines NBC1–6, 8, 11–20, 22–26, and 28–29 compounds (*r*^2^ of 0.71). Again, our analysis highlights the steric volume required for activity at position 6 (Figure 4E), and indicates the complementarity of the cyclohexyl substituent of NBC19 compared with the weakly active NBC20 compound, which presents a bulkier pyrene group at C_6_ (Figure 4E). In summary, the density functional calculations agree well with crystallographically determined structures and indicate a shape and electronic character of the oxazaborine ring that is distinct from the planar, aromatic diazaborine ring, suggesting that these features are responsible for the activity of the oxazaborines. Within the oxazaborine series, there is evidence of a specific steric constraint on substituents at position 6 of the ring.

### Mechanism of Action

**Figure 5:**
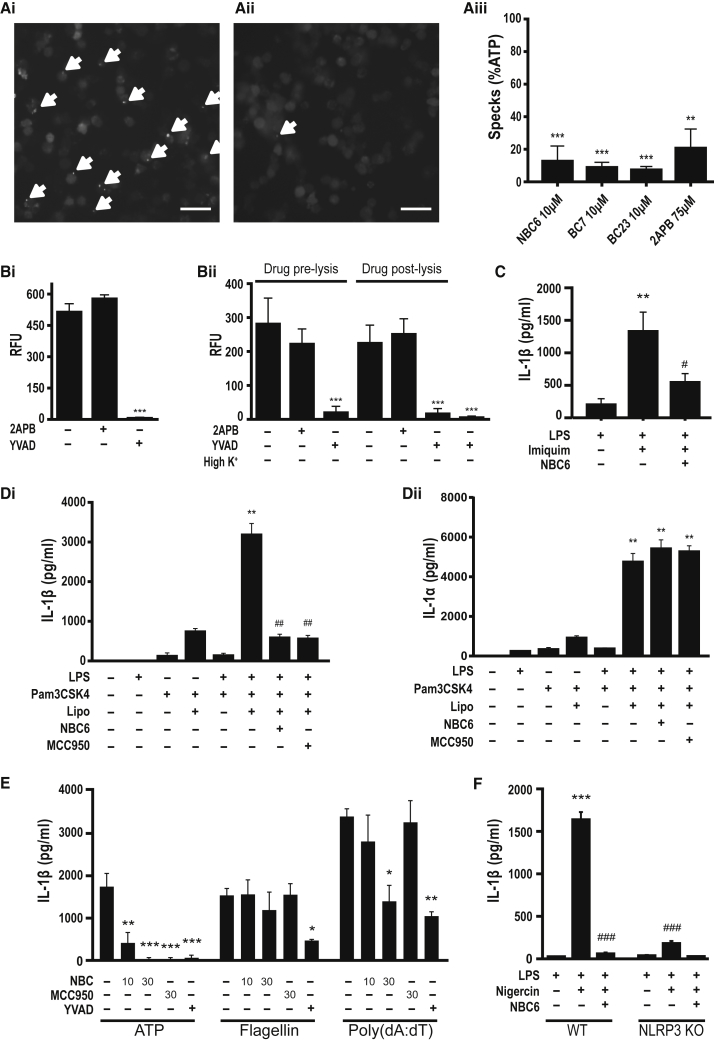
NBCs Are Effective NLRP3 Inflammasome Inhibitors (A) The effects of 2APB, BC7, BC23, and NBC6 on ASC speck formation following ATP stimulation were measured. iBMDMs stably expressing ASC protein conjugated to mCherry were primed with LPS (1 μg mL^−1^, 2 hr), then pre-treated with selected drug (indicated concentration, 15 min) before stimulation with ATP (5 mM, 30–45 min) under live microscopy. Formation of ASC specks (examples indicated by white arrows, Ai [no drug], Aii [plus NBC6]) were quantified (Aiii) and presented as mean percentage of specks counted versus vehicle + SEM (n = 5–6). **p < 0.01, ***p < 0.001, significant difference from 100% speck formation (Holm-Sidak corrected one-sample t test, n = 5–6). Scale bars, 20 μm. (B) Recombinant caspase-1 (10 U mL^−1^) was incubated with 0.5% DMSO, YVAD (100 μM), or 2APB (75 μM) before addition of the fluorogenic substrate Z-YVAD-AFC. Caspase-1 activity was measured 2 hr later (Bi) (***p < 0.001, significant difference from vehicle control, Holm-Sidak corrected post hoc comparison, n = 4). Hypotonic THP-1 cell lysate assay was also used to measure the effects of 2APB on caspase-1 activity. 2APB (75 μM) was added to the cells just prior to, or following, lysis in hypotonic buffer. The lysate was incubated with Z-YVAD-AFC and caspase-1 activity measured 2 hr later (Bii). YVAD or high K^+^ concentration were included as controls (Bii) (***p < 0.001, significant difference from relevant lysis vehicle control, Holm-Sidak corrected post hoc comparison, n = 4). (C) LPS-primed (1 μg mL^−1^, 4 hr) mouse primary BMDMs were treated with NBC6 (10 μM) or vehicle (DMSO) 15 min prior to 1 hr treatment with small-molecule NLRP3 activator imiquimod (70 μM) or DMSO control. Imiquimod significantly induced IL-1β release (**p < 0.01) and this was inhibited by NBC6 treatment (^#^p < 0.05, Holm-Sidak corrected post hoc comparison, n = 4). (D) Mouse primary BMDMs were primed with Pam3CSK4 (100 μg mL^−1^, 4 hr) followed by 15 min NBC6 (1 μM), MCC950 (1 μM), or vehicle, then treated with intracellular LPS (2 μg mL^−1^, transfected with Lipofectamine 3000, 24 hr) or Lipofectamine alone (**p < 0.01, significant induction of IL-1β [Di] or IL-1α [Dii] release versus Lipofectamine-alone control; ^##^p < 0.01, significant inhibition of IL-1β release; Holm-Sidak corrected post hoc comparison, n = 4). (E) Mouse primary BMDMs were primed with LPS (1 μg mL^−1^, 4 hr) followed by 15 min NBC6 (10 and 30 μM), MCC950 (30 μM), YVAD (100 μM), or vehicle, then treated with canonical NLRP3 activator ATP (5 mM, 1 hr), NLRC4 activator (flagellin, 667 ng mL^−1^, transfected with Lipofectamine 3000), or AIM2 activator (poly(dA:dT), 667 ng mL^−1^, transfected with Lipofectamine 3000) (*p < 0.05, **p < 0.01, ***p < 0.001, significant inhibition of IL-1β release, Holm-Sidak corrected post hoc comparison, n = 3). (F) Mouse primary bone marrow neutrophils from WT and NLRP3 KO mice (n = 4) were primed with LPS (1 μg mL^−1^, 2 hr), then NBC6 (10 μM) was added 15 min prior to the addition of nigericin (10 μM), which significantly induced IL-1β release (***p < 0.001), which was inhibited by NBC6 treatment (^###^p < 0.001, Holm-Sidak corrected post hoc comparison). Data are presented as the mean + SEM.

We measured the effects of 2APB, BC7, BC23, and NBC6 on ASC speck formation following ATP stimulation. Immortalized (i)BMDMs transduced with a lentiviral vector to express ASC-mCherry (Daniels et al., 2016) were treated with LPS and then stimulated with ATP for between 30 and 45 min with ASC speck formation measured as described previously (Daniels et al., 2016). 2APB was an effective inhibitor of ASC speck formation, as were BC23 and NBC6 (Figure 5A). Additionally, we showed that 2APB is not a direct inhibitor of caspase-1. Recombinant caspase-1 was incubated with vehicle, YVAD, or 2APB before addition of the fluorogenic substrate Z-YVAD-AFC. Caspase-1 activity was measured 2 hr later. Under these conditions 2APB had no effect on caspase-1 activity while YVAD caused complete inhibition (Figure 5Bi). We also used a hypotonic THP-1 cell lysate assay to measure the effects of 2APB on caspase-1 activity. 2APB was added to the cells just prior to, or following, lysis in hypotonic buffer. The lysate was incubated with the caspase substrate Z-YVAD-AFC, which in addition to caspase-1 would also be cleaved by caspase-4 and -5, and caspase activity was measured 2 hr later. 2APB had no effect on caspase-1 activity under these conditions, whereas caspase-1 activity was completely inhibited by YVAD or high K^+^ concentration (Figure 5Bii). An important step in the activation of NLRP3 is K^+^ efflux from the cell (Munoz-Planillo et al., 2013). It was reported recently that 2APB does not inhibit K^+^ efflux (Katsnelson et al., 2015), so the effect of 2APB must be downstream of K^+^ efflux and before caspase-1 activity. Recently additional mechanisms of NLRP3 activation have been reported, including by the small molecule imiquimod, which is suggested to be independent of K^+^ efflux (Gross et al., 2016). In LPS-primed primary BMDMs, NBC6 also inhibited imiquimod-induced IL-1β release (Figure 5C). We also tested whether NBC6 could inhibit IL-1β secretion through the non-canonical NLRP3 pathway. Priming with the TLR2 agonist Pam3CSK4 followed by LPS transfection stimulates the activation of NLRP3 via the non-canonical caspase-11-dependent pathway (Kayagaki et al., 2013). Primary BMDMs were primed with Pam3CSK4 followed by transfection with LPS (Kayagaki et al., 2013). Neither NBC6 nor MCC950 inhibited the release of IL-1α (Figure 5Dii), which occurs due to caspase-11-dependent pyroptosis independently of NLRP3 (Kayagaki et al., 2011). However, NBC6 and, as previously reported (Coll et al., 2015), MCC950 did inhibit the release of IL-1β (Figure 5Di). These data suggest that NBC6 can also inhibit NLRP3 via the non-canonical pathway. To determine whether the NBCs were selective inhibitors of NLRP3-dependent IL-1β release, we tested their effects against other well-characterized inflammasomes in primary wild-type (WT) and NLRP3 knockout (KO) BMDMs. LPS-primed WT BMDMs were treated with ATP in the absence and presence of NBC6 (10 and 30 μM), the established NLRP3 inhibitor MCC950 (Coll et al., 2015) (30 μM), and the caspase-1 inhibitor YVAD (100 μM). Under these conditions, all inhibitors inhibited the release of IL-1β (Figure 5E). In NLRP3 KO BMDMs a similar format was followed except that NLRC4 inflammasome activation was induced by transfection of *Salmonella typhimurium* flagellin, whereby this time 10 and 30 μM NBC6 and 30 μM MCC950 had no effect (Figure 5E). The same format was followed for AIM2 inflammasome activation whereby LPS-primed NLRP3 KO BMDMs were transfected with poly(dA:dT). Again 10 μM NBC6 and 30 μM MCC950 had no effect and YVAD inhibited IL-1β release, as did 30 μM NBC6 (Figure 5E). These data suggest that NBC6 selectively inhibits NLRP3 at low doses but may also be effective against other inflammasomes at higher doses. To further establish that NBC6 inhibits NLRP3 across cell types, neutrophils were isolated from WT and NLRP3 KO murine bone marrow and primed with LPS followed by nigericin treatment in the presence or absence of 10 μM NBC6. From this we observed complete inhibition of NLRP3-dependent IL-1β release from NBC6-treated neutrophils (Figure 5F).

**Figure 6:**
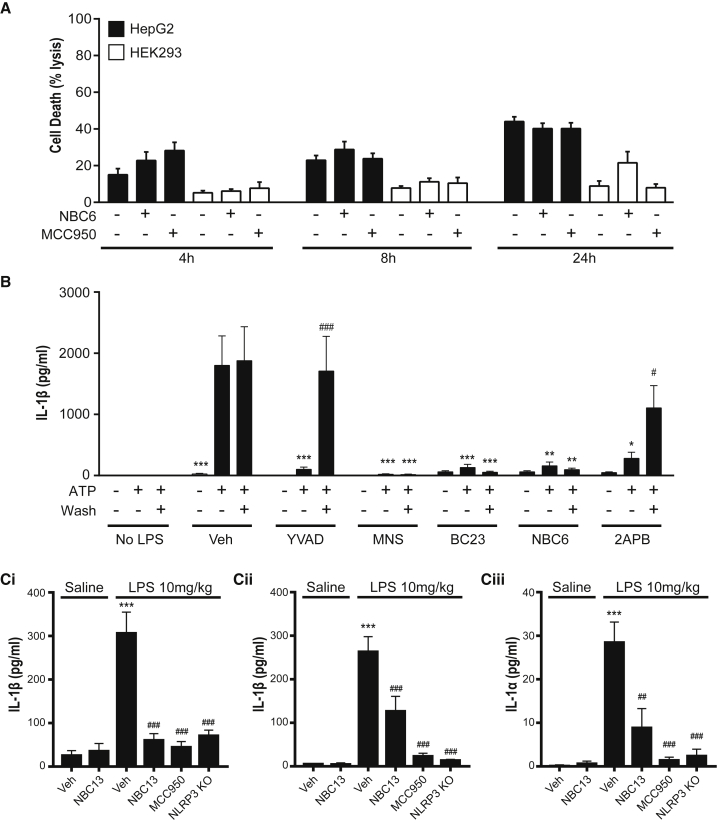
NBCs Are Effective against NLRP3 *In Vivo* (A) HEK293 or HepG2 cells were treated with NBC6 (10 μM), MCC950 (10 μM), or DMSO for 4 hr, 8 hr, and 24 hr. Cell death was measured by lactate dehydrogenase release and expressed as percentage lysis control. No significant effects were observed (two-way repeated-measures ANOVA). (B) LPS-primed (1 μg mL^−1^, 2 hr) iBMDMs were pre-treated with drugs (BC23, NBC6, 30 μM; MNS, 100 μM; YVAD, 100 μM; 2APB, 75 μM) or vehicle (DMSO) in serum-free media for 15 min and washed 3 times, before inflammasome activation was initiated by adding ATP (5 mM) for 1 hr. IL-1β release was measured by ELISA (*p < 0.05, **p < 0.01, ***p < 0.001, significant inhibition of IL-1β release compared with vehicle-ATP control; ^#^p < 0.05, ^###^p < 0.001, significant effect of washing compared with no-wash drug-ATP control, Holm-Sidak corrected post hoc comparison, n = 5–6). (C) C57BL/6 and NLRP3 KO mice (n = 6) were injected intraperitoneally with LPS (10 mg kg^-1^, 3 hr). Separate groups of WT animals receiving LPS were also given a 50 mg kg^1^ dose of MCC950 or NBC13. IL-1β in peritoneal lavage (Ci) and plasma (Cii) was measured by ELISA. IL-1α in plasma was measured by ELISA (Ciii). ***p < 0.001, significant difference from saline vehicle control; ^##^p < 0.01, ^###^p < 0.001, significant difference from LPS vehicle group (Holm-Sidak corrected post hoc comparison). Data are presented as the mean + SEM.

We next compared the toxicity of NBC6 with that of MCC950 in kidney (HEK293) and liver (HepG2) cell lines. Neither drug showed any toxicity up to 24 hr of incubation (Figure 6A). To further refine the mechanism of action of NBCs on NLRP3, we sought to determine the reversibility of NBC inhibition of NLRP3-dependent IL-1β release. iBMDMs were primed with LPS and then incubated with the reversible caspase-1 inhibitor YVAD, the irreversible NLRP3 inhibitor 3,4-methylenedioxy-β-nitrostyrene (MNS; He et al., 2014), NBC6, BC23 (from the BC series), and 2APB for 15 min before 3 washes over 15 min to remove unbound drug. Cells were then stimulated with ATP (5 mM) to activate NLRP3-dependent IL-1β release. As expected, YVAD washed out and MNS did not. The effects of NBC6 and BC23 resembled that of MNS and were irreversible over the time course of the experiment (Figure 6B). Interestingly 2APB was reversible (Figure 6B). To test whether the NBC molecules could inhibit NLRP3-dependent inflammation *in vivo*, we used a previously reported model of peritonitis (Coll et al., 2015). Previously, mice injected intraperitoneally with LPS showed increased IL-1β in the lavage fluid that was inhibited by the NLRP3 inhibitor MCC950 (Coll et al., 2015). Thus we injected WT and NLRP3 KO mice with LPS. Separate groups of animals receiving LPS were also given a dose of MCC950 or NBC13, chosen because it had similar potency to NBC6 at inhibiting IL-1β release and was soluble in corn oil, which was used to deliver the drug. LPS induced an increase in IL-1β in the lavage and plasma in WT mice but not NLRP3 KO mice, supporting the NLRP3 dependence of this response (Figure 6C). MCC950, as expected, also inhibited LPS-induced IL-1β production in the peritoneum, as did NBC13, suggesting that NBCs are effective NLRP3 inhibitors *in vivo* (Figure 6Ci). LPS also caused an increase in plasma IL-1β, which was also absent in NLRP3 KO mice and was completely inhibited by MCC950 (Figure 6Cii). NBC13 significantly inhibited LPS-induced plasma increases in IL-1β but was not quite as effective as MCC950, possibly due to reduced exposure or potency (Figure 6Cii). Both NBC13 and MCC950 significantly inhibited the production of the related cytokine IL-1α in the plasma (Figure 6Ciii). Together, these data show that the NBCs are effective inhibitors of the NLRP3 inflammasome and can also target NLRP3-dependent inflammation *in vivo*.

### Ca^2+^-Independent Effects of the NBCs

**Figure 7:**
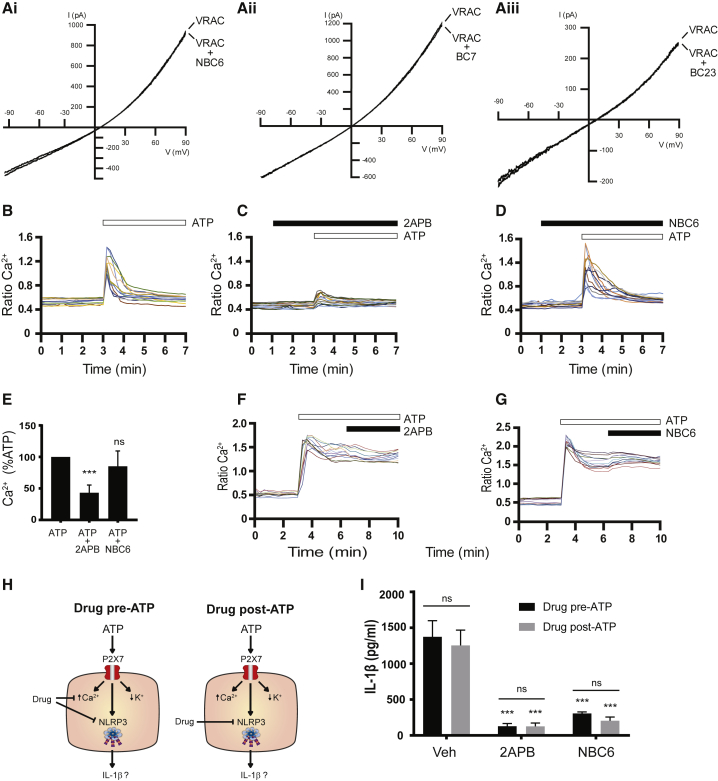
Ca^2+^-Independent Effects of the NBCs (A) To induce volume-regulated Cl^−^ currents (VRAC), LPS-primed (1 μg mL^−1^, 2 hr) iBMDMs were superfused with hypotonic solution. Representative current traces are shown, which have been measured in the absence (VRAC) or presence of 30 μM NBC6 (Ai), 30 μM BC7 (Aii), or 30 μM BC23 (Aiii). (B–E) LPS-primed (1 μg mL^−1^, 2 hr) iBMDMs were kept untreated or were pre-treated for 2 min with 75 μM 2APB or 30 μM NBC6. Subsequently, 100 μM ATP was added to the bath solution. (B–D) Representative Ca^2+^ traces of ATP-stimulated cells in the absence (B, n = 12) or presence of 75 μM 2APB (C, n = 14), or 30 μM NBC6 (D, n = 12). (E) Mean peak Ca^2+^ concentrations determined in cells treated with ATP alone (ATP) or with ATP in the presence of inhibitors. ns, no significant difference; ***p < 0.001, significant difference from Ca^2+^ signals of ATP-stimulated cells determined in the absence of inhibitors (Holm-Sidak corrected one-sample t test). (F and G) LPS-primed iBMDMs were also stimulated with 5 mM ATP. Following development of sustained Ca^2+^ increases, 75 μM 2APB or 30 μM NBC6 was added to the ATP-containing solution. Images show representative examples of Ca^2+^ responses following treatment with ATP and the addition of 2APB (n = 12, F) or the addition of NBC6 (n = 12, G). (H and I) LPS-primed iBMDMs were treated with concentrations of the inhibitors maximal for blocking IL-1β release (i.e., 2APB = 75 μM, NBC6 = 30 μM). Inhibitors were added to the iBMDMs 5 min before or 5 min after the addition of ATP (5 mM, 1 hr) (H) with IL-1β release measured by ELISA (I). ***p < 0.001, significant difference from corresponding vehicle control; ns, no significant effect of ATP administration time. Data are presented as representative traces from calcium imaging experiments (B–D, F, and G), mean + SEM peak Ca^2+^ concentrations versus treatment with ATP alone (E), or mean + SEM IL-1β release as detected by ELISA (I).

We recently reported that the fenamates were effective inhibitors of the NLRP3 inflammasome due to inhibition of Cl^−^ efflux through the volume-regulated anion channel (VRAC) (Daniels et al., 2016). Thus we tested the effects of BC23, BC7, and NBC6 on VRAC. VRAC currents measured by whole-cell patch clamp in LPS-primed iBMDMs were induced by hypotonicity. BC7, BC23, and NBC6 had no effect on VRAC (Figure 7A). As described above, 2APB is known to modulate Ca^2+^ homeostasis. Thus we tested the ability of 2APB and NBC6 to modify intracellular Ca^2+^ in LPS-treated iBMDMs stimulated with ATP. The cells were pre-treated with doses of the inhibitors maximal for blocking IL-1β release (i.e., 2APB 75 μM, NBC6 30 μM). The cells were then stimulated with 100 μM ATP (submaximal for P2X7 but saturating for other purinergic receptors) to induce increases in [Ca^2+^]_i_, which were measured using the ratiometric Ca^2+^ indicator Fura-2 (Figures 7B–7D). ATP induced a transient increase in [Ca^2+^]_i_ that was inhibited by 2APB but was not blocked by NBC6 (Figures 7B–7E), suggesting that the NBC compounds did not affect [Ca^2+^]_i_ changes dependent upon InsP_3_ receptor activation. iBMDM cells were then treated with LPS and stimulated with 5 mM ATP to activate the P2X7 receptor and induce activation of NLRP3. Under these conditions there was a marked and sustained increase in [Ca^2+^]_i_ (Figures 7F and 7G). Inhibitor (2APB 75 μM, NBC6 30 μM) applied to the cells 3 min after ATP did not affect [Ca^2+^]_i_, again supporting that the effects of 2APB and the NBC compounds are acting independently of effects on Ca^2+^ (Figures 7F and 7G). To correlate with this Ca^2+^ experiment, inhibitors were added to the iBMDMs following ATP stimulation as described above and effects on IL-1β release were measured 1 hr later by ELISA (Figures 7H and 7I). Adding drug after stimulation of the large Ca^2+^ increase inhibited inflammasome activation and IL-1β release as effectively as the pre-incubation (Figures 7H and 7I). Together, these data suggest that the inhibitors are acting independently of any effects on Ca^2+^ and that the effect on Ca^2+^ for the NBC compounds is not significant.

## Discussion

The NLRP3 inflammasome contributes to inflammatory diseases and is therefore an important therapeutic target (Lamkanfi and Dixit, 2012). Increasing recognition of the contribution of NLRP3 to disease has led to efforts to develop small-molecule inhibitors (Baldwin et al., 2016). Here we report the development of a unique B-based pharmacophore that inhibits NLRP3-dependent inflammation in both *in vitro* and *in vivo* models. Boron is an unusual element to be present in drug leads; bortezomib (Velcade) is the only B-containing drug used clinically. After establishing that B was essential for inhibition through screening of carbon analogs, we synthesized a range of new B-based inhibitors of NLRP3 derived from the early leads 2APB, BC7, and BC23, the most potent compound having an IC_50_ value of 574 nM for the inhibition of release of IL-1β from THP-1 monocytes.

During the preparation of the oxazaborines, for the synthesis of acetylated enaminone intermediates, we found that the choice of the metal catalyst was critical; Zn(acac)_2_ was amenable with good electrophiles (e.g., trichloroacetonitrile and benzonitrile); however, the stronger catalyst SnCl_4_ was required with non-electrophilic, weakly activating nitriles (e.g., acetonitrile and *tert*-butyl acetonitrile). The oxazaborines synthesized herein can be handled easily at room temperature, in contrast to the facile hydrolysis observed for 2APB (Hofer et al., 2013). Molecular modeling calculations agreed well with the X-ray crystal structures of NBC6/11, demonstrating the robustness of predictions using quantum mechanics. This is the first time that biological screening of oxaza-, dioxa-, and diazaborines has been reported. The SAR of the 31 NBC molecules revealed interesting key features required for bioactivity, with the oxazaborine ring and CCl_3_ group being essential pharmacophores for NLRP3 inflammasome inhibition.

NLRP3 is composed of three domains: an N-terminal pyrin domain for homotypic interaction with the pyrin domain of the adaptor ASC, a central NACHT domain that binds nucleotides, and a C-terminal leucine-rich repeat domain that senses the PAMPs or DAMPs. The mechanisms regulating the activation of NLRP3 are currently the focus of a major research effort in the field and are still being elucidated. There is very limited evidence for an interaction between PAMP/DAMP and NLRP3; instead these activating stimuli activate a common pathway dependent upon K^+^ efflux (Munoz-Planillo et al., 2013). Recently, the protein NEK7 has been identified as an interacting partner of NLRP3 required for its activation (Schmid-Burgk et al., 2016, Shi et al., 2016), and this interaction is also downstream of K^+^ efflux (He et al., 2016). In addition, ubiquitination and deubiquitination are also becoming established as essential steps (Lopez-Castejon et al., 2013, Juliana et al., 2012, Py et al., 2013). There has also been substantial literature to support an involvement of Ca^2+^ in inflammasome activation. Many of the studies reporting Ca^2+^ dependence of NLRP3 activation have been based on, or involved the use of 2APB as an inhibitor of Ca^2+^ signaling (Lee et al., 2012, Murakami et al., 2012, Compan et al., 2012, Rossol et al., 2012), or intracellular Ca^2+^ chelators such as BAPTA-AM (Brough et al., 2003). However, there is now evidence that both of these experimental manipulations may inhibit the inflammasome independently of effects on Ca^2+^ (Katsnelson et al., 2015). Further evidence for an effect independent of Ca^2+^ is provided in Figure 1, and is related to the work of Dobrydneva and Blackmore (2001) on store-operated Ca^2+^ entry. While they showed that DPBA inhibits Ca^2+^ entry (and IL-1β release presented here), DPTTF, which had no effect on IL-1β release in our study, inhibited Ca^2+^ entry as effectively as DPBA (Dobrydneva and Blackmore, 2001). Such data further support that the effects of 2APB on IL-1β processing and release are independent of Ca^2+^. Here we also show that while 2APB effectively inhibits increased [Ca^2+^]_i_ in response to 100 μM ATP, NBC6 does not, even at concentrations supra-maximal for the inhibition of IL-1β secretion (Figure 7). Furthermore, addition of 2APB, or NBC6 after the addition of 5 mM ATP, an NLRP3-activating stimulus, did not modify [Ca^2+^]_i_ dynamics, but did still inhibit IL-1β secretion following this protocol (Figure 7). These data suggest strongly that the effects of 2APB on IL-1β release are independent of Ca^2+^, and that we have deselected this property in our NBC molecules. This effectively provides a new and unique chemical scaffold for the development of NLRP3-inhibiting drugs that do not have the potentially harmful off-target effects on Ca^2+^ homeostasis.

## Significance

**Excellent evidence now points toward NLRP3 as an important therapeutic target for multiple major diseases (****Guo et al., 2015**)**. There are no drugs available clinically that specifically target NLRP3, although we (****Daniels et al., 2016****) and others (****Fowler et al., 2014****) have shown that some existing drugs may be repurposed. There is, however, a need for new inhibitors. MCC950 (formerly CRID3 or CP-456,773) is being developed as a potent and selective inhibitor of NLRP3 (****Coll et al., 2015****). Here we report a new class of molecules based on the oxazaborine ring that will further accelerate the development of NLRP3 inhibitors for use in disease and in generating new fundamental insights.**

## STAR★Methods

### Key Resources Table

**Table:** 

REAGENT or RESOURCE	SOURCE	IDENTIFIER
**Antibodies**		

Mouse IL-1â antibody	R&D Systems	Cat # AF-401-NA; RRID: AB_416684
Human IL-1â antibody	R&D Systems	Cat # AF-201-NA; RRID: AB_354387

**Chemicals**, **Peptides**, **and Recombinant Proteins**		

Dulbecco’s Modified Eagle’s Medium (DMEM)	Sigma	D6429
RPMI-1640	Sigma	R0883
Fetal bovine serum (FBS)	Thermo Fisher	10500064
2-APB	Sigma	D9754
diphenylborinic anhydride	Sigma	358835
diphenylhydramine	Sigma	D3630
2,2-diphenyltetrahydrofuran	Sigma	S408271
phenytoin	Sigma	PHR1139
LPS (E.coli O26:B6)	Sigma	L2654
LPS (E. coli 127:B8)	Sigma	L4516
ATP	Sigma	A2383
Nigericin	Sigma	N7143
Sphingosine	Sigma	S7049
MCC950	Sigma	PZ0280
Corn Oil	Sigma	C8267
3,4-methylenedioxy-â-nitrostyrene	Sigma	M7445
The BC compound library	Sigma	
Imiquimod	Sigma	1338313
Non-B analogs of 2-APB	NIH’s National Cancer Institute (NCI) Developmental Therapeutics Program	https://dtp.cancer.gov/organization/dscb/obtaining/default.htm
NBC compounds	This paper	
MSU crystals	Invivogen	Tlrl-msu
CPPD crystals	Invivogen	Tlrl-cppd
Alum crystals	Invivogen	Tlrl-alk
Flagellin from S. typhimurium	Invivogen	tlrl-stfla
Z-YVAD-AFC	Calbiochem	688225
Ac-YVAD-Cho	Merck-Millipore	400010
Recombinant caspase-1	Merck-Millipore	CC126
Silica	U.S Silica	MIN-U-SIL 15
Poly(deoxyadenylic-thymidylic) acid sodium salt (Poly dA:dT)	Sigma	P0883
Pam3CSK4	Invivogen	tlrl-pms
Lipofectamine 3000	Thermo Fisher	L3000008

**Critical Commercial Assays**		

IL-1â ELISA (mouse)	R&D Systems	DY401
IL-1á ELISA (mouse)	R&D Systems	DY400
IL-1â ELISA (human)	R&D Systems	DY201
CytoTox 96® Non-Radioactive Cytotoxicity Assay	Promega	G1780

**Deposited Data**		

Crystal structure NBC6	This paper	Cambridge Crystallographic Data Centre CCDC 1563191
Crystal structure NBC11	This paper	Cambridge Crystallographic Data Centre CCDC 1563192

**Experimental Models**: **Cell Lines**		

THP-1	ATCC	TIB-202
Primary mouse peritoneal macrophages	Brough lab, UoM	
Primary mouse BMDMs	Brough lab UoM	
ASC-mCherry BMDMs	Brough lab UoM	
HepG2	ATCC	HB-8065
HEK293	ATCC	CRL-1573

**Experimental Models**: **Organisms**/**Strains**		

C57BL/6 mice	Envigo	
NLRP3 knockout mice	Genentech	

**Software and Algorithms**		

SYBYL-X 2.1	Tripos Inc	https://www.certara.com/software/molecular-modeling-and-simulation/sybyl-x-suite/
GraphPad Prism version 7.00 for Windows	GraphPad Software	www.graphpad.com
R 3.30	R Foundation for Statistical Computing	http://www.R-project.org/
Gaussian 09	Gaussian, Inc	http://gaussian.com/
Omega 2.5.1.4	OpenEye Scientific Software	https://www.eyesopen.com/
ROCS 3.0.0	OpenEye Scientific Software	https://www.eyesopen.com/
OSIRIS DataWarrior 4.5.2	Actelion Pharmaceuticals Ltd	http://www.openmolecules.org/datawarrior/

### Contact for Reagent and Resource Sharing

Further information and requests for resources and reagents should be directed to the Lead Contact, David Brough (David.brough@manchester.ac.uk).

### Experimental Model and Subject Details

#### Cell Culture

Primary peritoneal macrophages were prepared as described previously (Le Feuvre et al., 2002). Briefly, peritoneums of male and female C57BL/6 mice (Charles River) were lavaged with 8 ml RPMI 1640 media and cells in the exudate cultured at a density of 1 × 10^6^ cells ml^-1^ in RPMI media supplemented with 10% fetal bovine serum (FBS), 100 U ml^-1^ penicillin and 100 μg ml^-1^ streptomycin (PenStrep). Primary bone marrow-derived macrophages (BMDMs) and primary bone marrow neutrophils were prepared by flushing femurs of male and female wild-type C57BL/6 or NLRP3 KO mice. Red cells were then lysed. BMDMs were generated by culturing the resulting bone marrow cells in 70% DMEM (containing 10% FBS, PenStrep) supplemented with 30% L929 mouse fibroblast-conditioned media for 7-10 days. Before experiments, cells were seeded overnight at 1 x 10^6^ ml^−1^ in 96-well plates. Neutrophils were isolated by density centrifugation of the extracted bone marrow cells in a 64% isotonic Percoll (Sigma-Aldrich) at 1,000xg for 30 min at 4°C. The pellet was then resuspended in RPMI (containing 10% FBS, PenStrep), counted, centrifuged again (2,000xg, 5 min), resuspended at 1 x 10^6^ ml^-1^, plated in 96-well plates and experimented on immediately. Purity (>90%) and viability (>95%) were determined by Diff-Quik^®^ staining (Cools-Lartigue et al., 2013). THP-1 peripheral blood monocyte-like cells were cultured in RPMI medium supplemented with 10% FBS, PenStrep, 20 mM L-Glutamine and 55 μM 2-mercaptoethanol. On the day of experiments, cells were seeded overnight at 1 x 10^6^ ml^-1^ in 96-well plates. Immortalized murine bone marrow-derived macrophages (iBMDMs) (Hornung et al., 2008) and iBMDMs stably expressing ASC conjugated to mCherry protein (Daniels et al., 2016) were cultured in DMEM, 10% FBS, PenStrep. HEK293T kidney cells and HepG2 liver cells were cultured in DMEM, 10% FBS, PenStrep.

#### *In Vivo* Peritoneal Inflammation Model

Animals were maintained under standard laboratory conditions: ambient temperatures of 21°C (± 2°C), humidity of 40–50%, 12 h light cycle, *ad libitum* access to water and standard rodent chow. All procedures were performed blinded to genotype. Treatment allocations were randomly allocated using True Random Generator™ software. All animal experiments were carried out in accordance with the United Kingdom Animals (Scientific Procedures) Act 1986 and approved by the Home Office and the local Animal Ethical Review Group, University of Manchester. Male WT C57BL/6 and strain matched NLRP3 KO mice (30 - 35g) were co-administered intraperitoneally (i.p.) with NBC13 (50 mg kg^-1^), MCC950 (50 mg kg^-1^) or vehicle (corn oil)), and 10 mg kg^-1^ LPS (from *Escherichia coli* 0127:B8) or saline control (n=6 per group). Three hours following injection the mice were anesthetized with 3-5% isoflurane, their peritoneums were lavaged with 3 ml of RPMI media and plasma taken by cardiac puncture. Levels of IL-1β in the plasma and lavage and IL-1α in the plasma were analysed by ELISA (DuoSet, R&D systems^®^).

### Method Details

#### ASC Speck Imaging

Live imaging of ASC speck formation was performed using iBMDMs transfected to stably express ASC conjugated to mCherry protein (Daniels et al., 2016). Stably transduced cells were plated overnight at 5x10^5^ cells ml^-1^. The following day, cells were primed with LPS (1 μg ml^-1^, 2 h). 1 h into priming, Hoechst 33342 (2 μg ml^-1^, Immunochemistry) was added to aid identification of the cells. Following priming, media was changed to DMEM containing 25 mM HEPES pH 7.4 and cells transferred to a BD Pathway Bioimager 855 (BD Biosciences) and imaged at 37°C as described previously (Daniels et al., 2016). Cells were pre-treated with 2APB, BC7, BC23, NBC6, or vehicle for 15 min before imaging.

#### Caspase-1 Assays

The caspase-1 activity of THP-1 cells was determined with the fluorogenic substrate Z-YVAD-AFC (caspase-1 substrate VI, Calbiochem) as previously described (Lopez-Castejon et al., 2013). Briefly, cells were lysed in hypotonic cell lysis buffer (25 mM HEPES, 5 mM EGTA, 5 mM dithiothreitol (DTT), pH 7.5) on ice for 5–10 min and centrifuged to remove the insoluble fraction (12,500×g, 10 min). THP-1 lysates (50 μl) or recombinant caspase-1 (10 U ml^-1^) was incubated with 50 μM YVAD-AFC and 50 μl of reaction buffer (0.2% CHAPS, 0.2 M HEPES, 20% sucrose, 29 mM DTT, pH 7.5) for 2 h. After incubation, the fluorescence of the AFC released from the Z-YVAD-AFC substrate was measured by an increase in fluorescence (excitation 335 nm, emission 460 nm).

#### Inflammasome Activation Assays

Peritoneal macrophages were primed with LPS (1 μg ml^-1^, 2 h) before incubation with inhibitors in serum free media (15 min) followed by stimulation with NLRP3 activators ATP (5 mM, 20 min), mono-sodium urate crystals (MSU, 250 μg ml^-1^, 1 h), calcium pyrophosphate dihydrate crystals (CPPD, 250 μg ml^-1^, 1 h), Aluminium hydroxide crystals (Alum, 250 μg ml^-1^, 1 h), nigericin (20 μM, 15 min), or sphingosine (20 μM, 1 h). THP-1 cells were primed with LPS (1 μg ml^-1^, 4 h) before incubation with inhibitors or vehicle (15 min) in serum-free media followed by stimulation with nigericin (10 μM, 1 h), or ATP (5 mM, 1 h). For AIM2/NLRC4 inflammasome activation primary BMDMs were primed with LPS (1 μg ml^-1^, 4 h). Subsequent to LPS priming, cells were pre-treated with drugs or vehicle (DMSO) in serum-free media for 15 min then stimulated with ATP (5 mM, 1 h), poly(deoxyadenylic-thymidylic) (polydA:dT) acid sodium salt transfected with Lipofectamine® 3000 (667 ng ml^-1^, 4 h) or flagellin from *S. typhimurium* (667 ng ml^-1^, 4 h). For K^+^ efflux-independent NLRP3 activation BMDMs were primed with LPS as above. Subsequent to LPS priming, cells were pre-treated with drugs or vehicle (DMSO, 15 min) in PBS then stimulated with imiquimod (10 μM, 4 h). For non-canonical inflammasome activation cells were primed with Pam3CSK4 (100 ng ml^-1^, 4 h). Subsequent to priming, cells were pre-treated with drugs or vehicle (DMSO, 15 min) in serum-free media then stimulated with LPS transfected with Lipofectamine® 3000 (2 μg ml^-1^, 24 h). Neutrophils were primed with LPS (1 μg ml^-1^, 2 h) in RPMI (containing 10% FBS, PenStrep), drug or vehicle (DMSO) was added 15 min prior to the stimulation with nigericin or vehicle (DMSO) for 1 h. Supernatants were removed and analysed for IL-1β or IL-1α content by ELISA (DuoSet, R&D systems) according to manufacturer’s instructions.

#### Washout Experiments

iBMDMs were seeded overnight at 7.5 x 10^5^ ml^-1^ in 24-well plates and primed with LPS (1 μg ml^-1^, 2 h). Subsequent to LPS priming, cells were pre-treated with drugs or vehicle (DMSO, 15 min) in serum-free media and washed 3 times, before inflammasome activation was initiated by adding ATP (5 mM, 1 h).

#### Cell Death Experiments

HEK293T kidney cells and HepG2 liver cells were treated with drug or vehicle (DMSO) for 4, 8 and 24 h in DMEM, 1% FBS, PenStrep.

Following treatment, cell death was measured by assessing lactate dehydrogenase release using the CytoTox 96 Non-Radioactive Cytotoxicity Assay (Promega) according to manufacturer’s instructions.

#### Chemistry Synthesis

Synthesis, purification and characterisation of NBC molecules are outlined in Methods S1 (Ibrahim et al., 1985, Coenen et al., 1965, Hosoya et al., 2006, Vasil'ev et al., 1994, Sridharan et al., 2010, Clemens and Hyatt, 1985). All chemicals, solvents and deuterated solvents were purchased from Sigma-Aldrich, Alfa-Aesar or Fisher Scientific. ^1^H, ^13^C and ^11^B{^1^H} NMR spectra were recorded on a Bruker Avance 400 or 300 MHz spectrometer. Chemical shifts (δ) are defined in parts per million (ppm). ^1^H NMR spectra were referenced to tetramethylsilane (TMS, δ=0.0 ppm) or residual undeuterated solvent (CDCl_3_, δ=7.26 ppm; DMSO-*d*_*6*_, δ=2.50 ppm). ^13^C NMR spectra were referenced to residual undeuterated solvent as an internal reference. ^11^B{^1^H}NMR chemical shifts were referenced to external reference BF_3_.OEt_2_ (δ=0.0 ppm). ESI and APCI mass spectrometry was carried out on a Waters Acquity UPLC system connected to a Waters SQD2 mass spectrometer. Accurate mass determination was carried out on a Thermo Exactive™ Plus EMR Orbitrap™ LC-MS system. Molecular ion peaks are defined as mass/charge (*m*/*z*) ratios. Infrared spectroscopy was recorded on a JASCO FT/IR-4100 spectrophotometer using the Spectra Manager II (JASCO) software package. Microwave irradiation was carried out on a Biotage^®^ Initiator Classic microwave using 2-5 ml Biotage^®^ glass vials. Analytical thin-layer chromatography (TLC) was performed using silica gel 60 on aluminium sheets coated with F_254_ indicator. All spots were visualised with KMnO_4_ or ultraviolet light using a MV Mineralight lamp (254/365) UVGL-58. Flash column chromatography was performed using silica gel with particle size 40-63 μm. Evaporation of solvents was conducted on a Buchi Rotavapor R-200.

#### X-ray Crystallography

X-ray diffraction data were collected at 100 K on the specimen crystals of NBC6 and NBC11 at the National Crystallography Service, Southampton, UK, with MoKα radiation produced by a rotating anode generator. The structures were solved by direct methods with SHELXS and refined with SHELXL, implemented in the WinGX package (Farrugia, 2012), by the full-matrix least-squares technique with anisotropic displacement parameters for the non-hydrogen atoms. Hydrogen atoms attached to carbon were placed in calculated positions and assumed to ride on their attached atom, methyl groups being allowed to rotate. The C12 methyl group of NBC11 showed signs of disorder in a difference electron density map and therefore was assigned two sets of sites rotated by 60° from one another with occupancy factors that refined to 0.59(2) : 0.41(2). Positions and isotropic displacement parameters for hydrogen atoms attached to nitrogen atoms were refined freely. Final discrepancy indices R(obs) and wR2 (all data) were 0.0408, 0.1120 for NBC6 and 0.0283, 0.0801 for NBC11. The highest peaks and deepest holes in a difference electron density map were 0.54, -0.36 and 0.39, -0.25 e Å^-3^ respectively. Cambridge Crystallographic Data Centre CCDC 1563191 (NBC6) and CCDC 1563192 (NBC11) contain the supplementary crystallographic data.

#### Chemistry Computational/Modelling

Initial 3D molecular structures of boron-containing compounds were constructed and then energy minimised using the Tripos force field in SYBYL-X. These geometries were subsequently optimised quantum mechanically with the semi-local M06-L density functional (Zhao and Truhlar, 2006) and the 6-31G* basis set, using the Gaussian 09 electronic structure package (Frisch et al., 2009). These geometries were used as input for structure-activity analysis and for virtual screening. For the latter, the ZINC subset *leadsNow* was employed, containing 1,943,551 molecules (4/20/12 update). Prior to shape-based screening, multiple conformations of each compound were generated *via* Omega (Hawkins et al., 2010). Shape-based screening was performed using ROCS (Grant et al., 1996) with Tanimoto scoring *via* the ShapeTanimoto and ColorTanimoto functions as implemented in OpenEye (Hawkins et al., 2007). Topomer CoMFA (Cramer, 2003) was performed using the Sybyl software package, based on NBC1-6, 8, 11-20, 22-26 and 28-29, with R-groups defined at positions 4, 5 and 6 of the oxazaborine ring.

cLogP and cLogS calculations were performed for BC7, BC23, NBC1-31 and NBC-EPPS using OSIRIS DataWarrior (version 4.5.2)(Sander et al., 2015).

#### Fluorescence Imaging

One day before experiments, 10^5^ iBMDMs were seeded on glass coverslips in 24-well plates. For Ca^2+^ imaging experiments cells were transferred to the following solution containing (in mM): NaCl, 130; KCl, 5; HEPES, 10; D-glucose, 10; CaCl_2_, 2; MgCl_2_, 1 (pH 7.4) and were loaded with 3 μM fura-2-acetoxymethylester (Fura-2-AM, Molecular Probes, Eugene, USA) for 30 min at RT (20-23°C). After washing, coverslips were mounted in a chamber on an inverted Olympus IX50 microscope equipped with a water immersion objective 40x UApo/340 (Olympus Optical Co. GmbH, Hamburg, Germany). The fluorescence imaging system consisted of a Polychrome V monochromator, a Hamamatsu Orca 03G camera and the Windows 7 based Live Acquisition software (Till Photonics, München, Germany). Cells were exposed to light of 340±5 and 380±5 nm wavelength every 10 or 20 s in experiments using 100 μM or 5 mM ATP, respectively. Emission light was passed through a 400 nm dichroic mirror and a 420 nm long pass emission filter (both Olympus, Germany) prior to acquisition. Cells were primed with LPS (1 μg ml^-1^, 4 h) and incubated with drug (2APB (75 μM), BC7, BC23, NBC6 (all 30 μM)) or vehicle, pre- (2 min) or post- (3 min) ATP stimulation. Data are presented as the ratio of the two background corrected fluorescence intensities. To enable fast drug application, cells were superfused using a four-barrel microperfusion pipette positioned in close proximity to the viewing field.

#### Electrophysiological Recordings

One day before experiments, 10^5^ iBMDMs were seeded on glass coverslips in 24-well plates. Membrane currents were measured using the whole-cell configuration of the patch-clamp technique. An EPC 10 patch-clamp amplifier (HEKA, Lambrecht/Pfalz, Germany) was interfaced to a computer for pulse application and data recording using the program PatchMaster (HEKA). Patch electrodes of 3-5 MΩ were fabricated on a two-stage puller (Narishige PC 10, Tokyo, Japan) from borosilicate glass (Hilgenberg, Malsfeld, Germany). For volume-regulated Cl^-^ current (VRAC) recordings, patch electrodes were filled with the following intracellular solution I1 (in mM): N-Methyl-D-Glucamine-Chloride (NMG-Cl), 120; HEPES, 10; EGTA, 11; CaCl_2_, 1; MgCl_2_, 2; Na_2_ATP, 3 (pH 7.3). Cells were kept in extracellular solution E1 containing (in mM): NMG-Cl, 50; HEPES, 10; D-glucose, 10; CaCl_2_, 2; MgCl_2_, 1; D-mannitol, 170 (300 mosmol kg^-1^, pH 7.3). To activate VRAC currents, cells were superfused with hypo-osmolar extracellular solution E2 containing (in mM): NMG-Cl, 50; HEPES, 10; D-glucose, 10; CaCl_2_, 2; MgCl_2_, 1 (130 mosmol kg^-1^, pH 7.3). All recordings were done at RT (20-23°C). For solution exchange, a four-barrel microperfusion pipette was used. Cells were primed with LPS (1 μg ml^-1^, 4 h) and incubated with drug (2APB (75 μM), BC7, BC23, NBC6 (all 30 μM)) or vehicle, 15 min before stimulation. Whole-cell currents were filtered at 3 kHz and stored for subsequent analyses, which were performed using the program s (HEKA, Lambrecht/Pfalz, Germany).

### Quantification and Statistical Analysis

Data are presented as mean values + standard error of the mean (s.e.m). Levels of significance were p<0.05 (*), p<0.01 (**), p<0.001 (***). Statistical analyses were carried out using GraphPad Prism (version 7) or R (version 3.3.0). Percentage control data were analysed with Holm-Sidak corrected one-sample t-tests against the value of 100%. Data with multiple groups were analysed with a one-way ANOVA. Experiments with two independent variables were analysed using two-ANOVA. These analyses were followed by Holm-Sidak corrected post-hoc comparisons. Homoscedasticity and normality of the residuals were evaluated with the Levene’s test and Shapiro Wilks, respectively, and appropriate transformations or corrections were applied where necessary. Dose response curves where fitted using non-linear least squares regression with a 3 parameter logistical sigmoidal model.

### Data and Software Availability

The crystallographic data for NBC6 and NBC11 is deposited with the Cambridge Crystallographic Data Centre (CCDC) with the deposition numbers CCDC 1563191 (NBC6) and CCDC 1563192 (NBC11).

## Author Contributions

Conceptualization, D.B. and S.F.; Methodology, D.B., S.F., M.K.H., S.M.A., C.B.L., and R.A.B.; Investigation, A.G.B., J.R.-A., M.J.D.D., C.S.W., C.H.S., T.S., H.H., P.J., N.G.S., H.E., N.M.L., and M.K.; Formal analysis, J.R.-A.; Writing – Original Draft, D.B., S.F., and A.G.B.; Writing – Review & Editing, D.B., S.F., and A.G.B.; Funding Acquisition, D.B. and S.F.; Resources, D.B., S.F., and S.M.A.; Supervision, D.B., N.J.R., R.A.B., C.E., and S.F.

## Supplemental Information

Document S1. Figure S1, Table S1, Schemes S1–S7, and Methods S1

Document S2. Article plus Supplemental Information
