# Supplementary material for: Boron-Based Inhibitors of the NLRP3 Inflammasome
Source: Cell Chem Biol. 2017 Nov 16;24(11):1321–1335.e5. doi: 10.1016/j.chembiol.2017.08.011 (PMC5696570; doi:10.1016/j.chembiol.2017.08.011)
Supplement: Document S1. Figure S1, Table S1, Schemes S1–S7, and Methods S1 [file mmc1.pdf]

Cell Chemical Biology, Volume *24*

Supplemental Information

Boron-Based Inhibitors of the NLRP3 Inflammasome

Alex G. Baldwin, Jack Rivers-Auty, Michael J.D. Daniels, Claire S. White, Carl H. Schwalbe, Tom Schilling, Halah Hammadi, Panichakorn Jaiyong, Nicholas G. Spencer, Hazel England, Nadia M. Luheshi, Manikandan Kadirvel, Catherine B. Lawrence, Nancy J. Rothwell, Michael K. Harte, Richard A. Bryce, Stuart M. Allan, Claudia Eder, Sally Freeman, and David Brough

Supplementary items

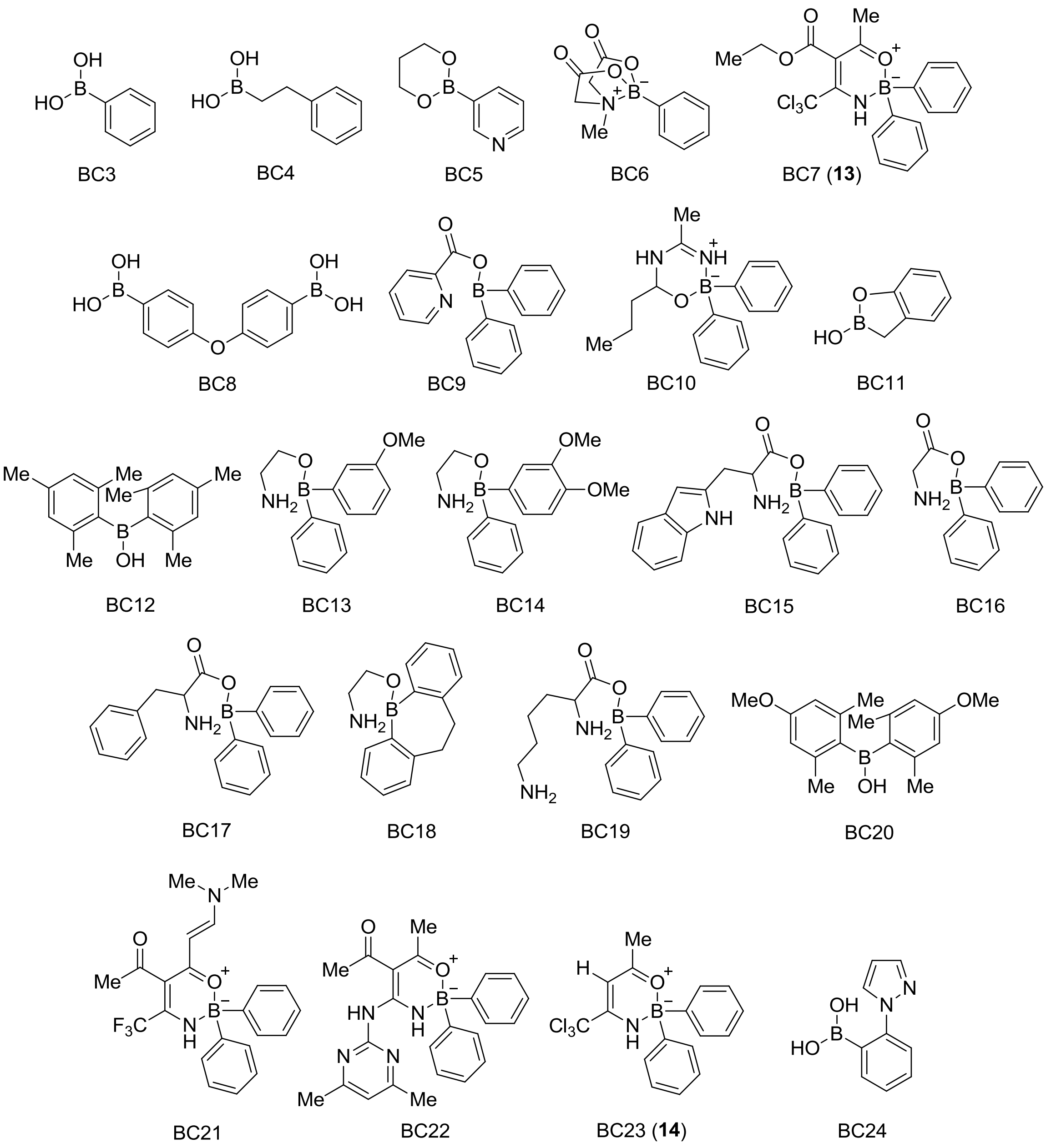

Figure S1 related to Figure 2: Structures of BC molecules. Structures of screened B-containing molecules with similar features and properties to 2-APB.

Parameter	NBC6 (**15**) (X-ray)	NBC6 (**15**) (calc)	NBC11 (**16**) (X-ray)	NBC11 (**16**) (calc)	NBC19 (**17**) (calc)	NBC27 (**18**) (calc)	NBC30 (**19**) (calc)
$q(X_1)^a$	-	-0.571	-	-0.542	-0.567	-0.506	-0.513
$q(B_2)$	-	0.605	-	0.587	0.574	0.515	0.508
$q(N_3)$	-	-0.711	-	-0.719	-0.723	-0.735	-0.747
$q(C_4)$	-	0.391	-	0.448	0.410	0.465	0.458
$q(C_5)$	-	-0.173	-	-0.132	-0.184	-0.346	-0.340
$q(C_6)$	-	0.663	-	0.480	0.664	0.440	0.435
$d(B_2\text{-}X_1)^a$	1.54	1.56	1.53	1.54	1.54	1.60	1.62
$d(B_2\text{-}N_1)$	1.55	1.57	1.57	1.58	1.57	1.58	1.58
$d(X_1\text{-}C_6)^a$	1.30	1.29	1.30	1.30	1.29	1.32	1.32
$d(C_6\text{-}C_5)$	1.44	1.45	1.39	1.40	1.46	1.41	1.41
$d(C_5\text{-}C_4)$	1.41	1.41	1.42	1.42	1.41	1.38	1.38
$d(C_4\text{-}N_3)$	1.31	1.32	1.30	1.31	1.32	1.32	1.32
$\theta(X_1\text{-}B_2\text{-}N_3)^a$	102.6	101.3	104.0	102.5	102.3	112.5	111.2
ν_1	-7.0	-9.2	-8.3	-14.7	-13.2	-0.9	-3.5
ν_2	-23.5	-25.4	-17.3	-17.8	-25.4	-1.7	-9.8
ν_3	16.2	20.3	12.9	14.3	24.6	-0.5	5.3
ν_4	21.4	19.7	17.6	22.2	16.2	5.3	11.9
ν_5	-45.4	-47.2	-37.3	-47.4	-48.8	-6.6	-20.8
ν_6	38.7	42.9	33.2	44.6	48.2	4.5	16.8
$\langle\nu\rangle$	25.4	27.5	21.1	26.8	29.4	3.3	11.4

[a]For NBC6, NBC11 and NBC19, $X_1 = O_1$; for NBC27 and NBC30, $X_1 = N_1$

Table S1 related to Figure 4: Electronic and structural properties of X-ray and calculated geometries of selected oxazaborines and diazaborines at the M06-L/6-31G* level of theory. Mulliken charges *q* (in *e*), bond distances *d* (in Å), ring pucker torsion angles $\nu_1 - \nu_6$ (defined by atoms B_2-N_3-C_4-C_5, N_3-C_4-C_5-C_6, C_4-C_5-C_6-X_1,C_5-C_6-X_1-B_2,C_6-X_1-B_2-N_3, X_1-B_2-N_3-C_4 respectively)[a] and their mean unsigned value $\langle\nu\rangle$ (in deg), bond angle θ (in deg) and donor-acceptor tetrahedral character at boron, THC_{DA} (%). Atom numbering follows Fig. 4A.

Methods S1, related to STAR Methods

1. Design, synthesis and characterisation of the NBC compounds

1.1 General procedure for enaminone reaction using $Zn(acac)_2$

An appropriate nitrile (1 eq) was added to a suspension containing 1,3-dicarbonyl (1 eq), $Zn(acac)_2$ (1-2 %mol) and Na_2CO_3 (1.1 eq) in anhydrous DCM (5 ml). The mixture was stirred at room temperature under N_2 for 3h, unless otherwise stated. DCM/EtOAc (15 ml) was added and the mixture washed with H_2O, dried over $MgSO_4$ and concentrated *in vacuo*.

For deacetylated compounds, $K_2CO_{3(sat)}$ (10 ml) and EtOH (5 ml) were then added and the mixture was stirred at room temperature for 24h. The mixture was extracted with EtOAc, dried over $MgSO_4$ and concentrated *in vacuo*. Alternatively, acetylated enaminones were passed through SiO_2 by flash column chromatography to give the corresponding deacetylated products.

1.2 General procedure for enaminone reaction using $SnCl_4$

An appropriate nitrile (1 eq) was added to a suspension containing 1,3-dicarbonyl (1 eq) and Na_2CO_3 (1.1 eq) in anhydrous toluene (10 ml). For deacetylated products, addition of Na_2CO_3 was omitted. $SnCl_4$ (1 eq) was slowly added and the mixture was stirred at room temperature under N_2 for 30 min. The mixture was then heated to 80°C and stirred for 4h, unless otherwise stated. MeCN (20 ml) was added to dissolve the gummy solid, filtered to remove Na_2CO_3, concentrated *in vacuo* and

dried under vacuum overnight. After 16h, boiling $CHCl_3$ (20 ml) was added and refluxed for 1h. The solid was filtered and dried under vacuum.

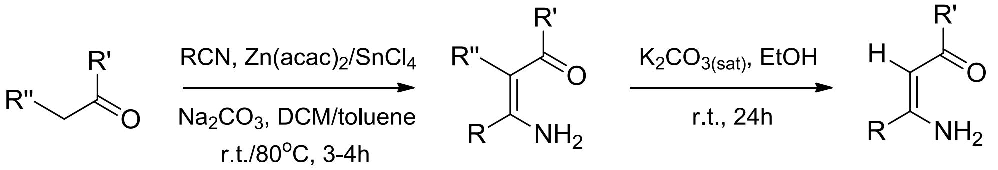

1a-g **2a-b**

R	R'	R''	
CCl_3	Me	COOEt	**1a**
Me	Me	COOEt	**1b**
Me	Me	COMe	**1c**
CCl_3	NH_2	COMe	**1d**
CCl_3	Me	$CONMe_2$	**1e**
CCl_3	NH_2	CN	**1f**
CCl_3	O^tBu	COMe	**1g**

R	R'	
CCl_3	Me	**2a**
CCl_3	NH_2	**2b**

Scheme 1 | Synthesis of enaminones 1a-g and 2a-b.

Ethyl (*E*)-2-acetyl-3-amino-4,4,4-trichloro-2-butenoate (1a). The product was purified using flash column chromatography. Ethyl acetate/*n*-hexane, 1:4. Yield: 14%, brown oil. ^1H NMR (400 MHz, $CDCl_3$): δ 4.19 (q, J = 7.2 Hz, 2H, $CH_3C\underline{H}_2O$), 2.23 (3H, s, 3H, CH_3CO), 1.29 (t, J = 7.0 Hz, 3H, $C\underline{H}_3CH_2O$), NH_2 signal was not observed; MS(ES^+) (*m/z*): 274.0 [M+H, ^{35}Cl, ^{35}Cl, ^{35}Cl, 38%]$^+$, 276.0 [M+H, ^{35}Cl, ^{35}Cl, ^{37}Cl, 48%]$^+$, 278.0 [M+H, ^{35}Cl, ^{37}Cl, ^{37}Cl, 23%]$^+$, 280.0 [M+H, ^{37}Cl, ^{37}Cl, ^{37}Cl, 6%]$^+$, 338.0 [M-H+Zn^{2+}, ^{35}Cl, ^{35}Cl, ^{35}Cl, 100%]$^+$, 340.0 [M-H+Zn^{2+}, ^{35}Cl, ^{35}Cl, ^{37}Cl, 50%]$^+$, 342.0 [M-H+Zn^{2+}, ^{35}Cl, ^{37}Cl, ^{37}Cl, 25%]$^+$.

Ethyl (*E*)-2-acetyl-3-amino-2-butenoate (1b). $SnCl_4$ could not be removed from enaminone, yield calculated based on a stoichiometric quantity of $SnCl_4$. Yield: 67%, orange solid. mp: 136-138 ^oC; ^1H NMR (400 MHz, DMSO-d_6): δ 10.77 (br s, 1H, $CH_3(N\underline{H}_2)C{=}CH$), 8.37 (br s, 1H, $CH_3(N\underline{H}_2)C{=}CH$), 4.11 (q, J = 7.1 Hz, 2H,

$CH_3C\underline{H}_2O$), 2.13 (s, 3H, CH_3), 2.11 (s, 3H, CH_3), 1.23 (t, *J* = 7.2 Hz, 3H, $C\underline{H}_3CH_2O$); ^{13}C NMR (100 MHz, DMSO-d_6): δ 195.3 ($CH_3\underline{C}O$), 169.5 ($CH_3(NH_2)\underline{C}$=C), 168.1 (EtO_2C), 102.1 ($CH_3(NH_2)C$=$\underline{C}$), 59.7 ($CH_3\underline{C}H_2O$), 30.4 ($\underline{C}H_3CO$), 22.4 ($\underline{C}H_3(NH_2)C$=C), 14.5 ($\underline{C}H_3CH_2O$); IR: 3263 (N-H), 3145 (N-H), 1651 (C=O), 1600 (C=C, conjugated) cm^{-1}; MS(ES^-) (*m/z*): 168.8 $[M-H, 100\%]^-$; MS(ES^+) (*m/z*): 130.9 $[M-Ac, 53\%]^+$, 171.0 $[M+H, 18\%]^+$, 194.1 $[M+Na, 25\%]^+$.

3-(1-Aminoethylidene)pentane-2,4-dione (1c). Yield: 61%, cream solid. Exists as a mixture of tautomers in a 9:1 ratio. mp: 129-132 ^oC; ^1H NMR (400 MHz, d_6-DMSO): δ 10.65 (br s, 1H, $CH_3(N\underline{H}_2)C$=C), 8.30 (br s, 1H, $CH_3(N\underline{H}_2)C$=C), 5.67 (s, 1H, C=$C\underline{H}_2$ of enol form), 4.88 (d, 1H, *J* = 1.4 Hz, C=$C\underline{H}_2$ of enol form), 2.17 (s, 6H, CH_3CO x 2), 2.04 (s, 3H, $C\underline{H}_3(NH_2)C$=C); 1.99 (s, 3H, $C\underline{H}_3C(OH)C$ of enol form), 1.91 (s, 3H, $C\underline{H}_3(OH)C$ of enol form); ^{13}C NMR (100 MHz, DMSO-d_6): δ 197.9 ($CH_3\underline{C}O$), 193.0 ($CH_3\underline{C}O$), 165.2 ($CH_3(NH_2)\underline{C}$=C), 113.1 ($CH_3(NH_2)C$=$\underline{C}$), 100.6 (H_2C=C(OH)$\underline{C}$ of enol form), 94.1 ($H_2\underline{C}$=C(OH)C of enol form), 31.2 ($\underline{C}H_3CO$), 27.5 ($\underline{C}H_3CO$), 21.3 ($\underline{C}H_3(NH_2)C$=C); MS(ES^+) (*m/z*): 142.1 $[M+H, 100\%]^+$, 164.1 $[M+Na, 29\%]^+$.

(*Z*)-2-Acetyl-3-amino-4,4,4-trichlorobut-2-enamide (1d). Yield: 54%, white solid. mp: 108-110 ^oC; ^1H NMR (300 MHz, $CDCl_3$): δ 8.75 (br s, 2H, NH_2), 6.02 (br s, 1H, $CONH_2$), 5.95 (br s, 1H, $CONH_2$), 2.38 (s, 3H, CH_3CO); ^{13}C NMR (75 MHz, $CDCl_3$): δ 197.2 ($CH_3\underline{C}O$), 169.9 ($CONH_2$), 156.7 ($Cl_3C(NH_2)\underline{C}$=C), 104.0 ($Cl_3C(NH_2)C$=$\underline{C}$), 93.3 (CCl_3), 28.5 ($\underline{C}H_3CO$); IR: 3417 (N-H), 3310 (N-H), 3209 (N-H), 1651 (C=O), 1592 (C=C, conjugated) cm^{-1}.

(*Z*)-2-Acetyl-3-amino-4,4,4-trichloro-*N*,*N*-dimethylbut-2-enamide (1e). Yield: 88%, cream solid. mp: 102-104 ^oC; ^1H NMR (300 MHz, $CDCl_3$): δ 8.56 (br s, 1H,

$Cl_3C(NH_2)C$=C), 2.97 (d, J = 3.3 Hz, 6H, $CON(CH_3)_2$), 2.14 (s, 3H, CH_3CO); ^{13}C NMR (100 MHz, $CDCl_3$): δ 196.7 ($CH_3\underline{C}O$), 168.6 ($Cl_3C(NH_2)\underline{C}$=C), 155.6 ($\underline{C}ON(CH_3)_2$), 103.2 ($Cl_3C(NH_2)C$=$\underline{C}$), 93.3 (CCl_3), 38.8 ($CON(\underline{C}H_3)_2$), 35.1 ($CON(\underline{C}H_3)_2$), 27.9 ($\underline{C}H_3CO$); IR: 3282 (N-H), 1602 (C=C, conjugated), 1497 (C=C-NH_2) cm^{-1}; MS(ES^-) (*m/z*): 270.9 [M-H, ^{35}Cl, ^{35}Cl, ^{35}Cl, 18%]$^-$, 273.0 [M-H, ^{35}Cl, ^{35}Cl, ^{37}Cl, 22%]$^-$, 275.0 [M-H, ^{35}Cl, ^{37}Cl, ^{37}Cl, 5%]$^-$, 277.0 [M-H, ^{37}Cl, ^{37}Cl, ^{37}Cl, 3%]$^-$; MS(ES^+) (*m/z*): 273.0 [M+H, ^{35}Cl, ^{35}Cl, ^{35}Cl, 100%]$^+$, 275.0 [M+H, ^{35}Cl, ^{35}Cl, ^{37}Cl, 54%]$^+$, 277.0 [M+H, ^{35}Cl, ^{37}Cl, ^{37}Cl, 83%]$^+$, 279.0 [M+H, ^{37}Cl, ^{37}Cl, ^{37}Cl, 12%]$^+$, 295.0 [M+Na, ^{35}Cl, ^{35}Cl, ^{35}Cl, 49%]$^+$, 297.0 [M+Na, ^{35}Cl, ^{35}Cl, ^{37}Cl, 52%]$^+$, 299.0 [M+Na, ^{35}Cl, ^{37}Cl, ^{37}Cl, 12%]$^+$, 301.0 [M+Na, ^{37}Cl, ^{37}Cl, ^{37}Cl, 2%]$^+$, 311.0 [M+K, ^{35}Cl, ^{35}Cl, ^{35}Cl, 23%]$^+$, 313.0 [M+K, ^{35}Cl, ^{35}Cl, ^{37}Cl, 20%]$^+$, 315.0 [M+K, ^{35}Cl, ^{37}Cl, ^{37}Cl, 8%]$^+$, 317.0 [M+K, ^{37}Cl, ^{37}Cl, ^{37}Cl, 1%]$^+$; HRMS(ES^+) (*m/z*): $[M+Na]^+$ calcd. for $C_8H_{11}{}^{35}Cl_3N_2O_2Na$, 294.9778; found, 294.9789, error: 3.7 ppm.

(*Z*)-2-Cyano-3-amino-4,4,4-trichlorobut-2-enamide (1f). Cyanoacetamide (1.68 g, 20 mmol) and trichloroacetonitrile (2.00 mL, 20 mmol) were added to a suspension of sodium acetate (1.64 g, 20 mmol) and anhydrous ethanol (100 ml). The reaction mixture was stirred at room temperature under N_2 for 16 h. The reaction mixture was dissolved in water, the precipitate was filtered and recrystallized from EtOH (Ibrahim et al., 1985). Yield: 0.45g (10%), cream solid. mp: 124-126 ^oC (lit.(Ibrahim et al., 1985) 152 ^oC); ^{1}H NMR (300 MHz, d_4-MeOH): δ 11.08 (br s, 1H, NH_2), 8.79 (br s, 1H, NH_2), 7.43 (br s, 1H, CONH), 7.20 (br s, 1H, CONH); IR: 3323 (N-H), 3198 (N-H), 2197 (C≡N), 1660 (C=O), 1593 (C=C, conjugated) cm^{-1}; MS(ES^-) (*m/z*): 225.9 [M-H, ^{35}Cl, ^{35}Cl, ^{35}Cl, 71%]$^-$, 227.9 [M-H, ^{35}Cl, ^{35}Cl, ^{37}Cl, 100%]$^-$, 229.9 [M-H, ^{35}Cl, ^{37}Cl, ^{37}Cl, 22%]$^-$, 231.9 [M-H, ^{37}Cl, ^{37}Cl, ^{37}Cl, 3%]$^-$. HRMS($APCI^+$) (*m/z*): $[M+H]^+$ calcd. for $C_5H_5{}^{35}Cl_3N_3O$, 227.9493; found, 227.9493, error: 0.0 ppm.

***tert*-Butyl (*Z*)-2-acetyl-3-amino-4,4,4-trichlorobut-2-enoate (1g)**. Yield: 88%, cream solid. mp: 75-76 ^{o}C; ^{1}H NMR (300 MHz, $CDCl_3$): δ 2.31 (s, 3H, CH_3CO), 1.55 (s, 9H, $(CH_3)_3C$); ^{13}C NMR (75 MHz, $CDCl_3$): δ 196.7 ($CH_3\underline{C}O$), 167.3 (C=$\underline{C}$(NH_2)CCl_3), 157.5 (CONH), 104.1 ($\underline{C}$=C(NH_2)CCl_3), 93.4 (CCl_3), 82.4 ((CH_3)$_3\underline{C}$), 29.1 ($\underline{C}H_3CO$), 27.7 (($\underline{C}H_3$)$_3$C); IR: 3310 (N-H), 2979 (N-H), 1712 (C=O), 1673 (C=O), 1605 (C=C, conjugated) cm^{-1}; MS(ES^{-}) (*m/z*): 300.0 [M-H, ^{35}Cl, ^{35}Cl, ^{35}Cl, 86%]$^{-}$, 301.9 [M-H, ^{35}Cl, ^{35}Cl, ^{37}Cl, 100%]$^{-}$, 304.0 [M-H, ^{35}Cl, ^{37}Cl, ^{37}Cl, 39%]$^{-}$, 306.0 [M-H, ^{37}Cl, ^{37}Cl, ^{37}Cl, 6%]$^{-}$; MS(ES^{+}) (*m/z*): 227.8 [M-O^{t}Bu, ^{35}Cl, ^{35}Cl, ^{35}Cl, 72%]$^{+}$, 229.8 [M-O^{t}Bu, ^{35}Cl, ^{35}Cl, ^{37}Cl, 74%]$^{+}$, 231.8 [M-O^{t}Bu, ^{35}Cl, ^{37}Cl, ^{37}Cl, 25%]$^{+}$, 233.8 [M-O^{t}Bu, ^{37}Cl, ^{37}Cl, ^{37}Cl, 2%]$^{+}$, 323.9 [M+Na, ^{35}Cl, ^{35}Cl, ^{35}Cl, 10%]$^{+}$, 325.9 [M+Na, ^{35}Cl, ^{35}Cl, ^{37}Cl, 13%]$^{+}$, 327.9 [M+Na, ^{35}Cl, ^{37}Cl, ^{37}Cl, 3%]$^{+}$, 329.9 [M+Na, ^{37}Cl, ^{37}Cl, ^{37}Cl, 1%]$^{+}$; HRMS(ES^{+}) (*m/z*): [M+Na]$^{+}$ calcd. for $C_{10}H_{14}{}^{35}Cl_3NO_3Na$, 323.9931; found, 323.9929, error: 0.6 ppm.

(*Z*)-4-Amino-5,5,5-trichloropent-3-en-2-one (2a). Yield: 52%, pale orange solid. mp: 66-68 ^{o}C (lit.(Coenen et al., 1965) 67-68 ^{o}C); ^{1}H NMR (400 MHz, $CDCl_3$): δ 5.85 (t, *J* = 1.2 Hz, 1H, $Cl_3C(NH_2)C{=}C\underline{H}$), 2.16 (s, 3H, CH_3CO), NH_2 signal was not observed; IR: 3299 (N-H), 3159 (N-H), 1621 (C=O), 1600 (C=C, conjugated), 1505 (C=C-NH_2) cm^{-1}; MS(ES^{+}) (*m/z*): 201.9 [M+H, ^{35}Cl, ^{35}Cl, ^{35}Cl, 96%]$^{+}$, 203.9 [M+H, ^{35}Cl, ^{35}Cl, ^{37}Cl, 83%]$^{+}$, 205.9 [M+H, ^{35}Cl, ^{37}Cl, ^{37}Cl, 32%]$^{+}$, 207.9 [M+H, ^{37}Cl, ^{37}Cl, ^{37}Cl, 6%]$^{+}$, 402.1 [2 x M, ^{35}Cl, ^{35}Cl, ^{35}Cl, 100%]$^{+}$.

(*Z*)-3-Amino-4,4,4-trichlorobut-2-enamide (2b). Yield: 39%, cream solid. mp: 95-97 ^{o}C (dec); ^{1}H NMR (400 MHz, $CDCl_3$): δ 6.72 (br s, 1H, NH), 5.33 (s, 1H, $Cl_3C(NH_2)C{=}C\underline{H}$), 5.30 (br s, 1H, NH), 1 x NH signal not observed; ^{13}C NMR (100 MHz, $CDCl_3$): δ 170.6 (C=$\underline{C}$(NH_2)CCl_3), 156.8 (CONH), 94.3 (CCl_3), 86.1 ($\underline{C}$=C(NH_2)CCl_3); IR: 3479 (N-H), 3412 (N-H), 3277 (N-H), 3154 (N-H), 1649 (C=O),

1623 (C=O), 1589 (C=C), 1539 (C=C-NH_2) cm^{-1}; MS(ES^-) (*m/z*): 201.9 [M-H, ^{35}Cl, ^{35}Cl, ^{35}Cl, 79%]$^-$, 203.9 [M-H, ^{35}Cl, ^{35}Cl, ^{37}Cl, 46%]$^-$, 205.9 [M-H, ^{35}Cl, ^{37}Cl, ^{37}Cl, 30%]$^-$, 207.9 [M-H, ^{37}Cl, ^{37}Cl, ^{37}Cl, 11%]$^-$; MS(ES^+) (*m/z*): 203.0 [M+H, ^{35}Cl, ^{35}Cl, ^{35}Cl, 71%]$^+$, 204.9 [M+H, ^{35}Cl, ^{35}Cl, ^{37}Cl, 57%]$^+$, 206.9 [M+H, ^{35}Cl, ^{37}Cl, ^{37}Cl, 9%]$^+$, 208.9 [M+H, ^{37}Cl, ^{37}Cl, ^{37}Cl, 2%]$^+$; HRMS($APCI^+$) (*m/z*): $[M+H]^+$ calcd. for $C_4H_6{}^{35}Cl_3N_2O$, 202.9540; found, 202.9545, error: 2.5 ppm.

1.3 General procedure for oxazaborine reaction

To prepare DPBA, 2-APB (0.50 g, 2.22 mmol) was dissolved in Me_2CO (5 ml) and MeOH (5 ml) in a flask covered in aluminium foil. HCl was added dropwise until the solution reached pH 1-2. The reaction mixture was stirred at room temperature for 1h. Et_2O (15 ml) was added and extracted with Et_2O. The organic layer was dried over $MgSO_4$ and concentrated *in vacuo* to give DPBA (0.47 g, 61%) as an orange oil (Hosoya et al., 2006).

An appropriate enaminone (3 eq) was added to a solution of DPBA (1 eq) in anhydrous THF (5 ml). The mixture was stirred at 50^oC under N_2 for 16h. The mixture was concentrated *in vacuo* and purified by flash column chromatography. The collected fractions were combined, evaporated *in vacuo* and stirred in cold *n*-hexane (15 ml) for 30 min. The precipitate was then filtered and dried under vacuum to give the corresponding oxazaborine products. Borylation of enaminodiones yields oxazaborine compounds of type **A** configuration, deacetylated enaminones of type **B** configuration and (*Z*)-2-cyano-3-amino-4,4,4-trichlorobut-2-enamide of type **C** configuration (NBC18) (Fig. 3A,B) (Vasilev et al., 1992, Vasilev et al., 1994).

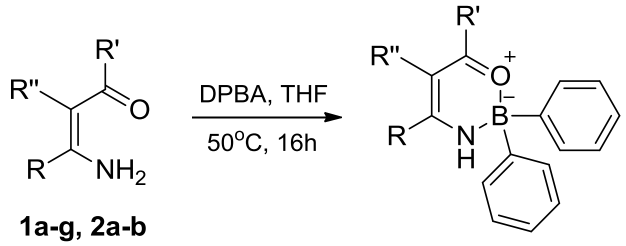

1a-g, 2a-b

R	R'	R''		R	R'	R''	
CCl_3	Me	COOEt	**BC7**	CCl_3	Me	$CONMe_2$	**NBC11**
CCl_3	Me	H	**BC23**	CCl_3	NH_2	CN	**NBC18**
CCl_3	Me	COMe	**NBC1**	Ph	Me	COMe	**NBC23**
Me	Me	COOEt	**NBC2**	CCl_3	NH_2	$CONH_2$	**NBC24**
Me	Me	H	**NBC3**	CCl_3	O^tBu	COMe	**NBC25**
Me	Me	COMe	**NBC4**	SCH_2CH_3	O^tBu	COMe	**NBC26**
CCl_3	NH_2	H	**NBC5**	^tBu	Me	H	**NBC28**
CCl_3	NH_2	COMe	**NBC6**				

Scheme 2 | Synthesis of BC7/23 and NBC1-6, NBC11, NBC18, NBC23-26 and NBC28.

5-(Ethoxycarbonyl)-6-methyl-2,2-diphenyl-4-(trichloromethyl)-2,3-dihydro-1,3,2-oxazaborinin-1-ium-2-uide (BC-7, 13). Ethyl acetate/*n*-hexane, 1:49. Yield: 8%, yellow solid. mp: 109-110 ^oC; ^1H NMR (400 MHz, $CDCl_3$): δ 8.04 (br s, 1H, $Cl_3C(NH)C{=}C$), 7.37 (dd, J = 8.0 Hz & 1.6 Hz, 4H, B-Ph(*o*)), 7.23-7.34 (m, 6H, B-Ph(*m*/*p*)), 4.18 (q, J = 7.2 Hz, 2H, $CH_3C\underline{H_2}O$), 2.50 (s, 3H, CH_3CO), 1.30 (t, J = 7.2 Hz, 3H, $C\underline{H_3}CH_2O$); ^{13}C NMR (100 MHz, $CDCl_3$): δ 188.9 ($CH_3\underline{C}O$), 165.4 ($Cl_3C(NH)\underline{C}{=}C$), 164.3 ($EtO_2C$), 131.8 (B-Ph(*o*)), 127.6 (B-Ph(*m*)), 127.0 (B-Ph(*p*)), 102.3 ($Cl_3C(NH)C{=}\underline{C}$), 93.4 ($CCl_3$), 61.7 ($CH_3\underline{C}H_2O$), 24.4 ($\underline{C}H_3CO$), 13.7 ($\underline{C}H_3CH_2O$), B-Ph(*i*) quaternary signal not observed; $^{11}B\{^1H\}$ (128 MHz, $CDCl_3$): δ 3.94; IR: 3306 (N-H), 1709 (C=O), 1592 (C=C, conjugated) cm^{-1}; MS(ES^-) (*m/z*): 435.2 [M-H, ^{10}B, ^{35}Cl, ^{35}Cl, ^{35}Cl, 10%]$^-$, 436.2 [M-H, ^{11}B, ^{35}Cl, ^{35}Cl, ^{35}Cl, 58%]$^-$, 437.2 [M-H, ^{10}B, ^{35}Cl, ^{35}Cl, ^{37}Cl, 29%]$^-$, 438.2 [M-H, ^{11}B ^{35}Cl, ^{35}Cl, ^{37}Cl, 58%]$^-$, 439.2 [M-H,

^{10}B, ^{35}Cl, ^{37}Cl, ^{37}Cl, 17%]$^{-}$, 440.2 [M-H, ^{11}B, ^{35}Cl, ^{37}Cl, ^{37}Cl, 26%]$^{-}$, 441.3 [M-H, ^{10}B, ^{37}Cl, ^{37}Cl, ^{37}Cl, 8%]$^{-}$, 442.2 [M-H, ^{11}B, ^{37}Cl, ^{37}Cl, ^{37}Cl, 3%]$^{-}$; HRMS(ES^{-}) (*m/z*): [M-H]$^{-}$ calcd. for $C_{20}H_{18}{}^{11}B^{35}Cl_3NO_3$, 436.0451; found, 436.0431, error: 4.6 ppm.

6-Methyl-2,2-diphenyl-4-(trichloromethyl)-2,3-dihydro-1,3,2-oxazaborinin-1-ium-2-uide (BC-23, 14). Ethyl acetate/hexane, 1:19. Yield: 56%, yellow solid. mp: 113-114 ^{o}C (lit.[19] 112.5-113.5 ^{o}C); ^{1}H NMR (400 MHz, $CDCl_3$): δ 7.39 (dd, *J* = 7.7 Hz & 1.4 Hz, 4H, B-Ph(*o*)), 7.21-7.35 (m, 6H, B-Ph(*m*/*p*)), 5.80 (d, *J* = 2.4 Hz, 1H, Cl_3C(NH)C=C<u>*H*</u>), 2.25 (s, 3H, CH_3CO), NH signal not observed; ^{13}C NMR (100 MHz, $CDCl_3$): δ 186.3 (CH_3<u>*C*</u>O), 165.8 (Cl_3C(NH)<u>*C*</u>=CH), 131.7 (B-Ph(*o*)), 127.5 (B-Ph(*m*)), 126.8 (B-Ph(*p*)), 92.9 (CCl_3), 91.6 (Cl_3C(NH)C=<u>*C*</u>H), 24.7 (<u>*C*</u>H_3CO), B-Ph(*i*) quaternary signal not observed; ^{11}B{^{1}H} (128 MHz, $CDCl_3$): δ 4.38; IR: 3321 (N-H), 1600 (C=C, conjugated), 1523 (C=C-NH) cm^{-1}; MS(ES^{-}) (*m/z*): 363.0 [M-H, ^{10}B, ^{35}Cl, ^{35}Cl, ^{35}Cl, 19%]$^{-}$, 364.0 [M-H, ^{11}B, ^{35}Cl, ^{35}Cl, ^{35}Cl, 100%]$^{-}$, 365.0 [M-H, ^{10}B, ^{35}Cl, ^{35}Cl, ^{37}Cl, 32%]$^{-}$, 366.0 [M-H, ^{11}B, ^{35}Cl, ^{35}Cl, ^{37}Cl, 95%]$^{-}$, 367.0 [M-H, ^{10}B, ^{35}Cl, ^{37}Cl, ^{37}Cl, 19%]$^{-}$, 368.0 [M-H, ^{11}B, ^{35}Cl, ^{37}Cl, ^{37}Cl, 28%]$^{-}$, 369.1 [M-H, ^{10}B, ^{37}Cl, ^{37}Cl, ^{37}Cl, 8%]$^{-}$, 370.1 [M-H, ^{11}B, ^{37}Cl, ^{37}Cl, ^{37}Cl, 3%]$^{-}$; MS(ES^{+}) (*m/z*): 286.9 [M-Ph, ^{10}B, ^{35}Cl, ^{35}Cl, ^{35}Cl, 18%]$^{+}$, 288.0 [M-Ph, ^{11}B, ^{35}Cl, ^{35}Cl, ^{35}Cl, 100%]$^{+}$, 289.0 [M-Ph, ^{10}B, ^{35}Cl, ^{35}Cl, ^{37}Cl, 30%]$^{+}$, 289.9 [M-Ph, ^{11}B, ^{35}Cl, ^{35}Cl, ^{37}Cl, 94%]$^{+}$, 290.9 [M-Ph, ^{10}B, ^{35}Cl, ^{37}Cl, ^{37}Cl, 13%]$^{+}$, 291.9 [M-Ph, ^{11}B, ^{35}Cl, ^{37}Cl, ^{37}Cl, 29%]$^{+}$, 293.0 [M-Ph, ^{10}B, ^{37}Cl, ^{37}Cl, ^{37}Cl, 3%]$^{+}$, 294.0 [M-Ph, ^{11}B, ^{37}Cl, ^{37}Cl, ^{37}Cl, 5%]$^{+}$, 365.0 [M+H, ^{10}B, ^{35}Cl, ^{35}Cl, ^{35}Cl, 3%]$^{+}$, 366.0 [M+H, ^{11}B, ^{35}Cl, ^{35}Cl, ^{35}Cl, 12%]$^{+}$, 387.0 [M+Na, ^{10}B, ^{35}Cl, ^{35}Cl, ^{35}Cl, 16%]$^{+}$ 388.0 [M+Na, ^{11}B, ^{35}Cl, ^{35}Cl, ^{35}Cl, 46%]$^{+}$, 389.0 [M+Na, ^{10}B, ^{35}Cl, ^{35}Cl, ^{37}C;, 16%]$^{+}$, 390.0 [M+Na, ^{11}B, ^{35}Cl, ^{35}Cl, ^{37}Cl, 42%]$^{+}$, 391.0 [M+Na, ^{10}B, ^{35}Cl, ^{37}Cl, ^{37}Cl, 10%]$^{+}$, 392.0 [M+Na, ^{11}B, ^{35}Cl, ^{37}Cl, ^{37}Cl, 13%]$^{+}$, 393.0 [M+Na, ^{10}B, ^{37}Cl, ^{37}Cl, ^{37}C, 4%]$^{+}$, 394.0 [M+Na, ^{11}B, ^{37}Cl, ^{37}Cl, ^{37}Cl, 2%]$^{+}$, 403.0 [M+K, ^{10}B, ^{35}Cl, ^{35}Cl, ^{35}Cl, 4%]$^{+}$,

404.0 [M+K, ^{11}B, ^{35}Cl, ^{35}Cl, ^{35}Cl, 20%]$^{+}$; 405.0 [M+K, ^{10}B, ^{35}Cl, ^{35}Cl, ^{37}Cl, 6%]$^{+}$, 406.0 [M+K, ^{11}B, ^{35}Cl, ^{35}Cl, ^{37}Cl, 19%]$^{+}$, 407.0 [M+K, ^{10}B, ^{35}Cl, ^{37}Cl, ^{37}Cl, 5%]$^{+}$, 408.0 [M+K, ^{11}B, ^{35}Cl, ^{37}Cl, ^{37}Cl, 7%]$^{+}$, 409.0 [M+K, ^{10}B, ^{37}Cl, ^{37}Cl, ^{37}Cl, 2%]$^{+}$, 410.0 [M+K, ^{37}Cl, ^{37}Cl, ^{37}Cl, 2%]$^{+}$; HRMS(ES^{-}) (*m*/*z*): [M-H]$^{-}$ calcd. for $C_{17}H_{14}{}^{11}B^{35}Cl_3NO$, 364.0240; found, 364.0243, error: 0.8 ppm.

5-Acetyl-6-methyl-2,2-diphenyl-4-(trichloromethyl)-2,3,-dihydro-1,3,2-oxazaborinin-1-ium-2-uide (NBC1). Crude enaminone product was directly borylated using DPBA due to difficulty in purification. Ethyl acetate/*n*-hexane, 1:4. Yield: 92%, yellow oil. ^{1}H NMR (400 MHz, $CDCl_3$): δ 7.30 (dd, *J* = 7.8 Hz & 1.8 Hz, 4H, B-Ph(*o*)), 7.16-7.27 (m, 6H, B-Ph(*m*/*p*), 2.26 (s, 3H, CH_3CO), 2.24 (s, 3H, CH_3CO), NH signal not observed; ^{13}C NMR (100 MHz, $CDCl_3$): δ 198.9 ($CH_3\underline{C}O$), 185.4 ($CH_3\underline{C}O$), 163.3 ($Cl_3C(NH)\underline{C}{=}C$), 131.8 (B-Ph(*o*)), 127.6 (B-Ph(*m*)), 127.1 (B-Ph(*p*)), 111.6 ($Cl_3C(NH)C{=}\underline{C}$), 93.0 ($CCl_3$), 34.5 ($\underline{C}H_3CO$), 23.6 ($\underline{C}H_3CO$), B-Ph(*i*) quaternary signal not observed; IR: 3343 (N-H), 1690 (C=O), 1578 (C=C, conjugated) cm^{-1}; MS(ES^{-}) (*m*/*z*): 405.1 [M-H, ^{10}B, ^{35}Cl, ^{35}Cl, ^{35}Cl, 13%]$^{-}$, 406.2 [M-H, ^{11}B, ^{35}Cl, ^{35}Cl, ^{35}Cl, 78%]$^{-}$, 407.2 [M-H, ^{10}B, ^{35}Cl, ^{35}Cl, ^{37}Cl, 21%]$^{-}$, 408.1 [M-H, ^{11}B, ^{35}Cl, ^{35}Cl, ^{37}Cl, 100%]$^{-}$, 409.1 [M-H, ^{10}B, ^{35}Cl, ^{37}Cl, ^{37}Cl, 22%]$^{-}$, 410.0 [M-H, ^{11}B, ^{35}Cl, ^{37}Cl, ^{37}Cl, 54%]$^{-}$, 411.2 [M-H, ^{10}B, ^{37}Cl, ^{37}Cl, ^{37}Cl, 7%]$^{-}$, 412.1 [M-H, ^{11}B, ^{37}Cl, ^{37}Cl, ^{37}Cl, 4%]$^{-}$; HRMS(ES^{-}) (*m*/*z*): [M-H]$^{-}$ calcd. for $C_{19}H_{16}{}^{11}B^{35}Cl_3NO_2$, 406.0345; found, 406.0353, error: 2.0 ppm.

5-(Ethoxycarbonyl)-4,6-dimethyl-2,2-diphenyl-2,3-dihydro-1,3,2-oxazaborinin-1-ium-2-uide (NBC2). Ethyl acetate/*n*-hexane, 1:11. Yield: 82%, white solid. mp: 112-113 ^{o}C; ^{1}H NMR (400 MHz, $CDCl_3$): δ 7.26 (dd, *J* = 7.8 Hz & 1.4 Hz, 4H, B-Ph(*o*)), 7.11-7.23 (m, 6H, B-Ph(*m*/*p*)), 4.11 (q, *J* = 7.2 Hz, 2H, $CH_3C\underline{H}_2O$), 2.42 (s, 3H, CH_3CO), 2.38 (d, *J* = 0.8 Hz, 3H, $C\underline{H}_3(NH)C{=}C$), 1.22 (t, *J* = 7.2 Hz, 3H, $C\underline{H}_3CH_2O$),

NH signal not observed; ^{13}C NMR (75 MHz, $CDCl_3$): δ 186.9 (CH_3<u>*C*</u>O), 170.9 (CH_3(NH)<u>*C*</u>=C), 166.3 (EtO_2C), 131.7 (B-Ph(*o*)), 127.4 (B-Ph(*m*)), 126.6 (B-Ph(*p*)), 103.7 (CH_3(NH)C=<u>*C*</u>), 60.4 (CH_3<u>*C*</u>H_2O), 26.4 (<u>*C*</u>H_3CO), 25.7 (<u>*C*</u>H_3(NH)C=C), 14.3 (<u>*C*</u>H_3CH_2O), B-Ph(*i*) quaternary signal not observed; ^{11}B{^{1}H} (128 MHz, $CDCl_3$): δ 2.63; IR: 3316 (N-H), 1701 (C=O), 1603 (C=C, conjugated), 1489 (C=C-NH) cm^{-1}; MS(ES$^-$) (*m/z*): 333.2 [M-H, ^{10}B, 7%]$^-$, 334.2 [M-H, ^{11}B, 76%]$^-$, 370.3 [M+^{35}Cl, 6%]$^-$, 372.3 [M+^{37}Cl, 2%]$^-$; MS(ES$^+$) (*m/z*): 257.2 [M-Ph, ^{10}B, 21%]$^+$, 258.2 [M-Ph, ^{11}B, 100%]$^+$, 335.4 [M+H, ^{10}B, 5%]$^+$, 336.3 [M+H, ^{11}B, 22%]$^+$, 358.3 [M+Na, ^{11}B, 28%]$^+$, 374.3 [M+K, ^{11}B, 18%]$^+$; HRMS(ES$^+$) (*m/z*): [M+NH_4]$^+$ calcd. for $C_{20}H_{26}{}^{11}BN_2O_3$, 353.2031; found, 353.2021, error: 2.8 ppm.

4,6-Dimethyl-2,2-diphenyl-2,3-dihydro-1,3,2-oxazaborinin-1-ium-2-uide (NBC3). Crude enaminone product was directly borylated using DPBA due to difficulty in purification. Ethyl acetate/*n*-hexane, 2:23. Yield: 10%, white solid. mp: 109-110 ^{o}C; ^{1}H NMR (300 MHz, $CDCl_3$): δ 7.37 (d, *J* = 6.9 Hz, 4H, B-Ph(*o*)), 7.14-7.31 (m, 6H, B-Ph(*m/p*)), 6.67 (br s, 1H, CH_3(N<u>*H*</u>)C=CH), 5.11 (d, *J* = 0.9 Hz, 1H, CH_3(NH)C=C<u>*H*</u>), 2.07 (s, 3H, C<u>*H*</u>$_3$(NH)C=CH), 2.04 (s, 3H, CH_3CO); ^{13}C NMR (75 MHz, $CDCl_3$): δ 179.7 (CH_3<u>*C*</u>O), 169.4 (CH_3(NH)<u>*C*</u>=C), 131.7 (B-Ph(*o*)), 127.3 (B-Ph(*m*)), 126.3 (B-Ph(*p*)), 96.7 (CH_3(NH)C=<u>*C*</u>), 23.8 (<u>*C*</u>H_3(NH)C=C), 23.7 (<u>*C*</u>H_3CO), B-Ph(*i*) quaternary signal not observed; ^{11}B{^{1}H} (128 MHz, $CDCl_3$): δ 2.97; IR: 3339 (N-H), 1621 (C=O), 1537 (C=C-NH) cm^{-1}; MS(ES$^-$) (*m/z*): 261.1 [M-H, ^{10}B, 12%]$^-$, 262.2 [M-H, ^{11}B, 100%]$^-$; MS(ES$^+$) (*m/z*): 185.2 [M-Ph, ^{10}B, 28%]$^+$, 186.2 [M-Ph, ^{11}B, 100%]$^+$, 263.3 [M+H, ^{10}B, 3%]$^+$, 264.3 [M+H, ^{11}B, 5%]$^+$, 285.2 [M+Na, ^{10}B, 2%]$^+$, 286.2 [M+Na, ^{11}B, 8%]$^+$; HRMS(ES$^+$) (*m/z*): [M+Na]$^+$ calcd. for $C_{17}H_{18}{}^{11}$BNONa, 286.1374; found, 286.1392, error: 6.3 ppm.

5-Acetyl-4,6-dimethyl-2,2-diphenyl-2,3-dihydro-1,3,2-oxazaborinin-1-ium-2-uide (NBC4). Ethyl acetate/*n*-hexane, 1:4. Yield: 40%, white solid. mp: 88-89 ^{o}C; ^{1}H NMR (400 MHz, $CDCl_3$): δ 7.26 (dd, *J* = 8.2 Hz & 1.4 Hz, 4H, B-Ph(*o*)), 7.11-7.23 (m, 6H, B-Ph(*m*/*p*)), 2.32 (s, 3H, CH_3CO), 2.28 (d, *J* = 0.8 Hz, 3H, C*H*$_3$(NH)C=C), 2.20 (s, 3H, C*H*$_3$CO), NH signal not observed; ^{13}C NMR (75 MHz, $CDCl_3$): δ 198.2 (CH_3*C*O), 183.5 (CH_3*C*O), 169.9 (CH_3(NH)*C*=C), 131.7 (B-Ph(*o*)), 127.4 (B-Ph(*m*)), 126.6 (B-Ph(*p*)), 114.9 (CH_3(NH)C=*C*), 32.8 (*C*H_3CO), 25.4 (*C*H_3(NH)C=C), 25.0 (*C*H_3CO), B-Ph(*i*) quaternary signal not observed; ^{11}B{^{1}H} (128 MHz, $CDCl_3$): δ 2.64; IR: 3283 (N-H), 1654 (C=O), 1604 (C=C, conjugated) cm^{-1}; MS(ES$^-$) (*m*/*z*): 303.3 [M-H, ^{10}B, ^{12}C, 15%]$^-$, 304.2 [M-H, ^{11}B, ^{12}C, 100%]; MS(ES$^+$) (*m*/*z*): 227.3 [M-Ph, ^{10}B, ^{12}C, 10%]$^+$, 228.2 [M-Ph, ^{11}B, ^{12}C, 40%]$^+$, 305.2 [M+H, ^{10}B, ^{12}C, 3%]$^+$, 306.4 [M+H, ^{11}B, ^{12}C, 45%]$^+$, 328.3 [M+Na, ^{11}B, ^{12}C, 30%]$^+$, 344.4 [M+K, ^{11}B, ^{12}C, 9%]$^+$; HRMS(APCI$^+$) (*m*/*z*): [M+H]$^+$ calcd. for $C_{19}H_{21}{}^{11}BNO_2$, 306.1660; found, 306.1654, error: 2.0 ppm.

6-Amino-2,2-diphenyl-4-(trichloromethyl)-2,3-dihydro-1,3,2-oxazaborinin-1-ium-2-uide (NBC5). Ethyl acetate/*n*-hexane, 1:4. Yield: 81%, cream solid. mp: 148-149 ^{o}C; ^{1}H NMR (300 MHz, $CDCl_3$): δ 7.34 (d, *J* = 6.3 Hz, 4H, B-Ph(*o*)), 7.13-7.29 (m, 6H, B-Ph(*m*/*p*)), 6.29 (br s, 1H, Cl_3C(N*H*)C=CH), 5.28 (br s, 2H, $CONH_2$), 5.14 (s, 1H, Cl_3C(NH)C=C*H*); ^{13}C NMR (75 MHz, $CDCl_3$): δ 171.0 ($CONH_2$), 164.7 (Cl_3C(NH)*C*=C), 132.1 (B-Ph(*o*)), 127.5 (B-Ph(*m*)), 126.7 (B-Ph(*p*)), 93.8 (CCl_3), 86.1 (Cl_3C(NH)C=*C*), B-Ph(*i*) quaternary signal not observed; ^{11}B{^{1}H} (128 MHz, $CDCl_3$): δ 4.32; IR: 3485 (N-H), 3363 (N-H), 1619 (C=O), 1577 (C=C, conjugated), 1542 (C=C-NH) cm^{-1}; MS(ES$^-$) (*m*/*z*): 364.1 [M-H, ^{10}B, ^{35}Cl, ^{35}Cl, ^{35}Cl, 8%]$^-$, 365.1 [M-H, ^{11}B, ^{35}Cl, ^{35}Cl, ^{35}Cl, 100%]$^-$, 366.0 [M-H, ^{10}B, ^{35}Cl, ^{35}Cl, ^{37}Cl, 16%]$^-$, 367.0 [M-H, ^{11}B, ^{35}Cl, ^{35}Cl, ^{37}Cl, 28%]$^-$, 368.0 [M-H, ^{10}B, ^{35}Cl, ^{37}Cl, ^{37}Cl, 8%]$^-$, 369.0 [M-H, ^{11}B, ^{35}Cl, ^{37}Cl, ^{37}Cl, 27%]$^-$, 370.1 [M-H, ^{10}B, ^{37}Cl, ^{37}Cl, ^{37}Cl, 6%]$^-$, 371.3 [M-H, ^{11}B, ^{37}Cl, ^{37}Cl, ^{37}Cl, 2%]$^-$;

MS(ES)$^+$ (m/z): 389.1 [M+Na, ^{11}B, ^{35}Cl, ^{35}Cl, ^{35}Cl, 5%]$^+$, 391.1 [M+Na, ^{11}B, ^{35}Cl, ^{35}Cl, ^{37}Cl, 8%]$^+$, 393.1 [M+Na, ^{11}B, ^{35}Cl, ^{37}Cl, ^{37}Cl, 3%]$^+$; HRMS(ES$^+$) (m/z): [M+H]$^+$ calcd. for $C_{16}H_{15}{}^{11}B^{35}Cl_3N_2O$, 367.0338; found, 367.0328, error: 2.7 ppm.

5-Acetyl-6-amino-2,2-diphenyl-4-(trichloromethyl)-2,3-dihydro-1,3,2-oxazaborinin-1-ium-2-uide (NBC6, 15). Ethyl acetate/*n*-hexane, 1:9. Yield: 34%, yellow solid. mp: 134-135 ^oC; ^{1}H NMR (300 MHz, $CDCl_3$): δ 9.16 (br s, 1H, $CONH_2$), 7.51 (br s, 1H, $Cl_3C(NH)C{=}C$), 7.31 (d, $J = 6.0$ Hz, 4H, B-Ph(*o*)), 7.13-7.25 (m, 6H, B-Ph(*m*/*p*)), 5.91 (br s, 1H, $CONH_2$), 2.20 (s, 3H, CH_3CO); ^{13}C NMR (75 MHz, $CDCl_3$): δ 197.1 ($CH_3\underline{C}O$), 168.2 ($CONH_2$), 164.5 ($Cl_3C(NH)\underline{C}{=}C$), 130.9 (B-Ph(*o*)), 126.4 (B-Ph(*m*)), 125.8 (B-Ph(*p*)), 97.1 ($Cl_3C(NH)C{=}\underline{C}$), 94.0 ($CCl_3$), 32.9 ($\underline{C}H_3CO$), B-Ph(*i*) quaternary signal not observed; ^{11}B{^{1}H} (128 MHz, $CDCl_3$): δ 2.42; IR: 3390 (N-H), 3329 (N-H), 1647 (C=O), 1606 (C=C, conjugated), 1552 (C=C-NH) cm^{-1}; MS(ES$^-$) (m/z): 202.0 [M-$C_{14}H_{11}BO$, ^{35}Cl, ^{35}Cl, ^{35}Cl, 91%]$^-$, 204.0 [M-$C_{14}H_{11}BO$, ^{35}Cl, ^{35}Cl, ^{37}Cl, 100%]$^-$, 206.0 [M-$C_{14}H_{11}BO$, ^{35}Cl, ^{37}Cl, ^{37}Cl, 15%]$^-$, 208.0 [M-$C_{14}H_{11}BO$, ^{37}Cl, ^{37}Cl, ^{37}Cl, 3%]$^-$; 406.1 [M-H, ^{10}B, ^{35}Cl, ^{35}Cl, ^{35}Cl, 20%]$^-$, 407.1 [M-H, ^{11}B, ^{35}Cl, ^{35}Cl, ^{35}Cl, 61%]$^-$, 408.0 [M-H, ^{10}B, ^{35}Cl, ^{35}Cl, ^{37}Cl, 31%]$^-$, 409.0 [M-H, ^{11}B, ^{35}Cl, ^{35}Cl, ^{37}Cl, 100%]$^-$, 410.2 [M-H, ^{10}B, ^{35}Cl, ^{37}Cl, ^{37}Cl, 24%]$^-$, 411.2 [M-H, ^{11}B, ^{35}Cl, ^{37}Cl, ^{37}Cl, 21%]$^-$, 412.5 [M-H, ^{10}B, ^{37}Cl, ^{37}Cl, ^{37}Cl, 4%]$^-$, 413.2 [M-H, ^{11}B, ^{37}Cl, ^{37}Cl, ^{37}Cl, 3%]$^-$; MS(ES$^+$) (m/z): 330.1 [M-Ph, ^{10}B, ^{35}Cl, ^{35}Cl, ^{35}Cl, 12%]$^+$, 331.2 [M-Ph, ^{11}B, ^{35}Cl, ^{35}Cl, ^{35}Cl, 65%]$^+$, 332.2 [M-Ph, ^{10}B, ^{35}Cl, ^{35}Cl, ^{37}Cl, 8%]$^+$, 333.2 [M-Ph, ^{11}B, ^{35}Cl, ^{35}Cl, ^{37}Cl, 63%]$^+$, 334.2 [M-Ph, ^{10}B, ^{35}Cl, ^{37}Cl, ^{37}Cl, 16%]$^+$, 335.3 [M-Ph, ^{11}B, ^{35}Cl, ^{37}Cl, ^{37}Cl, 33%]$^+$, 336.3 [M-Ph, ^{10}B, ^{37}Cl, ^{37}Cl, ^{37}Cl, 5%]$^+$, 337.3 [M-Ph, ^{11}B, ^{37}Cl, ^{37}Cl, ^{37}Cl, 3%]$^+$, 408.4 [M+H, ^{10}B, ^{35}Cl, ^{35}Cl, ^{35}Cl, 11%]$^+$, 409.2 [M+H, ^{11}B, ^{35}Cl, ^{35}Cl, ^{35}Cl, 73%]$^+$, 410.2 [M+H, ^{10}B, ^{35}Cl, ^{35}Cl, ^{37}C, 16%]$^-$, 411.2 [M+H, ^{11}B, ^{35}Cl, ^{35}Cl, ^{37}Cl, 84%]$^+$, 412.2 [M+H, ^{10}B, ^{35}Cl, ^{37}Cl, ^{37}Cl, 8%]$^+$, 413.2 [M+H, ^{11}B, ^{35}Cl, ^{37}Cl, ^{37}Cl, 17%]$^+$, 414.2 [M+H,

^{10}B, ^{37}Cl, ^{37}Cl, ^{37}Cl, 4%]$^+$, 415.2 [M+H, ^{11}B, ^{37}Cl, ^{37}Cl, ^{37}Cl, 3%]$^+$, 430.2 [M+Na, ^{10}B, ^{35}Cl, ^{35}Cl, ^{35}Cl, 4%]$^+$, 431.2 [M+Na, ^{11}B, ^{35}Cl, ^{35}Cl, ^{35}Cl, 46%]$^+$, 432.1 [M+Na, ^{10}B, ^{35}Cl, ^{35}Cl, ^{37}Cl, 13%]$^+$, 433.2 [M+Na, ^{11}B, ^{35}Cl, ^{35}Cl, ^{37}Cl, 17%]$^+$, 434.2 [M+Na, ^{10}B, ^{35}Cl, ^{37}Cl, ^{37}Cl, 9%]$^+$, 435.2 [M+Na, ^{11}B, ^{35}Cl, ^{37}Cl, ^{37}Cl, 10%]$^+$, 436.2 [M+Na, ^{10}B, ^{37}Cl, ^{37}Cl, ^{37}Cl, 3%]$^+$, 437.2 [M+Na, ^{11}B, ^{37}Cl, ^{37}Cl, ^{37}Cl, 5%]$^+$, 447.2 [M+K, ^{11}B, ^{35}Cl, ^{35}Cl, ^{35}Cl, 18%]$^+$, 449.5 [M+K, ^{11}B, ^{35}Cl, ^{35}Cl, ^{37}Cl, 8%]$^+$; HRMS(ES$^-$) (*m*/*z*): [M-H]$^-$ calcd. for $C_{18}H_{15}{}^{11}B^{35}Cl_3N_2O_2$, 407.0298; found, 407.0288, error: 2.5 ppm. Crystals suitable for X-ray diffraction were grown by slow evaporation of 1:1 *n*-hexane:toluene of NBC6 at room temperature.

5-(Dimethylcarbamoyl)-6-methyl-2,2-diphenyl-4-(trichloromethyl)-2,3-dihydro-1,3,2-oxazaborinin-1-ium-2-uide (NBC11, 16). Ethyl acetate/*n*-hexane, 1:2. Yield: 54%, yellow solid. mp: 112-113 ^oC; ^1H NMR (300 MHz, $CDCl_3$): δ 7.92 (br s, 1H, $Cl_3C(NH)C{=}C$), 7.17-7.44 (m, 10H, Aryl-H), 2.91 (s, 3H, CH_3), 2.32 (s, 3H, CH_3), 2.25 (s, 3H, CH_3); ^{13}C NMR (75 MHz, $CDCl_3$): δ 184.6 ($CH_3\underline{C}O$), 164.9 ($Cl_3C(NH)\underline{C}{=}C$), 160.7 ($\underline{C}ON(CH_3)_2$), 131.5 (B-Ph(*o*)), 130.0 (B-Ph(*o*)), 126.7 (B-Ph(*m*)), 126.3 (B-Ph(*p*)), 126.3 (B-Ph(*m*)), 125.7 (B-Ph(*p*)), 103.8 ($Cl_3C(NH)C{=}\underline{C}$), 91.5 ($CCl_3$), 36.8 ($CON(\underline{C}H_3)_2$), 34.3 ($CON(\underline{C}H_3)_2$), 21.8 ($\underline{C}H_3CO$), B-Ph(*i*) quaternary signal not observed; $^{11}B\{^1H\}$ (128 MHz, $CDCl_3$): δ 3.56; IR: 3301 (N-H), 1626 (C=O), 1585 (C=C, conjugated), 1460 (C=C-NH) cm^{-1}; MS(ES$^-$) (*m*/*z*): 434.1 [M-H, ^{10}B, ^{35}Cl, ^{35}Cl, ^{35}Cl, 24%]$^-$, 435.0 [M-H, ^{11}B, ^{35}Cl, ^{35}Cl, ^{35}Cl, 51%]$^-$, 436.0 [M-H, ^{10}B, ^{35}Cl, ^{35}Cl, ^{37}Cl, 21%]$^-$, 437.1 [M-H, ^{11}B, ^{35}Cl, ^{35}Cl, ^{37}Cl, 100%]$^-$, 438.0 [M-H, ^{10}B, ^{35}Cl, ^{37}Cl, ^{37}Cl, 11%]$^-$, 439.0 [M-H, ^{11}B, ^{35}Cl, ^{37}Cl, ^{37}Cl, 22%]$^-$, 440.0 [M-H, ^{10}B, ^{37}Cl, ^{37}Cl, ^{37}Cl, 6%]$^-$, 441.0 [M-H, ^{11}B, ^{37}Cl, ^{37}Cl, ^{37}Cl, 3%]$^-$; MS(ES$^+$) (*m*/*z*): 358.0 [M-Ph, ^{10}B, ^{35}Cl, ^{35}Cl, ^{35}Cl, 3%]$^+$, 359.0 [M-Ph, ^{11}B, ^{35}Cl, ^{35}Cl, ^{35}Cl, 10%]$^+$, 362.0 [M-Ph, ^{10}B, ^{35}Cl, ^{35}Cl, ^{37}Cl, 4%]$^+$, 361.0 [M-Ph, ^{11}B, ^{35}Cl, ^{35}Cl, ^{37}Cl, 13%]$^+$, 362.0 [M-Ph, ^{10}B, ^{35}Cl, ^{37}Cl, ^{37}Cl,

5%]$^+$, 363.0 [M-Ph, ^{11}B, ^{35}Cl, ^{37}Cl, ^{37}Cl, 6%]$^+$, 436.1 [M+H, ^{10}B, ^{35}Cl, ^{35}Cl, ^{35}Cl, 10%]$^+$, 437.1 [M+H, ^{11}B, ^{35}Cl, ^{35}Cl, ^{35}Cl, 70%]$^+$, 438.0 [M+H, ^{10}B, ^{35}Cl, ^{35}Cl, ^{37}Cl, 27%]$^+$, 439.0 [M+H, ^{11}B, ^{35}Cl, ^{35}Cl, ^{37}Cl, 45%]$^+$, 440.1 [M+H, ^{10}B, ^{35}Cl, ^{37}Cl, ^{37}Cl, 19%]$^+$, 441.1 [M+H, ^{11}B, ^{35}Cl, ^{37}Cl, ^{37}Cl, 26%]$^+$, 442.1 [M+H, ^{10}B, ^{37}Cl, ^{37}Cl, ^{37}Cl, 5%]$^+$, 443.1 [M+H, ^{11}B, ^{37}Cl, ^{37}Cl, ^{37}Cl, 2%]$^+$; HRMS(ES$^-$) (*m*/*z*): [M-H]$^-$ calcd. for $C_{20}H_{19}{}^{11}B^{35}Cl_3N_2O_2$, 435.0611; found, 435.0616, error: 1.2 ppm. Crystals suitable for X-ray diffraction were grown by slow evaporation of 1:1 *n*-hexane:toluene of NBC11 at room temperature.

6-Amino-5-cyano-2,2-diphenyl-4-(trichloromethyl)-2,3-dihydro-1,3,2-oxazaborinin-1-ium-2-uide (NBC18). Ethyl acetate/*n*-hexane, 1:9. Yield: 74%, grey solid. mp: 153-155 ^{o}C; ^{1}H NMR (300 MHz, $CDCl_3$): δ 7.66 (br s, 1H, $Cl_3C(NH)C{=}C$), 7.16-7.30 (m, 10H, Aryl-H), 6.48 (br s, 1H, $CONH_2$), 5.98 (br s, 1H, $CONH_2$); ^{13}C NMR (75 MHz, $CDCl_3$): δ 170.5 ($Cl_3C(NH)\underline{C}{=}C$), 163.9 (CONH), 130.6 (B-Ph(*o*)), 126.7 (B-Ph(*m*)), 126.3 (B-Ph(*p*)), 114.4 (CN), 90.8 (CCl_3), 63.3 ($Cl_3C(NH)C{=}\underline{C}$), B-Ph(*i*) quaternary signal not observed; ^{11}B{^{1}H} (128 MHz, $CDCl_3$): δ 4.59; IR: 3385 (N-H), 3353 (N-H), 2227 (C≡N), 1649 (C=O), 1596 (C=C, conjugated), 1524 (C=C-NH) cm^{-1}; MS(ES$^-$) (*m/z*): 389.1 [M-H, ^{10}B, ^{35}Cl, ^{35}Cl, ^{35}Cl, 19%]$^-$, 390.1 [M-H, ^{11}B, ^{35}Cl, ^{35}Cl, ^{35}Cl, 100%]$^-$, 391.1 [M-H, ^{10}B, ^{35}Cl, ^{35}Cl, ^{37}Cl, 22%]$^-$, 392.1 [M-H, ^{11}B, ^{35}Cl, ^{35}Cl, ^{37}Cl, 63%]$^-$, 393.1 [M-H, ^{10}B, ^{35}Cl, ^{37}Cl, ^{37}Cl, 21%]$^-$, 394.1 [M-H, ^{11}B, ^{35}Cl, ^{37}Cl, ^{37}Cl, 27%]$^-$, 395.1 [M-H, ^{10}B, ^{37}Cl, ^{37}Cl, ^{37}Cl, 2%]$^-$, 396.2 [M-H, ^{11}B, ^{37}Cl, ^{37}Cl, ^{37}Cl, 3%]$^-$; MS(ES$^+$) (*m*/*z*): 413.0 [M+Na, ^{10}B, ^{35}Cl, ^{35}Cl, ^{35}Cl, 14%]$^+$, 414.0 [M+Na, ^{11}B, ^{35}Cl, ^{35}Cl, ^{35}Cl, 67%]$^+$, 415.0 [M+Na, ^{10}B, ^{35}Cl, ^{35}Cl, ^{37}Cl, 24%]$^+$, 416.0 [M+Na, ^{11}B, ^{35}Cl, ^{35}Cl, ^{37}Cl, 100%]$^+$, 417.0 [M+Na, ^{10}B, ^{35}Cl, ^{37}Cl, ^{37}Cl, 11%]$^+$, 418.1 [M+Na, ^{11}B, ^{35}Cl, ^{37}Cl, ^{37}Cl, 18%]$^+$, 419.1 [M+Na, ^{10}B, ^{37}Cl, ^{37}Cl, ^{37}Cl, 4%]$^+$, 420.1 [M+Na, ^{11}B, ^{37}Cl,

^{37}Cl, ^{37}Cl, 3%]$^+$. HRMS(APCI$^+$) (*m/z*): [M-Ph]$^+$ calcd. for $C_{11}H_8{}^{11}B^{35}Cl_3N_3O$, 313.9821; found, 313.9823, error: 0.6 ppm.

5-Acetyl-6-methyl-2,2,4-triphenyl-2,3-dihydro-1,3,2-oxazaborinin-1-ium-2-uide (NBC23). Crude enaminone product was directly borylated using DPBA due to difficulty in purification. Ethyl acetate/*n*-hexane, 1:9. Yield: 17%, white solid. mp: 160-163 ^oC; ^1H NMR (300 MHz, $CDCl_3$): δ 7.41-7.59 (m, 5H, Aryl-H), 7.36 (d, J = 6.6 Hz, 4H, B-Ph(*o*)), 7.13-7.27 (m, 6H, B-Ph(*m*/*p*)), 7.04 (br s, 1H, C=C(NH)Ph), 2.50 (s, 3H, CH_3CO), 1.50 (s, 3H, CH_3CO); ^{13}C NMR (75 MHz, $CDCl_3$): δ 196.6 ($CH_3\underline{C}O$), 186.0 ($CH_3\underline{C}O$), 169.0 (C=$\underline{C}$(NH)Ph), 135.4 (Ph(*i*)), 131.8 (Ph(*p*)), 130.8 (B-Ph(*o*)), 128.7 (Ph(*m*)), 126.8 (Ph(*o*)), 126.4 (B-Ph(*m*)), 125.6 (B-Ph(*p*)), 113.9 ($\underline{C}$=C(NH)Ph), 31.3 ($\underline{C}H_3CO$), 23.5 ($\underline{C}H_3CO$); B-Ph(*i*) quaternary signal not observed; $^{11}B\{^1H\}$ (128 MHz, $CDCl_3$): δ 2.59; IR: 3208 (N-H), 1638 (C=O), 1628 (C=O) cm^{-1}; MS(ES$^-$) (*m/z*): 366.0 [M-H, ^{11}B, 100%]$^-$; MS(ES$^+$) (*m/z*): 368.0 [M+H, ^{11}B, 100%]$^+$, 390.0 [M+Na, ^{11}B, 70%]$^+$; HRMS(ES$^+$) (*m/z*): [M+Na]$^+$ calcd. for $C_{24}H_{22}{}^{11}BNO_2Na$, 390.1636; found, 390.1634, error: 0.5 ppm.

6-Amino-5-carbamoyl-2,2-diphenyl-4-(trichloromethyl)-2,3-dihydro-1,3,2-oxazaborinin-1-ium-2-uide (NBC24). Crude enaminone product was directly borylated using DPBA due to difficulty in purification. Ethyl acetate/*n*-hexane, 1:9. Yield: 13%, yellow solid. mp: 155-156 ^oC; ^1H NMR (300 MHz, DMSO-d_6): δ 8.80 (br s, 1H, $CONH_2$), 8.21 (br s, 1H, $CONH_2$), 7.33 (d, J = 6.6 Hz, 4H, B-Ph(*o*)), 7.05-7.24 (m, 6H, B-Ph(*m*/*p*)), $CONH_2$ signal not observed; ^{13}C NMR (75 MHz, DMSO-d_6): δ 169.0 ($CONH_2$), 167.5 ($Cl_3C(NH)\underline{C}$=C), 160.2 ($CONH_2$), 131.6 (B-Ph(*o*)), 126.7 (B-Ph(*m*)), 125.7 (B-Ph(*p*)), 93.5 (CCl_3), 89.6 ($Cl_3C(NH)C$=$\underline{C}$), B-Ph(*i*) quaternary signal not observed; IR: 3484 (N-H), 3419 (N-H), 3345 (N-H), 3318 (N-H), 1653 (C=O), 1614 (C=C, conjugated), 1559 (C=C-NH) cm^{-1}; MS(ES$^-$) (*m/z*): 407.0 [M-H, ^{10}B, ^{35}Cl,

^{35}Cl, ^{35}Cl, 12%]$^-$, 408.0 [M-H, ^{11}B, ^{35}Cl, ^{35}Cl, ^{35}Cl, 100%]$^-$, 409.0 [M-H, ^{10}B, ^{35}Cl, ^{35}Cl, ^{37}Cl, 19%]$^-$, 410.0 [M-H, ^{11}B, ^{35}Cl, ^{35}Cl, ^{37}Cl, 68%]$^-$, 411.0 [M-H, ^{10}B, ^{35}Cl, ^{37}Cl, ^{37}Cl, 5%]$^-$, 412.0 [M-H, ^{11}B, ^{35}Cl, ^{37}Cl, ^{37}Cl, 21%]$^-$, 413.0 [M-H, ^{10}B, ^{37}Cl, ^{37}Cl, ^{37}Cl, 3%]$^-$, 414.0 [M-H, ^{11}B, ^{37}Cl, ^{37}Cl, ^{37}Cl, 3%]$^-$; MS(ES$^+$) (*m*/*z*): 330.9 [M-Ph, ^{10}B, ^{35}Cl, ^{35}Cl, ^{35}Cl, 6%]$^+$, 331.9 [M-Ph, ^{11}B, ^{35}Cl, ^{35}Cl, ^{35}Cl, 62%]$^+$, 332.9 [M-Ph, ^{10}B, ^{35}Cl, ^{35}Cl, ^{37}Cl, 10%]$^+$, 333.9 [M-Ph, ^{11}B, ^{35}Cl, ^{35}Cl, ^{37}Cl, 34%]$^+$, 334.9 [M-Ph, ^{10}B, ^{35}Cl, ^{37}Cl, ^{37}Cl, 3%]$^+$, 335.9 [M-Ph, ^{11}B, ^{35}Cl, ^{37}Cl, ^{37}Cl, 8%]$^+$, 337.0 [M-Ph, ^{10}B, ^{37}Cl, ^{37}Cl, ^{37}, 3%]$^+$], 338.1 [M-Ph, ^{11}B, ^{37}Cl, ^{37}Cl, ^{37}Cl, 4%]$^+$, 409.0 [M+H, ^{10}B, ^{35}Cl, ^{35}Cl, ^{35}Cl, 5%]$^+$, 410.0 [M+H, ^{11}B, ^{35}Cl, ^{35}Cl, ^{35}Cl, 11%]$^+$, 411.0 [M+H, ^{10}B, ^{35}Cl, ^{35}Cl, ^{37}Cl, 7%]$^+$, 412.0 [M+H, ^{11}B, ^{35}Cl, ^{35}Cl, ^{37}Cl, 12%]$^+$, 413.0 [M+H, ^{10}B, ^{35}Cl, ^{37}Cl, ^{37}Cl, 3%]$^+$, 414.1 [M+H, ^{11}B, ^{35}Cl, ^{37}Cl, ^{37}Cl, 4%]$^+$, 415.1 [M+H, ^{10}B, ^{37}Cl, ^{37}Cl, ^{37}Cl, 2%]$^+$, 416.1 [M+H, ^{11}B, ^{37}Cl, ^{37}Cl, ^{37}Cl, 1%]$^+$, 431.0 [M+Na, ^{10}B, ^{35}Cl, ^{35}Cl, ^{35}Cl, 3%]$^+$, 432.0 [M+Na, ^{11}B, ^{35}Cl, ^{35}Cl, ^{35}Cl, 40%]$^+$, 433.0 [M+Na, ^{10}B, ^{35}Cl, ^{35}Cl, ^{37}Cl, 8%]$^+$, 434.0 [M+Na, ^{11}B, ^{35}Cl, ^{35}Cl, ^{37}Cl 18%]$^+$, 435.0 [M+Na, ^{10}B, ^{35}Cl, ^{37}Cl, ^{37}Cl, 5%]$^+$, 436.0 [M+Na, ^{11}B, ^{35}Cl, ^{37}Cl, ^{37}Cl, 6%]$^+$, 437.0 [M+Na, ^{10}B, ^{37}Cl, ^{37}Cl, ^{37}Cl, 2%]$^+$, 438.0 [M+Na, ^{11}B, ^{37}Cl, ^{37}Cl, ^{37}Cl, 1%]$^+$; HRMS(APCI$^+$) (*m*/*z*): [M+H]$^+$ calcd. for $C_{17}H_{16}{}^{11}B^{35}Cl_3N_3O_2$, 410.0396; found, 410.0396, error: 0.1 ppm.

5-(*tert*-Butoxycarbonyl)-2,2-diphenyl-6-methyl-4-(trichloromethyl)-2,3-dihydro-1,3,2-oxazaborinin-1-ium-2-uide (NBC25). Ethyl acetate/*n*-hexane, 1:9. Yield: 35%, yellow solid. mp: 147-149 ^{o}C; ^{1}H NMR (300 MHz, $CDCl_3$): δ 7.86 (br s, 1H, C=C(NH)CCl_3), 7.15-7.32 (m, 10H, 2 x Aryl-H), 2.34 (s, 3H, CH_3CO), 1.42 (s, 9H, $(CH_3)_3$C); ^{13}C NMR (75 MHz, $CDCl_3$): δ 187.2 (CH_3<u>C</u>O), 164.4 (C=<u>C</u>(NH)CCl_3), 163.3 (COO), 131.7 (B-Ph(*o*)), 127.5 (B-Ph(*m*)), 126.9 (B-Ph(*p*)), 103.8 (<u>C</u>=C(NH)CCl_3), 93.3 (CCl_3), 82.8 (($CH_3)_3$<u>C</u>), 27.8 ((<u>C</u>$H_3)_3$C), 24.3 (<u>C</u>H_3CO); B-Ph(*i*) quaternary signal not observed; ^{11}B{^{1}H} (128 MHz, $CDCl_3$): δ 3.96; IR: 3310 (N-H), 1715 (C=O), 1597

(C=C, conjugated) cm^{-1}; MS(ES$^-$) (*m/z*): 463.1 [M-H, ^{10}B, ^{35}Cl, ^{35}Cl, ^{35}Cl, 8%]$^-$, 464.1 [M-H, ^{11}B, ^{35}Cl, ^{35}Cl, ^{35}Cl, 64%]$^-$, 465.1 [M-H, ^{10}B, ^{35}Cl, ^{35}Cl, ^{37}Cl, 32%]$^-$, 466.1 [M-H, ^{11}B, ^{35}Cl, ^{35}Cl, ^{37}Cl, 100%]$^-$, 467.1 [M-H, ^{10}B, ^{35}Cl, ^{37}Cl, ^{37}Cl, 32%]$^-$, 468.1 [M-H, ^{11}B, ^{35}Cl, ^{37}Cl, ^{37}Cl, 20%]$^-$, 469.1 [M-H, ^{10}B, ^{37}Cl, ^{37}Cl, ^{37}Cl, 5%]$^-$, 470.1 [M-H, ^{11}B, ^{37}Cl, ^{37}Cl, ^{37}C, 3%]$^-$; HRMS(ES$^+$) (*m/z*): [M+Na]$^+$ calcd. for $C_{22}H_{23}{}^{11}B^{35}Cl_3NO_3Na$, 488.0729; found, 488.0727, error: 0.4 ppm.

5-(*tert*-Butoxycarbonyl)-4-(ethylthio)-2,2-diphenyl-6-methyl-2,3-dihydro-1,3,2-oxazaborinin-1-ium-2-uide (NBC26). Crude enaminone product was directly borylated using DPBA due to difficulty in purification. Ethyl acetate/*n*-hexane, 1:9. Yield: 30%, white solid. mp: 145-148 ^{o}C; ^{1}H NMR (300 MHz, $CDCl_3$): δ 7.11-7.30 (m, 10H, Aryl-H), 6.73 (br s, 1H, C=C(N*H̲*)SCH_2CH_3), 2.74 (q, *J* = 7.4 Hz, 2H, $CH_3C\underline{H}_2S$), 2.40 (s, 3H, CH_3CO), 1.44 (s, 9H, $(CH_3)_3C$), 1.33 (t, *J* = 7.5 Hz, 3H, $C\underline{H}_3CH_2S$); ^{13}C NMR (100 MHz, $CDCl_3$): δ 184.2 ($CH_3\underline{C}O$), 173.5 (COO), 165.6 (C=$\underline{C}$(NH)SCH_2CH_3), 131.9 (B-Ph(*o*)), 127.5 (B-Ph(*m*)), 126.7 (B-Ph(*p*)), 104.4 ($\underline{C}$=C(NH)CCl_3), 82.2 ($(CH_3)_3\underline{C}$), 28.5 ($(\underline{C}H_3)_3C$), 26.2 ($CH_3\underline{C}H_2S$), 24.4 ($\underline{C}H_3CO$), 12.0 ($\underline{C}H_3CH_2S$); B-Ph(*i*) quaternary signal not observed; ^{11}B{^{1}H} (128 MHz, $CDCl_3$): δ 3.00; IR: 3364 (N-H), 1660 (C=O), 1556 (C=C, conjugated), 1552 (C=C-NH) cm^{-1}; MS(ES$^-$) (*m/z*): 408.2 [M-H, ^{11}B, 100%]$^-$; HRMS(ES$^-$) (*m/z*): [M+Na]$^+$ calcd. for $C_{23}H_{28}{}^{11}BNO_3SNa$, 432.1775; found, 432.1774, error: 0.2 ppm.

4-(*tert*-Butyl)-6-methyl-2,2-diphenyl-2,3-dihydro-1,3,2-oxazaborinin-1-ium-2-uide (NBC28). Crude enaminone product was directly borylated using DPBA due to difficulty in purification. Ethyl acetate/*n*-hexane, 2:23. Yield: 3%, white solid. mp: 121-122 ^{o}C; ^{1}H NMR (300 MHz, $CDCl_3$): δ 7.29 (d, *J* = 6.6 Hz, 4H, B-Ph(*o*)), 7.08-7.23 (m, 6H, B-Ph(*m/p*)), 6.78 (br s, 1H, C=C(N*H̲*)$C(CH_3)_3$), 5.23 (d, *J* = 2.1 Hz, 1H, *H̲*C=C(NH)CCl_3), 2.02 (s, 3H, CH_3CO), 1.16 (s, 9H, $(CH_3)_3C$); ^{13}C NMR (75 MHz,

$CDCl_3$): δ 179.9, 179.8, 131.8 (B-Ph(*o*)), 127.3 (B-Ph(*m*)), 126.3 (B-Ph(*p*)), 93.7 (<u>*C*</u>=C(NH)CCl_3), 37.1 ((CH_3)$_3$<u>*C*</u>), 27.7 ((<u>*C*</u>H_3)$_3$C), 24.1 (<u>*C*</u>H_3CO); B-Ph(*i*) quaternary signal not observed; $^{11}B\{^1H\}$ (128 MHz, $CDCl_3$): δ 2.68; IR: 3381 (N-H), 1610 (C=C, conjugated), 1519 (C=C-NH) cm^{-1}; MS(ES^-) (*m/z*): 303.1 [M-H, ^{10}B, 27%]$^-$, 304.1 [M-H, ^{11}B, 100%]$^-$; MS(ES^+) (*m/z*): 227.1 [M-Ph, ^{10}B, 52%]$^+$, 228.1 [M-Ph, ^{11}B, 100%]$^+$, 305.2 [M+H, ^{10}B, 19%]$^+$, 306.1 [M+H, ^{11}B, 77%]$^+$, 327.1 [M+Na, ^{10}B, 5%]$^+$, 328.1 [M+Na, ^{11}B, 24%]$^+$; HRMS(ES^+) (*m/z*): [M+Na]$^+$ calcd. for $C_{20}H_{24}{}^{11}BNONa$, 328.1843; found, 328.1841, error: 0.6 ppm.

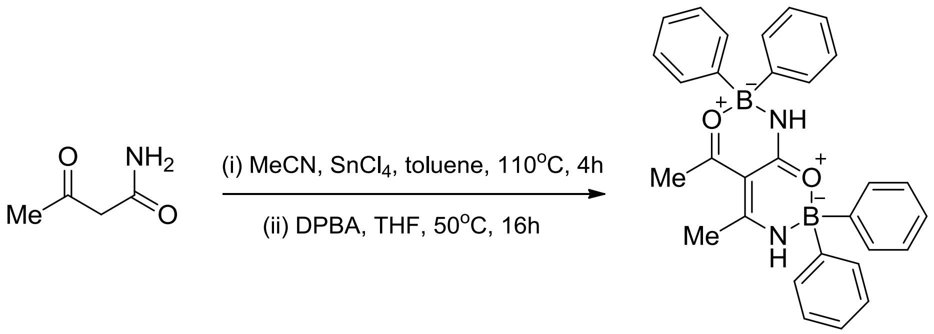

NBC8

Scheme 3 | Synthesis of NBC8.

4,5-Dimethyl-2,2,7,7-tetraphenyl-2,3,7,8-tetrahydro-[1,3,2]oxazaborinino[6,5-*d*][1,3,2]oxazaborinine-1,6,-diium-2,7-diuide (NBC8). Tin(IV) chloride (1.17 ml, 10 mmol) was slowly added to a stirring solution of acetoacetamide (1.01 g, 10 mmol) and acetonitrile (0.52 ml, 10 mmol) suspended in anhydrous toluene (10 ml). A white solid appeared and the reaction mixture was stirred at room temperature under N_2 for 30 min. The mixture was then heated at reflux at 110 ^oC under N_2 for 4 h. A yellow solid precipitated from the reaction mixture. The mixture was cooled to room temperature, washed with *n*-hexane (20 ml), decanted and then dried *in vacuo* overnight to give crude (*Z*)-3-acetyl-4-amino-3-penten-2-amide as a yellow oil. The crude product (0.42 g, 2.95 mmol) was added to DPBA (0.34 g, 0.98 mmol)

suspended in anhydrous THF (4 ml). The mixture was heated at 50 ^{o}C under N_2 for 16 h. The reaction mixture was extracted with DCM and extract dried over $MgSO_4$. The solution was concentrated and purified by flash column chromatography. Ethyl acetate/*n*-hexane, 1:9. Yield: 3%, cream solid. No other products were isolated. mp: 175-178 ^{o}C; ^{1}H NMR (400 MHz, $CDCl_3$): δ 7.12-7.29 (m, 20H, Aryl-H), 6.52 (br s, 1H, $CH_3(N\underline{H})C{=}C$), 2.43 (d, *J* = 0.8 Hz, 3H, $C\underline{H}_3(NH)C{=}C$), 2.41 (s, 3H, CH_3CO), NH broad signal was not observed; ^{13}C NMR (100 MHz, $CDCl_3$): δ 188.2 ($CH_3\underline{C}O$), 170.3 (CONH), 164.5 ($CH_3(NH)\underline{C}{=}C$), 131.7 (B-Ph(*o*)), 131.6 (B-Ph(*o*)), 127.7 (B-(*m*)), 127.4 (B-Ph(*m*)), 126.9 (B-Ph(*p*)), 126.5 (B-Ph(*p*)), 27.5 ($\underline{C}H_3(NH)C{=}C$), 27.1 ($\underline{C}H_3CO$), B-Ph(*i*) quaternary signal not observed; IR: 3300 (N-H), 1619 (C=O), 1473 (C=C-NH) cm^{-1}; MS(ES^{-}) (*m/z*): 467.4 [M-H, ^{10}B, ^{10}B, ^{12}C, 5%]$^{-}$, 468.4 [M-H, ^{10}B, ^{11}B, ^{12}C, 30%]$^{-}$, 469.3 [M-H, ^{11}B, ^{11}B, ^{12}C, 100%]$^{-}$; HRMS(ES^{-}) (*m*/*z*): [M-H]$^{-}$ calcd. for $C_{30}H_{27}{}^{11}B_2N_2O_2$, 469.2264; found, 469.2271, error: 1.5 ppm.

1.4 General procedure for dioxaborine reaction

Either acetylacetone or acetoacetamide (3 eq) was added to a solution of DPBA (1 eq) in anhydrous THF (5 ml). The mixture was stirred at 50°C under N_2 for 16h. The mixture was concentrated *in vacuo* and purified by flash column chromatography, unless otherwise stated. The collected fractions were combined, evaporated *in vacuo* and stirred in cold *n*-hexane (15 ml) for 30 mins. The precipitate was then filtered and dried under vacuum to give the corresponding dioxaborine products.

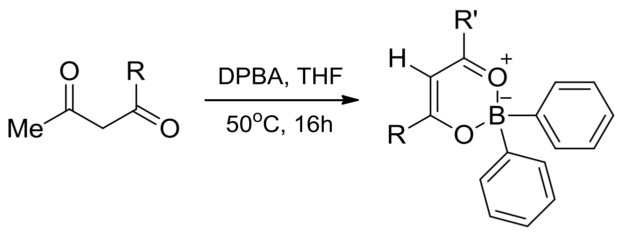

R	R'	
Me	NH_2	**NBC7**
CCl_3	NH_2	**NBC9**
Me	Me	**NBC10**

Scheme 4 | Synthesis of NBC7, NBC9 and NBC10.

6-Amino-4-methyl-2,2-diphenyl-2,3-dihydro-1,3,2-dioxaborinin-1-ium-2-uide (NBC7). Ethyl acetate/*n*-hexane, 1:4. Yield: 2%, white solid. mp: 164-166 °C; ^{1}H NMR (400 MHz, $CDCl_3$): δ 7.38 (d, *J* = 8.8 Hz, 4H, B-Ph(*o*)), 7.07-7.24 (m, 6H, B-Ph(*m*/*p*)), 5.42 (br s, 1H, $CONH_2$), 5.06 (br s, 1H, $CONH_2$), 4.88 (s, 1H, $CH_3C(O)C{=}CH$), 2.03 (s, 1H, CH_3CO); ^{13}C NMR (100 MHz, $CDCl_3$): δ 183.2 ($CONH_2$), 171.0 ($CH_3C(O)\underline{C}{=}C$), 131.6 (B-Ph(*o*)), 127.3 (B-Ph(*m*)), 126.5 (B-Ph(*p*)), 87.1 ($CH_3C(O)C{=}\underline{C}$), 23.9 ($\underline{C}H_3CO$), B-Ph(*i*) quaternary signal not observed; ^{11}B{^{1}H} (128 MHz, $CDCl_3$): δ 8.15; IR: 3429 (N-H), 3314 (N-H), 1637 (C=O), 1577 (C=C, conjugated), 1527 (C=C-NH_2) cm^{-1}; MS(ES$^-$) (*m/z*): 263.2 [M-H, ^{10}B, 18%]$^-$, 264.2 [M-H, ^{11}B, 100%]$^-$; MS(ES$^+$): (*m/z*) 187.3 [M-Ph, ^{10}B, 4%]$^+$, 188.3 [M-Ph, ^{11}B, 15%]$^+$, 265.3 [M+H, ^{10}B, 4%]$^+$, 266.3 [M+H, ^{11}B, 10%]$^+$, 288.2 [M+Na, ^{11}B, 4%]$^+$; HRMS(ES$^-$) (*m/z*): [M-H]$^-$ calcd. for $C_{16}H_{15}{}^{11}BNO_2$, 264.1201; found, 264.1206, error: 1.9 ppm.

6-Amino-2,2-diphenyl-4-(trichloromethyl)-2,3-dihydro-1,3,2-borinin-1-ium-2-uide (NBC9). A by-product obtained during the reaction to form **3g** from **2b**. Ethyl acetate/*n*-hexane, 3:7. Yield: 2%, cream solid. mp: 167-168 °C; ^{1}H NMR (300 MHz, DMSO-d_6): δ 9.69 (br s, 1H, $CONH_2$), 9.53 (br s, 1H, $CONH_2$), 7.39 (d, *J* = 6.3 Hz, 4H, B-Ph(*o*)), 7.07-7.25 (m, 6H, B-Ph(*m*/*p*)), 6.06 (s, 1H, $Cl_3C(O)C{=}CH$); ^{13}C NMR

(75 MHz, DMSO-d$_6$): δ 170.7 ($CONH_2$), 168.7 ($Cl_3C(O)\underline{C}=C$), 131.0 (B-Ph(*o*)), 126.8 (B-Ph(*m*)), 125.9 (B-Ph(*p*)), 86.3 (CCl_3), 64.4 ($Cl_3C(O)C=\underline{C}$), B-Ph(*i*) quaternary signal not observed; IR: 3433 (N-H), 3331 (N-H), 1644 (C=O), 1603 (C=C, conjugated), 1501 ($C=C-NH_2$) cm^{-1}; MS(ES^-) (*m/z*): 365.3 [M-H, ^{10}B, ^{35}Cl, ^{35}Cl, ^{35}Cl, 27%]$^-$, 366.1 [M-H, ^{11}B, ^{35}Cl, ^{35}Cl, ^{35}Cl, 91%]$^-$, 367.1 [M-H, ^{10}B, ^{35}Cl, ^{35}Cl, ^{37}Cl, 32%]$^-$, 368.1 [M-H, ^{11}B, ^{35}Cl, ^{35}Cl, ^{37}Cl, 100%]$^-$, 369.2 [M-H, ^{10}B, ^{35}Cl, ^{37}Cl, ^{37}Cl, 20%]$^-$, 370.4 [M-H, ^{11}B, ^{35}Cl, ^{37}Cl, ^{37}Cl, 19%]$^-$, 371.1 [M-H, ^{10}B, ^{37}Cl, ^{37}Cl, ^{37}Cl, 3%]$^-$, 372.0 [M-H, ^{11}B, ^{37}Cl, ^{37}Cl, ^{37}Cl, 2%]$^-$; MS(ES^+) (*m/z*): 290.0 [M-Ph, ^{11}B, ^{35}Cl, ^{35}Cl, ^{35}C]$^+$, 292.0 [M-Ph, ^{11}B, ^{35}Cl, ^{35}Cl, ^{37}Cl]$^+$, 294.0 [M-Ph, ^{11}B, ^{35}Cl, ^{37}Cl, ^{37}Cl]$^+$; HRMS(ES^-) (*m/z*): [M-H]$^-$ calcd. for $C_{16}H_{12}{}^{11}B^{35}Cl_3N_2O$, 366.0032; found, 366.0036, error: 1.1 ppm.

4,6-Dimethyl-2,2-diphenyl-2,3-dihydro-1,3,2-dioxaborinin-1-ium-2-uide (NBC10). The reaction mixture was extracted with DCM and the extract dried over $MgSO_4$. The solution was concentrated *in vacuo*. The residue was purified by recrystallisation from EtOH. Yield: 9%, yellow solid. mp: 108-110 ^oC; ^1H NMR (400 MHz, $CDCl_3$): δ 7.38 (dd, J = 7.8 Hz & 1.4 Hz, 4H, B-Ph(*o*)), 7.10-7.24 (m, 6H, B-Ph(*m*/*p*)), 5.60 (s, 1H, $CH_3C(O)C=C\underline{H}$), 2.15 (s, 6H, 2 x CH_3CO); ^{13}C NMR (100 MHz, $CDCl_3$) δ 191.1 (2 x $CH_3\underline{C}O$), 131.2 (B-Ph(*o*)), 127.3 (B-Ph(*m*)), 126.7 (B-Ph(*p*)), 102.2 ($CH_3C(O)C=\underline{C}H$), 24.5 (2 x $\underline{C}H_3CO$), B-Ph(*i*) quaternary signal and $CH_3C(O)\underline{C}=CH$ not observed; IR: 1713 (C=O), 1511 (C=C-O) cm^{-1}; MS(ES^-) (*m/z*): 262.2 [M-H, ^{10}B, ^{12}C, 18%]$^-$, 263.1 [M-H, ^{11}B, ^{12}C, 41%]$^-$; MS(ES^+) (*m/z*): 186.1 [M-Ph, ^{10}B, ^{12}C, 29%]$^+$, 187.1 [M-Ph, ^{11}B, ^{12}C, 64%]$^+$, 265.2 [M+H, ^{11}B, 4%]$^+$; HRMS(ES^-) (*m/z*): [M-H]$^-$ calcd. for $C_{17}H_{16}{}^{11}BO_2$, 263.1249; found, 263.1239, error: 3.8 ppm.

General procedure for acetoacetamide-based oxazaborine derivatives

Unless otherwise stated, an appropriate primary amine (1 eq) was added to a solution of *tert*-butyl acetoacetate (1.1 eq) in toluene (150 ml) or DMF (5 ml). The

mixture was stirred at 125°C under N_2 for 16h. Solvent was evaporated *in vacuo* and the residue was dissolved in Et_2O/DCM (20 ml), washed with 1N $HCl_{(aq)}$ (2 x 10 ml), dried over $MgSO_4$ and evaporated *in vacuo*. All further reactions were conducted in the same manner as previously described for the general procedures for the enaminone reaction and oxazaborine reaction to give the corresponding oxazaborine products.

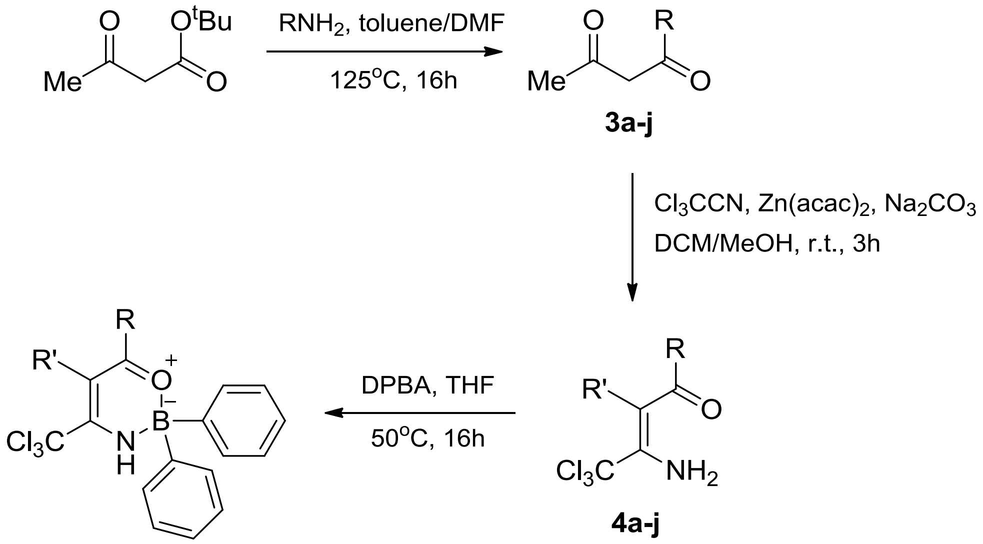

R	R'		R	R'	
NHMe	COMe	**NBC12**	NHAd	COMe	**NBC17**
NHPh	COMe	**NBC13**	NHCy	COMe	**NBC19**
$NHCH_2Ph$	COMe	**NBC14**	$NHCH_2Pyr$	COMe	**NBC20**
$NHCH_2CH_2Ph$	COMe	**NBC15**	NHPy	COMe	**NBC22**
$NHCH_2CH_2Ph$	H	**NBC16**	OEPPS	COMe	**NBC-EPPS**

Scheme 5 | Synthesis of NBC12-17, NBC19-20, NBC22 and NBC-EPPS.

***N*-Methylacetoacetamide (3a)**. Yield: 88%, orange oil. ^{1}H NMR (300 MHz, $CDCl_3$): δ 7.09 (br s, 1H, NH), 3.40 (s, 2H, $CH_3COC\underline{H_2}$), 2.80 (d, *J* = 4.5 Hz, 3H, $C\underline{H_3}NH$), 2.24 (s, 3H, CH_3CO); ^{13}C NMR (100 MHz, $CDCl_3$): δ 204.6 ($CH_3\underline{C}O$), 166.2 (CONH), 49.6 ($CH_3CO\underline{C}H_2$), 30.9 ($\underline{C}H_3CO$), 26.2 (CH_3NH); IR: 3305 (N-H), 1715 (C=O), 1641 (C=O), 1556 (C=O) cm^{-1}; MS(ES^+) (*m*/*z*): 116.0 $[M+H, 59\%]^+$, 138.0 $[M+Na, 81\%]^+$.

HRMS(APCI$^+$) (*m/z*): $[M+H]^+$ calcd. for $C_5H_{10}NO_2$, 116.0706; found, 116.0711, error: 4.3 ppm.

(*Z*)-2-Acetyl-3-amino-4,4,4-trichloro-*N*-methylbut-2-enamide (4a). Yield: 62%, white solid. mp: 112-113 ^oC; ^1H NMR (300 MHz, $CDCl_3$): δ 8.65 (br s, 2H, NH_2), 6.06 (br s, 1H, CONH), 2.85 (d, *J* = 4.8 Hz, C$\underline{H}_3$NH), 2.23 (s, 3H, CH_3CO); ^{13}C NMR (100 MHz, $CDCl_3$): δ 196.7 ($\underline{C}H_3CO$), 167.4 (C=$\underline{C}$(NH_2)CCl_3), 155.7 (CONH), 103.7 ($\underline{C}$=C(NH_2)CCl_3), 92.4 (CCl_3), 27.4 ($\underline{C}H_3CO$), 26.3 (CH_3NH); IR: 3276 (N-H), 1646 (C=O), 1637 (C=O), 1558 (C=O) cm^{-1}; MS(ES$^-$) (*m/z*): 257.0 [M-H, ^{35}Cl, ^{35}Cl, ^{35}Cl, 78%]$^-$, 258.9 [M-H, ^{35}Cl, ^{35}Cl, ^{37}Cl, 100%]$^-$, 261.0 [M-H, ^{35}Cl, ^{37}Cl, ^{37}Cl, 28%]$^-$, 263.0 [M-H, ^{37}Cl, ^{37}Cl, ^{37}Cl, 3%]$^-$, 292.9 [M+^{35}Cl, ^{35}Cl, ^{35}Cl, ^{35}Cl, 24%]$^-$, 295.0 [M+^{35}Cl, ^{35}Cl, ^{35}Cl, ^{37}Cl, 22%]$^-$, 297.0 [M+^{35}Cl, ^{35}Cl, ^{37}Cl, ^{37}Cl, 11%]$^-$, 209.0 [M+^{35}Cl, ^{37}Cl, ^{37}Cl, ^{37}Cl, 3%]$^-$; MS(ES$^+$) (*m/z*): 217.0 [M-C_2H_3O, 33%]$^+$, 259.0 [M+H, ^{35}Cl, ^{35}Cl, ^{35}Cl, 51%]$^+$, 261.0 [M+H, ^{35}Cl, ^{35}Cl, ^{37}Cl, 38%]$^+$, 263.0 [M+H, ^{35}Cl, ^{37}Cl, ^{37}Cl, 17%]$^+$, 265.0 [M+H, ^{37}Cl, ^{37}Cl, ^{37}Cl, 2%]$^+$, 281.0 [M+Na, ^{35}Cl, ^{35}Cl, ^{35}Cl, 70%]$^+$, 283.0 [M+Na, ^{35}Cl, ^{35}Cl, ^{37}Cl, 80%]$^+$, 285.0 [M+Na, ^{35}Cl, ^{37}Cl, ^{37}Cl, 34%]$^+$, 287.0 [M+Na, ^{37}Cl, ^{37}Cl, ^{37}Cl, 4%]$^+$.

5-Acetyl-6-(Methylamino)-2,2-diphenyl-4-(trichloromethyl)-2,3-dihydro-1,3,2-oxazaborinin-1-ium-2-uide (NBC12). Ethyl acetate/*n*-hexane, 3:7. Yield: 16%, yellow solid. mp: 157-158 ^oC (dec); ^1H NMR (300 MHz, $CDCl_3$): δ 7.83 (br s, 1H, Cl_3C(NH)C=C), 7.22-7.42 (m, 10H, Aryl-H), 5.52 (br s, 1H, CH_3NH), 2.82 (d, *J* = 4.8 Hz, 3H, CH_3NH), 2.37 (s, 3H, CH_3CO); ^{13}C NMR (75 MHz, $CDCl_3$): δ 187.5 ($CH_3\underline{C}O$), 165.5 (Cl_3C(NH)$\underline{C}$=C), 162.4 (CONH), 131.8 (B-Ph(*o*)), 127.7 (B-Ph(*m*)), 127.2 (B-Ph(*p*)), 105.6 (Cl_3C(NH)C=$\underline{C}$), 93.0 (CCl_3), 27.3 (CH_3NH), 23.5 ($\underline{C}H_3CO$), B-Ph(*i*) quaternary signal not observed; $^{11}B\{^1H\}$ (128 MHz, $CDCl_3$): δ 3.68; IR: 3313 (N-H), 1638 (C=O), 1593 (C=C, conjugated), 1524 (C=C-NH) cm^{-1}; MS(ES$^-$) (*m/z*): 420.0 [M-H, ^{10}B, ^{35}Cl, ^{35}Cl, ^{35}Cl, 23%]$^-$, 421.0 [M-H, ^{11}B, ^{35}Cl, ^{35}Cl, ^{35}Cl, 100%]$^-$, 422.0 [M-H,

^{10}B, ^{35}Cl, ^{35}Cl, ^{37}Cl, 38%]$^-$, 423.0 [M-H, ^{11}B, ^{35}Cl, ^{35}Cl, ^{37}Cl, 93%]$^-$, 424.0 [M-H, ^{10}B, ^{35}Cl, ^{37}Cl, ^{37}Cl, 25%]$^-$, 425.0 [M-H, ^{11}B, ^{35}Cl, ^{37}Cl, ^{37}Cl, 27%]$^-$, 426.0 [M-H, ^{10}B, ^{37}Cl, ^{37}Cl, ^{37}Cl, 4%]$^-$, 427.0 [M-H, ^{11}B, ^{37}Cl, ^{37}Cl, ^{37}Cl, 2%]$^-$; MS(ES$^+$) (*m*/*z*): 344.0 [M-Ph, ^{10}B, ^{35}Cl, ^{35}Cl, ^{35}Cl, 5%]$^+$, 345.0 [M-Ph, ^{11}B, ^{35}Cl, ^{35}Cl, ^{35}Cl, 43%]$^+$, 346.0 [M-Ph, ^{10}B, ^{35}Cl, ^{35}Cl, ^{37}Cl, 11%]$^+$, 347.0 [M-Ph, ^{11}B, ^{35}Cl, ^{35}Cl, ^{37}Cl, 40%]$^+$, 348.0 [M-Ph, ^{10}B, ^{35}Cl, ^{37}Cl, ^{37}Cl, 8%]$^+$, 349.0 [M-Ph, ^{11}B, ^{35}Cl, ^{37}Cl, ^{37}Cl, 7%]$^+$, 350.0 [M-Ph, ^{10}B, ^{37}Cl, ^{37}Cl, ^{37}Cl, 3%]$^+$, 351.0 [M-Ph, ^{11}B, ^{37}Cl, ^{37}Cl, ^{37}Cl, 6%]$^+$, 422.1 [M+H, ^{10}B, ^{35}Cl, ^{35}Cl, ^{35}Cl, 35%]$^+$, 423.1 [M+H, ^{11}B, ^{35}Cl, ^{35}Cl, ^{35}Cl, 77%]$^+$, 424.0 [M+H, ^{10}B, ^{35}Cl, ^{35}Cl, ^{37}Cl, 60%]$^+$, 425.0 [M+H, ^{11}B, ^{35}Cl, ^{35}Cl, ^{37}Cl, 100%]$^+$, 426.0 [M+H, ^{10}B, ^{35}Cl, ^{37}Cl, ^{35}Cl, 39%]$^+$, 427.0 [M+H, ^{11}B, ^{35}Cl, ^{37}Cl, ^{37}Cl, 48%]$^+$, 428.0 [M+H, ^{10}B, ^{37}Cl, ^{37}Cl, ^{37}Cl, 8%]$^+$, 429.0 [M+H, ^{11}B, ^{37}Cl, ^{37}Cl, ^{37}Cl, 7%]$^+$, 444.0 [M+Na, ^{10}B, ^{35}Cl, ^{35}Cl, ^{35}Cl, 20%]$^+$, 445.0 [M+Na, ^{11}B, ^{35}Cl, ^{35}Cl, ^{35}Cl, 53%]$^+$, 446.0 [M+Na, ^{10}B, ^{35}Cl, ^{35}Cl, ^{37}Cl, 12%]$^+$, 447.0 [M+Na, ^{11}B, ^{35}Cl, ^{35}Cl, ^{37}Cl, 49%]$^+$, 448.0 [M+Na, ^{10}B, ^{35}Cl, ^{37}Cl, ^{37}Cl, 12%]$^+$, 449.0 [M+Na, ^{11}B, ^{35}Cl, ^{37}Cl, ^{37}Cl, 14%]$^+$, 450.0 [M+Na, ^{10}B, ^{37}Cl, ^{37}Cl, ^{37}Cl, 2%]$^+$, 451.0 [M+Na, ^{11}B, ^{37}Cl, ^{37}Cl, ^{37}Cl, 3%]$^+$. HRMS(ES$^+$) (*m*/*z*): [M+Na]$^+$ calcd. for $C_{19}H_{18}{}^{11}B^{35}Cl_3N_2O_2Na$, 445.0419; found, 445.0420, error: 0.2 ppm.

***N*-Phenylacetoacetamide (3b)**. Yield: 56%, orange solid. mp: 81-83 ^oC (lit.(Sridharan et al., 2010) 85-86 ^oC); ^1H NMR (300 MHz, $CDCl_3$): δ 9.13 (br s, 1H, NH), 7.56 (d, *J* = 8.1 Hz, 2H, Ph(*o*)), 7.34 (t, *J* = 7.8 Hz, 2H, Ph(*m*)), 7.14 (t, *J* = 7.4 Hz, 1H, Ph(*p*)), 3.61 (s, 2H, $CH_3COC\underline{H_2}$), 2.34 (s, 3H, CH_3CO); ^{13}C NMR (75 MHz, $CDCl_3$): δ 205.2 ($CH_3\underline{C}O$), 163.3 (CONH), 137.5 (Ph(*i*)), 129.0 (Ph(*m*)), 124.6 (Ph(*p*)), 120.2 (Ph(*o*)), 49.7 ($CH_3CO\underline{C}H_2$), 31.3 ($\underline{C}H_3CO$); IR: 3253 (N-H), 1710 (C=O), 1659 (C=O), 1598 (C=O) cm^{-1}; MS(ES$^-$) (*m*/*z*): 204.0 [M-H, 100%]$^-$; MS(ES$^+$) (*m*/*z*): 164.1 [M-C_2H_3O, 47%]$^+$ 206.1 [M+H, 100%]$^+$, 228.0 [M+Na, 68%]$^+$, 244.0

[M+K, 62%]$^+$. HRMS(APCI$^+$) (*m/z*): [M+H]$^+$ calcd. for $C_{10}H_{12}NO_2$, 178.0863; found, 178.0855, error: 4.5 ppm.

(*Z*)-2-Acetyl-3-amino-4,4,4-trichloro-*N*-phenylbut-2-enamide (4b). Yield: 42%, cream solid. mp: 105-107 ^oC (lit.(Veronese et al., 1986) 139-143 ^oC); ^1H NMR (300 MHz, $CDCl_3$): δ 8.73 (br s, 2H, NH_2), 7.72 (br s, 1H, CONH), 7.50 (d, *J* = 8.1 Hz, 2H, Ph(*o*)), 7.30 (t, *J* = 7.8 Hz, 2H, Ph(*m*)), 7.10 (t, *J* = 7.4 Hz, 1H, Ph(*p*)), 2.32 (s, 3H, CH_3CO); ^{13}C NMR (75 MHz, $CDCl_3$): δ 197.6 ($CH_3\underline{C}O$), 165.5 ($C{=}\underline{C}(NH_2)CCl_3$), 157.0 (CONH), 137.8 (Ph(*i*)), 129.2 (Ph(*m*)), 125.0 (Ph(*p*)), 119.9 (Ph(*o*)), 105.4 ($\underline{C}{=}C(NH_2)CCl_3$), 93.4 ($CCl_3$), 28.6 ($\underline{C}H_3CO$); IR: 3267 (N-H), 1647 (C=O), 1617 (C=O), 1595 (C=C, conjugated), 1528 ($C{=}C{-}NH_2$) cm^{-1}; MS(ES$^-$) (*m/z*): 319.0 [M-H, ^{35}Cl, ^{35}Cl, ^{35}Cl, 100%]$^-$, 321.0 [M-H, ^{35}Cl, ^{35}Cl, ^{37}Cl, 65%]$^-$, 323.0 [M-H, ^{35}Cl, ^{37}Cl, ^{37}Cl, 30%]$^-$, 325.0 [M-H, ^{37}Cl, ^{37}Cl, ^{37}Cl, 3%]$^-$; MS(ES$^+$) (*m/z*): 227.9 [M-C_6H_6N, ^{35}Cl, ^{35}Cl, ^{35}Cl, 61%]$^+$, 229.9 [M-C_6H_6N, ^{35}Cl, ^{35}Cl, ^{37}Cl, 47%]$^+$, 231.9 [M-C_6H_6N, ^{35}Cl, ^{37}Cl, ^{37}Cl, 12%]$^+$, 233.0 [M-C_6H_6N, ^{37}Cl, ^{37}Cl, ^{37}Cl, 5%]$^+$, 279.1 [M-C_2H_3O, ^{35}Cl, ^{35}Cl, ^{35}Cl, 34%]$^+$, 281.0 [M-C_2H_3O, ^{35}Cl, ^{35}Cl, ^{37}Cl, 4%]$^+$, 283.0 [M-C_2H_3O, ^{35}Cl, ^{37}Cl, ^{37}Cl, 3%]$^+$, 285.0 [M-C_2H_3O, ^{37}Cl, ^{37}Cl, ^{37}Cl, 2%]$^+$, 321.0 [M+H, ^{35}Cl, ^{35}Cl, ^{35}Cl, 100%]$^+$, 323.0 [M+H, ^{35}Cl, ^{35}Cl, ^{37}Cl, 72%]$^+$, 325.0 [M+H, ^{35}Cl, ^{37}Cl, ^{37}Cl, 18%]$^+$, 327.0 [M+H, ^{37}Cl, ^{37}Cl, ^{37}Cl, 2%]$^+$, 343.0 [M+Na, ^{35}Cl, ^{35}Cl, ^{35}Cl, 24%]$^+$, 345.0 [M+Na, ^{35}Cl, ^{35}Cl, ^{37}Cl, 15%]$^+$, 347.0 [M+Na, ^{35}Cl, ^{37}Cl, ^{37}Cl, 4%]$^+$, 349.0 [M+Na, ^{37}Cl, ^{37}Cl, ^{37}Cl, 2%]$^+$.

5-Acetyl-6-(phenylamino)-2,2-diphenyl-4-(trichloromethyl)-2,3-dihydro-1,3,2-oxazaborinin-1-ium-2-uide (NBC13). Ethyl acetate/*n*-hexane, 1:7. Yield: 41%, yellow solid. mp: 131-133 ^oC; ^1H NMR (300 MHz, $CDCl_3$): δ 7.95 (br s, 1H, $C{=}C(NH)CCl_3$), 7.10-7.46 (m, 16H, 3 x Aryl-H and CONH), 2.44 (s, 3H, CH_3CO); ^{13}C NMR (75 MHz, $CDCl_3$): δ 187.6 ($CH_3\underline{C}O$), 162.5 ($C{=}\underline{C}(NH)CCl_3$), 162.4 (CONH), 137.4 (Ph(*i*)), 131.7 (B-Ph(*o*)), 129.2 (Ph(*m*)), 127.6 (B-Ph(*m*)), 127.2 (Ph(*p*)), 125.1

(B-Ph(*p*)), 119.7 (Ph(*o*)), 106.4 ($\underline{C}$=C(NH)CCl_3), 92.8 (CCl_3), 23.4 ($\underline{C}H_3CO$), B-Ph(*i*) quaternary signal not observed; $^{11}B\{^1H\}$ (128 MHz, $CDCl_3$): δ 3.77; IR: 3350 (N-H), 1654 (C=O), 1596 (C=C, conjugated), 1520 (C=C-NH) cm^{-1}; MS(ES^-) (*m/z*): 482.2 [M-H, ^{10}B, ^{35}Cl, ^{35}Cl, ^{35}Cl, 13%]$^-$, 483.2 [M-H, ^{11}B, ^{35}Cl, ^{35}Cl, ^{35}Cl, 63%]$^-$, 484.2 [M-H, ^{10}B, ^{35}Cl, ^{35}Cl, ^{37}Cl, 29%]$^-$, 485.1 [M-H, ^{11}B, ^{35}Cl, ^{35}Cl, ^{37}Cl, 100%]$^-$, 486.2 [M-H, ^{10}B, ^{35}Cl, ^{37}Cl, ^{37}Cl, 15%]$^-$, 487.2 [M-H, ^{11}B, ^{35}Cl, ^{37}Cl, ^{37}Cl, 23%]$^-$, 488.1 [M-H, ^{10}B, ^{37}Cl, ^{37}Cl, ^{37}Cl, 3%]$^-$, 489.1 [M-H, ^{11}B, ^{37}Cl, ^{37}Cl, ^{37}C, 1%]$^-$; MS(ES^+) (*m/z*): 484.1 [M+H, ^{10}B, ^{35}Cl, ^{35}Cl, ^{35}Cl, 5%]$^+$, 485.1 [M+H, ^{11}B, ^{35}Cl, ^{35}Cl, ^{35}Cl, 37%]$^+$, 486.1 [M+H, ^{10}B, ^{35}Cl, ^{35}Cl, ^{37}Cl, 12%]$^+$, 487.1 [M+H, ^{11}B, ^{35}Cl, ^{35}Cl, ^{37}Cl, 28%]$^+$, 488.1 [M+H, ^{10}B, ^{35}Cl, ^{37}Cl, ^{35}Cl, 4%]$^+$, 489.1 [M+H, ^{11}B, ^{35}Cl, ^{37}Cl, ^{37}Cl, 11%]$^+$, 490.2 [M+H, ^{10}B, ^{37}Cl, ^{37}Cl, ^{37}Cl, 3%]$^+$, 491.2 [M+H, ^{11}B, ^{37}Cl, ^{37}Cl, ^{37}Cl, 1%]$^+$, 506.1 [M+Na, ^{10}B, ^{35}Cl, ^{35}Cl, ^{35}Cl, 38%]$^+$, 507.1 [M+Na, ^{11}B, ^{35}Cl, ^{35}Cl, ^{35}Cl, 100%]$^+$, 508.1 [M+Na, ^{10}B, ^{35}Cl, ^{35}Cl, ^{37}Cl, 43%]$^+$, 509.1 [M+Na, ^{11}B, ^{35}Cl, ^{35}Cl, ^{37}Cl, 78%]$^+$, 510.1 [M+Na, ^{10}B, ^{35}Cl, ^{37}Cl, ^{37}Cl, 19%]$^+$, 511.1 [M+Na, ^{11}B, ^{35}Cl, ^{37}Cl, ^{37}Cl, 26%]$^+$, 512.1 [M+Na, ^{10}B, ^{37}Cl, ^{37}Cl, ^{37}Cl, 3%]$^+$, 513.1 [M+Na, ^{11}B, ^{37}Cl, ^{37}Cl, ^{37}Cl, 2%]$^+$. HRMS(ES^+) (*m/z*): $[M+H]^+$ calcd. for $C_{24}H_{21}{}^{11}B^{35}Cl_3N_2O_2$, 485.0756; found, 485.0759, error: 0.6 ppm.

***N*-Benzylacetoacetamide (3c)**. Yield: 97%, cream solid. mp: 100-102 ^oC (lit.(Sridharan et al., 2010) 92-93 ^oC); ^1H NMR (300 MHz, $CDCl_3$): δ 7.24-7.40 (m, 5H, Aryl-H), 7.36 (br s, 1H, NH), 4.46 (d, *J* = 5.7 Hz, 2H, $NHC\underline{H}_2$) 3.45 (s, 2H, $CH_3COC\underline{H}_2$), 2.27 (s, 3H, CH_3CO); ^{13}C NMR (75 MHz, $CDCl_3$): δ 204.5 ($CH_3\underline{C}O$), 165.5 (CONH), 137.9 (Ph(*i*)), 128.7 (Ph(*m*)), 127.7 (Ph(*o*)), 127.5 (Ph(*p*)), 49.6 ($CH_3CO\underline{C}H_2$), 43.5 ($NH\underline{C}H_2Ph$), 31.0 ($\underline{C}H_3CO$); IR: 3245 (N-H), 1712 (C=O), 1638 (C=O), 1577 (C=O) cm^{-1}; MS(ES^-) (*m/z*): 190.1 [M-H, 5%]$^-$; 226.1 [M+^{35}Cl, 100%]$^-$, 228.1 [M+^{37}Cl, 33%]$^-$; MS(ES^+) (*m/z*): 192.1 [M+H, 100%]$^+$ 214.1 [M+Na, 63%]$^+$,

228.0 [M+Na, 68%]$^+$, 244.0 [M+K, 62%]$^+$. HRMS(APCI$^+$) (*m/z*): [M+H]$^+$ calcd. for $C_{11}H_{14}NO_2$, 192.1019; found, 192.1020, error: 0.5 ppm.

(*Z*)-2-Acetyl-3-amino-4,4,4-trichloro-*N*-benzylbut-2-enamide (4c). Yield: 75%, cream solid. mp: 92-93 ^oC (lit.(Veronese et al., 1986) 120-124 ^oC); ^1H NMR (300 MHz, $CDCl_3$): δ 8.61 (br s, 2H, NH_2), 7.22-7.31 (m, 5H, Aryl-H), 6.12 (br s, 1H, CONH) 4.45 (d, *J* = 5.4 Hz, 2H, NHC$\underline{H_2}$), 2.24 (s, 3H, CH_3CO); ^{13}C NMR (75 MHz, $CDCl_3$): δ 197.6 ($CH_3\underline{C}O$), 167.3 (C=$\underline{C}$(NH_2)CCl_3), 156.7 (CONH), 136.9 (Ph(*i*)), 128.8 (Ph(*m*)), 128.5 (Ph(*o*)), 127.9 (Ph(*p*)), 104.6 ($\underline{C}$=C(NH_2)CCl_3), 93.4 (CCl_3), 45.1 (NH$\underline{C}H_2$Ph), 28.5 ($\underline{C}H_3CO$); IR: 3297 (N-H), 1619 (C=O), 1541 (C=O), 1495 (C=C-NH_2) cm^{-1}; MS(ES$^-$) (*m/z*): 333.1 [M-H, ^{35}Cl, ^{35}Cl, ^{35}Cl, 43%]$^-$, 335.1 [M-H, ^{35}Cl, ^{35}Cl, ^{37}Cl, 38%]$^-$, 337.1 [M-H, ^{35}Cl, ^{37}Cl, ^{37}Cl, 11%]$^-$, 339.1 [M-H, ^{37}Cl, ^{37}Cl, ^{37}Cl, 2%]$^-$, 369.1 [M+^{35}Cl, ^{35}Cl, ^{35}Cl, ^{35}Cl, 89%]$^-$, 370.1 [M+^{37}Cl, ^{35}Cl, ^{35}Cl, ^{35}Cl, 5%]$^-$, 371.1 [M+^{35}Cl, ^{35}Cl, ^{35}Cl, ^{37}Cl, 100%]$^-$, 372.1 [M+^{37}Cl, ^{35}Cl, ^{35}Cl, ^{37}Cl, 15%]$^-$, 373.1 [M+^{35}Cl, ^{35}Cl, ^{37}Cl, ^{37}Cl, 47%]$^-$, 374.1 [M+^{37}Cl, ^{35}Cl, ^{37}Cl, ^{37}Cl, 3%]$^-$, 375.1 [M+^{35}Cl, ^{37}Cl, ^{37}Cl, ^{37}Cl, 6%]$^-$, 376.1 [M+^{37}Cl, ^{37}Cl, ^{37}Cl, ^{37}Cl, 1%]$^-$; MS(ES$^+$) (*m/z*): 335.1 [M+H, ^{35}Cl, ^{35}Cl, ^{35}Cl, 70%]$^+$, 337.1 [M+H, ^{35}Cl, ^{35}Cl, ^{37}Cl, 98%]$^+$, 339.1 [M+H, ^{35}Cl, ^{37}Cl, ^{37}Cl, 29%]$^+$, 341.1 [M+H, ^{37}Cl, ^{37}Cl, ^{37}Cl, 3%]$^+$, 357.1 [M+Na, ^{35}Cl, ^{35}Cl, ^{35}Cl, 30%]$^+$, 359.1 [M+Na, ^{35}Cl, ^{35}Cl, ^{37}Cl, 18%]$^+$, 361.1 [M+Na, ^{35}Cl, ^{37}Cl, ^{37}Cl, 4%]$^+$, 363.1 [M+Na, ^{37}Cl, ^{37}Cl, ^{37}Cl, 1%]$^+$. HRMS(APCI$^+$) (*m/z*): [M+Na]$^+$ calcd. for $C_{13}H_{13}{}^{35}Cl_3N_2O_2Na$, 356.9935; found, 356.9916, error: 5.3 ppm.

5-Acetyl-6-(benzylamino)-2,2-diphenyl-4-(trichloromethyl)-2,3-dihydro-1,3,2-oxazaborinin-1-ium-2-uide (NBC14). Ethyl acetate/*n*-hexane, 1:7. Yield: 12%, yellow solid. Exists as a mixture of rotamers in a 4:1 ratio. mp: 131-132 ^oC; ^1H NMR (300 MHz, $CDCl_3$): δ 7.85 (br s, 1H, C=C(NH)CCl_3), 7.00-7.45 (m, 15H, 3 x Aryl-H of major and minor rotamers), 5.87 (br s, 1H, CONH of minor rotamer), 5.86 (br s, 1H,

CONH of major rotamer), 4.77 (d, 2H, NHC*H*$_2$Ph of minor rotamer), 4.43 (d, *J* = 5.7 Hz, 2H, NHC*H*$_2$Ph of major rotamer), 2.36 (s, 3H, CH_3CO); ^{13}C NMR (75 MHz, $CDCl_3$): δ 187.2 (CH_3*C*O), 164.5 (C=*C*(NH)CCl_3), 162.2 (CONH), 136.7 (Ph(*i*)), 131.7 (B-Ph(*o*)), 128.8 (Ph(*m*)), 128.4 (Ph(*o*)), 127.9 (Ph(*p*)), 127.5 (B-Ph(*m*)), 127.0 (B-Ph(*p*)), 105.2 (*C*=C(NH)CCl_3), 92.8 (CCl_3), 44.8 (NH*C*H_2Ph), 23.4 (*C*H_3CO), B-Ph(*i*) quaternary signal not observed; IR: 3303 (N-H), 1631 (C=O), 1596 (C=C, conjugated), 1526 (C=C-NH) cm^{-1}; MS(ES$^-$) (*m/z*): 455.1 [M-Ac, ^{10}B, ^{35}Cl, ^{35}Cl, ^{35}Cl, 9%]$^-$, 456.2 [M-Ac, ^{11}B, ^{35}Cl, ^{35}Cl, ^{35}Cl, 74%]$^-$, 457.2 [M-Ac, ^{10}B, ^{35}Cl, ^{35}Cl, ^{37}Cl, 40%]$^-$, 458.2 [M-Ac, ^{11}B, ^{35}Cl, ^{35}Cl, ^{37}Cl, 100%]$^-$, 459.2 [M-Ac, ^{10}B, ^{35}Cl, ^{37}Cl, ^{37}Cl, 17%]$^-$, 460.2 [M-Ac, ^{11}B, ^{35}Cl, ^{37}Cl, ^{37}Cl, 18%]$^-$, 461.1 [M-Ac, ^{10}B, ^{37}Cl, ^{37}Cl, ^{37}Cl, 4%]$^-$, 462.1 [M-Ac, ^{11}B, ^{37}Cl, ^{37}Cl, ^{37}C, 3%]$^-$, 496.2 [M-H, ^{10}B, ^{35}Cl, ^{35}Cl, ^{35}Cl, 50%]$^-$, 497.2 [M-H, ^{11}B, ^{35}Cl, ^{35}Cl, ^{35}Cl, 73%]$^-$, 498.2 [M-H, ^{10}B, ^{35}Cl, ^{35}Cl, ^{37}Cl, 60%]$^-$, 499.2 [M-H, ^{11}B, ^{35}Cl, ^{35}Cl, ^{37}Cl, 73%]$^-$, 500.2 [M-H, ^{10}B, ^{35}Cl, ^{37}Cl, ^{37}Cl, 37%]$^-$, 501.2 [M-H, ^{11}B, ^{35}Cl, ^{37}Cl, ^{37}Cl, 18%]$^-$, 502.2 [M-H, ^{10}B, ^{37}Cl, ^{37}Cl, ^{37}Cl, 3%]$^-$, 503.2 [M-H, ^{11}B, ^{37}Cl, ^{37}Cl, ^{37}C, 2%]$^-$; MS(ES$^+$) (*m/z*): 498.1 [M+H, ^{10}B, ^{35}Cl, ^{35}Cl, ^{35}Cl, 13%]$^+$, 499.2 [M+H, ^{11}B, ^{35}Cl, ^{35}Cl, ^{35}Cl, 80%]$^+$, 500.2 [M+H, ^{10}B, ^{35}Cl, ^{35}Cl, ^{37}Cl, 35%]$^+$, 501.2 [M+H, ^{11}B, ^{35}Cl, ^{35}Cl, ^{37}Cl, 100%]$^+$, 502.2 [M+H, ^{10}B, ^{35}Cl, ^{37}Cl, ^{35}Cl, 26%]$^+$, 503.1 [M+H, ^{11}B, ^{35}Cl, ^{37}Cl, ^{37}Cl, 32%]$^+$, 504.1 [M+H, ^{10}B, ^{37}Cl, ^{37}Cl, ^{37}Cl, 5%]$^+$, 505.1 [M+H, ^{11}B, ^{37}Cl, ^{37}Cl, ^{37}Cl, 3%]$^+$, 520.2 [M+Na, ^{10}B, ^{35}Cl, ^{35}Cl, ^{35}Cl, 18%]$^+$, 521.2 [M+Na, ^{11}B, ^{35}Cl, ^{35}Cl, ^{35}Cl, 24%]$^+$, 522.2 [M+Na, ^{10}B, ^{35}Cl, ^{35}Cl, ^{37}Cl, 11%]$^+$, 523.1 [M+Na, ^{11}B, ^{35}Cl, ^{35}Cl, ^{37}Cl, 19%]$^+$, 524.1 [M+Na, ^{10}B, ^{35}Cl, ^{37}Cl, ^{37}Cl, 6%]$^+$, 525.1 [M+Na, ^{11}B, ^{35}Cl, ^{37}Cl, ^{37}Cl, 4%]$^+$, 526.1 [M+Na, ^{10}B, ^{37}Cl, ^{37}Cl, ^{37}Cl, 2%]$^+$, 527.1 [M+Na, ^{11}B, ^{37}Cl, ^{37}Cl, ^{37}Cl, 1%]$^+$. HRMS(APCI$^+$) (*m/z*): [M+Na]$^+$ calcd. for $C_{25}H_{22}{}^{11}B^{35}Cl_3N_2O_2Na$, 521.0732; found, 521.0720, error: 2.3 ppm.

***N*-Phenethylacetoacetamide (3d)**. A yellow solid formed on addition of 1N $HCl_{(aq)}$ and was discarded by filtration prior to Et_2O extraction. Yield: 71%, orange oil. ^{1}H NMR (300 MHz, $CDCl_3$): δ 7.08-7.27 (m, 5H, Aryl-H), 6.94 (br s, 1H, NH), 3.45 (q, *J* = 6.6 Hz, 2H, NHC<u>*H*</u>$_2$CH$_2$), 3.27 (s, 2H, CH$_3$COC<u>*H*</u>$_2$), 2.74 (t, *J* = 7.1 Hz, 2H, NHCH$_2$C<u>*H*</u>$_2$), 2.14 (s, 3H, CH$_3$CO); ^{13}C NMR (75 MHz, $CDCl_3$): 204.4 (CH$_3$<u>*C*</u>O), 165.4 (CONH), 138.7 (Ph(*i*)), 128.7 (Ph(*o*)), 128.6 (Ph(*m*)), 126.5 (Ph(*p*)), 49.8 (CH$_3$CO<u>*C*</u>H$_2$), 40.7 (NH<u>*C*</u>H$_2$CH$_2$), 35.6 (NHCH$_2$<u>*C*</u>H$_2$), 30.9 (<u>*C*</u>H$_3$CO); IR: 3303 (N-H), 1716 (C=O), 1644 (C=O), 1542 (C=O) cm^{-1}; MS(ES$^-$) (*m*/*z*): 204.0 [M-H, 100%]$^-$; MS(ES$^+$) (*m*/*z*): 164.1 [M-C_2H_3O, 47%]$^+$ 206.1 [M+H, 100%]$^+$, 228.0 [M+Na, 68%]$^+$, 244.0 [M+K, 62%]$^+$.

(*Z*)-2-Acetyl-3-amino-4,4,4-trichloro-*N*-phenethylbut-2-enamide (4d). Yield: 90%, brown solid. mp: 89-91 ^{o}C; ^{1}H NMR (300 MHz, $CDCl_3$): δ 8.58 (br s, 2H, NH$_2$), 7.05-7.31 (m, 5H, Aryl-H), 3.61 (q, *J* = 6.5 Hz, 2H, NHC<u>*H*</u>$_2$CH$_2$), 2.85 (t, *J* = 6.9 Hz, 2H, NHCH$_2$C<u>*H*</u>$_2$), 2.16 (s, 3H, CH$_3$CO); ^{13}C NMR (75 MHz, $CDCl_3$): δ 197.7 (CH$_3$<u>*C*</u>O), 167.7 (C=<u>*C*</u>(NH$_2$)CCl$_3$), 156.5 (CONH), 138.4 (Ph(*i*)), 128.8 (Ph(*o*)), 128.7 (Ph(*m*)), 126.8 (Ph(*p*)), 104.8 (<u>*C*</u>=C(NH$_2$)CCl$_3$), 93.3 (CCl$_3$), 41.4 (NH<u>*C*</u>H$_2$CH$_2$), 34.7 (NHCH$_2$<u>*C*</u>H$_2$), 28.3 (<u>*C*</u>H$_3$CO); IR: 3293 (N-H), 1621 (C=O), 1536 (C=O), 1496 (C=C-NH$_2$) cm^{-1}.

5-Acetyl-6-(phenethylamino)-2,2-diphenyl-4-(trichloromethyl)-2,3-dihydro-1,3,2-oxazaborinin-1-ium-2-uide (NBC15). Ethyl acetate/*n*-hexane, 1:4. Yield: 18%, yellow oil. Exists as a mixture of rotamers in a 7:3 ratio. ^{1}H NMR (300 MHz, $CDCl_3$): δ 7.71 (br s, 1H, C=C(NH)CCl$_3$), 7.01-7.31 (m, 15H, 3 x Aryl-H of major and minor rotamers), 5.38 (br s, 1H, CONH of major rotamer), 5.31 (br s, 1H, CONH of minor rotamer), 3.70 (q, 2H, NHC<u>*H*</u>$_2$CH$_2$ of minor rotamer), 3.47 (q, *J* = 6.5 Hz, 2H, NHC<u>*H*</u>$_2$CH$_2$ of major rotamer), 2.86 (t, 2H, NHCH$_2$C<u>*H*</u>$_2$ of minor rotamer), 2.72 (t, *J* =

6.9 Hz, 2H, NHCH$_2$C*H*$_2$ of major rotamer), 2.17 (s, 3H, CH$_3$CO); ^{13}C NMR (75 MHz, CDCl$_3$): δ 187.2 (CH$_3$*C*O), 164.8 (C=*C*(NH)CCl$_3$), 162.1 (CONH), 138.2 (Ph(*i*)), 131.7 (B-Ph(*o*)), 128.8 (Ph(*m*)), 128.6 (Ph(*o*)), 127.6 (B-Ph(*m*)), 127.2 (Ph(*m*)), 127.0 (B-Ph(*p*)), 105.6 (*C*=C(NH)CCl$_3$), 92.7 (CCl$_3$), 41.3 (NH*C*H$_2$CH$_2$), 34.7 (NHCH$_2$*C*H$_2$), 23.2 (*C*H$_3$CO), B-Ph(*i*) quaternary signal not observed; IR: 3341 (N-H), 1649 (C=O), 1594 (C=C, conjugated), 1519 (C=C-NH) cm^{-1}; MS(ES$^-$) (*m*/*z*): 510.1 [M-H, ^{10}B, ^{35}Cl, ^{35}Cl, ^{35}Cl, 6%]$^-$, 511.1 [M-H, ^{11}B, ^{35}Cl, ^{35}Cl, ^{35}Cl, 33%]$^-$, 512.2 [M-H, ^{10}B, ^{35}Cl, ^{35}Cl, ^{37}Cl, 13%]$^-$, 513.2 [M-H, ^{11}B, ^{35}Cl, ^{35}Cl, ^{37}Cl, 32%]$^-$, 514.1 [M-H, ^{10}B, ^{35}Cl, ^{37}Cl, ^{37}Cl, 7%]$^-$, 515.1 [M-H, ^{11}B, ^{35}Cl, ^{37}Cl, ^{37}Cl, 11%]$^-$, 516.1 [M-H, ^{10}B, ^{37}Cl, ^{37}Cl, ^{37}Cl, 2%]$^-$, 517.1 [M-H, ^{11}B, ^{37}Cl, ^{37}Cl, ^{37}C, 1%]$^-$; MS(ES$^+$) (*m*/*z*): 512.2 [M+H, ^{10}B, ^{35}Cl, ^{35}Cl, ^{35}Cl, 14%]$^+$, 513.1 [M+H, ^{11}B, ^{35}Cl, ^{35}Cl, ^{35}Cl, 68%]$^+$, 514.1 [M+H, ^{10}B, ^{35}Cl, ^{35}Cl, ^{37}Cl, 31%]$^+$, 515.1 [M+H, ^{11}B, ^{35}Cl, ^{35}Cl, ^{37}Cl, 74%]$^+$, 516.1 [M+H, ^{10}B, ^{35}Cl, ^{37}Cl, ^{35}Cl, 30%]$^+$, 517.1 [M+H, ^{11}B, ^{35}Cl, ^{37}Cl, ^{37}Cl, 22%]$^+$, 518.1 [M+H, ^{10}B, ^{37}Cl, ^{37}Cl, ^{37}Cl, 13%]$^+$, 519.1 [M+H, ^{11}B, ^{37}Cl, ^{37}Cl, ^{37}Cl, 11%]$^+$, 534.1 [M+Na, ^{10}B, ^{35}Cl, ^{35}Cl, ^{35}Cl, 15%]$^+$, 535.1 [M+Na, ^{11}B, ^{35}Cl, ^{35}Cl, ^{35}Cl, 41%]$^+$, 536.1 [M+Na, ^{10}B, ^{35}Cl, ^{35}Cl, ^{37}Cl, 10%]$^+$, 537.1 [M+Na, ^{11}B, ^{35}Cl, ^{35}Cl, ^{37}Cl, 33%]$^+$, 538.1 [M+Na, ^{10}B, ^{35}Cl, ^{37}Cl, ^{37}Cl, 14%]$^+$, 539.1 [M+Na, ^{11}B, ^{35}Cl, ^{37}Cl, ^{37}Cl, 26%]$^+$, 540.1 [M+Na, ^{10}B, ^{37}Cl, ^{37}Cl, ^{37}Cl, 3%]$^+$, 540.1 [M+Na, ^{11}B, ^{37}Cl, ^{37}Cl, ^{37}Cl, 2%]$^+$; HRMS(ES$^+$) (*m*/*z*): [M+H]$^+$ calcd. for $C_{26}H_{25}{}^{11}B^{35}Cl_3N_2O_2$, 513.1069; found, 513.1073, error: 0.8 ppm.

(*Z*)-3-Amino-4,4,4-trichloro-*N*-phenethylbut-2-enamide (4e). Ethyl acetate/*n*-hexane, 1:3. Yield: 49%, brown oil. ^{1}H NMR (300 MHz, CDCl$_3$): δ 7.08-7.33 (m, 5H, Aryl-H), 6.61 (br s, 2H, NH$_2$), 5.41 (br s, 1H, N*H*CH$_2$CH$_2$), 5.21 (s, 1H, *H*C=C(NH$_2$)CCl$_3$), 3.50 (q, *J* = 6.6 Hz, 2H, NHC*H*$_2$CH$_2$), 2.77 (t, *J* = 6.9 Hz, 2H, NHCH$_2$C*H*$_2$); ^{13}C NMR (75 MHz, CDCl$_3$): δ 168.4 (C=*C*(NH$_2$)CCl$_3$), 155.4 (CONH), 138.9 (Ph(*i*)), 128.8 (Ph(*m*)), 128.7 (Ph(*o*)), 126.5 (Ph(*p*)), 94.5 (CCl$_3$), 87.8

(<u>C</u>=C(NH_2)CCl_3), 40.4 (NH<u>C</u>$H_2$$CH_2$), 35.9 (NH$CH_2$<u>$C$</u>$H_2$); MS($ES^-$) (*m/z*): 306.0 [M-H, ^{35}Cl, ^{35}Cl, ^{35}Cl, 100%]$^-$, 308.0 [M-H, ^{35}Cl, ^{35}Cl, ^{37}Cl, 92%]$^-$, 310.0 [M-H, ^{35}Cl, ^{37}Cl, ^{37}Cl, 30%]$^-$, 312.0 [M-H, ^{37}Cl, ^{37}Cl, ^{37}Cl, 5%]$^-$; MS(ES^+) (*m/z*): 307.0 [M+H, ^{35}Cl, ^{35}Cl, ^{35}Cl, 100%]$^+$, 309.0 [M+H, ^{35}Cl, ^{35}Cl, ^{37}Cl, 95%]$^+$, 311.0 [M+H, ^{35}Cl, ^{37}Cl, ^{37}Cl, 26%]$^+$, 313.0 [M+H, ^{37}Cl, ^{37}Cl, ^{37}Cl, 2%]$^+$, 329.0 [M+Na, ^{35}Cl, ^{35}Cl, ^{35}Cl, 84%]$^+$, 331.0 [M+Na, ^{35}Cl, ^{35}Cl, ^{37}Cl, 69%]$^+$, 333.0 [M+Na, ^{35}Cl, ^{37}Cl, ^{37}Cl, 25%]$^+$, 335.0 [M+Na, ^{37}Cl, ^{37}Cl, ^{37}Cl, 4%]$^+$, 345.0 [M+K, ^{35}Cl, ^{35}Cl, ^{35}Cl, 11%]$^+$, 347.0 [M+K, ^{35}Cl, ^{35}Cl, ^{37}Cl, 13%]$^+$, 349.0 [M+K, ^{35}Cl, ^{37}Cl, ^{37}Cl, 10%]$^+$, 351.0 [M+K, ^{37}Cl, ^{37}Cl, ^{37}Cl, 3%]$^+$. HRMS(ES^+) (*m/z*): $[M+H]^+$ calcd. for $C_{12}H_{13}{}^{35}Cl_2N_2O$, 271.0405; found, 271.0404, error: 0.4 ppm.

6-(Phenethylamino)-2,2-diphenyl-4-(trichloromethyl)-2,3-dihydro-1,3,2-oxazaborinin-1-ium-2-uide (NBC16). Ethyl acetate/*n*-hexane, 1:4. Yield: 18%, yellow solid. mp: 106-108 °C, exists as a mixture of rotamers in ~ 3:1 ratio. ^{1}H NMR (300 MHz, $CDCl_3$): δ 7.05-7.41 (m, 15H, 3 x Aryl-H of major and minor rotamers), 6.36 (br s, 1H, C=C(NH)CCl_3 of minor rotamer), 5.95 (br s, 1H, C=C(NH)CCl_3 of major rotamer), 5.47 (br s, 1H, CONH of minor rotamer), 5.32 (br s, 1H, CONH of major rotamer), 5.14 (s, 1H, <u>*H*</u>C=C(NH)CCl_3 of minor rotamer), 5.06 (d, *J* = 1.8 Hz, 1H, <u>*H*</u>C=C(NH)CCl_3 of major rotamer), 3.67 (q, *J* = 6.6 Hz, 2H, NHC<u>H_2</u>CH_2 of major rotamer), 3.42 (q, 2H, NHC<u>H_2</u>CH_2 of minor rotamer), 2.83 (t, *J* = 6.8 Hz, 2H, NHCH_2C<u>H_2</u>); ^{13}C NMR (75 MHz, $CDCl_3$): δ 169.3 (CONH), 162.9 (C=<u>C</u>(NH)CCl_3), 137.8 (Ph(*i*)), 131.8 (B-Ph(*o*)), 128.9 (Ph(*m*)), 128.8 (Ph(*o*)), 127.3 (B-Ph(*m*)), 126.5 (Ph(*p*)), 126.3 (B-Ph(*p*)), 94.5 (CCl_3), 42.2 (NH<u>C</u>$H_2$$CH_2$), 35.8 (NH$CH_2$<u>$C$</u>$H_2$), B-Ph(*i*) quaternary signal and <u>C</u>=C(NH)CCl_3 signal not observed; IR: 3367 (N-H), 1580 (C=C, conjugated), 1509 (C=C-NH) cm^{-1}; MS(ES^-) (*m/z*): 468.1 [M-H, ^{10}B, ^{35}Cl, ^{35}Cl, ^{35}Cl, 14%]$^-$, 469.1 [M-H, ^{11}B, ^{35}Cl, ^{35}Cl, ^{35}Cl, 100%]$^-$, 470.1 [M-H, ^{10}B, ^{35}Cl, ^{35}Cl, ^{37}Cl, 48%]$^-$, 471.2 [M-H, ^{11}B, ^{35}Cl, ^{35}Cl, ^{37}Cl, 77%]$^-$, 472.1 [M-H, ^{10}B, ^{35}Cl, ^{37}Cl, ^{37}Cl, 31%]$^-$,

473.2 [M-H, ^{11}B, ^{35}Cl, ^{37}Cl, ^{37}Cl, 24%]$^-$, 474.0 [M-H, ^{10}B, ^{37}Cl, ^{37}Cl, ^{37}Cl, 5%]$^-$, 475.2 [M-H, ^{11}B, ^{37}Cl, ^{37}Cl, ^{37}C, 3%]$^-$; MS(ES$^+$) (*m/z*): 392.1 [M-Ph, ^{10}B, ^{35}Cl, ^{35}Cl, ^{35}Cl, 13%]$^+$, 393.1 [M-Ph, ^{11}B, ^{35}Cl, ^{35}Cl, ^{35}Cl, 40%]$^+$, 394.1 [M-Ph, ^{10}B, ^{35}Cl, ^{35}Cl, ^{37}Cl, 30%]$^+$, 395.0 [M-Ph, ^{11}B, ^{35}Cl, ^{35}Cl, ^{37}Cl, 59%]$^+$, 396.1 [M-Ph, ^{10}B, ^{35}Cl, ^{37}Cl, ^{37}Cl, 12%]$^+$, 397.1 [M-Ph, ^{11}B, ^{35}Cl, ^{37}Cl, ^{37}Cl, 15%]$^+$, 398.1 [M-Ph, ^{10}B, ^{37}Cl, ^{37}Cl, ^{37}Cl, 5%]$^+$, 399.1 [M-Ph, ^{11}B, ^{37}Cl, ^{37}Cl, ^{37}Cl, 11%]$^+$, 470.1 [M+H, ^{10}B, ^{35}Cl, ^{35}Cl, ^{35}Cl, 22%]$^+$, 471.1 [M+H, ^{11}B, ^{35}Cl, ^{35}Cl, ^{35}Cl, 100%]$^+$, 472.1 [M+H, ^{10}B, ^{35}Cl, ^{35}Cl, ^{37}Cl, 36%]$^+$, 473.1 [M+H, ^{11}B, ^{35}Cl, ^{35}Cl, ^{37}Cl, 98%]$^+$, 474.1 [M+H, ^{10}B, ^{35}Cl, ^{37}Cl, ^{35}Cl, 37%]$^+$, 475.2 [M+H, ^{11}B, ^{35}Cl, ^{37}Cl, ^{37}Cl, 28%]$^+$, 476.2 [M+H, ^{10}B, ^{37}Cl, ^{37}Cl, ^{37}Cl, 12%]$^+$, 477.2 [M+H, ^{11}B, ^{37}Cl, ^{37}Cl, ^{37}Cl, 4%]$^+$, 492.1 [M+Na, ^{10}B, ^{35}Cl, ^{35}Cl, ^{35}Cl, 3%]$^+$, 493.1 [M+Na, ^{11}B, ^{35}Cl, ^{35}Cl, ^{35}Cl, 22%]$^+$, 494.1 [M+Na, ^{10}B, ^{35}Cl, ^{35}Cl, ^{37}Cl, 4%]$^+$, 495.2 [M+Na, ^{11}B, ^{35}Cl, ^{35}Cl, ^{37}Cl, 10%]$^+$, 496.2 [M+Na, ^{10}B, ^{35}Cl, ^{37}Cl, ^{37}Cl, 7%]$^+$, 497.2 [M+Na, ^{11}B, ^{35}Cl, ^{37}Cl, ^{37}Cl, 5%]$^+$, 498.2 [M+Na, ^{10}B, ^{37}Cl, ^{37}Cl, ^{37}Cl, 3%]$^+$, 499.2 [M+Na, ^{11}B, ^{37}Cl, ^{37}Cl, ^{37}Cl, 2%]$^+$, 508.1 [M+K, ^{10}B, ^{35}Cl, ^{35}Cl, ^{35}Cl, 3%]$^+$, 509.1 [M+K, ^{11}B, ^{35}Cl, ^{35}Cl, ^{35}Cl, 20%]$^+$, 510.1 [M+K, ^{10}B, ^{35}Cl, ^{35}Cl, ^{37}Cl, 4%]$^+$, 511.2 [M+K, ^{11}B, ^{35}Cl, ^{35}Cl, ^{37}Cl, 13%]$^+$, 512.2 [M+K, ^{10}B, ^{35}Cl, ^{37}Cl, ^{37}Cl, 5%]$^+$, 513.2 [M+K, ^{11}B, ^{35}Cl, ^{37}Cl, ^{37}Cl, 8%]$^+$, 514.2 [M+K, ^{10}B, ^{37}Cl, ^{37}Cl, ^{37}Cl, 3%]$^+$, 515.2 [M+K, ^{11}B, ^{37}Cl, ^{37}Cl, ^{37}Cl, 4%]$^+$. HRMS(ES$^-$) (*m/z*): [M-H]$^-$ calcd. for $C_{24}H_{21}{}^{11}B^{35}Cl_3N_2O$, 469.0818; found, 469.0830, error: 2.6 ppm.

***N*-Adamantylacetoacetamide (3f)**. Yield: 55%, orange solid. mp: 81-82 ^{o}C {lit.(Clemens and Hyatt, 1985) 82-83 ^{o}C); ^{1}H NMR (300 MHz, $CDCl_3$): δ 6.50 (br s, 1H, NH), 3.32 (s, 2H, $CH_3COC\underline{H}_2$), 2.26 (s, 3H, CH_3CO), 2.07 (m, 3H, Adamantyl-H_2), 2.01 (m, 6H, Adamantyl-H_3 and Adamantyl-H_2), 1.68 (m, 6H, Adamantyl-H_1); ^{13}C NMR (75 MHz, $CDCl_3$): δ 204.9 ($CH_3\underline{C}O$), 164.2 (CONH), 52.1 (Adamantyl-C_4), 51.2 ($CH_3CO\underline{C}H_2$), 41.5 (Adamantyl-C_3), 36.3 (Adamantyl-C_2), 30.9 ($\underline{C}H_3CO$), 29.4

(Adamantyl-C_1); IR: 3310 (N-H), 2903 (Adamantyl CH_2 stretch), 2849 (Adamantyl CH_2 stretch), 1721 (C=O), 1639 (C=O), 1545 (C=O) cm^{-1}; MS(ES^-) (*m/z*): 270.1 [M+Cl, ^{35}Cl, 100%]$^-$, 272.2 [M+Cl, ^{37}Cl, 48%]$^-$; MS(ES^+) (*m/z*): 236.1 [M+H, 100%]$^+$, 471.4 [2M+H, 93%]$^+$, 493.4 [2M+Na, 49%]$^+$. HRMS($APCI^+$) (*m/z*): [M+H]$^+$ calcd. for $C_{14}H_{22}NO_2$, 236.1645; found, 236.1633, error: 5.1 ppm.

(*Z*)-2-Acetyl-3-amino-4,4,4-trichloro-*N*-adamantylbut-2-enamide (4f). Yield: 41%, white solid. mp: 105-106 ^oC; ^1H NMR (300 MHz, $CDCl_3$): δ 8.55 (br s, 2H, NH_2), 5.54 (br s, 1H, CONH) 2.29 (s, 3H, CH_3CO), 2.02 (m, 10H, Adamantyl-H), 1.91 (d, *J* = 2.4 Hz, 1H, Adamantyl-H), 1.64 (m, 7H, Adamantyl-H); ^{13}C NMR (75 MHz, $CDCl_3$): δ 198.2 ($CH_3\underline{C}O$), 166.1 (C=$\underline{C}$(NH_2)CCl_3), 155.9 (CONH), 106.6 ($\underline{C}$=C(NH_2)CCl_3), 93.7 (CCl_3), 52.7 (Adamantyl-C_4), 41.1 (Adamantyl-C_3), 36.3 (Adamantyl-C_2), 29.4 (Adamantyl-C_1), 28.4 ($\underline{C}H_3CO$); IR: 3309 (N-H), 2903 (Adamantyl CH_2 stretch), 2849 (Adamantyl CH_2 stretch), 1633 (C=O), 1617 (C=C, conjugated), 1517 (C=C-NH_2) cm^{-1}; MS(ES^-) (*m/z*): 377.1 [M-H, ^{35}Cl, ^{35}Cl, ^{35}Cl, 12%]$^-$, 379.1 [M-H, ^{35}Cl, ^{35}Cl, ^{37}Cl, 9%]$^-$, 381.1 [M-H, ^{35}Cl, ^{37}Cl, ^{37}Cl, 4%]$^-$, 383.1 [M-H, ^{37}Cl, ^{37}Cl, ^{37}Cl, 1%]$^-$, 413.1 [M+^{35}Cl, ^{35}Cl, ^{35}Cl, ^{35}Cl, 91%]$^-$, 414.1 [M+^{37}Cl, ^{35}Cl, ^{35}Cl, ^{35}Cl, 8%]$^-$, 415.1 [M+^{35}Cl, ^{35}Cl, ^{35}Cl, ^{37}Cl, 100%]$^-$, 416.1 [M+^{37}Cl, ^{35}Cl, ^{35}Cl, ^{37}Cl, 15%]$^-$, 417.1 [M+^{35}Cl, ^{35}Cl, ^{37}Cl, ^{37}Cl, 44%]$^-$, 418.1 [M+^{37}Cl, ^{35}Cl, ^{37}Cl, ^{37}Cl, 13%]$^-$, 419.1 [M+^{35}Cl, ^{37}Cl, ^{37}Cl, ^{37}Cl, 7%]$^-$, 420.2 [M+^{37}Cl, ^{37}Cl, ^{37}Cl, ^{37}Cl, 3%]$^-$; MS(ES^+) (*m/z*): 379.1 [M+H, ^{35}Cl, ^{35}Cl, ^{35}Cl, 100%]$^+$, 381.1 [M+H, ^{35}Cl, ^{35}Cl, ^{37}Cl, 45%]$^+$, 383.1 [M+H, ^{35}Cl, ^{37}Cl, ^{37}Cl, 13%]$^+$, 385.1 [M+H, ^{37}Cl, ^{37}Cl, ^{37}Cl, 3%]$^+$, 401.1 [M+Na, ^{35}Cl, ^{35}Cl, ^{35}Cl, 81%]$^+$, 403.1 [M+Na, ^{35}Cl, ^{35}Cl, ^{37}Cl, 84%]$^+$, 405.1 [M+Na, ^{35}Cl, ^{37}Cl, ^{37}Cl, 19%]$^+$, 407.1 [M+Na, ^{37}Cl, ^{37}Cl, ^{37}Cl, 4%]$^+$. HRMS($APCI^+$) (*m/z*): [M+H]$^+$ calcd. for $C_{16}H_{22}{}^{35}Cl_3N_2O_2$, 379.0741; found, 379.0738, error: 0.8 ppm.

5-Acetyl-6-(adamantan-1-ylamino)-2,2-diphenyl-4-(trichloromethyl)-2,3-dihydro-1,3,2-oxazaborinin-1-ium-2-uide (NBC17). Ethyl acetate/*n*-hexane, 1:19. Yield: 33%, yellow solid. mp: 115-116 ^{o}C; ^{1}H NMR (300 MHz, $CDCl_3$): δ 7.73 (br s, 1H, C=C(NH)CCl_3), 7.12-7.33 (m, 10H, Aryl-H), 5.08 (br s, 1H, CONH), 2.29 (s, 3H, CH_3CO), 1.99 (m, 3H, Adamantyl-H), 1.87 (m, 6H, Adamantyl-H), 1.59 (m, 6H, Adamantyl-H); ^{13}C NMR (75 MHz, $CDCl_3$): δ 186.9 (CH_3<u>*C*</u>O), 163.4 (C=<u>*C*</u>(NH)CCl_3), 162.0 (CONH), 131.7 (B-Ph(*o*)), 127.5 (B-Ph(*m*)), 127.0 (B-Ph(*p*)), 107.1 (<u>*C*</u>=C(NH)CCl_3), 93.0 (CCl_3), 52.9 (Adamantyl-C_4), 41.0 (Adamantyl-C_3), 36.2 (Adamantyl-C_2), 29.3 (Adamantyl-C_1), 23.2 (<u>*C*</u>H_3CO), B-Ph(*i*) quaternary signal not observed; IR: 3318 (N-H), 2909 (Adamantyl CH_2 stretch), 2851 (Adamantyl CH_2 stretch), 1638 (C=O), 1603 (C=C, conjugated), 1526 (C=C-NH) cm^{-1}; MS(ES^{-}) (*m/z*): 540.2 [M-H, ^{10}B, ^{35}Cl, ^{35}Cl, ^{35}Cl, 10%]$^{-}$, 541.2 [M-H, ^{11}B, ^{35}Cl, ^{35}Cl, ^{35}Cl, 100%]$^{-}$, 542.2 [M-H, ^{10}B, ^{35}Cl, ^{35}Cl, ^{37}Cl, 45%]$^{-}$, 543.2 [M-H, ^{11}B, ^{35}Cl, ^{35}Cl, ^{37}Cl, 64%]$^{-}$, 544.2 [M-H, ^{10}B, ^{35}Cl, ^{37}Cl, ^{37}Cl, 23%]$^{-}$, 545.2 [M-H, ^{11}B, ^{35}Cl, ^{37}Cl, ^{37}Cl, 33%]$^{-}$, 546.2 [M-H, ^{10}B, ^{37}Cl, ^{37}Cl, ^{37}Cl, 3%]$^{-}$, 547.2 [M-H, ^{11}B, ^{37}Cl, ^{37}Cl, ^{37}C, 2%]$^{-}$; MS(ES^{+}) (*m/z*): 542.2 [M+H, ^{10}B, ^{35}Cl, ^{35}Cl, ^{35}Cl, 22%]$^{+}$, 543.2 [M+H, ^{11}B, ^{35}Cl, ^{35}Cl, ^{35}Cl, 100%]$^{+}$, 544.2 [M+H, ^{10}B, ^{35}Cl, ^{35}Cl, ^{37}Cl, 39%]$^{+}$, 545.2 [M+H, ^{11}B, ^{35}Cl, ^{35}Cl, ^{37}Cl, 70%]$^{+}$, 546.2 [M+H, ^{10}B, ^{35}Cl, ^{37}Cl, ^{35}Cl, 35%]$^{+}$, 547.2 [M+H, ^{11}B, ^{35}Cl, ^{37}Cl, ^{37}Cl, 24%]$^{+}$, 548.2 [M+H, ^{10}B, ^{37}Cl, ^{37}Cl, ^{37}Cl, 11%]$^{+}$, 549.2 [M+H, ^{11}B, ^{37}Cl, ^{37}Cl, ^{37}Cl, 2%]$^{+}$, 534.1 [M+Na, ^{10}B, ^{35}Cl, ^{35}Cl, ^{35}Cl, 15%]$^{+}$, 535.1 [M+Na, ^{11}B, ^{35}Cl, ^{35}Cl, ^{35}Cl, 41%]$^{+}$, 536.1 [M+Na, ^{10}B, ^{35}Cl, ^{35}Cl, ^{37}Cl, 10%]$^{+}$, 537.1 [M+Na, ^{11}B, ^{35}Cl, ^{35}Cl, ^{37}Cl, 33%]$^{+}$, 538.1 [M+Na, ^{10}B, ^{35}Cl, ^{37}Cl, ^{37}Cl, 14%]$^{+}$, 539.1 [M+Na, ^{11}B, ^{35}Cl, ^{37}Cl, ^{37}Cl, 26%]$^{+}$, 540.1 [M+Na, ^{10}B, ^{37}Cl, ^{37}Cl, ^{37}Cl, 3%]$^{+}$, 540.1 [M+Na, ^{11}B, ^{37}Cl, ^{37}Cl, ^{37}Cl, 2%]$^{+}$. HRMS(ES^{+}) (*m/z*): [M+Na]$^{+}$ calcd. for $C_{28}H_{30}{}^{11}B^{35}Cl_3N_2O_2Na$, 565.1358; found, 565.1359, error: 0.2 ppm.

***N*-Cyclohexylacetoacetamide (3g)**. Yield: 78%, cream solid. mp: 75-76°C (lit.(Sridharan et al., 2010) 76-77 ^{o}C); ^{1}H NMR (300 MHz, $CDCl_3$): δ 7.08 (br s, 1H, NH), 3.70 (p, *J* = 3.6 Hz, 1H, -C*H*$CH_2CH_2CH_2CH_2CH_2$-), 3.36 (s, 2H, CH_3COC*H_2*), 2.20 (s, 3H, CH_3CO); 1.82 (d, *J* = 11.7 Hz, 2H, -CHC*H_2*$(CH_2)_3$C*H_2*-), 1.04-1.70 (m, 8H, -CHC*H_2*(C*H_2*)$_3$C*H_2*-); ^{13}C NMR (75 MHz, $CDCl_3$): δ 204.8 (CH_3*C*O), 164.3 (CONH), 49.9 (CH_3CO*C*H_2), 48.2 (-*C*H$(CH_2)_2(CH_2)_2CH_2$-), 32.8 (-CH(*C*H_2)$_2(CH_2)_2CH_2$-), 31.0 (*C*H_3CO), 25.5 (-CH$(CH_2)_2(CH_2)_2$*C*H_2), 24.7 (-CH$(CH_2)_2$(*C*H_2)$_2CH_2$-); IR: 3290 (N-H), 1716 (C=O), 1638 (C=O), 1543 (C=O) cm^{-1}; MS(ES^{+}) (*m*/*z*): 184.1 [M+H, 100%]$^{+}$, 206.1 [M+Na, 82%]$^{+}$. HRMS(ES^{+}) (*m*/*z*): [M+H]$^{+}$ calcd. for $C_{10}H_{18}NO_2$, 184.1332; found, 184.1329, error: 1.6 ppm.

(*Z*)-2-Acetyl-3-amino-4,4,4-trichloro-*N*-cyclohexylbut-2-enamide (4g). Yield: 93%, cream solid. mp: 99-101 ^{o}C (dec); ^{1}H NMR (300 MHz, $CDCl_3$): δ 8.66 (br s, 1H, NH_2), 5.83 (d, *J* = 7.5 Hz, 1H, NH), 3.81-3.97 (m, *J* = 3.6 Hz, 1H, -C*H*$CH_2CH_2CH_2CH_2CH_2$-), 2.32 (s, 3H, CH_3CO); 1.95-2.08 (m, 2H, -CHC*H_2*$(CH_2)_3$C*H_2*-), 1.53-1.79 (m, 4H, -CHC*H_2*(C*H_2*)$_3$C*H_2*-), 1.32-1.49 (m, 2H, -$CHCH_2$(C*H_2*)$_3CH_2$-), 1.11-1.27 (m, 3H, -$CHCH_2$(C*H_2*)$_3CH_2$-); ^{13}C NMR (75 MHz, $CDCl_3$): δ 197.8 (CH_3*C*O), 166.5 (C=*C*$(NH_2)CCl_3$), 156.3 (CONH), 105.3 (*C*=C$(NH_2)CCl_3$), 93.5 (CCl_3), 49.0 (-*C*H$(CH_2)_2(CH_2)_2CH_2$-), 32.4 (-CH(*C*H_2)$_2(CH_2)_2CH_2$-), 28.4 (*C*H_3CO), 25.5 (-CH$(CH_2)_2(CH_2)_2$*C*H_2-), 24.7 (-CH$(CH_2)_2$(*C*H_2)$_2CH_2$-); IR: 3271 (N-H), 2929 (N-H), 1721 (C=O), 1620 (C=O), 1535 (C=C-NH_2) cm^{-1}; MS(ES^{-}) (*m*/*z*): 325.0 [M-H, ^{35}Cl, ^{35}Cl, ^{35}Cl, 100%]$^{-}$, 327.0 [M-H, ^{35}Cl, ^{35}Cl, ^{37}Cl, 53%]$^{-}$, 329.0 [M-H, ^{35}Cl, ^{37}Cl, ^{37}Cl, 33%]$^{-}$, 331.0 [M-H, ^{37}Cl, ^{37}Cl, ^{37}Cl, 3%]$^{-}$, 361.0 [M+^{35}Cl, ^{35}Cl, ^{35}Cl, ^{35}Cl, 87%]$^{-}$, 363.0 [M+^{35}Cl, ^{35}Cl, ^{35}Cl, ^{37}Cl, 48%]$^{-}$, 365.0 [M+^{35}Cl, ^{35}Cl, ^{37}Cl, ^{37}Cl, 24%]$^{-}$, 367.0 [M+^{35}Cl, ^{37}Cl, ^{37}Cl, ^{37}Cl, 5%]$^{-}$; MS(ES^{+}) (*m*/*z*): 327.0 [M+H, ^{35}Cl, ^{35}Cl, ^{35}Cl, 62%]$^{+}$, 329.0 [M+H, ^{35}Cl, ^{35}Cl, ^{37}Cl, 41%]$^{+}$, 331.0

[M+H, ^{35}Cl, ^{37}Cl, ^{37}Cl, 15%]$^+$, 333.0 [M+H, ^{37}Cl, ^{37}Cl, ^{37}Cl, 2%]$^+$; HRMS(ES$^+$) (*m*/*z*): [M+H]$^+$ calcd. for $C_{12}H_{18}{}^{35}Cl_3N_2O_2$, 327.0428; found, 327.0426, error: 0.6 ppm.

5-Acetyl-6-(cyclohexylamino)-2,2-diphenyl-4-(trichloromethyl)-2,3-dihydro-1,3,2-oxazaborinin-1-ium-2-uide (NBC19, 17). Ethyl acetate/*n*-hexane, 1:4. Yield: 9%, orange solid. mp: 53-54°C; ^{1}H NMR (300 MHz, $CDCl_3$): δ 7.82 (br s, 1H, C=C(NH)CCl_3), 7.27-7.39 (m, 10H, 2 x Aryl-H), 5.38 (d, *J* = 7.5 Hz, 1H, CONH), 3.69-3.85 (m, 1H, -C$\underline{H}$(CH_2)$_2$(CH_2)$_2$$CH_2$-), 2.35 (s, 3H, CH_3CO); 1.89 (d, *J* = 10.5 Hz, 2H, -CHC$\underline{H}_2$(CH_2)$_3$C$\underline{H}_2$-), 0.98-1.42 (m, 8H, -CHC$\underline{H}_2$(C$\underline{H}_2$)$_3$C$\underline{H}_2$-); ^{13}C NMR (75 MHz, $CDCl_3$): δ 187.2 ($CH_3$$\underline{C}$O), 163.8 (C=$\underline{C}(NH)CCl_3$), 162.3 (CONH), 156.3 (B-Ph(*i*)), 131.9 (B-Ph(*o*)), 127.7 (B-Ph(*m*)), 127.2 (B-Ph(*p*)), 105.9 ($\underline{C}$=C(NH)CCl_3), 93.1 (CCl_3), 49.3 (-$\underline{C}$H(CH_2)$_2$(CH_2)$_2$$CH_2$-), 32.6 (-CH($\underline{C}H_2$)$_2$($CH_2$)$_2$$CH_2$-), 25.5 (-CH($CH_2$)$_2$($CH_2$)$_2$$\underline{C}H_2$-), 24.8 (-CH($CH_2$)$_2$($\underline{C}H_2$)$_2$$CH_2$-), 23.4 ($\underline{C}H_3$CO); IR: 3311 (N-H), 2928 (N-H), 1631 (C=O), 1594 (C=O), 1514 (C=C-NH) cm^{-1}; MS(ES$^-$) (*m*/*z*): 447.1 [M-C_2H_3O, ^{10}B, ^{35}Cl, ^{35}Cl, ^{35}Cl, 20%]$^-$, 448.1 [M-C_2H_3O, ^{35}Cl, ^{35}Cl, ^{35}Cl, 99%]$^-$, 449.1 [M-C_2H_3O, ^{10}B, ^{35}Cl, ^{35}Cl, ^{37}Cl, 33%]$^-$, 450.1 [M-C_2H_3O, ^{11}B, ^{35}Cl, ^{35}Cl, ^{37}Cl, 100%]$^-$, 451.1 [M-C_2H_3O, ^{10}B, ^{35}Cl, ^{37}Cl, ^{37}Cl, 25%]$^-$, 452.1 [M-C_2H_3O, ^{11}B, ^{35}Cl, ^{37}Cl, ^{35}Cl, 23%]$^-$, 453.1 [M-C_2H_3O, ^{10}B, ^{37}Cl, ^{37}Cl, ^{37}Cl, 8%]$^-$, 454.1 [M-C_2H_3O, ^{11}B, ^{37}Cl, ^{37}Cl, ^{37}Cl, 3%]$^-$, 488.1 [M-H, ^{10}B, ^{35}Cl, ^{35}Cl, ^{35}Cl, 32%]$^-$, 489.1 [M-H, ^{11}B, ^{35}Cl, ^{35}Cl, ^{35}Cl, 100%]$^-$, 490.1 [M-H, ^{10}B, ^{35}Cl, ^{35}Cl, ^{37}Cl, 48%]$^-$, 491.2 [M-H, ^{11}B, ^{35}Cl, ^{35}Cl, ^{37}Cl, 94%]$^-$, 492.2 [M-H, ^{10}B, ^{35}Cl, ^{37}Cl, ^{35}Cl, 22%]$^-$, 493.2 [M-H, ^{11}B, ^{35}Cl, ^{37}Cl, ^{37}Cl, 32%]$^-$, 494.2 [M-H, ^{10}B, ^{37}Cl, ^{37}Cl, ^{37}Cl, 5%]$^-$, 495.2 [M-H, ^{11}B, ^{37}Cl, ^{37}Cl, ^{37}Cl, 4%]$^-$. MS(ES$^+$) (*m*/*z*): 490.2 [M+H, ^{10}B, ^{35}Cl, ^{35}Cl, ^{35}Cl, 13%]$^+$, 491.2 [M+H, ^{11}B, ^{35}Cl, ^{35}Cl, ^{35}Cl, 100%]$^+$, 492.2 [M+H, ^{10}B, ^{35}Cl, ^{35}Cl, ^{37}Cl, 45%]$^+$, 493.2 [M+H, ^{11}B, ^{35}Cl, ^{35}Cl, ^{37}Cl, 46%]$^+$, 494.2 [M+H, ^{10}B, ^{35}Cl, ^{37}Cl, ^{37}Cl, 18%]$^+$, 495.2 [M+H, ^{11}B, ^{35}Cl, ^{37}Cl, ^{37}Cl, 24%]$^+$, 496.2 [M+H, ^{10}B, ^{37}Cl, ^{37}Cl, ^{37}Cl, 6%]$^+$, 497.2 [M+H, ^{11}B, ^{37}Cl, ^{37}Cl, ^{37}Cl, 3%]$^+$.

HRMS(ES$^+$) (*m/z*): $[M+H]^+$ calcd. for $C_{24}H_{27}{}^{11}B^{35}Cl_3N_2O_2$, 491.1226; found, 491.1227, error: 0.2 ppm.

***N*-(Pyren-1-ylmethyl)acetoacetamide (3h)**. *tert*-Butyl acetoacetate (0.14 ml, 0.82 mmol) was added to a solution of 1-pyrenemethylamine hydrochloride (0.20 g, 0.75 mmol) and triethylamine (0.21 ml, 1.49 mmol) in toluene (50 ml). The procedure was carried out as described previously. Yield: 0.23 g (98%), orange solid. mp: 130-132 ^oC; ^1H NMR (300 MHz, $CDCl_3$): δ 7.88-8.21 (m, 9H, pyrene-H), 7.27 (br s, 1H, NH), 5.12 (d, *J* = 5.4 Hz, 2H, $C\underline{H_2}NH$) 3.41 (s, 2H, $CH_3COC\underline{H_2}$), 2.17 (s, 3H, CH_3CO); ^{13}C NMR (75 MHz, $CDCl_3$): δ 204.4 ($CH_3\underline{C}O$), 165.1 (CONH), 131.3, 130.8, 129.03, 128.96, 128.3, 128.2, 127.5, 127.4, 127.0, 126.1, 125.4, 125.3, 125.0, 124.8, 124.7, 122.7, 49.5 ($CH_3CO\underline{C}H_2$), 41.9 (CH_2NH) 31.0 ($\underline{C}H_3CO$); IR: 3284 (N-H), 1714 (C=O), 1622 (C=O), 1536 (C=O) cm^{-1}; MS(ES$^-$) (*m/z*): 314.3 $[M-H, 15\%]^-$, 350.1 [M+Cl, ^{35}Cl, 30%]$^-$, 352.1 [M+Cl, ^{37}Cl, 12%]$^-$;MS(ES$^+$) (*m/z*): 316.1 $[M+H, 84\%]^+$, 338.3 [M+Na, 23%]$^+$. HRMS(ES$^-$) (*m/z*): $[M-H]^-$ calcd. for $C_{21}H_{16}NO_2$, 314.1187; found, 314.1185, error: 0.6 ppm.

(*Z*)-2-Acetyl-3-amino-4,4,4-trichloro-*N*-(pyren-1-ylmethyl)but-2-enamide (4h). Yield: 76%, orange solid. mp: 124-126 ^oC (dec); ^1H NMR (300 MHz, $CDCl_3$): δ 8.29 (d, *J* = 9.0 Hz, 1H, pyrene-C8-H), 7.92-8.19 (m, 8H, pyrene-H), 6.21 (br s, 1H, $CON\underline{H}CH_2$), 5.18 (d, *J* = 5.1 Hz, 2H, $CONHC\underline{H_2}$), 2.28 (s, 3H, CH_3CO); ^{13}C NMR (75 MHz, $CDCl_3$): δ 197.6 ($CH_3\underline{C}O$), 167.2 ($C{=}\underline{C}(NH_2)CCl_3$), 156.7 (CONH), 131.5, 131.3, 130.7, 129.9, 128.4, 127.84, 127.77, 127.3, 126.2, 125.6, 125.5, 124.9, 122.9, 104.5 ($\underline{C}{=}C(NH_2)CCl_3$), 93.4 ($CCl_3$), 43.2 ($CH_3CO\underline{C}H_2$), 28.6 ($\underline{C}H_3CO$), $CONHCH_2\underline{C}$ quaternary signal not observed; IR: 3248 (N-H), 1717 (C=O), 1620 (C=O), 1520 ($C{=}C{-}NH_2$) cm^{-1}; MS(ES$^-$) (*m/z*): 416.0 [$M-C_2H_3O$, ^{35}Cl, ^{35}Cl, ^{35}Cl, 50%]$^-$, 418.1 [$M-C_2H_3O$, ^{35}Cl, ^{35}Cl, ^{37}Cl, 23%]$^-$, 420.1 [$M-C_2H_3O$, ^{35}Cl, ^{37}Cl, ^{37}Cl, 20%]$^-$, 421.1 [M-

C_2H_3O, ^{37}Cl, ^{37}Cl, ^{37}Cl, 8%]$^-$, 457.1 [M-H, ^{35}Cl, ^{35}Cl, ^{35}Cl, 65%]$^-$, 459.0 [M-H, ^{35}Cl, ^{35}Cl, ^{37}Cl, 100%]$^-$, 461.1 [M-H, ^{35}Cl, ^{37}Cl, ^{37}Cl, 12%]$^-$, 463.1 [M-H, ^{37}Cl, ^{37}Cl, ^{37}Cl, 3%]$^-$, 493.0 [M+^{35}Cl, ^{35}Cl, ^{35}Cl, ^{35}Cl, 34%]$^-$, 495.0 [M+^{35}Cl, ^{35}Cl, ^{35}Cl, ^{37}Cl, 35%]$^-$, 497.0 [M+^{35}Cl, ^{35}Cl, ^{37}Cl, ^{37}Cl, 26%]$^-$, 499.0 [M+^{35}Cl, ^{37}Cl, ^{37}Cl, ^{37}Cl, 4%]$^-$. HRMS(ES$^-$) (*m/z*): [M-H]$^-$ calcd. for $C_{23}H_{16}{}^{35}Cl_3N_2O_2$, 457.0283; found, 457.0277, error: 1.3 ppm.

5-Acetyl-2,2-diphenyl-6-((pyren-1-ylmethyl)amino)-4-(trichloromethyl)-2,3-dihydro-1,3,2-oxazaborinin-1-ium-2-uide (NBC20). Ethyl acetate/*n*-hexane, 1:4. Yield: 12%, yellow solid. mp: 167-169 ^oC (dec); ^1H NMR (300 MHz, $CDCl_3$): δ 8.03-8.30 (m, 8H, pyrene-H), 7.93 (d, *J* = 7.8 Hz, 1H, pyrene-C3-H), 7.80 (br s, 1H, C=C(NH)CCl_3), 7.13-7.31 (m, 10H, Aryl-H), 5.89 (t, *J* = 5.1 Hz, 1H, CONH), 5.16 (d, *J* = 5.4 Hz, 2H, CONHC$\underline{H_2}$), 2.33 (s, 3H, CH_3CO); ^{13}C NMR (75 MHz, $CDCl_3$): δ 187.3 ($CH_3\underline{C}O$), 164.5 (C=$\underline{C}$(NH)CCl_3), 162.3 (CONH), 131.8 (B-Ph(*o*)), 131.6, 131.4, 130.9, 130.0, 129.2, 128.5, 127.9, 127.9, 127.6 (B-Ph(*m*)), 127.5, 127.1 (B-Ph(*o*)), 126.4, 125.8, 125.6, 125.2, 125.0, 124.9, 123.0, 105.3 ($\underline{C}$=C(NH)CCl_3), 92.9 (CCl_3), 42.9 ($CH_3CO\underline{C}H_2$), 23.6 ($\underline{C}H_3CO$), B-Ph(*i*) quaternary signal not observed; IR: 3320 (N-H), 3288 (N-H), 1630 (C=O), 1597 (C=C, conjugated), 1521 (C=C-NH) cm^{-1}; MS(ES$^-$) (*m/z*): 620.2 [M-H, ^{10}B, ^{35}Cl, ^{35}Cl, ^{35}Cl, 20%]$^-$, 621.2 [M-H, ^{11}B, ^{35}Cl, ^{35}Cl, ^{35}Cl, 98%]$^-$, 622.2 [M-H, ^{10}B, ^{35}Cl, ^{35}Cl, ^{37}Cl, 47%]$^-$, 623.2 [M-H, ^{11}B, ^{35}Cl, ^{35}Cl, ^{37}Cl, 100%]$^-$, 624.2 [M-H, ^{10}B, ^{35}Cl, ^{37}Cl, ^{37}Cl, 33%]$^-$, 625.2 [M-H, ^{11}B, ^{35}Cl, ^{37}Cl, ^{37}Cl, 19%]$^-$, 626.2 [M-H, ^{10}B, ^{37}Cl, ^{37}Cl, ^{37}Cl, 9%]$^-$, 627.3 [M-H, ^{11}B, ^{37}Cl, ^{37}Cl, ^{37}C, 4%]$^-$; MS(ES$^+$) (*m/z*): 622.2 [M+H, ^{10}B, ^{35}Cl, ^{35}Cl, ^{35}Cl, 11%]$^+$, 623.2 [M+H, ^{11}B, ^{35}Cl, ^{35}Cl, ^{35}Cl, 69%]$^+$, 624.2 [M+H, ^{10}B, ^{35}Cl, ^{35}Cl, ^{37}Cl, 30%]$^+$, 625.2 [M+H, ^{11}B, ^{35}Cl, ^{35}Cl, ^{37}Cl, 100%]$^+$, 626.2 [M+H, ^{10}B, ^{35}Cl, ^{37}Cl, ^{37}Cl, 36%]$^+$, 627.2 [M+H, ^{11}B, ^{35}Cl, ^{37}Cl, ^{37}Cl, 28%]$^+$, 628.2 [M+H, ^{10}B, ^{37}Cl, ^{37}Cl, ^{37}Cl, 5%]$^+$, 629.2 [M+H, ^{11}B, ^{37}Cl, ^{37}Cl, ^{37}Cl, 3%]$^+$.

HRMS(ES$^-$) (*m/z*): [M-H]$^-$ calcd. for $C_{35}H_{25}{}^{11}B^{35}Cl_3N_2O_2$, 621.1080; found, 621.1072, error: 1.3 ppm.

***N*-Pyridin-4-ylacetoacetamide (3i)**. Yield: 57%, cream solid. mp: 92-93°C; ^{1}H NMR (300 MHz, $CDCl_3$): δ 9.61 (br s, 1H, NH), 8.53 (d, *J* = 6.0 Hz, 2H, Py-*m*), 7.52 (d, *J* = 6.0 Hz, 2H, Py-*o*), 3.64 (s, 2H, $CH_3CO\underline{CH_2}$), 2.36 (s, 3H, CH_3CO); ^{13}C NMR (75 MHz, $CDCl_3$): δ 204.7 ($CH_3\underline{C}O$), 164.4 (CONH), 150.6 (Py(*m*)), 144.7 (Py(*i*)), 114.0 (Py(*o*)), 49.8 ($CH_3CO\underline{C}H_2$), 31.2 ($\underline{C}H_3CO$); IR: 3245 (N-H), 1721 (C=O), 1690 (C=O), 1595 (C=O) cm^{-1}; MS(ES$^-$) (*m/z*): 176.9 [M-H, 100%]$^-$; MS(ES$^+$) (*m/z*): 178.9 [M+H, 100%]$^+$, 200.9 [M+Na, 11%]$^+$. HRMS(ES$^+$) (*m/z*): [M+H]$^+$ calcd. for $C_9H_{11}N_2O_2$, 179.0815; found, 179.0814, error: 0.6 ppm.

(*Z*)-2-Acetyl-3-amino-4,4,4-trichloro-*N*-(pyridine-4-yl)but-2-enamide (4i). Yield: 64%, cream solid. mp: 105-107 ^{o}C (dec); ^{1}H NMR (300 MHz, $CDCl_3$): δ 8.55 (d, *J* = 6.0 Hz, 2H, Py-*m*), 7.92 (br s, 1H, NH), 7.51 (d, *J* = 6.0 Hz, 2H, Py-*o*), 2.37 (s, 3H, CH_3CO), NH_2 signal not observed; ^{13}C NMR (75 MHz, $CDCl_3$): δ 166.3 ($C=\underline{C}(NH_2)CCl_3$), 157.6 (CONH), 150.9 (Py-*m*), 144.9 (Py-*i*), 113.4 (Py-*o*), 104.7 ($\underline{C}=C(NH_2)CCl_3$), 93.2 ($CCl_3$), 28.9 ($\underline{C}H_3CO$), $CH_3\underline{C}O$ quaternary signal not observed; IR: 3230 (N-H), 1654 (C=O), 1585 (C=C, conjugated), 1506 (C=C-NH_2) cm^{-1}; MS(ES$^-$) (*m/z*): 319.9 [M-H, ^{35}Cl, ^{35}Cl, ^{35}Cl, 72%]$^-$, 321.9 [M-H, ^{35}Cl, ^{35}Cl, ^{37}Cl, 100%]$^-$, 323.9 [M-H, ^{35}Cl, ^{37}Cl, ^{37}Cl, 25%]$^-$, 326.0 [M-H, ^{37}Cl, ^{37}Cl, ^{37}Cl, 3%]$^-$; MS(ES$^+$) (*m/z*): 322.0 [M+H, ^{35}Cl, ^{35}Cl, ^{35}Cl, 84%]$^+$, 324.0 [M+H, ^{35}Cl, ^{35}Cl, ^{37}Cl, 100%]$^+$, 326.0 [M+H, ^{35}Cl, ^{37}Cl, ^{37}Cl, 70%]$^+$, 328.0 [M+H, ^{37}Cl, ^{37}Cl, ^{37}Cl, 5%]$^+$. HRMS(APCI$^+$) (*m/z*): [M+H]$^+$ calcd. for $C_{11}H_{11}{}^{35}Cl_3N_3O_2$, 321.9911; found, 321.9910, error: 0.3 ppm.

5-Acetyl-2,2-diphenyl-6-(pyridine-4-ylamino)-4-(trichloromethyl)-2,3-dihydro-1,3,2-oxazaborinin-1-ium-2-uide (NBC22). Ethyl acetate/*n*-hexane, 3:5. Yield: 56%,

yellow solid. mp: 124-126 ^{o}C (dec); ^{1}H NMR (300 MHz, $CDCl_3$): δ 8.49 (br s, 2H, Py-*m*), 8.01 (br s, 1H, NH), 7.66 (br s, 1H, NH), 7.21-7.47 (m, 12H, Py-*o* & Aryl-H), 2.43 (s, 3H, CH_3CO); IR: 3337 (N-H), 3247 (N-H), 1684 (C=O), 1586 (C=C, conjugated), 1508 (C=C-NH) cm^{-1}; MS(ES^{-}) (*m*/*z*): 482.9 [M-H, ^{10}B, ^{35}Cl, ^{35}Cl, ^{35}Cl, 5%]$^{-}$, 483.9 [M-H, ^{11}B, ^{35}Cl, ^{35}Cl, ^{35}Cl, 21%]$^{-}$, 485.0 [M-H, ^{10}B, ^{35}Cl, ^{35}Cl, ^{37}Cl, 7%]$^{-}$, 486.1 [M-H, ^{11}B, ^{35}Cl, ^{35}Cl, ^{37}Cl, 22%]$^{-}$, 487.2 [M-H, ^{10}B, ^{35}Cl, ^{37}Cl, ^{37}Cl, 6%]$^{-}$, 488.2 [M-H, ^{11}B, ^{35}Cl, ^{37}Cl, ^{37}Cl, 5%]$^{-}$, 489.2 [M-H, ^{10}B, ^{37}Cl, ^{37}Cl, ^{37}Cl, 3%]$^{-}$, 490.2 [M-H, ^{11}B, ^{37}Cl, ^{37}Cl, ^{37}C, 1%]$^{-}$; MS(ES^{+}) (*m*/*z*): 485.1 [M+H, ^{10}B, ^{35}Cl, ^{35}Cl, ^{35}Cl, 4%]$^{+}$, 486.1 [M+H, ^{11}B, ^{35}Cl, ^{35}Cl, ^{35}Cl, 26%]$^{+}$, 487.1 [M+H, ^{10}B, ^{35}Cl, ^{35}Cl, ^{37}Cl, 11%]$^{+}$, 488.1 [M+H, ^{11}B, ^{35}Cl, ^{35}Cl, ^{37}Cl, 22%]$^{+}$, 489.1 [M+H, ^{10}B, ^{35}Cl, ^{37}Cl, ^{37}Cl, 3%]$^{+}$, 490.1 [M+H, ^{11}B, ^{35}Cl, ^{37}Cl, ^{37}Cl, 7%]$^{+}$, 491.1 [M+H, ^{10}B, ^{37}Cl, ^{37}Cl, ^{37}Cl, 2%]$^{+}$, 492.1 [M+H, ^{11}B, ^{37}Cl, ^{37}Cl, ^{37}Cl, 1%]$^{+}$. HRMS(APCI^{+}) (*m*/*z*): [M+H]$^{+}$ calcd. for $C_{23}H_{20}{}^{11}B^{35}Cl_3N_3O_2$, 486.0709; found, 486.0709, error: 0.0 ppm.

3-(4-(2-((3-Oxobutanoyl)oxy)ethyl)piperazino)-1-propanesulfonic acid (3j). The reaction mixture was purified by flash column chromatography. MeOH/DCM, 1:3. Yield: 75%, orange solid. mp: 107-109 ^{o}C; ^{1}H NMR (300 MHz, DMSO-d_6): δ 9.75 (br s, 1H, OH), 4.16 (t, *J* = 5.4 Hz, 2H, $OC\underline{H_2}CH_2N$), 3.61 (s, 2H, $CH_3COC\underline{H_2}$), 3.24-3.55 (m, 4H, Piperazine-H_{eq}), 2.76-3.09 (m, 4H, Piperazine-H_{ax}), 2.63 (t, *J* = 5.9 Hz, 4H, $OCH_2C\underline{H_2}N$ & $NCH_2CH_2C\underline{H_2}SO_3H$), 2.21-2.45 (m, 2H, $NC\underline{H_2}CH_2CH_2SO_3H$), 2.18 (s, 3H, CH_3CO), 1.97 (p, *J* = 6.3 Hz, 2H, $NCH_2C\underline{H_2}CH_2SO_3H$); ^{13}C NMR (75 MHz, DMSO-d_6): δ 201.5 ($CH_3\underline{C}O$), 167.2 ($\underline{C}OOCH_2$), 61.7 ($O\underline{C}H_2CH_2N$), 55.7, 54.9, 51.1, 49.7, 49.6, 49.1, 30.1 ($\underline{C}H_3CO$), 19.8 ($NCH_2\underline{C}H_2CH_2SO_3H$); MS(ES^{-}) (*m*/*z*): 335.1 [M-H, 100%]$^{-}$; MS(ES^{+}) (*m*/*z*): 337.1 [M+H, 23%]$^{+}$, 359.2 [M+Na, 100%]$^{+}$, 695.4 [2M+Na, 72%]$^{+}$; HRMS(ES^{+}) (*m*/*z*): [M+Na]$^{+}$ calcd. for $C_{13}H_{24}N_2O_6SNa$, 359.1247; found, 359.1243, error: 1.2 ppm.

(*Z*)-3-(4-(2-((2-Acetyl-3-amino-4,4,4-trichlorobut-2-enoyl)oxy)ethyl)piperazino)-1-propanesulfonic acid (4j). Anhydrous MeOH (5 ml) was used as solvent. Yield: 88%, orange solid. mp: 69-71 ^{o}C; ^{1}H NMR (300 MHz, DMSO-d$_6$): δ 9.79 (br s, 1H, OH), 4.21 (t, *J* = 5.3 Hz, 2H, OC$\underline{H}_2$CH$_2$N), 3.47 (br s, 2H, Piperazine-H$_{eq}$), 3.17 (br s, 2H, Piperazine-H$_{ax}$), 3.02 (br s, 2H, Piperazine-H$_{eq}$), 2.87 (br s, 2H, Piperazine-H$_{ax}$), 2.56-2.76 (m, 4H, OCH$_2$C$\underline{H}_2$N & NCH$_2$CH$_2$C$\underline{H}_2$SO$_3$H), 2.23-2.40 (m, 2H, NC$\underline{H}_2$CH$_2$CH$_2$SO$_3$H), 2.21 (s, 3H, CH$_3$CO), 1.98 (br s, 2H, NCH$_2$C$\underline{H}_2$CH$_2$SO$_3$H); ^{13}C NMR (75 MHz, DMSO-d$_6$): δ 195.4 (CH$_3\underline{C}$O), 167.9 (C=$\underline{C}$(NH$_2$)CCl$_3$), 158.9 (COO), 100.2 ($\underline{C}$=C(NH$_2$)CCl$_3$), 92.4 (CCl$_3$), 61.9 (O$\underline{C}$H$_2$CH$_2$N), 55.7, 54.6, 51.0, 49.6, 49.2, 29.0 ($\underline{C}$H$_3$CO), 19.6 (NCH$_2\underline{C}$H$_2$CH$_2$SO$_3$H); MS(ES$^-$) (*m/z*): 436.0 [M-C$_2$H$_3$O, ^{35}Cl, ^{35}Cl, ^{35}Cl, 60%]$^-$, 438.0 [M-C$_2$H$_3$O, ^{35}Cl, ^{35}Cl, ^{37}Cl, 69%]$^-$, 440.0 [M-C$_2$H$_3$O, ^{35}Cl, ^{37}Cl, ^{37}Cl, 16%]$^-$, 442.1 [M-C$_2$H$_3$O, ^{37}Cl, ^{37}Cl, ^{37}Cl, 12%]$^-$, 478.1 [M-H, ^{35}Cl, ^{35}Cl, ^{35}Cl, 100%]$^-$, 480.0 [M-H, ^{35}Cl, ^{35}Cl, ^{37}Cl, 53%]$^-$, 482.1 [M-H, ^{35}Cl, ^{37}Cl, ^{37}Cl, 18%]$^-$, 484.0 [M-H, ^{37}Cl, ^{37}Cl, ^{37}Cl, 2%]$^-$; MS(ES$^+$) (*m/z*): 480.0 [M+H, ^{35}Cl, ^{35}Cl, ^{35}Cl, 19%]$^+$, 482.0 [M+H, ^{35}Cl, ^{35}Cl, ^{37}Cl, 15%]$^+$, 484.0 [M+H, ^{35}Cl, ^{37}Cl, ^{37}Cl, 4%]$^+$, 486.0 [M+H, ^{37}Cl, ^{37}Cl, ^{37}Cl, 2%]$^+$, 502.1 [M+Na, ^{35}Cl, ^{35}Cl, ^{35}Cl, 73%]$^+$, 504.1 [M+Na, ^{35}Cl, ^{35}Cl, ^{37}Cl, 100%]$^+$, 506.0 [M+Na, ^{35}Cl, ^{37}Cl, ^{37}Cl, 39%]$^+$, 508.0 [M+Na, ^{37}Cl, ^{37}Cl, ^{37}Cl, 3%]$^+$; HRMS(ES$^+$) (*m/z*): [M+Na]$^+$ calcd. for C$_{15}$H$_{24}$^{35}Cl$_3$N$_3$O$_6$SNa, 502.0344; found, 502.0343, error: 0.2 ppm.

6-Methyl-2,2-diphenyl-5-((2-(4-(3-sulfopropyl)piperazin-1-yl)ethoxy)carbonyl)-4(trichloromethyl)-2,3-dihydro-1,3,2-oxazaborinin-1-ium-2-uide (NBC-EPPS). MeOH/DCM, 3:5. Yield: 56%, yellow solid. mp: 104-106 ^{o}C (dec), exists with minor impurity that could not be separated; ^{1}H NMR (300 MHz, DMSO-d$_6$): 10.82 (br s, 1H, NH), 9.83 (br s, 1H, OH), 7.13-7.35 (m, 10H, Aryl-H), 4.14 (br s, 2H, OC$\underline{H}_2$CH$_2$N), 3.53-3.65 (m, minor), 3.38-3.47 (m, 2H, Piperazine-H), 3.12-3.25 (m, 2H, Piperazine-

H), 2.74-2.98 (m, 4H, Piperazine-H), 2.55-2.72 (m, 4H, $OCH_2C\underline{H}_2N$ & $NCH_2CH_2C\underline{H}_2SO_3H$), 2.44 (s, 3H, CH_3CO), 2.25 (m, 2H, $NC\underline{H}_2CH_2CH_2SO_3H$), 1.90-2.07 (m, 2H, $NCH_2C\underline{H}_2CH_2SO_3H$), 1.71-1.80 (m, minor); ^{13}C NMR (75 MHz, DMSO-d_6): 185.8 ($CH_3\underline{C}O$), 164.8, 164.7, 131.9 (B-Ph(*o*)), 126.9 (B-Ph(*m*)), 126.3 (B-Ph(*p*)), 101.5 ($\underline{C}$=C(NH)CCl_3), 92.0 (CCl_3), 62.4 ($O\underline{C}H_2CH_2N$), 55.7, 54.3, 50.9, 49.6, 49.2, 23.4 ($\underline{C}H_3CO$), 19.6 ($NCH_2\underline{C}H_2CH_2SO_3H$), B-Ph(*i*) quaternary signal not observed; IR: 3350 (N-H), 1654 (C=O), 1596 (C=C, conjugated), 1520 (C=C-NH) cm^{-1}; MS(ES$^-$) (*m/z*): 608.2 [M-^{35}Cl, ^{10}B, ^{35}Cl, ^{35}Cl, 13%]$^-$, 609.2 [M-^{35}Cl, ^{11}B, ^{35}Cl, ^{35}Cl, 50%]$^-$, 610.2 [M-^{35}Cl, ^{10}B, ^{35}Cl, ^{37}Cl, 32%]$^-$, 611.2 [M-^{35}Cl, ^{11}B, ^{35}Cl, ^{37}Cl, 34%]$^-$, 612.2 [M-^{35}Cl, ^{10}B, ^{37}Cl, ^{37}Cl, 13%]$^-$, 613.3 [M-^{35}Cl, ^{11}B, ^{37}Cl, ^{37}Cl, 5%]$^-$, 642.2 [M-H, ^{10}B, ^{35}Cl, ^{35}Cl, ^{35}Cl, 8%]$^-$, 643.2 [M-H, ^{11}B, ^{35}Cl, ^{35}Cl, ^{35}Cl, 48%]$^-$, 644.2 [M-H, ^{10}B, ^{35}Cl, ^{35}Cl, ^{37}Cl, 51%]$^-$, 645.2 [M-H, ^{11}B, ^{35}Cl, ^{35}Cl, ^{37}Cl, 100%]$^-$, 646.2 [M-H, ^{10}B, ^{35}Cl, ^{37}Cl, ^{37}Cl, 58%]$^-$, 647.2 [M-H, ^{11}B, ^{35}Cl, ^{37}Cl, ^{37}Cl, 50%]$^-$, 648.3 [M-H, ^{10}B, ^{37}Cl, ^{37}Cl, ^{37}Cl, 22%]$^-$, 649.2 [M-H, ^{11}B, ^{37}Cl, ^{37}Cl, ^{37}C, 11%]$^-$; MS(ES$^+$) (*m/z*): 479.1 [M-BPh_2, ^{35}C, ^{35}Cl, ^{35}Cl, 100%]$^+$, 631.2 [M-^{35}Cl+Na, ^{10}B, ^{35}Cl, ^{35}Cl, 1%]$^+$, 632.2 [M-^{35}Cl+Na, ^{11}B, ^{35}Cl, ^{35}Cl, 9%]$^+$, 633.3 [M-^{35}Cl+Na, ^{10}B, ^{35}Cl, ^{37}Cl, 13%]$^+$, 634.2 [M-^{35}Cl, ^{11}B, ^{35}Cl, ^{37}Cl, 20%]$^+$, 635.2 [M-^{35}Cl+Na, ^{10}B, ^{37}Cl, ^{37}Cl, 10%]$^+$, 636.2 [M-^{35}Cl+Na, ^{11}B, ^{37}Cl, ^{37}Cl, 8%]$^+$, 665.2 [M+Na, ^{10}B, ^{35}Cl, ^{35}Cl, ^{35}Cl, 2%]$^+$, 666.2 [M+Na, ^{11}B, ^{35}Cl, ^{35}Cl, ^{35}Cl, 10%]$^+$, 667.2 [M+Na, ^{10}B, ^{35}Cl, ^{35}Cl, ^{37}Cl, 13%]$^+$, 668.2 [M+Na, ^{11}B, ^{35}Cl, ^{35}Cl, ^{37}Cl, 20%]$^+$, 669.2 [M+Na, ^{10}B, ^{35}Cl, ^{37}Cl, ^{35}Cl, 18%]$^+$, 670.2 [M+Na, ^{11}B, ^{35}Cl, ^{37}Cl, ^{37}Cl, 30%]$^+$, 671.2 [M+Na, ^{10}B, ^{37}Cl, ^{37}Cl, ^{37}Cl, 13%]$^+$, 672.2 [M+Na, ^{11}B, ^{37}Cl, ^{37}Cl, ^{37}Cl, 11%]$^+$. HRMS(APCI$^+$) (*m/z*): [M+Na]$^+$ calcd. for $C_{27}H_{33}{}^{11}B^{35}Cl_3N_3O_6SNa$, 666.1141; found, 666.1140, error: 0.2 ppm.

1.5 General Procedure for Diazaborine Reaction

An appropriate amine (2.5 mmol) was added to 6-methyl-2,2-diphenyl-4-(trichloromethyl)-2,3-dihydro-1,3,2-oxazaborinin-1-ium-2-uide (**3b**, 0.82 mmol) in anhydrous THF (total reaction volume of 5 ml). The mixture was stirred at 50°C under N_2 for 24 h, unless otherwise stated. The reaction mixture was concentrated and purified by flash column chromatography to give the corresponding diazaborine product (Vasilev et al., 2013).

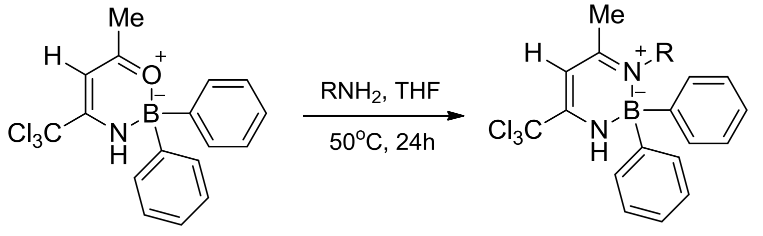

Scheme 6 | Synthesis of NBC27 and NBC30-31.

1,6-Dimethyl-2,2-diphenyl-4-(trichloromethyl)-2,3-dihydro-1,3,2-diazaborinin-1-ium-2-uide (NBC27, 18). Ethyl acetate/*n*-hexane, 1:9. Yield: 90%, yellow solid. mp: 117-118 °C; ^{1}H NMR (300 MHz, $CDCl_3$): δ 7.33 (d, *J* = 6.9 Hz, 4H, B-Ph(*o*)), 7.12-7.27 (m, 6H, B-Ph(*m*/*p*)), 5.67 (br s, 1H, $Cl_3C(N\underline{H})C=CH$), 5.33 (d, *J* = 2.7 Hz, 1H, $Cl_3C(NH)C=C\underline{H}$), 2.81 (s, 3H, CH_3N), 2.10 (s, 3H, CH_3CN); ^{13}C NMR (75 MHz, $CDCl_3$): δ 167.4 ($Cl_3C(NH)\underline{C}=C$), 158.8 ($CH_3\underline{C}N$), 133.2 (B-Ph(*o*)), 127.3 (B-Ph(*m*)), 126.2 (B-Ph(*p*)), 94.2 (CCl_3), 89.0 ($Cl_3C(NH)C=\underline{C}$), 37.4 (CH_3N), 21.6 ($\underline{C}H_3CN$), B-Ph(*i*) quaternary signal not observed; $^{11}B\{^{1}H\}$ (128 MHz, $CDCl_3$): δ 0.54; IR: 3370 (N-H), 1595 (C=C, conjugated), 1529 (C=C-NH) cm^{-1}; MS(ES^+) (*m/z*): 300.0 [M-Ph, ^{10}B, ^{35}Cl, ^{35}Cl, ^{35}Cl, 35%]$^+$, 301.0 [M-Ph, ^{11}B, ^{35}Cl, ^{35}Cl, ^{35}Cl, 100%]$^+$, 302.0 [M-Ph, ^{10}B, ^{35}Cl, ^{35}Cl, ^{37}Cl, 32%]$^+$, 303.0 [M-Ph, ^{11}B, ^{35}Cl, ^{35}Cl, ^{37}Cl, 69%]$^+$, 304.0 [M-Ph, ^{10}B,

^{35}Cl, ^{37}Cl, ^{37}Cl, 30%]$^+$, 305.0 [M-Ph, ^{11}B, ^{35}Cl, ^{37}Cl, ^{37}Cl, 27%]$^+$, 306.0 [M-Ph, ^{10}B, ^{37}Cl, ^{37}Cl, ^{37}Cl, 3%]$^+$, 307.0 [M-Ph, ^{11}B, ^{37}Cl, ^{37}Cl, ^{37}Cl, 4%]$^+$, 378.0 [M+H, ^{10}B, ^{35}Cl, ^{35}Cl, ^{35}Cl, 2%]$^+$, 379.0 [M+H, ^{11}B, ^{35}Cl, ^{35}Cl, ^{35}Cl, 11%]$^+$, 380.0 [M+H, ^{10}B, ^{35}Cl, ^{35}Cl, ^{37}Cl, 3%]$^+$, 381.0 [M+H, ^{11}B, ^{35}Cl, ^{35}Cl, ^{37}Cl, 10%]$^+$, 382.0 [M+H, ^{10}B, ^{35}Cl, ^{37}Cl, ^{37}Cl, 4%]$^+$, 383.0 [M+H, ^{11}B, ^{35}Cl, ^{37}Cl, ^{37}Cl, 5%]$^+$, 384.0 [M+H, ^{10}B, ^{37}Cl, ^{37}Cl, ^{37}Cl, 1%]$^+$, 385.0 [M+H, ^{11}B, ^{37}Cl, ^{37}Cl, ^{37}Cl, 2%]$^+$; HRMS(ES$^+$) (*m*/*z*): [M+H]$^+$ calcd. for $C_{18}H_{19}{}^{11}B^{35}Cl_3N_2$, 379.0701; found, 379.0699, error: 0.5 ppm.

1-Ethyl-6-methyl-2,2-diphenyl-4-(trichloromethyl)-2,3-dihydro-1,3,2-diazaborinin-1-ium-2-uide (NBC30, 19). Ethyl acetate/*n*-hexane, 1:24. Yield: 89%, yellow solid. mp: 104-106 ^{o}C (dec); ^{1}H NMR (300 MHz, $CDCl_3$): δ 7.40 (d, *J* = 6.6 Hz, 4H, B-Ph(*o*)), 7.21-7.35 (m, 6H, B-Ph(*m*/*p*)), 5.89 (br s, 1H, $Cl_3C(N\underline{H})C{=}CH$), 5.42 (d, *J* = 3.0 Hz, 1H, $Cl_3C(NH)C{=}C\underline{H}$), 3.40 (q, *J* = 7.1 Hz, 2H, $CH_3C\underline{H}_2N$), 2.29 (s, 3H, CH_3N), 0.69 (t, *J* = 7.2 Hz, $C\underline{H}_3CH_2N$); ^{13}C NMR (75 MHz, $CDCl_3$): δ 166.8 ($Cl_3C(NH)\underline{C}{=}C$), 158.5 ($CH_3\underline{C}N$), 133.3 (B-Ph(*o*)), 127.1 (B-Ph(*m*)), 126.2 (B-Ph(*p*)), 94.2 (CCl_3), 89.5 ($Cl_3C(NH)C{=}\underline{C}$), 44.4 ($CH_3\underline{C}H_2N$), 20.8 ($CH_3N$), 14.3 ($\underline{C}H_3CH_2N$), B-Ph(*i*) quaternary signal not observed; ^{11}B{^{1}H} (128 MHz, $CDCl_3$): δ 0.25; IR: 3375 (N-H), 1599 (C=C, conjugated), 1523 (C=C-NH) cm^{-1}; MS(APCI$^+$) (*m*/*z*): 314.0 [M-Ph, ^{10}B, ^{35}Cl, ^{35}Cl, ^{35}Cl, 4%]$^+$, 315.0 [M-Ph, ^{11}B, ^{35}Cl, ^{35}Cl, ^{35}Cl, 18%]$^+$, 316.0 [M-Ph, ^{10}B, ^{35}Cl, ^{35}Cl, ^{37}Cl, 3%]$^+$, 317.0 [M-Ph, ^{11}B, ^{35}Cl, ^{35}Cl, ^{37}Cl, 17%]$^+$, 318.0 [M-Ph, ^{10}B, ^{35}Cl, ^{37}Cl, ^{37}Cl, 2%]$^+$, 319.0 [M-Ph, ^{11}B, ^{35}Cl, ^{37}Cl, ^{37}Cl, 5%]$^+$, 320.0 [M-Ph, ^{10}B, ^{37}Cl, ^{37}Cl, ^{37}Cl, 1%]$^+$, 321.0 [M-Ph, ^{11}B, ^{37}Cl, ^{37}Cl, ^{37}Cl, 1%]$^+$, 392.1 [M+H, ^{10}B, ^{35}Cl, ^{35}Cl, ^{35}Cl, 11%]$^+$, 393.1 [M+H, ^{11}B, ^{35}Cl, ^{35}Cl, ^{35}Cl, 40%]$^+$, 394.1 [M+H, ^{10}B, ^{35}Cl, ^{35}Cl, ^{37}Cl, 15%]$^+$, 395.1 [M+H, ^{11}B, ^{35}Cl, ^{35}Cl, ^{37}Cl, 39%]$^+$, 396.1 [M+H, ^{10}B, ^{35}Cl, ^{37}Cl, ^{37}Cl, 9%]$^+$, 397.1 [M+H, ^{11}B, ^{35}Cl, ^{37}Cl, ^{37}Cl, 13%]$^+$, 398.1 [M+H, ^{10}B, ^{37}Cl, ^{37}Cl, ^{37}Cl, 3%]$^+$,

399.1 [M+H, ^{11}B, ^{37}Cl, ^{37}Cl, ^{37}Cl, 1%]$^+$; HRMS(APCI$^+$) (*m*/*z*): [M+H]$^+$ calcd. for $C_{19}H_{21}{}^{11}B^{35}Cl_3N_2$, 393.0858; found, 393.0847, error: 2.8 ppm.

1-Propyl-6-methyl-2,2-diphenyl-4-(trichloromethyl)-2,3-dihydro-1,3,2-diazaborinin-1-ium-2-uide (NBC31). Ethyl acetate/*n*-hexane, 1:24. Yield: 81%, yellow solid. mp: 127-129 °C; ^{1}H NMR (300 MHz, CDCl$_3$): δ 7.39 (d, *J* = 6.6 Hz, 4H, B-Ph(*o*)), 7.20-7.35 (m, 6H, B-Ph(*m*/*p*)), 5.90 (br s, 1H, Cl$_3$C(N*H*)C=CH), 5.41 (d, *J* = 3.0 Hz, 1H, Cl$_3$C(NH)C=C*H*), 3.18-3.28 (m, 2H, CH$_3$CH$_2$C*H*$_2$N), 2.28 (s, 3H, CH$_3$N), 1.02 (sext, *J* = 6.4 Hz, 2H, CH$_3$C*H*$_2$CH$_2$N), 0.51 (t, *J* = 7.4 Hz, C*H*$_3$CH$_2$CH$_2$N); ^{13}C NMR (75 MHz, CDCl$_3$): δ 166.8 (Cl$_3$C(NH)*C*=C), 158.5 (CH$_3$*C*N), 133.2 (B-Ph(*o*)), 127.1 (B-Ph(*m*)), 126.2 (B-Ph(*p*)), 94.2 (CCl$_3$), 89.4 (Cl$_3$C(NH)C=*C*), 51.7 (CH$_3$CH$_2$*C*H$_2$N), 22.5 (CH$_3$*C*H$_2$CH$_2$N), 20.9 (CH$_3$N), 11.2 (*C*H$_3$CH$_2$CH$_2$N), B-Ph(*i*) quaternary signal not observed; ^{11}B{^{1}H} (128 MHz, CDCl$_3$): δ 0.43; IR: 3373 (N-H), 1603 (C=C, conjugated), 1525 (C=C-NH) cm^{-1}; MS(APCI$^+$) (*m*/*z*): 328.0 [M-Ph, ^{10}B, ^{35}Cl, ^{35}Cl, ^{35}Cl, 7%]$^+$, 329.0 [M-Ph, ^{11}B, ^{35}Cl, ^{35}Cl, ^{35}Cl, 29%]$^+$, 330.0 [M-Ph, ^{10}B, ^{35}Cl, ^{35}Cl, ^{37}Cl, 13%]$^+$, 331.0 [M-Ph, ^{11}B, ^{35}Cl, ^{35}Cl, ^{37}Cl, 31%]$^+$, 332.1 [M-Ph, ^{10}B, ^{35}Cl, ^{37}Cl, ^{37}Cl, 6%]$^+$, 333.1 [M-Ph, ^{11}B, ^{35}Cl, ^{37}Cl, ^{37}Cl, 8%]$^+$, 334.1 [M-Ph, ^{10}B, ^{37}Cl, ^{37}Cl, ^{37}Cl, 2%]$^+$, 335.1 [M-Ph, ^{11}B, ^{37}Cl, ^{37}Cl, ^{37}Cl, 1%]$^+$, 406.1 [M+H, ^{10}B, ^{35}Cl, ^{35}Cl, ^{35}Cl, 23%]$^+$, 407.1 [M+H, ^{11}B, ^{35}Cl, ^{35}Cl, ^{35}Cl, 100%]$^+$, 408.1 [M+H, ^{10}B, ^{35}Cl, ^{35}Cl, ^{37}Cl, 42%]$^+$, 409.1 [M+H, ^{11}B, ^{35}Cl, ^{35}Cl, ^{37}Cl, 90%]$^+$, 410.1 [M+H, ^{10}B, ^{35}Cl, ^{37}Cl, ^{37}Cl, 27%]$^+$, 411.1 [M+H, ^{11}B, ^{35}Cl, ^{37}Cl, ^{37}Cl, 36%]$^+$, 412.1 [M+H, ^{10}B, ^{37}Cl, ^{37}Cl, ^{37}Cl, 10%]$^+$, 413.1 [M+H, ^{11}B, ^{37}Cl, ^{37}Cl, ^{37}Cl, 3%]$^+$. HRMS(APCI$^+$) (*m*/*z*): [M+H]$^+$ calcd. for $C_{20}H_{23}{}^{11}B^{35}Cl_3N_2$, 407.1014; found, 407.1016, error: 0.5 ppm.

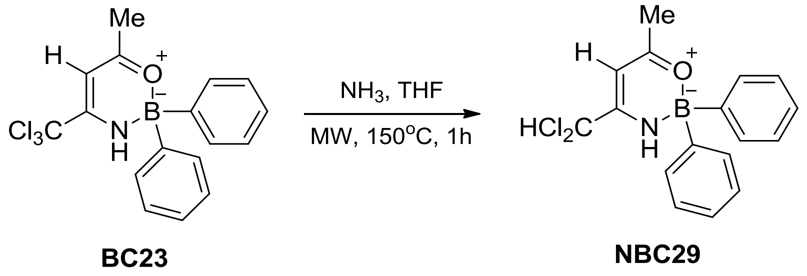

Scheme 7 | Synthesis of NBC29.

4-(Dichloromethyl)-6-Methyl-2,2-diphenyl-2,3-dihydro-1,3,2-oxazaborinin-1-ium-2-uide (NBC29). Ammonia (20.9 mg, 1.23 mmol) was added to **3b** (150 mg, 0.41 mmol) in anhydrous THF (total reaction volume of 3 ml). The mixture was subjected to microwave irradiation at 150°C for 1h in a 5 ml sealed vial. The reaction mixture was concentrated and purified by flash column chromatography (ethyl acetate/*n*-hexane, 2:23). Yield: 92.2 mg (68%), yellow oil. ^{1}H NMR (300 MHz, $CDCl_3$): δ 7.13-7.34 (m, 10H, Aryl-H), 6.06 (s, 1H, CCl_2H), 5.30 (d, *J* = 2.1 Hz, 1H, $Cl_3C(NH)C{=}C\underline{H}$), 2.12 (s, 3H, CH_3CO), NH signal not observed; ^{13}C NMR (75 MHz, $CDCl_3$): δ 185.2 ($CH_3\underline{C}O$), 164.8 ($Cl_3C(NH)\underline{C}{=}C$), 131.8 (B-Ph(*o*)), 127.5 (B-Ph(*m*)), 126.8 (B-Ph(*p*)), 93.2 ($Cl_3C(NH)C{=}\underline{C}$), 67.6 ($CCl_2H$), 24.5 ($\underline{C}H_3CO$), B-Ph(*i*) quaternary signal not observed; ^{11}B{^{1}H} (128 MHz, $CDCl_3$): δ 4.01; IR: 3354 (N-H), 1616 (C=C, conjugated), 1539 (C=C-NH) cm^{-1}; MS(ES^{-}) (*m/z*): 329.0 [M-H, ^{10}B, ^{35}Cl, ^{35}Cl, 15%]$^{-}$, 330.0 [M-H, ^{11}B, ^{35}Cl, ^{35}Cl, 100%]$^{-}$, 331.0 [M-H, ^{10}B, ^{35}Cl, ^{37}Cl, 29%]$^{-}$, 332.0 [M-H, ^{11}B, ^{35}Cl, ^{37}Cl, 40%]$^{-}$, 333.0 [M-H, ^{10}B, ^{37}Cl, ^{37}Cl, 15%]$^{-}$, 334.0 [M-H, ^{11}B, ^{37}Cl, ^{37}Cl, 11%]$^{-}$; MS(ES^{+}) (*m/z*): 253.0 [M-Ph, ^{10}B, ^{35}Cl, ^{35}Cl, 17%]$^{+}$, 253.9 [M-Ph, ^{11}B, ^{35}Cl, ^{35}Cl, 100%]$^{+}$, 255.0 [M-Ph, ^{10}B, ^{35}Cl, ^{37}Cl, 35%]$^{+}$, 256.0 [M-Ph, ^{11}B, ^{35}Cl, ^{37}Cl, 69%]$^{+}$, 257.0 [M-Ph, ^{10}B, ^{37}Cl, ^{37}Cl, 17%]$^{+}$, 257.9 [M-Ph, ^{11}B, ^{37}Cl, ^{37}Cl, 11%]$^{+}$, 330.0 [M+H, ^{10}B, ^{35}Cl, ^{35}Cl, 3%]$^{+}$, 331.0 [M+H, ^{11}B, ^{35}Cl, ^{35}Cl, 12%]$^{+}$, 332.0 [M+H, ^{10}B, ^{35}Cl, ^{37}Cl, 10%]$^{+}$, 333.0 [M+H, ^{11}B, ^{35}Cl, ^{37}Cl, 6%]$^{+}$, 334.0 [M+H, ^{10}B, ^{37}Cl, ^{37}Cl, 7%]$^{+}$, 335.0

[M+H, ^{11}B, ^{37}Cl, ^{37}Cl, 1%]$^+$; HRMS(ES$^-$) (*m/z*): [M-H]$^-$ calcd. for $C_{17}H_{15}{}^{11}B^{35}Cl_2NO$, 330.0629; found, 330.0640, error: 3.3 ppm.

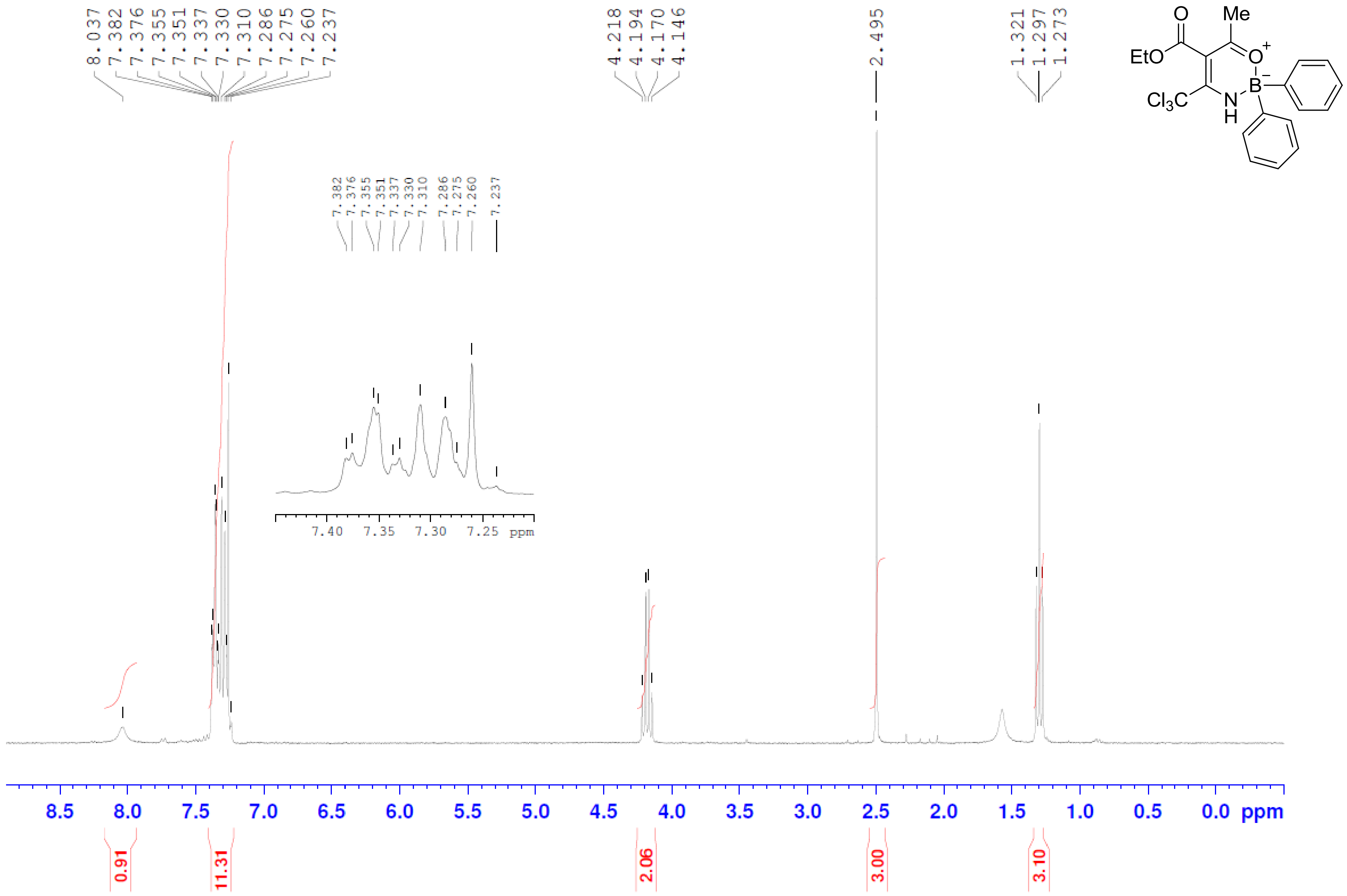

^{1}H NMR spectrum of BC-7 (**13**) in $CDCl_3$

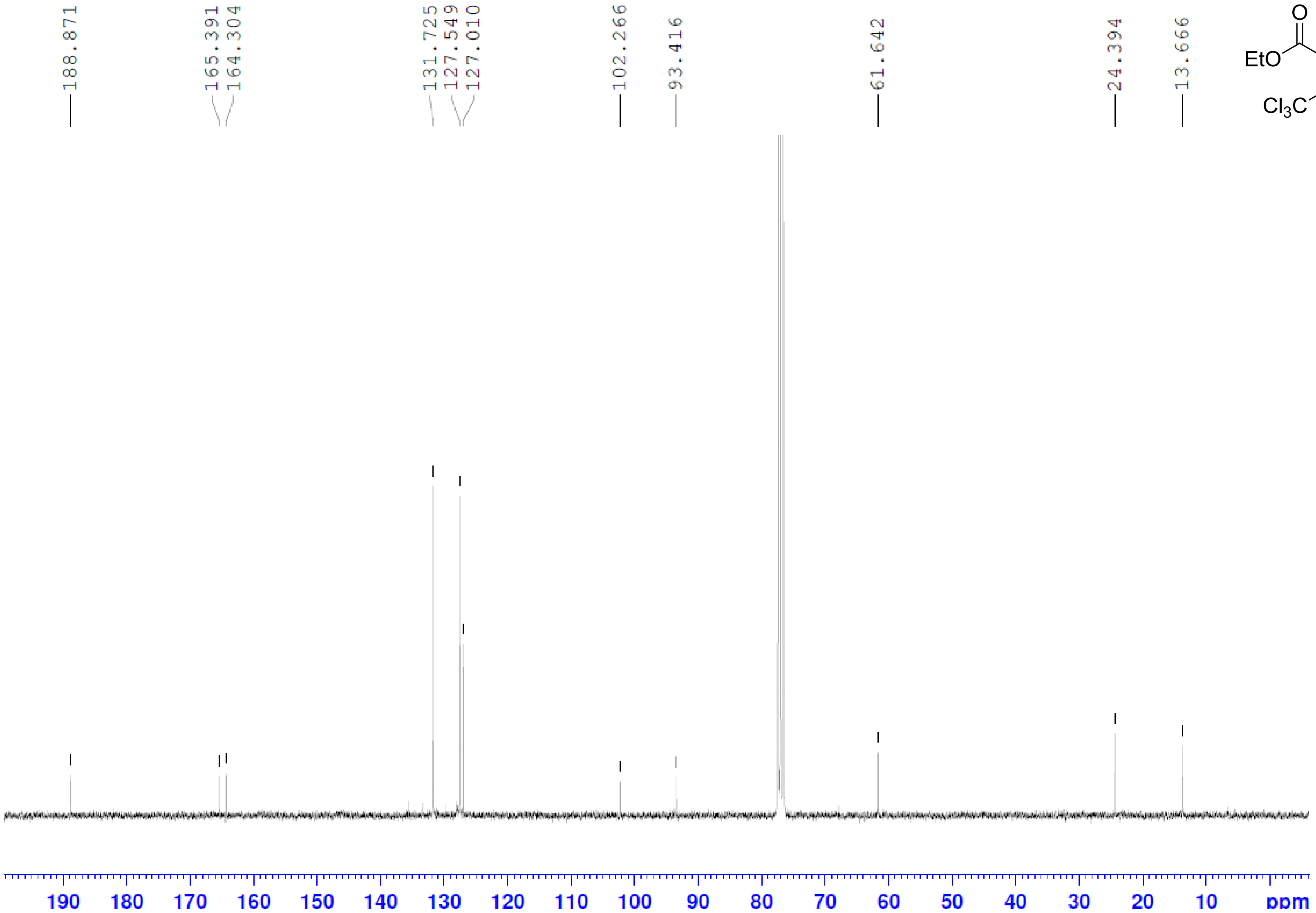

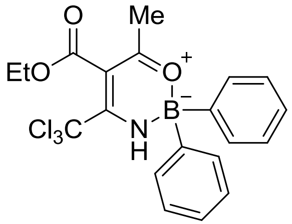

^{13}C NMR spectrum of BC-7 (**13**) in $CDCl_3$

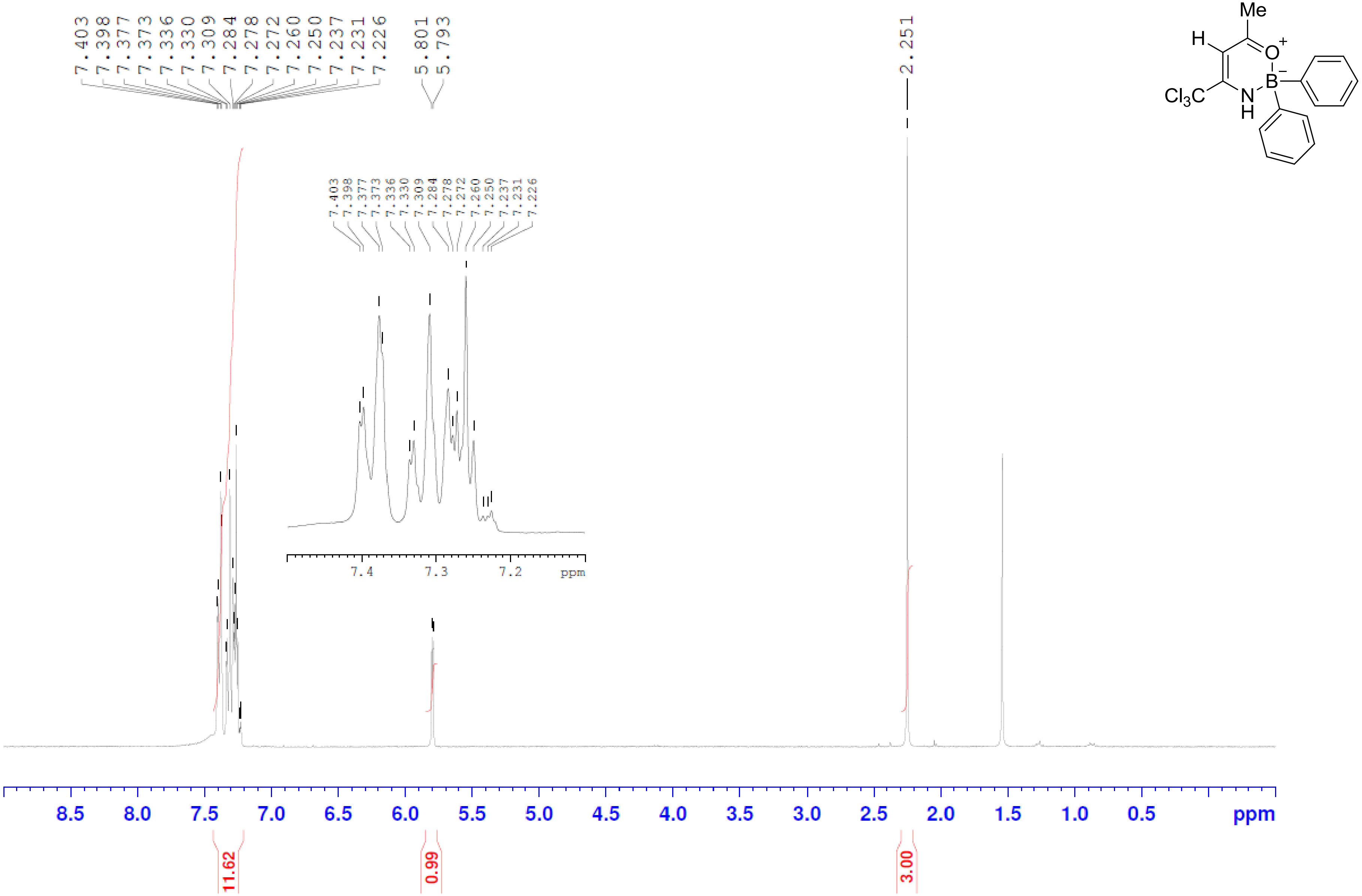

^{1}H NMR spectrum of BC-23 (**14**) in $CDCl_3$

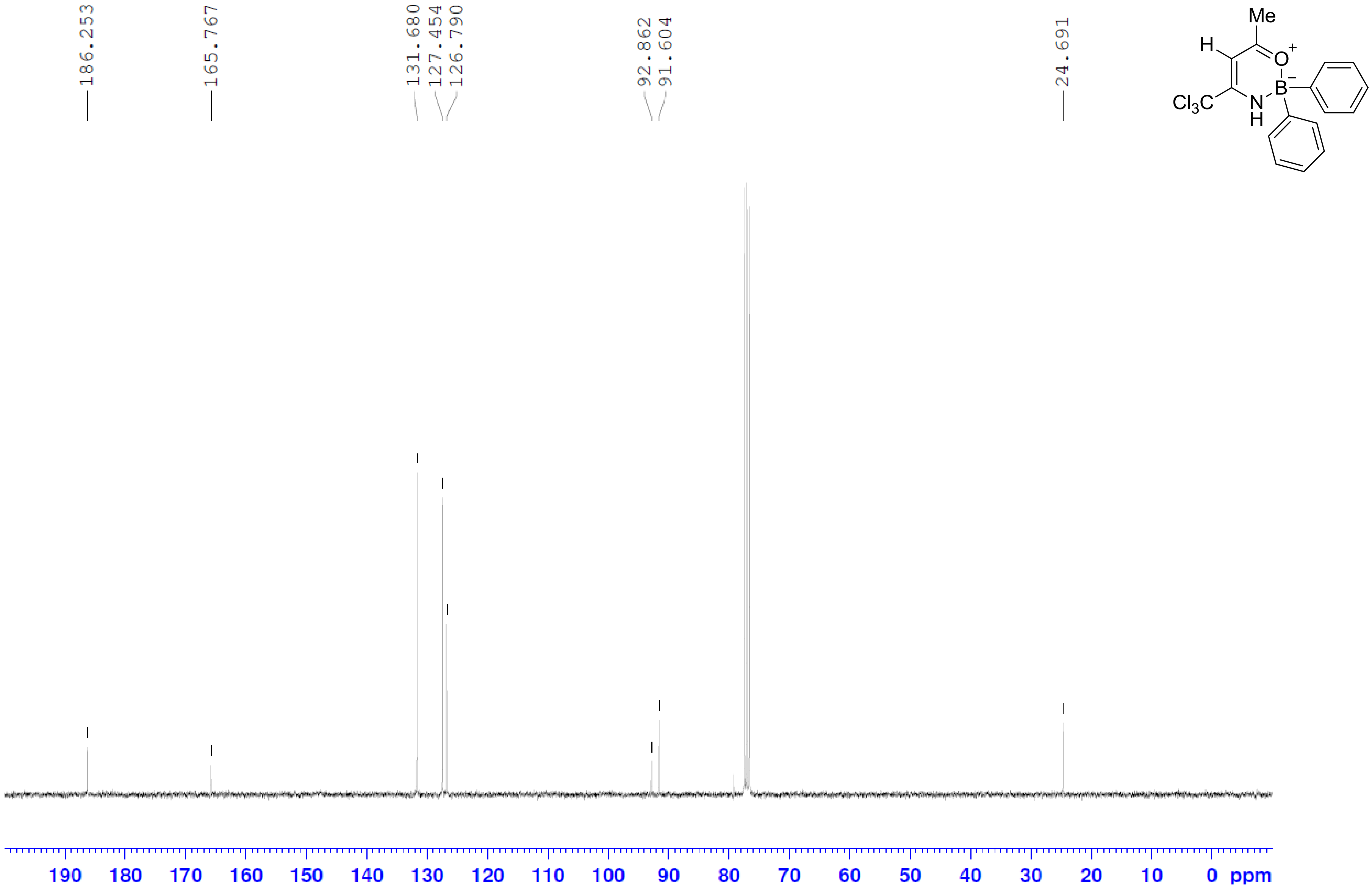

^{13}C NMR spectrum of BC-23 (**14**) in $CDCl_3$

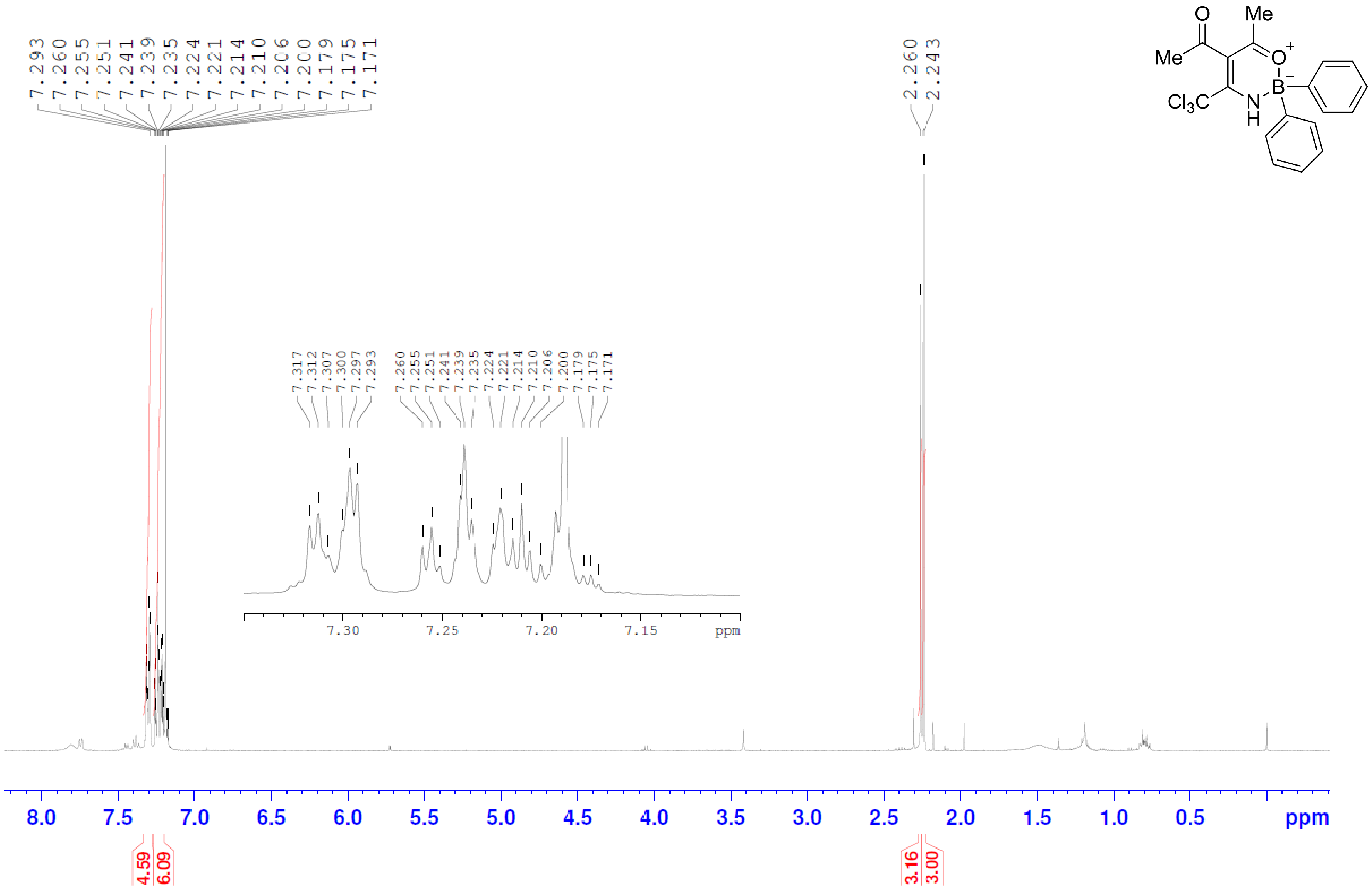

^{1}H NMR spectrum of NBC1 in $CDCl_3$

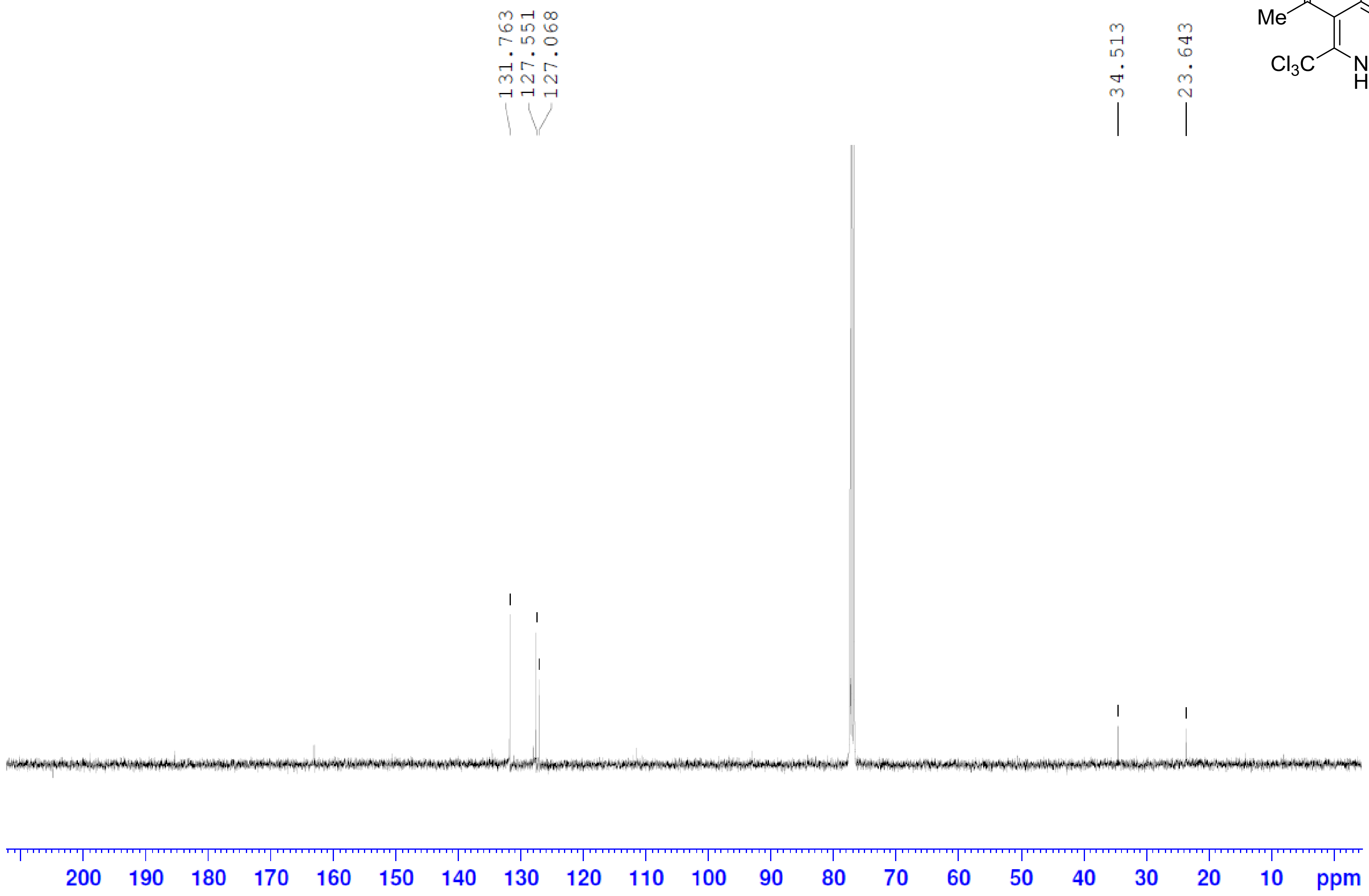

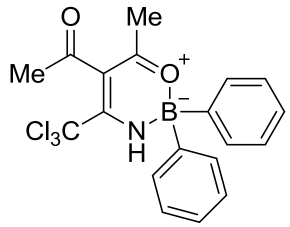

^{13}C NMR spectrum of NBC1 in $CDCl_3$

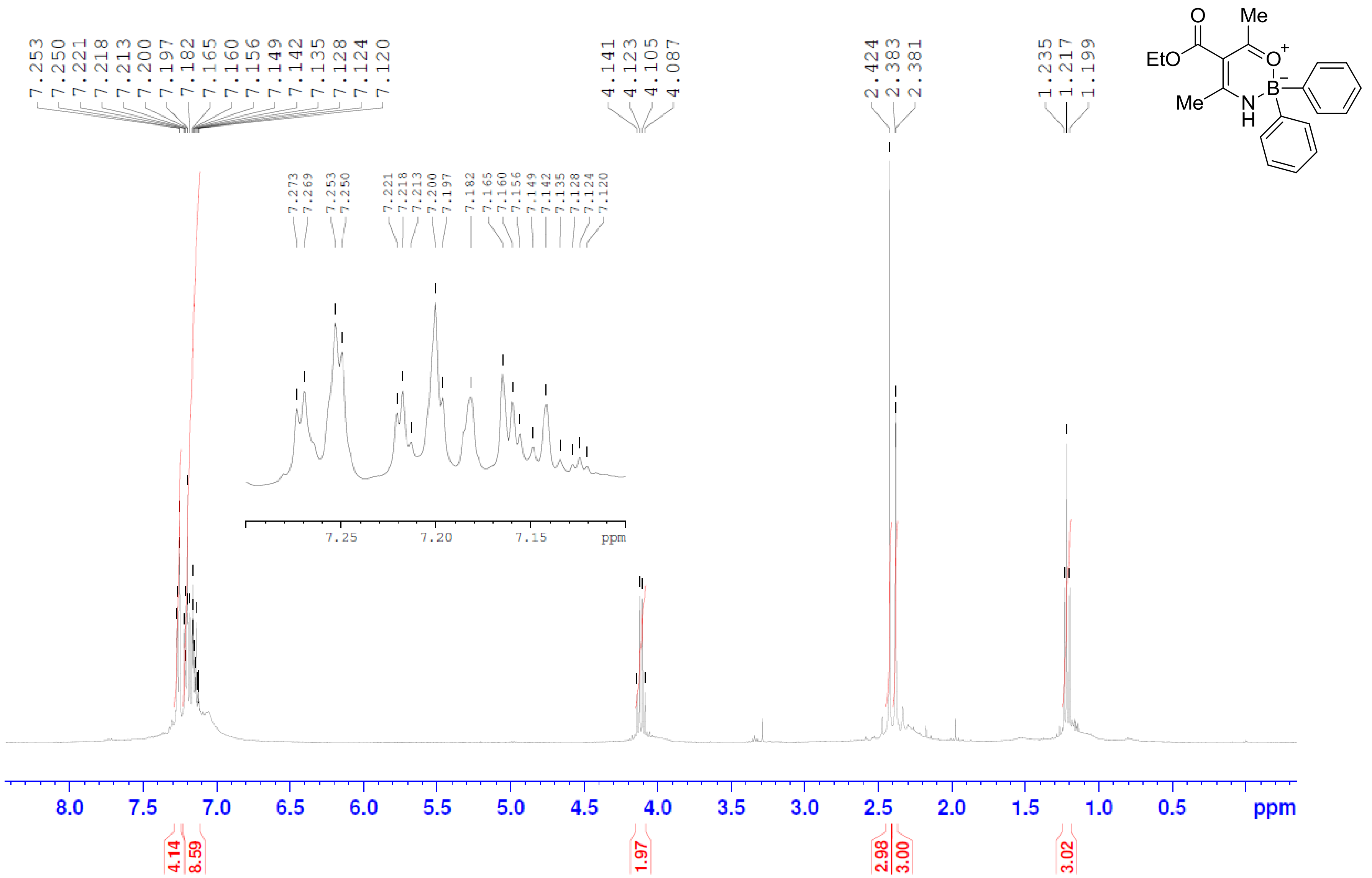

^{1}H NMR spectrum of NBC2 in $CDCl_3$

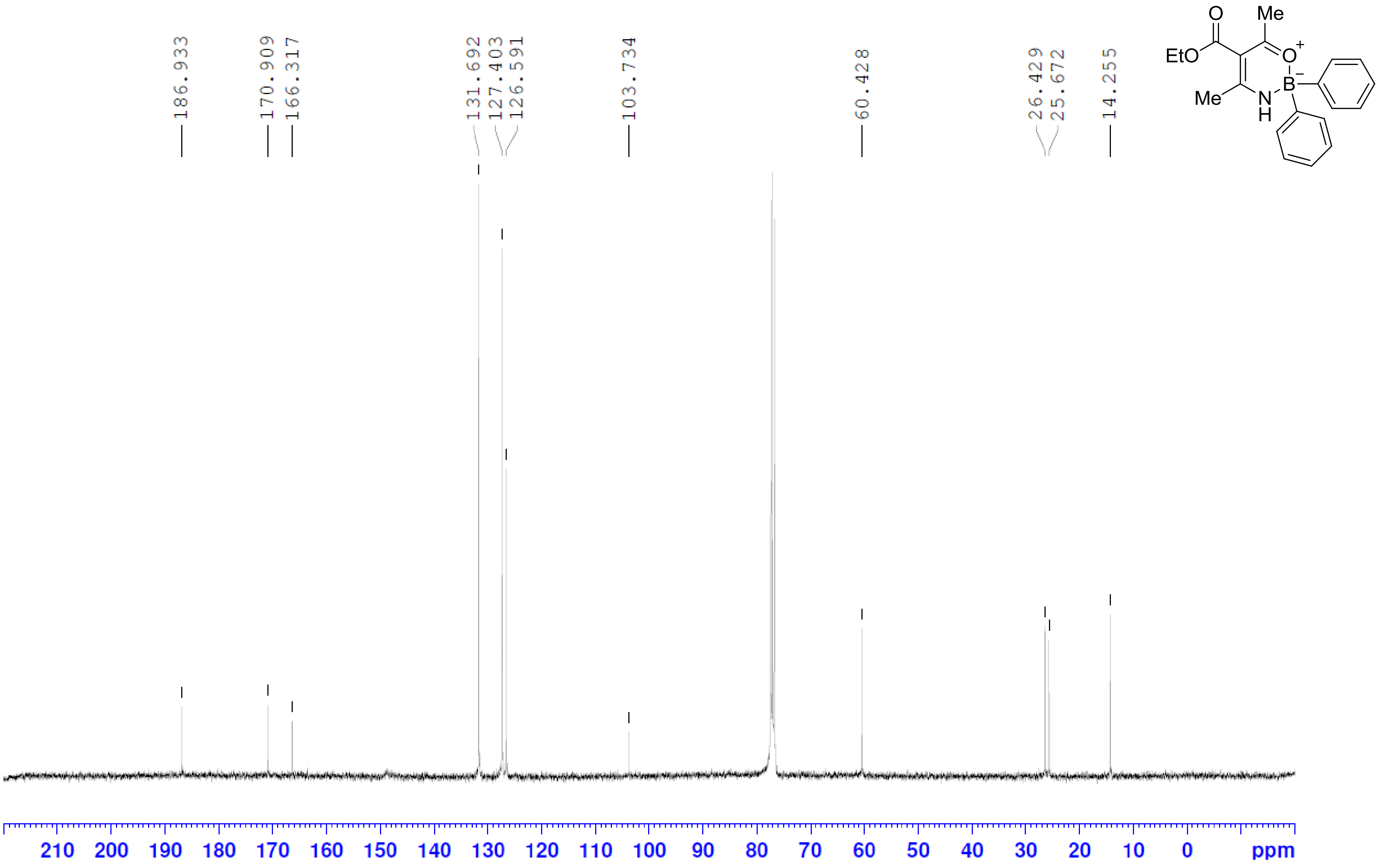

^{13}C NMR spectrum of NBC2 in $CDCl_3$

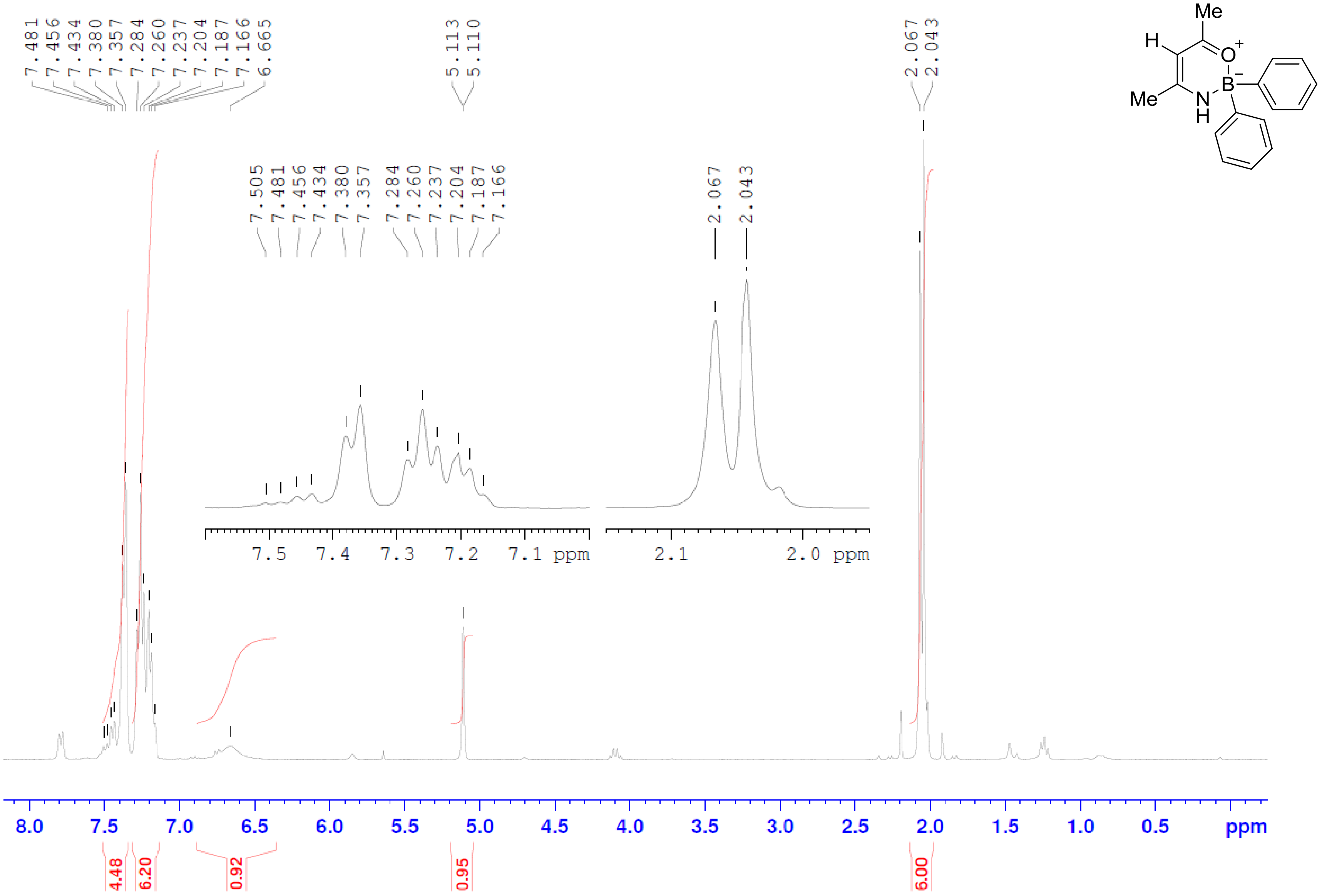

^{1}H NMR spectrum of NBC3 in $CDCl_3$

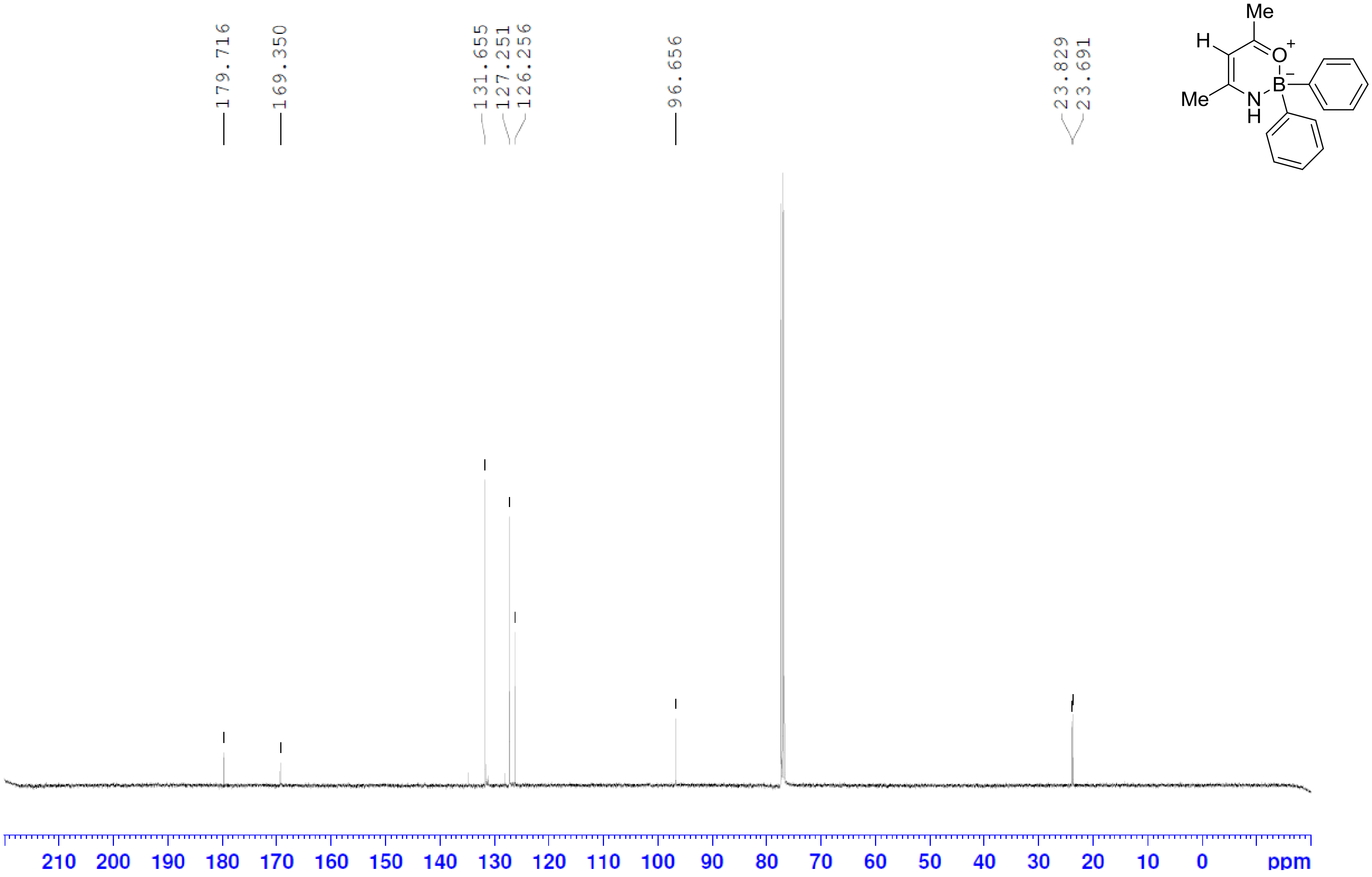

^{13}C NMR spectrum of NBC3 in $CDCl_3$

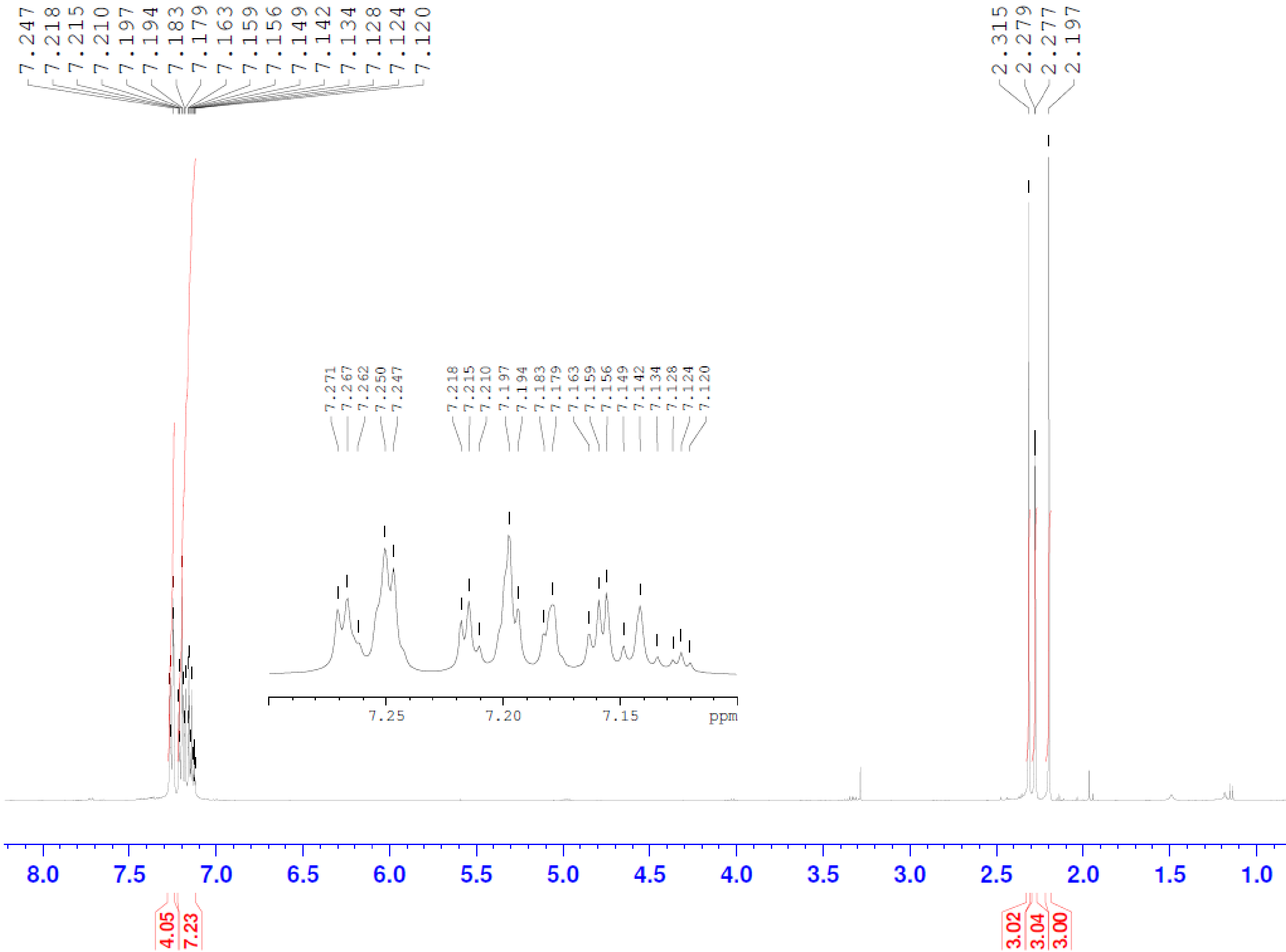

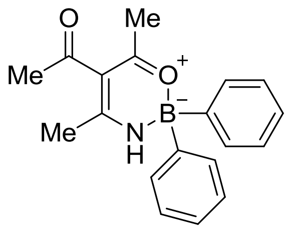

^{1}H NMR spectrum of NBC4 in $CDCl_3$

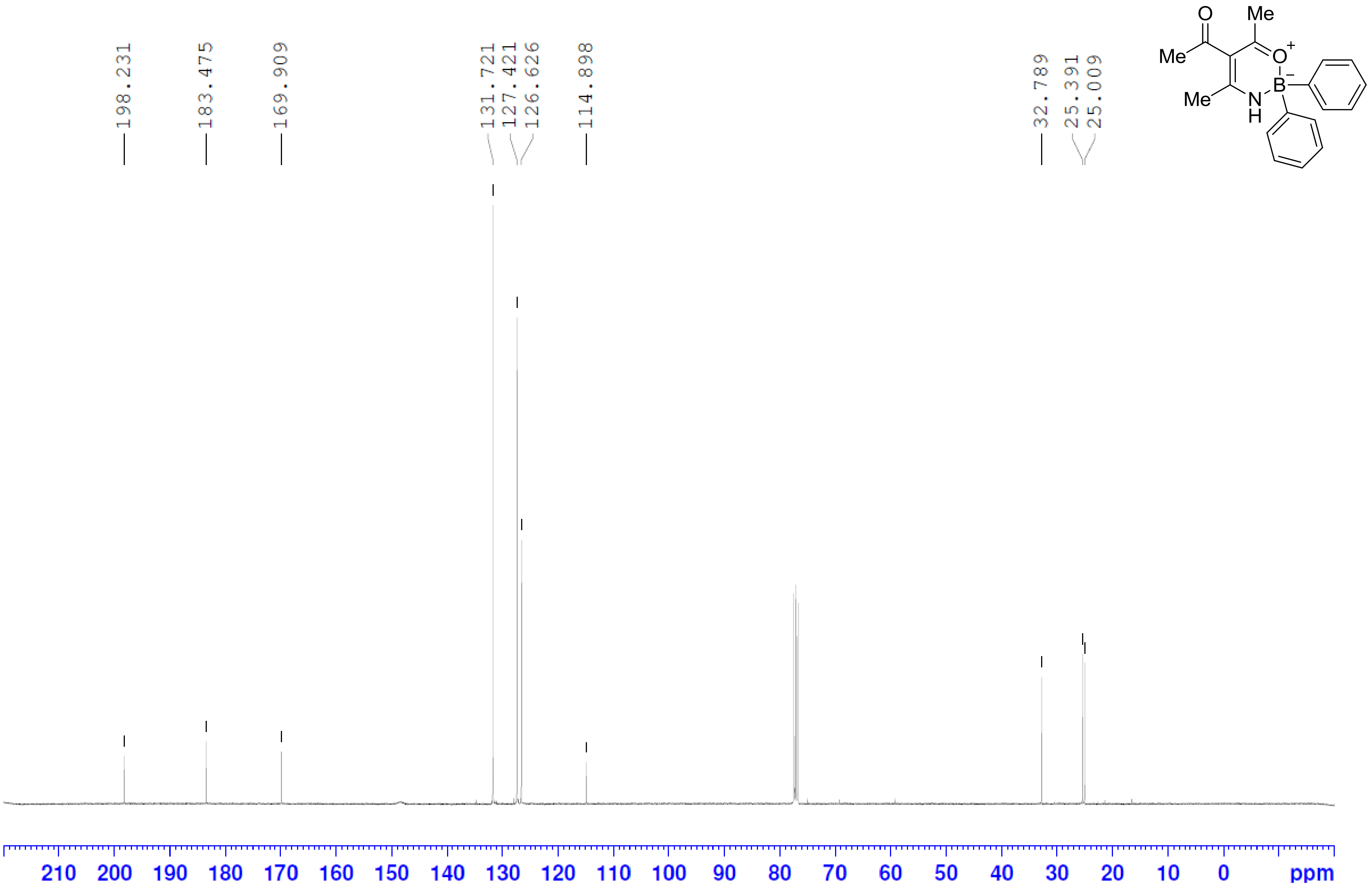

^{13}C NMR spectrum of NBC4 in $CDCl_3$

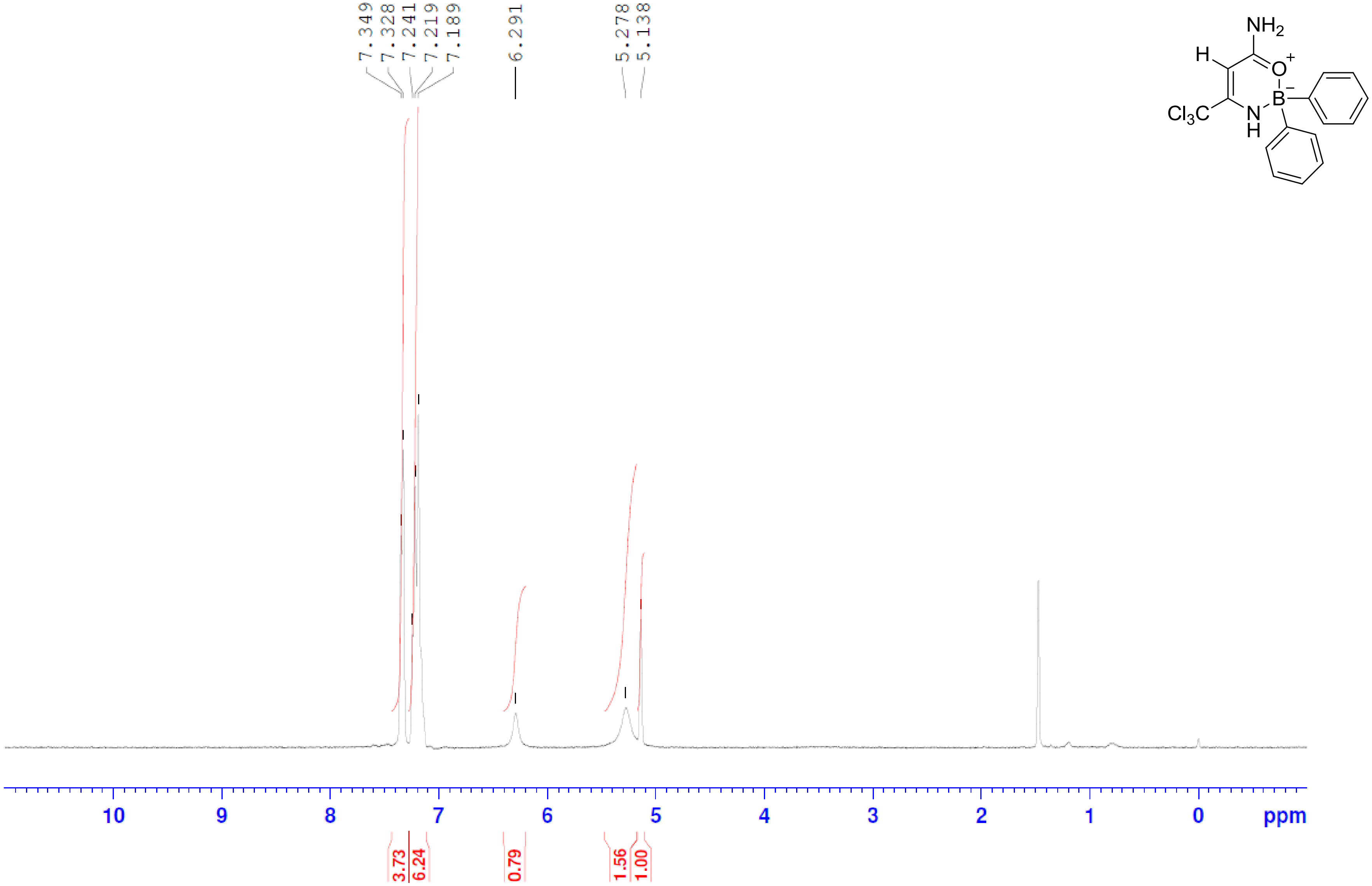

^{1}H NMR spectrum of NBC5 in $CDCl_3$

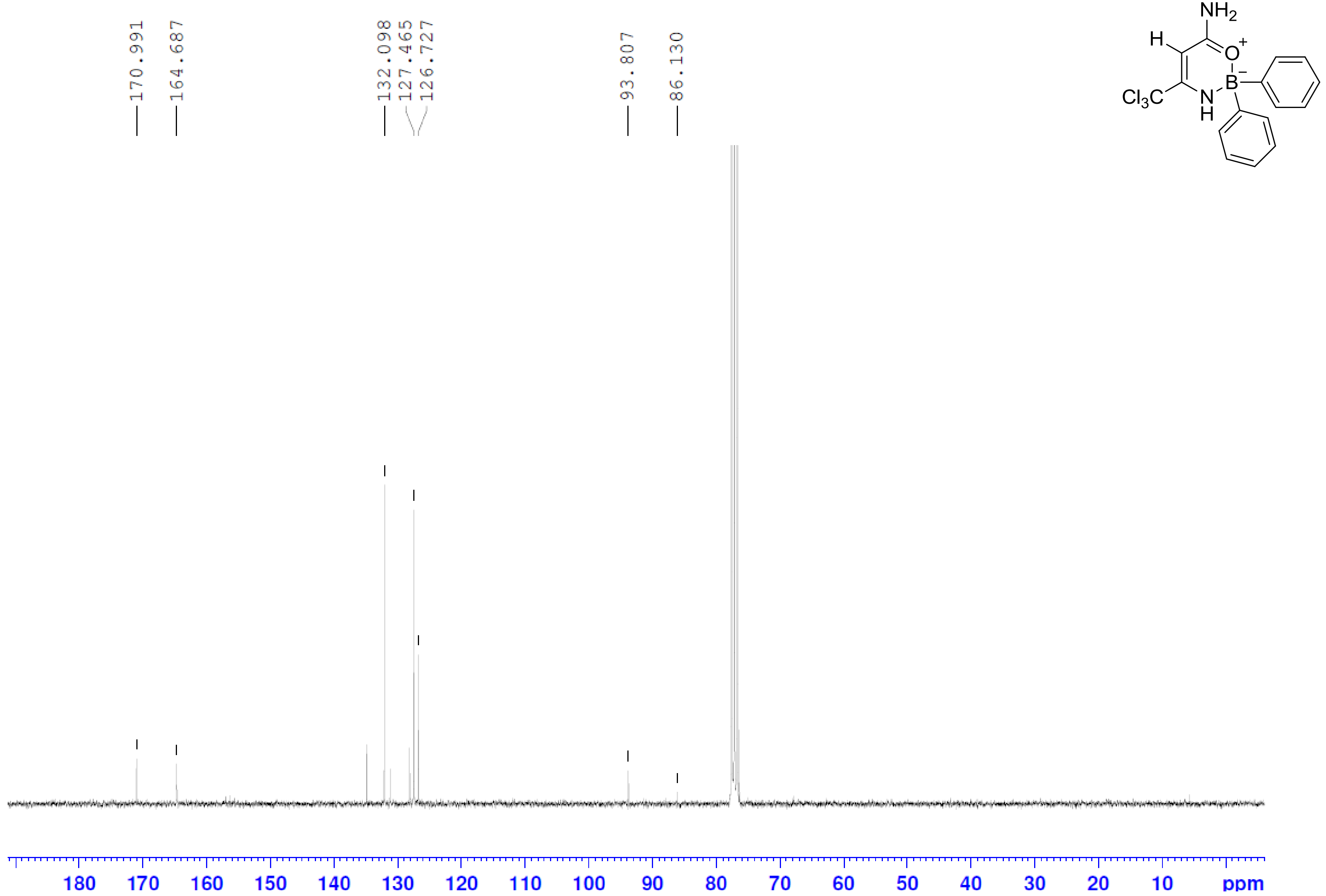

^{13}C NMR spectrum of NBC5 in $CDCl_3$ (3 x small singlet peaks ~ 127-135 ppm due to formation of BPh_2OH hydrolysis product)

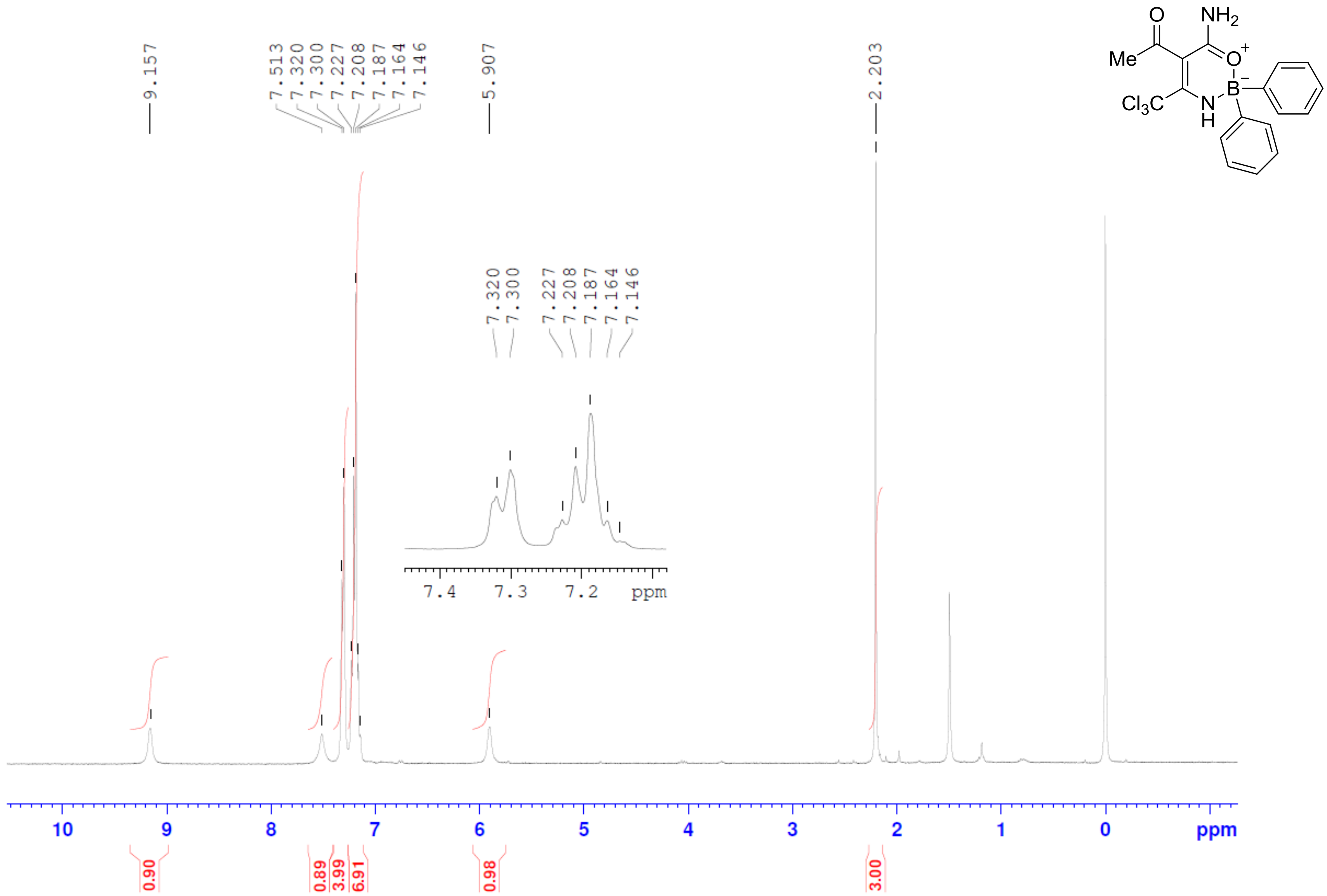

^{1}H NMR spectrum of NBC6 (**15**) in $CDCl_3$

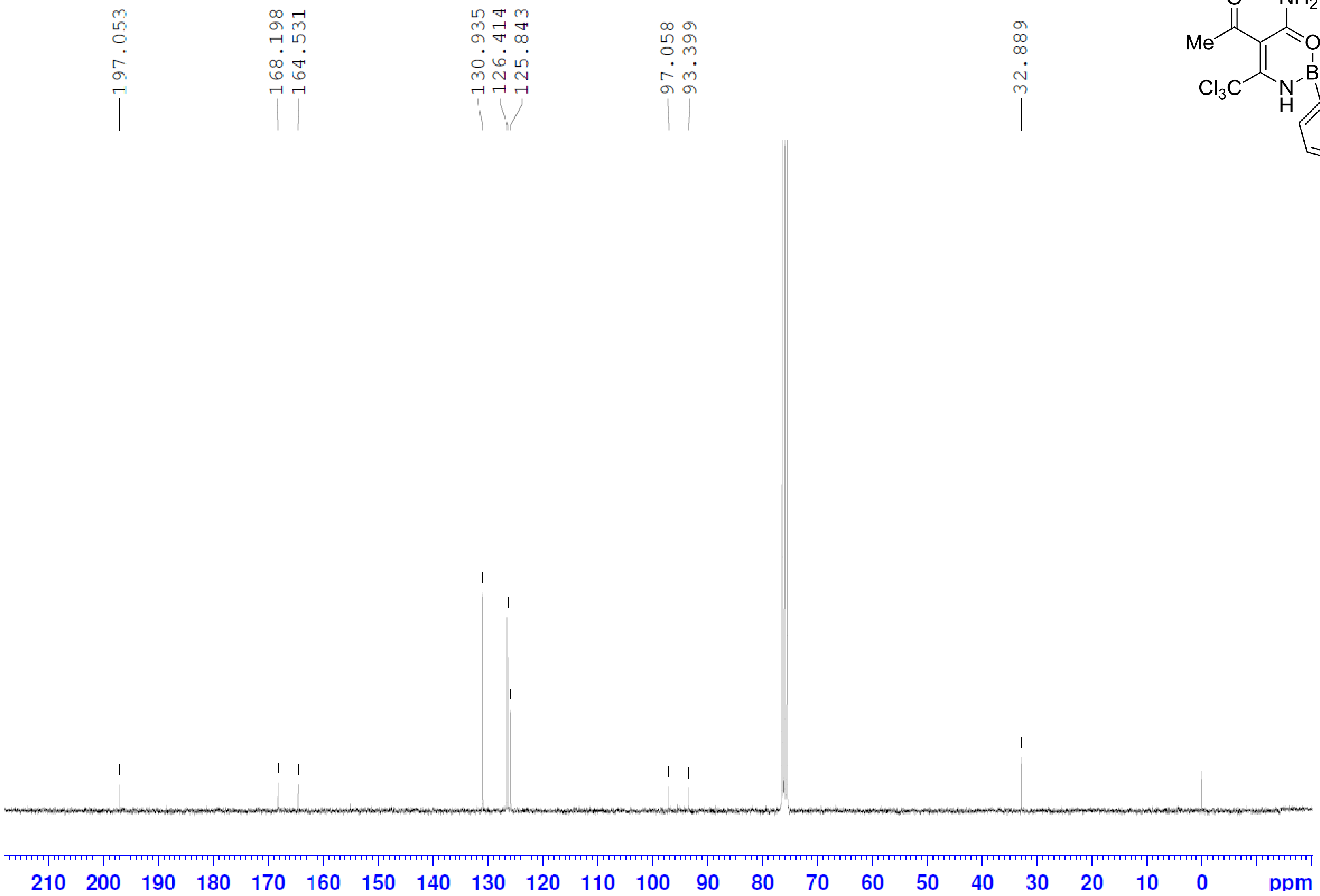

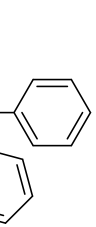

^{13}C NMR spectrum of NBC6 (**15**) in $CDCl_3$

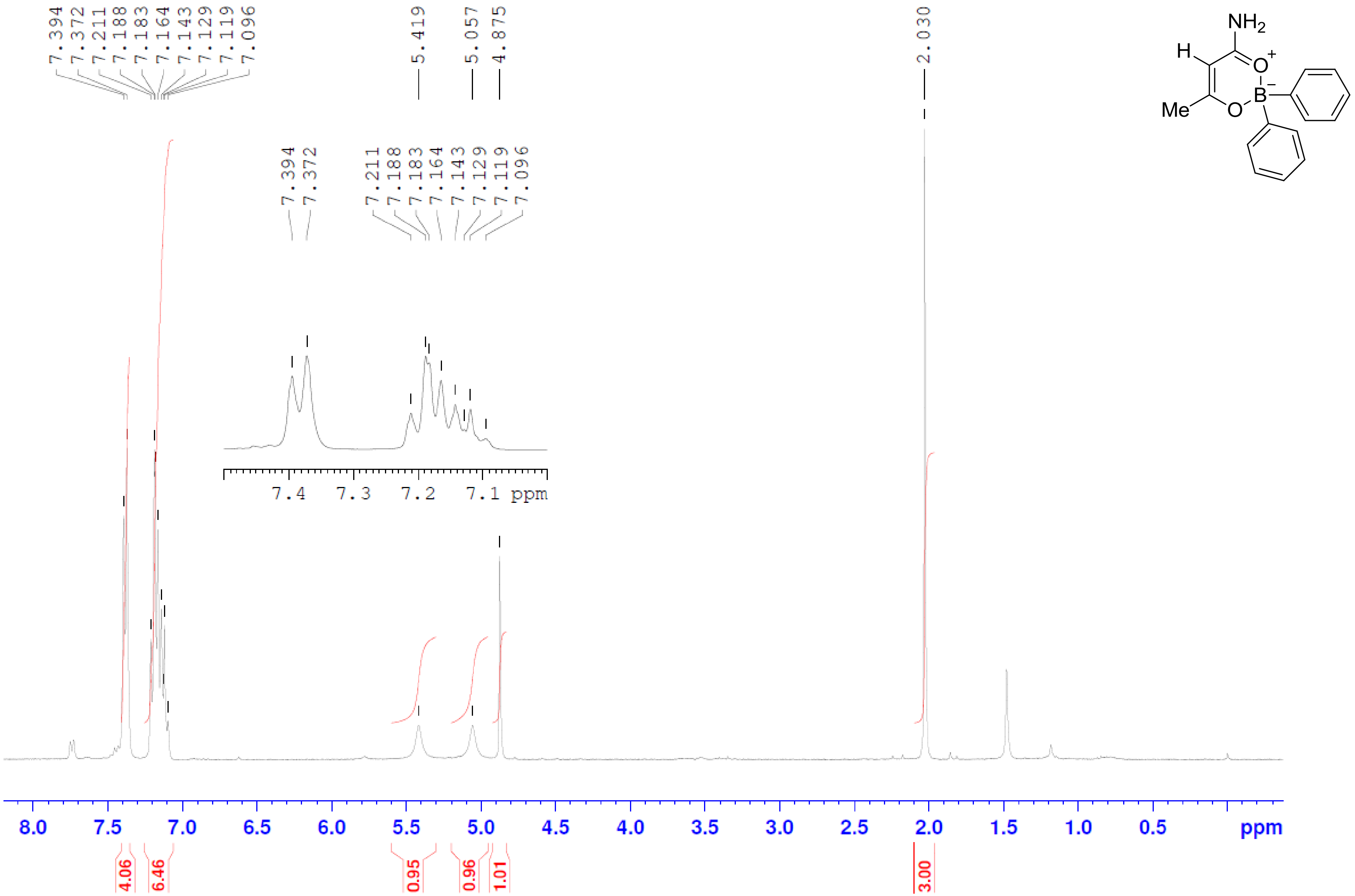

^{1}H NMR spectrum of NBC7 in $CDCl_3$

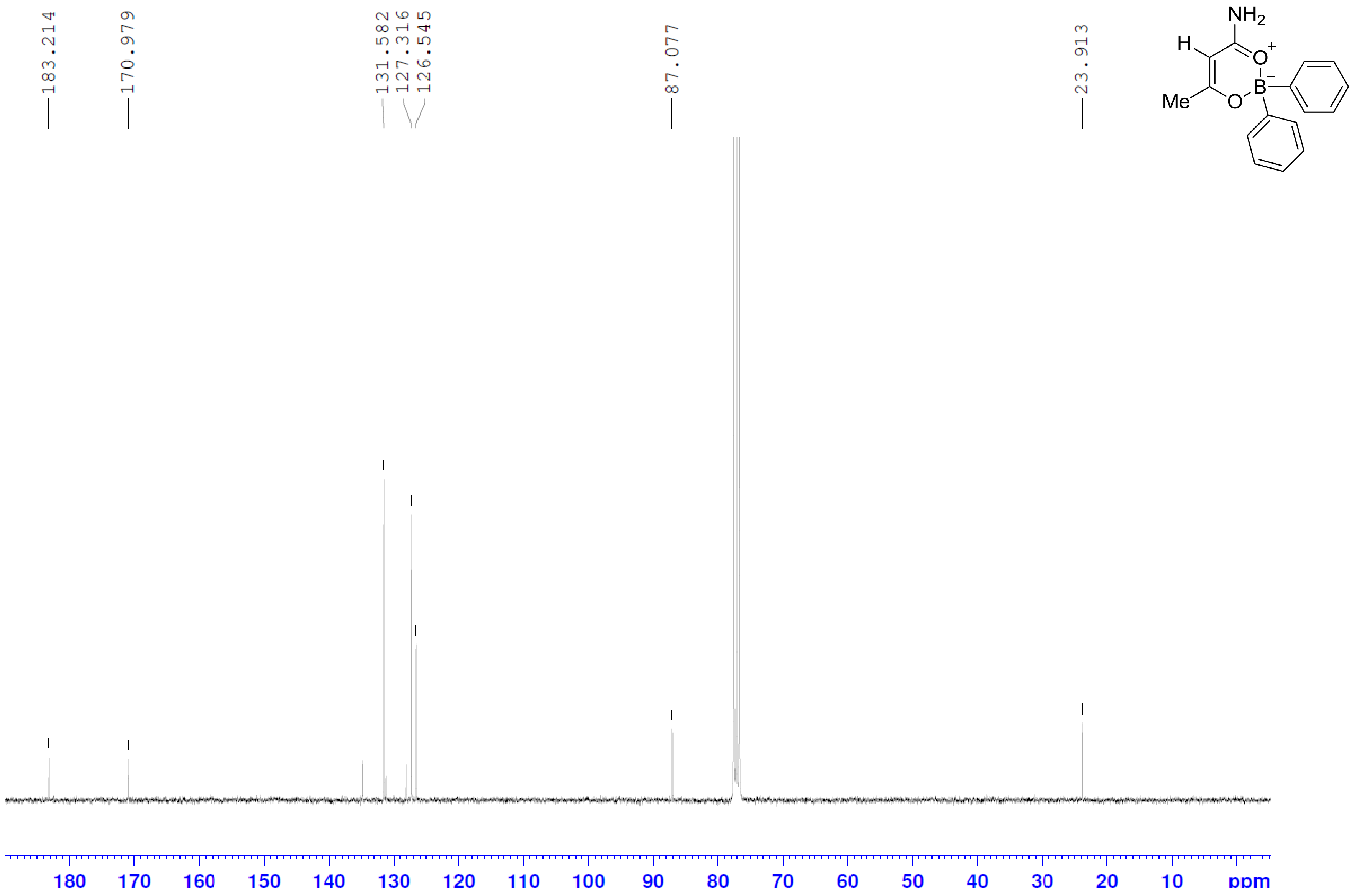

^{13}C NMR spectrum of NBC7 in $CDCl_3$

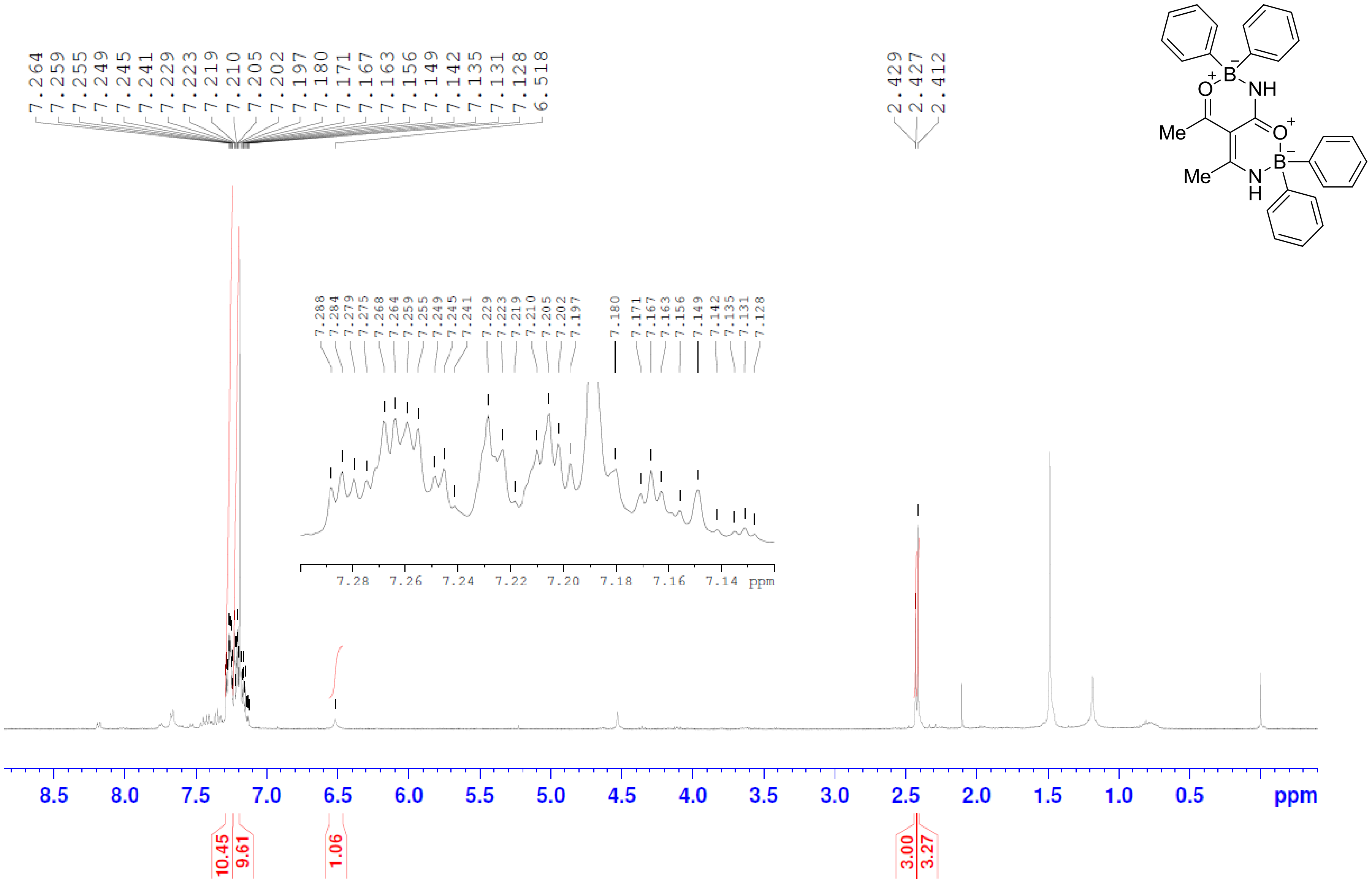

^{1}H NMR spectrum of NBC8 in $CDCl_3$

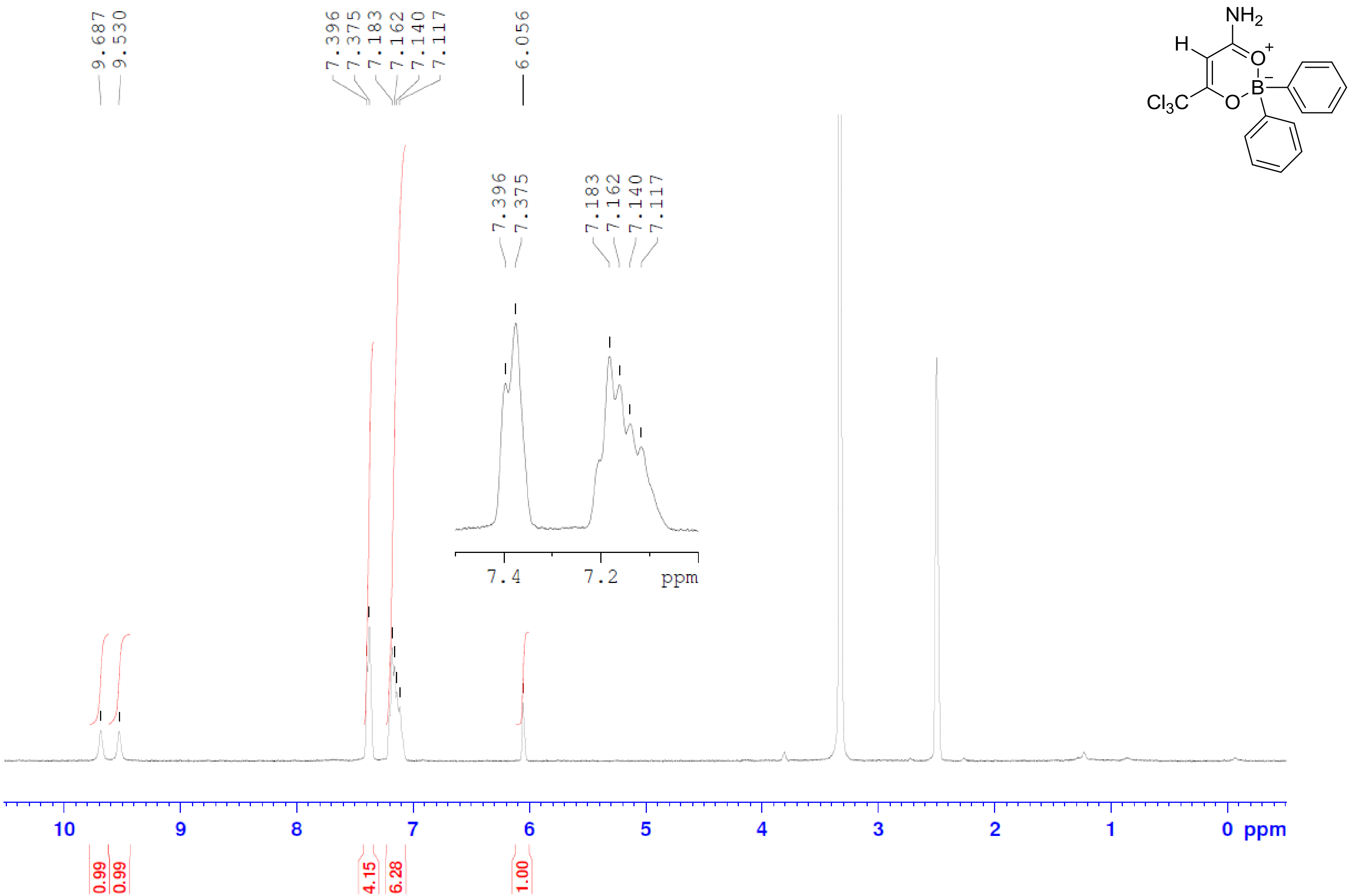

^{1}H NMR spectrum of NBC9 in DMSO-d_6

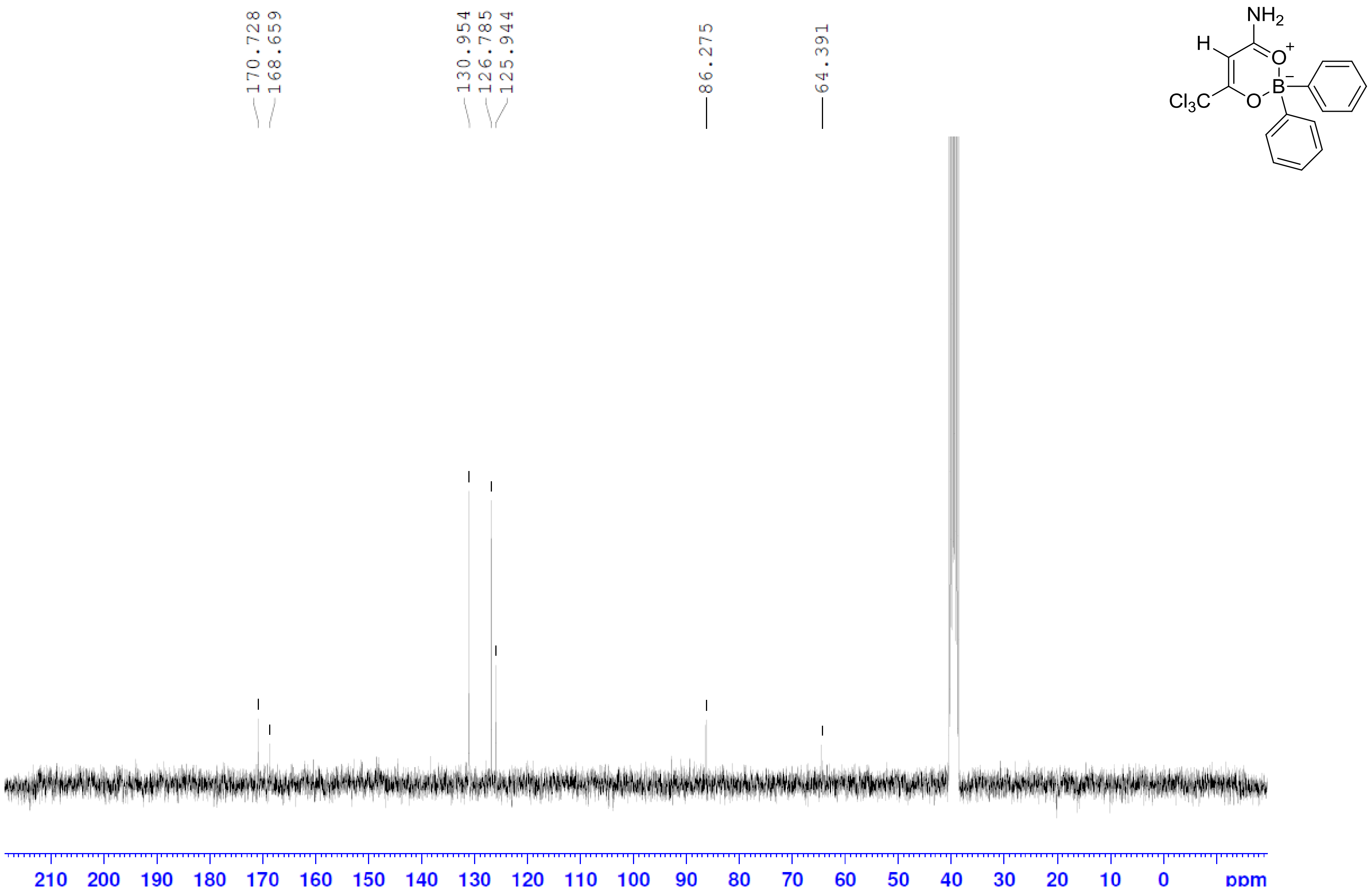

^{13}C NMR spectrum of NBC9 in DMSO-d_6

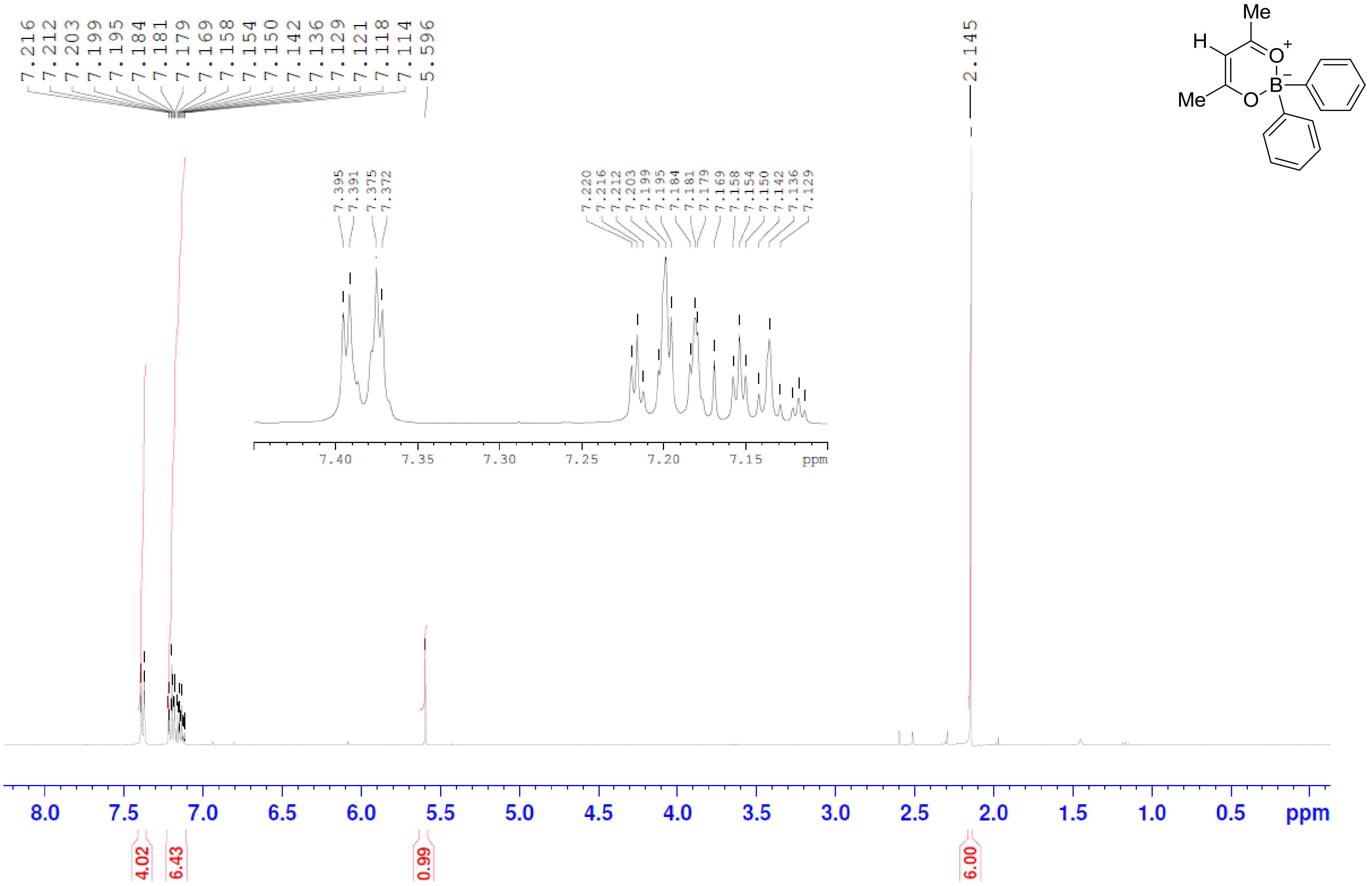

^{1}H NMR spectrum of NBC10 in $CDCl_3$

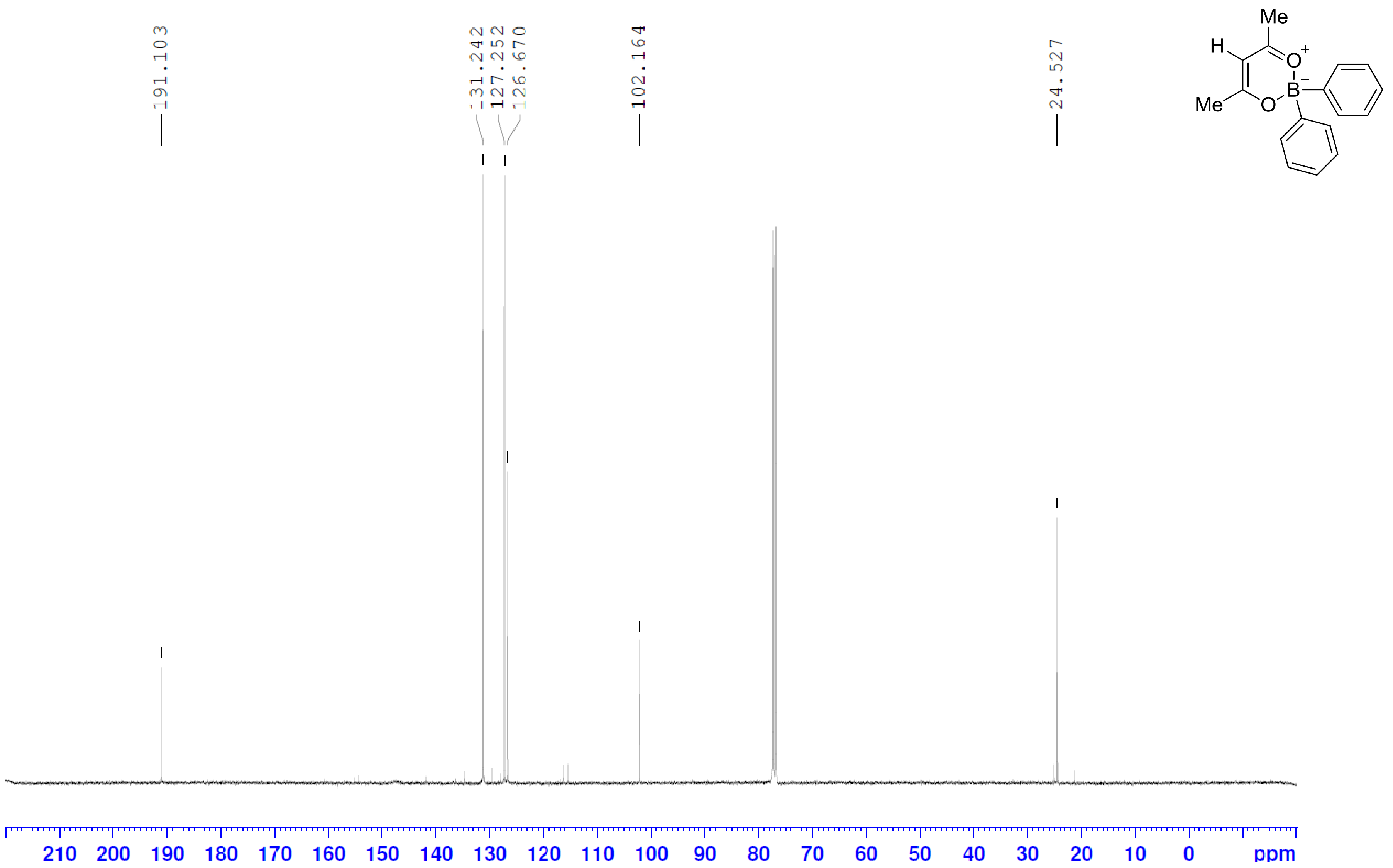

^{13}C NMR spectrum of NBC10 in $CDCl_3$

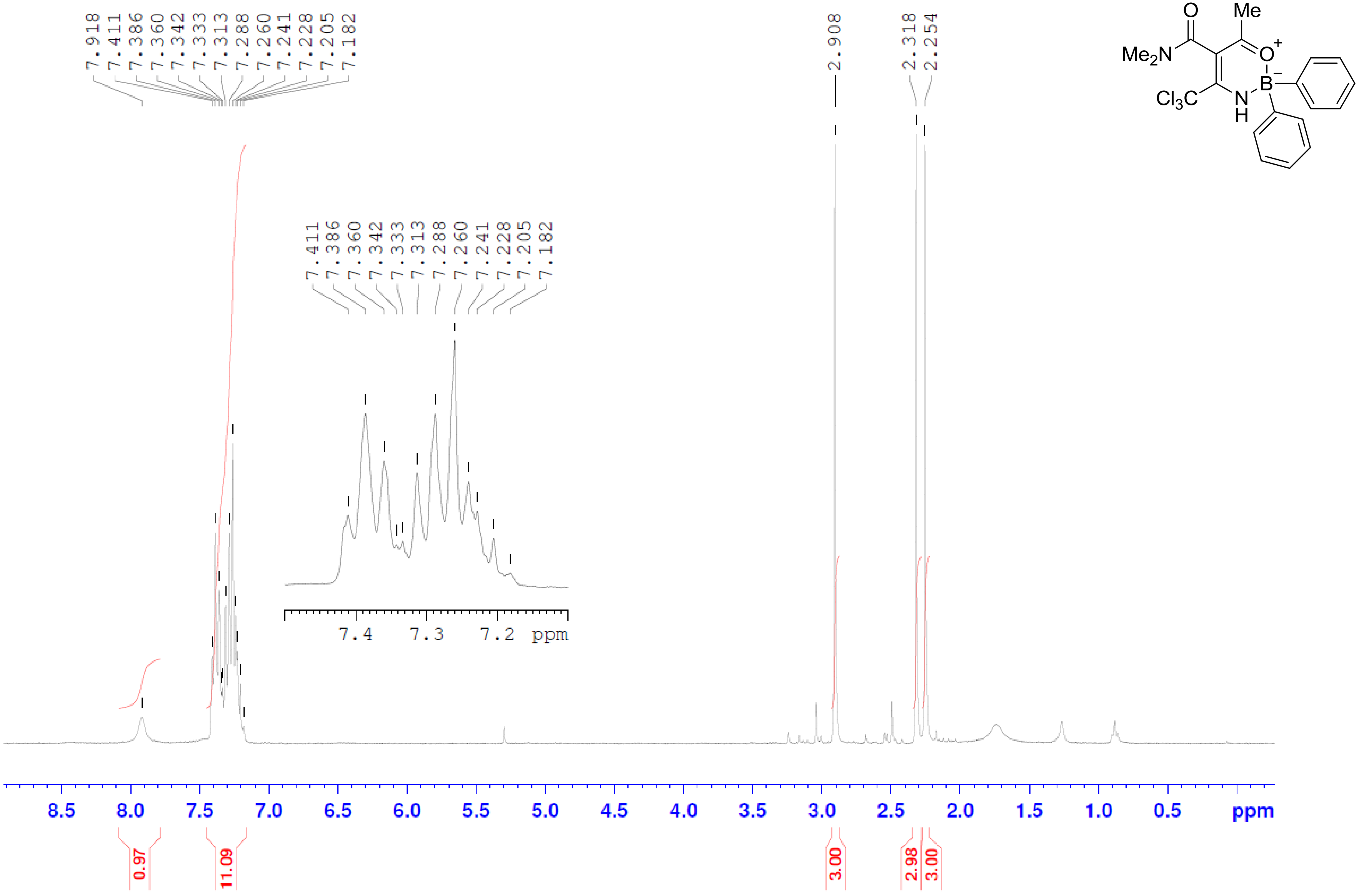

^{1}H NMR spectrum of NBC11 (**16**) in $CDCl_3$

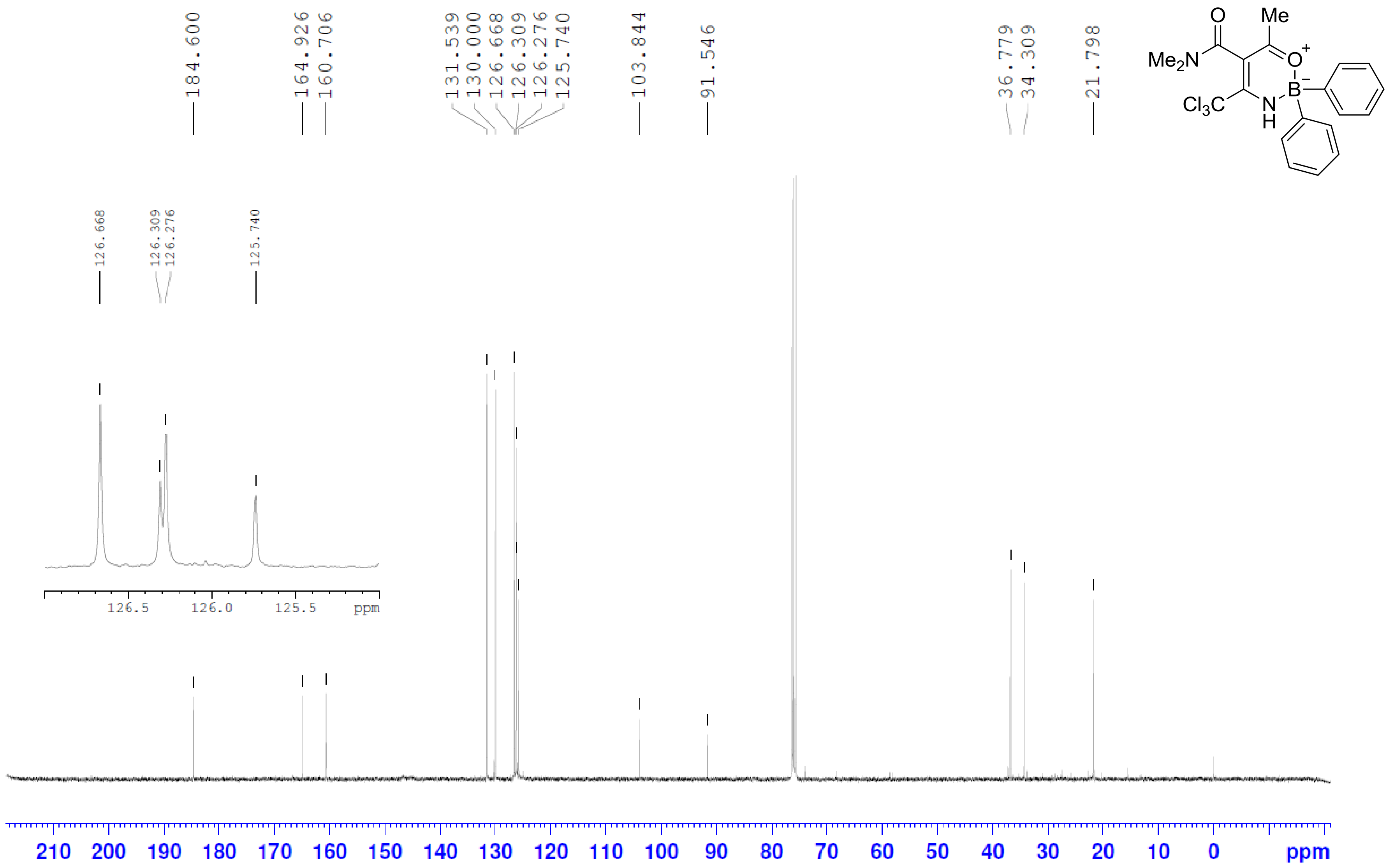

^{13}C NMR spectrum of NBC11 (**16**) in $CDCl_3$

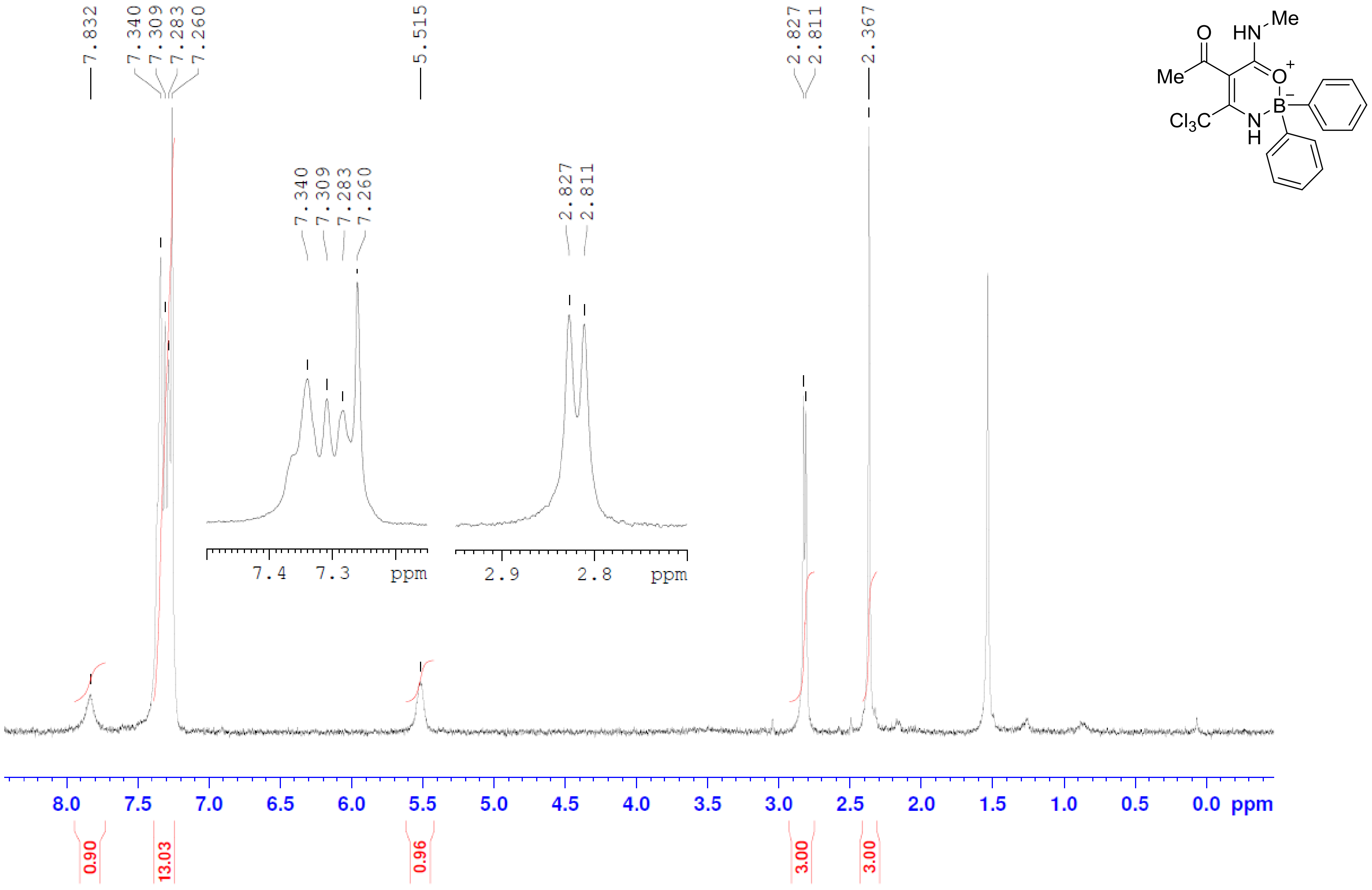

^{1}H NMR spectrum of NBC12 in $CDCl_3$

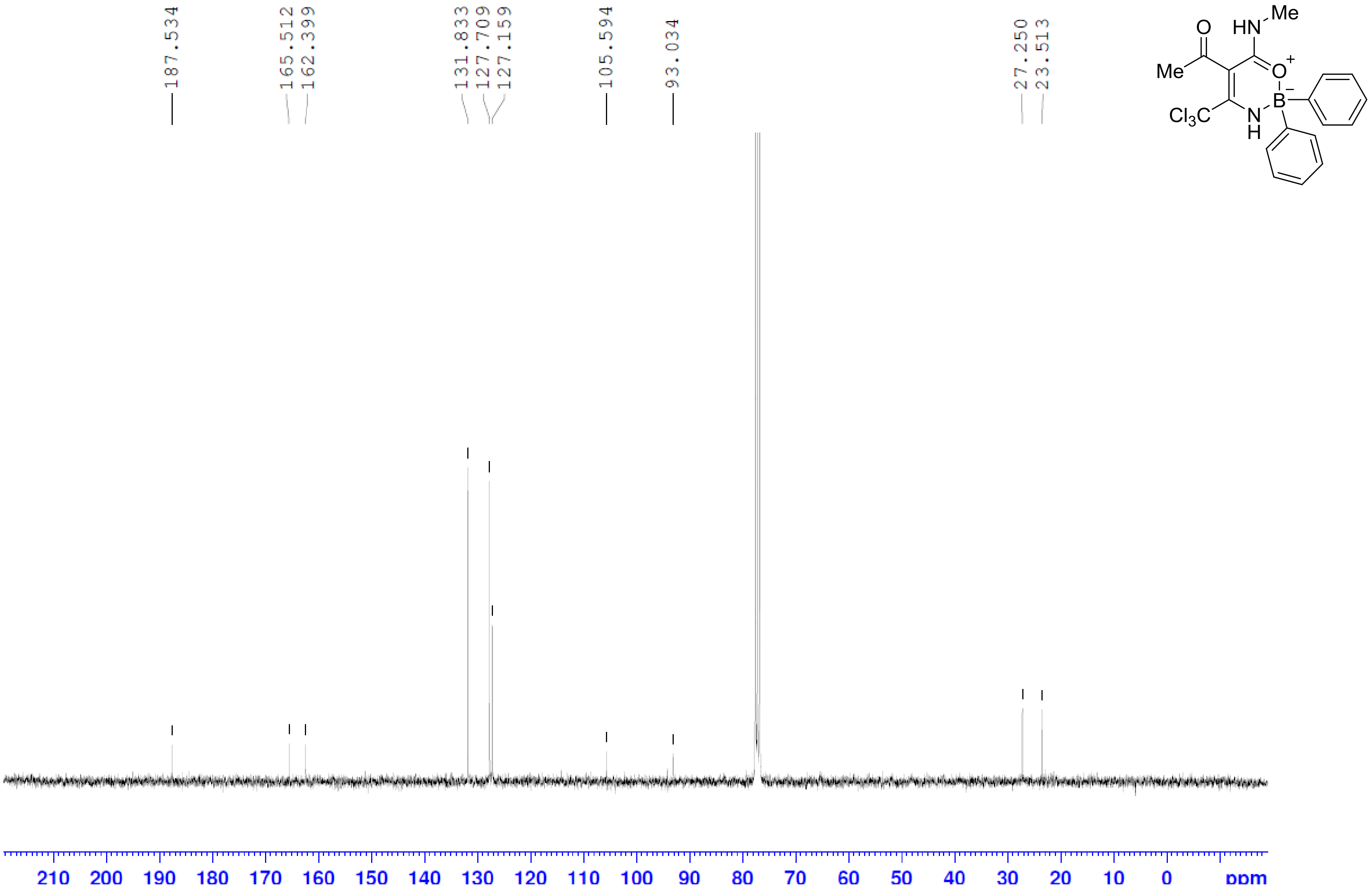

^{13}C NMR spectrum of NBC12 in $CDCl_3$

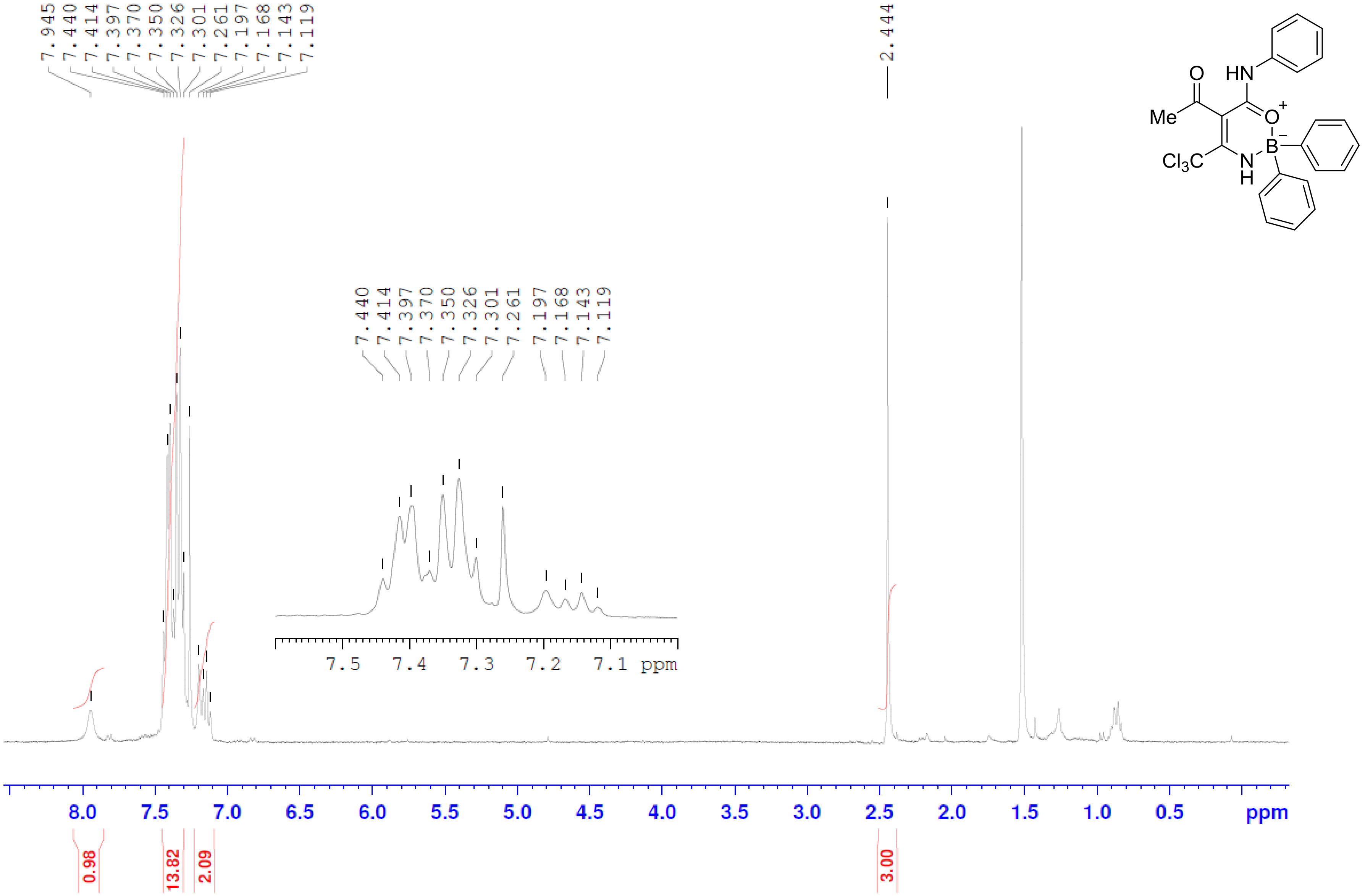

^{1}H NMR spectrum of NBC13 in $CDCl_3$

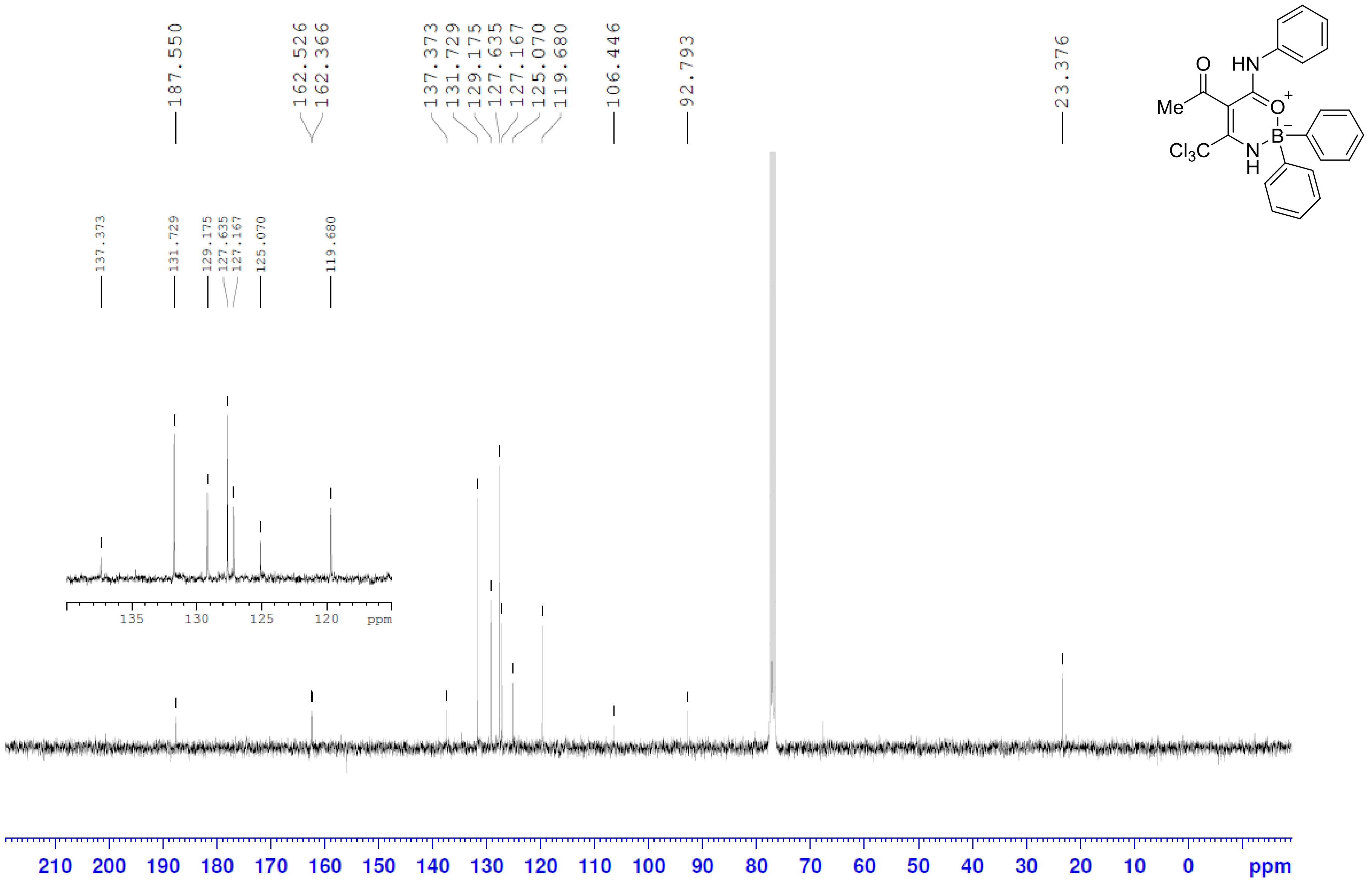

^{13}C NMR spectrum of NBC13 in $CDCl_3$

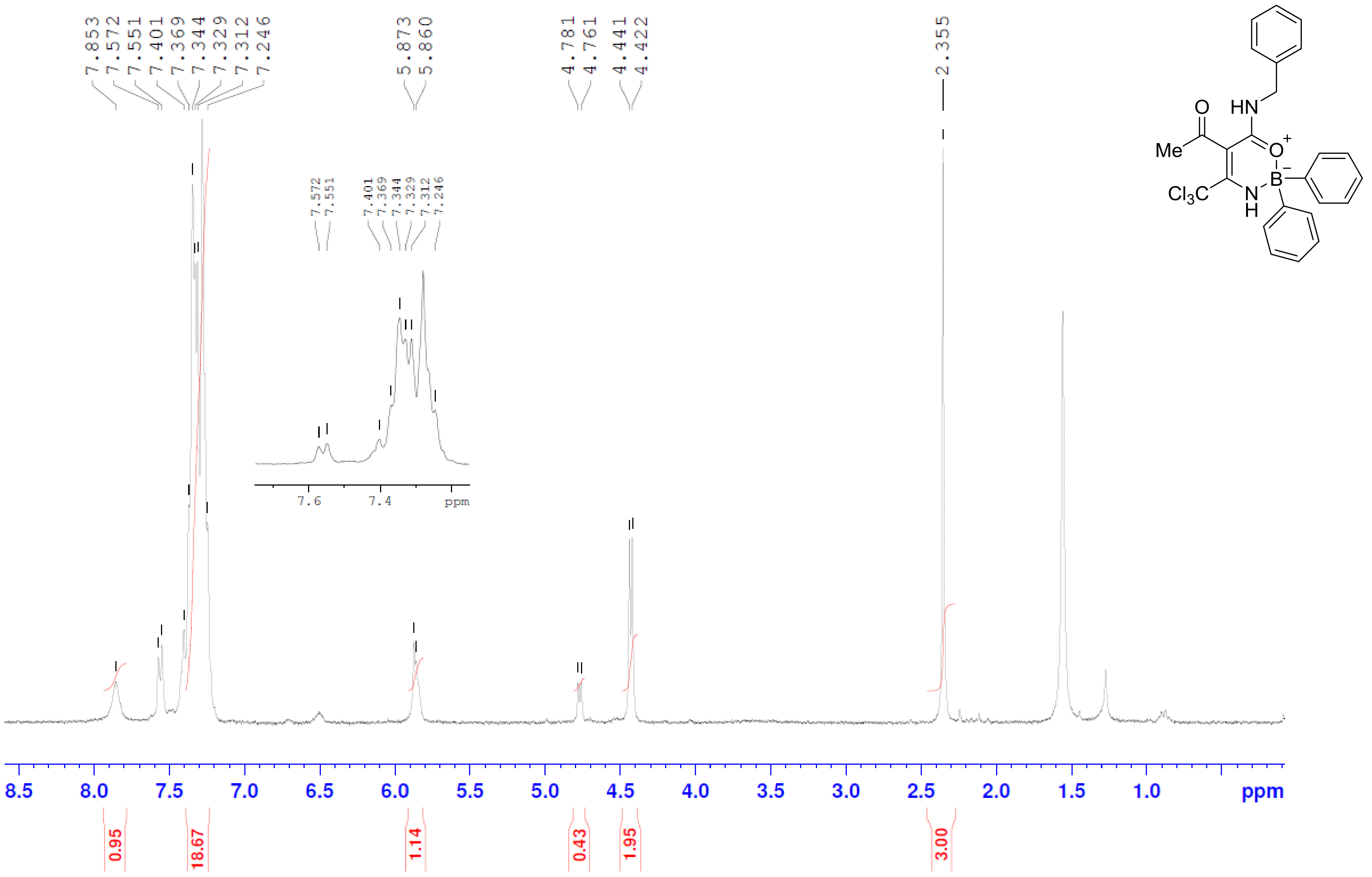

^{1}H NMR spectrum of NBC14 in $CDCl_3$

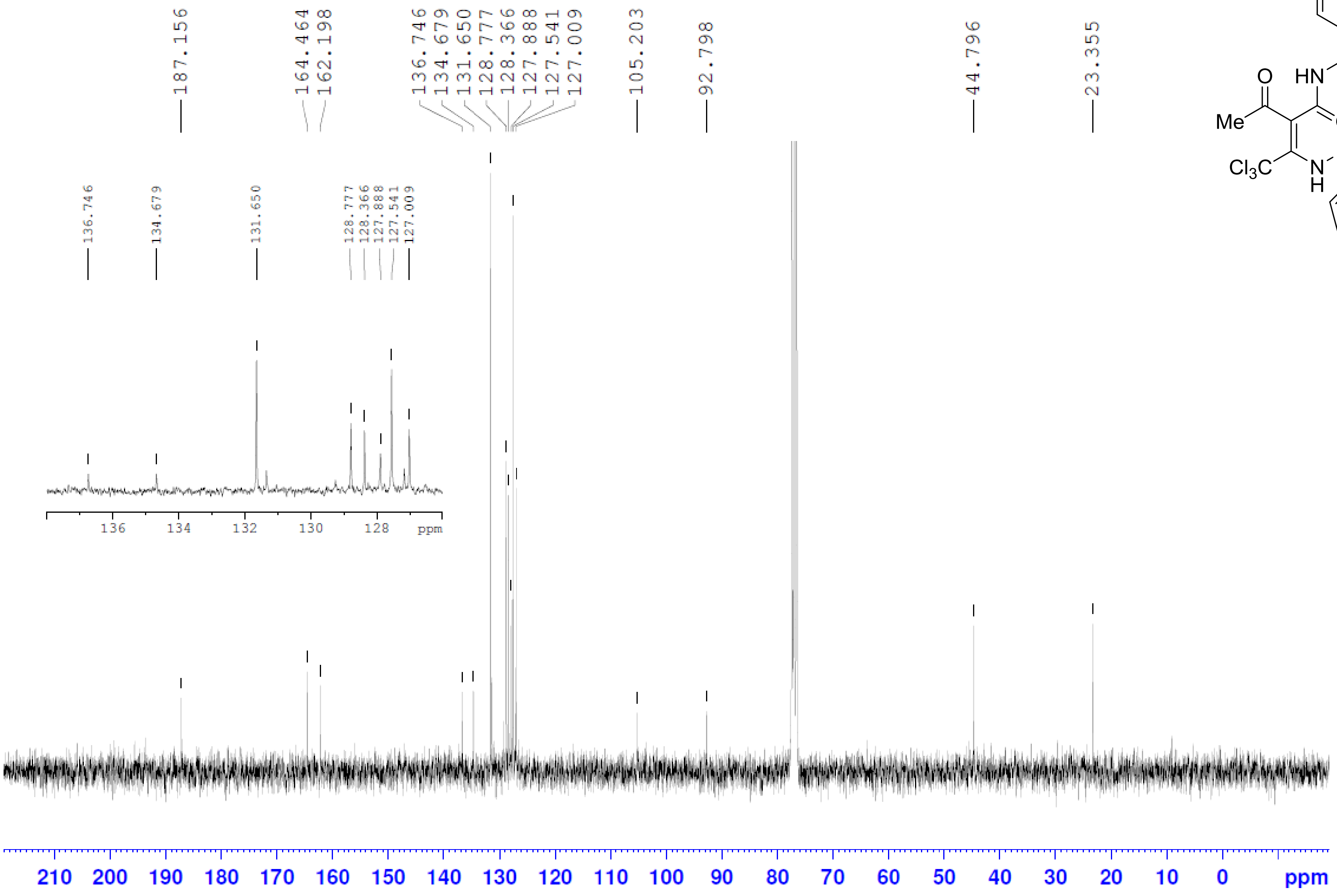

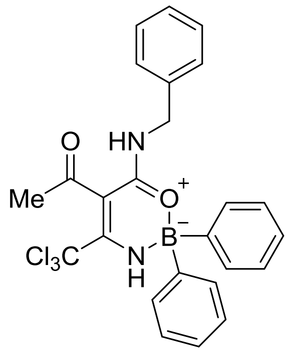

^{13}C NMR spectrum of NBC14 in $CDCl_3$

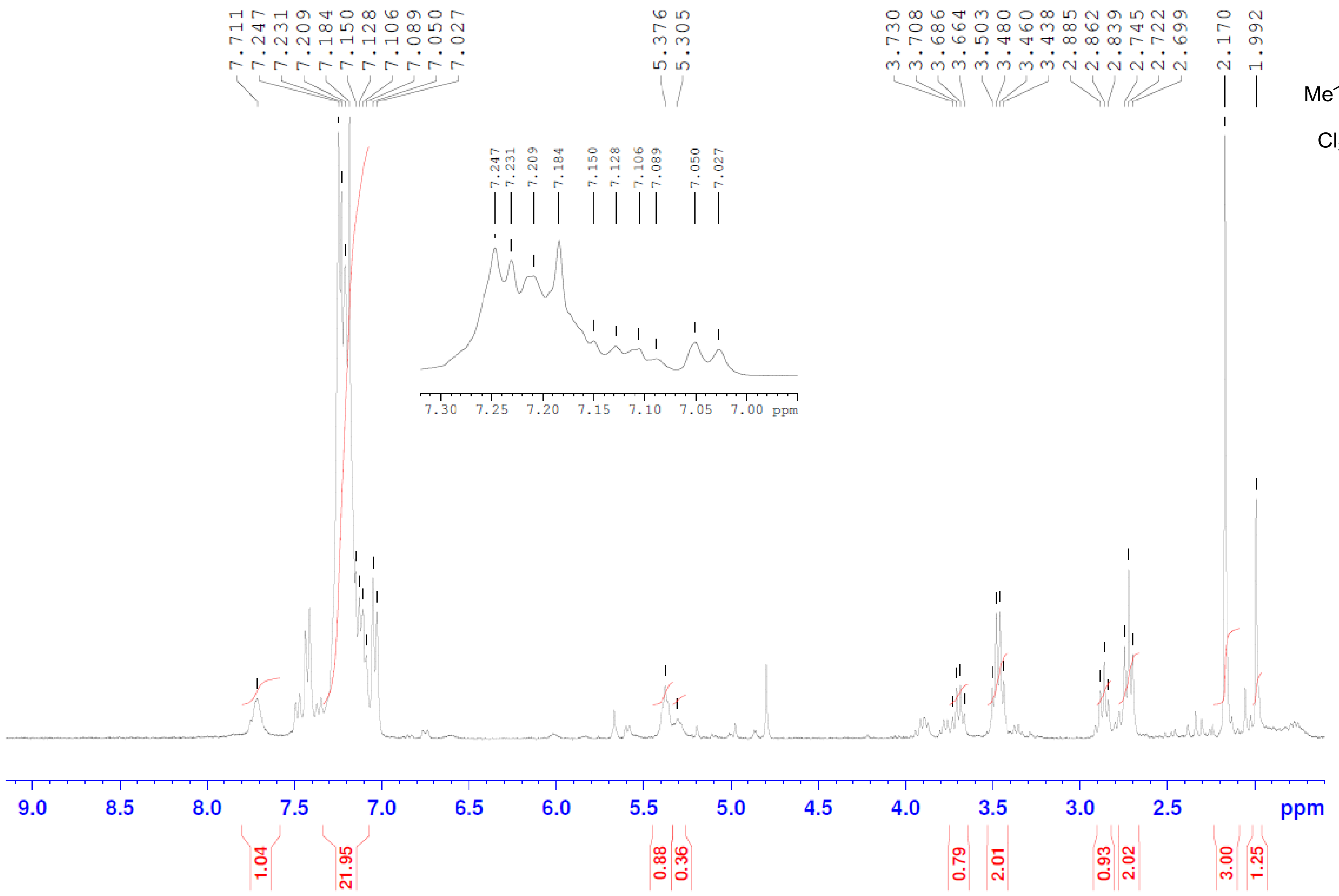

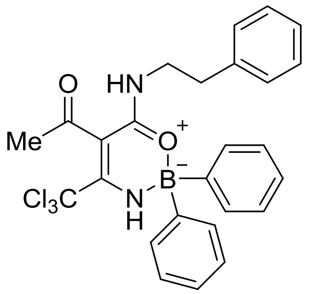

^{1}H NMR spectrum of NBC15 in $CDCl_3$

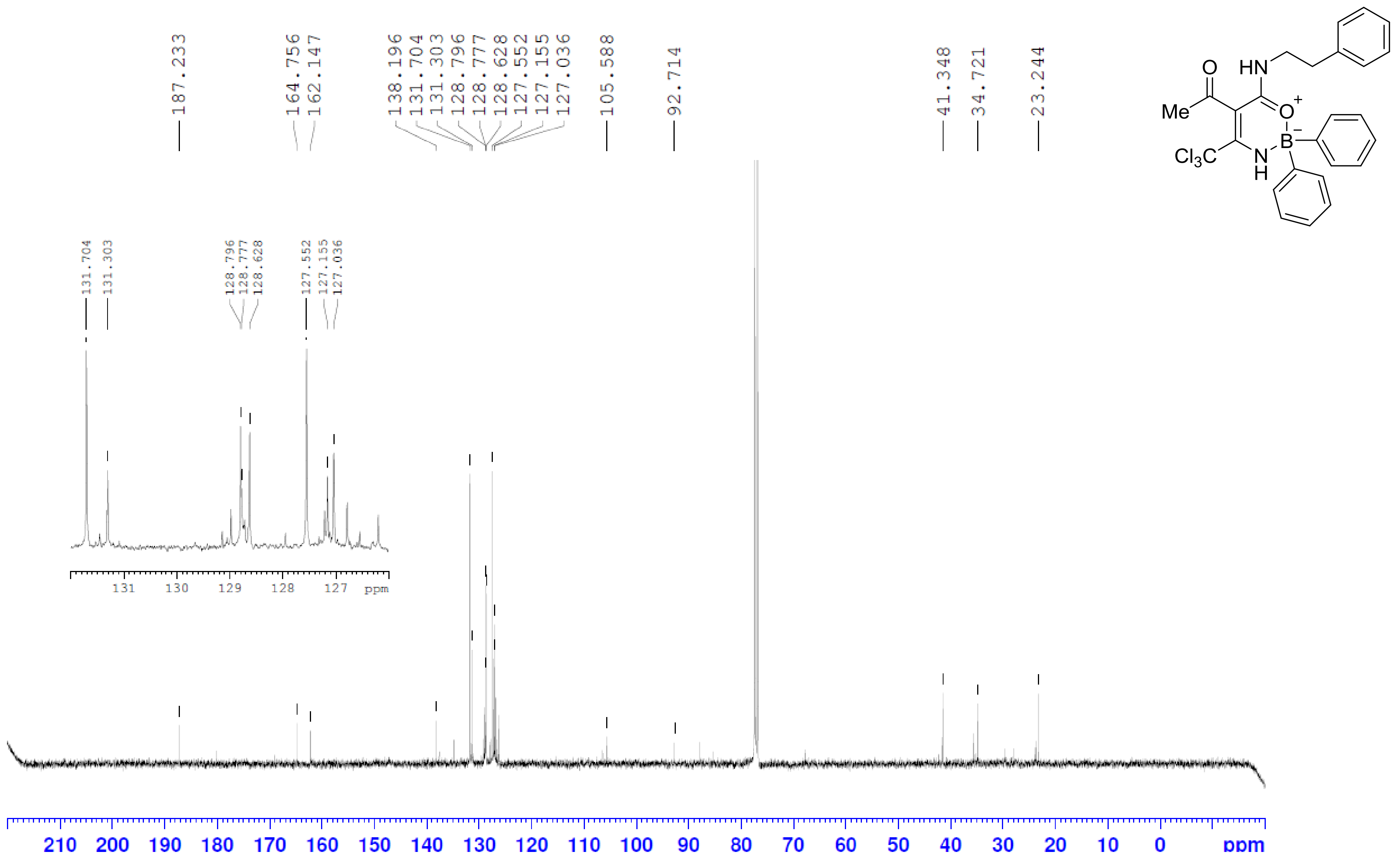

^{13}C NMR spectrum of NBC15 in $CDCl_3$

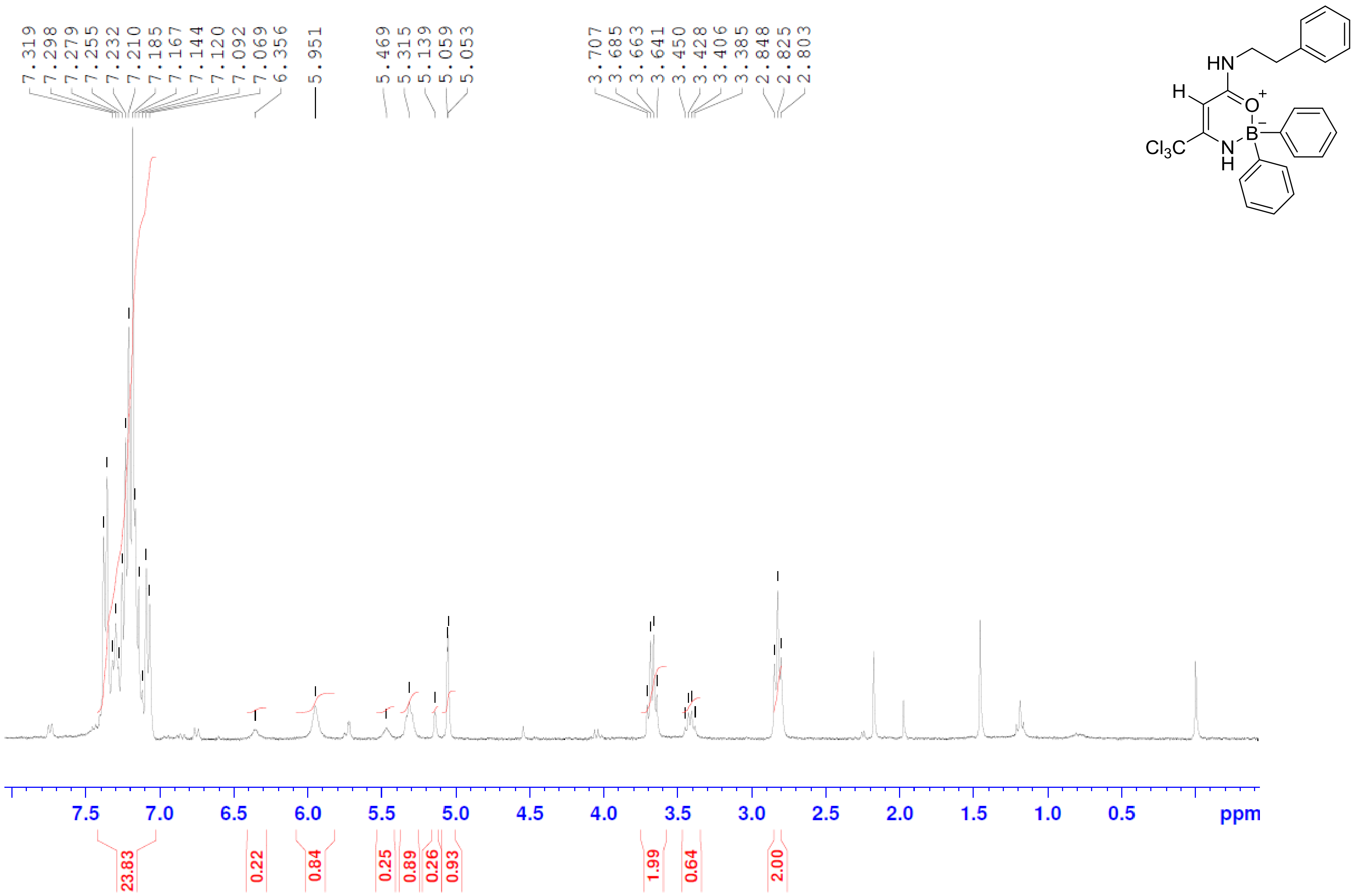

^{1}H NMR spectrum of NBC16 in $CDCl_3$

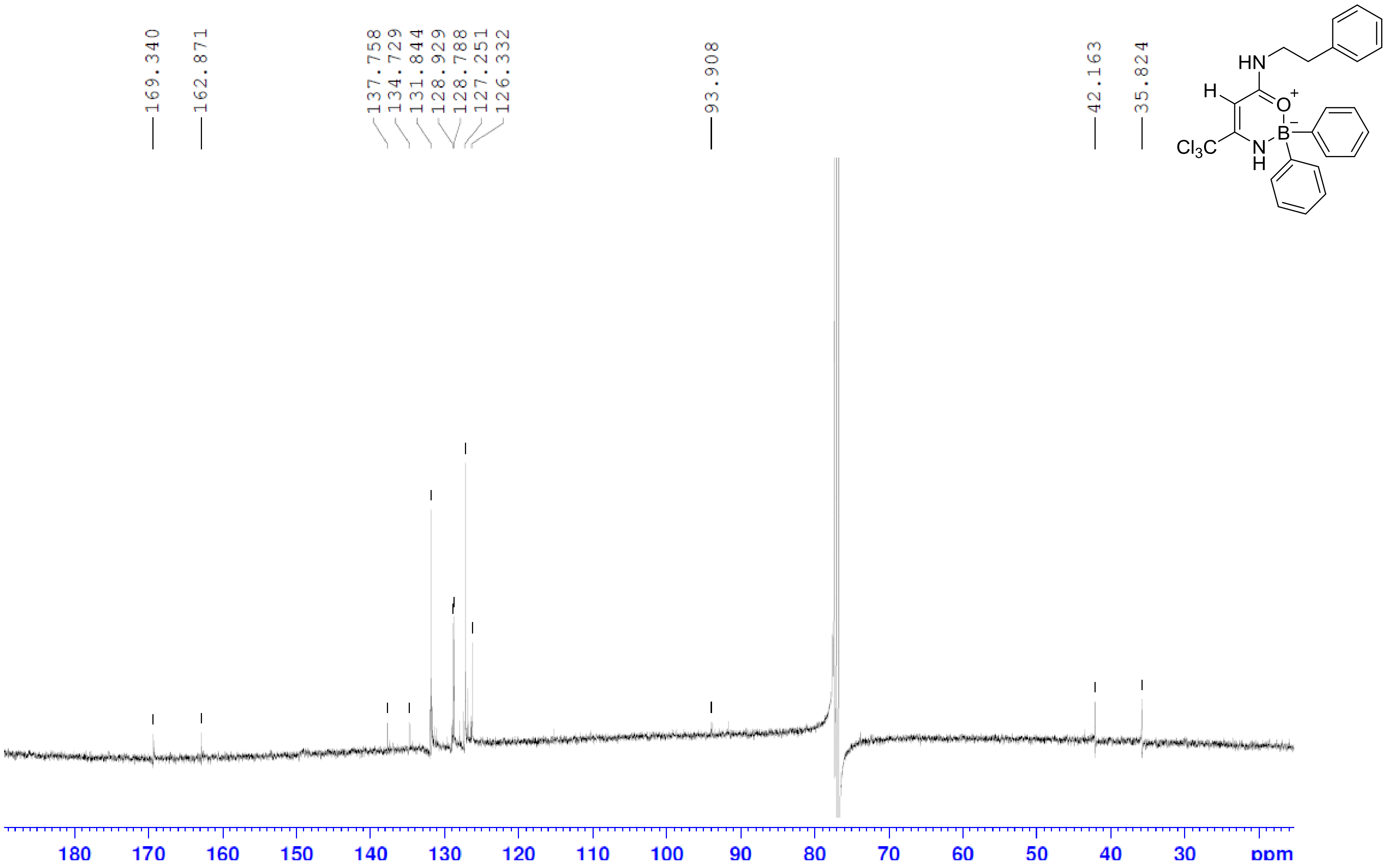

^{13}C NMR spectrum of NBC16 in $CDCl_3$

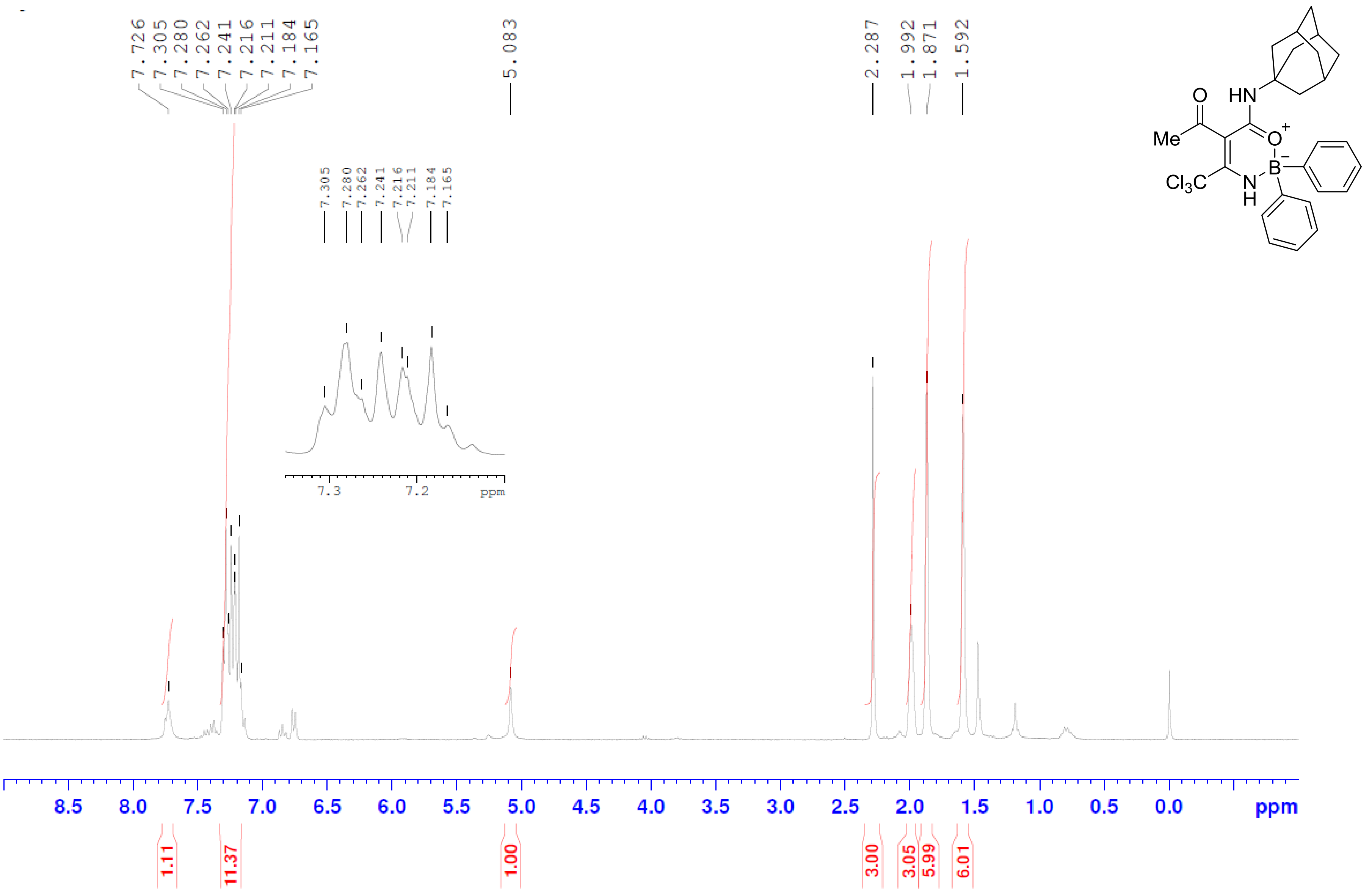

^{1}H NMR spectrum of NBC17 in $CDCl_3$

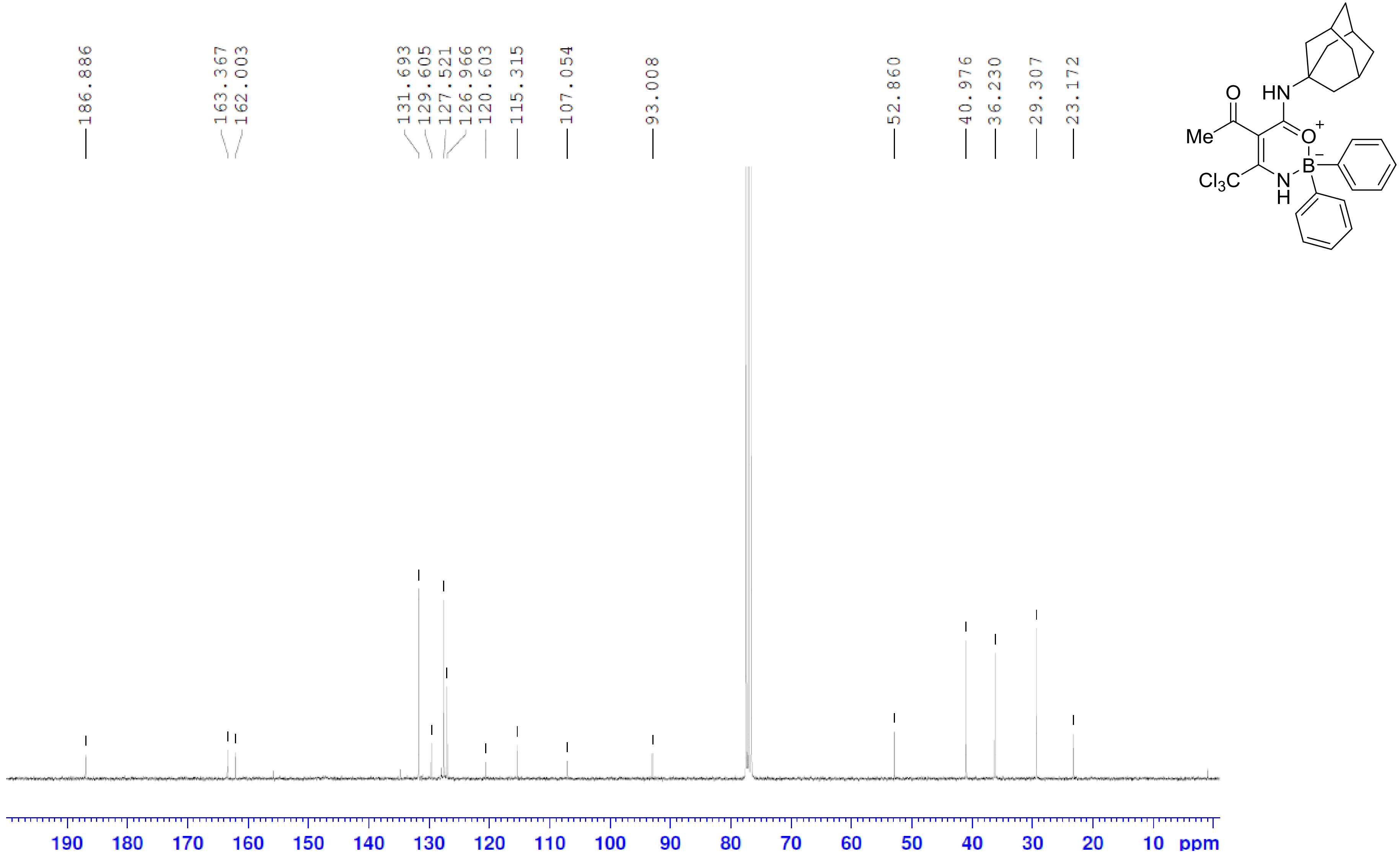

^{13}C NMR spectrum of NBC17 in $CDCl_3$

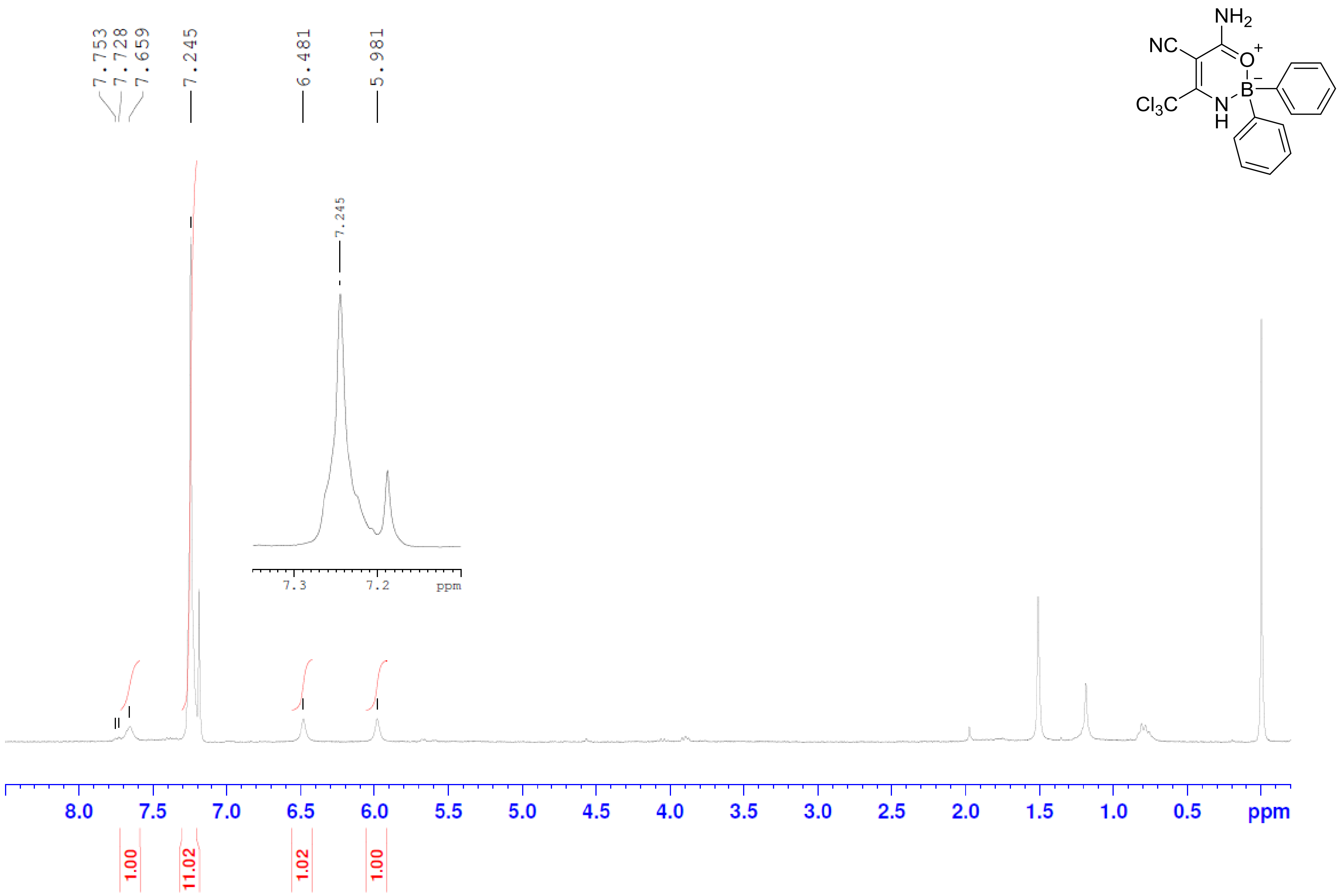

^{1}H NMR spectrum of NBC18 in $CDCl_3$

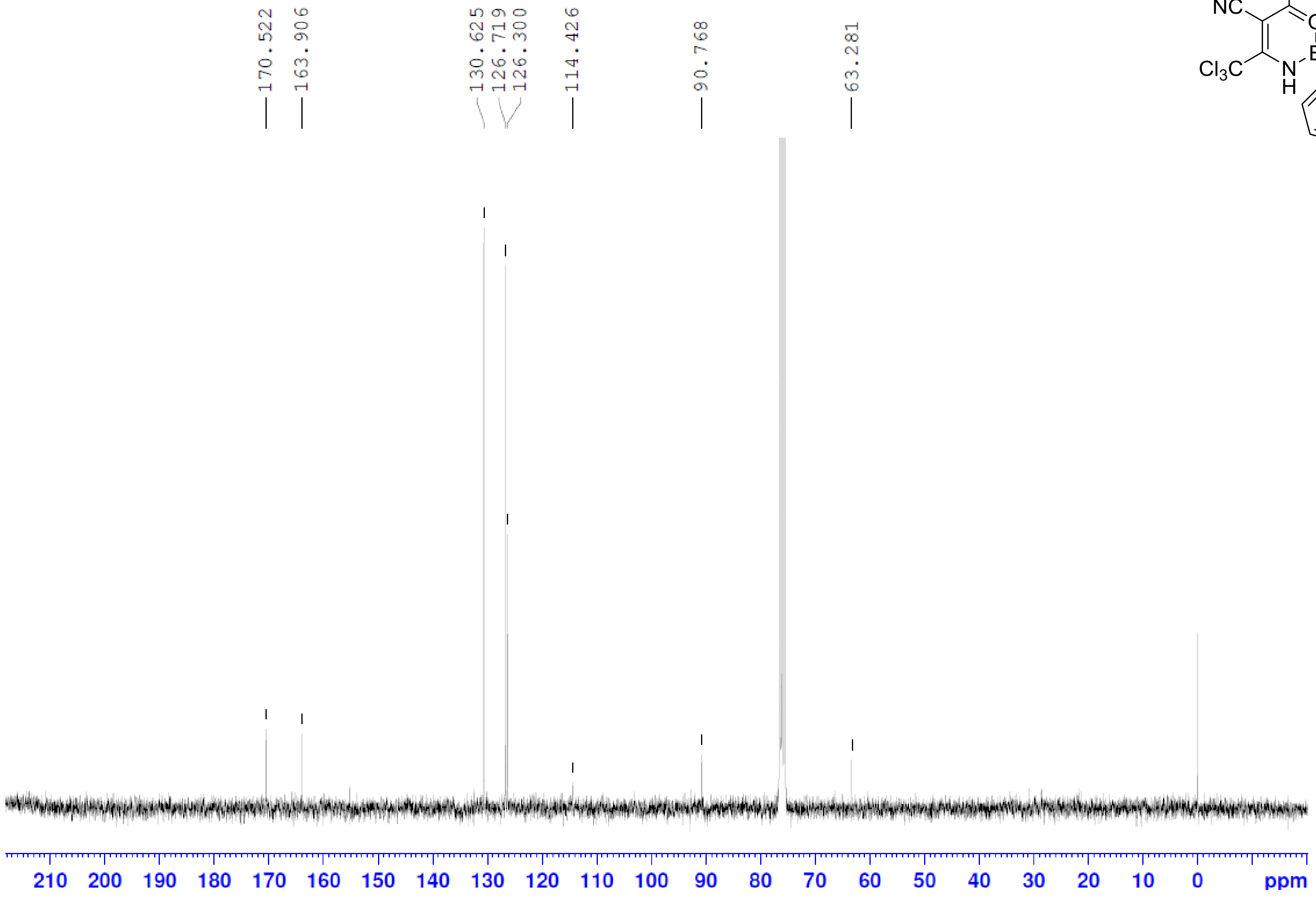

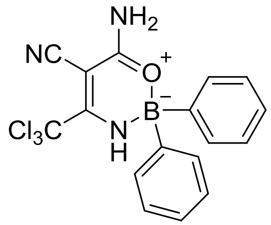

^{13}C NMR spectrum of NBC18 in $CDCl_3$

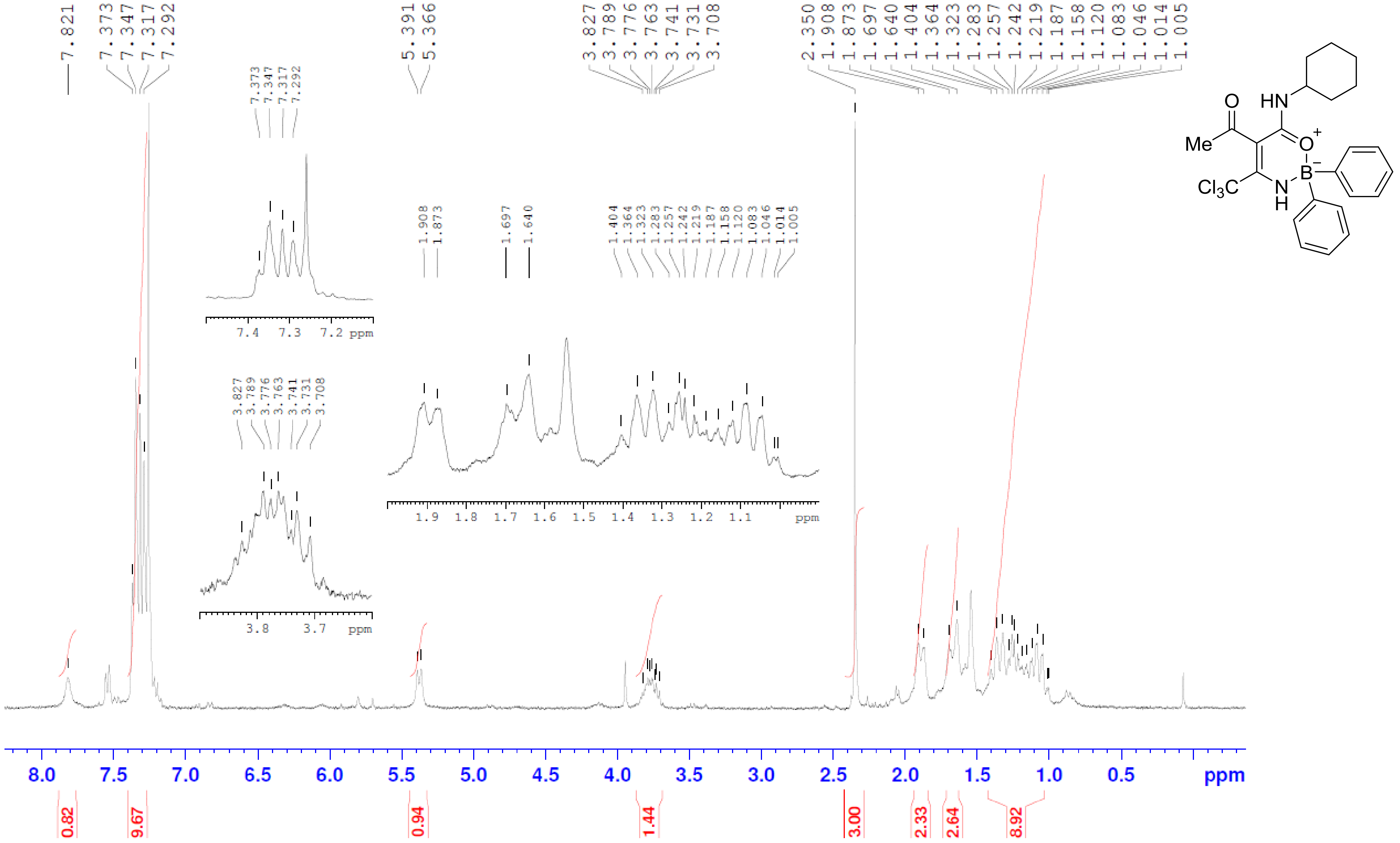

^{1}H NMR spectrum of NBC19 (**17**) in $CDCl_3$

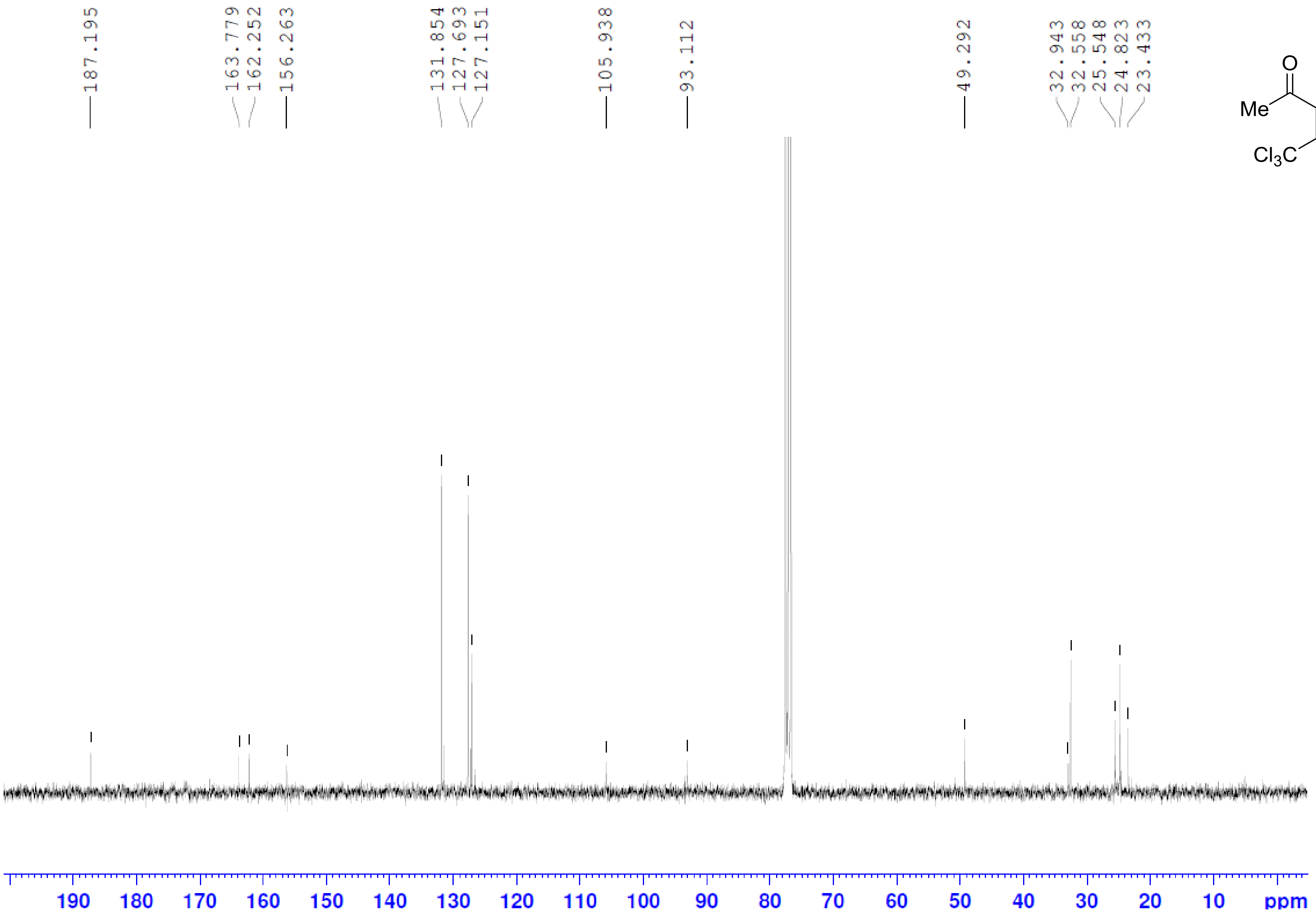

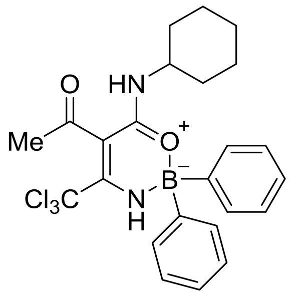

^{13}C NMR spectrum of NBC19 (**17**) in $CDCl_3$

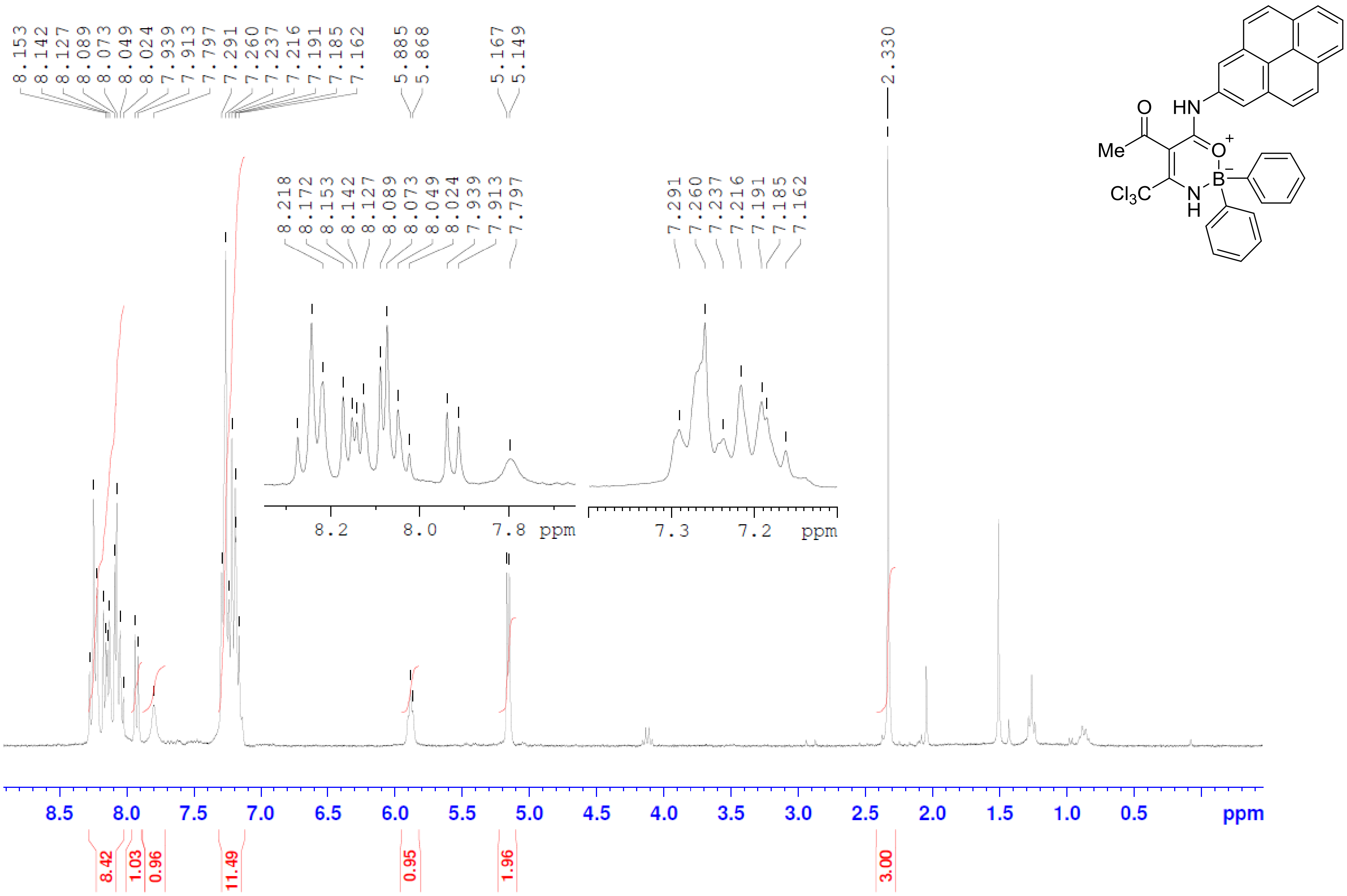

^{1}H NMR spectrum of NBC20 in $CDCl_3$

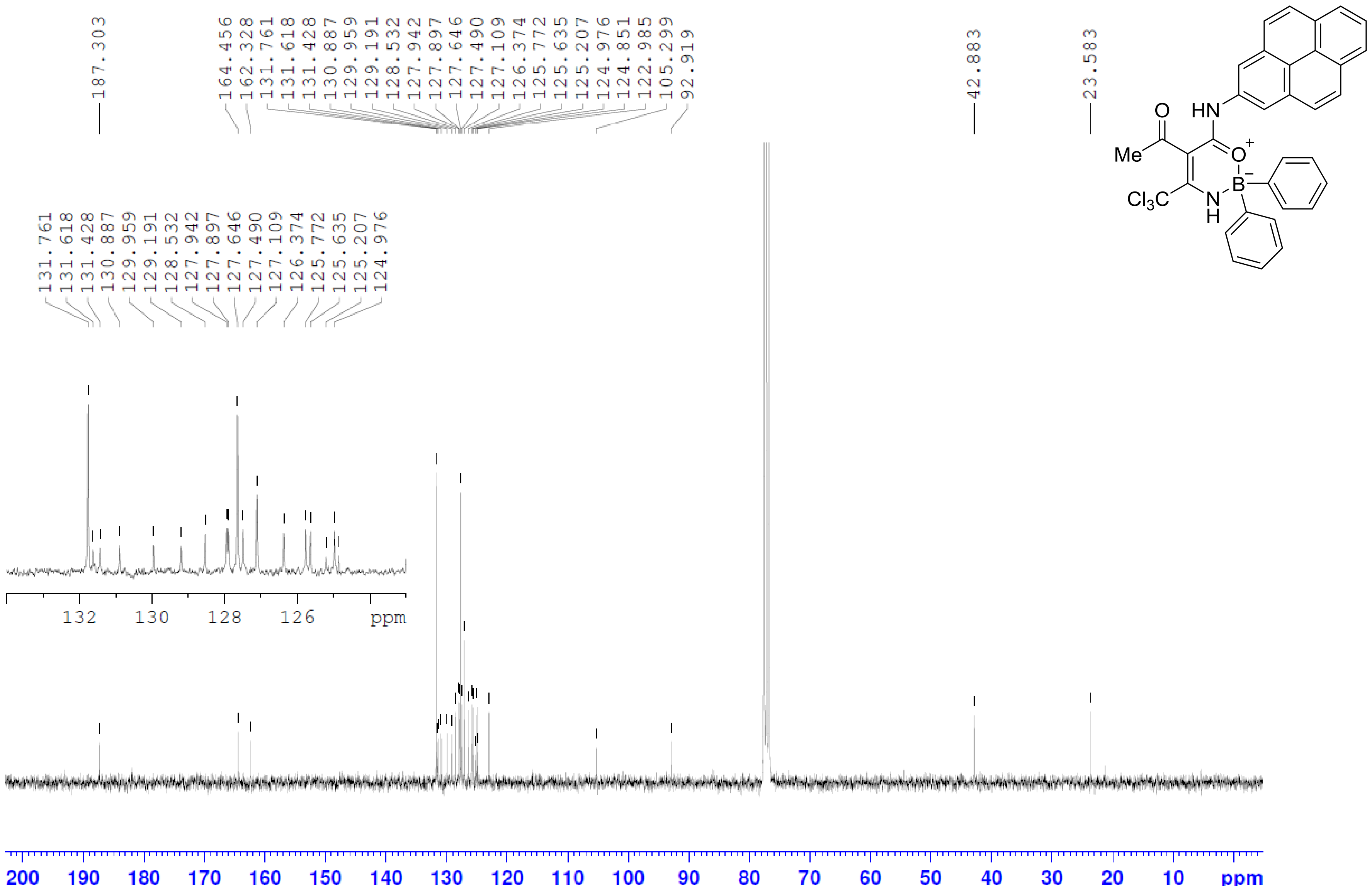

^{13}C NMR spectrum of NBC20 in $CDCl_3$

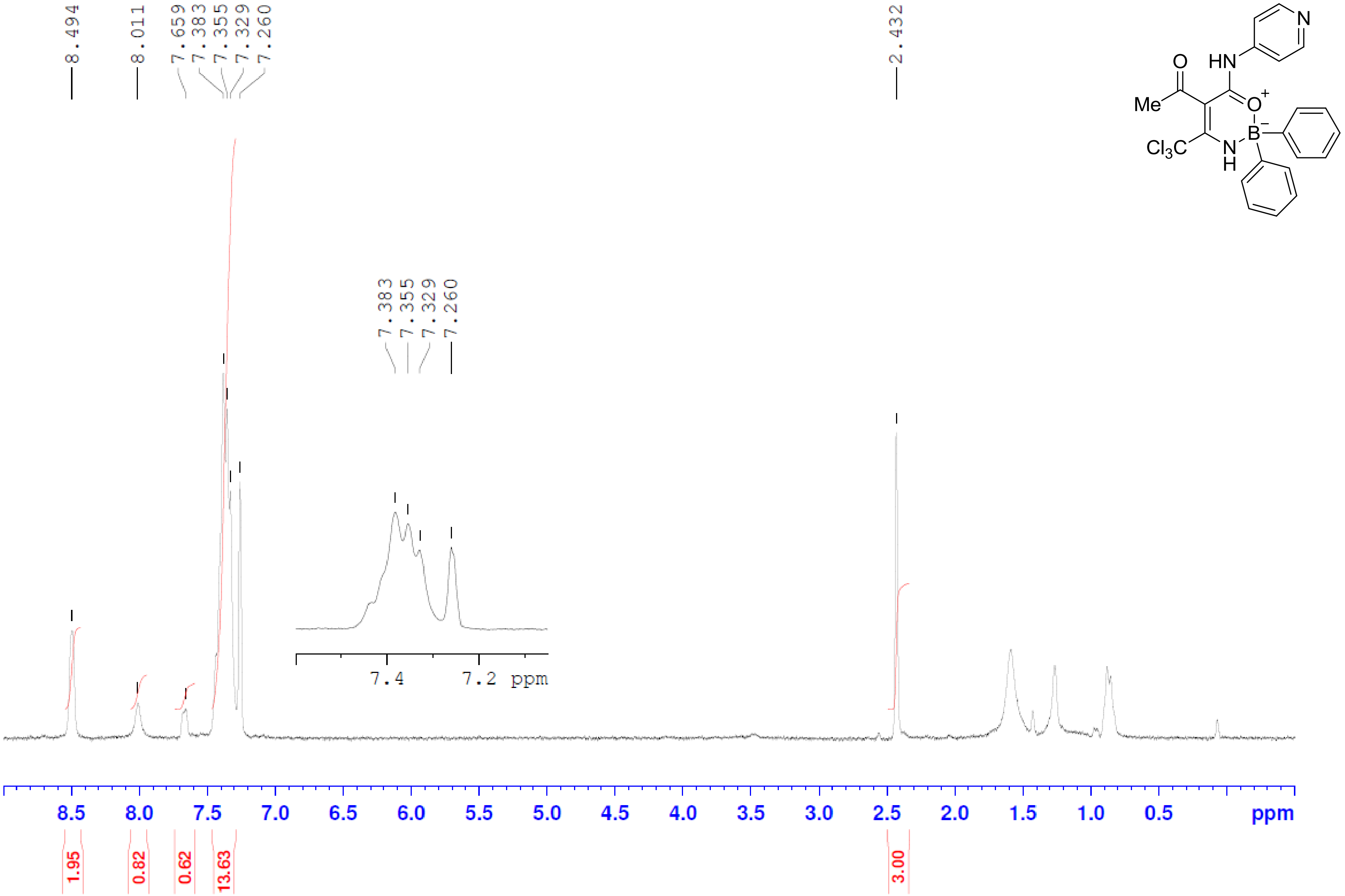

^{1}H NMR spectrum of NBC22 in $CDCl_3$

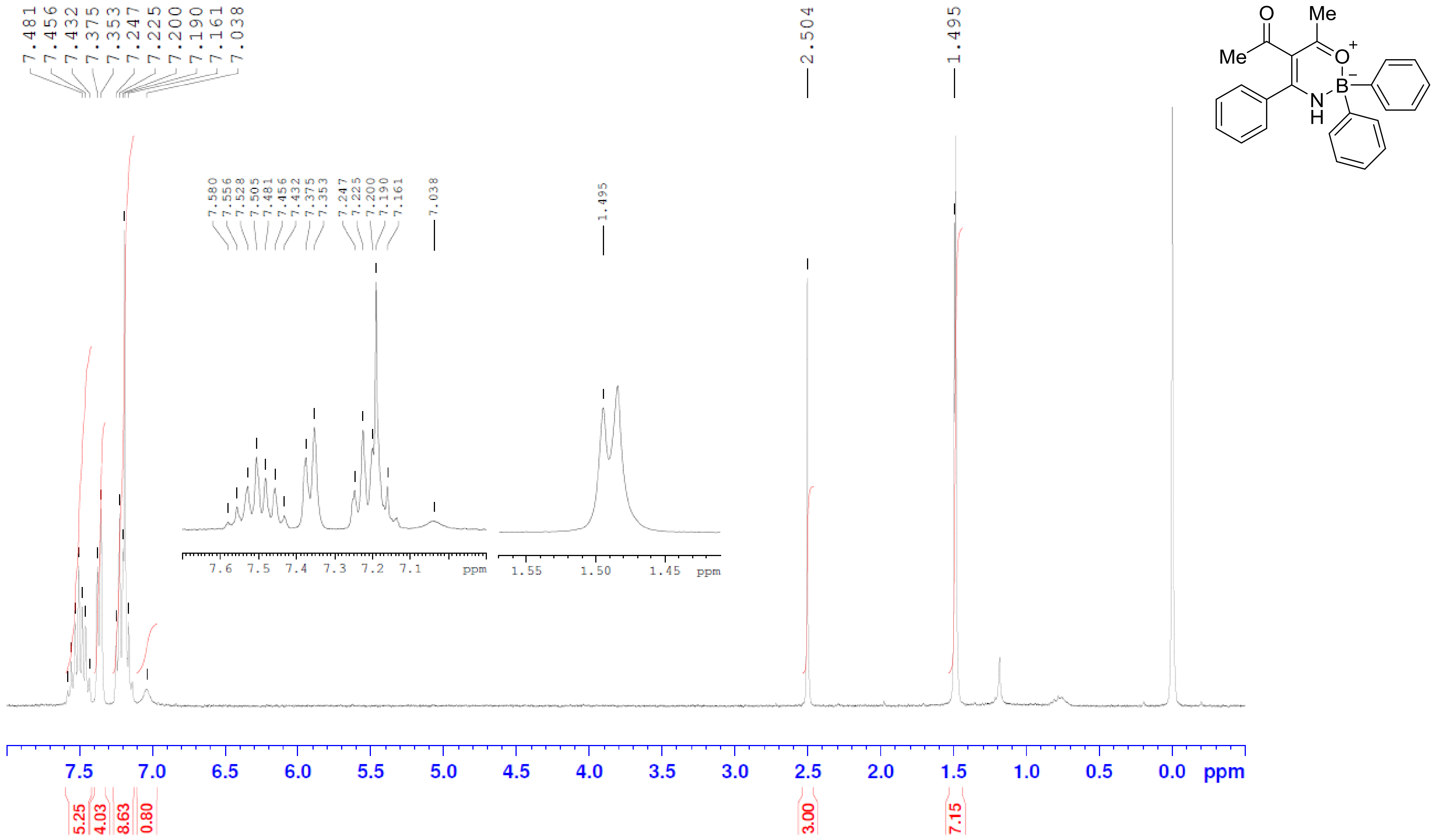

^{1}H NMR spectrum of NBC23 in $CDCl_3$

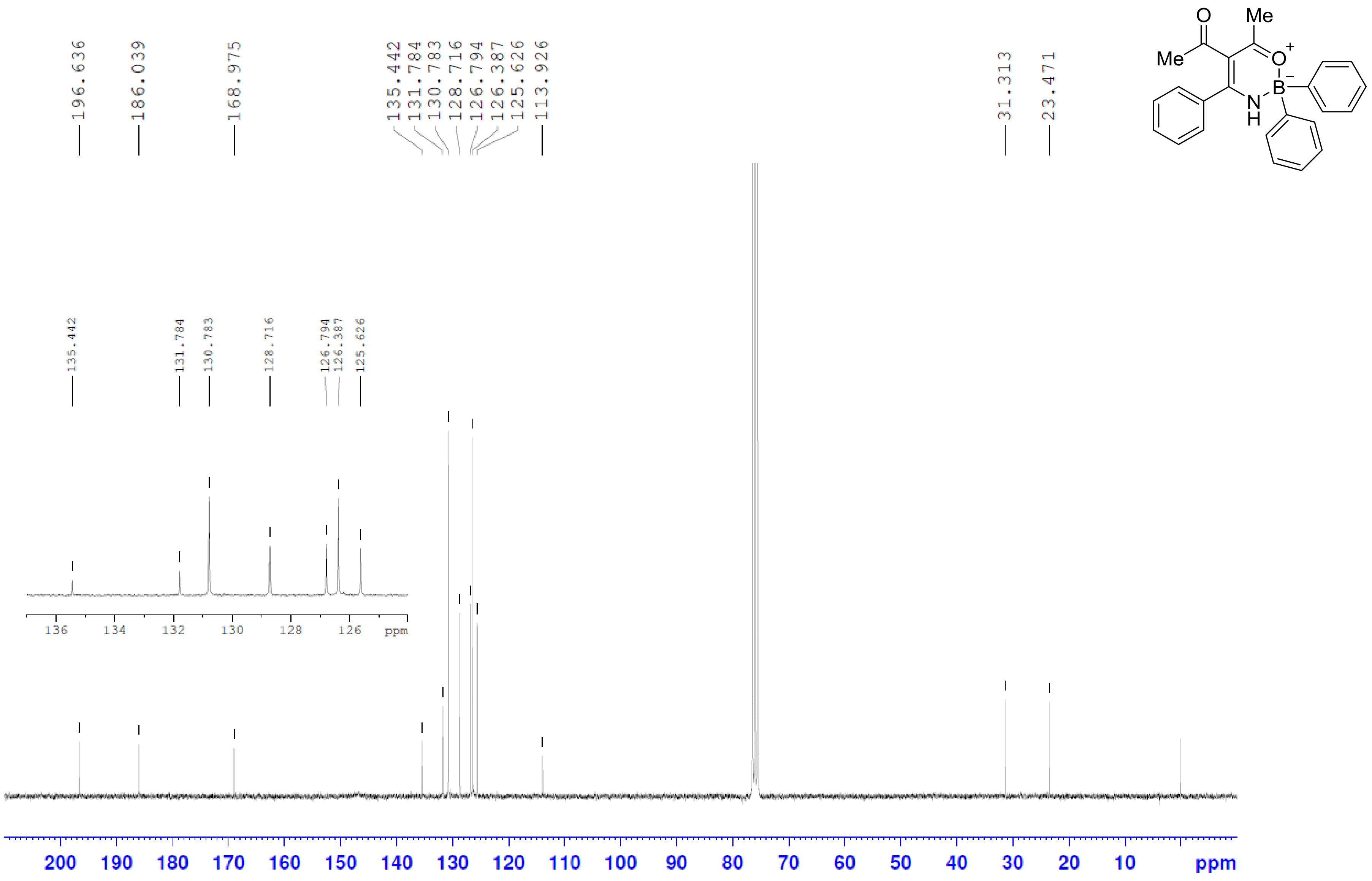

^{13}C NMR spectrum of NBC23 in $CDCl_3$

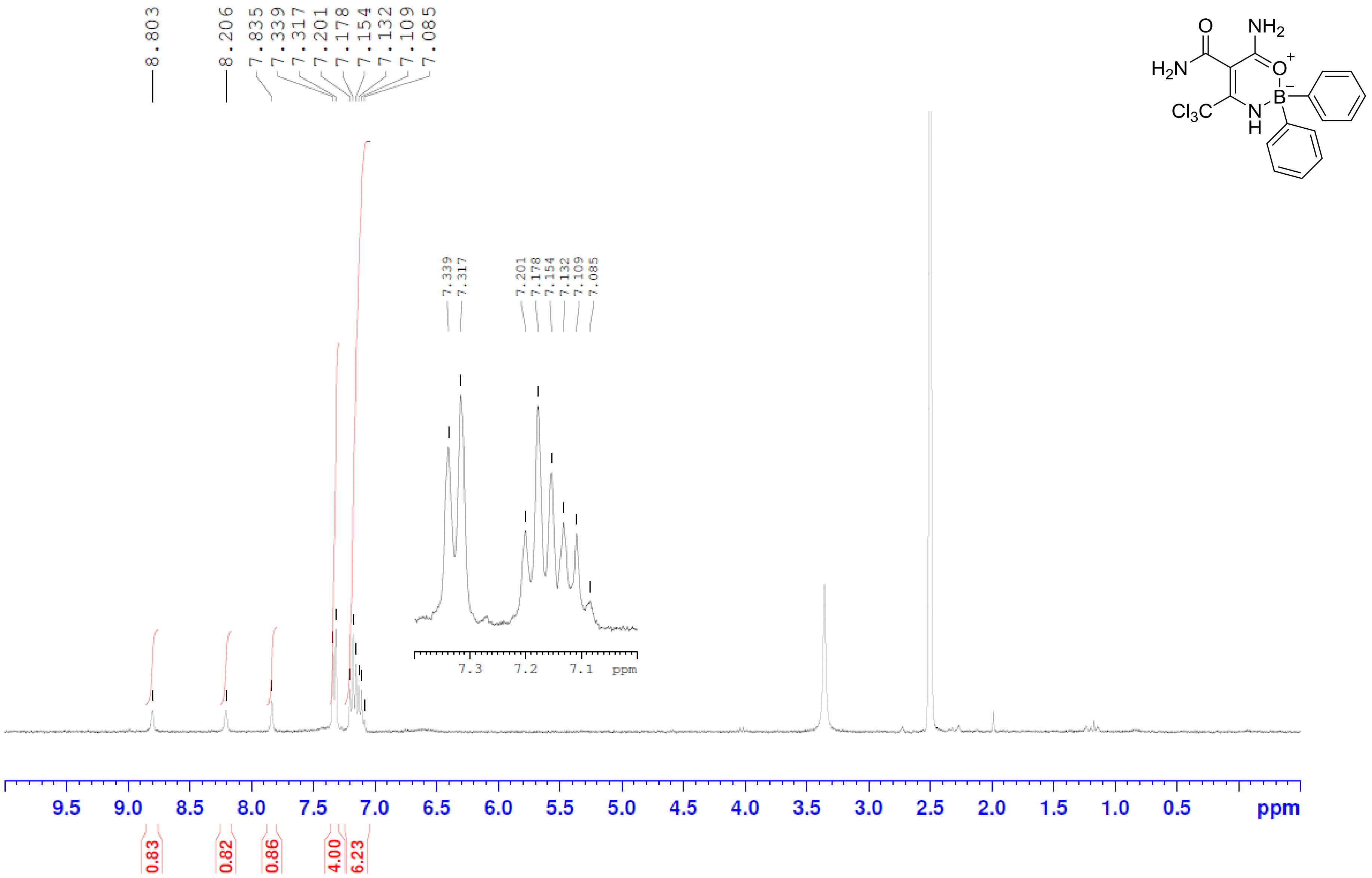

^{1}H NMR spectrum of NBC24 in $CDCl_3$

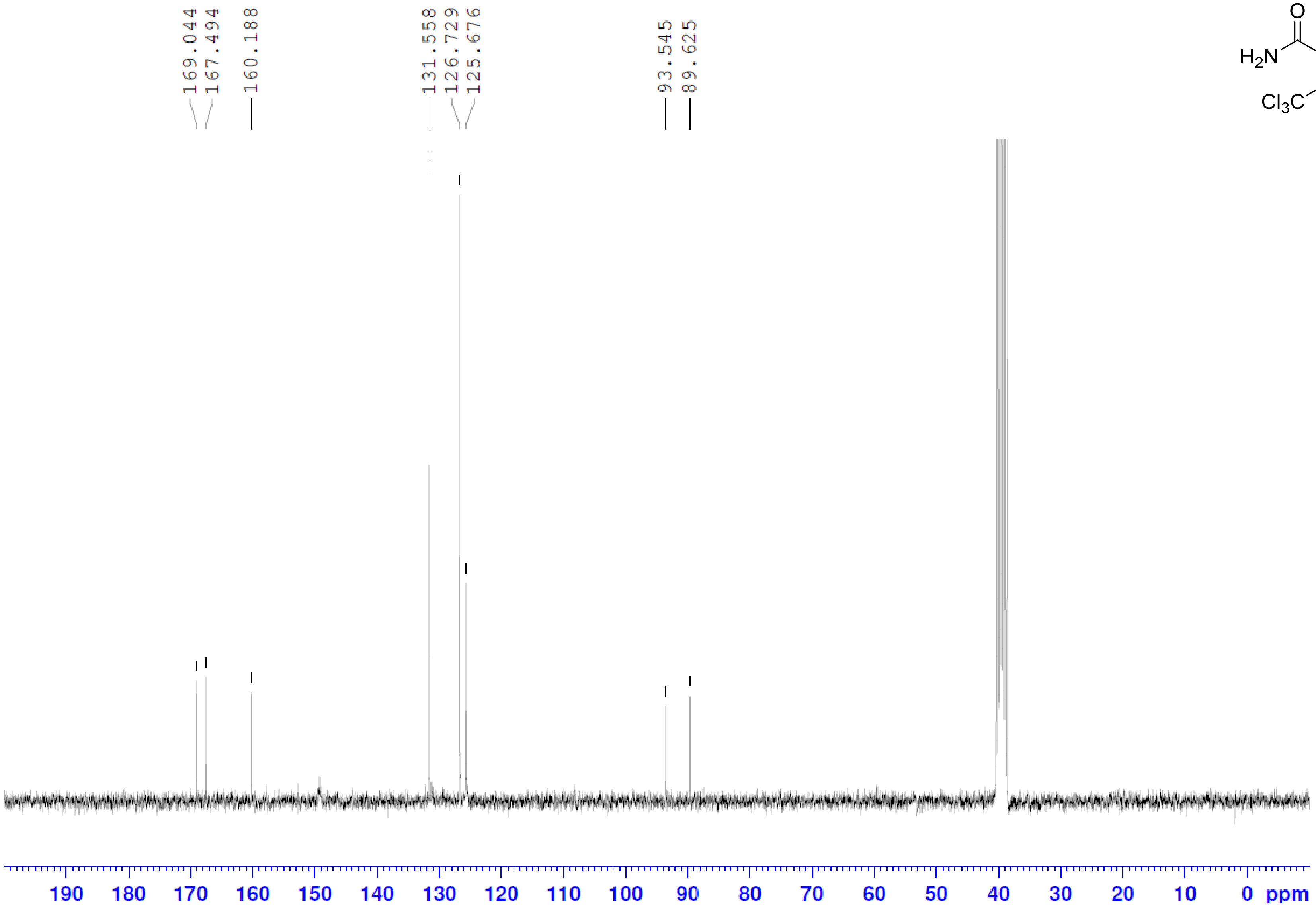

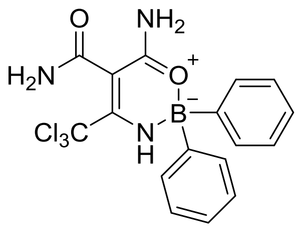

^{13}C NMR spectrum of NBC24 in $CDCl_3$

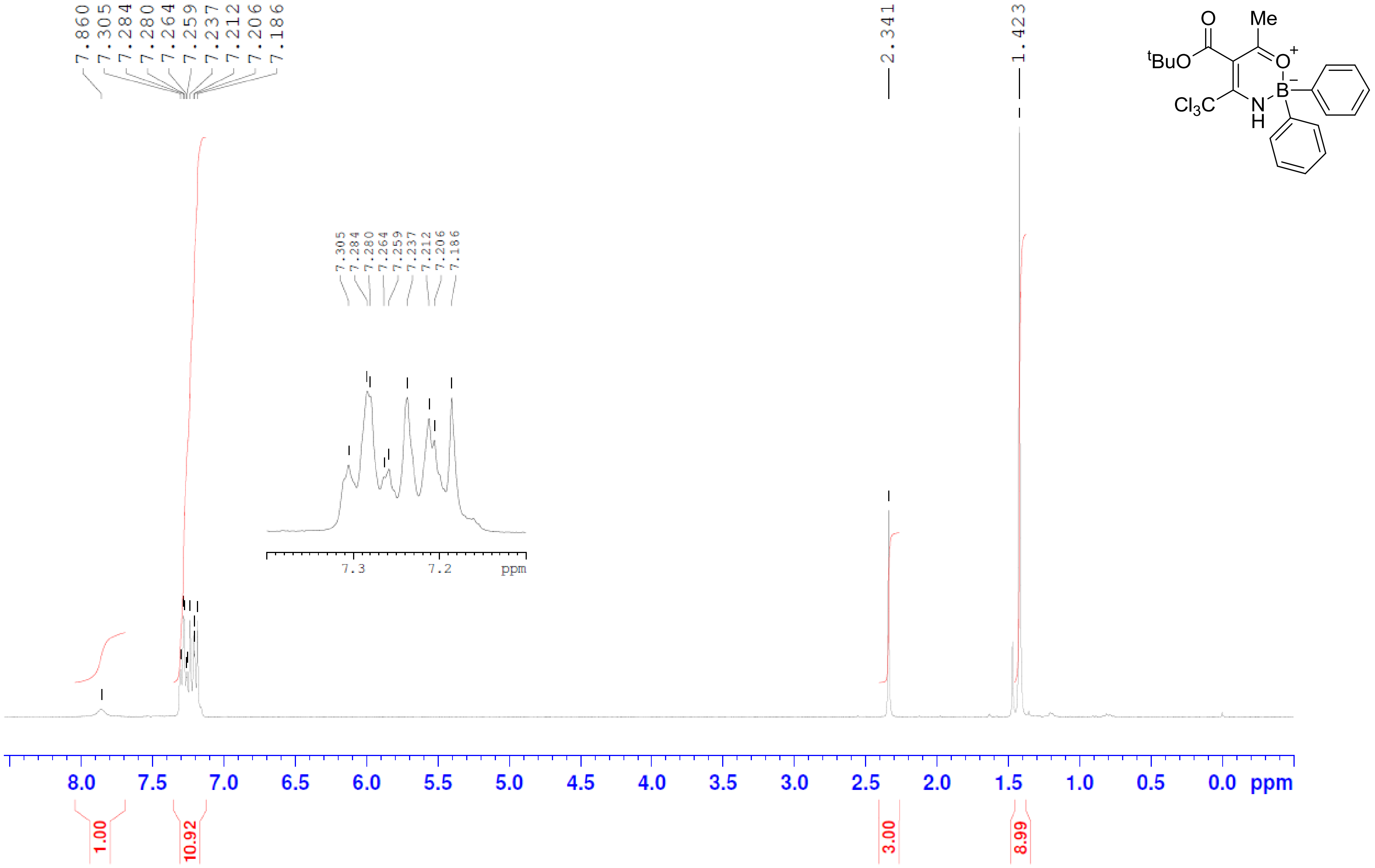

^{1}H NMR spectrum of NBC25 in $CDCl_3$

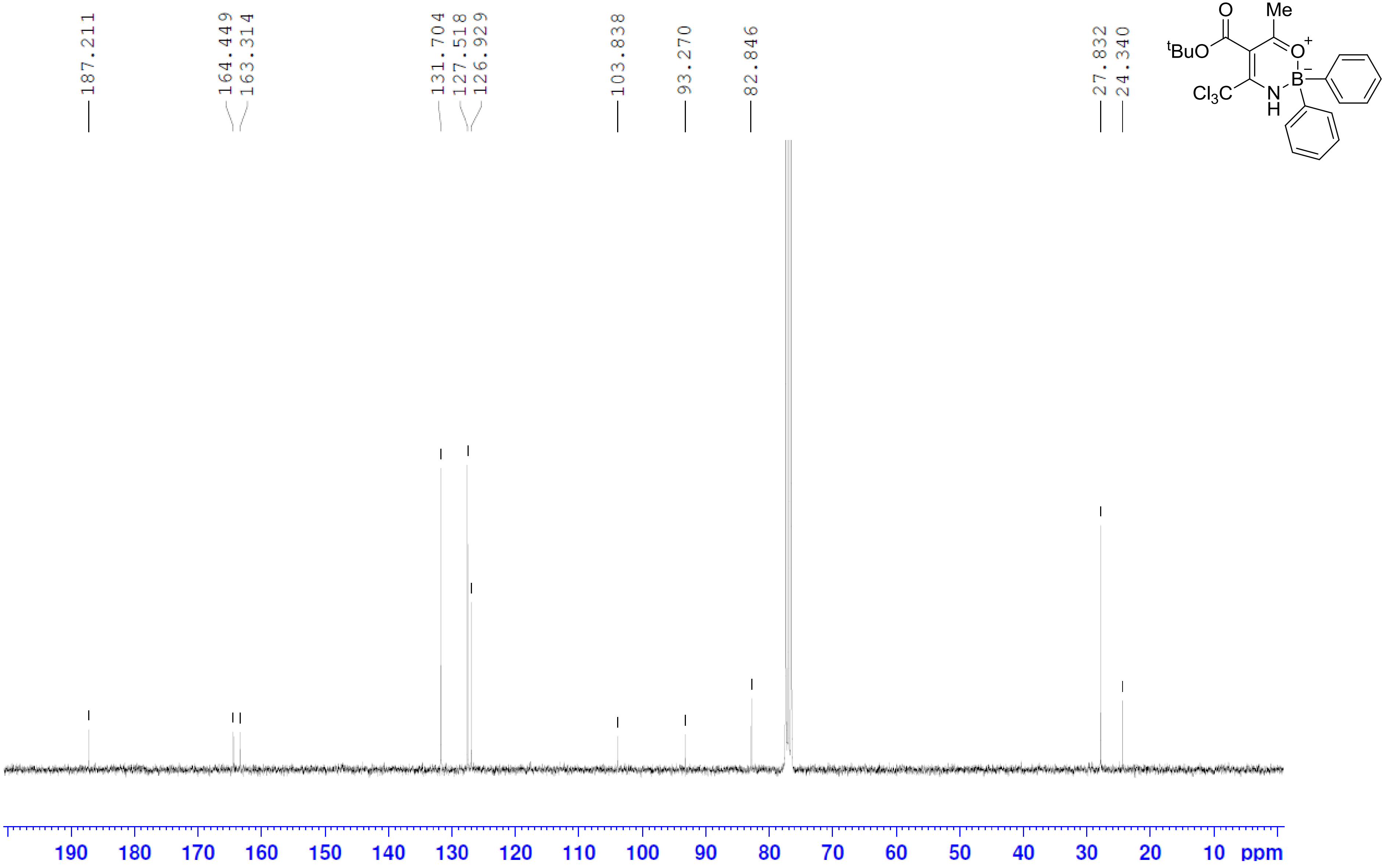

^{13}C NMR spectrum of NBC25 in $CDCl_3$

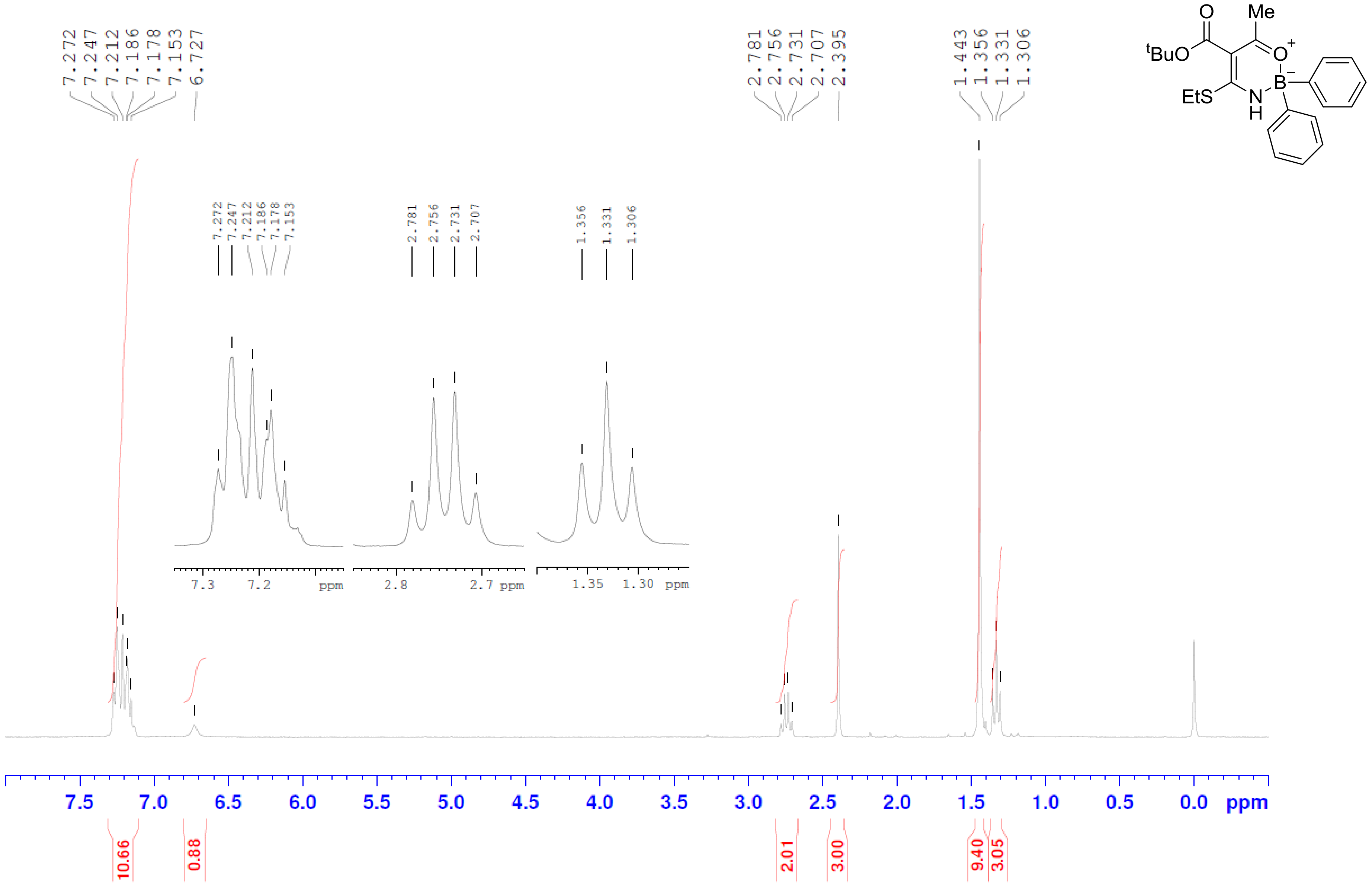

^{1}H NMR spectrum of NBC26 in $CDCl_3$

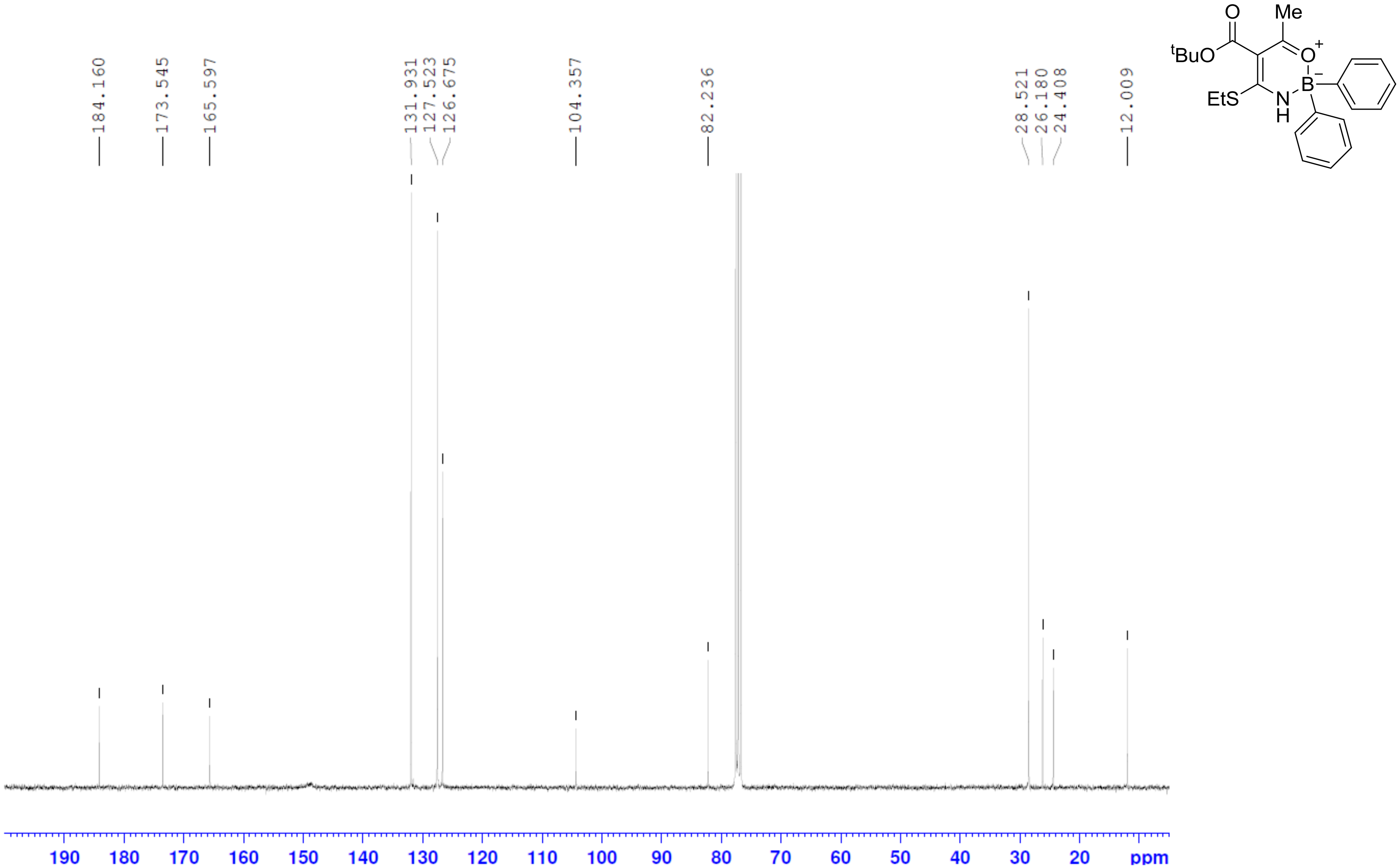

^{13}C NMR spectrum of NBC26 in $CDCl_3$

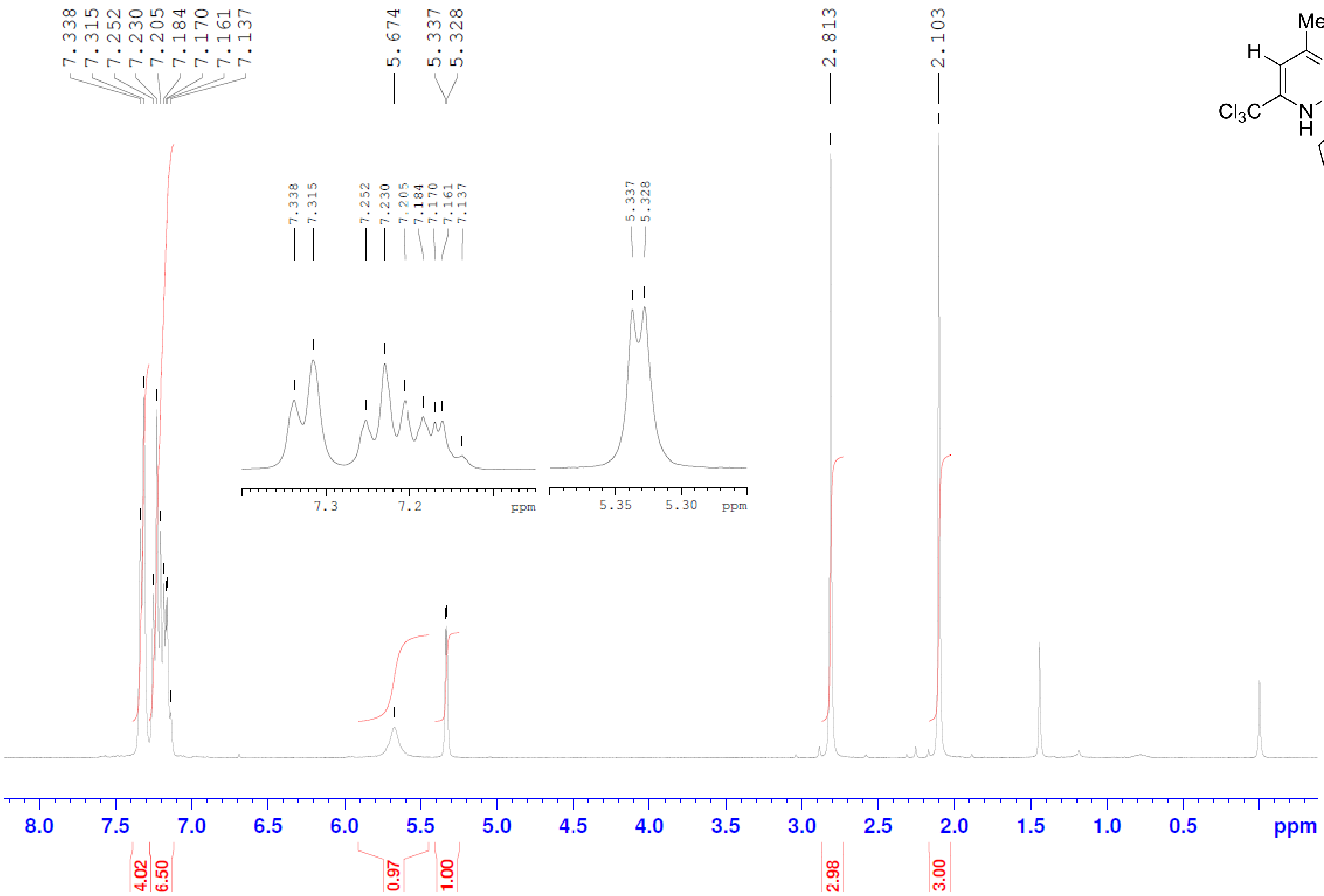

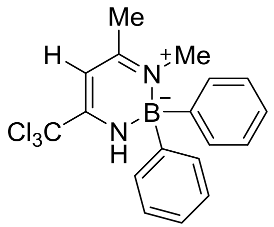

^{1}H NMR spectrum of NBC27 (**18**) in $CDCl_3$

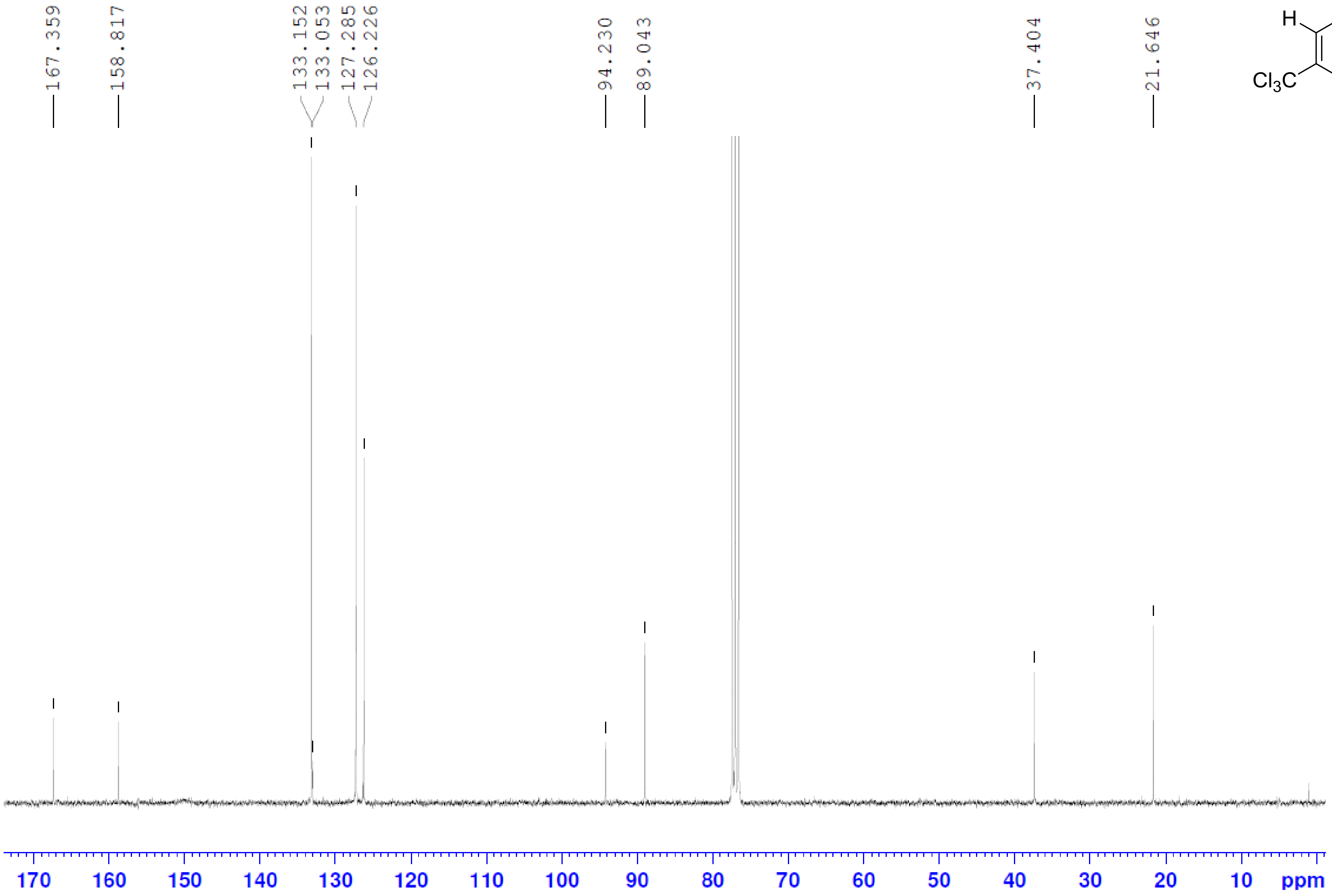

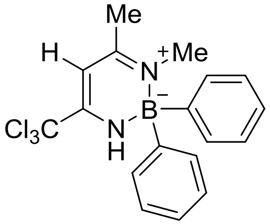

^{13}C NMR spectrum of NBC27 (**18**) in $CDCl_3$

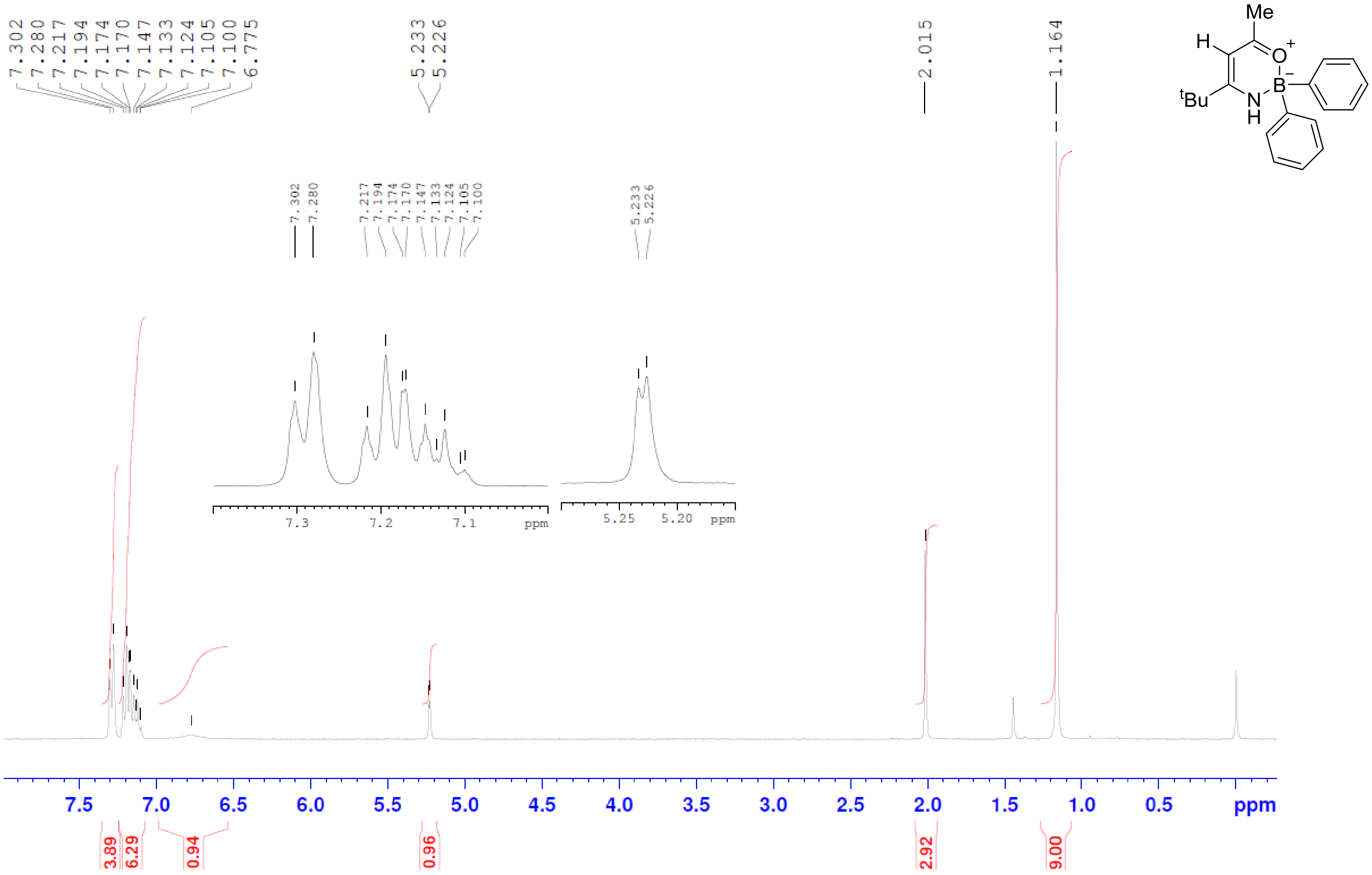

^{1}H NMR spectrum of NBC28 in $CDCl_3$

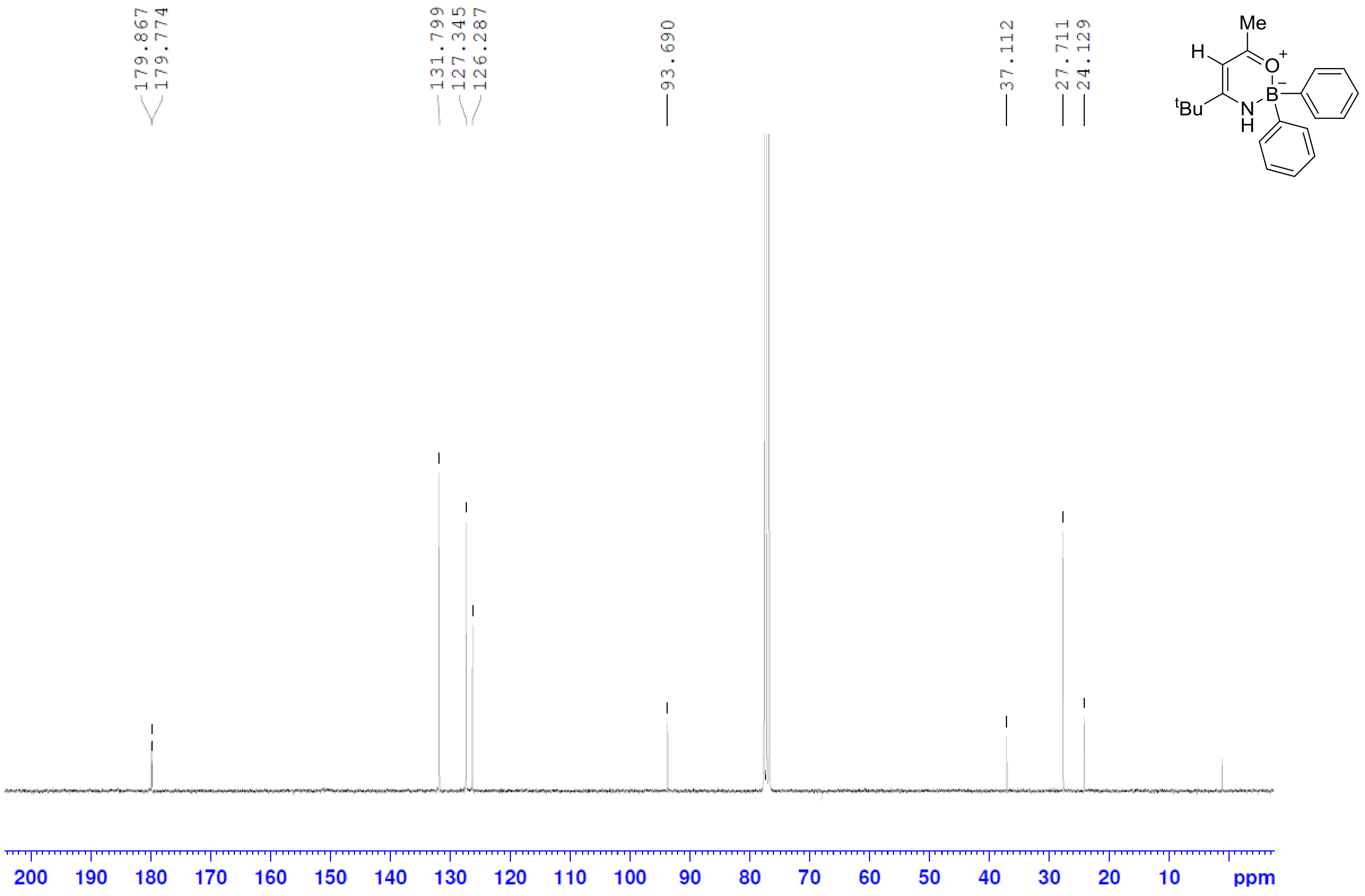

^{13}C NMR spectrum of NBC28 in $CDCl_3$

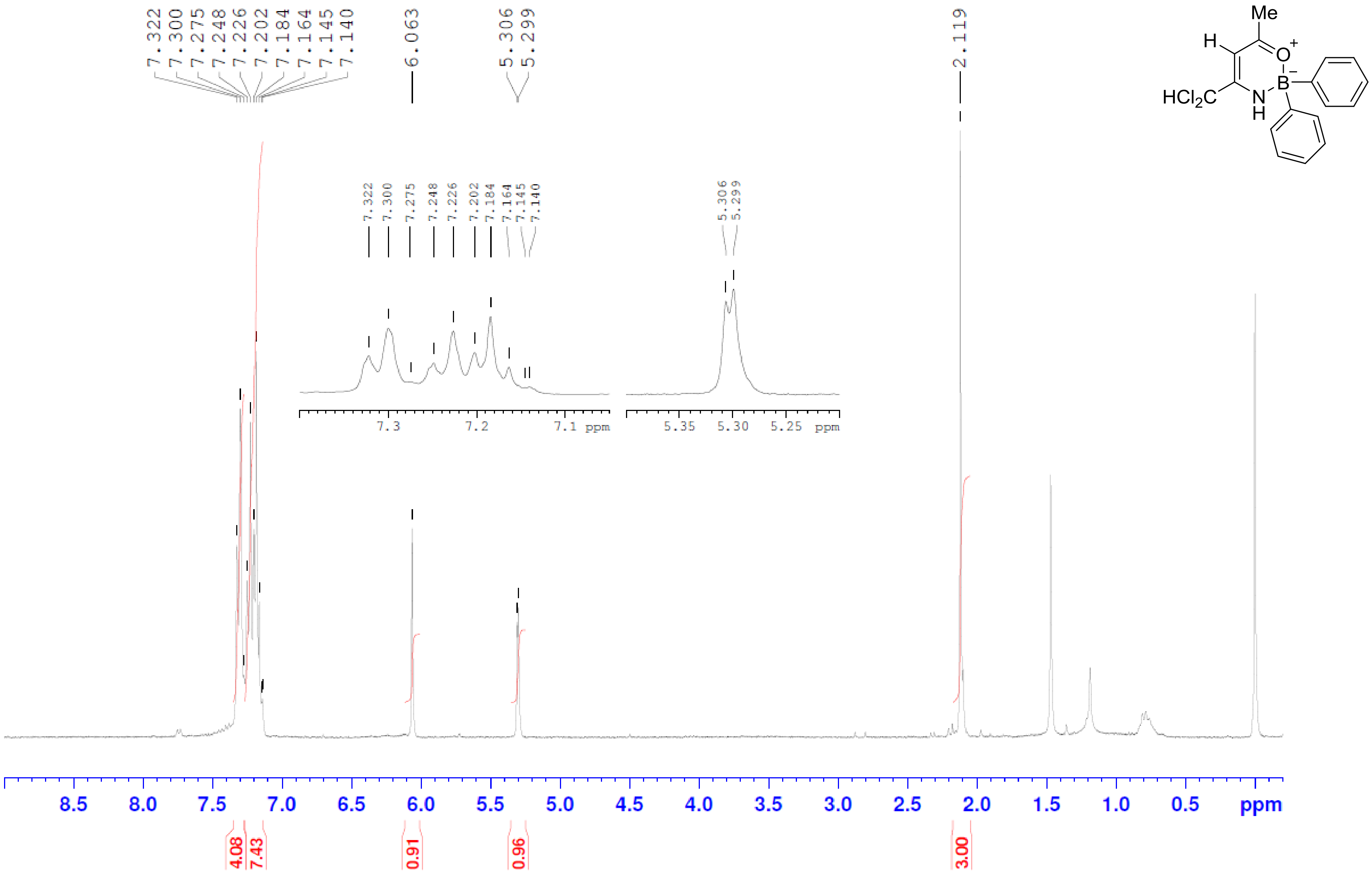

^{1}H NMR spectrum of NBC29 in $CDCl_3$

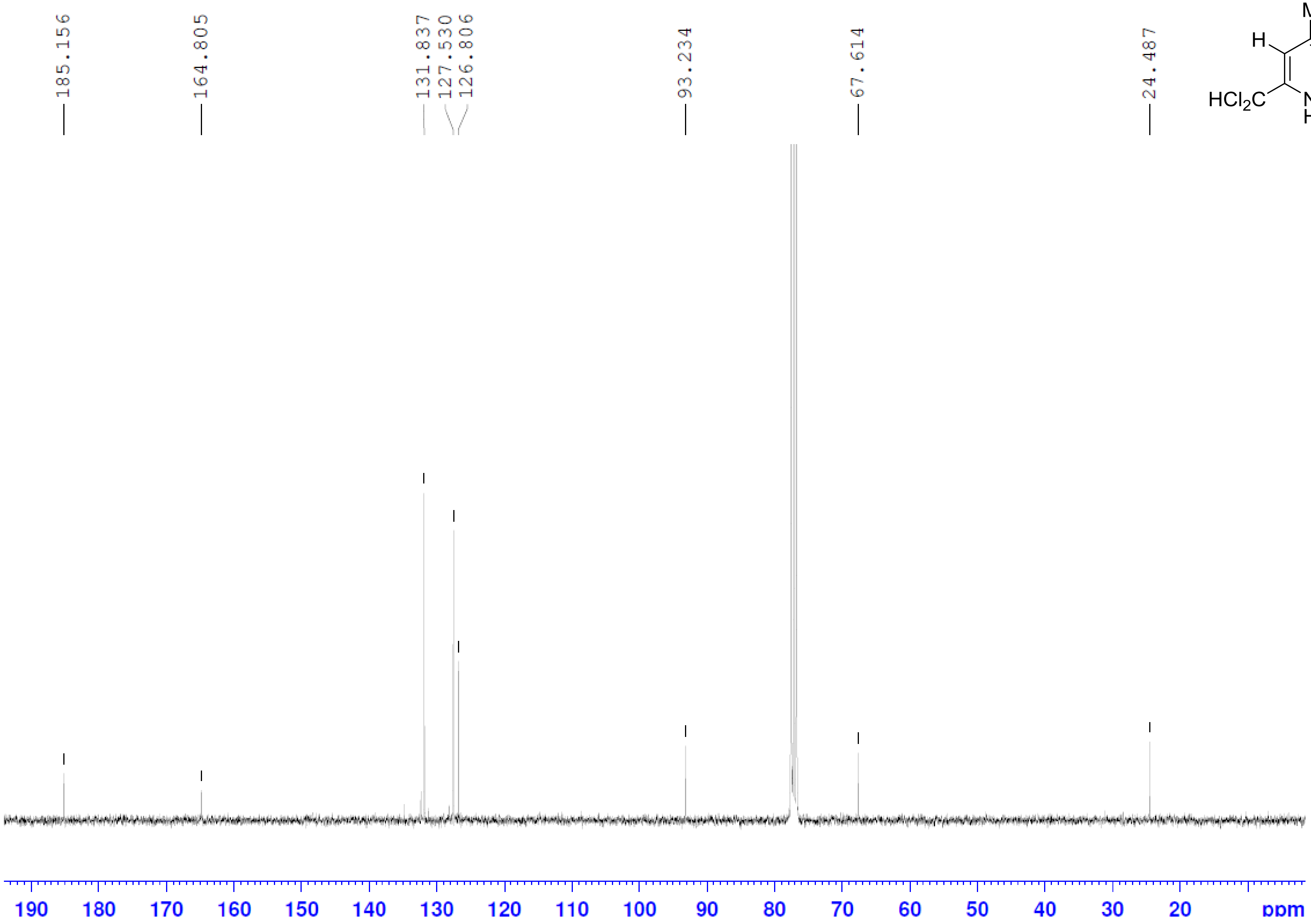

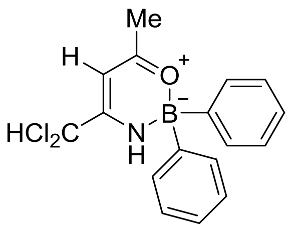

^{13}C NMR spectrum of NBC29 in $CDCl_3$

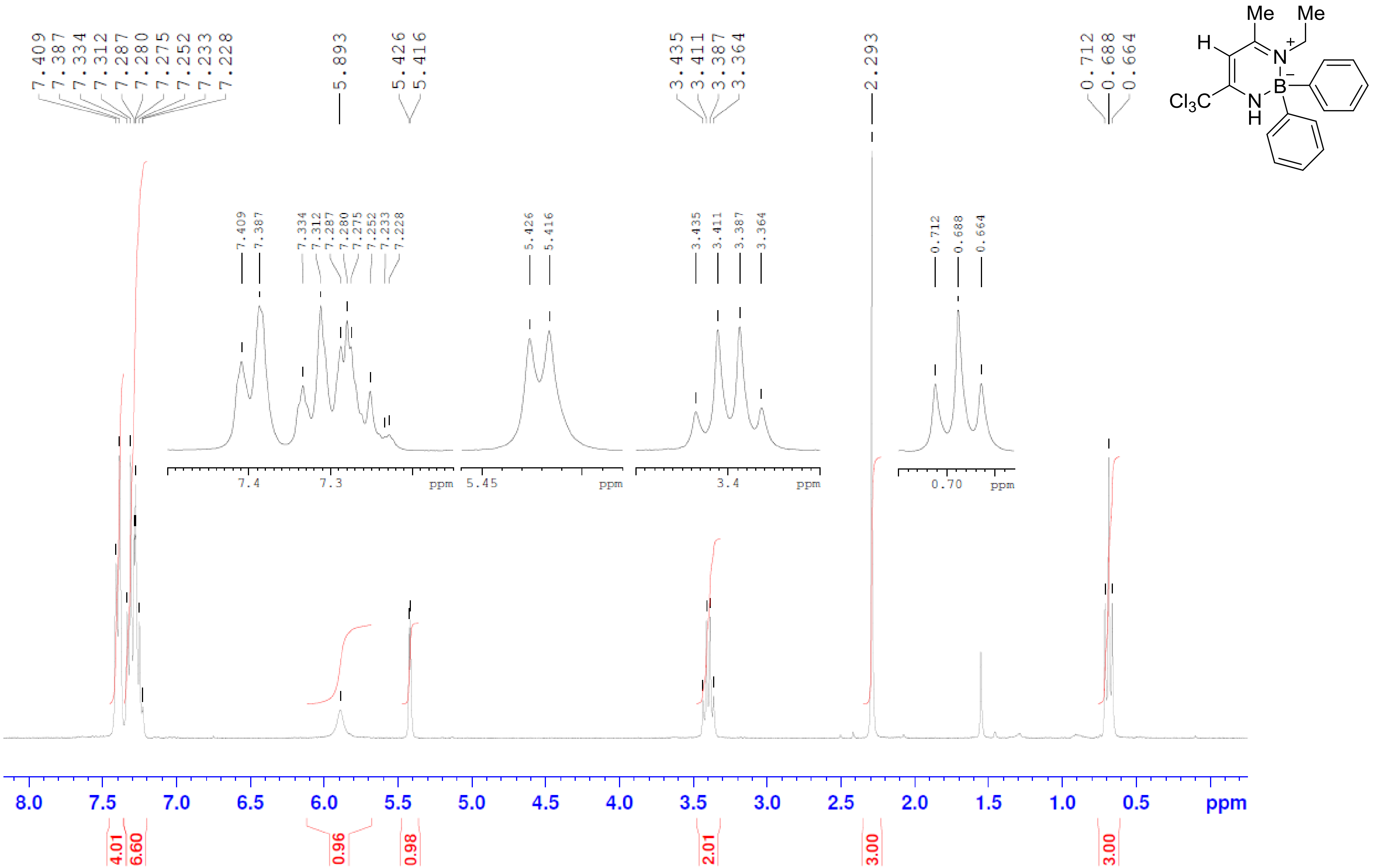

^{1}H NMR spectrum of NBC30 (**19**) in $CDCl_3$

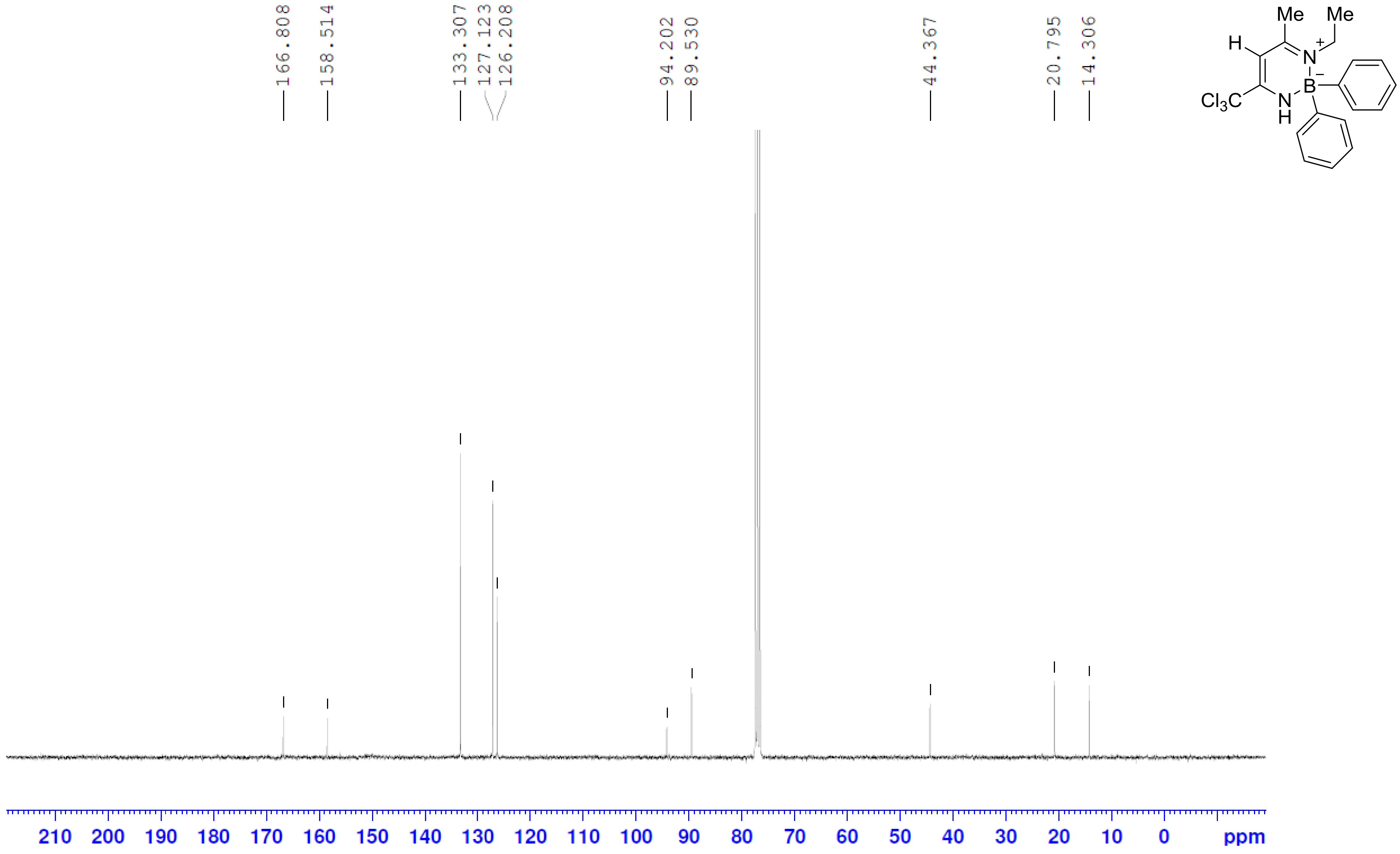

^{13}C NMR spectrum of NBC30 (**19**) in $CDCl_3$

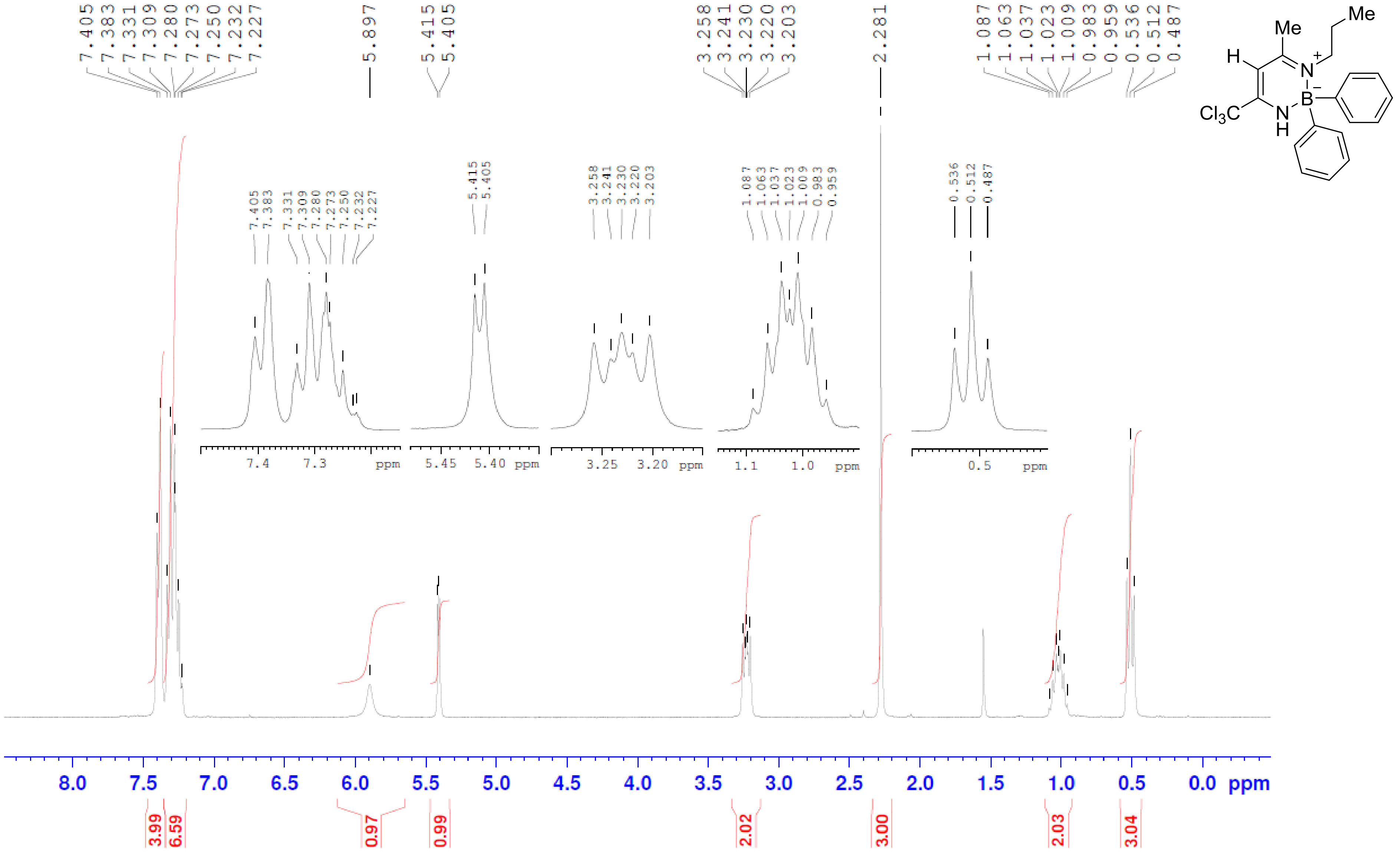

^{1}H NMR spectrum of NBC31 in $CDCl_3$

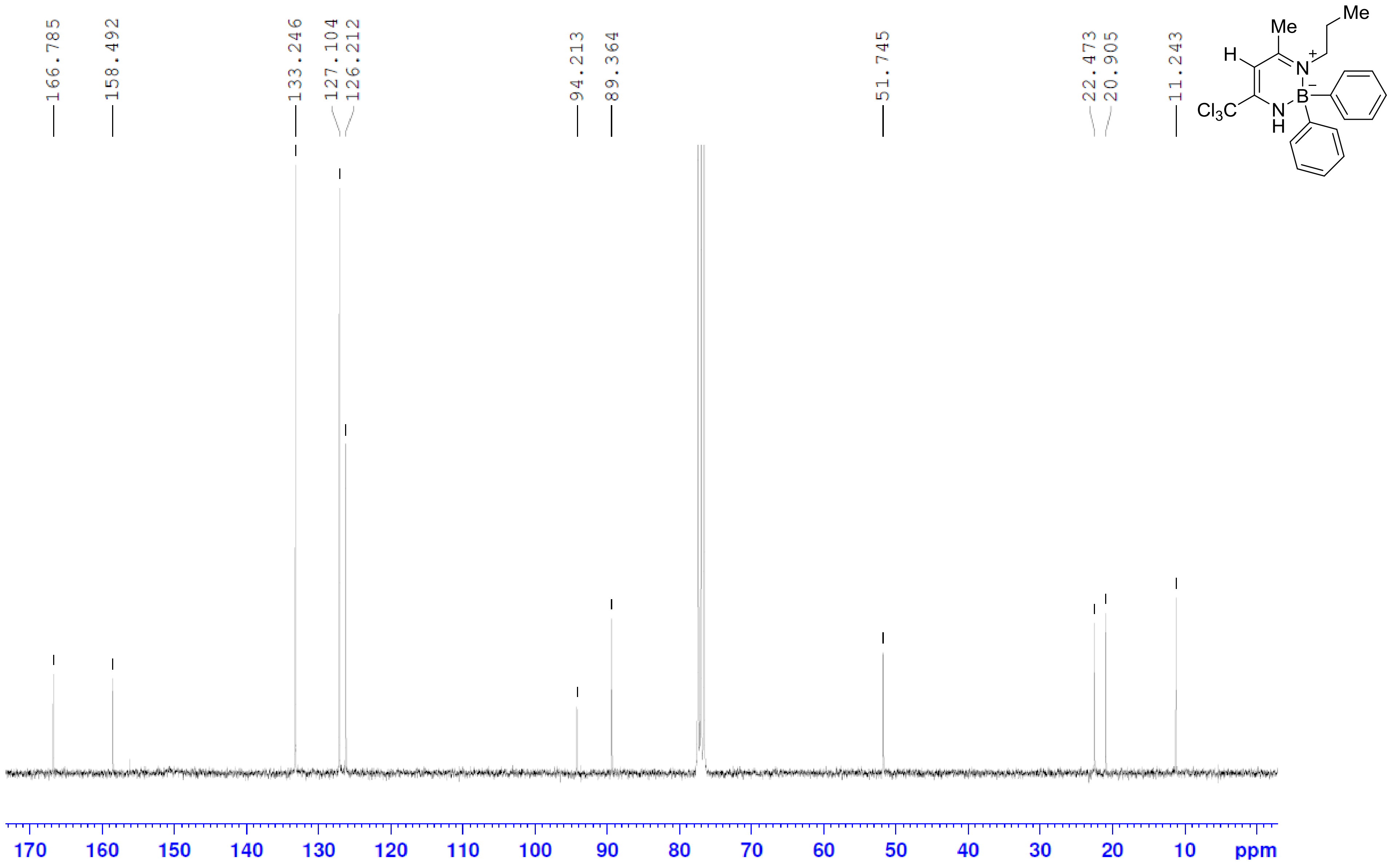

^{13}C NMR spectrum of NBC31 in $CDCl_3$

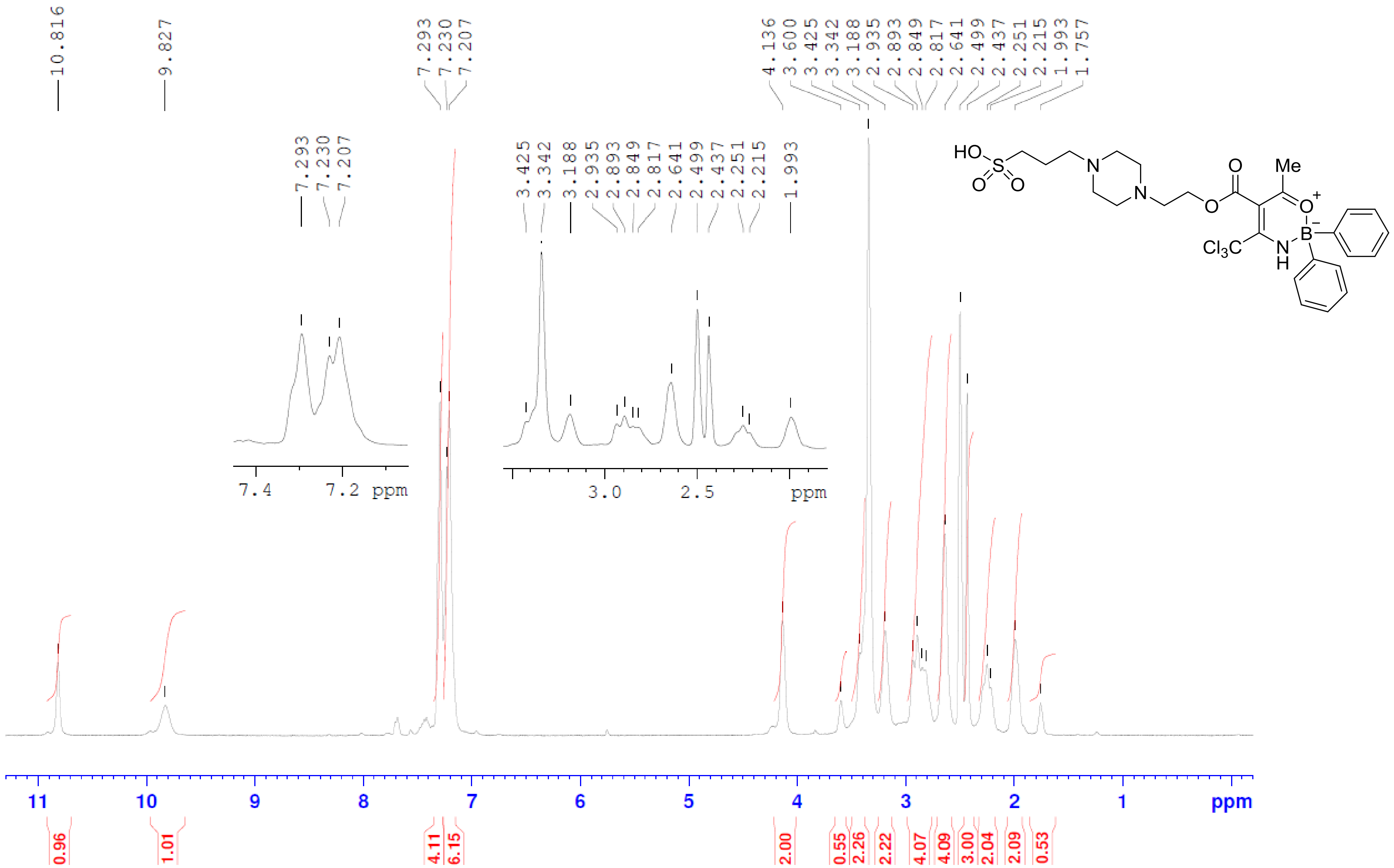

^{1}H NMR spectrum of NBC-EPPS in DMSO-d_6

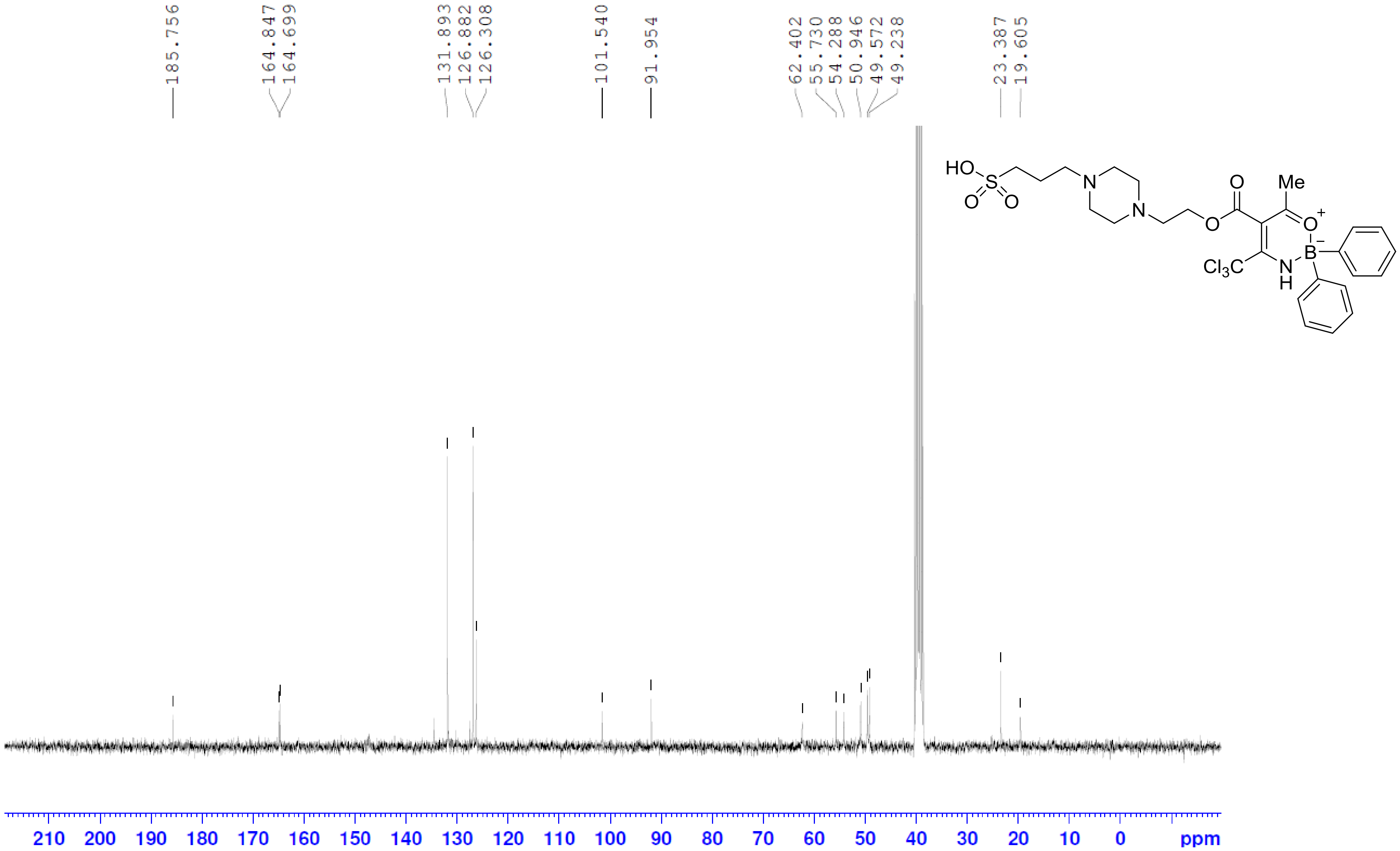

^{13}C NMR spectrum of NBC-EPPS in DMSO-d
