# Supplementary material for: Boron-Based Inhibitors of the NLRP3 Inflammasome
Source: Cell Chem Biol. 2017 Nov 16;24(11):1321–1335.e5. doi: 10.1016/j.chembiol.2017.08.011 (PMC5696570; doi:10.1016/j.chembiol.2017.08.011)
Supplement: Document S2. Article plus Supplemental Information [file mmc2.pdf]

Article

Cell Chemical Biology

Boron-Based Inhibitors of the NLRP3 Inflammasome

Graphical Abstract

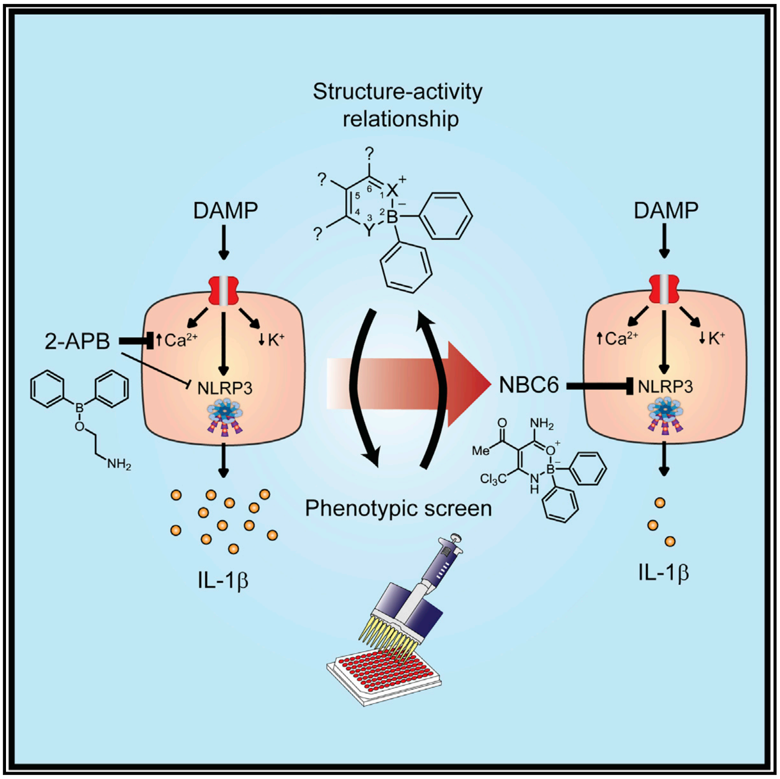

Authors

Alex G. Baldwin, Jack Rivers-Auty, Michael J.D. Daniels, ..., Claudia Eder, Sally Freeman, David Brough

Correspondence

sally.freeman@manchester.ac.uk (S.F.), david.brough@manchester.ac.uk (D.B.)

In Brief

The NLRP3 inflammasome is known to contribute to damaging inflammation during disease. Baldwin et al. describe a new boron-containing series of inflammasome inhibitors, which may lead to the development of new anti-inflammatory molecules and allow further interrogation of inflammatory mechanisms.

Highlights

- New inhibitors of the NLRP3 inflammasome are described
- NLRP3 inflammasome activation is independent of Ca^{2+}
- These new inflammasome inhibitors are effective *in vivo*
- The inhibitors described may lead to the development of new drugs

CrossMark
Baldwin et al., 2017, Cell Chemical Biology *24*, 1321–1335
November 16, 2017 © 2017 The Authors. Published by Elsevier Ltd.
http://dx.doi.org/10.1016/j.chembiol.2017.08.011

CellPress

Cell Chemical Biology
Article

Boron-Based Inhibitors of the NLRP3 Inflammasome

Alex G. Baldwin,[1] Jack Rivers-Auty,[2] Michael J.D. Daniels,[2] Claire S. White,[2] Carl H. Schwalbe,[3] Tom Schilling,[4] Halah Hammadi,[1] Panichakorn Jaiyong,[1] Nicholas G. Spencer,[4] Hazel England,[2] Nadia M. Luheshi,[2,5] Manikandan Kadirvel,[1] Catherine B. Lawrence,[2] Nancy J. Rothwell,[2] Michael K. Harte,[1] Richard A. Bryce,[1] Stuart M. Allan,[2] Claudia Eder,[4] Sally Freeman,[1,6,*] and David Brough[2,6,7,*]

[1]Division of Pharmacy and Optometry, School of Health Sciences, Faculty of Biology, Medicine and Health, Manchester Academic Health Science Centre, University of Manchester, Stopford Building, Oxford Road, Manchester M13 9PT, UK
[2]Division of Neuroscience and Experimental Psychology, School of Biological Sciences, Faculty of Biology, Medicine and Health, Manchester Academic Health Science Centre, University of Manchester, AV Hill Building, Oxford Road, Manchester M13 9PT, UK
[3]Aston Pharmacy School, School of Life & Health Sciences, Aston University, Aston Triangle, Birmingham B4 7ET, UK
[4]St. George's, University of London, Institute for Infection and Immunity, Cranmer Terrace, London SW17 0RE, UK
[5]MedImmune Ltd., Aaron Klug Building, Granta Park, Cambridge CB21 6GH, UK
[6]These authors contributed equally
[7]Lead Contact
*Correspondence: sally.freeman@manchester.ac.uk (S.F.), david.brough@manchester.ac.uk (D.B.)
http://dx.doi.org/10.1016/j.chembiol.2017.08.011

SUMMARY

NLRP3 is a receptor important for host responses to infection, yet is also known to contribute to devastating diseases such as Alzheimer's disease, diabetes, atherosclerosis, and others, making inhibitors for NLRP3 sought after. One of the inhibitors currently in use is 2-aminoethoxy diphenylborinate (2APB). Unfortunately, in addition to inhibiting NLRP3, 2APB also displays non-selective effects on cellular Ca^{2+} homeostasis. Here, we use 2APB as a chemical scaffold to build a series of inhibitors, the NBC series, which inhibit the NLRP3 inflammasome *in vitro* and *in vivo* without affecting Ca^{2+} homeostasis. The core chemical insight of this work is that the oxazaborine ring is a critical feature of the NBC series, and the main biological insight the use of NBC inhibitors led to was that NLRP3 inflammasome activation was independent of Ca^{2+}. The NBC compounds represent useful tools to dissect NLRP3 function, and may lead to oxazaborine ring-containing therapeutics.

INTRODUCTION

Inflammation, which contributes to almost all known non-infectious diseases, is triggered by infection or injury sensed by pattern recognition receptors (PRRs) on inflammatory cells. Soluble PRRs have received particular attention due to their ability to form molecular complexes known as inflammasomes, which facilitate the release of inflammatory cytokines such as interleukin-1β (IL-1β), an important aspect of the inflammatory response. Inflammasomes are formed following the activation of cytosolic PRRs, of which the NOD-like receptor (NLR) family, pyrin domain-containing protein 3 (NLRP3), is the best characterized. The NLRP3 inflammasome is formed when NLRP3, described mainly in macrophages and monocytes, senses the presence of pathogen-associated molecular patterns (PAMPs) or damage-associated molecular patterns (DAMPs). Upon its activation, NLRP3 binds to the adapter protein ASC (apoptosis-associated speck-like protein containing a caspase activation and recruitment domain), which in turn recruits pro-caspase-1 to form an inflammasome complex. This results in the activation of caspase-1, which in turn cleaves pro-forms of the pro-inflammatory cytokines IL-1β and IL-18, causing their activation and facilitating their release from the cell (Latz et al., 2013).

NLRP3-dependent cytokine release is implicated in the development of several important diseases (McGettrick and O'Neill, 2013; Heneka et al., 2015) and may represent a pharmacological target for the treatment of inflammatory disease (Coll et al., 2015; Daniels et al., 2016). Signaling mechanisms regulating the activation of NLRP3 remain to be fully characterized. One signaling mechanism proposed to regulate the activation of the NLRP3 inflammasome is an increase in intracellular calcium ($[Ca^{2+}]_i$) (Horng, 2014). Many reports suggesting an involvement of Ca^{2+} in inflammasome activation have used the Ca^{2+}-signaling inhibitor 2-aminoethoxy diphenylborinate (2APB, **1**) (Lee et al., 2012; Murakami et al., 2012; Compan et al., 2012; Rossol et al., 2012).

2APB is a cell-permeable small-molecule inhibitor of Ca^{2+} homeostasis with multiple targets including inositol 1,4,5-trisphosphate ($InsP_3$)-dependent Ca^{2+} release, store-operated Ca^{2+} entry, and potentially also Ca^{2+} pumps and mitochondria, where effects are described as use-dependent and poorly reversible (Peppiatt et al., 2003). 2APB is also a poorly selective TRP (transient receptor potential) channel blocker (Schaefer, 2014). However, recent evidence suggests that the effects of 2APB on inflammasome activation may be independent of an effect on Ca^{2+} (Katsnelson et al., 2015). The utility of 2APB as an inhibitor of NLRP3, however, is limited by its non-selective effects on cellular Ca^{2+} homeostasis. Our aim was to develop new and potent inflammasome inhibitors based on the scaffold of 2APB but with reduced non-specific effects on Ca^{2+} homeostasis. We describe NBC6 (and its analogs) as completely new and potent inhibitors of the NLRP3 inflammasome that act independently of Ca^{2+}.

© 2017 The Authors. Published by Elsevier Ltd.
This is an open access article under the CC BY license (http://creativecommons.org/licenses/by/4.0/).

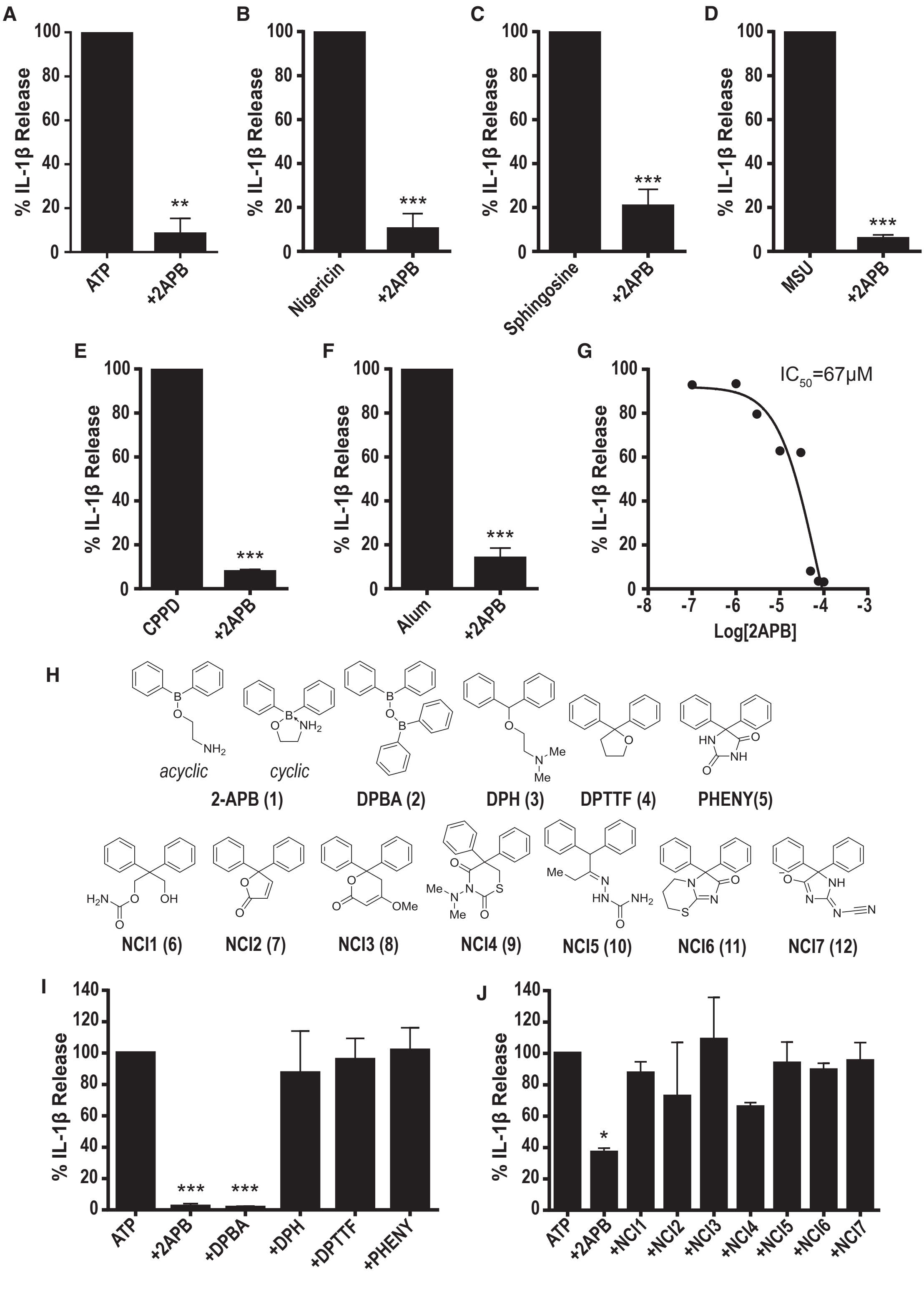

Figure 1. Establishing the Importance of Boron in 2APB for NLRP3 Inflammasome Inhibition

(A–G) Mouse peritoneal macrophages were primed with bacterial endotoxin (lipopolysaccharide [LPS], 1 μg mL^{-1}, 2 hr) and then stimulated with vehicle (0.5% DMSO) or 2APB (75 μM) before stimulation with ATP (5 mM, 20 min) (A), nigericin (20 μM, 15 min) (B), sphingosine (20 μM, 1 hr) (C), monosodium urate crystals (MSU; 250 μg mL^{-1}, 1 hr) (D), calcium pyrophosphate dehydrate crystals (CPPD; 250 μg mL^{-1}, 1 hr) (E), or aluminum hydroxide (Alum; 250 μg mL^{-1}, 1 hr) (F). The half-maximal inhibitory concentration (IC_{50}) for the effects of 2APB on IL-1β release induced by ATP was established using a 3-parameter logistical sigmoidal model (G).

(legend continued on next page)

RESULTS

Inhibitory Effects of 2APB Require Boron

To establish that 2APB was a robust NLRP3 inflammasome inhibitor, mouse peritoneal macrophages were primed with LPS and then stimulated with a range of NLRP3 inflammasome-activating DAMPs. After LPS cells received pre-treatment with 2APB, which was then present for the duration of DAMP stimulation, 2APB inhibited the release of IL-1β in response to NLRP3 inflammasome activators ATP, nigericin, sphingosine, monosodium urate crystals (MSU), calcium pyrophosphate dihydrate crystals (CPPD), or aluminum hydroxide (Alum) (Figures 1A–1F), consistent with previous work reporting 2APB as an inhibitor of the NLRP3 inflammasome (Lee et al., 2012; Murakami et al., 2012; Compan et al., 2012; Rossol et al., 2012; Katsnelson et al., 2015). To identify the pharmacophore of 2APB responsible for the inhibition of IL-1β processing and release, we screened a small library of 2APB analogs based on previously published data investigating the pharmacophore responsible for the effects of 2APB on store-operated Ca^{2+} entry (Dobrydneva and Blackmore, 2001). The acyclic structure of 2APB (**1**) is shown in Figure 1H, although in reality the ethanolamine coordinates to the boron (B) atom to give a 5-membered ring cyclic structure. Also shown is the analog diphenylborinic anhydride (DPBA, **2**, the dimerized dehydrated form of diphenylborinic acid), diphenhydramine (DPH, **3**, a carbon analog of acyclic 2APB in which there is no possibility of a closed ring form), 2,2-diphenyltetrahydrofuran (DPTTF, **4**, which contains no B), and phenytoin (PHENY, **5**, which is similar to 2APB in that it has two phenyl groups attached to a heterocyclic ring, but again does not contain B). To test the effects of these analogs, we primed mouse peritoneal macrophages with LPS and then pre-treated with drug before stimulation with ATP. The drug was also present throughout the ATP stimulation. The effects of the drugs were normalized to ATP-induced IL-1β release in the absence of any drug (Figure 1I). The only analog to inhibit IL-1β release, in addition to 2APB, was the B-containing DPBA, with the other analogs having no effect (Figure 1I). The half-maximal inhibitory concentration (IC_{50}) for the effects of 2APB on IL-1β release was 67 μM (Figure 1G).

These data suggest the B atom is essential for the inhibitory effects of 2APB on IL-1β release. To further test the requirement for B, we used computational similarity searching using ROCS (rapid overlay of chemical structures) (Grant et al., 1996) and Tanimoto scoring (ShapeTanimoto and ColorTanimoto, for shape and chemical similarity, respectively), to identify diverse commercially available carbon analogs of 2APB for screening. Several of the top ranked hits, selected on shape and pharmacophore match (from a library of ~2 million non-B-containing compounds from the ZINC "LeadsNow" database, zinc.docking.org), were sourced via the repository of the NIH's National Cancer Institute (NCI) Developmental Therapeutics Program and were screened against ATP-induced IL-1β release using primary mouse bone marrow-derived macrophages (BMDMs). BMDMs were primed with LPS and incubated with vehicle (0.5% DMSO) or molecules (NCI1–7, **6–12**, Figure 1H) at 40 μM (to allow any enhanced inhibitory activity to be observed) for 15 min before ATP stimulation. The effects of the molecules were normalized to ATP-induced IL-1β release (Figure 1J). None of the carbon analogs were as effective as 2APB at this concentration, suggesting that the B atom is important for the inhibitory activity of 2APB.

Refinement of the Structure-Activity Relationship

Given the apparent dependence on B, we screened a diverse library of commercially available B-containing compounds identified using SciFinder Scholar (called the BC series [for boron compound]) that shared some features and properties with 2APB (Figure S1). The BC molecules were screened against ATP-induced IL-1β release using primary mouse BMDMs as described above. Cells were primed with LPS and incubated with vehicle or molecules (BC1–24, Figure S1) before ATP stimulation. The effects of the molecules on IL-1β release were normalized to ATP-induced IL-1β release in the absence of any inhibitor (Figure 2A). Through this approach we identified analogs that were orders of magnitude more potent than 2APB at inhibiting IL-1β release (e.g., 2APB, IC_{50} = 67 μM; BC7 (**13**), IC_{50} = 1.2 μM; BC23 (**14**), IC_{50} = 2.3 μM; Figures 2B and 2C). Our preliminary qualitative structure-activity relationship (SAR) analysis identified the importance of the diarylborinic acid motif and an oxazaborine ring, with conformationally restricted analogs showing enhanced activity (Figure 2).

We then modified aspects of our lead BC molecules to improve activity and solubility, in addition to identifying the pharmacophore. Notably we modified the groups at each position of the oxazaborine ring (Figures 3A–3D), with atom numbering of the oxazaborine ring as shown in Figure 3E. A series of dioxa-, oxaza-, and diazaborines (novel boron compounds [NBC]1–31) based on the structures of BC7/23 were synthesized (for full details see Methods S1). In brief, 1,3-dicarbonyls were reacted with acetonitrile derivatives in the presence of a metal catalyst ($Zn(acac)_2$ or $SnCl_4$) to yield acetylated enaminones, by adapting previously reported methods (Veronese et al., 1986; Singh and Lesher, 1978). These intermediates were readily deacetylated by treating with K_2CO_3 (Veronese et al., 1986). Subsequent borylation of these enaminones using DPBA afforded the corresponding oxazaborine NBC molecules using a method similar to that previously described (Vasil'ev et al., 1992, Dorokhov et al., 1995) (Figure 3A). The synthesis of oxazaborine NBC18 used similar chemistry, except that the starting material was cyanoacetamide (Figure 3A). Dioxaborines were synthesized by directly borylating 1,3-dicarbonyls (Bally et al., 1965) (Figure 3C). Reaction of BC23 with a range of alkyl amines yielded diazaborines (Figure 3D) adapted from Vasil'ev et al. (2013).

(H) Chemical structures of B-containing compounds 2APB (**1**) and DPBA (**2**) and C-containing 2APB analogs (**3–12**).

(I and J) Mouse peritoneal macrophages were primed as before and stimulated with vehicle (0.5% DMSO) or inhibitor (**1–5**, 75 μM) before stimulation with ATP (5 mM, 20 min) (I). Mouse BMDMs were primed with LPS (1 μg mL^{-1}, 4 hr) and incubated with vehicle (0.5% DMSO) or molecules (NCI1–7, **6–12**, 40 μM) for 15 min before ATP stimulation (5 mM, 1 hr) (J).

In all cases supernatants were analyzed by ELISA. Data are presented as mean percentage of IL-1β release versus vehicle (DMSO) control + SEM (n = 3–9). *$p < 0.05$, **$p < 0.01$, ***$p < 0.001$, significant difference from 100% IL-1β release (Holm-Sidak corrected one-sample t test).

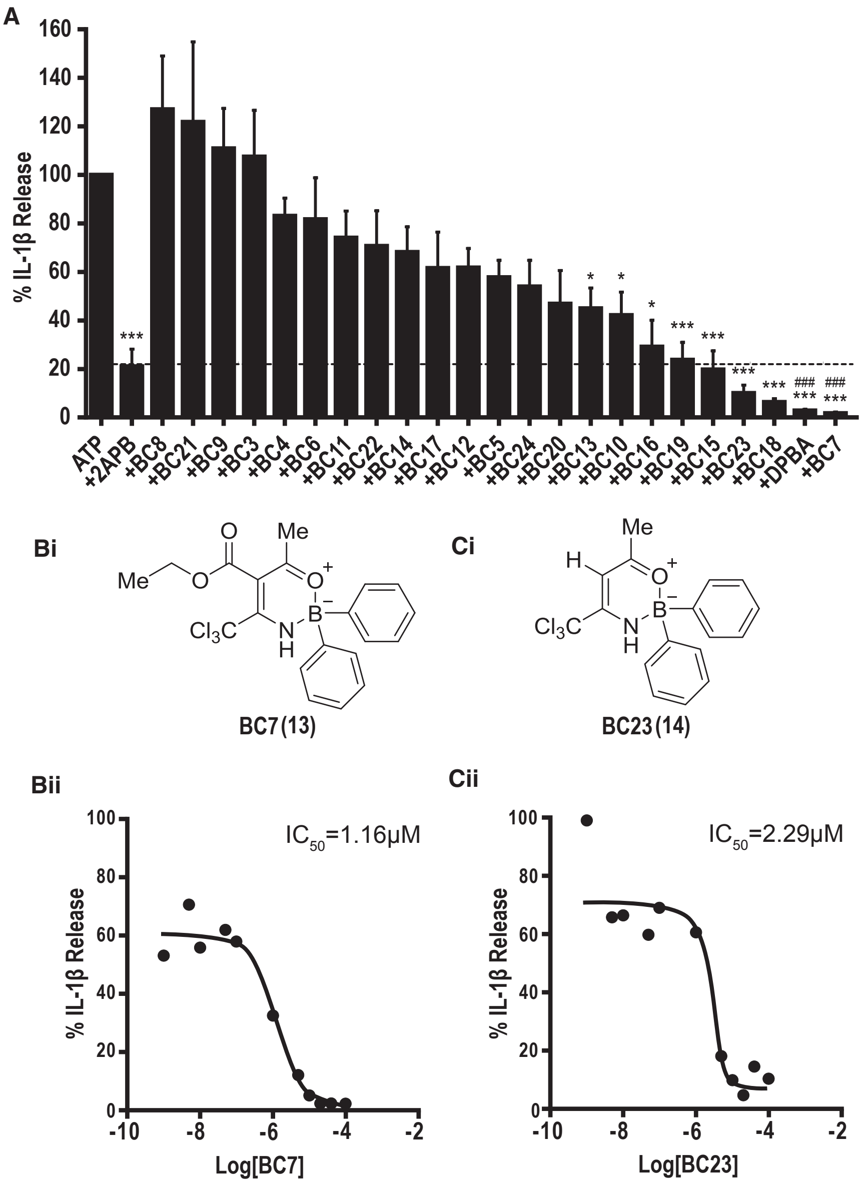

Figure 2. Identification of an Oxazaborine Ring in an Improved Pharmacophore

Primary mouse BMDMs were primed with LPS (1 μg mL^{-1}, 4 hr) and incubated with vehicle (0.5% DMSO) or molecules (BC1–24, Figure S1) at 40 μM for 15 min before ATP stimulation (5 mM, 1 hr). The effects of the molecules on IL-1β release were measured by ELISA and normalized to ATP-induced IL-1β release in the absence of any inhibitor (A). The chemical structures (i) and half-maximal inhibitory concentration curves (IC_{50}, ii) for BC7 (B) and BC23 (C) are also presented using a 3-parameter logistical sigmoidal model. Data are presented as mean percentage of IL-1β release versus vehicle (DMSO) control + SEM of at least 3 experiments. *p < 0.05, ***p < 0.001, significant difference from 100% IL-1β release (Holm-Sidak corrected one-sample t test). ###p < 0.001, significant improvement from 2APB treatment (Holm-Sidak corrected post hoc comparison).

Reaction of BC23 with ammonia did not give the expected diazaborine product, and only the dechlorinated compound containing a $CHCl_2$ group (NBC29) was isolated. cLogP and cLogS calculations were performed for BC7, BC23, NBC1–31, and NBC-EPPS (Figure 3B), and demonstrate that a number of potent NBC molecules (NBC6, 18, 24) have improved physicochemical properties compared with the original lead compounds BC7 and BC23. Furthermore, an experimental LogS value for our lead analog NBC6 was found to be −1.63 in MeOH (9.7 mg mL^{-1}) (data not shown). These data suggest that the NBC molecules are sparingly soluble in aqueous solution.

To screen the NBC series we used the human monocytic THP-1 cell line, since these cells would allow a higher throughput compared with the primary cells used above. Cells were primed with LPS and then treated with vehicle or NBC molecule (NBC1–31) at 10 μM for 15 min before activation of the inflammasome and IL-1β release with nigericin. The NBC molecule was present throughout nigericin stimulation. The effects of the molecules on IL-1β release were normalized to nigericin-induced IL-1β release in the absence of any inhibitor (Figure 3B). These data showed some additional features of the SAR (Figures 3B and 3E). Any substitution of the -CCl_3 group at position 4 (Figure 3E) was detrimental to bioactivity: its electronic character was important for inhibition of IL-1β release, confirmed by the substantially reduced activities of the isosteric ^{t}Bu (NBC28) and phenyl (NBC23) analogs. A *bis*-oxazaborine chelate (NBC8) was the only analog lacking the CCl_3 group that showed good bioactivity, which may be attributed to the extra BPh_2 group in the structure. Modifications of the oxazaborine ring (O-B-N bonding) to a dioxaborine (O-B-O) or diazaborine (N-B-N) ring structure was also detrimental to bioactivity. An oxazaborine ring structure was required, as the NBC6 enaminone intermediate prior to borylation (NBC6i, without B) was inactive (not shown). Modifications at the 5-position were generally tolerated, and typically molecules containing a carbonyl group-containing moiety showed enhanced bioactivity (Figures 3B and 3E). However, the ester NBC-EPPS analog containing the buffer 4-(2-hydroxyethyl)-1-piperazinepropanesulfonic acid (EPPS) was less active due to the substituent chain length either being too long or too hydrophilic. Deacetylation at position 5 (e.g., NBC6 cf. NBC5) leads to a loss in bioactivity (Figure 3E), so an acetyl group was preferentially retained in this position. Changing the methyl group at position 6 modulated bioactivity (Figure 3E), with substitution to the primary amide analog noticeably enhancing inflammasome inhibition (NBC6, **15**) (Figure 3B). Changing the primary amide to a secondary amide improved bioactivity as long as the substituents were hydrophobic (alkyl or aromatic) and the

A *Oxazaborines*

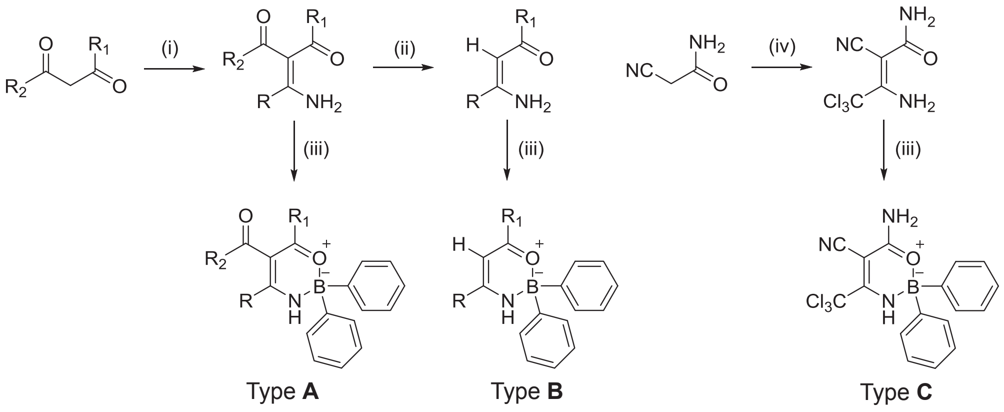

B

Type	Entry	R	R_1	R_2	cLogP	cLogS	Inhibition (%)	Signif.
A	BC-7 **(13)**	CCl_3	CH_3	OCH_2CH_3	4.99	-4.24	92.13	***
B	BC-23 **(14)**	CCl_3	CH_3	-	4.99	-4.22	91.55	***
A	NBC1	CCl_3	CH_3	CH_3	4.83	-4.26	47.94	ns
A	NBC2	CH_3	CH_3	OCH_2CH_3	3.54	-3.04	22.94	ns
B	NBC3	CH_3	CH_3	-	3.54	-3.02	4.61	ns
A	NBC4	CH_3	CH_3	CH_3	3.39	-3.06	9.08	ns
B	NBC5	CCl_3	NH_2	-	3.56	-3.85	77.72	***
A	NBC6 **(15)**	CCl_3	NH_2	CH_3	3.40	-3.89	99.1	***
A	NBC8	CH_3	$NHBPh_2$	CH_3	5.61	-4.48	78.49	***
A	NBC11 **(16)**	CCl_3	CH_3	$N(CH_3)_2$	4.37	-3.45	6.36	ns
A	NBC12	CCl_3	$NHCH_3$	CH_3	3.75	-3.81	8.54	ns
A	NBC13	CCl_3	NHPh	CH_3	5.46	-5.31	94.56	***
A	NBC14	CCl_3	$NHCH_2Ph$	CH_3	5.17	-5.13	93.02	**
A	NBC15	CCl_3	$NHCH_2CH_2Ph$	CH_3	5.60	-5.24	80.39	**
B	NBC16	CCl_3	$NHCH_2CH_2Ph$	-	5.76	-5.20	30.26	ns
A	NBC17	CCl_3	NHAd	CH_3	5.70	-6.14	89.28	**
C	NBC18	CCl_3	NH_2	CN	3.24	-4.12	93.84	***
A	NBC19 **(17)**	CCl_3	NHCy	CH_3	5.96	-5.38	100.00	***
A	NBC20	CCl_3	NHPyr	CH_3	8.49	-9.77	28.47	ns
A	NBC22	CCl_3	NHPy	CH_3	5.11	-4.52	61.00	*
A	NBC23	Ph	CH_3	CH_3	4.68	-3.89	-1.29	ns
A	NBC24	CCl_3	NH_2	NH_2	2.32	-3.52	94.11	***
A	NBC25	CCl_3	CH_3	$OC(CH_3)_3$	5.73	-4.73	89.64	***
A	NBC26	SCH_2CH_3	CH_3	$OC(CH_3)_3$	4.98	-4.24	-7.16	ns
B	NBC28	$C(CH_3)_3$	CH_3	-	4.78	-3.62	-12.00	ns
B	NBC29	$CHCl_2$	CH_3	-	4.18	-3.71	25.22	ns
A	NBC-EPPS	CCl_3	CH_3	EPPS	1.07	-2.73	26.96	ns

C *Dioxaborines*

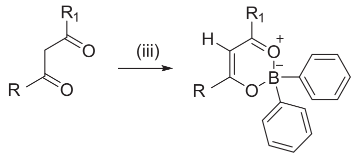

Entry	R	R_1	cLogP	cLogS	Inhibition (%)	Signif.
**NBC7**	CH_3	NH_2	2.11	-2.20	15.47	ns
**NBC9**[a]	CCl_3	NH_2	3.56	-3.41	42.7	ns
**NBC10**	CH_3	CH_3	3.54	-2.58	6.36	ns

D *Diazaborines*

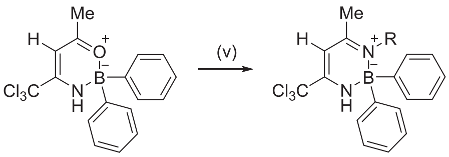

Entry	R	cLogP	cLogS	Inhibition (%)	Signif.
**NBC27 (18)**	CH_3	4.71	-3.97	7.88	ns
**NBC30 (19)**	CH_2CH_3	5.11	-4.27	7.62	ns
**NBC31**	$CH_2CH_2CH_3$	5.57	-4.54	15.35	ns

E

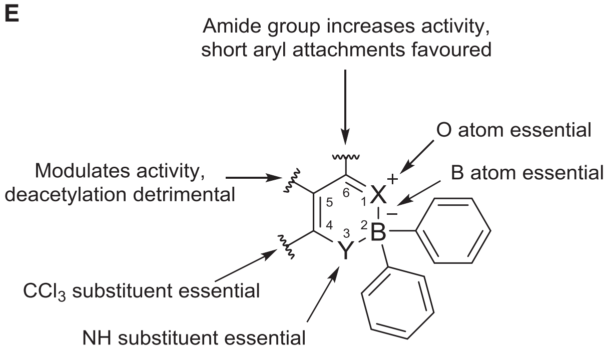

Fi

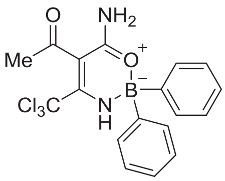

NBC6 **(15)**

Fii

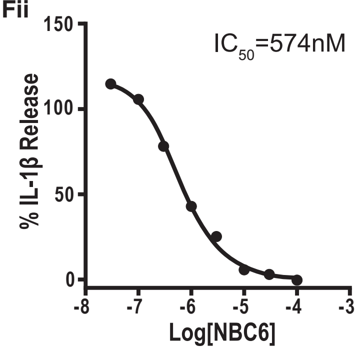

(legend on next page)

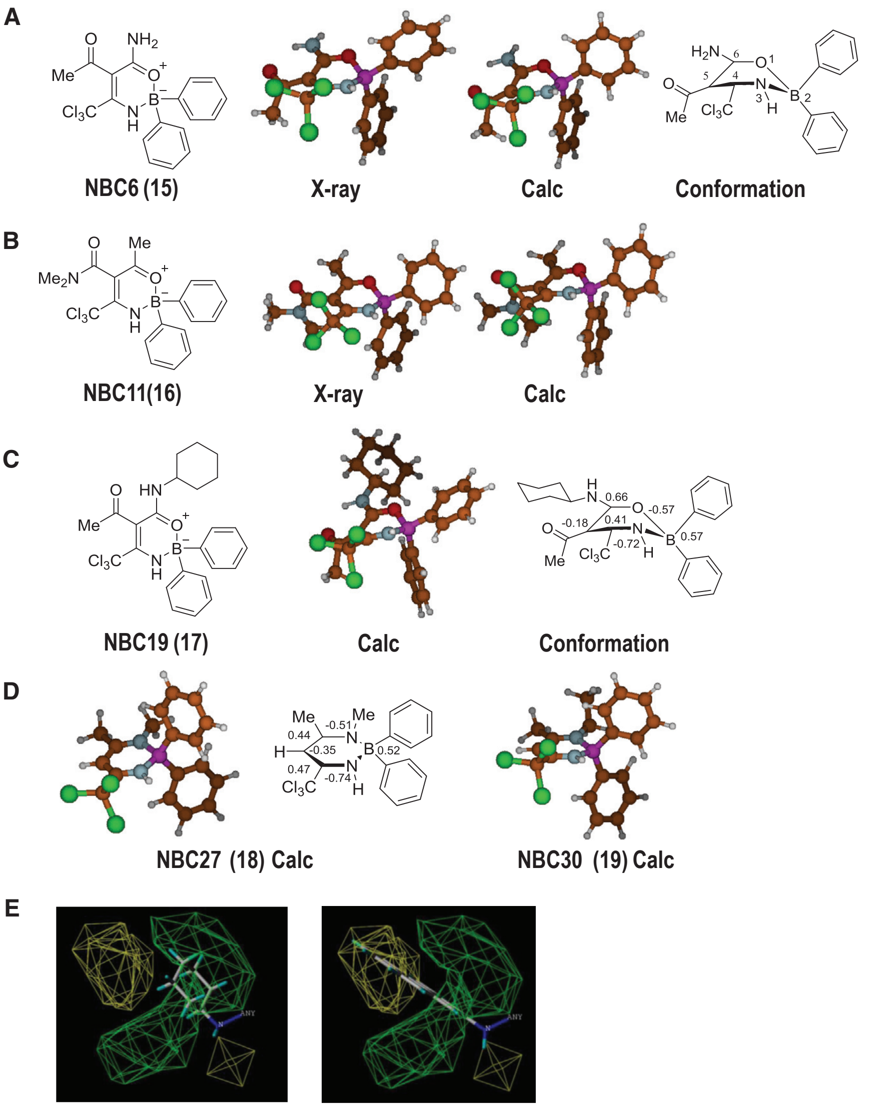

Figure 4. X-Ray Crystallography and Computational Modeling of NBCs

(A and B) Crystal (X-ray) and predicted structure (Calc) of NBC6 (**15**) (A) and NBC11 (**16**) (B), calculated at the M06-L/6-31G* level of theory. The ring is boat-like in conformation (atom numbering shown in A).

(C) Computed structure and Mulliken partial charges on ring atoms of oxazaborine NBC19 (**17**).

(D) Computed structures of NBC27 (**18**) and NBC30 (**19**) illustrating the planarity of the diazaborine ring and Mulliken partial charges of NBC27 and NBC30.

(E) Steric field arising from topomer CoMFA of 24 oxazaborine compounds, superimposed on structures of (left) NBC19 and (right) NBC20.

chain length of the substituent was not too short (R_1 = Me, NBC12) or too bulky (R_1 = pyrene, NBC22). Full substitution of the primary amide (NBC6) to the *N*,*N*-dimethyl tertiary amide derivative (NBC11, **16**) lost bioactivity, potentially due to a change in chelation (see X-ray in Figures 4A and 4B). It was interesting to note that secondary amide derivatives containing either phenyl (NBC13) or cyclohexyl (NBC19, **17**) substitutions are both active, showing that the ring can be either unsaturated or saturated. In summary, an oxazaborine scaffold and a CCl_3 group on the 4-position was required to inhibit IL-1β release (Figure 3E). From the screen of NBC molecules, NBC6 was most potent and more drug-like compared with leads BC7/23, and further analysis revealed increased potency with an IC_{50} of 574 nM (Figure 3F).

X-Ray Crystallography and Computational Modeling of NBCs

When borylating the enaminone intermediates using DPBA, the oxazaborine product could adopt a number of different B chelate structures. For example, NBC6 and NBC11 could chelate to B through the NH of the enamine and either the amide or ketone C=O. To determine the structures of the chelates in the solid phase, crystals of NBC6 and NBC11 were grown in *n*-hexane/toluene (1:1) and X-ray crystallographic analysis was undertaken. The *R* factors obtained for NBC6 and NBC11 were 4.08% and 2.83%, respectively. The B atom lies out of the ring plane in a boat-envelope conformation in both oxazaborine structures, whereas the other atoms in the heterocycle are planar and are involved in a π-electron conjugated system (Figure 4A) as previously reported for other oxazaborines (Josefíka et al., 2012; Mikyseka et al., 2017). NBC6 is chelated to B through O/N chelation of the amide C=O and enamine NH (Figure 4A),

Figure 3. Refinement of the Structure-Activity Relationship

(A) Pathway for oxazaborine syntheses. The method for the synthesis of the oxazaborine compounds are described as types A, B, and C. (i) RCN, $Zn(acac)_2$/$SnCl_4$, dichloromethane/toluene, room temperature to 80°C, 3–16 hr; (ii) $K_2CO_{3(sat)}$, EtOH, room temperature, 24 hr; (iii) DPBA, tetrahydrofuran (THF), 50°C, 16 hr; (iv) Cl_3CCN, NaOAc, EtOH, room temperature, 16 hr (A).

(B–D) Table of oxazaborines synthesized with structure type (A, B or C) identified (B). Ad, adamantyl; Cy, cyclohexyl; Pyr, pyrene; Py, pyridinyl; EPPS, 4-(2-hydroxyethyl)piperazine-1-propanesulfonic acid. Also shown in (B) to (D) is the percentage of inhibition of IL-1β release from LPS and nigericin-treated THP-1 cells with 10 μM inhibitor and the calculated cLogP and cLogS values for each compound. (C) Pathway for dioxaborine syntheses. (iii) DPBA, THF, 50°C, 16 hr. [a]NBC9 was isolated as a by-product during NBC5 synthesis. (D) Pathway for diazaborine synthesis. (v) RNH_2, THF, 50°C, 24 hr.

(E) Summary of SAR analysis of NBCs.

(F) Half-maximal inhibitory concentration curve (IC_{50}) for NBC6 (Fi) is presented using a 3-parameter logistical sigmoidal model (n = 6) (Fii).

*p < 0.05, **p < 0.01, ***p < 0.001, significant difference from 100% IL-1β release (Holm-Sidak corrected one-sample t test), n = 4. ns, not significant.

whereas NBC11 is chelated to B through O/N chelation of the ketone C=O and enamine NH (Figure 4B). Thus full substitution of the primary amide (NBC6) to an *N*,*N*-dimethyl tertiary amide (NBC11) has induced a change in B chelation, which could explain the large difference in observed bioactivity between these two oxazaborines (Figure 3B).

Computational modeling was applied to further investigate the impact of the shape and electronic properties of the NBCs on the bioactivity and observed SAR. All of the compounds were energy optimized at the M06-L/6-31G* level of quantum mechanics (Zhao and Truhlar, 2006). Consistent with the X-ray crystal structures, computational modeling predicted that the rings in the oxazaborines adopted boat-envelope conformations (NBC6 [Figure 4A] and NBC11 [Figure 4B]). Indeed, the agreement in ring pucker of the 6-membered ring between calculation and the X-ray structures was very good: for NBC6, the mean unsigned ring pucker torsion $<\nu>$ differs by 2.1° while for NBC11 the difference is 5.7° (Table S1). There was good agreement with experiment in the use of M06 functionals to model coordinate B-N bond lengths and bond enthalpies of methyl-substituted aminoboranes (Janesko, 2010). For NBC6 and NBC11, the average experimental pucker $<\nu>$ was 25.4° and 21.1°, respectively (Table S1). This indicates significant deviation from planarity; the greatest pucker for both rings centered around the B atom, with a ν_5 angle of $-45.4°$ and $-37.3°$, respectively (Table S1). Computed bond distances within the ring also reproduce the experiment well, with a maximum deviation from the crystallographic values of 0.02 Å for NBC6 and 0.01 Å for NBC11 (Table S1). The largest difference in ring bond distance between NBC6 and NBC11 is found for the C_5-C_6 bond, with predicted and experimental distances agreeing on a 0.05-Å larger bond distance in NBC6 (Table S1). In contrast, energy optimization of the NBC diazaborine compounds, containing the N-B-N motif, predict at the M06-L/6-31G* level a planar diazaborine ring (e.g., NBC27 [**18**], and NBC30 [**19**], Figure 4D). The planarity is reflected by a mean unsigned pucker angle $<\nu>$ of 3.3° and 11.4° for NBC27 and NBC30, respectively (Table S1), distinctly lower than the values for NBC oxaborines, which exceed 20°. Furthermore, the N-B-N angle in these compounds are 112.5° and 111.2° for NBC27 and NBC30, respectively, compared with O-B-N angles of 102.6° and 104.0° in the NBC6 and NBC11 X-ray structures (θ, Table S1). These geometric features reflect the more aromatic character of the diazaborine systems.

Further insight into electronic distribution was obtained from Mulliken population analysis: firstly we note that the B atom is predicted to possess a positive partial charge in all of the NBC compounds analyzed (Figures 4C and 4D; Table S1). The charge on the B atom is on average 0.59 *e* for the 24 O-B-N compounds, 0.60 *e* for 3 O-B-O compounds, and somewhat less for the 3 N-B-N compounds, with an average value of 0.51 *e*. This reflects the low Pauling electronegativity of the B atom, reported as 2.04 compared with values of 3.04 for N and 3.44 for O (Allen, 1989). Thus, although we traditionally represent 4-coordinate B atoms with a formal negative charge (e.g., Figure 3), quantum chemical analysis predicts that the B atom in the heterocycle of these NBC compounds possesses a partial positive charge. The magnitude of the partial atomic charge on carbon C_6, $q(C_6)$ is higher for NBC6 and NBC19 (>0.6 *e*) compared with less active compounds NBC11, NBC27, and NBC30 (<0.5 *e*, Table S1). For oxazaborines, a higher value of $q(C_6)$ appears to be due to the presence of an amido substituent at C_6. The correlation of $q(C_6)$ and observed activity of the compounds is somewhat modest, with a correlation coefficient r^2 of 0.5 (a similar correlation is found for the C_5-C_6 bond distance); this reflects the influence of other factors, in particular steric constraints on substituents. 3D-QSAR was performed using the topomer comparative molecular field analysis (CoMFA) method (Cramer, 2003), based on oxazaborines NBC1–6, 8, 11–20, 22–26, and 28–29 compounds (r^2 of 0.71). Again, our analysis highlights the steric volume required for activity at position 6 (Figure 4E), and indicates the complementarity of the cyclohexyl substituent of NBC19 compared with the weakly active NBC20 compound, which presents a bulkier pyrene group at C_6 (Figure 4E). In summary, the density functional calculations agree well with crystallographically determined structures and indicate a shape and electronic character of the oxazaborine ring that is distinct from the planar, aromatic diazaborine ring, suggesting that these features are responsible for the activity of the oxazaborines. Within the oxazaborine series, there is evidence of a specific steric constraint on substituents at position 6 of the ring.

Mechanism of Action

We measured the effects of 2APB, BC7, BC23, and NBC6 on ASC speck formation following ATP stimulation. Immortalized (i)BMDMs transduced with a lentiviral vector to express ASC-mCherry (Daniels et al., 2016) were treated with LPS and then stimulated with ATP for between 30 and 45 min with ASC speck formation measured as described previously (Daniels et al., 2016). 2APB was an effective inhibitor of ASC speck formation, as were BC23 and NBC6 (Figure 5A). Additionally, we showed that 2APB is not a direct inhibitor of caspase-1. Recombinant caspase-1 was incubated with vehicle, YVAD, or 2APB before addition of the fluorogenic substrate Z-YVAD-AFC. Caspase-1 activity was measured 2 hr later. Under these conditions 2APB had no effect on caspase-1 activity while YVAD caused complete inhibition (Figure 5Bi). We also used a hypotonic THP-1 cell lysate assay to measure the effects of 2APB on caspase-1 activity. 2APB was added to the cells just prior to, or following, lysis in hypotonic buffer. The lysate was incubated with the caspase substrate Z-YVAD-AFC, which in addition to caspase-1 would also be cleaved by caspase-4 and -5, and caspase activity was measured 2 hr later. 2APB had no effect on caspase-1 activity under these conditions, whereas caspase-1 activity was completely inhibited by YVAD or high K^+ concentration (Figure 5Bii). An important step in the activation of NLRP3 is K^+ efflux from the cell (Munoz-Planillo et al., 2013). It was reported recently that 2APB does not inhibit K^+ efflux (Katsnelson et al., 2015), so the effect of 2APB must be downstream of K^+ efflux and before caspase-1 activity. Recently additional mechanisms of NLRP3 activation have been reported, including by the small molecule imiquimod, which is suggested to be independent of K^+ efflux (Gross et al., 2016). In LPS-primed primary BMDMs, NBC6 also inhibited imiquimod-induced IL-1β release (Figure 5C). We also tested whether NBC6 could inhibit IL-1β secretion through the non-canonical NLRP3 pathway. Priming with the TLR2 agonist Pam3CSK4 followed by LPS transfection stimulates the activation of NLRP3 via the non-canonical

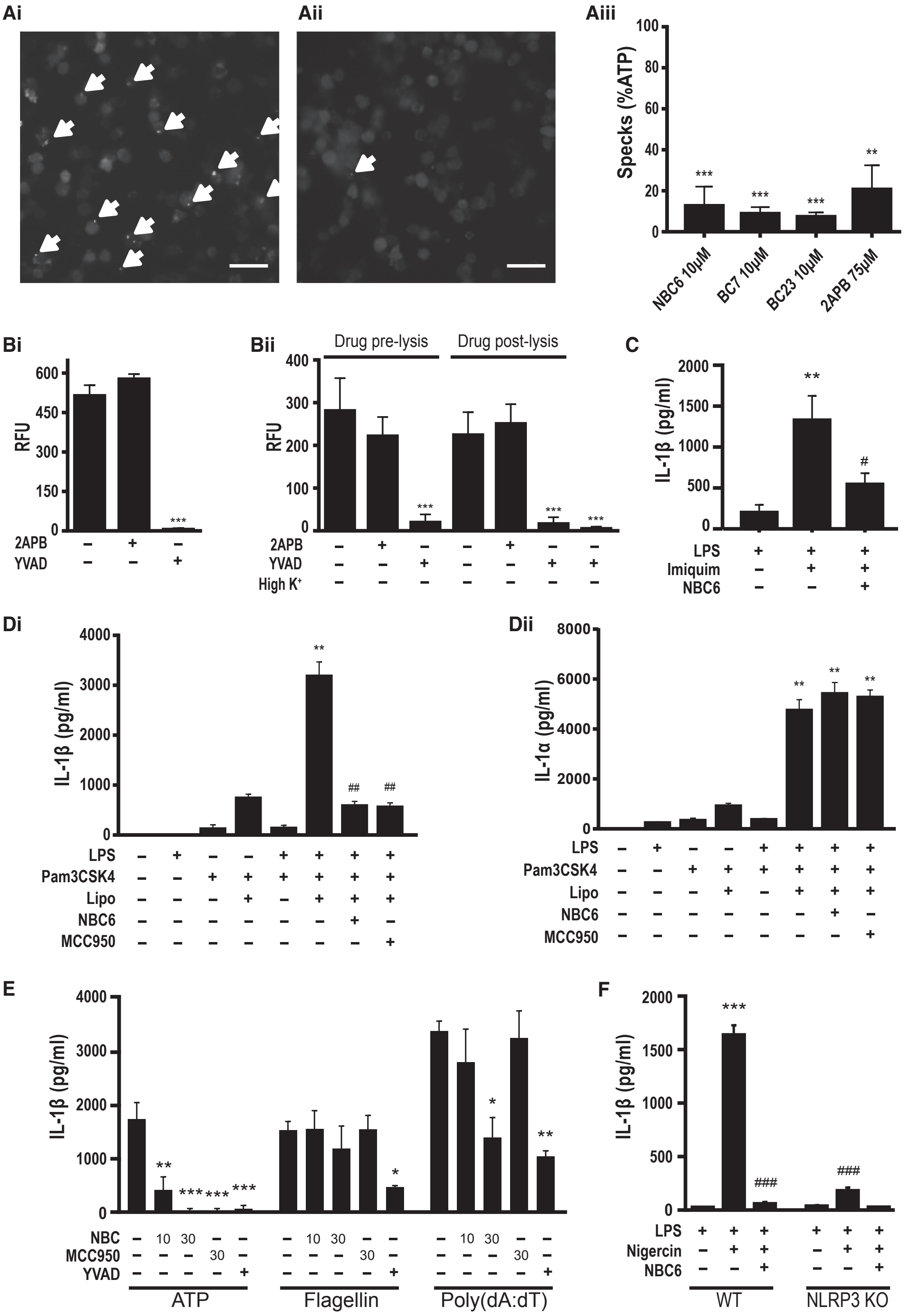

Figure 5. NBCs Are Effective NLRP3 Inflammasome Inhibitors

(A) The effects of 2APB, BC7, BC23, and NBC6 on ASC speck formation following ATP stimulation were measured. iBMDMs stably expressing ASC protein conjugated to mCherry were primed with LPS (1 μg mL^{-1}, 2 hr), then pre-treated with selected drug (indicated concentration, 15 min) before stimulation with ATP

(legend continued on next page)

caspase-11-dependent pathway (Kayagaki et al., 2013). Primary BMDMs were primed with Pam3CSK4 followed by transfection with LPS (Kayagaki et al., 2013). Neither NBC6 nor MCC950 inhibited the release of IL-1α (Figure 5Dii), which occurs due to caspase-11-dependent pyroptosis independently of NLRP3 (Kayagaki et al., 2011). However, NBC6 and, as previously reported (Coll et al., 2015), MCC950 did inhibit the release of IL-1β (Figure 5Di). These data suggest that NBC6 can also inhibit NLRP3 via the non-canonical pathway. To determine whether the NBCs were selective inhibitors of NLRP3-dependent IL-1β release, we tested their effects against other well-characterized inflammasomes in primary wild-type (WT) and NLRP3 knockout (KO) BMDMs. LPS-primed WT BMDMs were treated with ATP in the absence and presence of NBC6 (10 and 30 μM), the established NLRP3 inhibitor MCC950 (Coll et al., 2015) (30 μM), and the caspase-1 inhibitor YVAD (100 μM). Under these conditions, all inhibitors inhibited the release of IL-1β (Figure 5E). In NLRP3 KO BMDMs a similar format was followed except that NLRC4 inflammasome activation was induced by transfection of *Salmonella typhimurium* flagellin, whereby this time 10 and 30 μM NBC6 and 30 μM MCC950 had no effect (Figure 5E). The same format was followed for AIM2 inflammasome activation whereby LPS-primed NLRP3 KO BMDMs were transfected with poly(dA:dT). Again 10 μM NBC6 and 30 μM MCC950 had no effect and YVAD inhibited IL-1β release, as did 30 μM NBC6 (Figure 5E). These data suggest that NBC6 selectively inhibits NLRP3 at low doses but may also be effective against other inflammasomes at higher doses. To further establish that NBC6 inhibits NLRP3 across cell types, neutrophils were isolated from WT and NLRP3 KO murine bone marrow and primed with LPS followed by nigericin treatment in the presence or absence of 10 μM NBC6. From this we observed complete inhibition of NLRP3-dependent IL-1β release from NBC6-treated neutrophils (Figure 5F).

We next compared the toxicity of NBC6 with that of MCC950 in kidney (HEK293) and liver (HepG2) cell lines. Neither drug showed any toxicity up to 24 hr of incubation (Figure 6A). To further refine the mechanism of action of NBCs on NLRP3, we sought to determine the reversibility of NBC inhibition of NLRP3-dependent IL-1β release. iBMDMs were primed with LPS and then incubated with the reversible caspase-1 inhibitor YVAD, the irreversible NLRP3 inhibitor 3,4-methylenedioxy-β-nitrostyrene (MNS; He et al., 2014), NBC6, BC23 (from the BC series), and 2APB for 15 min before 3 washes over 15 min to remove unbound drug. Cells were then stimulated with ATP (5 mM) to activate NLRP3-dependent IL-1β release. As expected, YVAD washed out and MNS did not. The effects of NBC6 and BC23 resembled that of MNS and were irreversible over the time course of the experiment (Figure 6B). Interestingly 2APB was reversible (Figure 6B). To test whether the NBC molecules could inhibit NLRP3-dependent inflammation *in vivo*, we used a previously reported model of peritonitis (Coll et al., 2015). Previously, mice injected intraperitoneally with LPS showed increased IL-1β in the lavage fluid that was inhibited by the NLRP3 inhibitor MCC950 (Coll et al., 2015). Thus we injected WT and NLRP3 KO mice with LPS. Separate groups of animals receiving LPS were also given a dose of MCC950 or NBC13, chosen because it had similar potency to NBC6 at inhibiting IL-1β release and was soluble in corn oil, which was used to deliver the drug. LPS induced an increase in IL-1β in the lavage and plasma in WT mice but not NLRP3 KO mice, supporting the NLRP3 dependence of this response (Figure 6C). MCC950, as expected, also inhibited LPS-induced IL-1β production in the peritoneum, as did NBC13, suggesting that NBCs are effective NLRP3 inhibitors *in vivo* (Figure 6Ci). LPS also caused an increase in plasma IL-1β, which was also absent in NLRP3 KO mice and was completely inhibited by MCC950 (Figure 6Cii). NBC13 significantly inhibited LPS-induced plasma increases in IL-1β but was not quite as effective as MCC950, possibly due to reduced exposure or potency (Figure 6Cii). Both NBC13 and MCC950 significantly inhibited the production of the related cytokine IL-1α in the plasma (Figure 6Ciii). Together, these data show that the NBCs are effective inhibitors of the NLRP3 inflammasome and can also target NLRP3-dependent inflammation *in vivo*.

(5 mM, 30–45 min) under live microscopy. Formation of ASC specks (examples indicated by white arrows, Ai [no drug], Aii [plus NBC6]) were quantified (Aiii) and presented as mean percentage of specks counted versus vehicle + SEM (n = 5–6). **$p < 0.01$, ***$p < 0.001$, significant difference from 100% speck formation (Holm-Sidak corrected one-sample t test, n = 5–6). Scale bars, 20 μm.

(B) Recombinant caspase-1 (10 U mL^{-1}) was incubated with 0.5% DMSO, YVAD (100 μM), or 2APB (75 μM) before addition of the fluorogenic substrate Z-YVAD-AFC. Caspase-1 activity was measured 2 hr later (Bi) (***$p < 0.001$, significant difference from vehicle control, Holm-Sidak corrected post hoc comparison, n = 4). Hypotonic THP-1 cell lysate assay was also used to measure the effects of 2APB on caspase-1 activity. 2APB (75 μM) was added to the cells just prior to, or following, lysis in hypotonic buffer. The lysate was incubated with Z-YVAD-AFC and caspase-1 activity measured 2 hr later (Bii). YVAD or high K^+ concentration were included as controls (Bii) (***$p < 0.001$, significant difference from relevant lysis vehicle control, Holm-Sidak corrected post hoc comparison, n = 4).

(C) LPS-primed (1 μg mL^{-1}, 4 hr) mouse primary BMDMs were treated with NBC6 (10 μM) or vehicle (DMSO) 15 min prior to 1 hr treatment with small-molecule NLRP3 activator imiquimod (70 μM) or DMSO control. Imiquimod significantly induced IL-1β release (**$p < 0.01$) and this was inhibited by NBC6 treatment ($^{\#}p < 0.05$, Holm-Sidak corrected post hoc comparison, n = 4).

(D) Mouse primary BMDMs were primed with Pam3CSK4 (100 μg mL^{-1}, 4 hr) followed by 15 min NBC6 (1 μM), MCC950 (1 μM), or vehicle, then treated with intracellular LPS (2 μg mL^{-1}, transfected with Lipofectamine 3000, 24 hr) or Lipofectamine alone (**$p < 0.01$, significant induction of IL-1β [Di] or IL-1α [Dii] release versus Lipofectamine-alone control; $^{\#\#}p < 0.01$, significant inhibition of IL-1β release; Holm-Sidak corrected post hoc comparison, n = 4).

(E) Mouse primary BMDMs were primed with LPS (1 μg mL^{-1}, 4 hr) followed by 15 min NBC6 (10 and 30 μM), MCC950 (30 μM), YVAD (100 μM), or vehicle, then treated with canonical NLRP3 activator ATP (5 mM, 1 hr), NLRC4 activator (flagellin, 667 ng mL^{-1}, transfected with Lipofectamine 3000), or AIM2 activator (poly(dA:dT), 667 ng mL^{-1}, transfected with Lipofectamine 3000) (*$p < 0.05$, **$p < 0.01$, ***$p < 0.001$, significant inhibition of IL-1β release, Holm-Sidak corrected post hoc comparison, n = 3).

(F) Mouse primary bone marrow neutrophils from WT and NLRP3 KO mice (n = 4) were primed with LPS (1 μg mL^{-1}, 2 hr), then NBC6 (10 μM) was added 15 min prior to the addition of nigericin (10 μM), which significantly induced IL-1β release (***$p < 0.001$), which was inhibited by NBC6 treatment ($^{\#\#\#}p < 0.001$, Holm-Sidak corrected post hoc comparison).

Data are presented as the mean + SEM.

CellPress

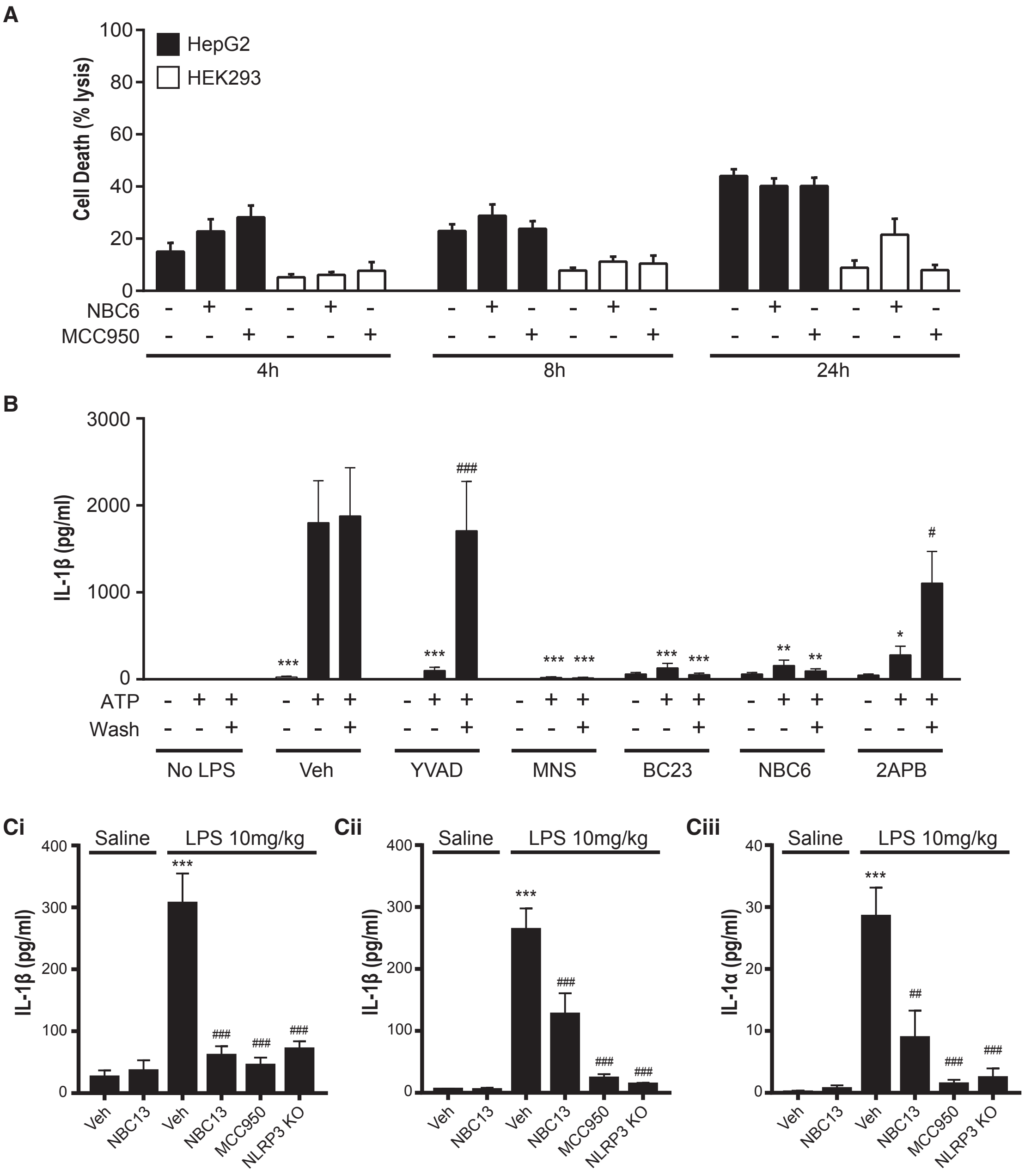

Figure 6. NBCs Are Effective against NLRP3 *In Vivo*

(A) HEK293 or HepG2 cells were treated with NBC6 (10 μM), MCC950 (10 μM), or DMSO for 4 hr, 8 hr, and 24 hr. Cell death was measured by lactate dehydrogenase release and expressed as percentage lysis control. No significant effects were observed (two-way repeated-measures ANOVA).

(B) LPS-primed (1 μg mL^{-1}, 2 hr) iBMDMs were pre-treated with drugs (BC23, NBC6, 30 μM; MNS, 100 μM; YVAD, 100 μM; 2APB, 75 μM) or vehicle (DMSO) in serum-free media for 15 min and washed 3 times, before inflammasome activation was initiated by adding ATP (5 mM) for 1 hr. IL-1β release was measured by ELISA (*$p < 0.05$, **$p < 0.01$, ***$p < 0.001$, significant inhibition of IL-1β release compared with vehicle-ATP control; $^{\#}p < 0.05$, $^{\#\#\#}p < 0.001$, significant effect of washing compared with no-wash drug-ATP control, Holm-Sidak corrected post hoc comparison, n = 5–6).

(C) C57BL/6 and NLRP3 KO mice (n = 6) were injected intraperitoneally with LPS (10 mg kg^{-1}, 3 hr). Separate groups of WT animals receiving LPS were also given a 50 mg kg^{1} dose of MCC950 or NBC13. IL-1β in peritoneal lavage (Ci) and plasma (Cii) was measured by ELISA. IL-1α in plasma was measured by ELISA (Ciii). ***$p < 0.001$, significant difference from saline vehicle control; $^{\#\#}p < 0.01$, $^{\#\#\#}p < 0.001$, significant difference from LPS vehicle group (Holm-Sidak corrected post hoc comparison).

Data are presented as the mean + SEM.

Ca^{2+}-Independent Effects of the NBCs

We recently reported that the fenamates were effective inhibitors of the NLRP3 inflammasome due to inhibition of Cl^{-} efflux through the volume-regulated anion channel (VRAC) (Daniels et al., 2016). Thus we tested the effects of BC23, BC7, and NBC6 on VRAC. VRAC currents measured by whole-cell patch

clamp in LPS-primed iBMDMs were induced by hypotonicity. BC7, BC23, and NBC6 had no effect on VRAC (Figure 7A). As described above, 2APB is known to modulate Ca^{2+} homeostasis. Thus we tested the ability of 2APB and NBC6 to modify intracellular Ca^{2+} in LPS-treated iBMDMs stimulated with ATP. The cells were pre-treated with doses of the inhibitors maximal for blocking IL-1β release (i.e., 2APB 75 μM, NBC6 30 μM). The cells were then stimulated with 100 μM ATP (submaximal for P2X7 but saturating for other purinergic receptors) to induce increases in $[Ca^{2+}]_i$, which were measured using the ratiometric Ca^{2+} indicator Fura-2 (Figures 7B–7D). ATP induced a transient increase in $[Ca^{2+}]_i$ that was inhibited by 2APB but was not blocked by NBC6 (Figures 7B–7E), suggesting that the NBC compounds did not affect $[Ca^{2+}]_i$ changes dependent upon $InsP_3$ receptor activation. iBMDM cells were then treated with LPS and stimulated with 5 mM ATP to activate the P2X7 receptor and induce activation of NLRP3. Under these conditions there was a marked and sustained increase in $[Ca^{2+}]_i$ (Figures 7F and 7G). Inhibitor (2APB 75 μM, NBC6 30 μM) applied to the cells 3 min after ATP did not affect $[Ca^{2+}]_i$, again supporting that the effects of 2APB and the NBC compounds are acting independently of effects on Ca^{2+} (Figures 7F and 7G). To correlate with this Ca^{2+} experiment, inhibitors were added to the iBMDMs following ATP stimulation as described above and effects on IL-1β release were measured 1 hr later by ELISA (Figures 7H and 7I). Adding drug after stimulation of the large Ca^{2+} increase inhibited inflammasome activation and IL-1β release as effectively as the pre-incubation (Figures 7H and 7I). Together, these data suggest that the inhibitors are acting independently of any effects on Ca^{2+} and that the effect on Ca^{2+} for the NBC compounds is not significant.

DISCUSSION

The NLRP3 inflammasome contributes to inflammatory diseases and is therefore an important therapeutic target (Lamkanfi and Dixit, 2012). Increasing recognition of the contribution of NLRP3 to disease has led to efforts to develop small-molecule inhibitors (Baldwin et al., 2016). Here we report the development of a unique B-based pharmacophore that inhibits NLRP3-dependent inflammation in both *in vitro* and *in vivo* models. Boron is an unusual element to be present in drug leads; bortezomib (Velcade) is the only B-containing drug used clinically. After establishing that B was essential for inhibition through screening of carbon analogs, we synthesized a range of new B-based inhibitors of NLRP3 derived from the early leads 2APB, BC7, and BC23, the most potent compound having an IC_{50} value of 574 nM for the inhibition of release of IL-1β from THP-1 monocytes.

During the preparation of the oxazaborines, for the synthesis of acetylated enaminone intermediates, we found that the choice of the metal catalyst was critical; $Zn(acac)_2$ was amenable with good electrophiles (e.g., trichloroacetonitrile and benzonitrile); however, the stronger catalyst $SnCl_4$ was required with non-electrophilic, weakly activating nitriles (e.g., acetonitrile and *tert*-butyl acetonitrile). The oxazaborines synthesized herein can be handled easily at room temperature, in contrast to the facile hydrolysis observed for 2APB (Hofer et al., 2013). Molecular modeling calculations agreed well with the X-ray crystal structures of NBC6/11, demonstrating the robustness of predictions using quantum mechanics. This is the first time that biological screening of oxaza-, dioxa-, and diazaborines has been reported. The SAR of the 31 NBC molecules revealed interesting key features required for bioactivity, with the oxazaborine ring and CCl_3 group being essential pharmacophores for NLRP3 inflammasome inhibition.

NLRP3 is composed of three domains: an N-terminal pyrin domain for homotypic interaction with the pyrin domain of the adaptor ASC, a central NACHT domain that binds nucleotides, and a C-terminal leucine-rich repeat domain that senses the PAMPs or DAMPs. The mechanisms regulating the activation of NLRP3 are currently the focus of a major research effort in the field and are still being elucidated. There is very limited evidence for an interaction between PAMP/DAMP and NLRP3; instead these activating stimuli activate a common pathway dependent upon K^+ efflux (Munoz-Planillo et al., 2013). Recently, the protein NEK7 has been identified as an interacting partner of NLRP3 required for its activation (Schmid-Burgk et al., 2016; Shi et al., 2016), and this interaction is also downstream of K^+ efflux (He et al., 2016). In addition, ubiquitination and deubiquitination are also becoming established as essential steps (Lopez-Castejon et al., 2013; Juliana et al., 2012; Py et al., 2013). There has also been substantial literature to support an involvement of Ca^{2+} in inflammasome activation. Many of the studies reporting Ca^{2+} dependence of NLRP3 activation have been based on, or involved the use of 2APB as an inhibitor of Ca^{2+} signaling (Lee et al., 2012; Murakami et al., 2012; Compan et al., 2012; Rossol et al., 2012), or intracellular Ca^{2+} chelators such as BAPTA-AM (Brough et al., 2003). However, there is now evidence that both of these experimental manipulations may inhibit the inflammasome independently of effects on Ca^{2+} (Katsnelson et al., 2015). Further evidence for an effect independent of Ca^{2+} is provided in Figure 1, and is related to the work of Dobrydneva and Blackmore (2001) on store-operated Ca^{2+} entry. While they showed that DPBA inhibits Ca^{2+} entry (and IL-1β release presented here), DPTTF, which had no effect on IL-1β release in our study, inhibited Ca^{2+} entry as effectively as DPBA (Dobrydneva and Blackmore, 2001). Such data further support that the effects of 2APB on IL-1β processing and release are independent of Ca^{2+}. Here we also show that while 2APB effectively inhibits increased $[Ca^{2+}]_i$ in response to 100 μM ATP, NBC6 does not, even at concentrations supra-maximal for the inhibition of IL-1β secretion (Figure 7). Furthermore, addition of 2APB, or NBC6 after the addition of 5 mM ATP, an NLRP3-activating stimulus, did not modify $[Ca^{2+}]_i$ dynamics, but did still inhibit IL-1β secretion following this protocol (Figure 7). These data suggest strongly that the effects of 2APB on IL-1β release are independent of Ca^{2+}, and that we have deselected this property in our NBC molecules. This effectively provides a new and unique chemical scaffold for the development of NLRP3-inhibiting drugs that do not have the potentially harmful off-target effects on Ca^{2+} homeostasis.

SIGNIFICANCE

Excellent evidence now points toward NLRP3 as an important therapeutic target for multiple major diseases (Guo et al., 2015). There are no drugs available clinically that specifically target NLRP3, although we (Daniels et al., 2016)

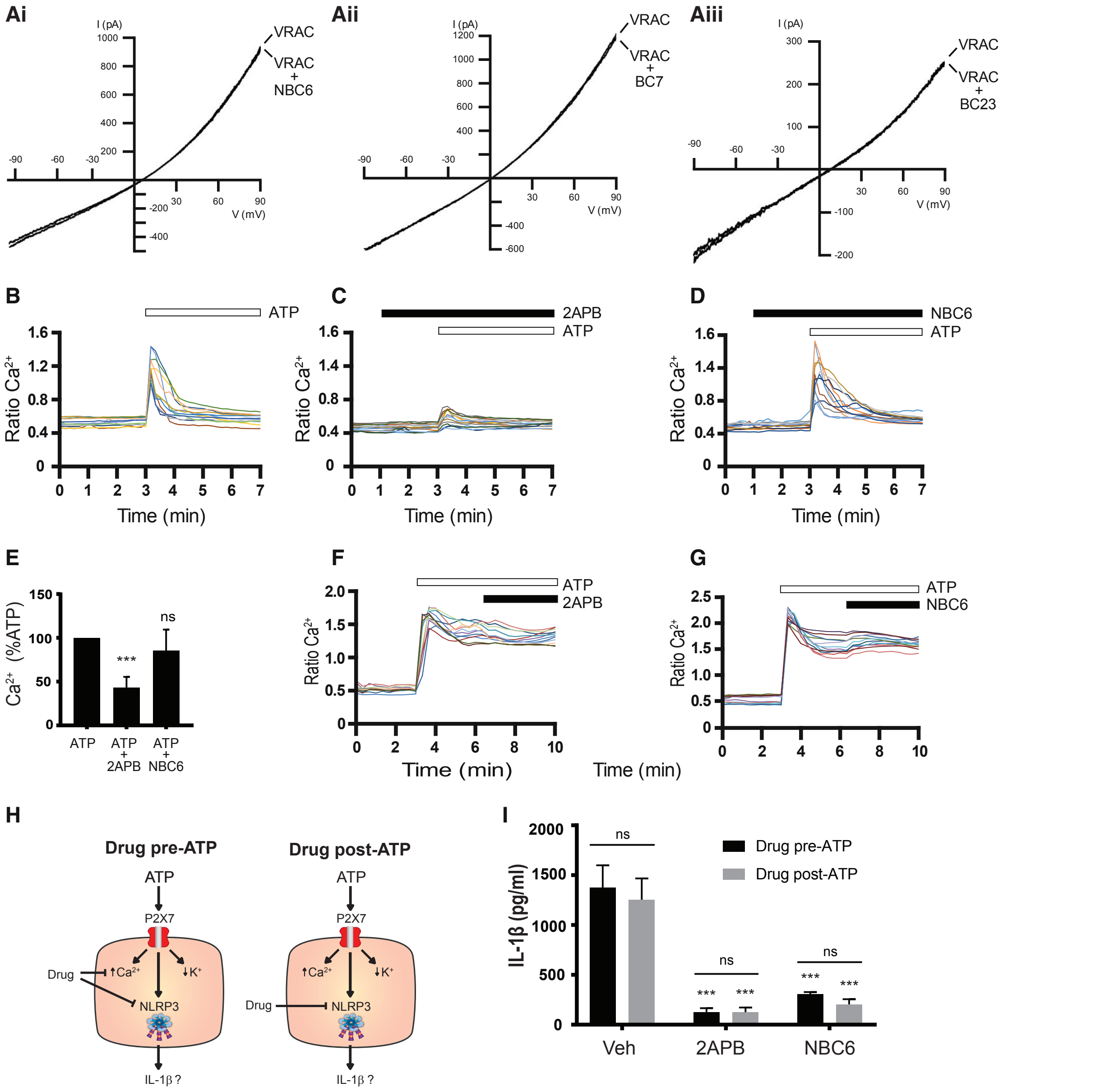

Figure 7. Ca^{2+}-Independent Effects of the NBCs

(A) To induce volume-regulated Cl^- currents (VRAC), LPS-primed (1 μg mL^{-1}, 2 hr) iBMDMs were superfused with hypotonic solution. Representative current traces are shown, which have been measured in the absence (VRAC) or presence of 30 μM NBC6 (Ai), 30 μM BC7 (Aii), or 30 μM BC23 (Aiii).

(B–E) LPS-primed (1 μg mL^{-1}, 2 hr) iBMDMs were kept untreated or were pre-treated for 2 min with 75 μM 2APB or 30 μM NBC6. Subsequently, 100 μM ATP was added to the bath solution. (B–D) Representative Ca^{2+} traces of ATP-stimulated cells in the absence (B, n = 12) or presence of 75 μM 2APB (C, n = 14), or 30 μM NBC6 (D, n = 12). (E) Mean peak Ca^{2+} concentrations determined in cells treated with ATP alone (ATP) or with ATP in the presence of inhibitors. ns, no significant difference; ***$p < 0.001$, significant difference from Ca^{2+} signals of ATP-stimulated cells determined in the absence of inhibitors (Holm-Sidak corrected one-sample t test).

(F and G) LPS-primed iBMDMs were also stimulated with 5 mM ATP. Following development of sustained Ca^{2+} increases, 75 μM 2APB or 30 μM NBC6 was added to the ATP-containing solution. Images show representative examples of Ca^{2+} responses following treatment with ATP and the addition of 2APB (n = 12, F) or the addition of NBC6 (n = 12, G).

(H and I) LPS-primed iBMDMs were treated with concentrations of the inhibitors maximal for blocking IL-1β release (i.e., 2APB = 75 μM, NBC6 = 30 μM). Inhibitors were added to the iBMDMs 5 min before or 5 min after the addition of ATP (5 mM, 1 hr) (H) with IL-1β release measured by ELISA (I). ***$p < 0.001$, significant difference from corresponding vehicle control; ns, no significant effect of ATP administration time.

Data are presented as representative traces from calcium imaging experiments (B–D, F, and G), mean + SEM peak Ca^{2+} concentrations versus treatment with ATP alone (E), or mean + SEM IL-1β release as detected by ELISA (I).

and others (Fowler et al., 2014) have shown that some existing drugs may be repurposed. There is, however, a need for new inhibitors. MCC950 (formerly CRID3 or CP-456,773) is being developed as a potent and selective inhibitor of NLRP3 (Coll et al., 2015). Here we report a new class of molecules based on the oxazaborine ring that will further accelerate the development of NLRP3 inhibitors for use in disease and in generating new fundamental insights.

STAR★METHODS

Detailed methods are provided in the online version of this paper and include the following:

- KEY RESOURCES TABLE
- CONTACT FOR REAGENT AND RESOURCE SHARING
- EXPERIMENTAL MODEL AND SUBJECT DETAILS
  - Cell Culture
  - *In Vivo* Peritoneal Inflammation Model
- METHOD DETAILS
  - ASC Speck Imaging
  - Caspase-1 Assays
  - Inflammasome Activation Assays
  - Washout Experiments
  - Cell Death Experiments
  - Chemistry Synthesis
  - X-ray Crystallography
  - Chemistry Computational/Modelling
  - Fluorescence Imaging
  - Electrophysiological Recordings
- QUANTIFICATION AND STATISTICAL ANALYSIS
- DATA AND SOFTWARE AVAILABILITY

SUPPLEMENTAL INFORMATION

Supplemental Information includes one figure, one table, seven schemes, and supplemental text and can be found with this article online at http://dx.doi.org/10.1016/j.chembiol.2017.08.011.

AUTHOR CONTRIBUTIONS

Conceptualization, D.B. and S.F.; Methodology, D.B., S.F., M.K.H., S.M.A., C.B.L., and R.A.B.; Investigation, A.G.B., J.R.-A., M.J.D.D., C.S.W., C.H.S., T.S., H.H., P.J., N.G.S., H.E., N.M.L., and M.K.; Formal analysis, J.R.-A.; Writing – Original Draft, D.B., S.F., and A.G.B.; Writing – Review & Editing, D.B., S.F., and A.G.B.; Funding Acquisition, D.B. and S.F.; Resources, D.B., S.F., and S.M.A.; Supervision, D.B., N.J.R., R.A.B., C.E., and S.F.

ACKNOWLEDGMENTS

We thank Paul MacLellan and Soraya Alnabulsi for initial support with organic synthesis. Thanks to Neil O'Hara for assistance with NMR kinetic runs and the Mass Spectroscopy Service in the School of Chemistry, University of Manchester. We thank Dr P.N. Horton and Dr G.J. Tizzard of the UK National Crystallography Service, Southampton, for collection of X-ray crystallographic data. We are also grateful to Dr. Arnaud Garcon and UMIP for guidance and financial support provided to this project. Preliminary work in this project was supported by a Wellcome Trust fellowship (D.B. grant ref. no. 083482/Z/07/Z). We are also grateful to the SBC Open Innovation Challenge. This work was also supported by the Medical Research Council Confidence in Concept scheme (grant ref. no. MC_PC_13070). A.G.B. is funded by the Division of Pharmacy and Optometry, University of Manchester and the Presidential Doctoral Scholar award. M.J.D.D. is funded by an MRC DTP studentship (MR/K501311/1). C.B.L., J.R.-A., and D.B. are funded by the Alzheimer's Society (211(AS-PG-2013-2007)). We are grateful to Dr. Vishva Dixit (Genentech) for providing the NLRP3 KO mice and to Prof. Clare Bryant (University of Cambridge) for providing the immortalized BMDMs.

Received: March 10, 2017
Revised: June 15, 2017
Accepted: August 14, 2017
Published: September 21, 2017

REFERENCES

Allen, L.C. (1989). Electronegativity is the average one-electron energy of the valence-shell electrons in ground-state free atoms. J. Am. Chem. Soc. *111*, 9003–9014.

Baldwin, A.G., Brough, D., and Freeman, S. (2016). Inhibiting the inflammasome: a chemical perspective. J. Med. Chem. *59*, 1691–1710.

Bally, I., Arsene, A., Bacescur, M., and Balaban, A.T. (1965). Reaction of beta-ketoenols with diphenylborinic esters. Tetrahedron Lett. *6*, 3929–3931.

Brough, D., Le Feuvre, R.A., Wheeler, R.D., Solovyova, N., Hllfiker, S., Rothwell, N.J., and Verkhratsky, A. (2003). Ca^{2+} stores and Ca^{2+} entry differentially contribute to the release of IL-1 beta and IL-1 alpha from murine macrophages. J. Immunol. *170*, 3029–3036.

Clemens, R.J., and Hyatt, J.A. (1985). Acetoacetylation with 2,2,6-trimethyl-4H-1,3-dioxin-4-one—a convenient alternative to diketene. J. Org. Chem. *50*, 2431–2435.

Coenen, M., Faust, J., Rigel, C., and Mayer, R. (1965). Synthesen mit trichloracetonitril. J. Praktische Chem. *27*, 239.

Coll, R.C., Robertson, A.A., Chae, J.J., Higgins, S.C., Munoz-Planillo, R., Inserra, M.C., Vetter, I., Dungan, L.S., Monks, B.G., Stutz, A., et al. (2015). A small-molecule inhibitor of the NLRP3 inflammasome for the treatment of inflammatory diseases. Nat. Med. *21*, 248–255.

Compan, V., Baroja-Mazo, A., Lopez-Castejon, G., Gomez, A.I., Martinez, C.M., Angosto, D., Montero, M.T., Herranz, A.S., Bazan, E., Reimers, D., et al. (2012). Cell volume regulation modulates NLRP3 inflammasome activation. Immunity *37*, 487–500.

Cools-Lartigue, J., Spicer, J., McDonald, B., Gowing, S., Chow, S., Giannias, B., Bourdeau, F., Kubes, P., and Ferri, L. (2013). Neutrophil extracellular traps sequester circulating tumor cells and promote metastasis. J. Clin. Invest. *123*, 3446–3458.

Cramer, R.D. (2003). Topomer CoMFA: a design methodology for rapid lead optimization. J. Med. Chem. *46*, 374–388.

Daniels, M.J., Rivers-Auty, J., Schilling, T., Spencer, N.G., Watremez, W., Fasolino, V., Booth, S.J., White, C.S., Baldwin, A.G., Freeman, S., et al. (2016). Fenamate NSAIDs inhibit the NLRP3 inflammasome and protect against Alzheimer's disease in rodent models. Nat. Commun. *7*, 12504.

Dobrydneva, Y., and Blackmore, P. (2001). 2-Aminoethoxydiphenyl borate directly inhibits store-operated calcium entry channels in human platelets. Mol. Pharmacol. *60*, 541–552.

Dorokhov, V.A., Vasil'ev, L.S., Surzhikov, F.E., and Bogdanov, V.S. (1995). Chelate synthesis of 3-ethoxycarbonyl-4-hydroxy-2-trifluoromethylpyridine from ethyl acetoacetate and trifluoroacetonitrile. Russ. Chem. Bull. *44*, 1283–1285.

Farrugia, L.J. (2012). WinGX and ORTEP for Windows: an update. J. Appl. Crystallogr. *45*, 849–854.

Fowler, B.J., Gelfand, B.D., Kim, Y., Kerur, N., Tarallo, V., Hirano, Y., Amarnath, S., Fowler, D.H., Radwan, M., Young, M.T., et al. (2014). Nucleoside reverse transcriptase inhibitors possess intrinsic anti-inflammatory activity. Science *346*, 1000–1003.

Frisch, M.J., Trucks, G.W., Schlegel, H.B., Scuseria, G.E., Robb, M.A., Cheeseman, J.R., Scalmani, G., Barone, V., Mennucci, B., Petersson, G.A., et al. (2009). Gaussian 09 (Gaussian, Inc).

Grant, J.A., Gallardo, M.A., and Pickup, B.T. (1996). A fast method of molecular shape comparison: a simple application of a Gaussian description of molecular shape. J. Comput. Chem. *17*, 1653–1666.

Gross, C.J., Mishra, R., Schneider, K.S., Medard, G., Wettmarshausen, J., Dittlein, D.C., Shi, H., Gorka, O., Koenig, P.A., Fromm, S., et al. (2016). K^+ efflux-independent NLRP3 inflammasome activation by small molecules targeting mitochondria. Immunity *45*, 761–773.

Guo, H., Callaway, J.B., and Ting, J.P. (2015). Inflammasomes: mechanism of action, role in disease, and therapeutics. Nat. Med. *21*, 677–687.

Hawkins, P.C.D., Skillman, A.G., and Nicholls, A. (2007). Comparison of shape-matching and docking as virtual screening tools. J. Med. Chem. *50*, 74–82.

Hawkins, P.C.D., Skillman, A.G., Warren, G.L., Ellingson, B.A., and Stahl, M.T. (2010). Conformer generation with OMEGA: algorithm and validation using high quality structures from the Protein Databank and Cambridge Structural Database. J. Chem. Inf. Model. *50*, 572–584.

He, Y., Varadarajan, S., Munoz-Planillo, R., Burberry, A., Nakamura, Y., and Nunez, G. (2014). 3,4-Methylenedioxy-beta-nitrostyrene inhibits NLRP3 inflammasome activation by blocking assembly of the inflammasome. J. Biol. Chem. *289*, 1142–1150.

He, Y., Zeng, M.Y., Yang, D., Motro, B., and Nunez, G. (2016). NEK7 is an essential mediator of NLRP3 activation downstream of potassium efflux. Nature *530*, 354–357.

Heneka, M.T., Golenbock, D.T., and Latz, E. (2015). Innate immunity in Alzheimer's disease. Nat. Immunol. *16*, 229–236.

Hofer, A., Kovacs, G., Zappatini, A., Leuenberger, M., Hediger, M.A., and Lochner, M. (2013). Design, synthesis and pharmacological characterization of analogs of 2-aminoethyl diphenylborinate (2-APB), a known store-operated calcium channel blocker, for inhibition of TRPV6-mediated calcium transport. Bioorg. Med. Chem. *21*, 3202–3213.

Horng, T. (2014). Calcium signaling and mitochondrial destabilization in the triggering of the NLRP3 inflammasome. Trends Immunol. *35*, 253–261.

Hornung, V., Bauernfeind, F., Halle, A., Samstad, E.O., Kono, H., Rock, K.L., Fitzgerald, K.A., and Latz, E. (2008). Silica crystals and aluminum salts activate the NALP3 inflammasome through phagosomal destabilization. Nat. Immunol. *9*, 847–856.

Hosoya, T., Uekusa, H., Ohashi, Y., Ohhara, T., and Kuroki, R. (2006). A new photoisomerization process of the 4-cyanobutyl group in a cobaloxime complex crystal observed by neutron diffraction. Bull. Chem. Soc. Jpn. *79*, 692–701.

Ibrahim, N.S., Abdelrazek, F.M., Aziz, S.I., and Elnagdi, M.H. (1985). Nitriles in organic-synthesis—the reaction of trichloroacetonitrile with active methylene reagents. Monatshefte Chem. *116*, 551–556.

Janesko, B.G. (2010). Using nonempirical semilocal density functionals and empirical dispersion corrections to model dative bonding in substituted boranes. J. Chem. Theor. Comput. *6*, 1825–1833.

Josefíka, F., Svobodováa, M., Bertolasib, V., Šimůneka, P., Macháčeka, V., Almonasya, N., and Černoškovác, E. (2012). A new bicyclic oxazaborines with a bridged nitrogen atom, their thermic rearrangement and fluorescence properties. J. Organomet. Chem. *699*, 75–81.

Juliana, C., Fernandes-Alnemri, T., Kang, S., Farias, A., Qin, F., and Alnemri, E.S. (2012). Non-transcriptional priming and deubiquitination regulate NLRP3 inflammasome activation. J. Biol. Chem. *287*, 36617–36622.

Katsnelson, M.A., Rucker, L.G., Russo, H.M., and Dubyak, G.R. (2015). K^+ efflux agonists induce NLRP3 inflammasome activation independently of Ca^{2+} signaling. J. Immunol. *194*, 3937–3952.

Kayagaki, N., Warming, S., Lamkanfi, M., Vande Walle, L., Louie, S., Dong, J., Newton, K., Qu, Y., Liu, J., Heldens, S., et al. (2011). Non-canonical inflammasome activation targets caspase-11. Nature *479*, 117–121.

Kayagaki, N., Wong, M.T., Stowe, I.B., Ramani, S.R., Gonzalez, L.C., Akashi-Takamura, S., Miyake, K., Zhang, J., Lee, W.P., Muszynski, A., et al. (2013). Noncanonical inflammasome activation by intracellular LPS independent of TLR4. Science *341*, 1246–1249.

Lamkanfi, M., and Dixit, V.M. (2012). Inflammasomes and their roles in health and disease. Annu. Rev. Cell Dev. Biol. *28*, 137–161.

Latz, E., Xiao, T.S., and Stutz, A. (2013). Activation and regulation of the inflammasomes. Nat. Rev. Immunol. *13*, 397–411.

Le Feuvre, R.A., Brough, D., Iwakura, Y., Takeda, K., and Rothwell, N.J. (2002). Priming of macrophages with lipopolysaccharide potentiates P2X7-mediated cell death via a caspase-1-dependent mechanism, independently of cytokine production. J. Biol. Chem. *277*, 3210–3218.

Lee, G.S., Subramanian, N., Kim, A.I., Aksentijevich, I., Goldbach-Mansky, R., Sacks, D.B., Germain, R.N., Kastner, D.L., and Chae, J.J. (2012). The calcium-sensing receptor regulates the NLRP3 inflammasome through Ca^{2+} and cAMP. Nature *492*, 123–127.

Lopez-Castejon, G., Luheshi, N.M., Compan, V., High, S., Whitehead, R.C., Flitsch, S., Kirov, A., Prudovsky, I., Swanton, E., and Brough, D. (2013). Deubiquitinases regulate the activity of caspase-1 and interleukin-1beta secretion via assembly of the inflammasome. J. Biol. Chem. *288*, 2721–2733.

McGettrick, A.F., and O'Neill, L.A. (2013). NLRP3 and IL-1beta in macrophages as critical regulators of metabolic diseases. Diabetes Obes. Metab. *15*, 19–25.

Mikyseka, T., Kvapilováb, H., Doušovác, H., Josefíkc, F., Šimůnekc, P., Růžičkovád, Z., and Ludvík, J. (2017). Synthesis, electrochemical, structural and theoretical study of new derivatives of O-B-N and O-B-O heterocycles. Inorg. Chim. Acta *455*, 465–472.

Munoz-Planillo, R., Kuffa, P., Martinez-Colon, G., Smith, B.L., Rajendiran, T.M., and Nunez, G. (2013). K(+) efflux is the common trigger of NLRP3 inflammasome activation by bacterial toxins and particulate matter. Immunity *38*, 1142–1153.

Murakami, T., Ockinger, J., Yu, J., Byles, V., McColl, A., Hofer, A.M., and Horng, T. (2012). Critical role for calcium mobilization in activation of the NLRP3 inflammasome. Proc. Natl. Acad. Sci. USA *109*, 11282–11287.

Peppiatt, C.M., Collins, T.J., MacKenzie, L., Conway, S.J., Holmes, A.B., Bootman, M.D., Berridge, M.J., Seo, J.T., and Roderick, H.L. (2003). 2-Aminoethoxydiphenyl borate (2-APB) antagonises inositol 1,4,5-trisphosphate-induced calcium release, inhibits calcium pumps and has a use-dependent and slowly reversible action on store-operated calcium entry channels. Cell Calcium *34*, 97–108.

Py, B.F., Kim, M.S., Vakifahmetoglu-Norberg, H., and Yuan, J. (2013). Deubiquitination of NLRP3 by BRCC3 critically regulates inflammasome activity. Mol. Cell *49*, 331–338.

Rossol, M., Pierer, M., Raulien, N., Quandt, D., Meusch, U., Rothe, K., Schubert, K., Schoneberg, T., Schaefer, M., Krugel, U., et al. (2012). Extracellular Ca^{2+} is a danger signal activating the NLRP3 inflammasome through G protein-coupled calcium sensing receptors. Nat. Commun. *3*, 1329.

Sander, T., Freyss, J., Von Korff, M., and Rufener, C. (2015). DataWarrior: an open-source program for chemistry aware data visualization and analysis. J. Chem. Inf. Model. *55*, 460–473.

Schaefer, M. (2014). TRPs: modulation by drug-like compounds. Handb. Exp. Pharmacol. *223*, 1077–1106.

Schmid-Burgk, J.L., Chauhan, D., Schmidt, T., Ebert, T.S., Reinhardt, J., Endl, E., and Hornung, V. (2016). A genome-wide CRISPR (Clustered Regularly Interspaced Short Palindromic Repeats) screen identifies NEK7 as an essential component of NLRP3 inflammasome activation. J. Biol. Chem. *291*, 103–109.

Shi, H., Wang, Y., Li, X., Zhan, X., Tang, M., Fina, M., Su, L., Pratt, D., Bu, C.H., Hildebrand, S., et al. (2016). NLRP3 activation and mitosis are mutually exclusive events coordinated by NEK7, a new inflammasome component. Nat. Immunol. *17*, 250–258.

Singh, B., and Lesher, G.Y. (1978). Convenient preparations of ethyl 3-oxopentanoate and 3,5-disubstituted 1,2-oxazole-4-carboxylic acids from a common type of intermediate. Synthesis-Stuttgart *1978*, 829–830.

Sridharan, V., Ruiz, M., and Menendez, J.C. (2010). Mild and high-yielding synthesis of beta-keto esters and beta-ketoamides. Synthesis-Stuttgart *2010*, 1053–1057.

Vasil'ev, L.S., Azarevich, O.G., Bogdanov, V.S., Bochkareva, M.N., and Dorokhov, V.A. (1992). Boron chelates with 5,5,5-trifluoro- and 5,5,5-trichloro-4-aminopent-3-en-2-ones. Bull. Russ. Acad. Sci. Division Chem. Sci. *41*, 2104–2107.

Vasil'ev, L.S., Surzhikov, F.E., Baranin, S.V., and Dorokhov, V.A. (2013). Trifluoromethyl-substituted 1,6-naphthyridines and pyrido[4,3-d]pyrimidines. Russ. Chem. Bull. *62*, 1255–1261.

Vasil'ev, L.S., Azarevich, O.G., Bogdanov, V.S., Ugrak, B.I., and Dorokhov, V.A. (1994). Chelate synthesis of functionally-substituted 2-trichloromethylpyridines. Russ. Chem. Bull. *43*, 1282–1283.

Veronese, A.C., Talmelli, C., Gandolfi, V., Corain, B., and Basato, M. (1986). Metal-catalyzed reactions of beta-dicarbonyls with trichloroacetonitrile - synthesis of beta-trichloromethylenaminodiones. J. Mol. Catal. *34*, 195–198.

Zhao, Y., and Truhlar, D.G. (2006). A new local density functional for main-group thermochemistry, transition metal bonding, thermochemical kinetics, and noncovalent interactions. J. Chem. Phys. *125*, 194101.

STAR★METHODS

KEY RESOURCES TABLE

REAGENT or RESOURCE	SOURCE	IDENTIFIER
Antibodies		
Mouse IL-1â antibody	R&D Systems	Cat # AF-401-NA; RRID: AB_416684
Human IL-1â antibody	R&D Systems	Cat # AF-201-NA; RRID: AB_354387
Chemicals, Peptides, and Recombinant Proteins		
Dulbecco's Modified Eagle's Medium (DMEM)	Sigma	D6429
RPMI-1640	Sigma	R0883
Fetal bovine serum (FBS)	Thermo Fisher	10500064
2-APB	Sigma	D9754
diphenylborinic anhydride	Sigma	358835
diphenylhydramine	Sigma	D3630
2,2-diphenyltetrahydrofuran	Sigma	S408271
phenytoin	Sigma	PHR1139
LPS (E.coli O26:B6)	Sigma	L2654
LPS (E. coli 127:B8)	Sigma	L4516
ATP	Sigma	A2383
Nigericin	Sigma	N7143
Sphingosine	Sigma	S7049
MCC950	Sigma	PZ0280
Corn Oil	Sigma	C8267
3,4-methylenedioxy-â-nitrostyrene	Sigma	M7445
The BC compound library	Sigma	
Imiquimod	Sigma	1338313
Non-B analogs of 2-APB	NIH's National Cancer Institute (NCI) Developmental Therapeutics Program	https://dtp.cancer.gov/organization/dscb/obtaining/default.htm
NBC compounds	This paper	
MSU crystals	Invivogen	Tlrl-msu
CPPD crystals	Invivogen	Tlrl-cppd
Alum crystals	Invivogen	Tlrl-alk
Flagellin from S. typhimurium	Invivogen	tlrl-stfla
Z-YVAD-AFC	Calbiochem	688225
Ac-YVAD-Cho	Merck-Millipore	400010
Recombinant caspase-1	Merck-Millipore	CC126
Silica	U.S Silica	MIN-U-SIL 15
Poly(deoxyadenylic-thymidylic) acid sodium salt (Poly dA:dT)	Sigma	P0883
Pam3CSK4	Invivogen	tlrl-pms
Lipofectamine 3000	Thermo Fisher	L3000008
Critical Commercial Assays		
IL-1â ELISA (mouse)	R&D Systems	DY401
IL-1á ELISA (mouse)	R&D Systems	DY400
IL-1â ELISA (human)	R&D Systems	DY201
CytoTox 96® Non-Radioactive Cytotoxicity Assay	Promega	G1780

(*Continued on next page*)

Continued		
REAGENT or RESOURCE	SOURCE	IDENTIFIER
Deposited Data		
Crystal structure NBC6	This paper	Cambridge Crystallographic Data Centre CCDC 1563191
Crystal structure NBC11	This paper	Cambridge Crystallographic Data Centre CCDC 1563192
Experimental Models: Cell Lines		
THP-1	ATCC	TIB-202
Primary mouse peritoneal macrophages	Brough lab, UoM	
Primary mouse BMDMs	Brough lab UoM	
ASC-mCherry BMDMs	Brough lab UoM	
HepG2	ATCC	HB-8065
HEK293	ATCC	CRL-1573
Experimental Models: Organisms/Strains		
C57BL/6 mice	Envigo	
NLRP3 knockout mice	Genentech	
Software and Algorithms		
SYBYL-X 2.1	Tripos Inc	https://www.certara.com/software/molecular-modeling-and-simulation/sybyl-x-suite/
GraphPad Prism version 7.00 for Windows	GraphPad Software	www.graphpad.com
R 3.30	R Foundation for Statistical Computing	http://www.R-project.org/
Gaussian 09	Gaussian, Inc	http://gaussian.com/
Omega 2.5.1.4	OpenEye Scientific Software	https://www.eyesopen.com/
ROCS 3.0.0	OpenEye Scientific Software	https://www.eyesopen.com/
OSIRIS DataWarrior 4.5.2	Actelion Pharmaceuticals Ltd	http://www.openmolecules.org/datawarrior/

CONTACT FOR REAGENT AND RESOURCE SHARING

Further information and requests for resources and reagents should be directed to the Lead Contact, David Brough (David.brough@manchester.ac.uk).

EXPERIMENTAL MODEL AND SUBJECT DETAILS

Cell Culture

Primary peritoneal macrophages were prepared as described previously (Le Feuvre et al., 2002). Briefly, peritoneums of male and female C57BL/6 mice (Charles River) were lavaged with 8 ml RPMI 1640 media and cells in the exudate cultured at a density of 1×10^6 cells ml^{-1} in RPMI media supplemented with 10% fetal bovine serum (FBS), 100 U ml^{-1} penicillin and 100 $\mu g\ ml^{-1}$ streptomycin (PenStrep). Primary bone marrow-derived macrophages (BMDMs) and primary bone marrow neutrophils were prepared by flushing femurs of male and female wild-type C57BL/6 or NLRP3 KO mice. Red cells were then lysed. BMDMs were generated by culturing the resulting bone marrow cells in 70% DMEM (containing 10% FBS, PenStrep) supplemented with 30% L929 mouse fibroblast-conditioned media for 7-10 days. Before experiments, cells were seeded overnight at $1 \times 10^6\ ml^{-1}$ in 96-well plates. Neutrophils were isolated by density centrifugation of the extracted bone marrow cells in a 64% isotonic Percoll (Sigma-Aldrich) at 1,000xg for 30 min at 4°C. The pellet was then resuspended in RPMI (containing 10% FBS, PenStrep), counted, centrifuged again (2,000xg, 5 min), resuspended at $1 \times 10^6\ ml^{-1}$, plated in 96-well plates and experimented on immediately. Purity (>90%) and viability (>95%) were determined by Diff-Quik® staining (Cools-Lartigue et al., 2013). THP-1 peripheral blood monocyte-like cells were cultured in RPMI medium supplemented with 10% FBS, PenStrep, 20 mM L-Glutamine and 55 μM 2-mercaptoethanol. On the day of experiments, cells were seeded overnight at $1 \times 10^6\ ml^{-1}$ in 96-well plates. Immortalized murine bone marrow-derived macrophages (iBMDMs) (Hornung et al., 2008) and iBMDMs stably expressing ASC conjugated to mCherry protein (Daniels et al., 2016) were cultured in DMEM, 10% FBS, PenStrep. HEK293T kidney cells and HepG2 liver cells were cultured in DMEM, 10% FBS, PenStrep.

In Vivo Peritoneal Inflammation Model

Animals were maintained under standard laboratory conditions: ambient temperatures of 21°C (± 2°C), humidity of 40–50%, 12 h light cycle, *ad libitum* access to water and standard rodent chow. All procedures were performed blinded to genotype. Treatment

allocations were randomly allocated using True Random Generator™ software. All animal experiments were carried out in accordance with the United Kingdom Animals (Scientific Procedures) Act 1986 and approved by the Home Office and the local Animal Ethical Review Group, University of Manchester. Male WT C57BL/6 and strain matched NLRP3 KO mice (30 - 35g) were co-administered intraperitoneally (i.p.) with NBC13 (50 mg kg^{-1}), MCC950 (50 mg kg^{-1}) or vehicle (corn oil)), and 10 mg kg^{-1} LPS (from *Escherichia coli* 0127:B8) or saline control (n=6 per group). Three hours following injection the mice were anesthetized with 3-5% isoflurane, their peritoneums were lavaged with 3 ml of RPMI media and plasma taken by cardiac puncture. Levels of IL-1β in the plasma and lavage and IL-1α in the plasma were analysed by ELISA (DuoSet, R&D systems®).

METHOD DETAILS

ASC Speck Imaging

Live imaging of ASC speck formation was performed using iBMDMs transfected to stably express ASC conjugated to mCherry protein (Daniels et al., 2016). Stably transduced cells were plated overnight at $5x10^5$ cells ml^{-1}. The following day, cells were primed with LPS (1 μg ml^{-1}, 2 h). 1 h into priming, Hoechst 33342 (2 μg ml^{-1}, Immunochemistry) was added to aid identification of the cells. Following priming, media was changed to DMEM containing 25 mM HEPES pH 7.4 and cells transferred to a BD Pathway Bioimager 855 (BD Biosciences) and imaged at 37°C as described previously (Daniels et al., 2016). Cells were pre-treated with 2APB, BC7, BC23, NBC6, or vehicle for 15 min before imaging.

Caspase-1 Assays

The caspase-1 activity of THP-1 cells was determined with the fluorogenic substrate Z-YVAD-AFC (caspase-1 substrate VI, Calbiochem) as previously described (Lopez-Castejon et al., 2013). Briefly, cells were lysed in hypotonic cell lysis buffer (25 mM HEPES, 5 mM EGTA, 5 mM dithiothreitol (DTT), pH 7.5) on ice for 5–10 min and centrifuged to remove the insoluble fraction (12,500×g, 10 min). THP-1 lysates (50 μl) or recombinant caspase-1 (10 U ml^{-1}) was incubated with 50 μM YVAD-AFC and 50 μl of reaction buffer (0.2% CHAPS, 0.2 M HEPES, 20% sucrose, 29 mM DTT, pH 7.5) for 2 h. After incubation, the fluorescence of the AFC released from the Z-YVAD-AFC substrate was measured by an increase in fluorescence (excitation 335 nm, emission 460 nm).

Inflammasome Activation Assays

Peritoneal macrophages were primed with LPS (1 μg ml^{-1}, 2 h) before incubation with inhibitors in serum free media (15 min) followed by stimulation with NLRP3 activators ATP (5 mM, 20 min), mono-sodium urate crystals (MSU, 250 μg ml^{-1}, 1 h), calcium pyrophosphate dihydrate crystals (CPPD, 250 μg ml^{-1}, 1 h), Aluminium hydroxide crystals (Alum, 250 μg ml^{-1}, 1 h), nigericin (20 μM, 15 min), or sphingosine (20 μM, 1 h). THP-1 cells were primed with LPS (1 μg ml^{-1}, 4 h) before incubation with inhibitors or vehicle (15 min) in serum-free media followed by stimulation with nigericin (10 μM, 1 h), or ATP (5 mM, 1 h). For AIM2/NLRC4 inflammasome activation primary BMDMs were primed with LPS (1 μg ml^{-1}, 4 h). Subsequent to LPS priming, cells were pre-treated with drugs or vehicle (DMSO) in serum-free media for 15 min then stimulated with ATP (5 mM, 1 h), poly(deoxyadenylic-thymidylic) (polydA:dT) acid sodium salt transfected with Lipofectamine® 3000 (667 ng ml^{-1}, 4 h) or flagellin from *S. typhimurium* (667 ng ml^{-1}, 4 h). For K^+ efflux-independent NLRP3 activation BMDMs were primed with LPS as above. Subsequent to LPS priming, cells were pre-treated with drugs or vehicle (DMSO, 15 min) in PBS then stimulated with imiquimod (10 μM, 4 h). For non-canonical inflammasome activation cells were primed with Pam3CSK4 (100 ng ml^{-1}, 4 h). Subsequent to priming, cells were pre-treated with drugs or vehicle (DMSO, 15 min) in serum-free media then stimulated with LPS transfected with Lipofectamine® 3000 (2 μg ml^{-1}, 24 h). Neutrophils were primed with LPS (1 μg ml^{-1}, 2 h) in RPMI (containing 10% FBS, PenStrep), drug or vehicle (DMSO) was added 15 min prior to the stimulation with nigericin or vehicle (DMSO) for 1 h. Supernatants were removed and analysed for IL-1β or IL-1α content by ELISA (DuoSet, R&D systems) according to manufacturer's instructions.

Washout Experiments

iBMDMs were seeded overnight at 7.5 x 10^5 ml^{-1} in 24-well plates and primed with LPS (1 μg ml^{-1}, 2 h). Subsequent to LPS priming, cells were pre-treated with drugs or vehicle (DMSO, 15 min) in serum-free media and washed 3 times, before inflammasome activation was initiated by adding ATP (5 mM, 1 h).

Cell Death Experiments

HEK293T kidney cells and HepG2 liver cells were treated with drug or vehicle (DMSO) for 4, 8 and 24 h in DMEM, 1% FBS, PenStrep.

Following treatment, cell death was measured by assessing lactate dehydrogenase release using the CytoTox 96 Non-Radioactive Cytotoxicity Assay (Promega) according to manufacturer's instructions.

Chemistry Synthesis

Synthesis, purification and characterisation of NBC molecules are outlined in Methods S1 (Ibrahim et al., 1985; Coenen et al., 1965; Hosoya et al., 2006; Vasil'ev et al., 1994; Sridharan et al., 2010; Clemens and Hyatt, 1985). All chemicals, solvents and deuterated solvents were purchased from Sigma-Aldrich, Alfa-Aesar or Fisher Scientific. ^1H, ^{13}C and $^{11}B\{^1H\}$ NMR spectra were recorded on a Bruker Avance 400 or 300 MHz spectrometer. Chemical shifts (δ) are defined in parts per million (ppm). ^1H NMR spectra were referenced to tetramethylsilane (TMS, δ=0.0 ppm) or residual undeuterated solvent ($CDCl_3$, δ=7.26 ppm; DMSO-d_6, δ=2.50 ppm).

^{13}C NMR spectra were referenced to residual undeuterated solvent as an internal reference. $^{11}B\{^{1}H\}$NMR chemical shifts were referenced to external reference $BF_3.OEt_2$ (δ=0.0 ppm). ESI and APCI mass spectrometry was carried out on a Waters Acquity UPLC system connected to a Waters SQD2 mass spectrometer. Accurate mass determination was carried out on a Thermo Exactive™ Plus EMR Orbitrap™ LC-MS system. Molecular ion peaks are defined as mass/charge (*m/z*) ratios. Infrared spectroscopy was recorded on a JASCO FT/IR-4100 spectrophotometer using the Spectra Manager II (JASCO) software package. Microwave irradiation was carried out on a Biotage® Initiator Classic microwave using 2-5 ml Biotage® glass vials. Analytical thin-layer chromatography (TLC) was performed using silica gel 60 on aluminium sheets coated with F_{254} indicator. All spots were visualised with $KMnO_4$ or ultraviolet light using a MV Mineralight lamp (254/365) UVGL-58. Flash column chromatography was performed using silica gel with particle size 40-63 μm. Evaporation of solvents was conducted on a Buchi Rotavapor R-200.

X-ray Crystallography

X-ray diffraction data were collected at 100 K on the specimen crystals of NBC6 and NBC11 at the National Crystallography Service, Southampton, UK, with MoKα radiation produced by a rotating anode generator. The structures were solved by direct methods with SHELXS and refined with SHELXL, implemented in the WinGX package (Farrugia, 2012), by the full-matrix least-squares technique with anisotropic displacement parameters for the non-hydrogen atoms. Hydrogen atoms attached to carbon were placed in calculated positions and assumed to ride on their attached atom, methyl groups being allowed to rotate. The C12 methyl group of NBC11 showed signs of disorder in a difference electron density map and therefore was assigned two sets of sites rotated by 60° from one another with occupancy factors that refined to 0.59(2) : 0.41(2). Positions and isotropic displacement parameters for hydrogen atoms attached to nitrogen atoms were refined freely. Final discrepancy indices R(obs) and wR2 (all data) were 0.0408, 0.1120 for NBC6 and 0.0283, 0.0801 for NBC11. The highest peaks and deepest holes in a difference electron density map were 0.54, -0.36 and 0.39, -0.25 e $Å^{-3}$ respectively. Cambridge Crystallographic Data Centre CCDC 1563191 (NBC6) and CCDC 1563192 (NBC11) contain the supplementary crystallographic data.

Chemistry Computational/Modelling

Initial 3D molecular structures of boron-containing compounds were constructed and then energy minimised using the Tripos force field in SYBYL-X. These geometries were subsequently optimised quantum mechanically with the semi-local M06-L density functional (Zhao and Truhlar, 2006) and the 6-31G* basis set, using the Gaussian 09 electronic structure package (Frisch et al., 2009). These geometries were used as input for structure-activity analysis and for virtual screening. For the latter, the ZINC subset *leadsNow* was employed, containing 1,943,551 molecules (4/20/12 update). Prior to shape-based screening, multiple conformations of each compound were generated *via* Omega (Hawkins et al., 2010). Shape-based screening was performed using ROCS (Grant et al., 1996) with Tanimoto scoring *via* the ShapeTanimoto and ColorTanimoto functions as implemented in OpenEye (Hawkins et al., 2007). Topomer CoMFA (Cramer, 2003) was performed using the Sybyl software package, based on NBC1-6, 8, 11-20, 22-26 and 28-29, with R-groups defined at positions 4, 5 and 6 of the oxazaborine ring.

cLogP and cLogS calculations were performed for BC7, BC23, NBC1-31 and NBC-EPPS using OSIRIS DataWarrior (version 4.5.2)(Sander et al., 2015).

Fluorescence Imaging

One day before experiments, 10^5 iBMDMs were seeded on glass coverslips in 24-well plates. For Ca^{2+} imaging experiments cells were transferred to the following solution containing (in mM): NaCl, 130; KCl, 5; HEPES, 10; D-glucose, 10; $CaCl_2$, 2; $MgCl_2$, 1 (pH 7.4) and were loaded with 3 μM fura-2-acetoxymethylester (Fura-2-AM, Molecular Probes, Eugene, USA) for 30 min at RT (20-23°C). After washing, coverslips were mounted in a chamber on an inverted Olympus IX50 microscope equipped with a water immersion objective 40x UApo/340 (Olympus Optical Co. GmbH, Hamburg, Germany). The fluorescence imaging system consisted of a Polychrome V monochromator, a Hamamatsu Orca 03G camera and the Windows 7 based Live Acquisition software (Till Photonics, München, Germany). Cells were exposed to light of 340±5 and 380±5 nm wavelength every 10 or 20 s in experiments using 100 μM or 5 mM ATP, respectively. Emission light was passed through a 400 nm dichroic mirror and a 420 nm long pass emission filter (both Olympus, Germany) prior to acquisition. Cells were primed with LPS (1 μg ml^{-1}, 4 h) and incubated with drug (2APB (75 μM), BC7, BC23, NBC6 (all 30 μM)) or vehicle, pre- (2 min) or post- (3 min) ATP stimulation. Data are presented as the ratio of the two background corrected fluorescence intensities. To enable fast drug application, cells were superfused using a four-barrel microperfusion pipette positioned in close proximity to the viewing field.

Electrophysiological Recordings

One day before experiments, 10^5 iBMDMs were seeded on glass coverslips in 24-well plates. Membrane currents were measured using the whole-cell configuration of the patch-clamp technique. An EPC 10 patch-clamp amplifier (HEKA, Lambrecht/Pfalz, Germany) was interfaced to a computer for pulse application and data recording using the program PatchMaster (HEKA). Patch electrodes of 3-5 MΩ were fabricated on a two-stage puller (Narishige PC 10, Tokyo, Japan) from borosilicate glass (Hilgenberg, Malsfeld, Germany). For volume-regulated Cl^- current (VRAC) recordings, patch electrodes were filled with the following intracellular solution I1 (in mM): N-Methyl-D-Glucamine-Chloride (NMG-Cl), 120; HEPES, 10; EGTA, 11; $CaCl_2$, 1; $MgCl_2$, 2; Na_2ATP, 3 (pH 7.3). Cells were kept in extracellular solution E1 containing (in mM): NMG-Cl, 50; HEPES, 10; D-glucose, 10; $CaCl_2$, 2; $MgCl_2$, 1; D-mannitol, 170 (300 mosmol kg^{-1}, pH 7.3). To activate VRAC currents, cells were superfused with hypo-osmolar extracellular solution E2 containing

(in mM): NMG-Cl, 50; HEPES, 10; D-glucose, 10; $CaCl_2$, 2; $MgCl_2$, 1 (130 mosmol kg^{-1}, pH 7.3). All recordings were done at RT (20-23°C). For solution exchange, a four-barrel microperfusion pipette was used. Cells were primed with LPS (1 μg ml^{-1}, 4 h) and incubated with drug (2APB (75 μM), BC7, BC23, NBC6 (all 30 μM)) or vehicle, 15 min before stimulation. Whole-cell currents were filtered at 3 kHz and stored for subsequent analyses, which were performed using the program s (HEKA, Lambrecht/Pfalz, Germany).

QUANTIFICATION AND STATISTICAL ANALYSIS

Data are presented as mean values + standard error of the mean (s.e.m). Levels of significance were $p<0.05$ (*), $p<0.01$ (**), $p<0.001$ (***). Statistical analyses were carried out using GraphPad Prism (version 7) or R (version 3.3.0). Percentage control data were analysed with Holm-Sidak corrected one-sample t-tests against the value of 100%. Data with multiple groups were analysed with a one-way ANOVA. Experiments with two independent variables were analysed using two-ANOVA. These analyses were followed by Holm-Sidak corrected post-hoc comparisons. Homoscedasticity and normality of the residuals were evaluated with the Levene's test and Shapiro Wilks, respectively, and appropriate transformations or corrections were applied where necessary. Dose response curves where fitted using non-linear least squares regression with a 3 parameter logistical sigmoidal model.

DATA AND SOFTWARE AVAILABILITY

The crystallographic data for NBC6 and NBC11 is deposited with the Cambridge Crystallographic Data Centre (CCDC) with the deposition numbers CCDC 1563191 (NBC6) and CCDC 1563192 (NBC11).

Cell Chemical Biology, Volume *24*

Supplemental Information

Boron-Based Inhibitors of the NLRP3 Inflammasome

Alex G. Baldwin, Jack Rivers-Auty, Michael J.D. Daniels, Claire S. White, Carl H. Schwalbe, Tom Schilling, Halah Hammadi, Panichakorn Jaiyong, Nicholas G. Spencer, Hazel England, Nadia M. Luheshi, Manikandan Kadirvel, Catherine B. Lawrence, Nancy J. Rothwell, Michael K. Harte, Richard A. Bryce, Stuart M. Allan, Claudia Eder, Sally Freeman, and David Brough

Supplementary items

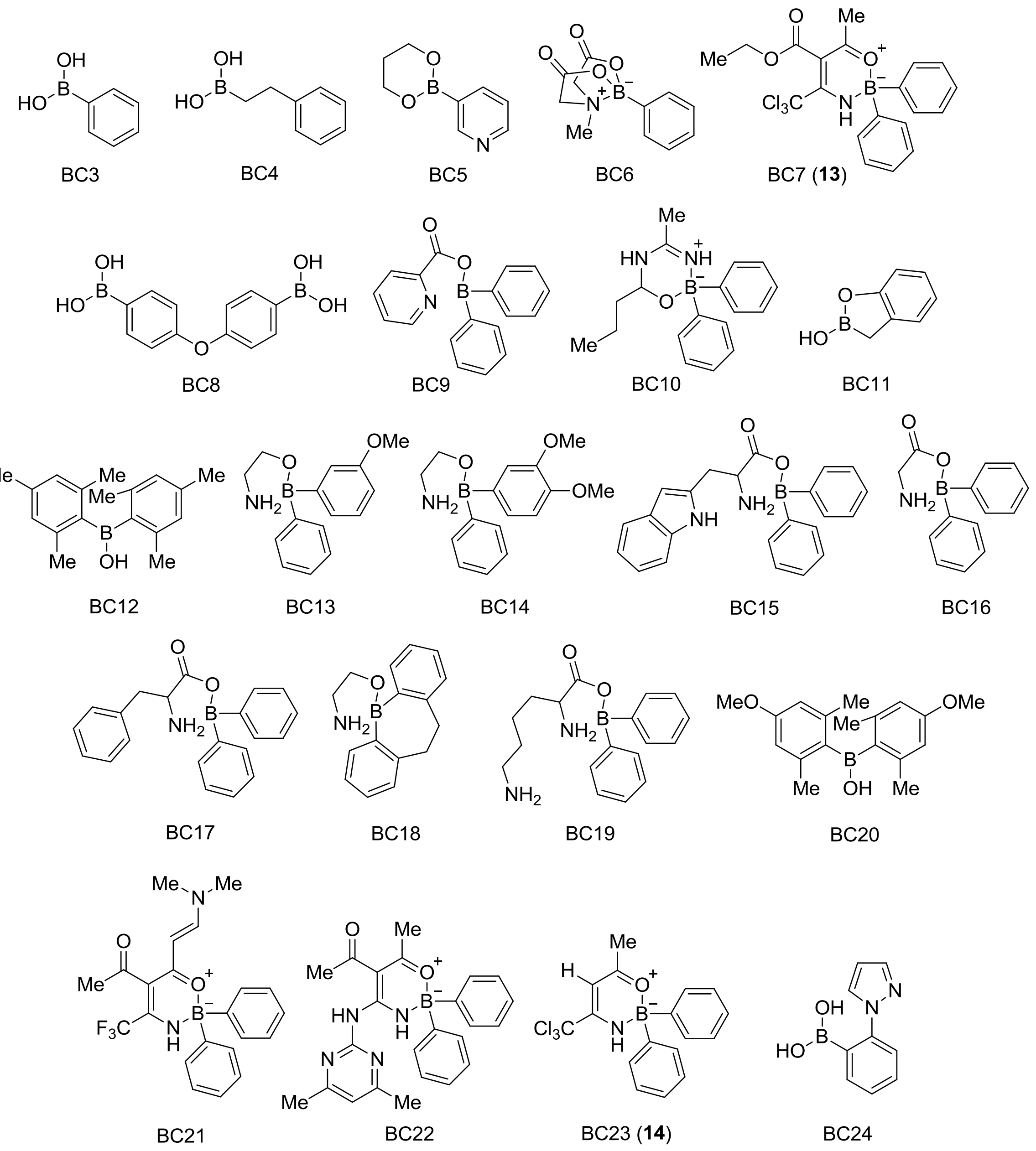

Figure S1 related to Figure 2: Structures of BC molecules. Structures of screened B-containing molecules with similar features and properties to 2-APB.

Parameter	NBC6 (**15**) (X-ray)	NBC6 (**15**) (calc)	NBC11 (**16**) (X-ray)	NBC11 (**16**) (calc)	NBC19 (**17**) (calc)	NBC27 (**18**) (calc)	NBC30 (**19**) (calc)
$q(X_1)^a$	-	-0.571	-	-0.542	-0.567	-0.506	-0.513
$q(B_2)$	-	0.605	-	0.587	0.574	0.515	0.508
$q(N_3)$	-	-0.711	-	-0.719	-0.723	-0.735	-0.747
$q(C_4)$	-	0.391	-	0.448	0.410	0.465	0.458
$q(C_5)$	-	-0.173	-	-0.132	-0.184	-0.346	-0.340
$q(C_6)$	-	0.663	-	0.480	0.664	0.440	0.435
$d(B_2\text{-}X_1)^a$	1.54	1.56	1.53	1.54	1.54	1.60	1.62
$d(B_2\text{-}N_1)$	1.55	1.57	1.57	1.58	1.57	1.58	1.58
$d(X_1\text{-}C_6)^a$	1.30	1.29	1.30	1.30	1.29	1.32	1.32
$d(C_6\text{-}C_5)$	1.44	1.45	1.39	1.40	1.46	1.41	1.41
$d(C_5\text{-}C_4)$	1.41	1.41	1.42	1.42	1.41	1.38	1.38
$d(C_4\text{-}N_3)$	1.31	1.32	1.30	1.31	1.32	1.32	1.32
$\theta(X_1\text{-}B_2\text{-}N_3)^a$	102.6	101.3	104.0	102.5	102.3	112.5	111.2
ν_1	-7.0	-9.2	-8.3	-14.7	-13.2	-0.9	-3.5
ν_2	-23.5	-25.4	-17.3	-17.8	-25.4	-1.7	-9.8
ν_3	16.2	20.3	12.9	14.3	24.6	-0.5	5.3
ν_4	21.4	19.7	17.6	22.2	16.2	5.3	11.9
ν_5	-45.4	-47.2	-37.3	-47.4	-48.8	-6.6	-20.8
ν_6	38.7	42.9	33.2	44.6	48.2	4.5	16.8
$\langle\nu\rangle$	25.4	27.5	21.1	26.8	29.4	3.3	11.4

[a]For NBC6, NBC11 and NBC19, $X_1 = O_1$; for NBC27 and NBC30, $X_1 = N_1$

Table S1 related to Figure 4: Electronic and structural properties of X-ray and calculated geometries of selected oxazaborines and diazaborines at the M06-L/6-31G* level of theory. Mulliken charges q (in e), bond distances d (in Å), ring pucker torsion angles $\nu_1 - \nu_6$ (defined by atoms B_2-N_3-C_4-C_5, N_3-C_4-C_5-C_6, C_4-C_5-C_6-X_1,C_5-C_6-X_1-B_2,C_6-X_1-B_2-N_3, X_1-B_2-N_3-C_4 respectively)[a] and their mean unsigned value $\langle\nu\rangle$ (in deg), bond angle θ (in deg) and donor-acceptor tetrahedral character at boron, THC_{DA} (%). Atom numbering follows Fig. 4A.

Methods S1, related to STAR Methods

1. Design, synthesis and characterisation of the NBC compounds

1.1 General procedure for enaminone reaction using $Zn(acac)_2$

An appropriate nitrile (1 eq) was added to a suspension containing 1,3-dicarbonyl (1 eq), $Zn(acac)_2$ (1-2 %mol) and Na_2CO_3 (1.1 eq) in anhydrous DCM (5 ml). The mixture was stirred at room temperature under N_2 for 3h, unless otherwise stated. DCM/EtOAc (15 ml) was added and the mixture washed with H_2O, dried over $MgSO_4$ and concentrated *in vacuo*.

For deacetylated compounds, $K_2CO_{3(sat)}$ (10 ml) and EtOH (5 ml) were then added and the mixture was stirred at room temperature for 24h. The mixture was extracted with EtOAc, dried over $MgSO_4$ and concentrated *in vacuo*. Alternatively, acetylated enaminones were passed through SiO_2 by flash column chromatography to give the corresponding deacetylated products.

1.2 General procedure for enaminone reaction using $SnCl_4$

An appropriate nitrile (1 eq) was added to a suspension containing 1,3-dicarbonyl (1 eq) and Na_2CO_3 (1.1 eq) in anhydrous toluene (10 ml). For deacetylated products, addition of Na_2CO_3 was omitted. $SnCl_4$ (1 eq) was slowly added and the mixture was stirred at room temperature under N_2 for 30 min. The mixture was then heated to 80°C and stirred for 4h, unless otherwise stated. MeCN (20 ml) was added to dissolve the gummy solid, filtered to remove Na_2CO_3, concentrated *in vacuo* and

dried under vacuum overnight. After 16h, boiling $CHCl_3$ (20 ml) was added and refluxed for 1h. The solid was filtered and dried under vacuum.

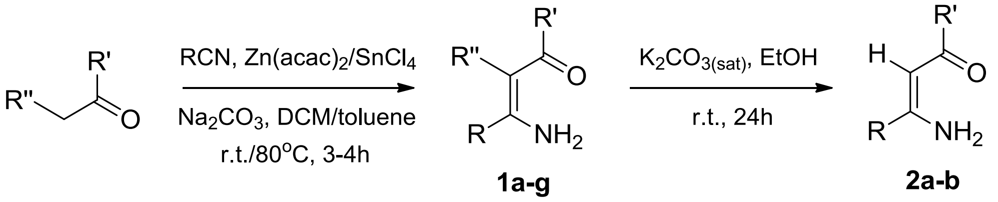

R	R'	R''	
CCl_3	Me	COOEt	**1a**
Me	Me	COOEt	**1b**
Me	Me	COMe	**1c**
CCl_3	NH_2	COMe	**1d**
CCl_3	Me	$CONMe_2$	**1e**
CCl_3	NH_2	CN	**1f**
CCl_3	O^tBu	COMe	**1g**

R	R'	
CCl_3	Me	**2a**
CCl_3	NH_2	**2b**

Scheme 1 | Synthesis of enaminones 1a-g and 2a-b.

Ethyl (*E*)-2-acetyl-3-amino-4,4,4-trichloro-2-butenoate (1a). The product was purified using flash column chromatography. Ethyl acetate/*n*-hexane, 1:4. Yield: 14%, brown oil. ^1H NMR (400 MHz, $CDCl_3$): δ 4.19 (q, J = 7.2 Hz, 2H, $CH_3C\underline{H}_2O$), 2.23 (3H, s, 3H, CH_3CO), 1.29 (t, J = 7.0 Hz, 3H, $C\underline{H}_3CH_2O$), NH_2 signal was not observed; MS(ES^+) (*m*/*z*): 274.0 [M+H, ^{35}Cl, ^{35}Cl, ^{35}Cl, 38%]$^+$, 276.0 [M+H, ^{35}Cl, ^{35}Cl, ^{37}Cl, 48%]$^+$, 278.0 [M+H, ^{35}Cl, ^{37}Cl, ^{37}Cl, 23%]$^+$, 280.0 [M+H, ^{37}Cl, ^{37}Cl, ^{37}Cl, 6%]$^+$, 338.0 [M-H+Zn^{2+}, ^{35}Cl, ^{35}Cl, ^{35}Cl, 100%]$^+$, 340.0 [M-H+Zn^{2+}, ^{35}Cl, ^{35}Cl, ^{37}Cl, 50%]$^+$, 342.0 [M-H+Zn^{2+}, ^{35}Cl, ^{37}Cl, ^{37}Cl, 25%]$^+$.

Ethyl (*E*)-2-acetyl-3-amino-2-butenoate (1b). $SnCl_4$ could not be removed from enaminone, yield calculated based on a stoichiometric quantity of $SnCl_4$. Yield: 67%, orange solid. mp: 136-138 °C; ^1H NMR (400 MHz, DMSO-d_6): δ 10.77 (br s, 1H, $CH_3(N\underline{H}_2)C{=}CH$), 8.37 (br s, 1H, $CH_3(N\underline{H}_2)C{=}CH$), 4.11 (q, J = 7.1 Hz, 2H,

$CH_3C\underline{H}_2O$), 2.13 (s, 3H, CH_3), 2.11 (s, 3H, CH_3), 1.23 (t, *J* = 7.2 Hz, 3H, $C\underline{H}_3CH_2O$); ^{13}C NMR (100 MHz, DMSO-d_6): δ 195.3 ($CH_3\underline{C}O$), 169.5 ($CH_3(NH_2)\underline{C}$=C), 168.1 (EtO_2C), 102.1 ($CH_3(NH_2)C=\underline{C}$), 59.7 ($CH_3\underline{C}H_2O$), 30.4 ($\underline{C}H_3CO$), 22.4 ($\underline{C}H_3(NH_2)C=C$), 14.5 ($\underline{C}H_3CH_2O$); IR: 3263 (N-H), 3145 (N-H), 1651 (C=O), 1600 (C=C, conjugated) cm^{-1}; MS(ES^-) (*m/z*): 168.8 $[M-H, 100\%]^-$; MS(ES^+) (*m/z*): 130.9 $[M-Ac, 53\%]^+$, 171.0 $[M+H, 18\%]^+$, 194.1 $[M+Na, 25\%]^+$.

3-(1-Aminoethylidene)pentane-2,4-dione (1c). Yield: 61%, cream solid. Exists as a mixture of tautomers in a 9:1 ratio. mp: 129-132 ^oC; ^1H NMR (400 MHz, d_6-DMSO): δ 10.65 (br s, 1H, $CH_3(N\underline{H}_2)C=C$), 8.30 (br s, 1H, $CH_3(N\underline{H}_2)C=C$), 5.67 (s, 1H, $C=C\underline{H}_2$ of enol form), 4.88 (d, 1H, *J* = 1.4 Hz, $C=C\underline{H}_2$ of enol form), 2.17 (s, 6H, CH_3CO x 2), 2.04 (s, 3H, $C\underline{H}_3(NH_2)C=C$); 1.99 (s, 3H, $C\underline{H}_3C(OH)C$ of enol form), 1.91 (s, 3H, $C\underline{H}_3(OH)C$ of enol form); ^{13}C NMR (100 MHz, DMSO-d_6): δ 197.9 ($CH_3\underline{C}O$), 193.0 ($CH_3\underline{C}O$), 165.2 ($CH_3(NH_2)\underline{C}$=C), 113.1 ($CH_3(NH_2)C=\underline{C}$), 100.6 ($H_2C=C(OH)\underline{C}$ of enol form), 94.1 ($H_2\underline{C}=C(OH)C$ of enol form), 31.2 ($\underline{C}H_3CO$), 27.5 ($\underline{C}H_3CO$), 21.3 ($\underline{C}H_3(NH_2)C=C$); MS($ES^+$) (*m/z*): 142.1 $[M+H, 100\%]^+$, 164.1 $[M+Na, 29\%]^+$.

(*Z*)-2-Acetyl-3-amino-4,4,4-trichlorobut-2-enamide (1d). Yield: 54%, white solid. mp: 108-110 ^oC; ^1H NMR (300 MHz, $CDCl_3$): δ 8.75 (br s, 2H, NH_2), 6.02 (br s, 1H, $CONH_2$), 5.95 (br s, 1H, $CONH_2$), 2.38 (s, 3H, CH_3CO); ^{13}C NMR (75 MHz, $CDCl_3$): δ 197.2 ($CH_3\underline{C}O$), 169.9 ($CONH_2$), 156.7 ($Cl_3C(NH_2)\underline{C}$=C), 104.0 ($Cl_3C(NH_2)C=\underline{C}$), 93.3 ($CCl_3$), 28.5 ($\underline{C}H_3CO$); IR: 3417 (N-H), 3310 (N-H), 3209 (N-H), 1651 (C=O), 1592 (C=C, conjugated) cm^{-1}.

(*Z*)-2-Acetyl-3-amino-4,4,4-trichloro-*N*,*N*-dimethylbut-2-enamide (1e). Yield: 88%, cream solid. mp: 102-104 ^oC; ^1H NMR (300 MHz, $CDCl_3$): δ 8.56 (br s, 1H,

$Cl_3C(NH_2)C{=}C$), 2.97 (d, J = 3.3 Hz, 6H, $CON(CH_3)_2$), 2.14 (s, 3H, CH_3CO); ^{13}C NMR (100 MHz, $CDCl_3$): δ 196.7 ($CH_3\underline{C}O$), 168.6 ($Cl_3C(NH_2)\underline{C}{=}C$), 155.6 ($\underline{C}ON(CH_3)_2$), 103.2 ($Cl_3C(NH_2)C{=}\underline{C}$), 93.3 ($CCl_3$), 38.8 ($CON(\underline{C}H_3)_2$), 35.1 ($CON(\underline{C}H_3)_2$), 27.9 ($\underline{C}H_3CO$); IR: 3282 (N-H), 1602 (C=C, conjugated), 1497 (C=C-NH_2) cm^{-1}; MS(ES^-) (*m*/*z*): 270.9 [M-H, ^{35}Cl, ^{35}Cl, ^{35}Cl, 18%]$^-$, 273.0 [M-H, ^{35}Cl, ^{35}Cl, ^{37}Cl, 22%]$^-$, 275.0 [M-H, ^{35}Cl, ^{37}Cl, ^{37}Cl, 5%]$^-$, 277.0 [M-H, ^{37}Cl, ^{37}Cl, ^{37}Cl, 3%]$^-$; MS(ES^+) (*m*/*z*): 273.0 [M+H, ^{35}Cl, ^{35}Cl, ^{35}Cl, 100%]$^+$, 275.0 [M+H, ^{35}Cl, ^{35}Cl, ^{37}Cl, 54%]$^+$, 277.0 [M+H, ^{35}Cl, ^{37}Cl, ^{37}Cl, 83%]$^+$, 279.0 [M+H, ^{37}Cl, ^{37}Cl, ^{37}Cl, 12%]$^+$, 295.0 [M+Na, ^{35}Cl, ^{35}Cl, ^{35}Cl, 49%]$^+$, 297.0 [M+Na, ^{35}Cl, ^{35}Cl, ^{37}Cl, 52%]$^+$, 299.0 [M+Na, ^{35}Cl, ^{37}Cl, ^{37}Cl, 12%]$^+$, 301.0 [M+Na, ^{37}Cl, ^{37}Cl, ^{37}Cl, 2%]$^+$, 311.0 [M+K, ^{35}Cl, ^{35}Cl, ^{35}Cl, 23%]$^+$, 313.0 [M+K, ^{35}Cl, ^{35}Cl, ^{37}Cl, 20%]$^+$, 315.0 [M+K, ^{35}Cl, ^{37}Cl, ^{37}Cl, 8%]$^+$, 317.0 [M+K, ^{37}Cl, ^{37}Cl, ^{37}Cl, 1%]$^+$; HRMS(ES^+) (*m*/*z*): $[M+Na]^+$ calcd. for $C_8H_{11}{}^{35}Cl_3N_2O_2Na$, 294.9778; found, 294.9789, error: 3.7 ppm.

(*Z*)-2-Cyano-3-amino-4,4,4-trichlorobut-2-enamide (1f). Cyanoacetamide (1.68 g, 20 mmol) and trichloroacetonitrile (2.00 mL, 20 mmol) were added to a suspension of sodium acetate (1.64 g, 20 mmol) and anhydrous ethanol (100 ml). The reaction mixture was stirred at room temperature under N_2 for 16 h. The reaction mixture was dissolved in water, the precipitate was filtered and recrystallized from EtOH (Ibrahim et al., 1985). Yield: 0.45g (10%), cream solid. mp: 124-126 ^oC (lit.(Ibrahim et al., 1985) 152 ^oC); ^1H NMR (300 MHz, d_4-MeOH): δ 11.08 (br s, 1H, NH_2), 8.79 (br s, 1H, NH_2), 7.43 (br s, 1H, CONH), 7.20 (br s, 1H, CONH); IR: 3323 (N-H), 3198 (N-H), 2197 (C≡N), 1660 (C=O), 1593 (C=C, conjugated) cm^{-1}; MS(ES^-) (*m*/*z*): 225.9 [M-H, ^{35}Cl, ^{35}Cl, ^{35}Cl, 71%]$^-$, 227.9 [M-H, ^{35}Cl, ^{35}Cl, ^{37}Cl, 100%]$^-$, 229.9 [M-H, ^{35}Cl, ^{37}Cl, ^{37}Cl, 22%]$^-$, 231.9 [M-H, ^{37}Cl, ^{37}Cl, ^{37}Cl, 3%]$^-$. HRMS($APCI^+$) (*m*/*z*): $[M+H]^+$ calcd. for $C_5H_5{}^{35}Cl_3N_3O$, 227.9493; found, 227.9493, error: 0.0 ppm.

***tert*-Butyl (*Z*)-2-acetyl-3-amino-4,4,4-trichlorobut-2-enoate (1g)**. Yield: 88%, cream solid. mp: 75-76 °C; ^{1}H NMR (300 MHz, $CDCl_3$): δ 2.31 (s, 3H, CH_3CO), 1.55 (s, 9H, $(CH_3)_3C$); ^{13}C NMR (75 MHz, $CDCl_3$): δ 196.7 ($CH_3\underline{C}O$), 167.3 (C=$\underline{C}$(NH_2)CCl_3), 157.5 (CONH), 104.1 ($\underline{C}$=C(NH_2)CCl_3), 93.4 (CCl_3), 82.4 ($(CH_3)_3\underline{C}$), 29.1 ($\underline{C}H_3CO$), 27.7 ($(\underline{C}H_3)_3C$); IR: 3310 (N-H), 2979 (N-H), 1712 (C=O), 1673 (C=O), 1605 (C=C, conjugated) cm^{-1}; MS(ES^-) (*m*/*z*): 300.0 [M-H, ^{35}Cl, ^{35}Cl, ^{35}Cl, 86%]$^-$, 301.9 [M-H, ^{35}Cl, ^{35}Cl, ^{37}Cl, 100%]$^-$, 304.0 [M-H, ^{35}Cl, ^{37}Cl, ^{37}Cl, 39%]$^-$, 306.0 [M-H, ^{37}Cl, ^{37}Cl, ^{37}Cl, 6%]$^-$; MS(ES^+) (*m*/*z*): 227.8 [M-O^{t}Bu, ^{35}Cl, ^{35}Cl, ^{35}Cl, 72%]$^+$, 229.8 [M-O^{t}Bu, ^{35}Cl, ^{35}Cl, ^{37}Cl, 74%]$^+$, 231.8 [M-O^{t}Bu, ^{35}Cl, ^{37}Cl, ^{37}Cl, 25%]$^+$, 233.8 [M-O^{t}Bu, ^{37}Cl, ^{37}Cl, ^{37}Cl, 2%]$^+$, 323.9 [M+Na, ^{35}Cl, ^{35}Cl, ^{35}Cl, 10%]$^+$, 325.9 [M+Na, ^{35}Cl, ^{35}Cl, ^{37}Cl, 13%]$^+$, 327.9 [M+Na, ^{35}Cl, ^{37}Cl, ^{37}Cl, 3%]$^+$, 329.9 [M+Na, ^{37}Cl, ^{37}Cl, ^{37}Cl, 1%]$^+$; HRMS(ES^+) (*m*/*z*): [M+Na]$^+$ calcd. for $C_{10}H_{14}{}^{35}Cl_3NO_3Na$, 323.9931; found, 323.9929, error: 0.6 ppm.

(*Z*)-4-Amino-5,5,5-trichloropent-3-en-2-one (2a). Yield: 52%, pale orange solid. mp: 66-68 °C (lit.(Coenen et al., 1965) 67-68 °C); ^{1}H NMR (400 MHz, $CDCl_3$): δ 5.85 (t, *J* = 1.2 Hz, 1H, $Cl_3C(NH_2)C$=C$\underline{H}$), 2.16 (s, 3H, CH_3CO), NH_2 signal was not observed; IR: 3299 (N-H), 3159 (N-H), 1621 (C=O), 1600 (C=C, conjugated), 1505 (C=C-NH_2) cm^{-1}; MS(ES^+) (*m*/*z*): 201.9 [M+H, ^{35}Cl, ^{35}Cl, ^{35}Cl, 96%]$^+$, 203.9 [M+H, ^{35}Cl, ^{35}Cl, ^{37}Cl, 83%]$^+$, 205.9 [M+H, ^{35}Cl, ^{37}Cl, ^{37}Cl, 32%]$^+$, 207.9 [M+H, ^{37}Cl, ^{37}Cl, ^{37}Cl, 6%]$^+$, 402.1 [2 x M, ^{35}Cl, ^{35}Cl, ^{35}Cl, 100%]$^+$.

(*Z*)-3-Amino-4,4,4-trichlorobut-2-enamide (2b). Yield: 39%, cream solid. mp: 95-97 °C (dec); ^{1}H NMR (400 MHz, $CDCl_3$): δ 6.72 (br s, 1H, NH), 5.33 (s, 1H, $Cl_3C(NH_2)C$=C$\underline{H}$), 5.30 (br s, 1H, NH), 1 x NH signal not observed; ^{13}C NMR (100 MHz, $CDCl_3$): δ 170.6 (C=$\underline{C}$(NH_2)CCl_3), 156.8 (CONH), 94.3 (CCl_3), 86.1 ($\underline{C}$=C(NH_2)CCl_3); IR: 3479 (N-H), 3412 (N-H), 3277 (N-H), 3154 (N-H), 1649 (C=O),

1623 (C=O), 1589 (C=C), 1539 (C=C-NH_2) cm^{-1}; MS(ES^-) (*m/z*): 201.9 [M-H, ^{35}Cl, ^{35}Cl, ^{35}Cl, 79%]$^-$, 203.9 [M-H, ^{35}Cl, ^{35}Cl, ^{37}Cl, 46%]$^-$, 205.9 [M-H, ^{35}Cl, ^{37}Cl, ^{37}Cl, 30%]$^-$, 207.9 [M-H, ^{37}Cl, ^{37}Cl, ^{37}Cl, 11%]$^-$; MS(ES^+) (*m/z*): 203.0 [M+H, ^{35}Cl, ^{35}Cl, ^{35}Cl, 71%]$^+$, 204.9 [M+H, ^{35}Cl, ^{35}Cl, ^{37}Cl, 57%]$^+$, 206.9 [M+H, ^{35}Cl, ^{37}Cl, ^{37}Cl, 9%]$^+$, 208.9 [M+H, ^{37}Cl, ^{37}Cl, ^{37}Cl, 2%]$^+$; HRMS($APCI^+$) (*m/z*): $[M+H]^+$ calcd. for $C_4H_6{}^{35}Cl_3N_2O$, 202.9540; found, 202.9545, error: 2.5 ppm.

1.3 General procedure for oxazaborine reaction

To prepare DPBA, 2-APB (0.50 g, 2.22 mmol) was dissolved in Me_2CO (5 ml) and MeOH (5 ml) in a flask covered in aluminium foil. HCl was added dropwise until the solution reached pH 1-2. The reaction mixture was stirred at room temperature for 1h. Et_2O (15 ml) was added and extracted with Et_2O. The organic layer was dried over $MgSO_4$ and concentrated *in vacuo* to give DPBA (0.47 g, 61%) as an orange oil (Hosoya et al., 2006).

An appropriate enaminone (3 eq) was added to a solution of DPBA (1 eq) in anhydrous THF (5 ml). The mixture was stirred at 50°C under N_2 for 16h. The mixture was concentrated *in vacuo* and purified by flash column chromatography. The collected fractions were combined, evaporated *in vacuo* and stirred in cold *n*-hexane (15 ml) for 30 min. The precipitate was then filtered and dried under vacuum to give the corresponding oxazaborine products. Borylation of enaminodiones yields oxazaborine compounds of type **A** configuration, deacetylated enaminones of type **B** configuration and (*Z*)-2-cyano-3-amino-4,4,4-trichlorobut-2-enamide of type **C** configuration (NBC18) (Fig. 3A,B) (Vasilev et al., 1992, Vasilev et al., 1994).

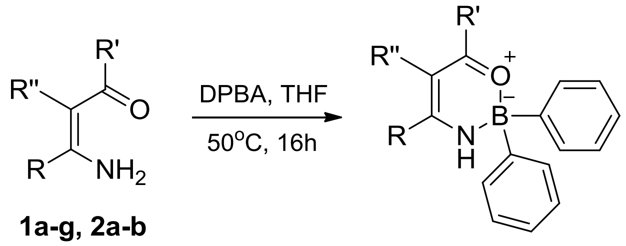

1a-g, 2a-b

R	R'	R"		R	R'	R"	
CCl_3	Me	COOEt	**BC7**	CCl_3	Me	$CONMe_2$	**NBC11**
CCl_3	Me	H	**BC23**	CCl_3	NH_2	CN	**NBC18**
CCl_3	Me	COMe	**NBC1**	Ph	Me	COMe	**NBC23**
Me	Me	COOEt	**NBC2**	CCl_3	NH_2	$CONH_2$	**NBC24**
Me	Me	H	**NBC3**	CCl_3	O^tBu	COMe	**NBC25**
Me	Me	COMe	**NBC4**	SCH_2CH_3	O^tBu	COMe	**NBC26**
CCl_3	NH_2	H	**NBC5**	^tBu	Me	H	**NBC28**
CCl_3	NH_2	COMe	**NBC6**				

Scheme 2 | Synthesis of BC7/23 and NBC1-6, NBC11, NBC18, NBC23-26 and NBC28.

5-(Ethoxycarbonyl)-6-methyl-2,2-diphenyl-4-(trichloromethyl)-2,3-dihydro-1,3,2-oxazaborinin-1-ium-2-uide (BC-7, 13). Ethyl acetate/*n*-hexane, 1:49. Yield: 8%, yellow solid. mp: 109-110 °C; ^{1}H NMR (400 MHz, $CDCl_3$): δ 8.04 (br s, 1H, $Cl_3C(NH)C{=}C$), 7.37 (dd, *J* = 8.0 Hz & 1.6 Hz, 4H, B-Ph(*o*)), 7.23-7.34 (m, 6H, B-Ph(*m*/*p*)), 4.18 (q, *J* = 7.2 Hz, 2H, $CH_3C\underline{H_2}O$), 2.50 (s, 3H, CH_3CO), 1.30 (t, *J* = 7.2 Hz, 3H, $C\underline{H_3}CH_2O$); ^{13}C NMR (100 MHz, $CDCl_3$): δ 188.9 ($CH_3\underline{C}O$), 165.4 ($Cl_3C(NH)\underline{C}{=}C$), 164.3 ($EtO_2C$), 131.8 (B-Ph(*o*)), 127.6 (B-Ph(*m*)), 127.0 (B-Ph(*p*)), 102.3 ($Cl_3C(NH)C{=}\underline{C}$), 93.4 ($CCl_3$), 61.7 ($CH_3\underline{C}H_2O$), 24.4 ($\underline{C}H_3CO$), 13.7 ($\underline{C}H_3CH_2O$), B-Ph(*i*) quaternary signal not observed; ^{11}B{^{1}H} (128 MHz, $CDCl_3$): δ 3.94; IR: 3306 (N-H), 1709 (C=O), 1592 (C=C, conjugated) cm^{-1}; MS(ES^-) (*m/z*): 435.2 [M-H, ^{10}B, ^{35}Cl, ^{35}Cl, ^{35}Cl, 10%]$^-$, 436.2 [M-H, ^{11}B, ^{35}Cl, ^{35}Cl, ^{35}Cl, 58%]$^-$, 437.2 [M-H, ^{10}B, ^{35}Cl, ^{35}Cl, ^{37}Cl, 29%]$^-$, 438.2 [M-H, ^{11}B ^{35}Cl, ^{35}Cl, ^{37}Cl, 58%]$^-$, 439.2 [M-H,

^{10}B, ^{35}Cl, ^{37}Cl, ^{37}Cl, 17%]$^-$, 440.2 [M-H, ^{11}B, ^{35}Cl, ^{37}Cl, ^{37}Cl, 26%]$^-$, 441.3 [M-H, ^{10}B, ^{37}Cl, ^{37}Cl, ^{37}Cl, 8%]$^-$, 442.2 [M-H, ^{11}B, ^{37}Cl, ^{37}Cl, ^{37}Cl, 3%]$^-$; HRMS(ES$^-$) (*m/z*): [M-H]$^-$ calcd. for $C_{20}H_{18}{}^{11}B^{35}Cl_3NO_3$, 436.0451; found, 436.0431, error: 4.6 ppm.

6-Methyl-2,2-diphenyl-4-(trichloromethyl)-2,3-dihydro-1,3,2-oxazaborinin-1-ium-2-uide (BC-23, 14). Ethyl acetate/hexane, 1:19. Yield: 56%, yellow solid. mp: 113-114 ^{o}C (lit.[19] 112.5-113.5 ^{o}C); ^{1}H NMR (400 MHz, $CDCl_3$): δ 7.39 (dd, *J* = 7.7 Hz & 1.4 Hz, 4H, B-Ph(*o*)), 7.21-7.35 (m, 6H, B-Ph(*m*/*p*)), 5.80 (d, *J* = 2.4 Hz, 1H, Cl_3C(NH)C=C<u>*H*</u>), 2.25 (s, 3H, CH_3CO), NH signal not observed; ^{13}C NMR (100 MHz, $CDCl_3$): δ 186.3 (CH_3<u>*C*</u>O), 165.8 (Cl_3C(NH)<u>*C*</u>=CH), 131.7 (B-Ph(*o*)), 127.5 (B-Ph(*m*)), 126.8 (B-Ph(*p*)), 92.9 (CCl_3), 91.6 (Cl_3C(NH)C=<u>*C*</u>H), 24.7 (<u>*C*</u>H_3CO), B-Ph(*i*) quaternary signal not observed; ^{11}B{^{1}H} (128 MHz, $CDCl_3$): δ 4.38; IR: 3321 (N-H), 1600 (C=C, conjugated), 1523 (C=C-NH) cm^{-1}; MS(ES$^-$) (*m/z*): 363.0 [M-H, ^{10}B, ^{35}Cl, ^{35}Cl, ^{35}Cl, 19%]$^-$, 364.0 [M-H, ^{11}B, ^{35}Cl, ^{35}Cl, ^{35}Cl, 100%]$^-$, 365.0 [M-H, ^{10}B, ^{35}Cl, ^{35}Cl, ^{37}Cl, 32%]$^-$, 366.0 [M-H, ^{11}B, ^{35}Cl, ^{35}Cl, ^{37}Cl, 95%]$^-$, 367.0 [M-H, ^{10}B, ^{35}Cl, ^{37}Cl, ^{37}Cl, 19%]$^-$, 368.0 [M-H, ^{11}B, ^{35}Cl, ^{37}Cl, ^{37}Cl, 28%]$^-$, 369.1 [M-H, ^{10}B, ^{37}Cl, ^{37}Cl, ^{37}Cl, 8%]$^-$, 370.1 [M-H, ^{11}B, ^{37}Cl, ^{37}Cl, ^{37}Cl, 3%]$^-$; MS(ES$^+$) (*m/z*): 286.9 [M-Ph, ^{10}B, ^{35}Cl, ^{35}Cl, ^{35}Cl, 18%]$^+$, 288.0 [M-Ph, ^{11}B, ^{35}Cl, ^{35}Cl, ^{35}Cl, 100%]$^+$, 289.0 [M-Ph, ^{10}B, ^{35}Cl, ^{35}Cl, ^{37}Cl, 30%]$^+$, 289.9 [M-Ph, ^{11}B, ^{35}Cl, ^{35}Cl, ^{37}Cl, 94%]$^+$, 290.9 [M-Ph, ^{10}B, ^{35}Cl, ^{37}Cl, ^{37}Cl, 13%]$^+$, 291.9 [M-Ph, ^{11}B, ^{35}Cl, ^{37}Cl, ^{37}Cl, 29%]$^+$, 293.0 [M-Ph, ^{10}B, ^{37}Cl, ^{37}Cl, ^{37}Cl, 3%]$^+$, 294.0 [M-Ph, ^{11}B, ^{37}Cl, ^{37}Cl, ^{37}Cl, 5%]$^+$, 365.0 [M+H, ^{10}B, ^{35}Cl, ^{35}Cl, ^{35}Cl, 3%]$^+$, 366.0 [M+H, ^{11}B, ^{35}Cl, ^{35}Cl, ^{35}Cl, 12%]$^+$, 387.0 [M+Na, ^{10}B, ^{35}Cl, ^{35}Cl, ^{35}Cl, 16%]$^+$ 388.0 [M+Na, ^{11}B, ^{35}Cl, ^{35}Cl, ^{35}Cl, 46%]$^+$, 389.0 [M+Na, ^{10}B, ^{35}Cl, ^{35}Cl, ^{37}C;, 16%]$^+$, 390.0 [M+Na, ^{11}B, ^{35}Cl, ^{35}Cl, ^{37}Cl, 42%]$^+$, 391.0 [M+Na, ^{10}B, ^{35}Cl, ^{37}Cl, ^{37}Cl, 10%]$^+$, 392.0 [M+Na, ^{11}B, ^{35}Cl, ^{37}Cl, ^{37}Cl, 13%]$^+$, 393.0 [M+Na, ^{10}B, ^{37}Cl, ^{37}Cl, ^{37}C, 4%]$^+$, 394.0 [M+Na, ^{11}B, ^{37}Cl, ^{37}Cl, ^{37}Cl, 2%]$^+$, 403.0 [M+K, ^{10}B, ^{35}Cl, ^{35}Cl, ^{35}Cl, 4%]$^+$,

404.0 [M+K, ^{11}B, ^{35}Cl, ^{35}Cl, ^{35}Cl, 20%]$^{+}$; 405.0 [M+K, ^{10}B, ^{35}Cl, ^{35}Cl, ^{37}Cl, 6%]$^{+}$, 406.0 [M+K, ^{11}B, ^{35}Cl, ^{35}Cl, ^{37}Cl, 19%]$^{+}$, 407.0 [M+K, ^{10}B, ^{35}Cl, ^{37}Cl, ^{37}Cl, 5%]$^{+}$, 408.0 [M+K, ^{11}B, ^{35}Cl, ^{37}Cl, ^{37}Cl, 7%]$^{+}$, 409.0 [M+K, ^{10}B, ^{37}Cl, ^{37}Cl, ^{37}Cl, 2%]$^{+}$, 410.0 [M+K, ^{37}Cl, ^{37}Cl, ^{37}Cl, 2%]$^{+}$; HRMS(ES^{-}) (*m*/*z*): [M-H]$^{-}$ calcd. for $C_{17}H_{14}{}^{11}B^{35}Cl_3NO$, 364.0240; found, 364.0243, error: 0.8 ppm.

5-Acetyl-6-methyl-2,2-diphenyl-4-(trichloromethyl)-2,3,-dihydro-1,3,2-oxazaborinin-1-ium-2-uide (NBC1). Crude enaminone product was directly borylated using DPBA due to difficulty in purification. Ethyl acetate/*n*-hexane, 1:4. Yield: 92%, yellow oil. ^{1}H NMR (400 MHz, $CDCl_3$): δ 7.30 (dd, *J* = 7.8 Hz & 1.8 Hz, 4H, B-Ph(*o*)), 7.16-7.27 (m, 6H, B-Ph(*m*/*p*), 2.26 (s, 3H, CH_3CO), 2.24 (s, 3H, CH_3CO), NH signal not observed; ^{13}C NMR (100 MHz, $CDCl_3$): δ 198.9 (CH_3*C̲*O), 185.4 (CH_3*C̲*O), 163.3 (Cl_3C(NH)*C̲*=C), 131.8 (B-Ph(*o*)), 127.6 (B-Ph(*m*)), 127.1 (B-Ph(*p*)), 111.6 (Cl_3C(NH)C=*C̲*), 93.0 (CCl_3), 34.5 (*C̲*H_3CO), 23.6 (*C̲*H_3CO), B-Ph(*i*) quaternary signal not observed; IR: 3343 (N-H), 1690 (C=O), 1578 (C=C, conjugated) cm^{-1}; MS(ES^{-}) (*m*/*z*): 405.1 [M-H, ^{10}B, ^{35}Cl, ^{35}Cl, ^{35}Cl, 13%]$^{-}$, 406.2 [M-H, ^{11}B, ^{35}Cl, ^{35}Cl, ^{35}Cl, 78%]$^{-}$, 407.2 [M-H, ^{10}B, ^{35}Cl, ^{35}Cl, ^{37}Cl, 21%]$^{-}$, 408.1 [M-H, ^{11}B, ^{35}Cl, ^{35}Cl, ^{37}Cl, 100%]$^{-}$, 409.1 [M-H, ^{10}B, ^{35}Cl, ^{37}Cl, ^{37}Cl, 22%]$^{-}$, 410.0 [M-H, ^{11}B, ^{35}Cl, ^{37}Cl, ^{37}Cl, 54%]$^{-}$, 411.2 [M-H, ^{10}B, ^{37}Cl, ^{37}Cl, ^{37}Cl, 7%]$^{-}$, 412.1 [M-H, ^{11}B, ^{37}Cl, ^{37}Cl, ^{37}Cl, 4%]$^{-}$; HRMS(ES^{-}) (*m*/*z*): [M-H]$^{-}$ calcd. for $C_{19}H_{16}{}^{11}B^{35}Cl_3NO_2$, 406.0345; found, 406.0353, error: 2.0 ppm.

5-(Ethoxycarbonyl)-4,6-dimethyl-2,2-diphenyl-2,3-dihydro-1,3,2-oxazaborinin-1-ium-2-uide (NBC2). Ethyl acetate/*n*-hexane, 1:11. Yield: 82%, white solid. mp: 112-113 ^{o}C; ^{1}H NMR (400 MHz, $CDCl_3$): δ 7.26 (dd, *J* = 7.8 Hz & 1.4 Hz, 4H, B-Ph(*o*)), 7.11-7.23 (m, 6H, B-Ph(*m*/*p*)), 4.11 (q, *J* = 7.2 Hz, 2H, CH_3C*H̲*$_2$O), 2.42 (s, 3H, CH_3CO), 2.38 (d, *J* = 0.8 Hz, 3H, C*H̲*$_3$(NH)C=C), 1.22 (t, *J* = 7.2 Hz, 3H, C*H̲*$_3$$CH_2$O),

NH signal not observed; ^{13}C NMR (75 MHz, $CDCl_3$): δ 186.9 ($CH_3\underline{C}O$), 170.9 ($CH_3(NH)\underline{C}$=C), 166.3 (EtO_2C), 131.7 (B-Ph(*o*)), 127.4 (B-Ph(*m*)), 126.6 (B-Ph(*p*)), 103.7 ($CH_3(NH)C=\underline{C}$), 60.4 ($CH_3\underline{C}H_2O$), 26.4 ($\underline{C}H_3CO$), 25.7 ($\underline{C}H_3$(NH)C=C), 14.3 ($\underline{C}H_3CH_2O$), B-Ph(*i*) quaternary signal not observed; $^{11}B\{^1H\}$ (128 MHz, $CDCl_3$): δ 2.63; IR: 3316 (N-H), 1701 (C=O), 1603 (C=C, conjugated), 1489 (C=C-NH) cm^{-1}; MS(ES^-) (*m/z*): 333.2 [M-H, ^{10}B, 7%]$^-$, 334.2 [M-H, ^{11}B, 76%]$^-$, 370.3 [M+^{35}Cl, 6%]$^-$, 372.3 [M+^{37}Cl, 2%]$^-$; MS(ES^+) (*m/z*): 257.2 [M-Ph, ^{10}B, 21%]$^+$, 258.2 [M-Ph, ^{11}B, 100%]$^+$, 335.4 [M+H, ^{10}B, 5%]$^+$, 336.3 [M+H, ^{11}B, 22%]$^+$, 358.3 [M+Na, ^{11}B, 28%]$^+$, 374.3 [M+K, ^{11}B, 18%]$^+$; HRMS(ES^+) (*m/z*): $[M+NH_4]^+$ calcd. for $C_{20}H_{26}{}^{11}BN_2O_3$, 353.2031; found, 353.2021, error: 2.8 ppm.

4,6-Dimethyl-2,2-diphenyl-2,3-dihydro-1,3,2-oxazaborinin-1-ium-2-uide (NBC3). Crude enaminone product was directly borylated using DPBA due to difficulty in purification. Ethyl acetate/*n*-hexane, 2:23. Yield: 10%, white solid. mp: 109-110 ^oC; ^1H NMR (300 MHz, $CDCl_3$): δ 7.37 (d, *J* = 6.9 Hz, 4H, B-Ph(*o*)), 7.14-7.31 (m, 6H, B-Ph(*m/p*)), 6.67 (br s, 1H, $CH_3(N\underline{H})C=CH$), 5.11 (d, *J* = 0.9 Hz, 1H, $CH_3(NH)C=C\underline{H}$), 2.07 (s, 3H, $C\underline{H}_3$(NH)C=CH), 2.04 (s, 3H, CH_3CO); ^{13}C NMR (75 MHz, $CDCl_3$): δ 179.7 ($CH_3\underline{C}O$), 169.4 ($CH_3(NH)\underline{C}$=C), 131.7 (B-Ph(*o*)), 127.3 (B-Ph(*m*)), 126.3 (B-Ph(*p*)), 96.7 ($CH_3(NH)C=\underline{C}$), 23.8 ($\underline{C}H_3$(NH)C=C), 23.7 ($\underline{C}H_3CO$), B-Ph(*i*) quaternary signal not observed; $^{11}B\{^1H\}$ (128 MHz, $CDCl_3$): δ 2.97; IR: 3339 (N-H), 1621 (C=O), 1537 (C=C-NH) cm^{-1}; MS(ES^-) (*m/z*): 261.1 [M-H, ^{10}B, 12%]$^-$, 262.2 [M-H, ^{11}B, 100%]$^-$; MS(ES^+) (*m/z*): 185.2 [M-Ph, ^{10}B, 28%]$^+$, 186.2 [M-Ph, ^{11}B, 100%]$^+$, 263.3 [M+H, ^{10}B, 3%]$^+$, 264.3 [M+H, ^{11}B, 5%]$^+$, 285.2 [M+Na, ^{10}B, 2%]$^+$, 286.2 [M+Na, ^{11}B, 8%]$^+$; HRMS(ES^+) (*m/z*): $[M+Na]^+$ calcd. for $C_{17}H_{18}{}^{11}BNONa$, 286.1374; found, 286.1392, error: 6.3 ppm.

5-Acetyl-4,6-dimethyl-2,2-diphenyl-2,3-dihydro-1,3,2-oxazaborinin-1-ium-2-uide (NBC4). Ethyl acetate/*n*-hexane, 1:4. Yield: 40%, white solid. mp: 88-89 °C; ^{1}H NMR (400 MHz, $CDCl_3$): δ 7.26 (dd, *J* = 8.2 Hz & 1.4 Hz, 4H, B-Ph(*o*)), 7.11-7.23 (m, 6H, B-Ph(*m*/*p*)), 2.32 (s, 3H, CH_3CO), 2.28 (d, *J* = 0.8 Hz, 3H, $C\underline{H}_3$(NH)C=C), 2.20 (s, 3H, $C\underline{H}_3CO$), NH signal not observed; ^{13}C NMR (75 MHz, $CDCl_3$): δ 198.2 ($CH_3\underline{C}O$), 183.5 ($CH_3\underline{C}O$), 169.9 (CH_3(NH)$\underline{C}$=C), 131.7 (B-Ph(*o*)), 127.4 (B-Ph(*m*)), 126.6 (B-Ph(*p*)), 114.9 (CH_3(NH)C=$\underline{C}$), 32.8 ($\underline{C}H_3CO$), 25.4 ($\underline{C}H_3$(NH)C=C), 25.0 ($\underline{C}H_3CO$), B-Ph(*i*) quaternary signal not observed; ^{11}B{^{1}H} (128 MHz, $CDCl_3$): δ 2.64; IR: 3283 (N-H), 1654 (C=O), 1604 (C=C, conjugated) cm^{-1}; MS(ES$^-$) (*m*/*z*): 303.3 [M-H, ^{10}B, ^{12}C, 15%]$^-$, 304.2 [M-H, ^{11}B, ^{12}C, 100%]; MS(ES$^+$) (*m*/*z*): 227.3 [M-Ph, ^{10}B, ^{12}C, 10%]$^+$, 228.2 [M-Ph, ^{11}B, ^{12}C, 40%]$^+$, 305.2 [M+H, ^{10}B, ^{12}C, 3%]$^+$, 306.4 [M+H, ^{11}B, ^{12}C, 45%]$^+$, 328.3 [M+Na, ^{11}B, ^{12}C, 30%]$^+$, 344.4 [M+K, ^{11}B, ^{12}C, 9%]$^+$; HRMS(APCI$^+$) (*m*/*z*): [M+H]$^+$ calcd. for $C_{19}H_{21}{}^{11}BNO_2$, 306.1660; found, 306.1654, error: 2.0 ppm.

6-Amino-2,2-diphenyl-4-(trichloromethyl)-2,3-dihydro-1,3,2-oxazaborinin-1-ium-2-uide (NBC5). Ethyl acetate/*n*-hexane, 1:4. Yield: 81%, cream solid. mp: 148-149 °C; ^{1}H NMR (300 MHz, $CDCl_3$): δ 7.34 (d, *J* = 6.3 Hz, 4H, B-Ph(*o*)), 7.13-7.29 (m, 6H, B-Ph(*m*/*p*)), 6.29 (br s, 1H, Cl_3C(N$\underline{H}$)C=CH), 5.28 (br s, 2H, $CONH_2$), 5.14 (s, 1H, Cl_3C(NH)C=C$\underline{H}$); ^{13}C NMR (75 MHz, $CDCl_3$): δ 171.0 ($CONH_2$), 164.7 (Cl_3C(NH)$\underline{C}$=C), 132.1 (B-Ph(*o*)), 127.5 (B-Ph(*m*)), 126.7 (B-Ph(*p*)), 93.8 (CCl_3), 86.1 (Cl_3C(NH)C=$\underline{C}$), B-Ph(*i*) quaternary signal not observed; ^{11}B{^{1}H} (128 MHz, $CDCl_3$): δ 4.32; IR: 3485 (N-H), 3363 (N-H), 1619 (C=O), 1577 (C=C, conjugated), 1542 (C=C-NH) cm^{-1}; MS(ES$^-$) (*m*/*z*): 364.1 [M-H, ^{10}B, ^{35}Cl, ^{35}Cl, ^{35}Cl, 8%]$^-$, 365.1 [M-H, ^{11}B, ^{35}Cl, ^{35}Cl, ^{35}Cl, 100%]$^-$, 366.0 [M-H, ^{10}B, ^{35}Cl, ^{35}Cl, ^{37}Cl, 16%]$^-$, 367.0 [M-H, ^{11}B, ^{35}Cl, ^{35}Cl, ^{37}Cl, 28%]$^-$, 368.0 [M-H, ^{10}B, ^{35}Cl, ^{37}Cl, ^{37}Cl, 8%]$^-$, 369.0 [M-H, ^{11}B, ^{35}Cl, ^{37}Cl, ^{37}Cl, 27%]$^-$, 370.1 [M-H, ^{10}B, ^{37}Cl, ^{37}Cl, ^{37}Cl, 6%]$^-$, 371.3 [M-H, ^{11}B, ^{37}Cl, ^{37}Cl, ^{37}Cl, 2%]$^-$;

MS(ES)$^+$ (m/z): 389.1 [M+Na, ^{11}B, ^{35}Cl, ^{35}Cl, ^{35}Cl, 5%]$^+$, 391.1 [M+Na, ^{11}B, ^{35}Cl, ^{35}Cl, ^{37}Cl, 8%]$^+$, 393.1 [M+Na, ^{11}B, ^{35}Cl, ^{37}Cl, ^{37}Cl, 3%]$^+$; HRMS(ES$^+$) (m/z): [M+H]$^+$ calcd. for $C_{16}H_{15}{}^{11}B^{35}Cl_3N_2O$, 367.0338; found, 367.0328, error: 2.7 ppm.

5-Acetyl-6-amino-2,2-diphenyl-4-(trichloromethyl)-2,3-dihydro-1,3,2-oxazaborinin-1-ium-2-uide (NBC6, 15). Ethyl acetate/*n*-hexane, 1:9. Yield: 34%, yellow solid. mp: 134-135 ^oC; ^{1}H NMR (300 MHz, $CDCl_3$): δ 9.16 (br s, 1H, $CONH_2$), 7.51 (br s, 1H, Cl_3C(NH)C=C), 7.31 (d, $J = 6.0$ Hz, 4H, B-Ph(*o*)), 7.13-7.25 (m, 6H, B-Ph(*m*/*p*)), 5.91 (br s, 1H, $CONH_2$), 2.20 (s, 3H, CH_3CO); ^{13}C NMR (75 MHz, $CDCl_3$): δ 197.1 ($CH_3\underline{C}O$), 168.2 ($CONH_2$), 164.5 (Cl_3C(NH)$\underline{C}$=C), 130.9 (B-Ph(*o*)), 126.4 (B-Ph(*m*)), 125.8 (B-Ph(*p*)), 97.1 (Cl_3C(NH)C=$\underline{C}$), 94.0 (CCl_3), 32.9 ($\underline{C}H_3CO$), B-Ph(*i*) quaternary signal not observed; ^{11}B{^{1}H} (128 MHz, $CDCl_3$): δ 2.42; IR: 3390 (N-H), 3329 (N-H), 1647 (C=O), 1606 (C=C, conjugated), 1552 (C=C-NH) cm^{-1}; MS(ES$^-$) (m/z): 202.0 [M-$C_{14}H_{11}$BO, ^{35}Cl, ^{35}Cl, ^{35}Cl, 91%]$^-$, 204.0 [M-$C_{14}H_{11}$BO, ^{35}Cl, ^{35}Cl, ^{37}Cl, 100%]$^-$, 206.0 [M-$C_{14}H_{11}$BO, ^{35}Cl, ^{37}Cl, ^{37}Cl, 15%]$^-$, 208.0 [M-$C_{14}H_{11}$BO, ^{37}Cl, ^{37}Cl, ^{37}Cl, 3%]$^-$; 406.1 [M-H, ^{10}B, ^{35}Cl, ^{35}Cl, ^{35}Cl, 20%]$^-$, 407.1 [M-H, ^{11}B, ^{35}Cl, ^{35}Cl, ^{35}Cl, 61%]$^-$, 408.0 [M-H, ^{10}B, ^{35}Cl, ^{35}Cl, ^{37}Cl, 31%]$^-$, 409.0 [M-H, ^{11}B, ^{35}Cl, ^{35}Cl, ^{37}Cl, 100%]$^-$, 410.2 [M-H, ^{10}B, ^{35}Cl, ^{37}Cl, ^{37}Cl, 24%]$^-$, 411.2 [M-H, ^{11}B, ^{35}Cl, ^{37}Cl, ^{37}Cl, 21%]$^-$, 412.5 [M-H, ^{10}B, ^{37}Cl, ^{37}Cl, ^{37}Cl, 4%]$^-$, 413.2 [M-H, ^{11}B, ^{37}Cl, ^{37}Cl, ^{37}Cl, 3%]$^-$; MS(ES$^+$) (m/z): 330.1 [M-Ph, ^{10}B, ^{35}Cl, ^{35}Cl, ^{35}Cl, 12%]$^+$, 331.2 [M-Ph, ^{11}B, ^{35}Cl, ^{35}Cl, ^{35}Cl, 65%]$^+$, 332.2 [M-Ph, ^{10}B, ^{35}Cl, ^{35}Cl, ^{37}Cl, 8%]$^+$, 333.2 [M-Ph, ^{11}B, ^{35}Cl, ^{35}Cl, ^{37}Cl, 63%]$^+$, 334.2 [M-Ph, ^{10}B, ^{35}Cl, ^{37}Cl, ^{37}Cl, 16%]$^+$, 335.3 [M-Ph, ^{11}B, ^{35}Cl, ^{37}Cl, ^{37}Cl, 33%]$^+$, 336.3 [M-Ph, ^{10}B, ^{37}Cl, ^{37}Cl, ^{37}Cl, 5%]$^+$, 337.3 [M-Ph, ^{11}B, ^{37}Cl, ^{37}Cl, ^{37}Cl, 3%]$^+$, 408.4 [M+H, ^{10}B, ^{35}Cl, ^{35}Cl, ^{35}Cl, 11%]$^+$, 409.2 [M+H, ^{11}B, ^{35}Cl, ^{35}Cl, ^{35}Cl, 73%]$^+$, 410.2 [M+H, ^{10}B, ^{35}Cl, ^{35}Cl, ^{37}C, 16%]$^-$, 411.2 [M+H, ^{11}B, ^{35}Cl, ^{35}Cl, ^{37}Cl, 84%]$^+$, 412.2 [M+H, ^{10}B, ^{35}Cl, ^{37}Cl, ^{37}Cl, 8%]$^+$, 413.2 [M+H, ^{11}B, ^{35}Cl, ^{37}Cl, ^{37}Cl, 17%]$^+$, 414.2 [M+H,

^{10}B, ^{37}Cl, ^{37}Cl, ^{37}Cl, 4%]$^+$, 415.2 [M+H, ^{11}B, ^{37}Cl, ^{37}Cl, ^{37}Cl, 3%]$^+$, 430.2 [M+Na, ^{10}B, ^{35}Cl, ^{35}Cl, ^{35}Cl, 4%]$^+$, 431.2 [M+Na, ^{11}B, ^{35}Cl, ^{35}Cl, ^{35}Cl, 46%]$^+$, 432.1 [M+Na, ^{10}B, ^{35}Cl, ^{35}Cl, ^{37}Cl, 13%]$^+$, 433.2 [M+Na, ^{11}B, ^{35}Cl, ^{35}Cl, ^{37}Cl, 17%]$^+$, 434.2 [M+Na, ^{10}B, ^{35}Cl, ^{37}Cl, ^{37}Cl, 9%]$^+$, 435.2 [M+Na, ^{11}B, ^{35}Cl, ^{37}Cl, ^{37}Cl, 10%]$^+$, 436.2 [M+Na, ^{10}B, ^{37}Cl, ^{37}Cl, ^{37}Cl, 3%]$^+$, 437.2 [M+Na, ^{11}B, ^{37}Cl, ^{37}Cl, ^{37}Cl, 5%]$^+$, 447.2 [M+K, ^{11}B, ^{35}Cl, ^{35}Cl, ^{35}Cl, 18%]$^+$, 449.5 [M+K, ^{11}B, ^{35}Cl, ^{35}Cl, ^{37}Cl, 8%]$^+$; HRMS(ES$^-$) (*m*/*z*): [M-H]$^-$ calcd. for $C_{18}H_{15}{}^{11}B^{35}Cl_3N_2O_2$, 407.0298; found, 407.0288, error: 2.5 ppm. Crystals suitable for X-ray diffraction were grown by slow evaporation of 1:1 *n*-hexane:toluene of NBC6 at room temperature.

5-(Dimethylcarbamoyl)-6-methyl-2,2-diphenyl-4-(trichloromethyl)-2,3-dihydro-1,3,2-oxazaborinin-1-ium-2-uide (NBC11, 16). Ethyl acetate/*n*-hexane, 1:2. Yield: 54%, yellow solid. mp: 112-113 ^oC; ^1H NMR (300 MHz, $CDCl_3$): δ 7.92 (br s, 1H, $Cl_3C(NH)C=C$), 7.17-7.44 (m, 10H, Aryl-H), 2.91 (s, 3H, CH_3), 2.32 (s, 3H, CH_3), 2.25 (s, 3H, CH_3); ^{13}C NMR (75 MHz, $CDCl_3$): δ 184.6 ($CH_3\underline{C}O$), 164.9 ($Cl_3C(NH)\underline{C}=C$), 160.7 ($\underline{C}ON(CH_3)_2$), 131.5 (B-Ph(*o*)), 130.0 (B-Ph(*o*)), 126.7 (B-Ph(*m*)), 126.3 (B-Ph(*p*)), 126.3 (B-Ph(*m*)), 125.7 (B-Ph(*p*)), 103.8 ($Cl_3C(NH)C=\underline{C}$), 91.5 (CCl_3), 36.8 ($CON(\underline{C}H_3)_2$), 34.3 ($CON(\underline{C}H_3)_2$), 21.8 ($\underline{C}H_3CO$), B-Ph(*i*) quaternary signal not observed; $^{11}B\{^1H\}$ (128 MHz, $CDCl_3$): δ 3.56; IR: 3301 (N-H), 1626 (C=O), 1585 (C=C, conjugated), 1460 (C=C-NH) cm^{-1}; MS(ES$^-$) (*m*/*z*): 434.1 [M-H, ^{10}B, ^{35}Cl, ^{35}Cl, ^{35}Cl, 24%]$^-$, 435.0 [M-H, ^{11}B, ^{35}Cl, ^{35}Cl, ^{35}Cl, 51%]$^-$, 436.0 [M-H, ^{10}B, ^{35}Cl, ^{35}Cl, ^{37}Cl, 21%]$^-$, 437.1 [M-H, ^{11}B, ^{35}Cl, ^{35}Cl, ^{37}Cl, 100%]$^-$, 438.0 [M-H, ^{10}B, ^{35}Cl, ^{37}Cl, ^{37}Cl, 11%]$^-$, 439.0 [M-H, ^{11}B, ^{35}Cl, ^{37}Cl, ^{37}Cl, 22%]$^-$, 440.0 [M-H, ^{10}B, ^{37}Cl, ^{37}Cl, ^{37}Cl, 6%]$^-$, 441.0 [M-H, ^{11}B, ^{37}Cl, ^{37}Cl, ^{37}Cl, 3%]$^-$; MS(ES$^+$) (*m*/*z*): 358.0 [M-Ph, ^{10}B, ^{35}Cl, ^{35}Cl, ^{35}Cl, 3%]$^+$, 359.0 [M-Ph, ^{11}B, ^{35}Cl, ^{35}Cl, ^{35}Cl, 10%]$^+$, 362.0 [M-Ph, ^{10}B, ^{35}Cl, ^{35}Cl, ^{37}Cl, 4%]$^+$, 361.0 [M-Ph, ^{11}B, ^{35}Cl, ^{35}Cl, ^{37}Cl, 13%]$^+$, 362.0 [M-Ph, ^{10}B, ^{35}Cl, ^{37}Cl, ^{37}Cl,

5%]$^+$, 363.0 [M-Ph, ^{11}B, ^{35}Cl, ^{37}Cl, ^{37}Cl, 6%]$^+$, 436.1 [M+H, ^{10}B, ^{35}Cl, ^{35}Cl, ^{35}Cl, 10%]$^+$, 437.1 [M+H, ^{11}B, ^{35}Cl, ^{35}Cl, ^{35}Cl, 70%]$^+$, 438.0 [M+H, ^{10}B, ^{35}Cl, ^{35}Cl, ^{37}Cl, 27%]$^+$, 439.0 [M+H, ^{11}B, ^{35}Cl, ^{35}Cl, ^{37}Cl, 45%]$^+$, 440.1 [M+H, ^{10}B, ^{35}Cl, ^{37}Cl, ^{37}Cl, 19%]$^+$, 441.1 [M+H, ^{11}B, ^{35}Cl, ^{37}Cl, ^{37}Cl, 26%]$^+$, 442.1 [M+H, ^{10}B, ^{37}Cl, ^{37}Cl, ^{37}Cl, 5%]$^+$, 443.1 [M+H, ^{11}B, ^{37}Cl, ^{37}Cl, ^{37}Cl, 2%]$^+$; HRMS(ES$^-$) (*m*/*z*): [M-H]$^-$ calcd. for $C_{20}H_{19}{}^{11}B^{35}Cl_3N_2O_2$, 435.0611; found, 435.0616, error: 1.2 ppm. Crystals suitable for X-ray diffraction were grown by slow evaporation of 1:1 *n*-hexane:toluene of NBC11 at room temperature.

6-Amino-5-cyano-2,2-diphenyl-4-(trichloromethyl)-2,3-dihydro-1,3,2-oxazaborinin-1-ium-2-uide (NBC18). Ethyl acetate/*n*-hexane, 1:9. Yield: 74%, grey solid. mp: 153-155 ^{o}C; ^{1}H NMR (300 MHz, $CDCl_3$): δ 7.66 (br s, 1H, $Cl_3C(NH)C{=}C$), 7.16-7.30 (m, 10H, Aryl-H), 6.48 (br s, 1H, $CONH_2$), 5.98 (br s, 1H, $CONH_2$); ^{13}C NMR (75 MHz, $CDCl_3$): δ 170.5 ($Cl_3C(NH)\underline{C}{=}C$), 163.9 (CONH), 130.6 (B-Ph(*o*)), 126.7 (B-Ph(*m*)), 126.3 (B-Ph(*p*)), 114.4 (CN), 90.8 (CCl_3), 63.3 ($Cl_3C(NH)C{=}\underline{C}$), B-Ph(*i*) quaternary signal not observed; ^{11}B{^{1}H} (128 MHz, $CDCl_3$): δ 4.59; IR: 3385 (N-H), 3353 (N-H), 2227 (C≡N), 1649 (C=O), 1596 (C=C, conjugated), 1524 (C=C-NH) cm^{-1}; MS(ES$^-$) (*m/z*): 389.1 [M-H, ^{10}B, ^{35}Cl, ^{35}Cl, ^{35}Cl, 19%]$^-$, 390.1 [M-H, ^{11}B, ^{35}Cl, ^{35}Cl, ^{35}Cl, 100%]$^-$, 391.1 [M-H, ^{10}B, ^{35}Cl, ^{35}Cl, ^{37}Cl, 22%]$^-$, 392.1 [M-H, ^{11}B, ^{35}Cl, ^{35}Cl, ^{37}Cl, 63%]$^-$, 393.1 [M-H, ^{10}B, ^{35}Cl, ^{37}Cl, ^{37}Cl, 21%]$^-$, 394.1 [M-H, ^{11}B, ^{35}Cl, ^{37}Cl, ^{37}Cl, 27%]$^-$, 395.1 [M-H, ^{10}B, ^{37}Cl, ^{37}Cl, ^{37}Cl, 2%]$^-$, 396.2 [M-H, ^{11}B, ^{37}Cl, ^{37}Cl, ^{37}Cl, 3%]$^-$; MS(ES$^+$) (*m*/*z*): 413.0 [M+Na, ^{10}B, ^{35}Cl, ^{35}Cl, ^{35}Cl, 14%]$^+$, 414.0 [M+Na, ^{11}B, ^{35}Cl, ^{35}Cl, ^{35}Cl, 67%]$^+$, 415.0 [M+Na, ^{10}B, ^{35}Cl, ^{35}Cl, ^{37}Cl, 24%]$^+$, 416.0 [M+Na, ^{11}B, ^{35}Cl, ^{35}Cl, ^{37}Cl, 100%]$^+$, 417.0 [M+Na, ^{10}B, ^{35}Cl, ^{37}Cl, ^{37}Cl, 11%]$^+$, 418.1 [M+Na, ^{11}B, ^{35}Cl, ^{37}Cl, ^{37}Cl, 18%]$^+$, 419.1 [M+Na, ^{10}B, ^{37}Cl, ^{37}Cl, ^{37}Cl, 4%]$^+$, 420.1 [M+Na, ^{11}B, ^{37}Cl,

^{37}Cl, ^{37}Cl, 3%]$^+$. HRMS(APCI$^+$) (*m*/*z*): [M-Ph]$^+$ calcd. for $C_{11}H_8{}^{11}B^{35}Cl_3N_3O$, 313.9821; found, 313.9823, error: 0.6 ppm.

5-Acetyl-6-methyl-2,2,4-triphenyl-2,3-dihydro-1,3,2-oxazaborinin-1-ium-2-uide (NBC23). Crude enaminone product was directly borylated using DPBA due to difficulty in purification. Ethyl acetate/*n*-hexane, 1:9. Yield: 17%, white solid. mp: 160-163 ^{o}C; ^{1}H NMR (300 MHz, $CDCl_3$): δ 7.41-7.59 (m, 5H, Aryl-H), 7.36 (d, *J* = 6.6 Hz, 4H, B-Ph(*o*)), 7.13-7.27 (m, 6H, B-Ph(*m*/*p*)), 7.04 (br s, 1H, C=C(NH)Ph), 2.50 (s, 3H, CH_3CO), 1.50 (s, 3H, CH_3CO); ^{13}C NMR (75 MHz, $CDCl_3$): δ 196.6 ($CH_3\underline{C}O$), 186.0 ($CH_3\underline{C}O$), 169.0 (C=$\underline{C}$(NH)Ph), 135.4 (Ph(*i*)), 131.8 (Ph(*p*)), 130.8 (B-Ph(*o*)), 128.7 (Ph(*m*)), 126.8 (Ph(*o*)), 126.4 (B-Ph(*m*)), 125.6 (B-Ph(*p*)), 113.9 ($\underline{C}$=C(NH)Ph), 31.3 ($\underline{C}H_3CO$), 23.5 ($\underline{C}H_3CO$); B-Ph(*i*) quaternary signal not observed; ^{11}B{^{1}H} (128 MHz, $CDCl_3$): δ 2.59; IR: 3208 (N-H), 1638 (C=O), 1628 (C=O) cm^{-1}; MS(ES$^-$) (*m/z*): 366.0 [M-H, ^{11}B, 100%]$^-$; MS(ES$^+$) (*m*/*z*): 368.0 [M+H, ^{11}B, 100%]$^+$, 390.0 [M+Na, ^{11}B, 70%]$^+$; HRMS(ES$^+$) (*m*/*z*): [M+Na]$^+$ calcd. for $C_{24}H_{22}{}^{11}BNO_2Na$, 390.1636; found, 390.1634, error: 0.5 ppm.

6-Amino-5-carbamoyl-2,2-diphenyl-4-(trichloromethyl)-2,3-dihydro-1,3,2-oxazaborinin-1-ium-2-uide (NBC24). Crude enaminone product was directly borylated using DPBA due to difficulty in purification. Ethyl acetate/*n*-hexane, 1:9. Yield: 13%, yellow solid. mp: 155-156 ^{o}C; ^{1}H NMR (300 MHz, DMSO-d_6): δ 8.80 (br s, 1H, $CONH_2$), 8.21 (br s, 1H, $CONH_2$), 7.33 (d, *J* = 6.6 Hz, 4H, B-Ph(*o*)), 7.05-7.24 (m, 6H, B-Ph(*m*/*p*)), $CONH_2$ signal not observed; ^{13}C NMR (75 MHz, DMSO-d_6): δ 169.0 ($CONH_2$), 167.5 (Cl_3C(NH)$\underline{C}$=C), 160.2 ($CONH_2$), 131.6 (B-Ph(*o*)), 126.7 (B-Ph(*m*)), 125.7 (B-Ph(*p*)), 93.5 (CCl_3), 89.6 (Cl_3C(NH)C=$\underline{C}$), B-Ph(*i*) quaternary signal not observed; IR: 3484 (N-H), 3419 (N-H), 3345 (N-H), 3318 (N-H), 1653 (C=O), 1614 (C=C, conjugated), 1559 (C=C-NH) cm^{-1}; MS(ES$^-$) (*m*/*z*): 407.0 [M-H, ^{10}B, ^{35}Cl,

^{35}Cl, ^{35}Cl, 12%]$^{-}$, 408.0 [M-H, ^{11}B, ^{35}Cl, ^{35}Cl, ^{35}Cl, 100%]$^{-}$, 409.0 [M-H, ^{10}B, ^{35}Cl, ^{35}Cl, ^{37}Cl, 19%]$^{-}$, 410.0 [M-H, ^{11}B, ^{35}Cl, ^{35}Cl, ^{37}Cl, 68%]$^{-}$, 411.0 [M-H, ^{10}B, ^{35}Cl, ^{37}Cl, ^{37}Cl, 5%]$^{-}$, 412.0 [M-H, ^{11}B, ^{35}Cl, ^{37}Cl, ^{37}Cl, 21%]$^{-}$, 413.0 [M-H, ^{10}B, ^{37}Cl, ^{37}Cl, ^{37}Cl, 3%]$^{-}$, 414.0 [M-H, ^{11}B, ^{37}Cl, ^{37}Cl, ^{37}Cl, 3%]$^{-}$; MS(ES^{+}) (*m*/*z*): 330.9 [M-Ph, ^{10}B, ^{35}Cl, ^{35}Cl, ^{35}Cl, 6%]$^{+}$, 331.9 [M-Ph, ^{11}B, ^{35}Cl, ^{35}Cl, ^{35}Cl, 62%]$^{+}$, 332.9 [M-Ph, ^{10}B, ^{35}Cl, ^{35}Cl, ^{37}Cl, 10%]$^{+}$, 333.9 [M-Ph, ^{11}B, ^{35}Cl, ^{35}Cl, ^{37}Cl, 34%]$^{+}$, 334.9 [M-Ph, ^{10}B, ^{35}Cl, ^{37}Cl, ^{37}Cl, 3%]$^{+}$, 335.9 [M-Ph, ^{11}B, ^{35}Cl, ^{37}Cl, ^{37}Cl, 8%]$^{+}$, 337.0 [M-Ph, ^{10}B, ^{37}Cl, ^{37}Cl, ^{37}, 3%]$^{+}$], 338.1 [M-Ph, ^{11}B, ^{37}Cl, ^{37}Cl, ^{37}Cl, 4%]$^{+}$, 409.0 [M+H, ^{10}B, ^{35}Cl, ^{35}Cl, ^{35}Cl, 5%]$^{+}$, 410.0 [M+H, ^{11}B, ^{35}Cl, ^{35}Cl, ^{35}Cl, 11%]$^{+}$, 411.0 [M+H, ^{10}B, ^{35}Cl, ^{35}Cl, ^{37}Cl, 7%]$^{+}$, 412.0 [M+H, ^{11}B, ^{35}Cl, ^{35}Cl, ^{37}Cl, 12%]$^{+}$, 413.0 [M+H, ^{10}B, ^{35}Cl, ^{37}Cl, ^{37}Cl, 3%]$^{+}$, 414.1 [M+H, ^{11}B, ^{35}Cl, ^{37}Cl, ^{37}Cl, 4%]$^{+}$, 415.1 [M+H, ^{10}B, ^{37}Cl, ^{37}Cl, ^{37}Cl, 2%]$^{+}$, 416.1 [M+H, ^{11}B, ^{37}Cl, ^{37}Cl, ^{37}Cl, 1%]$^{+}$, 431.0 [M+Na, ^{10}B, ^{35}Cl, ^{35}Cl, ^{35}Cl, 3%]$^{+}$, 432.0 [M+Na, ^{11}B, ^{35}Cl, ^{35}Cl, ^{35}Cl, 40%]$^{+}$, 433.0 [M+Na, ^{10}B, ^{35}Cl, ^{35}Cl, ^{37}Cl, 8%]$^{+}$, 434.0 [M+Na, ^{11}B, ^{35}Cl, ^{35}Cl, ^{37}Cl 18%]$^{+}$, 435.0 [M+Na, ^{10}B, ^{35}Cl, ^{37}Cl, ^{37}Cl, 5%]$^{+}$, 436.0 [M+Na, ^{11}B, ^{35}Cl, ^{37}Cl, ^{37}Cl, 6%]$^{+}$, 437.0 [M+Na, ^{10}B, ^{37}Cl, ^{37}Cl, ^{37}Cl, 2%]$^{+}$, 438.0 [M+Na, ^{11}B, ^{37}Cl, ^{37}Cl, ^{37}Cl, 1%]$^{+}$; HRMS(APCI^{+}) (*m*/*z*): [M+H]$^{+}$ calcd. for $C_{17}H_{16}{}^{11}B^{35}Cl_3N_3O_2$, 410.0396; found, 410.0396, error: 0.1 ppm.

5-(*tert*-Butoxycarbonyl)-2,2-diphenyl-6-methyl-4-(trichloromethyl)-2,3-dihydro-1,3,2-oxazaborinin-1-ium-2-uide (NBC25). Ethyl acetate/*n*-hexane, 1:9. Yield: 35%, yellow solid. mp: 147-149 ^{o}C; ^{1}H NMR (300 MHz, CDCl$_3$): δ 7.86 (br s, 1H, C=C(NH)CCl$_3$), 7.15-7.32 (m, 10H, 2 x Aryl-H), 2.34 (s, 3H, CH$_3$CO), 1.42 (s, 9H, (CH$_3$)$_3$C); ^{13}C NMR (75 MHz, CDCl$_3$): δ 187.2 (CH$_3\underline{C}$O), 164.4 (C=$\underline{C}$(NH)CCl$_3$), 163.3 (COO), 131.7 (B-Ph(*o*)), 127.5 (B-Ph(*m*)), 126.9 (B-Ph(*p*)), 103.8 ($\underline{C}$=C(NH)CCl$_3$), 93.3 (CCl$_3$), 82.8 ((CH$_3$)$_3\underline{C}$), 27.8 (($\underline{C}$H$_3$)$_3$C), 24.3 ($\underline{C}$H$_3$CO); B-Ph(*i*) quaternary signal not observed; ^{11}B{^{1}H} (128 MHz, CDCl$_3$): δ 3.96; IR: 3310 (N-H), 1715 (C=O), 1597

(C=C, conjugated) cm^{-1}; MS(ES$^-$) (*m/z*): 463.1 [M-H, ^{10}B, ^{35}Cl, ^{35}Cl, ^{35}Cl, 8%]$^-$, 464.1 [M-H, ^{11}B, ^{35}Cl, ^{35}Cl, ^{35}Cl, 64%]$^-$, 465.1 [M-H, ^{10}B, ^{35}Cl, ^{35}Cl, ^{37}Cl, 32%]$^-$, 466.1 [M-H, ^{11}B, ^{35}Cl, ^{35}Cl, ^{37}Cl, 100%]$^-$, 467.1 [M-H, ^{10}B, ^{35}Cl, ^{37}Cl, ^{37}Cl, 32%]$^-$, 468.1 [M-H, ^{11}B, ^{35}Cl, ^{37}Cl, ^{37}Cl, 20%]$^-$, 469.1 [M-H, ^{10}B, ^{37}Cl, ^{37}Cl, ^{37}Cl, 5%]$^-$, 470.1 [M-H, ^{11}B, ^{37}Cl, ^{37}Cl, ^{37}C, 3%]$^-$; HRMS(ES$^+$) (*m/z*): [M+Na]$^+$ calcd. for $C_{22}H_{23}{}^{11}B^{35}Cl_3NO_3Na$, 488.0729; found, 488.0727, error: 0.4 ppm.

5-(*tert*-Butoxycarbonyl)-4-(ethylthio)-2,2-diphenyl-6-methyl-2,3-dihydro-1,3,2-oxazaborinin-1-ium-2-uide (NBC26). Crude enaminone product was directly borylated using DPBA due to difficulty in purification. Ethyl acetate/*n*-hexane, 1:9. Yield: 30%, white solid. mp: 145-148 ^{o}C; ^{1}H NMR (300 MHz, $CDCl_3$): δ 7.11-7.30 (m, 10H, Aryl-H), 6.73 (br s, 1H, C=C(N*H̲*)SCH_2CH_3), 2.74 (q, *J* = 7.4 Hz, 2H, $CH_3C\underline{H}_2S$), 2.40 (s, 3H, CH_3CO), 1.44 (s, 9H, $(CH_3)_3C$), 1.33 (t, *J* = 7.5 Hz, 3H, $C\underline{H}_3CH_2S$); ^{13}C NMR (100 MHz, $CDCl_3$): δ 184.2 ($CH_3\underline{C}O$), 173.5 (COO), 165.6 (C=$\underline{C}$(NH)SCH_2CH_3), 131.9 (B-Ph(*o*)), 127.5 (B-Ph(*m*)), 126.7 (B-Ph(*p*)), 104.4 ($\underline{C}$=C(NH)CCl_3), 82.2 ($(CH_3)_3\underline{C}$), 28.5 ($(\underline{C}H_3)_3C$), 26.2 ($CH_3\underline{C}H_2S$), 24.4 ($\underline{C}H_3CO$), 12.0 ($\underline{C}H_3CH_2S$); B-Ph(*i*) quaternary signal not observed; ^{11}B{^{1}H} (128 MHz, $CDCl_3$): δ 3.00; IR: 3364 (N-H), 1660 (C=O), 1556 (C=C, conjugated), 1552 (C=C-NH) cm^{-1}; MS(ES$^-$) (*m/z*): 408.2 [M-H, ^{11}B, 100%]$^-$; HRMS(ES$^-$) (*m/z*): [M+Na]$^+$ calcd. for $C_{23}H_{28}{}^{11}BNO_3SNa$, 432.1775; found, 432.1774, error: 0.2 ppm.

4-(*tert*-Butyl)-6-methyl-2,2-diphenyl-2,3-dihydro-1,3,2-oxazaborinin-1-ium-2-uide (NBC28). Crude enaminone product was directly borylated using DPBA due to difficulty in purification. Ethyl acetate/*n*-hexane, 2:23. Yield: 3%, white solid. mp: 121-122 ^{o}C; ^{1}H NMR (300 MHz, $CDCl_3$): δ 7.29 (d, *J* = 6.6 Hz, 4H, B-Ph(*o*)), 7.08-7.23 (m, 6H, B-Ph(*m/p*)), 6.78 (br s, 1H, C=C(N*H̲*)$C(CH_3)_3$), 5.23 (d, *J* = 2.1 Hz, 1H, *H̲*C=C(NH)CCl_3), 2.02 (s, 3H, CH_3CO), 1.16 (s, 9H, $(CH_3)_3C$); ^{13}C NMR (75 MHz,

$CDCl_3$): δ 179.9, 179.8, 131.8 (B-Ph(*o*)), 127.3 (B-Ph(*m*)), 126.3 (B-Ph(*p*)), 93.7 (<u>*C*</u>=C(NH)CCl_3), 37.1 ((CH_3)$_3$<u>*C*</u>), 27.7 ((<u>*C*</u>H_3)$_3$C), 24.1 (<u>*C*</u>H_3CO); B-Ph(*i*) quaternary signal not observed; $^{11}B\{^1H\}$ (128 MHz, $CDCl_3$): δ 2.68; IR: 3381 (N-H), 1610 (C=C, conjugated), 1519 (C=C-NH) cm^{-1}; MS(ES^-) (*m/z*): 303.1 [M-H, ^{10}B, 27%]$^-$, 304.1 [M-H, ^{11}B, 100%]$^-$; MS(ES^+) (*m/z*): 227.1 [M-Ph, ^{10}B, 52%]$^+$, 228.1 [M-Ph, ^{11}B, 100%]$^+$, 305.2 [M+H, ^{10}B, 19%]$^+$, 306.1 [M+H, ^{11}B, 77%]$^+$, 327.1 [M+Na, ^{10}B, 5%]$^+$, 328.1 [M+Na, ^{11}B, 24%]$^+$; HRMS(ES^+) (*m/z*): [M+Na]$^+$ calcd. for $C_{20}H_{24}{}^{11}BNONa$, 328.1843; found, 328.1841, error: 0.6 ppm.

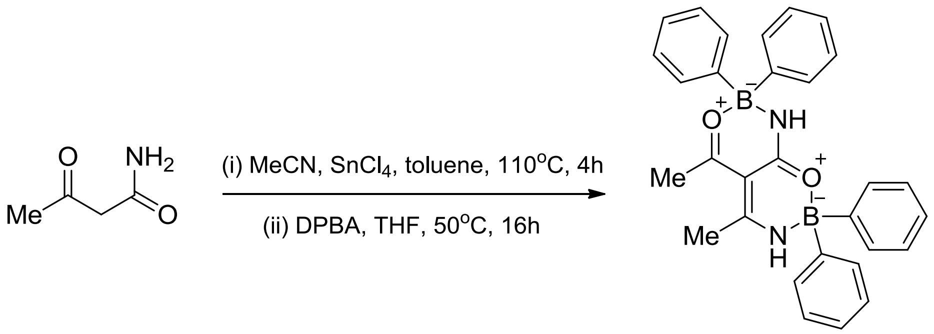

NBC8

Scheme 3 | Synthesis of NBC8.

4,5-Dimethyl-2,2,7,7-tetraphenyl-2,3,7,8-tetrahydro-[1,3,2]oxazaborinino[6,5-*d*][1,3,2]oxazaborinine-1,6,-diium-2,7-diuide (NBC8). Tin(IV) chloride (1.17 ml, 10 mmol) was slowly added to a stirring solution of acetoacetamide (1.01 g, 10 mmol) and acetonitrile (0.52 ml, 10 mmol) suspended in anhydrous toluene (10 ml). A white solid appeared and the reaction mixture was stirred at room temperature under N_2 for 30 min. The mixture was then heated at reflux at 110 ^oC under N_2 for 4 h. A yellow solid precipitated from the reaction mixture. The mixture was cooled to room temperature, washed with *n*-hexane (20 ml), decanted and then dried *in vacuo* overnight to give crude (*Z*)-3-acetyl-4-amino-3-penten-2-amide as a yellow oil. The crude product (0.42 g, 2.95 mmol) was added to DPBA (0.34 g, 0.98 mmol)

suspended in anhydrous THF (4 ml). The mixture was heated at 50 ^{o}C under N_2 for 16 h. The reaction mixture was extracted with DCM and extract dried over $MgSO_4$. The solution was concentrated and purified by flash column chromatography. Ethyl acetate/*n*-hexane, 1:9. Yield: 3%, cream solid. No other products were isolated. mp: 175-178 ^{o}C; ^{1}H NMR (400 MHz, $CDCl_3$): δ 7.12-7.29 (m, 20H, Aryl-H), 6.52 (br s, 1H, CH_3(N<u>*H*</u>)C=C), 2.43 (d, *J* = 0.8 Hz, 3H, C<u>*H*</u>$_3$(NH)C=C), 2.41 (s, 3H, CH_3CO), NH broad signal was not observed; ^{13}C NMR (100 MHz, $CDCl_3$): δ 188.2 (CH_3<u>*C*</u>O), 170.3 (CONH), 164.5 (CH_3(NH)<u>*C*</u>=C), 131.7 (B-Ph(*o*)), 131.6 (B-Ph(*o*)), 127.7 (B-(*m*)), 127.4 (B-Ph(*m*)), 126.9 (B-Ph(*p*)), 126.5 (B-Ph(*p*)), 27.5 (<u>*C*</u>H$_3$(NH)C=C), 27.1 (<u>*C*</u>H$_3$CO), B-Ph(*i*) quaternary signal not observed; IR: 3300 (N-H), 1619 (C=O), 1473 (C=C-NH) cm^{-1}; MS(ES$^-$) (*m/z*): 467.4 [M-H, ^{10}B, ^{10}B, ^{12}C, 5%]$^-$, 468.4 [M-H, ^{10}B, ^{11}B, ^{12}C, 30%]$^-$, 469.3 [M-H, ^{11}B, ^{11}B, ^{12}C, 100%]$^-$; HRMS(ES$^-$) (*m*/*z*): [M-H]$^-$ calcd. for $C_{30}H_{27}{}^{11}B_2N_2O_2$, 469.2264; found, 469.2271, error: 1.5 ppm.

1.4 General procedure for dioxaborine reaction

Either acetylacetone or acetoacetamide (3 eq) was added to a solution of DPBA (1 eq) in anhydrous THF (5 ml). The mixture was stirred at 50°C under N_2 for 16h. The mixture was concentrated *in vacuo* and purified by flash column chromatography, unless otherwise stated. The collected fractions were combined, evaporated *in vacuo* and stirred in cold *n*-hexane (15 ml) for 30 mins. The precipitate was then filtered and dried under vacuum to give the corresponding dioxaborine products.

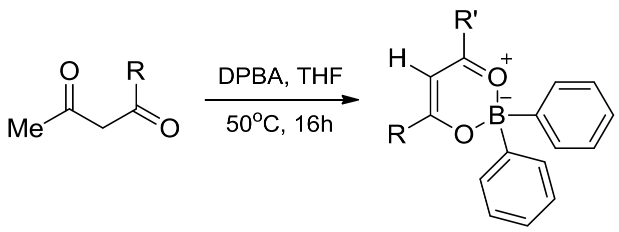

R	R'	
Me	NH_2	**NBC7**
CCl_3	NH_2	**NBC9**
Me	Me	**NBC10**

Scheme 4 | Synthesis of NBC7, NBC9 and NBC10.

6-Amino-4-methyl-2,2-diphenyl-2,3-dihydro-1,3,2-dioxaborinin-1-ium-2-uide (NBC7). Ethyl acetate/*n*-hexane, 1:4. Yield: 2%, white solid. mp: 164-166 °C; ^{1}H NMR (400 MHz, $CDCl_3$): δ 7.38 (d, *J* = 8.8 Hz, 4H, B-Ph(*o*)), 7.07-7.24 (m, 6H, B-Ph(*m*/*p*)), 5.42 (br s, 1H, $CONH_2$), 5.06 (br s, 1H, $CONH_2$), 4.88 (s, 1H, $CH_3C(O)C{=}CH$), 2.03 (s, 1H, CH_3CO); ^{13}C NMR (100 MHz, $CDCl_3$): δ 183.2 ($CONH_2$), 171.0 ($CH_3C(O)\underline{C}{=}C$), 131.6 (B-Ph(*o*)), 127.3 (B-Ph(*m*)), 126.5 (B-Ph(*p*)), 87.1 ($CH_3C(O)C{=}\underline{C}$), 23.9 ($\underline{C}H_3CO$), B-Ph(*i*) quaternary signal not observed; $^{11}B\{^{1}H\}$ (128 MHz, $CDCl_3$): δ 8.15; IR: 3429 (N-H), 3314 (N-H), 1637 (C=O), 1577 (C=C, conjugated), 1527 ($C{=}C{-}NH_2$) cm^{-1}; MS(ES^-) (*m/z*): 263.2 [M-H, ^{10}B, 18%]$^-$, 264.2 [M-H, ^{11}B, 100%]$^-$; MS(ES^+): (*m/z*) 187.3 [M-Ph, ^{10}B, 4%]$^+$, 188.3 [M-Ph, ^{11}B, 15%]$^+$, 265.3 [M+H, ^{10}B, 4%]$^+$, 266.3 [M+H, ^{11}B, 10%]$^+$, 288.2 [M+Na, ^{11}B, 4%]$^+$; HRMS(ES^-) (*m/z*): [M-H]$^-$ calcd. for $C_{16}H_{15}{}^{11}BNO_2$, 264.1201; found, 264.1206, error: 1.9 ppm.

6-Amino-2,2-diphenyl-4-(trichloromethyl)-2,3-dihydro-1,3,2-borinin-1-ium-2-uide (NBC9). A by-product obtained during the reaction to form **3g** from **2b**. Ethyl acetate/*n*-hexane, 3:7. Yield: 2%, cream solid. mp: 167-168 °C; ^{1}H NMR (300 MHz, DMSO-d_6): δ 9.69 (br s, 1H, $CONH_2$), 9.53 (br s, 1H, $CONH_2$), 7.39 (d, *J* = 6.3 Hz, 4H, B-Ph(*o*)), 7.07-7.25 (m, 6H, B-Ph(*m*/*p*)), 6.06 (s, 1H, $Cl_3C(O)C{=}CH$); ^{13}C NMR

(75 MHz, DMSO-d$_6$): δ 170.7 (CONH$_2$), 168.7 (Cl$_3$C(O)$\underline{C}$=C), 131.0 (B-Ph(*o*)), 126.8 (B-Ph(*m*)), 125.9 (B-Ph(*p*)), 86.3 (CCl$_3$), 64.4 (Cl$_3$C(O)C=$\underline{C}$), B-Ph(*i*) quaternary signal not observed; IR: 3433 (N-H), 3331 (N-H), 1644 (C=O), 1603 (C=C, conjugated), 1501 (C=C-NH$_2$) cm^{-1}; MS(ES$^-$) (*m/z*): 365.3 [M-H, ^{10}B, ^{35}Cl, ^{35}Cl, ^{35}Cl, 27%]$^-$, 366.1 [M-H, ^{11}B, ^{35}Cl, ^{35}Cl, ^{35}Cl, 91%]$^-$, 367.1 [M-H, ^{10}B, ^{35}Cl, ^{35}Cl, ^{37}Cl, 32%]$^-$, 368.1 [M-H, ^{11}B, ^{35}Cl, ^{35}Cl, ^{37}Cl, 100%]$^-$, 369.2 [M-H, ^{10}B, ^{35}Cl, ^{37}Cl, ^{37}Cl, 20%]$^-$, 370.4 [M-H, ^{11}B, ^{35}Cl, ^{37}Cl, ^{37}Cl, 19%]$^-$, 371.1 [M-H, ^{10}B, ^{37}Cl, ^{37}Cl, ^{37}Cl, 3%]$^-$, 372.0 [M-H, ^{11}B, ^{37}Cl, ^{37}Cl, ^{37}Cl, 2%]$^-$; MS(ES$^+$) (*m*/*z*): 290.0 [M-Ph, ^{11}B, ^{35}Cl, ^{35}Cl, ^{35}C]$^+$, 292.0 [M-Ph, ^{11}B, ^{35}Cl, ^{35}Cl, ^{37}Cl]$^+$, 294.0 [M-Ph, ^{11}B, ^{35}Cl, ^{37}Cl, ^{37}Cl]$^+$; HRMS(ES$^-$) (*m*/*z*): [M-H]$^-$ calcd. for $C_{16}H_{12}{}^{11}B^{35}Cl_3N_2O$, 366.0032; found, 366.0036, error: 1.1 ppm.

4,6-Dimethyl-2,2-diphenyl-2,3-dihydro-1,3,2-dioxaborinin-1-ium-2-uide (NBC10). The reaction mixture was extracted with DCM and the extract dried over MgSO$_4$. The solution was concentrated *in vacuo*. The residue was purified by recrystallisation from EtOH. Yield: 9%, yellow solid. mp: 108-110 ^{o}C; ^{1}H NMR (400 MHz, CDCl$_3$): δ 7.38 (dd, *J* = 7.8 Hz & 1.4 Hz, 4H, B-Ph(*o*)), 7.10-7.24 (m, 6H, B-Ph(*m*/*p*)), 5.60 (s, 1H, CH$_3$C(O)C=C$\underline{H}$), 2.15 (s, 6H, 2 x CH$_3$CO); ^{13}C NMR (100 MHz, CDCl$_3$) δ 191.1 (2 x CH$_3$$\underline{C}$O), 131.2 (B-Ph(*o*)), 127.3 (B-Ph(*m*)), 126.7 (B-Ph(*p*)), 102.2 (CH$_3$C(O)C=$\underline{C}$H), 24.5 (2 x $\underline{C}$H$_3$CO), B-Ph(*i*) quaternary signal and CH$_3$C(O)$\underline{C}$=CH not observed; IR: 1713 (C=O), 1511 (C=C-O) cm^{-1}; MS(ES$^-$) (*m/z*): 262.2 [M-H, ^{10}B, ^{12}C, 18%]$^-$, 263.1 [M-H, ^{11}B, ^{12}C, 41%]$^-$; MS(ES$^+$) (*m*/*z*): 186.1 [M-Ph, ^{10}B, ^{12}C, 29%]$^+$, 187.1 [M-Ph, ^{11}B, ^{12}C, 64%]$^+$, 265.2 [M+H, ^{11}B, 4%]$^+$; HRMS(ES$^-$) (*m*/*z*): [M-H]$^-$ calcd. for $C_{17}H_{16}{}^{11}BO_2$, 263.1249; found, 263.1239, error: 3.8 ppm.

General procedure for acetoacetamide-based oxazaborine derivatives

Unless otherwise stated, an appropriate primary amine (1 eq) was added to a solution of *tert*-butyl acetoacetate (1.1 eq) in toluene (150 ml) or DMF (5 ml). The

mixture was stirred at 125°C under N_2 for 16h. Solvent was evaporated *in vacuo* and the residue was dissolved in Et_2O/DCM (20 ml), washed with 1N $HCl_{(aq)}$ (2 x 10 ml), dried over $MgSO_4$ and evaporated *in vacuo*. All further reactions were conducted in the same manner as previously described for the general procedures for the enaminone reaction and oxazaborine reaction to give the corresponding oxazaborine products.

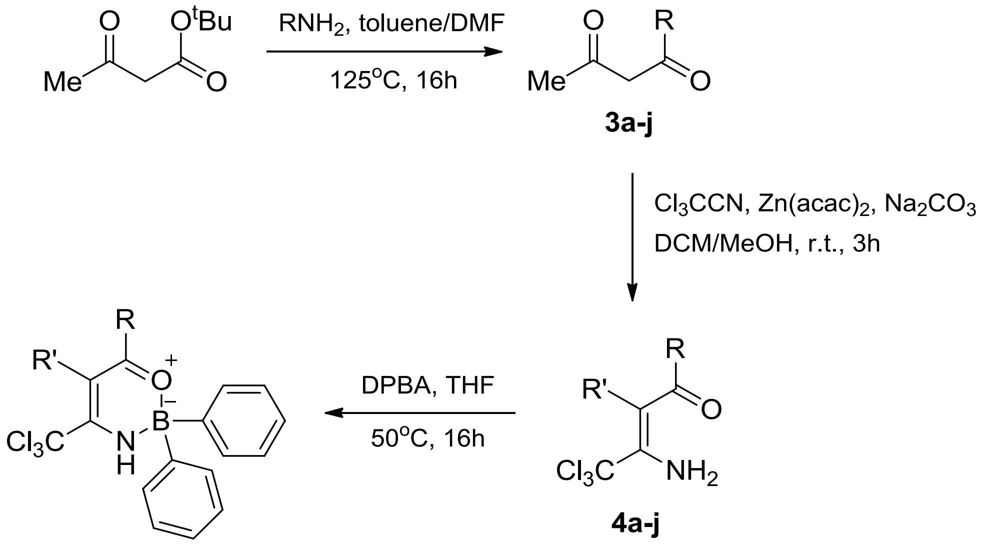

**R**	**R'**		**R**	**R'**	
NHMe	COMe	**NBC12**	NHAd	COMe	**NBC17**
NHPh	COMe	**NBC13**	NHCy	COMe	**NBC19**
$NHCH_2Ph$	COMe	**NBC14**	$NHCH_2Pyr$	COMe	**NBC20**
$NHCH_2CH_2Ph$	COMe	**NBC15**	NHPy	COMe	**NBC22**
$NHCH_2CH_2Ph$	H	**NBC16**	OEPPS	COMe	**NBC-EPPS**

Scheme 5 | Synthesis of NBC12-17, NBC19-20, NBC22 and NBC-EPPS.

***N*-Methylacetoacetamide (3a)**. Yield: 88%, orange oil. ^1H NMR (300 MHz, $CDCl_3$): δ 7.09 (br s, 1H, NH), 3.40 (s, 2H, $CH_3COC\underline{H}_2$), 2.80 (d, J = 4.5 Hz, 3H, $C\underline{H}_3NH$), 2.24 (s, 3H, CH_3CO); ^{13}C NMR (100 MHz, $CDCl_3$): δ 204.6 ($CH_3\underline{C}O$), 166.2 (CONH), 49.6 ($CH_3CO\underline{C}H_2$), 30.9 ($\underline{C}H_3CO$), 26.2 (CH_3NH); IR: 3305 (N-H), 1715 (C=O), 1641 (C=O), 1556 (C=O) cm^{-1}; MS(ES^+) (*m/z*): 116.0 $[M+H, 59\%]^+$, 138.0 $[M+Na, 81\%]^+$.

HRMS(APCI$^+$) (*m*/*z*): $[M+H]^+$ calcd. for $C_5H_{10}NO_2$, 116.0706; found, 116.0711, error: 4.3 ppm.

(*Z*)-2-Acetyl-3-amino-4,4,4-trichloro-*N*-methylbut-2-enamide (4a). Yield: 62%, white solid. mp: 112-113 ^oC; ^1H NMR (300 MHz, $CDCl_3$): δ 8.65 (br s, 2H, NH_2), 6.06 (br s, 1H, CONH), 2.85 (d, *J* = 4.8 Hz, $C\underline{H}_3NH$), 2.23 (s, 3H, CH_3CO); ^{13}C NMR (100 MHz, $CDCl_3$): δ 196.7 ($\underline{C}H_3CO$), 167.4 ($C{=}\underline{C}(NH_2)CCl_3$), 155.7 (CONH), 103.7 ($\underline{C}{=}C(NH_2)CCl_3$), 92.4 ($CCl_3$), 27.4 ($\underline{C}H_3CO$), 26.3 ($CH_3NH$); IR: 3276 (N-H), 1646 (C=O), 1637 (C=O), 1558 (C=O) cm^{-1}; MS(ES$^-$) (*m*/*z*): 257.0 [M-H, ^{35}Cl, ^{35}Cl, ^{35}Cl, 78%]$^-$, 258.9 [M-H, ^{35}Cl, ^{35}Cl, ^{37}Cl, 100%]$^-$, 261.0 [M-H, ^{35}Cl, ^{37}Cl, ^{37}Cl, 28%]$^-$, 263.0 [M-H, ^{37}Cl, ^{37}Cl, ^{37}Cl, 3%]$^-$, 292.9 [M+^{35}Cl, ^{35}Cl, ^{35}Cl, ^{35}Cl, 24%]$^-$, 295.0 [M+^{35}Cl, ^{35}Cl, ^{35}Cl, ^{37}Cl, 22%]$^-$, 297.0 [M+^{35}Cl, ^{35}Cl, ^{37}Cl, ^{37}Cl, 11%]$^-$, 209.0 [M+^{35}Cl, ^{37}Cl, ^{37}Cl, ^{37}Cl, 3%]$^-$; MS(ES$^+$) (*m*/*z*): 217.0 [M-C_2H_3O, 33%]$^+$, 259.0 [M+H, ^{35}Cl, ^{35}Cl, ^{35}Cl, 51%]$^+$, 261.0 [M+H, ^{35}Cl, ^{35}Cl, ^{37}Cl, 38%]$^+$, 263.0 [M+H, ^{35}Cl, ^{37}Cl, ^{37}Cl, 17%]$^+$, 265.0 [M+H, ^{37}Cl, ^{37}Cl, ^{37}Cl, 2%]$^+$, 281.0 [M+Na, ^{35}Cl, ^{35}Cl, ^{35}Cl, 70%]$^+$, 283.0 [M+Na, ^{35}Cl, ^{35}Cl, ^{37}Cl, 80%]$^+$, 285.0 [M+Na, ^{35}Cl, ^{37}Cl, ^{37}Cl, 34%]$^+$, 287.0 [M+Na, ^{37}Cl, ^{37}Cl, ^{37}Cl, 4%]$^+$.

5-Acetyl-6-(Methylamino)-2,2-diphenyl-4-(trichloromethyl)-2,3-dihydro-1,3,2-oxazaborinin-1-ium-2-uide (NBC12). Ethyl acetate/*n*-hexane, 3:7. Yield: 16%, yellow solid. mp: 157-158 ^oC (dec); ^1H NMR (300 MHz, $CDCl_3$): δ 7.83 (br s, 1H, $Cl_3C(NH)C{=}C$), 7.22-7.42 (m, 10H, Aryl-H), 5.52 (br s, 1H, CH_3NH), 2.82 (d, *J* = 4.8 Hz, 3H, CH_3NH), 2.37 (s, 3H, CH_3CO); ^{13}C NMR (75 MHz, $CDCl_3$): δ 187.5 ($CH_3\underline{C}O$), 165.5 ($Cl_3C(NH)\underline{C}{=}C$), 162.4 (CONH), 131.8 (B-Ph(*o*)), 127.7 (B-Ph(*m*)), 127.2 (B-Ph(*p*)), 105.6 ($Cl_3C(NH)C{=}\underline{C}$), 93.0 ($CCl_3$), 27.3 ($CH_3NH$), 23.5 ($\underline{C}H_3CO$), B-Ph(*i*) quaternary signal not observed; $^{11}B\{^1H\}$ (128 MHz, $CDCl_3$): δ 3.68; IR: 3313 (N-H), 1638 (C=O), 1593 (C=C, conjugated), 1524 (C=C-NH) cm^{-1}; MS(ES$^-$) (*m*/*z*): 420.0 [M-H, ^{10}B, ^{35}Cl, ^{35}Cl, ^{35}Cl, 23%]$^-$, 421.0 [M-H, ^{11}B, ^{35}Cl, ^{35}Cl, ^{35}Cl, 100%]$^-$, 422.0 [M-H,

^{10}B, ^{35}Cl, ^{35}Cl, ^{37}Cl, 38%]$^-$, 423.0 [M-H, ^{11}B, ^{35}Cl, ^{35}Cl, ^{37}Cl, 93%]$^-$, 424.0 [M-H, ^{10}B, ^{35}Cl, ^{37}Cl, ^{37}Cl, 25%]$^-$, 425.0 [M-H, ^{11}B, ^{35}Cl, ^{37}Cl, ^{37}Cl, 27%]$^-$, 426.0 [M-H, ^{10}B, ^{37}Cl, ^{37}Cl, ^{37}Cl, 4%]$^-$, 427.0 [M-H, ^{11}B, ^{37}Cl, ^{37}Cl, ^{37}Cl, 2%]$^-$; MS(ES$^+$) (*m*/*z*): 344.0 [M-Ph, ^{10}B, ^{35}Cl, ^{35}Cl, ^{35}Cl, 5%]$^+$, 345.0 [M-Ph, ^{11}B, ^{35}Cl, ^{35}Cl, ^{35}Cl, 43%]$^+$, 346.0 [M-Ph, ^{10}B, ^{35}Cl, ^{35}Cl, ^{37}Cl, 11%]$^+$, 347.0 [M-Ph, ^{11}B, ^{35}Cl, ^{35}Cl, ^{37}Cl, 40%]$^+$, 348.0 [M-Ph, ^{10}B, ^{35}Cl, ^{37}Cl, ^{37}Cl, 8%]$^+$, 349.0 [M-Ph, ^{11}B, ^{35}Cl, ^{37}Cl, ^{37}Cl, 7%]$^+$, 350.0 [M-Ph, ^{10}B, ^{37}Cl, ^{37}Cl, ^{37}Cl, 3%]$^+$, 351.0 [M-Ph, ^{11}B, ^{37}Cl, ^{37}Cl, ^{37}Cl, 6%]$^+$, 422.1 [M+H, ^{10}B, ^{35}Cl, ^{35}Cl, ^{35}Cl, 35%]$^+$, 423.1 [M+H, ^{11}B, ^{35}Cl, ^{35}Cl, ^{35}Cl, 77%]$^+$, 424.0 [M+H, ^{10}B, ^{35}Cl, ^{35}Cl, ^{37}Cl, 60%]$^+$, 425.0 [M+H, ^{11}B, ^{35}Cl, ^{35}Cl, ^{37}Cl, 100%]$^+$, 426.0 [M+H, ^{10}B, ^{35}Cl, ^{37}Cl, ^{35}Cl, 39%]$^+$, 427.0 [M+H, ^{11}B, ^{35}Cl, ^{37}Cl, ^{37}Cl, 48%]$^+$, 428.0 [M+H, ^{10}B, ^{37}Cl, ^{37}Cl, ^{37}Cl, 8%]$^+$, 429.0 [M+H, ^{11}B, ^{37}Cl, ^{37}Cl, ^{37}Cl, 7%]$^+$, 444.0 [M+Na, ^{10}B, ^{35}Cl, ^{35}Cl, ^{35}Cl, 20%]$^+$, 445.0 [M+Na, ^{11}B, ^{35}Cl, ^{35}Cl, ^{35}Cl, 53%]$^+$, 446.0 [M+Na, ^{10}B, ^{35}Cl, ^{35}Cl, ^{37}Cl, 12%]$^+$, 447.0 [M+Na, ^{11}B, ^{35}Cl, ^{35}Cl, ^{37}Cl, 49%]$^+$, 448.0 [M+Na, ^{10}B, ^{35}Cl, ^{37}Cl, ^{37}Cl, 12%]$^+$, 449.0 [M+Na, ^{11}B, ^{35}Cl, ^{37}Cl, ^{37}Cl, 14%]$^+$, 450.0 [M+Na, ^{10}B, ^{37}Cl, ^{37}Cl, ^{37}Cl, 2%]$^+$, 451.0 [M+Na, ^{11}B, ^{37}Cl, ^{37}Cl, ^{37}Cl, 3%]$^+$. HRMS(ES$^+$) (*m*/*z*): [M+Na]$^+$ calcd. for $C_{19}H_{18}{}^{11}B^{35}Cl_3N_2O_2Na$, 445.0419; found, 445.0420, error: 0.2 ppm.

***N*-Phenylacetoacetamide (3b)**. Yield: 56%, orange solid. mp: 81-83 ^oC (lit.(Sridharan et al., 2010) 85-86 ^oC); ^1H NMR (300 MHz, $CDCl_3$): δ 9.13 (br s, 1H, NH), 7.56 (d, *J* = 8.1 Hz, 2H, Ph(*o*)), 7.34 (t, *J* = 7.8 Hz, 2H, Ph(*m*)), 7.14 (t, *J* = 7.4 Hz, 1H, Ph(*p*)), 3.61 (s, 2H, $CH_3COC\underline{H_2}$), 2.34 (s, 3H, CH_3CO); ^{13}C NMR (75 MHz, $CDCl_3$): δ 205.2 ($CH_3\underline{C}O$), 163.3 (CONH), 137.5 (Ph(*i*)), 129.0 (Ph(*m*)), 124.6 (Ph(*p*)), 120.2 (Ph(*o*)), 49.7 ($CH_3CO\underline{C}H_2$), 31.3 ($\underline{C}H_3CO$); IR: 3253 (N-H), 1710 (C=O), 1659 (C=O), 1598 (C=O) cm^{-1}; MS(ES$^-$) (*m*/*z*): 204.0 [M-H, 100%]$^-$; MS(ES$^+$) (*m*/*z*): 164.1 [M-C_2H_3O, 47%]$^+$ 206.1 [M+H, 100%]$^+$, 228.0 [M+Na, 68%]$^+$, 244.0

[M+K, 62%]$^+$. HRMS(APCI$^+$) (*m/z*): [M+H]$^+$ calcd. for $C_{10}H_{12}NO_2$, 178.0863; found, 178.0855, error: 4.5 ppm.

(*Z*)-2-Acetyl-3-amino-4,4,4-trichloro-*N*-phenylbut-2-enamide (4b). Yield: 42%, cream solid. mp: 105-107 ^oC (lit.(Veronese et al., 1986) 139-143 ^oC); ^1H NMR (300 MHz, $CDCl_3$): δ 8.73 (br s, 2H, NH_2), 7.72 (br s, 1H, CONH), 7.50 (d, *J* = 8.1 Hz, 2H, Ph(*o*)), 7.30 (t, *J* = 7.8 Hz, 2H, Ph(*m*)), 7.10 (t, *J* = 7.4 Hz, 1H, Ph(*p*)), 2.32 (s, 3H, CH_3CO); ^{13}C NMR (75 MHz, $CDCl_3$): δ 197.6 ($CH_3\underline{C}O$), 165.5 (C=$\underline{C}$(NH_2)CCl_3), 157.0 (CONH), 137.8 (Ph(*i*)), 129.2 (Ph(*m*)), 125.0 (Ph(*p*)), 119.9 (Ph(*o*)), 105.4 ($\underline{C}$=C(NH_2)CCl_3), 93.4 (CCl_3), 28.6 ($\underline{C}H_3CO$); IR: 3267 (N-H), 1647 (C=O), 1617 (C=O), 1595 (C=C, conjugated), 1528 (C=C-NH_2) cm^{-1}; MS(ES$^-$) (*m/z*): 319.0 [M-H, ^{35}Cl, ^{35}Cl, ^{35}Cl, 100%]$^-$, 321.0 [M-H, ^{35}Cl, ^{35}Cl, ^{37}Cl, 65%]$^-$, 323.0 [M-H, ^{35}Cl, ^{37}Cl, ^{37}Cl, 30%]$^-$, 325.0 [M-H, ^{37}Cl, ^{37}Cl, ^{37}Cl, 3%]$^-$; MS(ES$^+$) (*m/z*): 227.9 [M-C_6H_6N, ^{35}Cl, ^{35}Cl, ^{35}Cl, 61%]$^+$, 229.9 [M-C_6H_6N, ^{35}Cl, ^{35}Cl, ^{37}Cl, 47%]$^+$, 231.9 [M-C_6H_6N, ^{35}Cl, ^{37}Cl, ^{37}Cl, 12%]$^+$, 233.0 [M-C_6H_6N, ^{37}Cl, ^{37}Cl, ^{37}Cl, 5%]$^+$, 279.1 [M-C_2H_3O, ^{35}Cl, ^{35}Cl, ^{35}Cl, 34%]$^+$, 281.0 [M-C_2H_3O, ^{35}Cl, ^{35}Cl, ^{37}Cl, 4%]$^+$, 283.0 [M-C_2H_3O, ^{35}Cl, ^{37}Cl, ^{37}Cl, 3%]$^+$, 285.0 [M-C_2H_3O, ^{37}Cl, ^{37}Cl, ^{37}Cl, 2%]$^+$, 321.0 [M+H, ^{35}Cl, ^{35}Cl, ^{35}Cl, 100%]$^+$, 323.0 [M+H, ^{35}Cl, ^{35}Cl, ^{37}Cl, 72%]$^+$, 325.0 [M+H, ^{35}Cl, ^{37}Cl, ^{37}Cl, 18%]$^+$, 327.0 [M+H, ^{37}Cl, ^{37}Cl, ^{37}Cl, 2%]$^+$, 343.0 [M+Na, ^{35}Cl, ^{35}Cl, ^{35}Cl, 24%]$^+$, 345.0 [M+Na, ^{35}Cl, ^{35}Cl, ^{37}Cl, 15%]$^+$, 347.0 [M+Na, ^{35}Cl, ^{37}Cl, ^{37}Cl, 4%]$^+$, 349.0 [M+Na, ^{37}Cl, ^{37}Cl, ^{37}Cl, 2%]$^+$.

5-Acetyl-6-(phenylamino)-2,2-diphenyl-4-(trichloromethyl)-2,3-dihydro-1,3,2-oxazaborinin-1-ium-2-uide (NBC13). Ethyl acetate/*n*-hexane, 1:7. Yield: 41%, yellow solid. mp: 131-133 ^oC; ^1H NMR (300 MHz, $CDCl_3$): δ 7.95 (br s, 1H, C=C(NH)CCl_3), 7.10-7.46 (m, 16H, 3 x Aryl-H and CONH), 2.44 (s, 3H, CH_3CO); ^{13}C NMR (75 MHz, $CDCl_3$): δ 187.6 ($CH_3\underline{C}O$), 162.5 (C=$\underline{C}$(NH)CCl_3), 162.4 (CONH), 137.4 (Ph(*i*)), 131.7 (B-Ph(*o*)), 129.2 (Ph(*m*)), 127.6 (B-Ph(*m*)), 127.2 (Ph(*p*)), 125.1

(B-Ph(*p*)), 119.7 (Ph(*o*)), 106.4 ($\underline{C}$=C(NH)CCl_3), 92.8 (CCl_3), 23.4 ($\underline{C}H_3CO$), B-Ph(*i*) quaternary signal not observed; $^{11}B\{^1H\}$ (128 MHz, $CDCl_3$): δ 3.77; IR: 3350 (N-H), 1654 (C=O), 1596 (C=C, conjugated), 1520 (C=C-NH) cm^{-1}; MS(ES^-) (*m/z*): 482.2 [M-H, ^{10}B, ^{35}Cl, ^{35}Cl, ^{35}Cl, 13%]$^-$, 483.2 [M-H, ^{11}B, ^{35}Cl, ^{35}Cl, ^{35}Cl, 63%]$^-$, 484.2 [M-H, ^{10}B, ^{35}Cl, ^{35}Cl, ^{37}Cl, 29%]$^-$, 485.1 [M-H, ^{11}B, ^{35}Cl, ^{35}Cl, ^{37}Cl, 100%]$^-$, 486.2 [M-H, ^{10}B, ^{35}Cl, ^{37}Cl, ^{37}Cl, 15%]$^-$, 487.2 [M-H, ^{11}B, ^{35}Cl, ^{37}Cl, ^{37}Cl, 23%]$^-$, 488.1 [M-H, ^{10}B, ^{37}Cl, ^{37}Cl, ^{37}Cl, 3%]$^-$, 489.1 [M-H, ^{11}B, ^{37}Cl, ^{37}Cl, ^{37}C, 1%]$^-$; MS(ES^+) (*m/z*): 484.1 [M+H, ^{10}B, ^{35}Cl, ^{35}Cl, ^{35}Cl, 5%]$^+$, 485.1 [M+H, ^{11}B, ^{35}Cl, ^{35}Cl, ^{35}Cl, 37%]$^+$, 486.1 [M+H, ^{10}B, ^{35}Cl, ^{35}Cl, ^{37}Cl, 12%]$^+$, 487.1 [M+H, ^{11}B, ^{35}Cl, ^{35}Cl, ^{37}Cl, 28%]$^+$, 488.1 [M+H, ^{10}B, ^{35}Cl, ^{37}Cl, ^{35}Cl, 4%]$^+$, 489.1 [M+H, ^{11}B, ^{35}Cl, ^{37}Cl, ^{37}Cl, 11%]$^+$, 490.2 [M+H, ^{10}B, ^{37}Cl, ^{37}Cl, ^{37}Cl, 3%]$^+$, 491.2 [M+H, ^{11}B, ^{37}Cl, ^{37}Cl, ^{37}Cl, 1%]$^+$, 506.1 [M+Na, ^{10}B, ^{35}Cl, ^{35}Cl, ^{35}Cl, 38%]$^+$, 507.1 [M+Na, ^{11}B, ^{35}Cl, ^{35}Cl, ^{35}Cl, 100%]$^+$, 508.1 [M+Na, ^{10}B, ^{35}Cl, ^{35}Cl, ^{37}Cl, 43%]$^+$, 509.1 [M+Na, ^{11}B, ^{35}Cl, ^{35}Cl, ^{37}Cl, 78%]$^+$, 510.1 [M+Na, ^{10}B, ^{35}Cl, ^{37}Cl, ^{37}Cl, 19%]$^+$, 511.1 [M+Na, ^{11}B, ^{35}Cl, ^{37}Cl, ^{37}Cl, 26%]$^+$, 512.1 [M+Na, ^{10}B, ^{37}Cl, ^{37}Cl, ^{37}Cl, 3%]$^+$, 513.1 [M+Na, ^{11}B, ^{37}Cl, ^{37}Cl, ^{37}Cl, 2%]$^+$. HRMS(ES^+) (*m/z*): $[M+H]^+$ calcd. for $C_{24}H_{21}{}^{11}B^{35}Cl_3N_2O_2$, 485.0756; found, 485.0759, error: 0.6 ppm.

***N*-Benzylacetoacetamide (3c)**. Yield: 97%, cream solid. mp: 100-102 ^oC (lit.(Sridharan et al., 2010) 92-93 ^oC); ^1H NMR (300 MHz, $CDCl_3$): δ 7.24-7.40 (m, 5H, Aryl-H), 7.36 (br s, 1H, NH), 4.46 (d, *J* = 5.7 Hz, 2H, $NHC\underline{H}_2$) 3.45 (s, 2H, $CH_3COC\underline{H}_2$), 2.27 (s, 3H, CH_3CO); ^{13}C NMR (75 MHz, $CDCl_3$): δ 204.5 ($CH_3\underline{C}O$), 165.5 (CONH), 137.9 (Ph(*i*)), 128.7 (Ph(*m*)), 127.7 (Ph(*o*)), 127.5 (Ph(*p*)), 49.6 ($CH_3CO\underline{C}H_2$), 43.5 ($NH\underline{C}H_2Ph$), 31.0 ($\underline{C}H_3CO$); IR: 3245 (N-H), 1712 (C=O), 1638 (C=O), 1577 (C=O) cm^{-1}; MS(ES^-) (*m/z*): 190.1 [M-H, 5%]$^-$; 226.1 [M+^{35}Cl, 100%]$^-$, 228.1 [M+^{37}Cl, 33%]$^-$; MS(ES^+) (*m/z*): 192.1 [M+H, 100%]$^+$ 214.1 [M+Na, 63%]$^+$,

228.0 [M+Na, 68%]$^+$, 244.0 [M+K, 62%]$^+$. HRMS(APCI$^+$) (*m*/*z*): [M+H]$^+$ calcd. for $C_{11}H_{14}NO_2$, 192.1019; found, 192.1020, error: 0.5 ppm.

(*Z*)-2-Acetyl-3-amino-4,4,4-trichloro-*N*-benzylbut-2-enamide (4c). Yield: 75%, cream solid. mp: 92-93 ^oC (lit.(Veronese et al., 1986) 120-124 ^oC); ^1H NMR (300 MHz, $CDCl_3$): δ 8.61 (br s, 2H, NH_2), 7.22-7.31 (m, 5H, Aryl-H), 6.12 (br s, 1H, CONH) 4.45 (d, *J* = 5.4 Hz, 2H, NHC$\underline{H_2}$), 2.24 (s, 3H, CH_3CO); ^{13}C NMR (75 MHz, $CDCl_3$): δ 197.6 ($CH_3\underline{C}O$), 167.3 (C=$\underline{C}$(NH_2)CCl_3), 156.7 (CONH), 136.9 (Ph(*i*)), 128.8 (Ph(*m*)), 128.5 (Ph(*o*)), 127.9 (Ph(*p*)), 104.6 ($\underline{C}$=C(NH_2)CCl_3), 93.4 (CCl_3), 45.1 (NH$\underline{C}H_2$Ph), 28.5 ($\underline{C}H_3CO$); IR: 3297 (N-H), 1619 (C=O), 1541 (C=O), 1495 (C=C-NH_2) cm^{-1}; MS(ES$^-$) (*m*/*z*): 333.1 [M-H, ^{35}Cl, ^{35}Cl, ^{35}Cl, 43%]$^-$, 335.1 [M-H, ^{35}Cl, ^{35}Cl, ^{37}Cl, 38%]$^-$, 337.1 [M-H, ^{35}Cl, ^{37}Cl, ^{37}Cl, 11%]$^-$, 339.1 [M-H, ^{37}Cl, ^{37}Cl, ^{37}Cl, 2%]$^-$, 369.1 [M+^{35}Cl, ^{35}Cl, ^{35}Cl, ^{35}Cl, 89%]$^-$, 370.1 [M+^{37}Cl, ^{35}Cl, ^{35}Cl, ^{35}Cl, 5%]$^-$, 371.1 [M+^{35}Cl, ^{35}Cl, ^{35}Cl, ^{37}Cl, 100%]$^-$, 372.1 [M+^{37}Cl, ^{35}Cl, ^{35}Cl, ^{37}Cl, 15%]$^-$, 373.1 [M+^{35}Cl, ^{35}Cl, ^{37}Cl, ^{37}Cl, 47%]$^-$, 374.1 [M+^{37}Cl, ^{35}Cl, ^{37}Cl, ^{37}Cl, 3%]$^-$, 375.1 [M+^{35}Cl, ^{37}Cl, ^{37}Cl, ^{37}Cl, 6%]$^-$, 376.1 [M+^{37}Cl, ^{37}Cl, ^{37}Cl, ^{37}Cl, 1%]$^-$; MS(ES$^+$) (*m*/*z*): 335.1 [M+H, ^{35}Cl, ^{35}Cl, ^{35}Cl, 70%]$^+$, 337.1 [M+H, ^{35}Cl, ^{35}Cl, ^{37}Cl, 98%]$^+$, 339.1 [M+H, ^{35}Cl, ^{37}Cl, ^{37}Cl, 29%]$^+$, 341.1 [M+H, ^{37}Cl, ^{37}Cl, ^{37}Cl, 3%]$^+$, 357.1 [M+Na, ^{35}Cl, ^{35}Cl, ^{35}Cl, 30%]$^+$, 359.1 [M+Na, ^{35}Cl, ^{35}Cl, ^{37}Cl, 18%]$^+$, 361.1 [M+Na, ^{35}Cl, ^{37}Cl, ^{37}Cl, 4%]$^+$, 363.1 [M+Na, ^{37}Cl, ^{37}Cl, ^{37}Cl, 1%]$^+$. HRMS(APCI$^+$) (*m*/*z*): [M+Na]$^+$ calcd. for $C_{13}H_{13}{}^{35}Cl_3N_2O_2Na$, 356.9935; found, 356.9916, error: 5.3 ppm.

5-Acetyl-6-(benzylamino)-2,2-diphenyl-4-(trichloromethyl)-2,3-dihydro-1,3,2-oxazaborinin-1-ium-2-uide (NBC14). Ethyl acetate/*n*-hexane, 1:7. Yield: 12%, yellow solid. Exists as a mixture of rotamers in a 4:1 ratio. mp: 131-132 ^oC; ^1H NMR (300 MHz, $CDCl_3$): δ 7.85 (br s, 1H, C=C(NH)CCl_3), 7.00-7.45 (m, 15H, 3 x Aryl-H of major and minor rotamers), 5.87 (br s, 1H, CONH of minor rotamer), 5.86 (br s, 1H,

CONH of major rotamer), 4.77 (d, 2H, NHC*$\underline{H}_2$*Ph of minor rotamer), 4.43 (d, *J* = 5.7 Hz, 2H, NHC*$\underline{H}_2$*Ph of major rotamer), 2.36 (s, 3H, CH_3CO); ^{13}C NMR (75 MHz, $CDCl_3$): δ 187.2 ($CH_3\underline{C}O$), 164.5 (C=$\underline{C}$(NH)CCl_3), 162.2 (CONH), 136.7 (Ph(*i*)), 131.7 (B-Ph(*o*)), 128.8 (Ph(*m*)), 128.4 (Ph(*o*)), 127.9 (Ph(*p*)), 127.5 (B-Ph(*m*)), 127.0 (B-Ph(*p*)), 105.2 ($\underline{C}$=C(NH)CCl_3), 92.8 (CCl_3), 44.8 (NH$\underline{C}H_2$Ph), 23.4 ($\underline{C}H_3CO$), B-Ph(*i*) quaternary signal not observed; IR: 3303 (N-H), 1631 (C=O), 1596 (C=C, conjugated), 1526 (C=C-NH) cm^{-1}; MS(ES^-) (*m/z*): 455.1 [M-Ac, ^{10}B, ^{35}Cl, ^{35}Cl, ^{35}Cl, 9%]$^-$, 456.2 [M-Ac, ^{11}B, ^{35}Cl, ^{35}Cl, ^{35}Cl, 74%]$^-$, 457.2 [M-Ac, ^{10}B, ^{35}Cl, ^{35}Cl, ^{37}Cl, 40%]$^-$, 458.2 [M-Ac, ^{11}B, ^{35}Cl, ^{35}Cl, ^{37}Cl, 100%]$^-$, 459.2 [M-Ac, ^{10}B, ^{35}Cl, ^{37}Cl, ^{37}Cl, 17%]$^-$, 460.2 [M-Ac, ^{11}B, ^{35}Cl, ^{37}Cl, ^{37}Cl, 18%]$^-$, 461.1 [M-Ac, ^{10}B, ^{37}Cl, ^{37}Cl, ^{37}Cl, 4%]$^-$, 462.1 [M-Ac, ^{11}B, ^{37}Cl, ^{37}Cl, ^{37}C, 3%]$^-$, 496.2 [M-H, ^{10}B, ^{35}Cl, ^{35}Cl, ^{35}Cl, 50%]$^-$, 497.2 [M-H, ^{11}B, ^{35}Cl, ^{35}Cl, ^{35}Cl, 73%]$^-$, 498.2 [M-H, ^{10}B, ^{35}Cl, ^{35}Cl, ^{37}Cl, 60%]$^-$, 499.2 [M-H, ^{11}B, ^{35}Cl, ^{35}Cl, ^{37}Cl, 73%]$^-$, 500.2 [M-H, ^{10}B, ^{35}Cl, ^{37}Cl, ^{37}Cl, 37%]$^-$, 501.2 [M-H, ^{11}B, ^{35}Cl, ^{37}Cl, ^{37}Cl, 18%]$^-$, 502.2 [M-H, ^{10}B, ^{37}Cl, ^{37}Cl, ^{37}Cl, 3%]$^-$, 503.2 [M-H, ^{11}B, ^{37}Cl, ^{37}Cl, ^{37}C, 2%]$^-$; MS(ES^+) (*m/z*): 498.1 [M+H, ^{10}B, ^{35}Cl, ^{35}Cl, ^{35}Cl, 13%]$^+$, 499.2 [M+H, ^{11}B, ^{35}Cl, ^{35}Cl, ^{35}Cl, 80%]$^+$, 500.2 [M+H, ^{10}B, ^{35}Cl, ^{35}Cl, ^{37}Cl, 35%]$^+$, 501.2 [M+H, ^{11}B, ^{35}Cl, ^{35}Cl, ^{37}Cl, 100%]$^+$, 502.2 [M+H, ^{10}B, ^{35}Cl, ^{37}Cl, ^{35}Cl, 26%]$^+$, 503.1 [M+H, ^{11}B, ^{35}Cl, ^{37}Cl, ^{37}Cl, 32%]$^+$, 504.1 [M+H, ^{10}B, ^{37}Cl, ^{37}Cl, ^{37}Cl, 5%]$^+$, 505.1 [M+H, ^{11}B, ^{37}Cl, ^{37}Cl, ^{37}Cl, 3%]$^+$, 520.2 [M+Na, ^{10}B, ^{35}Cl, ^{35}Cl, ^{35}Cl, 18%]$^+$, 521.2 [M+Na, ^{11}B, ^{35}Cl, ^{35}Cl, ^{35}Cl, 24%]$^+$, 522.2 [M+Na, ^{10}B, ^{35}Cl, ^{35}Cl, ^{37}Cl, 11%]$^+$, 523.1 [M+Na, ^{11}B, ^{35}Cl, ^{35}Cl, ^{37}Cl, 19%]$^+$, 524.1 [M+Na, ^{10}B, ^{35}Cl, ^{37}Cl, ^{37}Cl, 6%]$^+$, 525.1 [M+Na, ^{11}B, ^{35}Cl, ^{37}Cl, ^{37}Cl, 4%]$^+$, 526.1 [M+Na, ^{10}B, ^{37}Cl, ^{37}Cl, ^{37}Cl, 2%]$^+$, 527.1 [M+Na, ^{11}B, ^{37}Cl, ^{37}Cl, ^{37}Cl, 1%]$^+$. HRMS($APCI^+$) (*m/z*): $[M+Na]^+$ calcd. for $C_{25}H_{22}{}^{11}B^{35}Cl_3N_2O_2Na$, 521.0732; found, 521.0720, error: 2.3 ppm.

***N*-Phenethylacetoacetamide (3d)**. A yellow solid formed on addition of 1N $HCl_{(aq)}$ and was discarded by filtration prior to Et_2O extraction. Yield: 71%, orange oil. ^1H NMR (300 MHz, $CDCl_3$): δ 7.08-7.27 (m, 5H, Aryl-H), 6.94 (br s, 1H, NH), 3.45 (q, *J* = 6.6 Hz, 2H, $NHC\underline{H_2}CH_2$), 3.27 (s, 2H, $CH_3COC\underline{H_2}$), 2.74 (t, *J* = 7.1 Hz, 2H, $NHCH_2C\underline{H_2}$), 2.14 (s, 3H, CH_3CO); ^{13}C NMR (75 MHz, $CDCl_3$): 204.4 ($CH_3\underline{C}O$), 165.4 (CONH), 138.7 (Ph(*i*)), 128.7 (Ph(*o*)), 128.6 (Ph(*m*)), 126.5 (Ph(*p*)), 49.8 ($CH_3CO\underline{C}H_2$), 40.7 ($NH\underline{C}H_2CH_2$), 35.6 ($NHCH_2\underline{C}H_2$), 30.9 ($\underline{C}H_3CO$); IR: 3303 (N-H), 1716 (C=O), 1644 (C=O), 1542 (C=O) cm^{-1}; MS(ES^-) (*m*/*z*): 204.0 $[M-H, 100\%]^-$; MS(ES^+) (*m*/*z*): 164.1 $[M-C_2H_3O, 47\%]^+$ 206.1 $[M+H, 100\%]^+$, 228.0 $[M+Na, 68\%]^+$, 244.0 $[M+K, 62\%]^+$.

(*Z*)-2-Acetyl-3-amino-4,4,4-trichloro-*N*-phenethylbut-2-enamide (4d). Yield: 90%, brown solid. mp: 89-91 ^oC; ^1H NMR (300 MHz, $CDCl_3$): δ 8.58 (br s, 2H, NH_2), 7.05-7.31 (m, 5H, Aryl-H), 3.61 (q, *J* = 6.5 Hz, 2H, $NHC\underline{H_2}CH_2$), 2.85 (t, *J* = 6.9 Hz, 2H, $NHCH_2C\underline{H_2}$), 2.16 (s, 3H, CH_3CO); ^{13}C NMR (75 MHz, $CDCl_3$): δ 197.7 ($CH_3\underline{C}O$), 167.7 ($C=\underline{C}(NH_2)CCl_3$), 156.5 (CONH), 138.4 (Ph(*i*)), 128.8 (Ph(*o*)), 128.7 (Ph(*m*)), 126.8 (Ph(*p*)), 104.8 ($\underline{C}=C(NH_2)CCl_3$), 93.3 ($CCl_3$), 41.4 ($NH\underline{C}H_2CH_2$), 34.7 ($NHCH_2\underline{C}H_2$), 28.3 ($\underline{C}H_3CO$); IR: 3293 (N-H), 1621 (C=O), 1536 (C=O), 1496 ($C=C-NH_2$) cm^{-1}.

5-Acetyl-6-(phenethylamino)-2,2-diphenyl-4-(trichloromethyl)-2,3-dihydro-1,3,2-oxazaborinin-1-ium-2-uide (NBC15). Ethyl acetate/*n*-hexane, 1:4. Yield: 18%, yellow oil. Exists as a mixture of rotamers in a 7:3 ratio. ^1H NMR (300 MHz, $CDCl_3$): δ 7.71 (br s, 1H, $C=C(NH)CCl_3$), 7.01-7.31 (m, 15H, 3 x Aryl-H of major and minor rotamers), 5.38 (br s, 1H, CONH of major rotamer), 5.31 (br s, 1H, CONH of minor rotamer), 3.70 (q, 2H, $NHC\underline{H_2}CH_2$ of minor rotamer), 3.47 (q, *J* = 6.5 Hz, 2H, $NHC\underline{H_2}CH_2$ of major rotamer), 2.86 (t, 2H, $NHCH_2C\underline{H_2}$ of minor rotamer), 2.72 (t, *J* =

6.9 Hz, 2H, $NHCH_2C\underline{H}_2$ of major rotamer), 2.17 (s, 3H, CH_3CO); ^{13}C NMR (75 MHz, $CDCl_3$): δ 187.2 ($CH_3\underline{C}O$), 164.8 (C=$\underline{C}$(NH)CCl_3), 162.1 (CONH), 138.2 (Ph(*i*)), 131.7 (B-Ph(*o*)), 128.8 (Ph(*m*)), 128.6 (Ph(*o*)), 127.6 (B-Ph(*m*)), 127.2 (Ph(*m*)), 127.0 (B-Ph(*p*)), 105.6 ($\underline{C}$=C(NH)CCl_3), 92.7 (CCl_3), 41.3 ($NH\underline{C}H_2CH_2$), 34.7 ($NHCH_2\underline{C}H_2$), 23.2 ($\underline{C}H_3CO$), B-Ph(*i*) quaternary signal not observed; IR: 3341 (N-H), 1649 (C=O), 1594 (C=C, conjugated), 1519 (C=C-NH) cm^{-1}; MS(ES^-) (*m*/*z*): 510.1 $[M-H, ^{10}B, ^{35}Cl, ^{35}Cl, ^{35}Cl, 6\%]^-$, 511.1 $[M-H, ^{11}B, ^{35}Cl, ^{35}Cl, ^{35}Cl, 33\%]^-$, 512.2 $[M-H, ^{10}B, ^{35}Cl, ^{35}Cl, ^{37}Cl, 13\%]^-$, 513.2 $[M-H, ^{11}B, ^{35}Cl, ^{35}Cl, ^{37}Cl, 32\%]^-$, 514.1 $[M-H, ^{10}B, ^{35}Cl, ^{37}Cl, ^{37}Cl, 7\%]^-$, 515.1 $[M-H, ^{11}B, ^{35}Cl, ^{37}Cl, ^{37}Cl, 11\%]^-$, 516.1 $[M-H, ^{10}B, ^{37}Cl, ^{37}Cl, ^{37}Cl, 2\%]^-$, 517.1 $[M-H, ^{11}B, ^{37}Cl, ^{37}Cl, ^{37}C, 1\%]^-$; MS($ES^+$) (*m*/*z*): 512.2 $[M+H, ^{10}B, ^{35}Cl, ^{35}Cl, ^{35}Cl, 14\%]^+$, 513.1 $[M+H, ^{11}B, ^{35}Cl, ^{35}Cl, ^{35}Cl, 68\%]^+$, 514.1 $[M+H, ^{10}B, ^{35}Cl, ^{35}Cl, ^{37}Cl, 31\%]^+$, 515.1 $[M+H, ^{11}B, ^{35}Cl, ^{35}Cl, ^{37}Cl, 74\%]^+$, 516.1 $[M+H, ^{10}B, ^{35}Cl, ^{37}Cl, ^{35}Cl, 30\%]^+$, 517.1 $[M+H, ^{11}B, ^{35}Cl, ^{37}Cl, ^{37}Cl, 22\%]^+$, 518.1 $[M+H, ^{10}B, ^{37}Cl, ^{37}Cl, ^{37}Cl, 13\%]^+$, 519.1 $[M+H, ^{11}B, ^{37}Cl, ^{37}Cl, ^{37}Cl, 11\%]^+$, 534.1 $[M+Na, ^{10}B, ^{35}Cl, ^{35}Cl, ^{35}Cl, 15\%]^+$, 535.1 $[M+Na, ^{11}B, ^{35}Cl, ^{35}Cl, ^{35}Cl, 41\%]^+$, 536.1 $[M+Na, ^{10}B, ^{35}Cl, ^{35}Cl, ^{37}Cl, 10\%]^+$, 537.1 $[M+Na, ^{11}B, ^{35}Cl, ^{35}Cl, ^{37}Cl, 33\%]^+$, 538.1 $[M+Na, ^{10}B, ^{35}Cl, ^{37}Cl, ^{37}Cl, 14\%]^+$, 539.1 $[M+Na, ^{11}B, ^{35}Cl, ^{37}Cl, ^{37}Cl, 26\%]^+$, 540.1 $[M+Na, ^{10}B, ^{37}Cl, ^{37}Cl, ^{37}Cl, 3\%]^+$, 540.1 $[M+Na, ^{11}B, ^{37}Cl, ^{37}Cl, ^{37}Cl, 2\%]^+$; HRMS($ES^+$) (*m*/*z*): $[M+H]^+$ calcd. for $C_{26}H_{25}{}^{11}B^{35}Cl_3N_2O_2$, 513.1069; found, 513.1073, error: 0.8 ppm.

(*Z*)-3-Amino-4,4,4-trichloro-*N*-phenethylbut-2-enamide (4e). Ethyl acetate/*n*-hexane, 1:3. Yield: 49%, brown oil. ^1H NMR (300 MHz, $CDCl_3$): δ 7.08-7.33 (m, 5H, Aryl-H), 6.61 (br s, 2H, NH_2), 5.41 (br s, 1H, $N\underline{H}CH_2CH_2$), 5.21 (s, 1H, $\underline{H}C=C(NH_2)CCl_3$), 3.50 (q, *J* = 6.6 Hz, 2H, $NHC\underline{H}_2CH_2$), 2.77 (t, *J* = 6.9 Hz, 2H, $NHCH_2C\underline{H}_2$); ^{13}C NMR (75 MHz, $CDCl_3$): δ 168.4 (C=$\underline{C}(NH_2)CCl_3$), 155.4 (CONH), 138.9 (Ph(*i*)), 128.8 (Ph(*m*)), 128.7 (Ph(*o*)), 126.5 (Ph(*p*)), 94.5 (CCl_3), 87.8

($\underline{C}$=C(NH$_2$)CCl$_3$), 40.4 (NH$\underline{C}$H$_2$CH$_2$), 35.9 (NHCH$_2\underline{C}$H$_2$); MS(ES$^-$) (*m/z*): 306.0 [M-H, ^{35}Cl, ^{35}Cl, ^{35}Cl, 100%]$^-$, 308.0 [M-H, ^{35}Cl, ^{35}Cl, ^{37}Cl, 92%]$^-$, 310.0 [M-H, ^{35}Cl, ^{37}Cl, ^{37}Cl, 30%]$^-$, 312.0 [M-H, ^{37}Cl, ^{37}Cl, ^{37}Cl, 5%]$^-$; MS(ES$^+$) (*m/z*): 307.0 [M+H, ^{35}Cl, ^{35}Cl, ^{35}Cl, 100%]$^+$, 309.0 [M+H, ^{35}Cl, ^{35}Cl, ^{37}Cl, 95%]$^+$, 311.0 [M+H, ^{35}Cl, ^{37}Cl, ^{37}Cl, 26%]$^+$, 313.0 [M+H, ^{37}Cl, ^{37}Cl, ^{37}Cl, 2%]$^+$, 329.0 [M+Na, ^{35}Cl, ^{35}Cl, ^{35}Cl, 84%]$^+$, 331.0 [M+Na, ^{35}Cl, ^{35}Cl, ^{37}Cl, 69%]$^+$, 333.0 [M+Na, ^{35}Cl, ^{37}Cl, ^{37}Cl, 25%]$^+$, 335.0 [M+Na, ^{37}Cl, ^{37}Cl, ^{37}Cl, 4%]$^+$, 345.0 [M+K, ^{35}Cl, ^{35}Cl, ^{35}Cl, 11%]$^+$, 347.0 [M+K, ^{35}Cl, ^{35}Cl, ^{37}Cl, 13%]$^+$, 349.0 [M+K, ^{35}Cl, ^{37}Cl, ^{37}Cl, 10%]$^+$, 351.0 [M+K, ^{37}Cl, ^{37}Cl, ^{37}Cl, 3%]$^+$. HRMS(ES$^+$) (*m/z*): [M+H]$^+$ calcd. for $C_{12}H_{13}{}^{35}Cl_2N_2O$, 271.0405; found, 271.0404, error: 0.4 ppm.

6-(Phenethylamino)-2,2-diphenyl-4-(trichloromethyl)-2,3-dihydro-1,3,2-oxazaborinin-1-ium-2-uide (NBC16). Ethyl acetate/*n*-hexane, 1:4. Yield: 18%, yellow solid. mp: 106-108 ^{o}C, exists as a mixture of rotamers in ~ 3:1 ratio. ^{1}H NMR (300 MHz, CDCl$_3$): δ 7.05-7.41 (m, 15H, 3 x Aryl-H of major and minor rotamers), 6.36 (br s, 1H, C=C(NH)CCl$_3$ of minor rotamer), 5.95 (br s, 1H, C=C(NH)CCl$_3$ of major rotamer), 5.47 (br s, 1H, CONH of minor rotamer), 5.32 (br s, 1H, CONH of major rotamer), 5.14 (s, 1H, $\underline{H}$C=C(NH)CCl$_3$ of minor rotamer), 5.06 (d, *J* = 1.8 Hz, 1H, $\underline{H}$C=C(NH)CCl$_3$ of major rotamer), 3.67 (q, *J* = 6.6 Hz, 2H, NHC$\underline{H_2}$CH$_2$ of major rotamer), 3.42 (q, 2H, NHC$\underline{H_2}$CH$_2$ of minor rotamer), 2.83 (t, *J* = 6.8 Hz, 2H, NHCH$_2$C$\underline{H_2}$); ^{13}C NMR (75 MHz, CDCl$_3$): δ 169.3 (CONH), 162.9 (C=$\underline{C}$(NH)CCl$_3$), 137.8 (Ph(*i*)), 131.8 (B-Ph(*o*)), 128.9 (Ph(*m*)), 128.8 (Ph(*o*)), 127.3 (B-Ph(*m*)), 126.5 (Ph(*p*)), 126.3 (B-Ph(*p*)), 94.5 (CCl$_3$), 42.2 (NH$\underline{C}$H$_2$CH$_2$), 35.8 (NHCH$_2\underline{C}$H$_2$), B-Ph(*i*) quaternary signal and $\underline{C}$=C(NH)CCl$_3$ signal not observed; IR: 3367 (N-H), 1580 (C=C, conjugated), 1509 (C=C-NH) cm^{-1}; MS(ES$^-$) (*m/z*): 468.1 [M-H, ^{10}B, ^{35}Cl, ^{35}Cl, ^{35}Cl, 14%]$^-$, 469.1 [M-H, ^{11}B, ^{35}Cl, ^{35}Cl, ^{35}Cl, 100%]$^-$, 470.1 [M-H, ^{10}B, ^{35}Cl, ^{35}Cl, ^{37}Cl, 48%]$^-$, 471.2 [M-H, ^{11}B, ^{35}Cl, ^{35}Cl, ^{37}Cl, 77%]$^-$, 472.1 [M-H, ^{10}B, ^{35}Cl, ^{37}Cl, ^{37}Cl, 31%]$^-$,

473.2 [M-H, ^{11}B, ^{35}Cl, ^{37}Cl, ^{37}Cl, 24%]$^-$, 474.0 [M-H, ^{10}B, ^{37}Cl, ^{37}Cl, ^{37}Cl, 5%]$^-$, 475.2 [M-H, ^{11}B, ^{37}Cl, ^{37}Cl, ^{37}C, 3%]$^-$; MS(ES$^+$) (*m*/*z*): 392.1 [M-Ph, ^{10}B, ^{35}Cl, ^{35}Cl, ^{35}Cl, 13%]$^+$, 393.1 [M-Ph, ^{11}B, ^{35}Cl, ^{35}Cl, ^{35}Cl, 40%]$^+$, 394.1 [M-Ph, ^{10}B, ^{35}Cl, ^{35}Cl, ^{37}Cl, 30%]$^+$, 395.0 [M-Ph, ^{11}B, ^{35}Cl, ^{35}Cl, ^{37}Cl, 59%]$^+$, 396.1 [M-Ph, ^{10}B, ^{35}Cl, ^{37}Cl, ^{37}Cl, 12%]$^+$, 397.1 [M-Ph, ^{11}B, ^{35}Cl, ^{37}Cl, ^{37}Cl, 15%]$^+$, 398.1 [M-Ph, ^{10}B, ^{37}Cl, ^{37}Cl, ^{37}Cl, 5%]$^+$, 399.1 [M-Ph, ^{11}B, ^{37}Cl, ^{37}Cl, ^{37}Cl, 11%]$^+$, 470.1 [M+H, ^{10}B, ^{35}Cl, ^{35}Cl, ^{35}Cl, 22%]$^+$, 471.1 [M+H, ^{11}B, ^{35}Cl, ^{35}Cl, ^{35}Cl, 100%]$^+$, 472.1 [M+H, ^{10}B, ^{35}Cl, ^{35}Cl, ^{37}Cl, 36%]$^+$, 473.1 [M+H, ^{11}B, ^{35}Cl, ^{35}Cl, ^{37}Cl, 98%]$^+$, 474.1 [M+H, ^{10}B, ^{35}Cl, ^{37}Cl, ^{35}Cl, 37%]$^+$, 475.2 [M+H, ^{11}B, ^{35}Cl, ^{37}Cl, ^{37}Cl, 28%]$^+$, 476.2 [M+H, ^{10}B, ^{37}Cl, ^{37}Cl, ^{37}Cl, 12%]$^+$, 477.2 [M+H, ^{11}B, ^{37}Cl, ^{37}Cl, ^{37}Cl, 4%]$^+$, 492.1 [M+Na, ^{10}B, ^{35}Cl, ^{35}Cl, ^{35}Cl, 3%]$^+$, 493.1 [M+Na, ^{11}B, ^{35}Cl, ^{35}Cl, ^{35}Cl, 22%]$^+$, 494.1 [M+Na, ^{10}B, ^{35}Cl, ^{35}Cl, ^{37}Cl, 4%]$^+$, 495.2 [M+Na, ^{11}B, ^{35}Cl, ^{35}Cl, ^{37}Cl, 10%]$^+$, 496.2 [M+Na, ^{10}B, ^{35}Cl, ^{37}Cl, ^{37}Cl, 7%]$^+$, 497.2 [M+Na, ^{11}B, ^{35}Cl, ^{37}Cl, ^{37}Cl, 5%]$^+$, 498.2 [M+Na, ^{10}B, ^{37}Cl, ^{37}Cl, ^{37}Cl, 3%]$^+$, 499.2 [M+Na, ^{11}B, ^{37}Cl, ^{37}Cl, ^{37}Cl, 2%]$^+$, 508.1 [M+K, ^{10}B, ^{35}Cl, ^{35}Cl, ^{35}Cl, 3%]$^+$, 509.1 [M+K, ^{11}B, ^{35}Cl, ^{35}Cl, ^{35}Cl, 20%]$^+$, 510.1 [M+K, ^{10}B, ^{35}Cl, ^{35}Cl, ^{37}Cl, 4%]$^+$, 511.2 [M+K, ^{11}B, ^{35}Cl, ^{35}Cl, ^{37}Cl, 13%]$^+$, 512.2 [M+K, ^{10}B, ^{35}Cl, ^{37}Cl, ^{37}Cl, 5%]$^+$, 513.2 [M+K, ^{11}B, ^{35}Cl, ^{37}Cl, ^{37}Cl, 8%]$^+$, 514.2 [M+K, ^{10}B, ^{37}Cl, ^{37}Cl, ^{37}Cl, 3%]$^+$, 515.2 [M+K, ^{11}B, ^{37}Cl, ^{37}Cl, ^{37}Cl, 4%]$^+$. HRMS(ES$^-$) (*m*/*z*): [M-H]$^-$ calcd. for $C_{24}H_{21}{}^{11}B^{35}Cl_3N_2O$, 469.0818; found, 469.0830, error: 2.6 ppm.

***N*-Adamantylacetoacetamide (3f)**. Yield: 55%, orange solid. mp: 81-82 ^oC {lit.(Clemens and Hyatt, 1985) 82-83 ^oC); ^1H NMR (300 MHz, $CDCl_3$): δ 6.50 (br s, 1H, NH), 3.32 (s, 2H, $CH_3COC\underline{H}_2$), 2.26 (s, 3H, CH_3CO), 2.07 (m, 3H, Adamantyl-H_2), 2.01 (m, 6H, Adamantyl-H_3 and Adamantyl-H_2), 1.68 (m, 6H, Adamantyl-H_1); ^{13}C NMR (75 MHz, $CDCl_3$): δ 204.9 ($CH_3\underline{C}O$), 164.2 (CONH), 52.1 (Adamantyl-C_4), 51.2 ($CH_3CO\underline{C}H_2$), 41.5 (Adamantyl-C_3), 36.3 (Adamantyl-C_2), 30.9 ($\underline{C}H_3CO$), 29.4

(Adamantyl-C_1); IR: 3310 (N-H), 2903 (Adamantyl CH_2 stretch), 2849 (Adamantyl CH_2 stretch), 1721 (C=O), 1639 (C=O), 1545 (C=O) cm^{-1}; MS(ES^-) (*m*/*z*): 270.1 [M+Cl, ^{35}Cl, 100%]$^-$, 272.2 [M+Cl, ^{37}Cl, 48%]$^-$; MS(ES^+) (*m*/*z*): 236.1 [M+H, 100%]$^+$, 471.4 [2M+H, 93%]$^+$, 493.4 [2M+Na, 49%]$^+$. HRMS($APCI^+$) (*m*/*z*): [M+H]$^+$ calcd. for $C_{14}H_{22}NO_2$, 236.1645; found, 236.1633, error: 5.1 ppm.

(*Z*)-2-Acetyl-3-amino-4,4,4-trichloro-*N*-adamantylbut-2-enamide (4f). Yield: 41%, white solid. mp: 105-106 ^oC; ^1H NMR (300 MHz, $CDCl_3$): δ 8.55 (br s, 2H, NH_2), 5.54 (br s, 1H, CONH) 2.29 (s, 3H, CH_3CO), 2.02 (m, 10H, Adamantyl-H), 1.91 (d, *J* = 2.4 Hz, 1H, Adamantyl-H), 1.64 (m, 7H, Adamantyl-H); ^{13}C NMR (75 MHz, $CDCl_3$): δ 198.2 ($CH_3\underline{C}O$), 166.1 (C=$\underline{C}$(NH_2)CCl_3), 155.9 (CONH), 106.6 ($\underline{C}$=C(NH_2)CCl_3), 93.7 (CCl_3), 52.7 (Adamantyl-C_4), 41.1 (Adamantyl-C_3), 36.3 (Adamantyl-C_2), 29.4 (Adamantyl-C_1), 28.4 ($\underline{C}H_3CO$); IR: 3309 (N-H), 2903 (Adamantyl CH_2 stretch), 2849 (Adamantyl CH_2 stretch), 1633 (C=O), 1617 (C=C, conjugated), 1517 (C=C-NH_2) cm^{-1}; MS(ES^-) (*m*/*z*): 377.1 [M-H, ^{35}Cl, ^{35}Cl, ^{35}Cl, 12%]$^-$, 379.1 [M-H, ^{35}Cl, ^{35}Cl, ^{37}Cl, 9%]$^-$, 381.1 [M-H, ^{35}Cl, ^{37}Cl, ^{37}Cl, 4%]$^-$, 383.1 [M-H, ^{37}Cl, ^{37}Cl, ^{37}Cl, 1%]$^-$, 413.1 [M+^{35}Cl, ^{35}Cl, ^{35}Cl, ^{35}Cl, 91%]$^-$, 414.1 [M+^{37}Cl, ^{35}Cl, ^{35}Cl, ^{35}Cl, 8%]$^-$, 415.1 [M+^{35}Cl, ^{35}Cl, ^{35}Cl, ^{37}Cl, 100%]$^-$, 416.1 [M+^{37}Cl, ^{35}Cl, ^{35}Cl, ^{37}Cl, 15%]$^-$, 417.1 [M+^{35}Cl, ^{35}Cl, ^{37}Cl, ^{37}Cl, 44%]$^-$, 418.1 [M+^{37}Cl, ^{35}Cl, ^{37}Cl, ^{37}Cl, 13%]$^-$, 419.1 [M+^{35}Cl, ^{37}Cl, ^{37}Cl, ^{37}Cl, 7%]$^-$, 420.2 [M+^{37}Cl, ^{37}Cl, ^{37}Cl, ^{37}Cl, 3%]$^-$; MS(ES^+) (*m*/*z*): 379.1 [M+H, ^{35}Cl, ^{35}Cl, ^{35}Cl, 100%]$^+$, 381.1 [M+H, ^{35}Cl, ^{35}Cl, ^{37}Cl, 45%]$^+$, 383.1 [M+H, ^{35}Cl, ^{37}Cl, ^{37}Cl, 13%]$^+$, 385.1 [M+H, ^{37}Cl, ^{37}Cl, ^{37}Cl, 3%]$^+$, 401.1 [M+Na, ^{35}Cl, ^{35}Cl, ^{35}Cl, 81%]$^+$, 403.1 [M+Na, ^{35}Cl, ^{35}Cl, ^{37}Cl, 84%]$^+$, 405.1 [M+Na, ^{35}Cl, ^{37}Cl, ^{37}Cl, 19%]$^+$, 407.1 [M+Na, ^{37}Cl, ^{37}Cl, ^{37}Cl, 4%]$^+$. HRMS($APCI^+$) (*m*/*z*): [M+H]$^+$ calcd. for $C_{16}H_{22}{}^{35}Cl_3N_2O_2$, 379.0741; found, 379.0738, error: 0.8 ppm.

5-Acetyl-6-(adamantan-1-ylamino)-2,2-diphenyl-4-(trichloromethyl)-2,3-dihydro-1,3,2-oxazaborinin-1-ium-2-uide (NBC17). Ethyl acetate/*n*-hexane, 1:19. Yield: 33%, yellow solid. mp: 115-116 ^{o}C; ^{1}H NMR (300 MHz, $CDCl_3$): δ 7.73 (br s, 1H, C=C(NH)CCl_3), 7.12-7.33 (m, 10H, Aryl-H), 5.08 (br s, 1H, CONH), 2.29 (s, 3H, CH_3CO), 1.99 (m, 3H, Adamantyl-H), 1.87 (m, 6H, Adamantyl-H), 1.59 (m, 6H, Adamantyl-H); ^{13}C NMR (75 MHz, $CDCl_3$): δ 186.9 (CH_3<u>C</u>O), 163.4 (C=<u>C</u>(NH)CCl_3), 162.0 (CONH), 131.7 (B-Ph(*o*)), 127.5 (B-Ph(*m*)), 127.0 (B-Ph(*p*)), 107.1 (<u>C</u>=C(NH)CCl_3), 93.0 (CCl_3), 52.9 (Adamantyl-C_4), 41.0 (Adamantyl-C_3), 36.2 (Adamantyl-C_2), 29.3 (Adamantyl-C_1), 23.2 (<u>C</u>H_3CO), B-Ph(*i*) quaternary signal not observed; IR: 3318 (N-H), 2909 (Adamantyl CH_2 stretch), 2851 (Adamantyl CH_2 stretch), 1638 (C=O), 1603 (C=C, conjugated), 1526 (C=C-NH) cm^{-1}; MS(ES^-) (*m/z*): 540.2 [M-H, ^{10}B, ^{35}Cl, ^{35}Cl, ^{35}Cl, 10%]$^-$, 541.2 [M-H, ^{11}B, ^{35}Cl, ^{35}Cl, ^{35}Cl, 100%]$^-$, 542.2 [M-H, ^{10}B, ^{35}Cl, ^{35}Cl, ^{37}Cl, 45%]$^-$, 543.2 [M-H, ^{11}B, ^{35}Cl, ^{35}Cl, ^{37}Cl, 64%]$^-$, 544.2 [M-H, ^{10}B, ^{35}Cl, ^{37}Cl, ^{37}Cl, 23%]$^-$, 545.2 [M-H, ^{11}B, ^{35}Cl, ^{37}Cl, ^{37}Cl, 33%]$^-$, 546.2 [M-H, ^{10}B, ^{37}Cl, ^{37}Cl, ^{37}Cl, 3%]$^-$, 547.2 [M-H, ^{11}B, ^{37}Cl, ^{37}Cl, ^{37}C, 2%]$^-$; MS(ES^+) (*m/z*): 542.2 [M+H, ^{10}B, ^{35}Cl, ^{35}Cl, ^{35}Cl, 22%]$^+$, 543.2 [M+H, ^{11}B, ^{35}Cl, ^{35}Cl, ^{35}Cl, 100%]$^+$, 544.2 [M+H, ^{10}B, ^{35}Cl, ^{35}Cl, ^{37}Cl, 39%]$^+$, 545.2 [M+H, ^{11}B, ^{35}Cl, ^{35}Cl, ^{37}Cl, 70%]$^+$, 546.2 [M+H, ^{10}B, ^{35}Cl, ^{37}Cl, ^{35}Cl, 35%]$^+$, 547.2 [M+H, ^{11}B, ^{35}Cl, ^{37}Cl, ^{37}Cl, 24%]$^+$, 548.2 [M+H, ^{10}B, ^{37}Cl, ^{37}Cl, ^{37}Cl, 11%]$^+$, 549.2 [M+H, ^{11}B, ^{37}Cl, ^{37}Cl, ^{37}Cl, 2%]$^+$, 534.1 [M+Na, ^{10}B, ^{35}Cl, ^{35}Cl, ^{35}Cl, 15%]$^+$, 535.1 [M+Na, ^{11}B, ^{35}Cl, ^{35}Cl, ^{35}Cl, 41%]$^+$, 536.1 [M+Na, ^{10}B, ^{35}Cl, ^{35}Cl, ^{37}Cl, 10%]$^+$, 537.1 [M+Na, ^{11}B, ^{35}Cl, ^{35}Cl, ^{37}Cl, 33%]$^+$, 538.1 [M+Na, ^{10}B, ^{35}Cl, ^{37}Cl, ^{37}Cl, 14%]$^+$, 539.1 [M+Na, ^{11}B, ^{35}Cl, ^{37}Cl, ^{37}Cl, 26%]$^+$, 540.1 [M+Na, ^{10}B, ^{37}Cl, ^{37}Cl, ^{37}Cl, 3%]$^+$, 540.1 [M+Na, ^{11}B, ^{37}Cl, ^{37}Cl, ^{37}Cl, 2%]$^+$. HRMS(ES^+) (*m/z*): $[M+Na]^+$ calcd. for $C_{28}H_{30}{}^{11}B^{35}Cl_3N_2O_2Na$, 565.1358; found, 565.1359, error: 0.2 ppm.

***N*-Cyclohexylacetoacetamide (3g)**. Yield: 78%, cream solid. mp: 75-76°C (lit.(Sridharan et al., 2010) 76-77 ^{o}C); ^{1}H NMR (300 MHz, $CDCl_3$): δ 7.08 (br s, 1H, NH), 3.70 (p, *J* = 3.6 Hz, 1H, -C*H*$CH_2CH_2CH_2CH_2CH_2$-), 3.36 (s, 2H, CH_3COC*H_2*), 2.20 (s, 3H, CH_3CO); 1.82 (d, *J* = 11.7 Hz, 2H, -CHC*H_2*$(CH_2)_3$C*H_2*-), 1.04-1.70 (m, 8H, -CHC*H_2*(C*H_2*$)_3$C*H_2*-); ^{13}C NMR (75 MHz, $CDCl_3$): δ 204.8 (CH_3*C*O), 164.3 (CONH), 49.9 (CH_3CO*C*H_2), 48.2 (-*C*H$(CH_2)_2(CH_2)_2CH_2$-), 32.8 (-CH(*C*$H_2)_2(CH_2)_2CH_2$-), 31.0 (*C*H_3CO), 25.5 (-CH$(CH_2)_2(CH_2)_2$*C*H_2), 24.7 (-CH$(CH_2)_2$(*C*$H_2)_2CH_2$-); IR: 3290 (N-H), 1716 (C=O), 1638 (C=O), 1543 (C=O) cm^{-1}; MS(ES^{+}) (*m*/*z*): 184.1 [M+H, 100%]$^{+}$, 206.1 [M+Na, 82%]$^{+}$. HRMS(ES^{+}) (*m*/*z*): [M+H]$^{+}$ calcd. for $C_{10}H_{18}NO_2$, 184.1332; found, 184.1329, error: 1.6 ppm.

(*Z*)-2-Acetyl-3-amino-4,4,4-trichloro-*N*-cyclohexylbut-2-enamide (4g). Yield: 93%, cream solid. mp: 99-101 ^{o}C (dec); ^{1}H NMR (300 MHz, $CDCl_3$): δ 8.66 (br s, 1H, NH_2), 5.83 (d, *J* = 7.5 Hz, 1H, NH), 3.81-3.97 (m, *J* = 3.6 Hz, 1H, -C*H*$CH_2CH_2CH_2CH_2CH_2$-), 2.32 (s, 3H, CH_3CO); 1.95-2.08 (m, 2H, -CHC*H_2*$(CH_2)_3$C*H_2*-), 1.53-1.79 (m, 4H, -CHC*H_2*(C*H_2*$)_3$C*H_2*-), 1.32-1.49 (m, 2H, -$CHCH_2$(C*H_2*$)_3CH_2$-), 1.11-1.27 (m, 3H, -$CHCH_2$(C*H_2*$)_3CH_2$-); ^{13}C NMR (75 MHz, $CDCl_3$): δ 197.8 (CH_3*C*O), 166.5 (C=*C*(NH_2)CCl_3), 156.3 (CONH), 105.3 (*C*=C(NH_2)CCl_3), 93.5 (CCl_3), 49.0 (-*C*H$(CH_2)_2(CH_2)_2CH_2$-), 32.4 (-CH(*C*$H_2)_2(CH_2)_2CH_2$-), 28.4 (*C*H_3CO), 25.5 (-CH$(CH_2)_2(CH_2)_2$*C*H_2-), 24.7 (-CH$(CH_2)_2$(*C*$H_2)_2CH_2$-); IR: 3271 (N-H), 2929 (N-H), 1721 (C=O), 1620 (C=O), 1535 (C=C-NH_2) cm^{-1}; MS(ES^{-}) (*m*/*z*): 325.0 [M-H, ^{35}Cl, ^{35}Cl, ^{35}Cl, 100%]$^{-}$, 327.0 [M-H, ^{35}Cl, ^{35}Cl, ^{37}Cl, 53%]$^{-}$, 329.0 [M-H, ^{35}Cl, ^{37}Cl, ^{37}Cl, 33%]$^{-}$, 331.0 [M-H, ^{37}Cl, ^{37}Cl, ^{37}Cl, 3%]$^{-}$, 361.0 [M+^{35}Cl, ^{35}Cl, ^{35}Cl, ^{35}Cl, 87%]$^{-}$, 363.0 [M+^{35}Cl, ^{35}Cl, ^{35}Cl, ^{37}Cl, 48%]$^{-}$, 365.0 [M+^{35}Cl, ^{35}Cl, ^{37}Cl, ^{37}Cl, 24%]$^{-}$, 367.0 [M+^{35}Cl, ^{37}Cl, ^{37}Cl, ^{37}Cl, 5%]$^{-}$; MS(ES^{+}) (*m*/*z*): 327.0 [M+H, ^{35}Cl, ^{35}Cl, ^{35}Cl, 62%]$^{+}$, 329.0 [M+H, ^{35}Cl, ^{35}Cl, ^{37}Cl, 41%]$^{+}$, 331.0

[M+H, ^{35}Cl, ^{37}Cl, ^{37}Cl, 15%]$^+$, 333.0 [M+H, ^{37}Cl, ^{37}Cl, ^{37}Cl, 2%]$^+$; HRMS(ES$^+$) (*m/z*): [M+H]$^+$ calcd. for $C_{12}H_{18}{}^{35}Cl_3N_2O_2$, 327.0428; found, 327.0426, error: 0.6 ppm.

5-Acetyl-6-(cyclohexylamino)-2,2-diphenyl-4-(trichloromethyl)-2,3-dihydro-1,3,2-oxazaborinin-1-ium-2-uide (NBC19, 17). Ethyl acetate/*n*-hexane, 1:4. Yield: 9%, orange solid. mp: 53-54°C; ^{1}H NMR (300 MHz, $CDCl_3$): δ 7.82 (br s, 1H, C=C(NH)CCl_3), 7.27-7.39 (m, 10H, 2 x Aryl-H), 5.38 (d, *J* = 7.5 Hz, 1H, CONH), 3.69-3.85 (m, 1H, -C<u>*H*</u>$(CH_2)_2(CH_2)_2CH_2$-), 2.35 (s, 3H, CH_3CO); 1.89 (d, *J* = 10.5 Hz, 2H, -CHC<u>*H*</u>$_2(CH_2)_3$C<u>*H*</u>$_2$-), 0.98-1.42 (m, 8H, -CHC<u>*H*</u>$_2$(C<u>*H*</u>$_2$)$_3$C<u>*H*</u>$_2$-); ^{13}C NMR (75 MHz, $CDCl_3$): δ 187.2 (CH_3<u>*C*</u>O), 163.8 (C=<u>*C*</u>(NH)CCl_3), 162.3 (CONH), 156.3 (B-Ph(*i*)), 131.9 (B-Ph(*o*)), 127.7 (B-Ph(*m*)), 127.2 (B-Ph(*p*)), 105.9 (<u>*C*</u>=C(NH)CCl_3), 93.1 (CCl_3), 49.3 (-<u>*C*</u>H$(CH_2)_2(CH_2)_2CH_2$-), 32.6 (-CH(<u>*C*</u>H$_2$)$_2(CH_2)_2CH_2$-), 25.5 (-CH$(CH_2)_2(CH_2)_2$<u>*C*</u>H$_2$-), 24.8 (-CH$(CH_2)_2$(<u>*C*</u>H$_2$)$_2CH_2$-), 23.4 (<u>*C*</u>H$_3$CO); IR: 3311 (N-H), 2928 (N-H), 1631 (C=O), 1594 (C=O), 1514 (C=C-NH) cm^{-1}; MS(ES$^-$) (*m/z*): 447.1 [M-C_2H_3O, ^{10}B, ^{35}Cl, ^{35}Cl, ^{35}Cl, 20%]$^-$, 448.1 [M-C_2H_3O, ^{35}Cl, ^{35}Cl, ^{35}Cl, 99%]$^-$, 449.1 [M-C_2H_3O, ^{10}B, ^{35}Cl, ^{35}Cl, ^{37}Cl, 33%]$^-$, 450.1 [M-C_2H_3O, ^{11}B, ^{35}Cl, ^{35}Cl, ^{37}Cl, 100%]$^-$, 451.1 [M-C_2H_3O, ^{10}B, ^{35}Cl, ^{37}Cl, ^{37}Cl, 25%]$^-$, 452.1 [M-C_2H_3O, ^{11}B, ^{35}Cl, ^{37}Cl, ^{35}Cl, 23%]$^-$, 453.1 [M-C_2H_3O, ^{10}B, ^{37}Cl, ^{37}Cl, ^{37}Cl, 8%]$^-$, 454.1 [M-C_2H_3O, ^{11}B, ^{37}Cl, ^{37}Cl, ^{37}Cl, 3%]$^-$, 488.1 [M-H, ^{10}B, ^{35}Cl, ^{35}Cl, ^{35}Cl, 32%]$^-$, 489.1 [M-H, ^{11}B, ^{35}Cl, ^{35}Cl, ^{35}Cl, 100%]$^-$, 490.1 [M-H, ^{10}B, ^{35}Cl, ^{35}Cl, ^{37}Cl, 48%]$^-$, 491.2 [M-H, ^{11}B, ^{35}Cl, ^{35}Cl, ^{37}Cl, 94%]$^-$, 492.2 [M-H, ^{10}B, ^{35}Cl, ^{37}Cl, ^{35}Cl, 22%]$^-$, 493.2 [M-H, ^{11}B, ^{35}Cl, ^{37}Cl, ^{37}Cl, 32%]$^-$, 494.2 [M-H, ^{10}B, ^{37}Cl, ^{37}Cl, ^{37}Cl, 5%]$^-$, 495.2 [M-H, ^{11}B, ^{37}Cl, ^{37}Cl, ^{37}Cl, 4%]$^-$. MS(ES$^+$) (*m/z*): 490.2 [M+H, ^{10}B, ^{35}Cl, ^{35}Cl, ^{35}Cl, 13%]$^+$, 491.2 [M+H, ^{11}B, ^{35}Cl, ^{35}Cl, ^{35}Cl, 100%]$^+$, 492.2 [M+H, ^{10}B, ^{35}Cl, ^{35}Cl, ^{37}Cl, 45%]$^+$, 493.2 [M+H, ^{11}B, ^{35}Cl, ^{35}Cl, ^{37}Cl, 46%]$^+$, 494.2 [M+H, ^{10}B, ^{35}Cl, ^{37}Cl, ^{37}Cl, 18%]$^+$, 495.2 [M+H, ^{11}B, ^{35}Cl, ^{37}Cl, ^{37}Cl, 24%]$^+$, 496.2 [M+H, ^{10}B, ^{37}Cl, ^{37}Cl, ^{37}Cl, 6%]$^+$, 497.2 [M+H, ^{11}B, ^{37}Cl, ^{37}Cl, ^{37}Cl, 3%]$^+$.

HRMS(ES^+) (*m*/*z*): $[M+H]^+$ calcd. for $C_{24}H_{27}{}^{11}B^{35}Cl_3N_2O_2$, 491.1226; found, 491.1227, error: 0.2 ppm.

***N*-(Pyren-1-ylmethyl)acetoacetamide (3h)**. *tert*-Butyl acetoacetate (0.14 ml, 0.82 mmol) was added to a solution of 1-pyrenemethylamine hydrochloride (0.20 g, 0.75 mmol) and triethylamine (0.21 ml, 1.49 mmol) in toluene (50 ml). The procedure was carried out as described previously. Yield: 0.23 g (98%), orange solid. mp: 130-132 ^oC; ^1H NMR (300 MHz, $CDCl_3$): δ 7.88-8.21 (m, 9H, pyrene-H), 7.27 (br s, 1H, NH), 5.12 (d, *J* = 5.4 Hz, 2H, $C\underline{H_2}NH$) 3.41 (s, 2H, $CH_3COC\underline{H_2}$), 2.17 (s, 3H, CH_3CO); ^{13}C NMR (75 MHz, $CDCl_3$): δ 204.4 ($CH_3\underline{C}O$), 165.1 (CONH), 131.3, 130.8, 129.03, 128.96, 128.3, 128.2, 127.5, 127.4, 127.0, 126.1, 125.4, 125.3, 125.0, 124.8, 124.7, 122.7, 49.5 ($CH_3CO\underline{C}H_2$), 41.9 (CH_2NH) 31.0 ($\underline{C}H_3CO$); IR: 3284 (N-H), 1714 (C=O), 1622 (C=O), 1536 (C=O) cm^{-1}; MS(ES^-) (*m*/*z*): 314.3 $[M-H, 15\%]^-$, 350.1 [M+Cl, ^{35}Cl, $30\%]^-$, 352.1 [M+Cl, ^{37}Cl, $12\%]^-$;MS(ES^+) (*m*/*z*): 316.1 $[M+H, 84\%]^+$, 338.3 [M+Na, $23\%]^+$. HRMS(ES^-) (*m*/*z*): $[M-H]^-$ calcd. for $C_{21}H_{16}NO_2$, 314.1187; found, 314.1185, error: 0.6 ppm.

(*Z*)-2-Acetyl-3-amino-4,4,4-trichloro-*N*-(pyren-1-ylmethyl)but-2-enamide (4h). Yield: 76%, orange solid. mp: 124-126 ^oC (dec); ^1H NMR (300 MHz, $CDCl_3$): δ 8.29 (d, *J* = 9.0 Hz, 1H, pyrene-C8-H), 7.92-8.19 (m, 8H, pyrene-H), 6.21 (br s, 1H, $CON\underline{H}CH_2$), 5.18 (d, *J* = 5.1 Hz, 2H, $CONHC\underline{H_2}$), 2.28 (s, 3H, CH_3CO); ^{13}C NMR (75 MHz, $CDCl_3$): δ 197.6 ($CH_3\underline{C}O$), 167.2 ($C{=}\underline{C}(NH_2)CCl_3$), 156.7 (CONH), 131.5, 131.3, 130.7, 129.9, 128.4, 127.84, 127.77, 127.3, 126.2, 125.6, 125.5, 124.9, 122.9, 104.5 ($\underline{C}{=}C(NH_2)CCl_3$), 93.4 ($CCl_3$), 43.2 ($CH_3CO\underline{C}H_2$), 28.6 ($\underline{C}H_3CO$), $CONHCH_2\underline{C}$ quaternary signal not observed; IR: 3248 (N-H), 1717 (C=O), 1620 (C=O), 1520 ($C{=}C{-}NH_2$) cm^{-1}; MS(ES^-) (*m*/*z*): 416.0 [$M-C_2H_3O$, ^{35}Cl, ^{35}Cl, ^{35}Cl, $50\%]^-$, 418.1 [$M-C_2H_3O$, ^{35}Cl, ^{35}Cl, ^{37}Cl, $23\%]^-$, 420.1 [$M-C_2H_3O$, ^{35}Cl, ^{37}Cl, ^{37}Cl, $20\%]^-$, 421.1 [M-

C_2H_3O, ^{37}Cl, ^{37}Cl, ^{37}Cl, 8%]$^-$, 457.1 [M-H, ^{35}Cl, ^{35}Cl, ^{35}Cl, 65%]$^-$, 459.0 [M-H, ^{35}Cl, ^{35}Cl, ^{37}Cl, 100%]$^-$, 461.1 [M-H, ^{35}Cl, ^{37}Cl, ^{37}Cl, 12%]$^-$, 463.1 [M-H, ^{37}Cl, ^{37}Cl, ^{37}Cl, 3%]$^-$, 493.0 [M+^{35}Cl, ^{35}Cl, ^{35}Cl, ^{35}Cl, 34%]$^-$, 495.0 [M+^{35}Cl, ^{35}Cl, ^{35}Cl, ^{37}Cl, 35%]$^-$, 497.0 [M+^{35}Cl, ^{35}Cl, ^{37}Cl, ^{37}Cl, 26%]$^-$, 499.0 [M+^{35}Cl, ^{37}Cl, ^{37}Cl, ^{37}Cl, 4%]$^-$. HRMS(ES$^-$) (*m/z*): [M-H]$^-$ calcd. for $C_{23}H_{16}{}^{35}Cl_3N_2O_2$, 457.0283; found, 457.0277, error: 1.3 ppm.

5-Acetyl-2,2-diphenyl-6-((pyren-1-ylmethyl)amino)-4-(trichloromethyl)-2,3-dihydro-1,3,2-oxazaborinin-1-ium-2-uide (NBC20). Ethyl acetate/*n*-hexane, 1:4. Yield: 12%, yellow solid. mp: 167-169 ^oC (dec); ^1H NMR (300 MHz, $CDCl_3$): δ 8.03-8.30 (m, 8H, pyrene-H), 7.93 (d, *J* = 7.8 Hz, 1H, pyrene-C3-H), 7.80 (br s, 1H, C=C(NH)CCl_3), 7.13-7.31 (m, 10H, Aryl-H), 5.89 (t, *J* = 5.1 Hz, 1H, CONH), 5.16 (d, *J* = 5.4 Hz, 2H, CONHC$\underline{H_2}$), 2.33 (s, 3H, CH_3CO); ^{13}C NMR (75 MHz, $CDCl_3$): δ 187.3 ($CH_3\underline{C}O$), 164.5 (C=$\underline{C}$(NH)CCl_3), 162.3 (CONH), 131.8 (B-Ph(*o*)), 131.6, 131.4, 130.9, 130.0, 129.2, 128.5, 127.9, 127.9, 127.6 (B-Ph(*m*)), 127.5, 127.1 (B-Ph(*o*)), 126.4, 125.8, 125.6, 125.2, 125.0, 124.9, 123.0, 105.3 ($\underline{C}$=C(NH)CCl_3), 92.9 (CCl_3), 42.9 ($CH_3CO\underline{C}H_2$), 23.6 ($\underline{C}H_3CO$), B-Ph(*i*) quaternary signal not observed; IR: 3320 (N-H), 3288 (N-H), 1630 (C=O), 1597 (C=C, conjugated), 1521 (C=C-NH) cm^{-1}; MS(ES$^-$) (*m/z*): 620.2 [M-H, ^{10}B, ^{35}Cl, ^{35}Cl, ^{35}Cl, 20%]$^-$, 621.2 [M-H, ^{11}B, ^{35}Cl, ^{35}Cl, ^{35}Cl, 98%]$^-$, 622.2 [M-H, ^{10}B, ^{35}Cl, ^{35}Cl, ^{37}Cl, 47%]$^-$, 623.2 [M-H, ^{11}B, ^{35}Cl, ^{35}Cl, ^{37}Cl, 100%]$^-$, 624.2 [M-H, ^{10}B, ^{35}Cl, ^{37}Cl, ^{37}Cl, 33%]$^-$, 625.2 [M-H, ^{11}B, ^{35}Cl, ^{37}Cl, ^{37}Cl, 19%]$^-$, 626.2 [M-H, ^{10}B, ^{37}Cl, ^{37}Cl, ^{37}Cl, 9%]$^-$, 627.3 [M-H, ^{11}B, ^{37}Cl, ^{37}Cl, ^{37}C, 4%]$^-$; MS(ES$^+$) (*m/z*): 622.2 [M+H, ^{10}B, ^{35}Cl, ^{35}Cl, ^{35}Cl, 11%]$^+$, 623.2 [M+H, ^{11}B, ^{35}Cl, ^{35}Cl, ^{35}Cl, 69%]$^+$, 624.2 [M+H, ^{10}B, ^{35}Cl, ^{35}Cl, ^{37}Cl, 30%]$^+$, 625.2 [M+H, ^{11}B, ^{35}Cl, ^{35}Cl, ^{37}Cl, 100%]$^+$, 626.2 [M+H, ^{10}B, ^{35}Cl, ^{37}Cl, ^{37}Cl, 36%]$^+$, 627.2 [M+H, ^{11}B, ^{35}Cl, ^{37}Cl, ^{37}Cl, 28%]$^+$, 628.2 [M+H, ^{10}B, ^{37}Cl, ^{37}Cl, ^{37}Cl, 5%]$^+$, 629.2 [M+H, ^{11}B, ^{37}Cl, ^{37}Cl, ^{37}Cl, 3%]$^+$.

HRMS(ES^-) (*m*/*z*): $[M-H]^-$ calcd. for $C_{35}H_{25}{}^{11}B^{35}Cl_3N_2O_2$, 621.1080; found, 621.1072, error: 1.3 ppm.

***N*-Pyridin-4-ylacetoacetamide (3i)**. Yield: 57%, cream solid. mp: 92-93^oC; ^1H NMR (300 MHz, $CDCl_3$): δ 9.61 (br s, 1H, NH), 8.53 (d, *J* = 6.0 Hz, 2H, Py-*m*), 7.52 (d, *J* = 6.0 Hz, 2H, Py-*o*), 3.64 (s, 2H, $CH_3CO\underline{C}\underline{H}_2$), 2.36 (s, 3H, CH_3CO); ^{13}C NMR (75 MHz, $CDCl_3$): δ 204.7 ($CH_3\underline{C}O$), 164.4 (CONH), 150.6 (Py(*m*)), 144.7 (Py(*i*)), 114.0 (Py(*o*)), 49.8 ($CH_3CO\underline{C}H_2$), 31.2 ($\underline{C}H_3CO$); IR: 3245 (N-H), 1721 (C=O), 1690 (C=O), 1595 (C=O) cm^{-1}; MS(ES^-) (*m*/*z*): 176.9 $[M-H, 100\%]^-$; MS(ES^+) (*m*/*z*): 178.9 [M+H, $100\%]^+$, 200.9 $[M+Na, 11\%]^+$. HRMS(ES^+) (*m*/*z*): $[M+H]^+$ calcd. for $C_9H_{11}N_2O_2$, 179.0815; found, 179.0814, error: 0.6 ppm.

(*Z*)-2-Acetyl-3-amino-4,4,4-trichloro-*N*-(pyridine-4-yl)but-2-enamide (4i). Yield: 64%, cream solid. mp: 105-107 ^oC (dec); ^1H NMR (300 MHz, $CDCl_3$): δ 8.55 (d, *J* = 6.0 Hz, 2H, Py-*m*), 7.92 (br s, 1H, NH), 7.51 (d, *J* = 6.0 Hz, 2H, Py-*o*), 2.37 (s, 3H, CH_3CO), NH_2 signal not observed; ^{13}C NMR (75 MHz, $CDCl_3$): δ 166.3 ($C=\underline{C}(NH_2)CCl_3$), 157.6 (CONH), 150.9 (Py-*m*), 144.9 (Py-*i*), 113.4 (Py-*o*), 104.7 ($\underline{C}=C(NH_2)CCl_3$), 93.2 ($CCl_3$), 28.9 ($\underline{C}H_3CO$), $CH_3\underline{C}O$ quaternary signal not observed; IR: 3230 (N-H), 1654 (C=O), 1585 (C=C, conjugated), 1506 ($C=C-NH_2$) cm^{-1}; MS(ES^-) (*m*/*z*): 319.9 $[M-H, {}^{35}Cl, {}^{35}Cl, {}^{35}Cl, 72\%]^-$, 321.9 $[M-H, {}^{35}Cl, {}^{35}Cl, {}^{37}Cl, 100\%]^-$, 323.9 $[M-H, {}^{35}Cl, {}^{37}Cl, {}^{37}Cl, 25\%]^-$, 326.0 $[M-H, {}^{37}Cl, {}^{37}Cl, {}^{37}Cl, 3\%]^-$; MS($ES^+$) (*m*/*z*): 322.0 $[M+H, {}^{35}Cl, {}^{35}Cl, {}^{35}Cl, 84\%]^+$, 324.0 $[M+H, {}^{35}Cl, {}^{35}Cl, {}^{37}Cl, 100\%]^+$, 326.0 $[M+H, {}^{35}Cl, {}^{37}Cl, {}^{37}Cl, 70\%]^+$, 328.0 $[M+H, {}^{37}Cl, {}^{37}Cl, {}^{37}Cl, 5\%]^+$. HRMS($APCI^+$) (*m*/*z*): $[M+H]^+$ calcd. for $C_{11}H_{11}{}^{35}Cl_3N_3O_2$, 321.9911; found, 321.9910, error: 0.3 ppm.

5-Acetyl-2,2-diphenyl-6-(pyridine-4-ylamino)-4-(trichloromethyl)-2,3-dihydro-1,3,2-oxazaborinin-1-ium-2-uide (NBC22). Ethyl acetate/*n*-hexane, 3:5. Yield: 56%,

yellow solid. mp: 124-126 ^{o}C (dec); ^{1}H NMR (300 MHz, $CDCl_3$): δ 8.49 (br s, 2H, Py-*m*), 8.01 (br s, 1H, NH), 7.66 (br s, 1H, NH), 7.21-7.47 (m, 12H, Py-*o* & Aryl-H), 2.43 (s, 3H, CH_3CO); IR: 3337 (N-H), 3247 (N-H), 1684 (C=O), 1586 (C=C, conjugated), 1508 (C=C-NH) cm^{-1}; MS(ES^{-}) (*m/z*): 482.9 [M-H, ^{10}B, ^{35}Cl, ^{35}Cl, ^{35}Cl, 5%]$^{-}$, 483.9 [M-H, ^{11}B, ^{35}Cl, ^{35}Cl, ^{35}Cl, 21%]$^{-}$, 485.0 [M-H, ^{10}B, ^{35}Cl, ^{35}Cl, ^{37}Cl, 7%]$^{-}$, 486.1 [M-H, ^{11}B, ^{35}Cl, ^{35}Cl, ^{37}Cl, 22%]$^{-}$, 487.2 [M-H, ^{10}B, ^{35}Cl, ^{37}Cl, ^{37}Cl, 6%]$^{-}$, 488.2 [M-H, ^{11}B, ^{35}Cl, ^{37}Cl, ^{37}Cl, 5%]$^{-}$, 489.2 [M-H, ^{10}B, ^{37}Cl, ^{37}Cl, ^{37}Cl, 3%]$^{-}$, 490.2 [M-H, ^{11}B, ^{37}Cl, ^{37}Cl, ^{37}C, 1%]$^{-}$; MS(ES^{+}) (*m/z*): 485.1 [M+H, ^{10}B, ^{35}Cl, ^{35}Cl, ^{35}Cl, 4%]$^{+}$, 486.1 [M+H, ^{11}B, ^{35}Cl, ^{35}Cl, ^{35}Cl, 26%]$^{+}$, 487.1 [M+H, ^{10}B, ^{35}Cl, ^{35}Cl, ^{37}Cl, 11%]$^{+}$, 488.1 [M+H, ^{11}B, ^{35}Cl, ^{35}Cl, ^{37}Cl, 22%]$^{+}$, 489.1 [M+H, ^{10}B, ^{35}Cl, ^{37}Cl, ^{37}Cl, 3%]$^{+}$, 490.1 [M+H, ^{11}B, ^{35}Cl, ^{37}Cl, ^{37}Cl, 7%]$^{+}$, 491.1 [M+H, ^{10}B, ^{37}Cl, ^{37}Cl, ^{37}Cl, 2%]$^{+}$, 492.1 [M+H, ^{11}B, ^{37}Cl, ^{37}Cl, ^{37}Cl, 1%]$^{+}$. HRMS(APCI^{+}) (*m/z*): [M+H]$^{+}$ calcd. for $C_{23}H_{20}{}^{11}B^{35}Cl_3N_3O_2$, 486.0709; found, 486.0709, error: 0.0 ppm.

3-(4-(2-((3-Oxobutanoyl)oxy)ethyl)piperazino)-1-propanesulfonic acid (3j). The reaction mixture was purified by flash column chromatography. MeOH/DCM, 1:3. Yield: 75%, orange solid. mp: 107-109 ^{o}C; ^{1}H NMR (300 MHz, DMSO-d_6): δ 9.75 (br s, 1H, OH), 4.16 (t, *J* = 5.4 Hz, 2H, $OC\underline{H_2}CH_2N$), 3.61 (s, 2H, $CH_3COC\underline{H_2}$), 3.24-3.55 (m, 4H, Piperazine-H_{eq}), 2.76-3.09 (m, 4H, Piperazine-H_{ax}), 2.63 (t, *J* = 5.9 Hz, 4H, $OCH_2C\underline{H_2}N$ & $NCH_2CH_2C\underline{H_2}SO_3H$), 2.21-2.45 (m, 2H, $NC\underline{H_2}CH_2CH_2SO_3H$), 2.18 (s, 3H, CH_3CO), 1.97 (p, *J* = 6.3 Hz, 2H, $NCH_2C\underline{H_2}CH_2SO_3H$); ^{13}C NMR (75 MHz, DMSO-d_6): δ 201.5 ($CH_3\underline{C}O$), 167.2 ($\underline{C}OOCH_2$), 61.7 ($O\underline{C}H_2CH_2N$), 55.7, 54.9, 51.1, 49.7, 49.6, 49.1, 30.1 ($\underline{C}H_3CO$), 19.8 ($NCH_2\underline{C}H_2CH_2SO_3H$); MS(ES^{-}) (*m/z*): 335.1 [M-H, 100%]$^{-}$; MS(ES^{+}) (*m/z*): 337.1 [M+H, 23%]$^{+}$, 359.2 [M+Na, 100%]$^{+}$, 695.4 [2M+Na, 72%]$^{+}$; HRMS(ES^{+}) (*m/z*): [M+Na]$^{+}$ calcd. for $C_{13}H_{24}N_2O_6SNa$, 359.1247; found, 359.1243, error: 1.2 ppm.

(*Z*)-3-(4-(2-((2-Acetyl-3-amino-4,4,4-trichlorobut-2-enoyl)oxy)ethyl)piperazino)-1-propanesulfonic acid (4j). Anhydrous MeOH (5 ml) was used as solvent. Yield: 88%, orange solid. mp: 69-71 ^{o}C; ^{1}H NMR (300 MHz, DMSO-d_6): δ 9.79 (br s, 1H, OH), 4.21 (t, *J* = 5.3 Hz, 2H, $OC\underline{H_2}CH_2N$), 3.47 (br s, 2H, Piperazine-H_{eq}), 3.17 (br s, 2H, Piperazine-H_{ax}), 3.02 (br s, 2H, Piperazine-H_{eq}), 2.87 (br s, 2H, Piperazine-H_{ax}), 2.56-2.76 (m, 4H, $OCH_2C\underline{H_2}N$ & $NCH_2CH_2C\underline{H_2}SO_3H$), 2.23-2.40 (m, 2H, $NC\underline{H_2}CH_2CH_2SO_3H$), 2.21 (s, 3H, CH_3CO), 1.98 (br s, 2H, $NCH_2C\underline{H_2}CH_2SO_3H$); ^{13}C NMR (75 MHz, DMSO-d_6): δ 195.4 ($CH_3\underline{C}O$), 167.9 ($C=\underline{C}(NH_2)CCl_3$), 158.9 (COO), 100.2 ($\underline{C}=C(NH_2)CCl_3$), 92.4 ($CCl_3$), 61.9 ($O\underline{C}H_2CH_2N$), 55.7, 54.6, 51.0, 49.6, 49.2, 29.0 ($\underline{C}H_3CO$), 19.6 ($NCH_2\underline{C}H_2CH_2SO_3H$); MS(ES$^-$) (*m/z*): 436.0 [M-$C_2H_3O$, ^{35}Cl, ^{35}Cl, ^{35}Cl, 60%]$^-$, 438.0 [M-C_2H_3O, ^{35}Cl, ^{35}Cl, ^{37}Cl, 69%]$^-$, 440.0 [M-C_2H_3O, ^{35}Cl, ^{37}Cl, ^{37}Cl, 16%]$^-$, 442.1 [M-C_2H_3O, ^{37}Cl, ^{37}Cl, ^{37}Cl, 12%]$^-$, 478.1 [M-H, ^{35}Cl, ^{35}Cl, ^{35}Cl, 100%]$^-$, 480.0 [M-H, ^{35}Cl, ^{35}Cl, ^{37}Cl, 53%]$^-$, 482.1 [M-H, ^{35}Cl, ^{37}Cl, ^{37}Cl, 18%]$^-$, 484.0 [M-H, ^{37}Cl, ^{37}Cl, ^{37}Cl, 2%]$^-$; MS(ES$^+$) (*m/z*): 480.0 [M+H, ^{35}Cl, ^{35}Cl, ^{35}Cl, 19%]$^+$, 482.0 [M+H, ^{35}Cl, ^{35}Cl, ^{37}Cl, 15%]$^+$, 484.0 [M+H, ^{35}Cl, ^{37}Cl, ^{37}Cl, 4%]$^+$, 486.0 [M+H, ^{37}Cl, ^{37}Cl, ^{37}Cl, 2%]$^+$, 502.1 [M+Na, ^{35}Cl, ^{35}Cl, ^{35}Cl, 73%]$^+$, 504.1 [M+Na, ^{35}Cl, ^{35}Cl, ^{37}Cl, 100%]$^+$, 506.0 [M+Na, ^{35}Cl, ^{37}Cl, ^{37}Cl, 39%]$^+$, 508.0 [M+Na, ^{37}Cl, ^{37}Cl, ^{37}Cl, 3%]$^+$; HRMS(ES$^+$) (*m/z*): [M+Na]$^+$ calcd. for $C_{15}H_{24}{}^{35}Cl_3N_3O_6SNa$, 502.0344; found, 502.0343, error: 0.2 ppm.

6-Methyl-2,2-diphenyl-5-((2-(4-(3-sulfopropyl)piperazin-1-yl)ethoxy)carbonyl)-4(trichloromethyl)-2,3-dihydro-1,3,2-oxazaborinin-1-ium-2-uide (NBC-EPPS). MeOH/DCM, 3:5. Yield: 56%, yellow solid. mp: 104-106 ^{o}C (dec), exists with minor impurity that could not be separated; ^{1}H NMR (300 MHz, DMSO-d_6): 10.82 (br s, 1H, NH), 9.83 (br s, 1H, OH), 7.13-7.35 (m, 10H, Aryl-H), 4.14 (br s, 2H, $OC\underline{H_2}CH_2N$), 3.53-3.65 (m, minor), 3.38-3.47 (m, 2H, Piperazine-H), 3.12-3.25 (m, 2H, Piperazine-

H), 2.74-2.98 (m, 4H, Piperazine-H), 2.55-2.72 (m, 4H, $OCH_2C\underline{H}_2N$ & $NCH_2CH_2C\underline{H}_2SO_3H$), 2.44 (s, 3H, CH_3CO), 2.25 (m, 2H, $NC\underline{H}_2CH_2CH_2SO_3H$), 1.90-2.07 (m, 2H, $NCH_2C\underline{H}_2CH_2SO_3H$), 1.71-1.80 (m, minor); ^{13}C NMR (75 MHz, DMSO-d_6): 185.8 ($CH_3\underline{C}O$), 164.8, 164.7, 131.9 (B-Ph(*o*)), 126.9 (B-Ph(*m*)), 126.3 (B-Ph(*p*)), 101.5 ($\underline{C}$=C(NH)CCl_3), 92.0 (CCl_3), 62.4 ($O\underline{C}H_2CH_2N$), 55.7, 54.3, 50.9, 49.6, 49.2, 23.4 ($\underline{C}H_3CO$), 19.6 ($NCH_2\underline{C}H_2CH_2SO_3H$), B-Ph(*i*) quaternary signal not observed; IR: 3350 (N-H), 1654 (C=O), 1596 (C=C, conjugated), 1520 (C=C-NH) cm^{-1}; MS(ES^-) (*m/z*): 608.2 [M-^{35}Cl, ^{10}B, ^{35}Cl, ^{35}Cl, 13%]$^-$, 609.2 [M-^{35}Cl, ^{11}B, ^{35}Cl, ^{35}Cl, 50%]$^-$, 610.2 [M-^{35}Cl, ^{10}B, ^{35}Cl, ^{37}Cl, 32%]$^-$, 611.2 [M-^{35}Cl, ^{11}B, ^{35}Cl, ^{37}Cl, 34%]$^-$, 612.2 [M-^{35}Cl, ^{10}B, ^{37}Cl, ^{37}Cl, 13%]$^-$, 613.3 [M-^{35}Cl, ^{11}B, ^{37}Cl, ^{37}Cl, 5%]$^-$, 642.2 [M-H, ^{10}B, ^{35}Cl, ^{35}Cl, ^{35}Cl, 8%]$^-$, 643.2 [M-H, ^{11}B, ^{35}Cl, ^{35}Cl, ^{35}Cl, 48%]$^-$, 644.2 [M-H, ^{10}B, ^{35}Cl, ^{35}Cl, ^{37}Cl, 51%]$^-$, 645.2 [M-H, ^{11}B, ^{35}Cl, ^{35}Cl, ^{37}Cl, 100%]$^-$, 646.2 [M-H, ^{10}B, ^{35}Cl, ^{37}Cl, ^{37}Cl, 58%]$^-$, 647.2 [M-H, ^{11}B, ^{35}Cl, ^{37}Cl, ^{37}Cl, 50%]$^-$, 648.3 [M-H, ^{10}B, ^{37}Cl, ^{37}Cl, ^{37}Cl, 22%]$^-$, 649.2 [M-H, ^{11}B, ^{37}Cl, ^{37}Cl, ^{37}C, 11%]$^-$; MS(ES^+) (*m/z*): 479.1 [M-BPh_2, ^{35}C, ^{35}Cl, ^{35}Cl, 100%]$^+$, 631.2 [M-^{35}Cl+Na, ^{10}B, ^{35}Cl, ^{35}Cl, 1%]$^+$, 632.2 [M-^{35}Cl+Na, ^{11}B, ^{35}Cl, ^{35}Cl, 9%]$^+$, 633.3 [M-^{35}Cl+Na, ^{10}B, ^{35}Cl, ^{37}Cl, 13%]$^+$, 634.2 [M-^{35}Cl, ^{11}B, ^{35}Cl, ^{37}Cl, 20%]$^+$, 635.2 [M-^{35}Cl+Na, ^{10}B, ^{37}Cl, ^{37}Cl, 10%]$^+$, 636.2 [M-^{35}Cl+Na, ^{11}B, ^{37}Cl, ^{37}Cl, 8%]$^+$, 665.2 [M+Na, ^{10}B, ^{35}Cl, ^{35}Cl, ^{35}Cl, 2%]$^+$, 666.2 [M+Na, ^{11}B, ^{35}Cl, ^{35}Cl, ^{35}Cl, 10%]$^+$, 667.2 [M+Na, ^{10}B, ^{35}Cl, ^{35}Cl, ^{37}Cl, 13%]$^+$, 668.2 [M+Na, ^{11}B, ^{35}Cl, ^{35}Cl, ^{37}Cl, 20%]$^+$, 669.2 [M+Na, ^{10}B, ^{35}Cl, ^{37}Cl, ^{35}Cl, 18%]$^+$, 670.2 [M+Na, ^{11}B, ^{35}Cl, ^{37}Cl, ^{37}Cl, 30%]$^+$, 671.2 [M+Na, ^{10}B, ^{37}Cl, ^{37}Cl, ^{37}Cl, 13%]$^+$, 672.2 [M+Na, ^{11}B, ^{37}Cl, ^{37}Cl, ^{37}Cl, 11%]$^+$. HRMS($APCI^+$) (*m/z*): $[M+Na]^+$ calcd. for $C_{27}H_{33}{}^{11}B^{35}Cl_3N_3O_6SNa$, 666.1141; found, 666.1140, error: 0.2 ppm.

1.5 General Procedure for Diazaborine Reaction

An appropriate amine (2.5 mmol) was added to 6-methyl-2,2-diphenyl-4-(trichloromethyl)-2,3-dihydro-1,3,2-oxazaborinin-1-ium-2-uide (**3b**, 0.82 mmol) in anhydrous THF (total reaction volume of 5 ml). The mixture was stirred at 50°C under N_2 for 24 h, unless otherwise stated. The reaction mixture was concentrated and purified by flash column chromatography to give the corresponding diazaborine product (Vasilev et al., 2013).

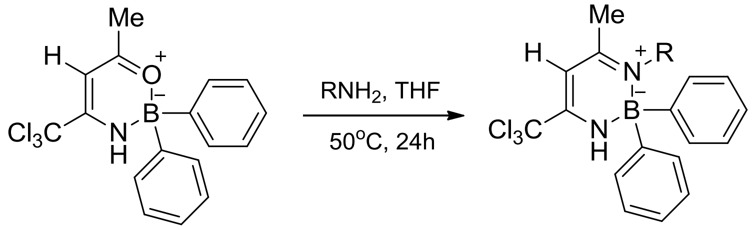

BC23

R = Me **NBC27**
R = Et **NBC30**
R = Pr **NBC31**

Scheme 6 | Synthesis of NBC27 and NBC30-31.

1,6-Dimethyl-2,2-diphenyl-4-(trichloromethyl)-2,3-dihydro-1,3,2-diazaborinin-1-ium-2-uide (NBC27, 18). Ethyl acetate/*n*-hexane, 1:9. Yield: 90%, yellow solid. mp: 117-118 ^{o}C; ^{1}H NMR (300 MHz, $CDCl_3$): δ 7.33 (d, *J* = 6.9 Hz, 4H, B-Ph(*o*)), 7.12-7.27 (m, 6H, B-Ph(*m*/*p*)), 5.67 (br s, 1H, Cl_3C(N<u>*H*</u>)C=CH), 5.33 (d, *J* = 2.7 Hz, 1H, Cl_3C(NH)C=C<u>*H*</u>), 2.81 (s, 3H, CH_3N), 2.10 (s, 3H, CH_3CN); ^{13}C NMR (75 MHz, $CDCl_3$): δ 167.4 (Cl_3C(NH)<u>*C*</u>=C), 158.8 (CH_3<u>*C*</u>N), 133.2 (B-Ph(*o*)), 127.3 (B-Ph(*m*)), 126.2 (B-Ph(*p*)), 94.2 (CCl_3), 89.0 (Cl_3C(NH)C=<u>*C*</u>), 37.4 (CH_3N), 21.6 (<u>*C*</u>H_3CN), B-Ph(*i*) quaternary signal not observed; ^{11}B{^{1}H} (128 MHz, $CDCl_3$): δ 0.54; IR: 3370 (N-H), 1595 (C=C, conjugated), 1529 (C=C-NH) cm^{-1}; MS(ES$^+$) (*m/z*): 300.0 [M-Ph, ^{10}B, ^{35}Cl, ^{35}Cl, ^{35}Cl, 35%]$^+$, 301.0 [M-Ph, ^{11}B, ^{35}Cl, ^{35}Cl, ^{35}Cl, 100%]$^+$, 302.0 [M-Ph, ^{10}B, ^{35}Cl, ^{35}Cl, ^{37}Cl, 32%]$^+$, 303.0 [M-Ph, ^{11}B, ^{35}Cl, ^{35}Cl, ^{37}Cl, 69%]$^+$, 304.0 [M-Ph, ^{10}B,

^{35}Cl, ^{37}Cl, ^{37}Cl, 30%]$^+$, 305.0 [M-Ph, ^{11}B, ^{35}Cl, ^{37}Cl, ^{37}Cl, 27%]$^+$, 306.0 [M-Ph, ^{10}B, ^{37}Cl, ^{37}Cl, ^{37}Cl, 3%]$^+$, 307.0 [M-Ph, ^{11}B, ^{37}Cl, ^{37}Cl, ^{37}Cl, 4%]$^+$, 378.0 [M+H, ^{10}B, ^{35}Cl, ^{35}Cl, ^{35}Cl, 2%]$^+$, 379.0 [M+H, ^{11}B, ^{35}Cl, ^{35}Cl, ^{35}Cl, 11%]$^+$, 380.0 [M+H, ^{10}B, ^{35}Cl, ^{35}Cl, ^{37}Cl, 3%]$^+$, 381.0 [M+H, ^{11}B, ^{35}Cl, ^{35}Cl, ^{37}Cl, 10%]$^+$, 382.0 [M+H, ^{10}B, ^{35}Cl, ^{37}Cl, ^{37}Cl, 4%]$^+$, 383.0 [M+H, ^{11}B, ^{35}Cl, ^{37}Cl, ^{37}Cl, 5%]$^+$, 384.0 [M+H, ^{10}B, ^{37}Cl, ^{37}Cl, ^{37}Cl, 1%]$^+$, 385.0 [M+H, ^{11}B, ^{37}Cl, ^{37}Cl, ^{37}Cl, 2%]$^+$; HRMS(ES$^+$) (*m/z*): [M+H]$^+$ calcd. for $C_{18}H_{19}{}^{11}B^{35}Cl_3N_2$, 379.0701; found, 379.0699, error: 0.5 ppm.

1-Ethyl-6-methyl-2,2-diphenyl-4-(trichloromethyl)-2,3-dihydro-1,3,2-diazaborinin-1-ium-2-uide (NBC30, 19). Ethyl acetate/*n*-hexane, 1:24. Yield: 89%, yellow solid. mp: 104-106 ^{o}C (dec); ^{1}H NMR (300 MHz, $CDCl_3$): δ 7.40 (d, *J* = 6.6 Hz, 4H, B-Ph(*o*)), 7.21-7.35 (m, 6H, B-Ph(*m*/*p*)), 5.89 (br s, 1H, Cl_3C(N$\underline{H}$)C=CH), 5.42 (d, *J* = 3.0 Hz, 1H, Cl_3C(NH)C=C$\underline{H}$), 3.40 (q, *J* = 7.1 Hz, 2H, $CH_3C\underline{H_2}N$), 2.29 (s, 3H, CH_3N), 0.69 (t, *J* = 7.2 Hz, $C\underline{H_3}CH_2N$); ^{13}C NMR (75 MHz, $CDCl_3$): δ 166.8 (Cl_3C(NH)$\underline{C}$=C), 158.5 ($CH_3\underline{C}N$), 133.3 (B-Ph(*o*)), 127.1 (B-Ph(*m*)), 126.2 (B-Ph(*p*)), 94.2 (CCl_3), 89.5 (Cl_3C(NH)C=$\underline{C}$), 44.4 ($CH_3\underline{C}H_2N$), 20.8 (CH_3N), 14.3 ($\underline{C}H_3CH_2N$), B-Ph(*i*) quaternary signal not observed; ^{11}B{^{1}H} (128 MHz, $CDCl_3$): δ 0.25; IR: 3375 (N-H), 1599 (C=C, conjugated), 1523 (C=C-NH) cm^{-1}; MS(APCI$^+$) (*m/z*): 314.0 [M-Ph, ^{10}B, ^{35}Cl, ^{35}Cl, ^{35}Cl, 4%]$^+$, 315.0 [M-Ph, ^{11}B, ^{35}Cl, ^{35}Cl, ^{35}Cl, 18%]$^+$, 316.0 [M-Ph, ^{10}B, ^{35}Cl, ^{35}Cl, ^{37}Cl, 3%]$^+$, 317.0 [M-Ph, ^{11}B, ^{35}Cl, ^{35}Cl, ^{37}Cl, 17%]$^+$, 318.0 [M-Ph, ^{10}B, ^{35}Cl, ^{37}Cl, ^{37}Cl, 2%]$^+$, 319.0 [M-Ph, ^{11}B, ^{35}Cl, ^{37}Cl, ^{37}Cl, 5%]$^+$, 320.0 [M-Ph, ^{10}B, ^{37}Cl, ^{37}Cl, ^{37}Cl, 1%]$^+$, 321.0 [M-Ph, ^{11}B, ^{37}Cl, ^{37}Cl, ^{37}Cl, 1%]$^+$, 392.1 [M+H, ^{10}B, ^{35}Cl, ^{35}Cl, ^{35}Cl, 11%]$^+$, 393.1 [M+H, ^{11}B, ^{35}Cl, ^{35}Cl, ^{35}Cl, 40%]$^+$, 394.1 [M+H, ^{10}B, ^{35}Cl, ^{35}Cl, ^{37}Cl, 15%]$^+$, 395.1 [M+H, ^{11}B, ^{35}Cl, ^{35}Cl, ^{37}Cl, 39%]$^+$, 396.1 [M+H, ^{10}B, ^{35}Cl, ^{37}Cl, ^{37}Cl, 9%]$^+$, 397.1 [M+H, ^{11}B, ^{35}Cl, ^{37}Cl, ^{37}Cl, 13%]$^+$, 398.1 [M+H, ^{10}B, ^{37}Cl, ^{37}Cl, ^{37}Cl, 3%]$^+$,

399.1 [M+H, ^{11}B, ^{37}Cl, ^{37}Cl, ^{37}Cl, 1%]$^+$; HRMS(APCI$^+$) (*m*/*z*): [M+H]$^+$ calcd. for $C_{19}H_{21}{}^{11}B^{35}Cl_3N_2$, 393.0858; found, 393.0847, error: 2.8 ppm.

1-Propyl-6-methyl-2,2-diphenyl-4-(trichloromethyl)-2,3-dihydro-1,3,2-diazaborinin-1-ium-2-uide (NBC31). Ethyl acetate/*n*-hexane, 1:24. Yield: 81%, yellow solid. mp: 127-129 ^{o}C; ^{1}H NMR (300 MHz, $CDCl_3$): δ 7.39 (d, *J* = 6.6 Hz, 4H, B-Ph(*o*)), 7.20-7.35 (m, 6H, B-Ph(*m*/*p*)), 5.90 (br s, 1H, $Cl_3C(N\underline{\mathit{H}})C{=}CH$), 5.41 (d, *J* = 3.0 Hz, 1H, $Cl_3C(NH)C{=}C\underline{\mathit{H}}$), 3.18-3.28 (m, 2H, $CH_3CH_2C\underline{\mathit{H}}_2N$), 2.28 (s, 3H, CH_3N), 1.02 (sext, *J* = 6.4 Hz, 2H, $CH_3C\underline{\mathit{H}}_2CH_2N$), 0.51 (t, *J* = 7.4 Hz, $C\underline{\mathit{H}}_3CH_2CH_2N$); ^{13}C NMR (75 MHz, $CDCl_3$): δ 166.8 ($Cl_3C(NH)\underline{\mathit{C}}{=}C$), 158.5 ($CH_3\underline{\mathit{C}}N$), 133.2 (B-Ph(*o*)), 127.1 (B-Ph(*m*)), 126.2 (B-Ph(*p*)), 94.2 (CCl_3), 89.4 ($Cl_3C(NH)C{=}\underline{\mathit{C}}$), 51.7 ($CH_3CH_2\underline{\mathit{C}}H_2N$), 22.5 ($CH_3\underline{\mathit{C}}H_2CH_2N$), 20.9 ($CH_3N$), 11.2 ($\underline{\mathit{C}}H_3CH_2CH_2N$), B-Ph(*i*) quaternary signal not observed; ^{11}B{^{1}H} (128 MHz, $CDCl_3$): δ 0.43; IR: 3373 (N-H), 1603 (C=C, conjugated), 1525 (C=C-NH) cm^{-1}; MS(APCI$^+$) (*m*/*z*): 328.0 [M-Ph, ^{10}B, ^{35}Cl, ^{35}Cl, ^{35}Cl, 7%]$^+$, 329.0 [M-Ph, ^{11}B, ^{35}Cl, ^{35}Cl, ^{35}Cl, 29%]$^+$, 330.0 [M-Ph, ^{10}B, ^{35}Cl, ^{35}Cl, ^{37}Cl, 13%]$^+$, 331.0 [M-Ph, ^{11}B, ^{35}Cl, ^{35}Cl, ^{37}Cl, 31%]$^+$, 332.1 [M-Ph, ^{10}B, ^{35}Cl, ^{37}Cl, ^{37}Cl, 6%]$^+$, 333.1 [M-Ph, ^{11}B, ^{35}Cl, ^{37}Cl, ^{37}Cl, 8%]$^+$, 334.1 [M-Ph, ^{10}B, ^{37}Cl, ^{37}Cl, ^{37}Cl, 2%]$^+$, 335.1 [M-Ph, ^{11}B, ^{37}Cl, ^{37}Cl, ^{37}Cl, 1%]$^+$, 406.1 [M+H, ^{10}B, ^{35}Cl, ^{35}Cl, ^{35}Cl, 23%]$^+$, 407.1 [M+H, ^{11}B, ^{35}Cl, ^{35}Cl, ^{35}Cl, 100%]$^+$, 408.1 [M+H, ^{10}B, ^{35}Cl, ^{35}Cl, ^{37}Cl, 42%]$^+$, 409.1 [M+H, ^{11}B, ^{35}Cl, ^{35}Cl, ^{37}Cl, 90%]$^+$, 410.1 [M+H, ^{10}B, ^{35}Cl, ^{37}Cl, ^{37}Cl, 27%]$^+$, 411.1 [M+H, ^{11}B, ^{35}Cl, ^{37}Cl, ^{37}Cl, 36%]$^+$, 412.1 [M+H, ^{10}B, ^{37}Cl, ^{37}Cl, ^{37}Cl, 10%]$^+$, 413.1 [M+H, ^{11}B, ^{37}Cl, ^{37}Cl, ^{37}Cl, 3%]$^+$. HRMS(APCI$^+$) (*m*/*z*): [M+H]$^+$ calcd. for $C_{20}H_{23}{}^{11}B^{35}Cl_3N_2$, 407.1014; found, 407.1016, error: 0.5 ppm.

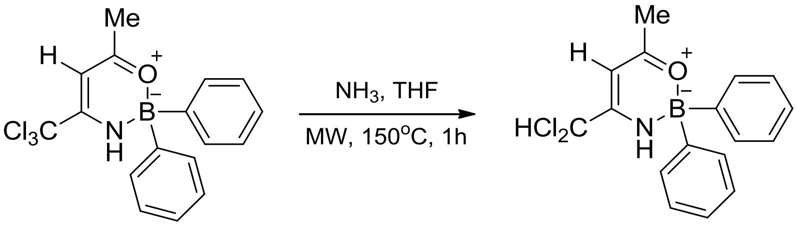

Scheme 7 | Synthesis of NBC29.

4-(Dichloromethyl)-6-Methyl-2,2-diphenyl-2,3-dihydro-1,3,2-oxazaborinin-1-ium-2-uide (NBC29). Ammonia (20.9 mg, 1.23 mmol) was added to **3b** (150 mg, 0.41 mmol) in anhydrous THF (total reaction volume of 3 ml). The mixture was subjected to microwave irradiation at 150^oC for 1h in a 5 ml sealed vial. The reaction mixture was concentrated and purified by flash column chromatography (ethyl acetate/*n*-hexane, 2:23). Yield: 92.2 mg (68%), yellow oil. ^1H NMR (300 MHz, $CDCl_3$): δ 7.13-7.34 (m, 10H, Aryl-H), 6.06 (s, 1H, CCl_2H), 5.30 (d, *J* = 2.1 Hz, 1H, $Cl_3C(NH)C{=}C\underline{H}$), 2.12 (s, 3H, CH_3CO), NH signal not observed; ^{13}C NMR (75 MHz, $CDCl_3$): δ 185.2 ($CH_3\underline{C}O$), 164.8 ($Cl_3C(NH)\underline{C}{=}C$), 131.8 (B-Ph(*o*)), 127.5 (B-Ph(*m*)), 126.8 (B-Ph(*p*)), 93.2 ($Cl_3C(NH)C{=}\underline{C}$), 67.6 ($CCl_2H$), 24.5 ($\underline{C}H_3CO$), B-Ph(*i*) quaternary signal not observed; $^{11}B\{^1H\}$ (128 MHz, $CDCl_3$): δ 4.01; IR: 3354 (N-H), 1616 (C=C, conjugated), 1539 (C=C-NH) cm^{-1}; MS(ES^-) (*m/z*): 329.0 [M-H, ^{10}B, ^{35}Cl, ^{35}Cl, 15%]$^-$, 330.0 [M-H, ^{11}B, ^{35}Cl, ^{35}Cl, 100%]$^-$, 331.0 [M-H, ^{10}B, ^{35}Cl, ^{37}Cl, 29%]$^-$, 332.0 [M-H, ^{11}B, ^{35}Cl, ^{37}Cl, 40%]$^-$, 333.0 [M-H, ^{10}B, ^{37}Cl, ^{37}Cl, 15%]$^-$, 334.0 [M-H, ^{11}B, ^{37}Cl, ^{37}Cl, 11%]$^-$; MS(ES^+) (*m/z*): 253.0 [M-Ph, ^{10}B, ^{35}Cl, ^{35}Cl, 17%]$^+$, 253.9 [M-Ph, ^{11}B, ^{35}Cl, ^{35}Cl, 100%]$^+$, 255.0 [M-Ph, ^{10}B, ^{35}Cl, ^{37}Cl, 35%]$^+$, 256.0 [M-Ph, ^{11}B, ^{35}Cl, ^{37}Cl, 69%]$^+$, 257.0 [M-Ph, ^{10}B, ^{37}Cl, ^{37}Cl, 17%]$^+$, 257.9 [M-Ph, ^{11}B, ^{37}Cl, ^{37}Cl, 11%]$^+$, 330.0 [M+H, ^{10}B, ^{35}Cl, ^{35}Cl, 3%]$^+$, 331.0 [M+H, ^{11}B, ^{35}Cl, ^{35}Cl, 12%]$^+$, 332.0 [M+H, ^{10}B, ^{35}Cl, ^{37}Cl, 10%]$^+$, 333.0 [M+H, ^{11}B, ^{35}Cl, ^{37}Cl, 6%]$^+$, 334.0 [M+H, ^{10}B, ^{37}Cl, ^{37}Cl, 7%]$^+$, 335.0

[M+H, ^{11}B, ^{37}Cl, ^{37}Cl, 1%]$^+$; HRMS(ES$^-$) (*m/z*): [M-H]$^-$ calcd. for $C_{17}H_{15}{}^{11}B{}^{35}Cl_2NO$, 330.0629; found, 330.0640, error: 3.3 ppm.

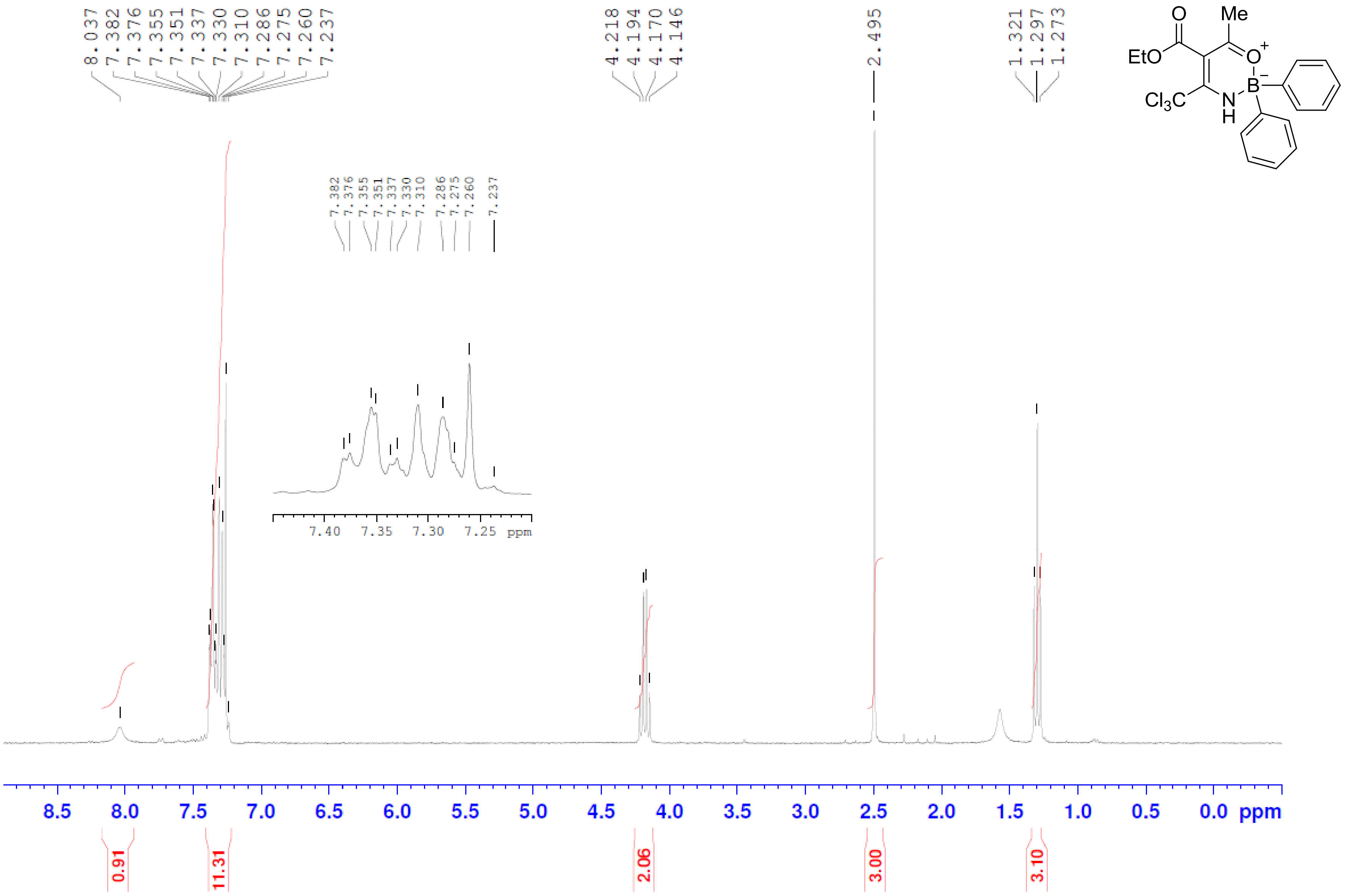

^{1}H NMR spectrum of BC-7 (**13**) in $CDCl_3$

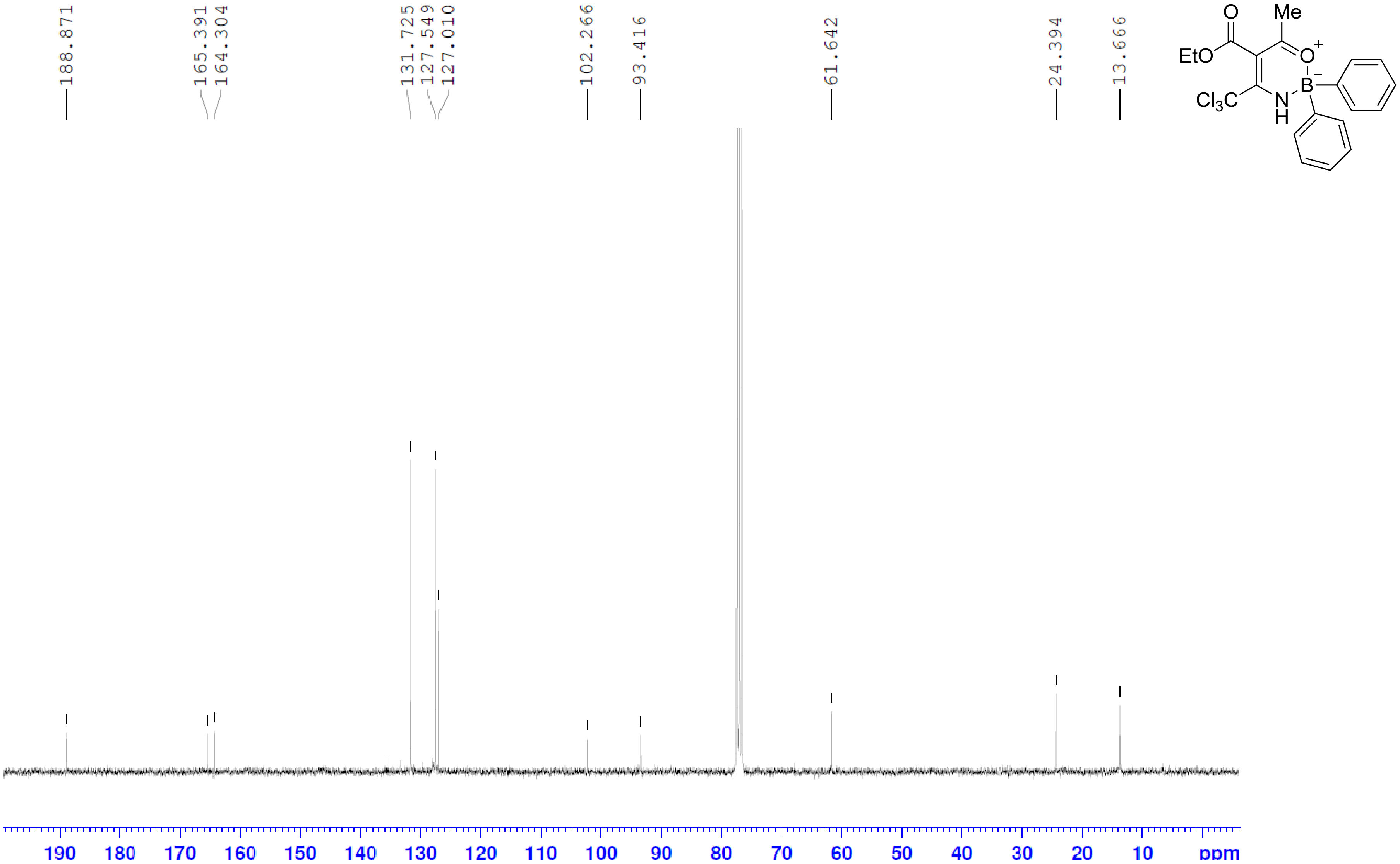

^{13}C NMR spectrum of BC-7 (**13**) in $CDCl_3$

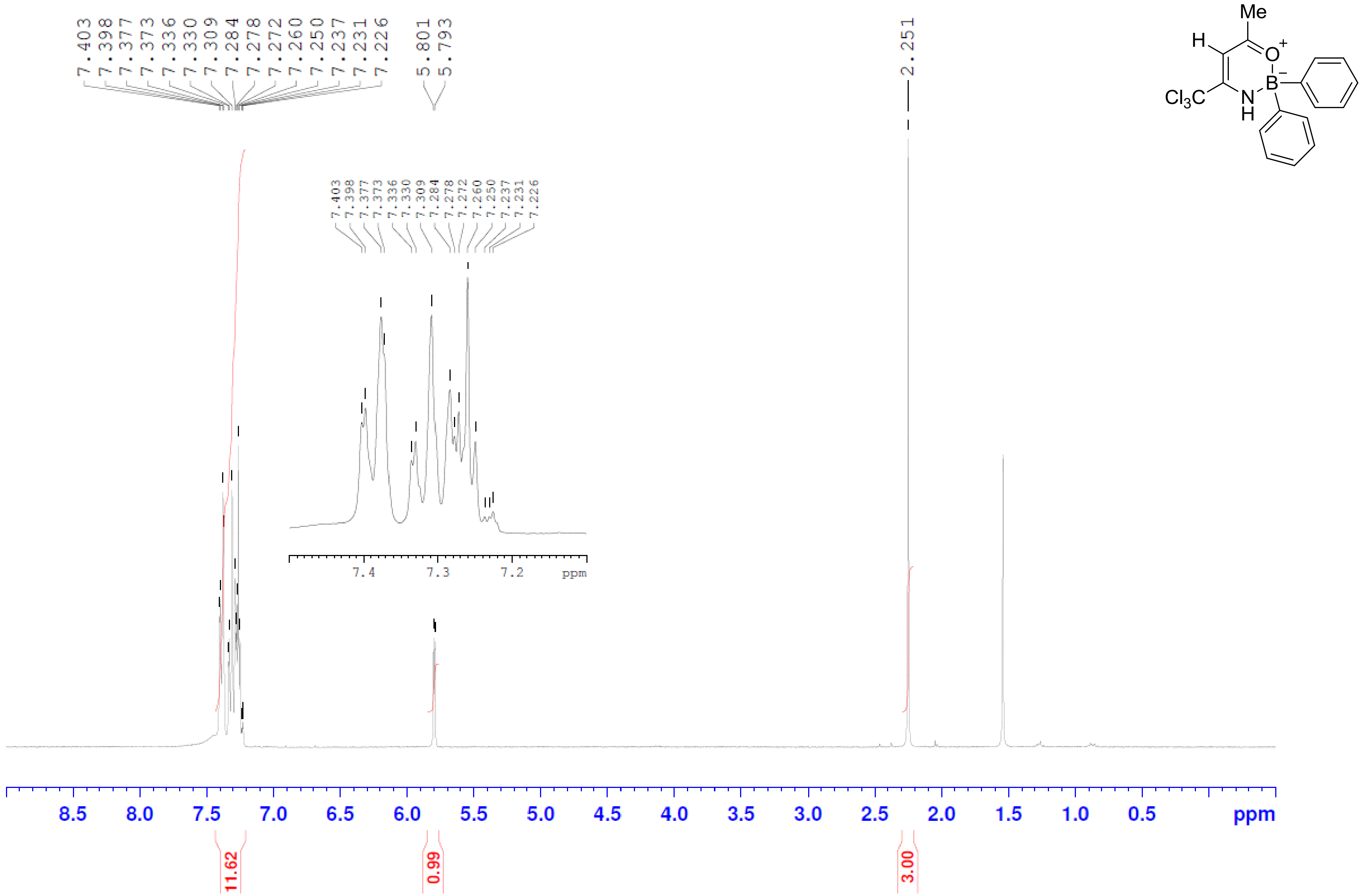

^{1}H NMR spectrum of BC-23 (**14**) in $CDCl_3$

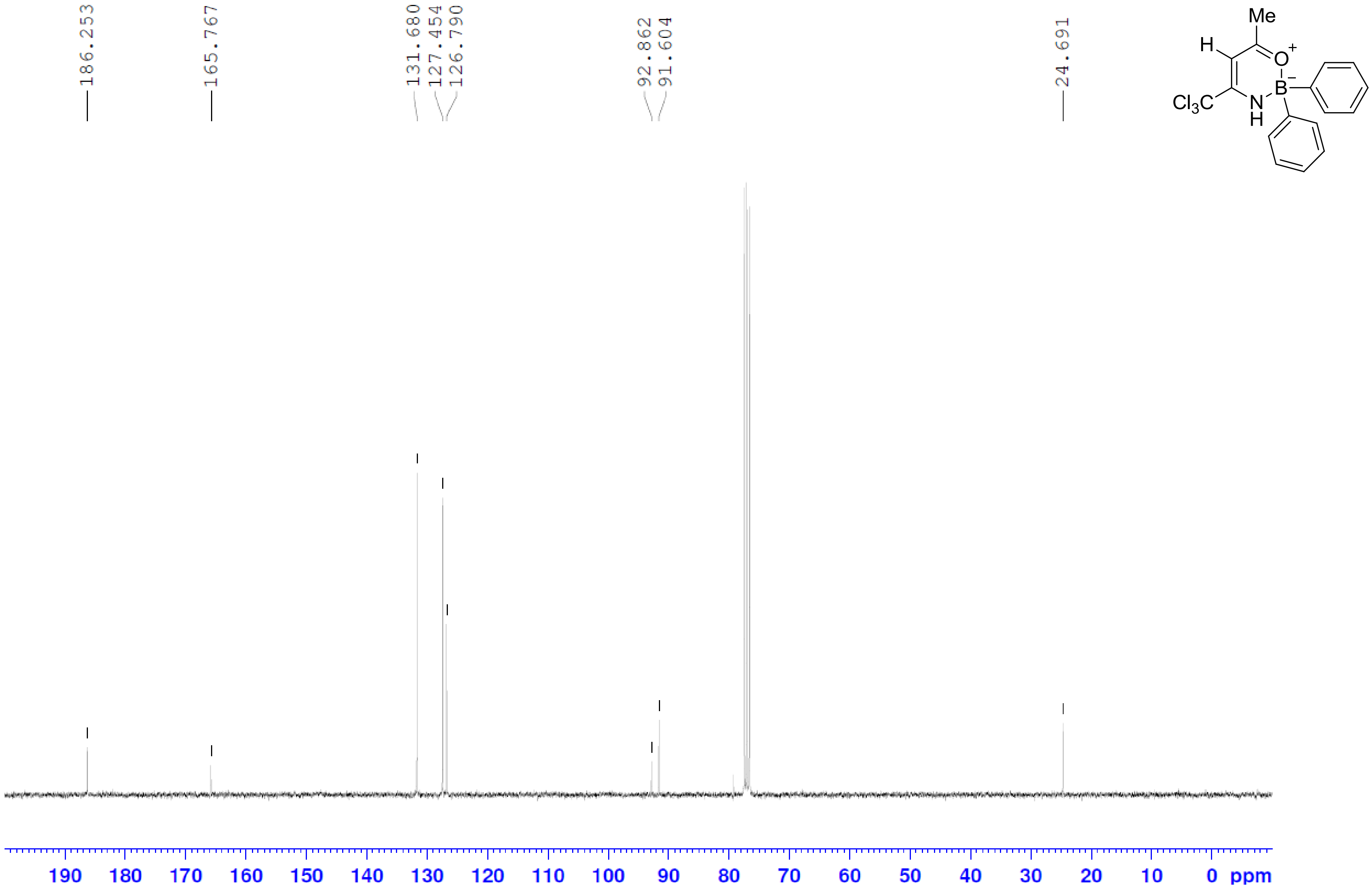

^{13}C NMR spectrum of BC-23 (**14**) in $CDCl_3$

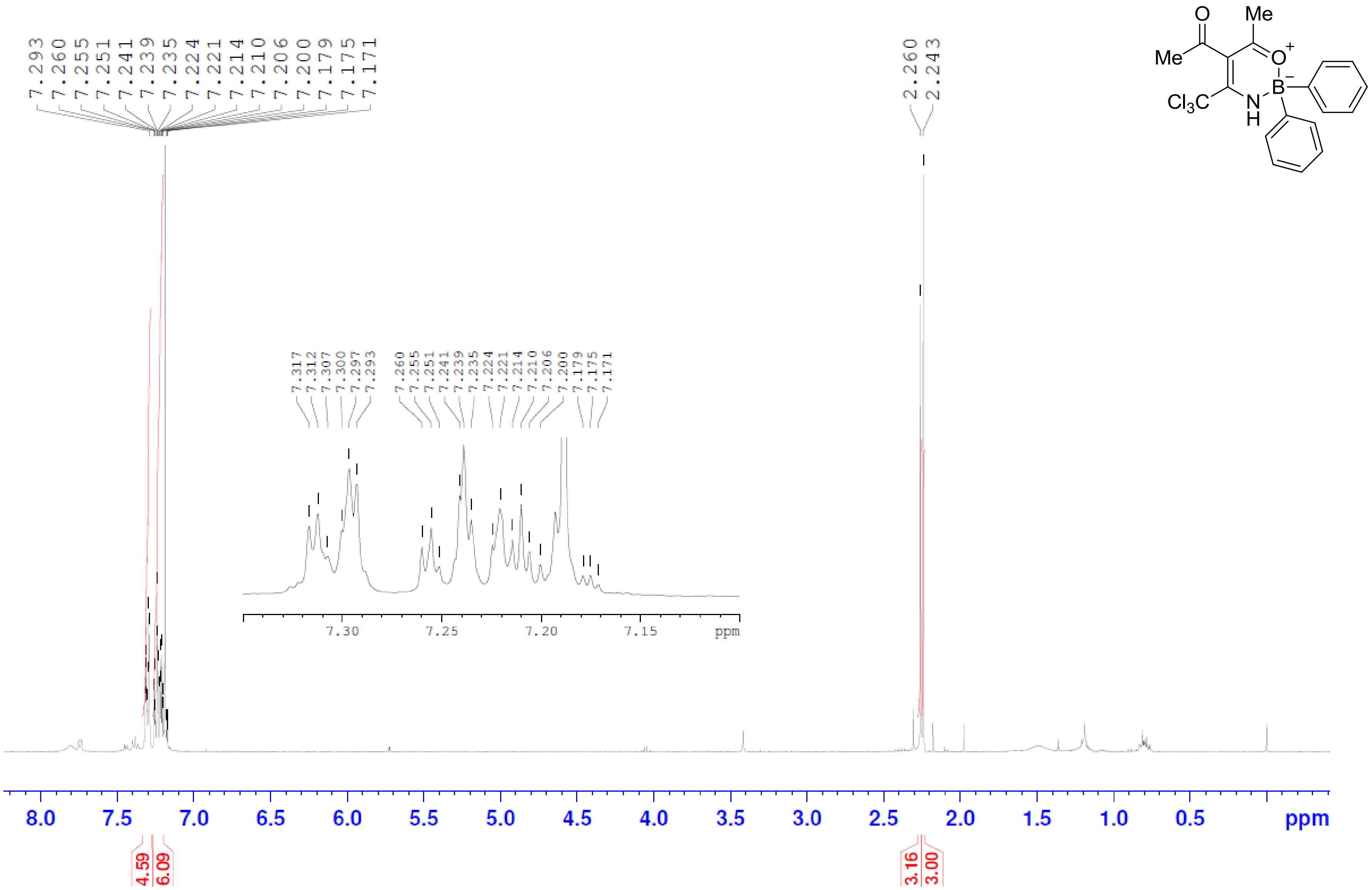

^{1}H NMR spectrum of NBC1 in $CDCl_3$

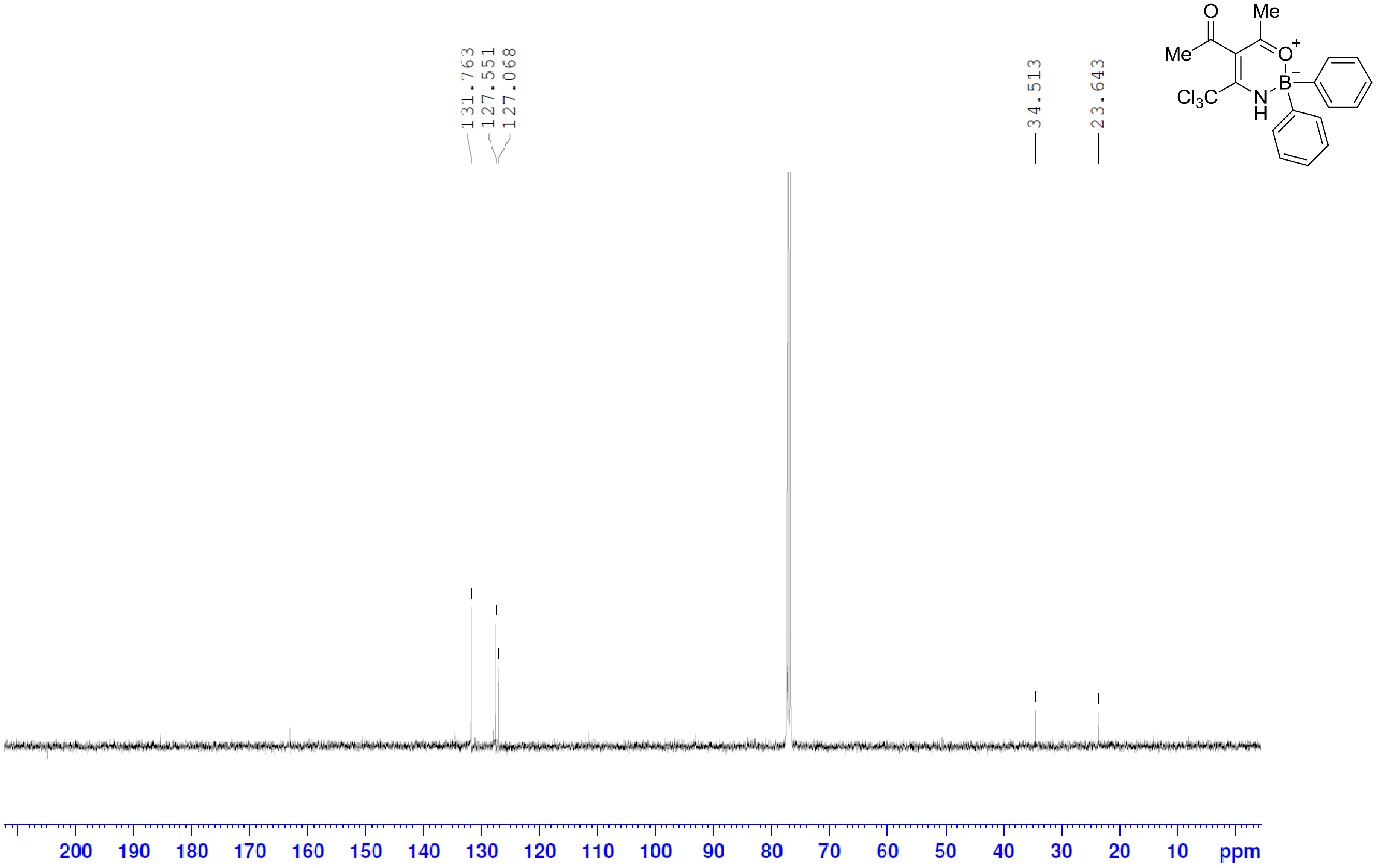

^{13}C NMR spectrum of NBC1 in $CDCl_3$

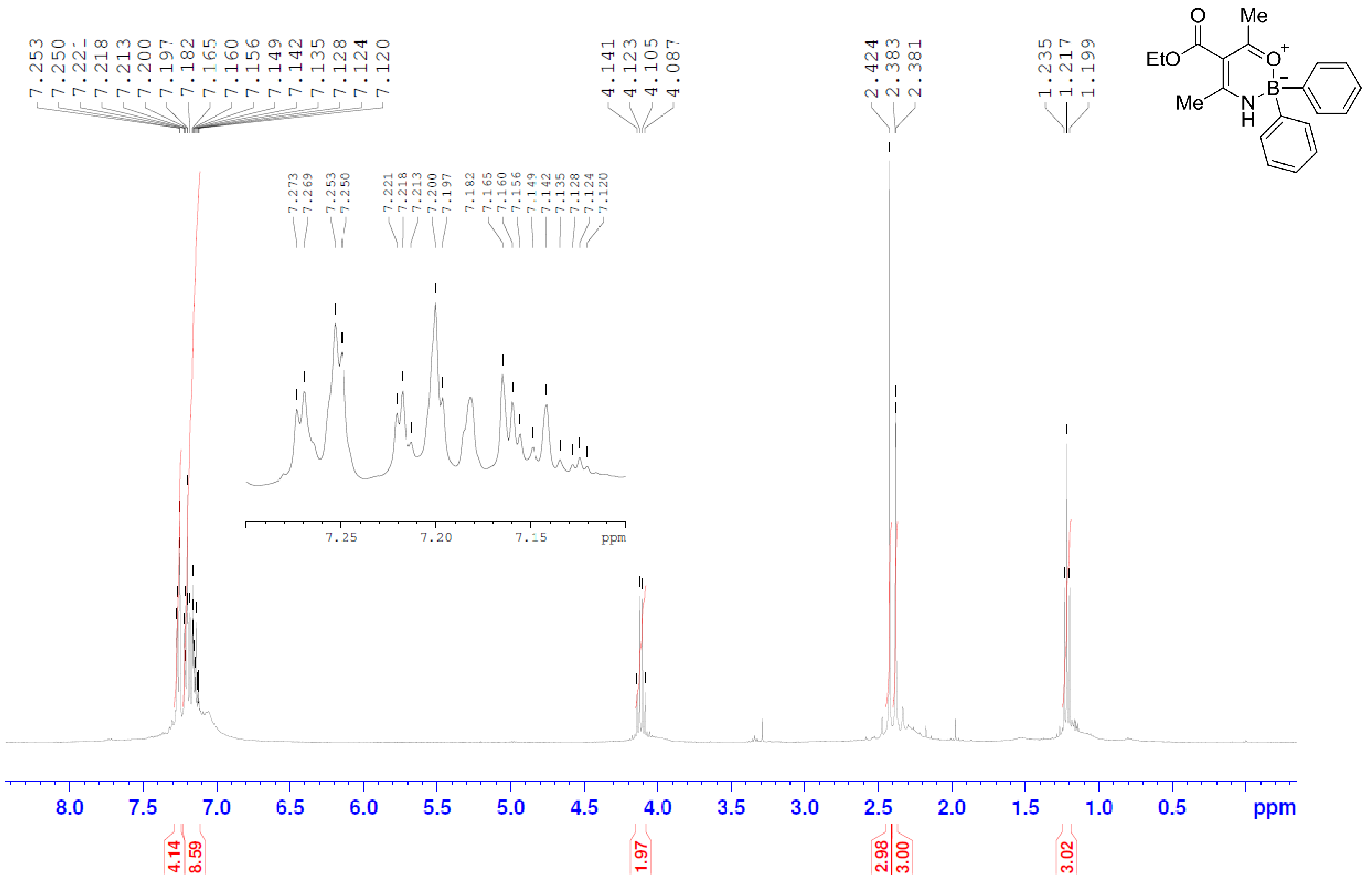

^{1}H NMR spectrum of NBC2 in $CDCl_3$

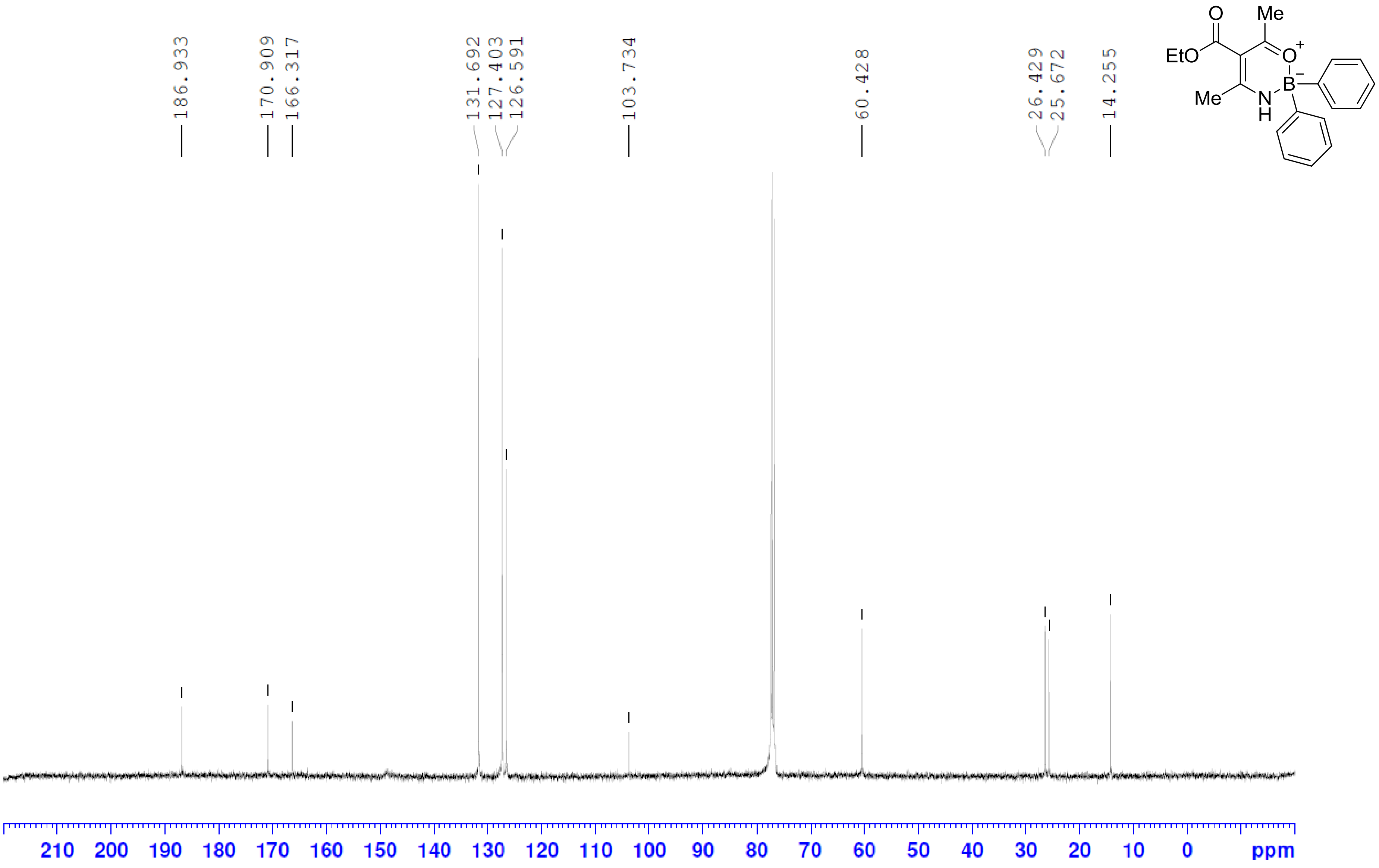

^{13}C NMR spectrum of NBC2 in $CDCl_3$

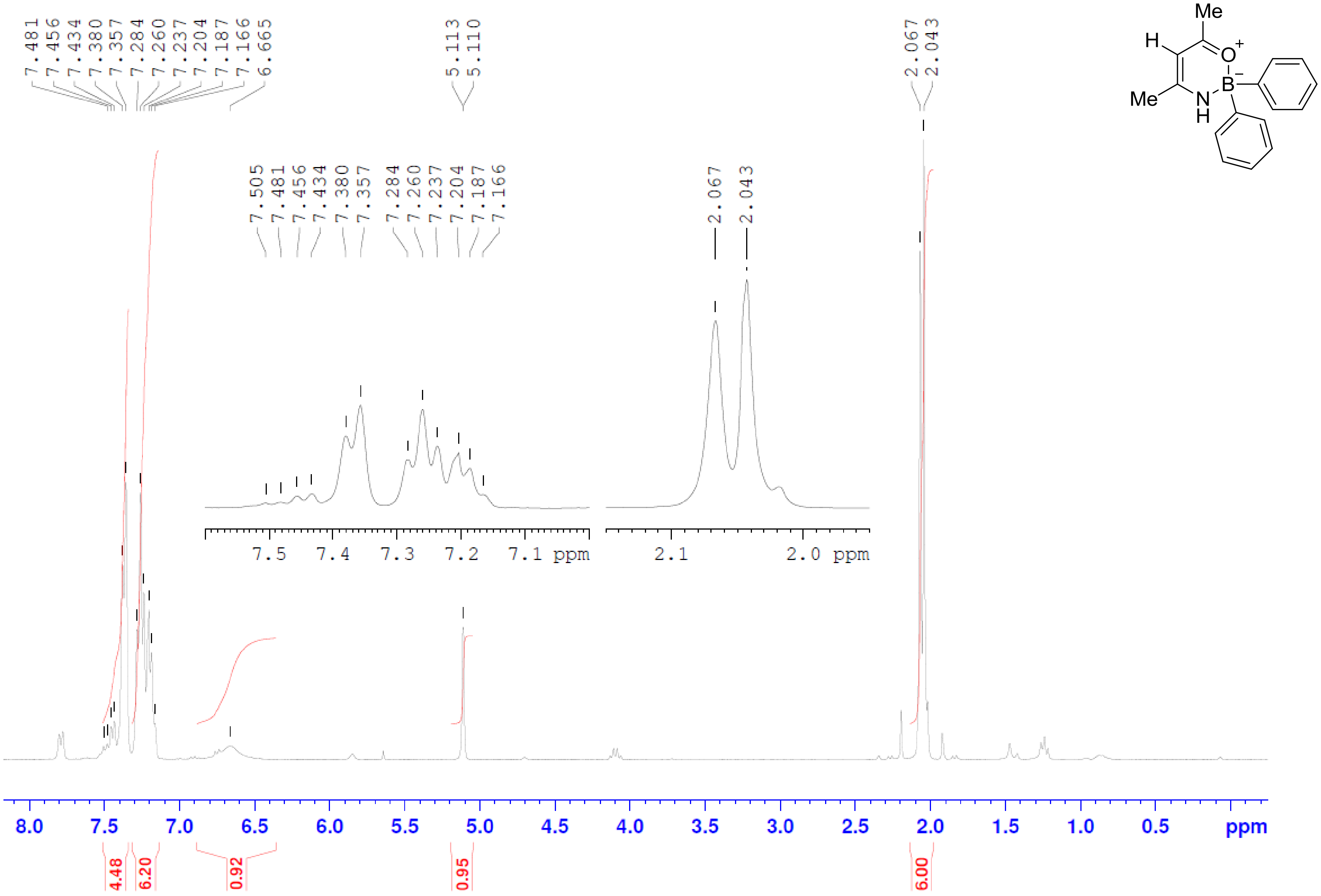

^{1}H NMR spectrum of NBC3 in $CDCl_3$

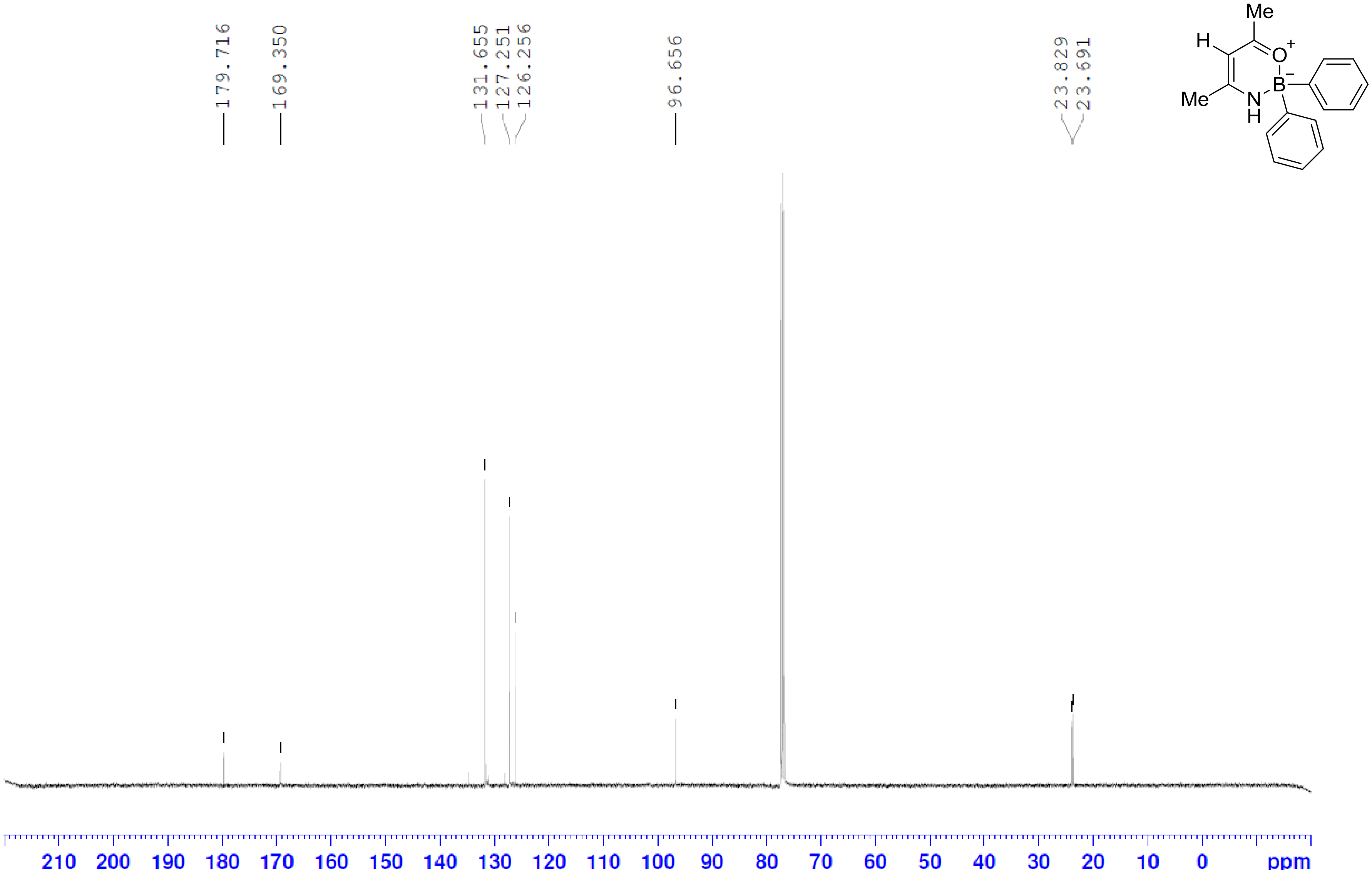

^{13}C NMR spectrum of NBC3 in $CDCl_3$

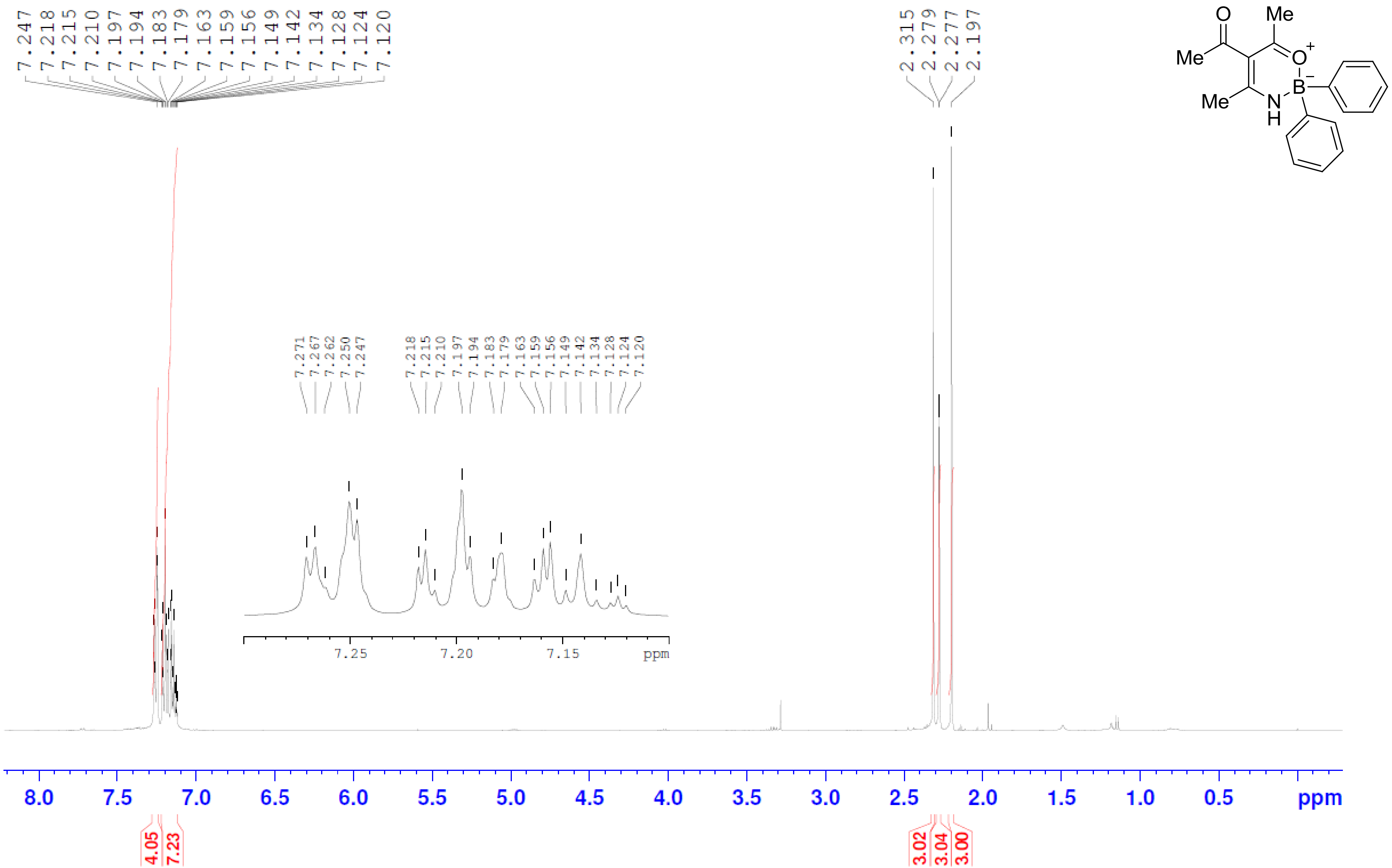

^{1}H NMR spectrum of NBC4 in $CDCl_3$

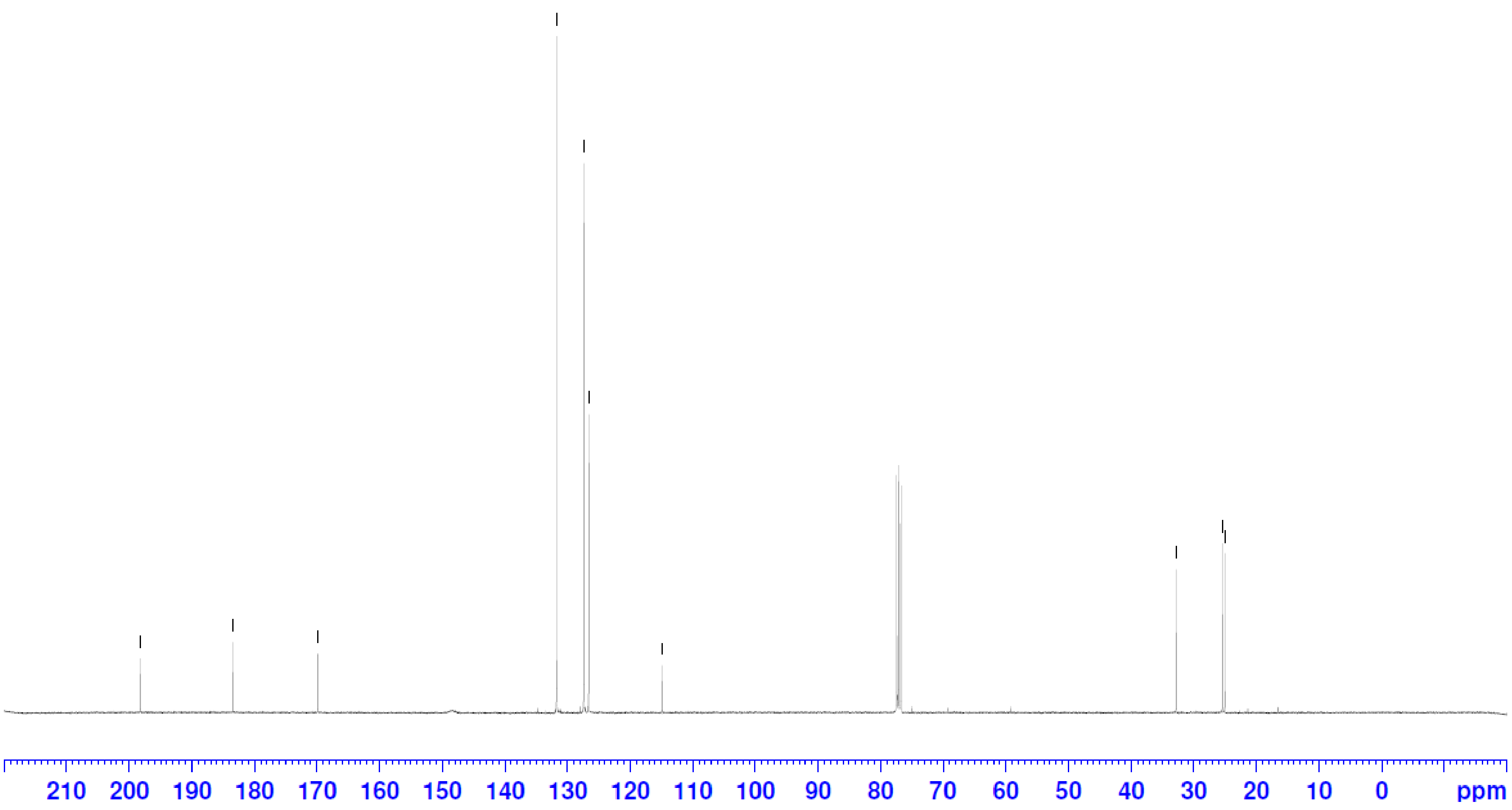

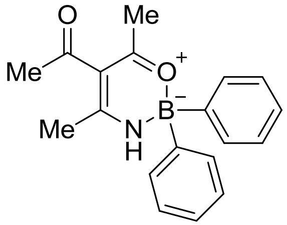

^{13}C NMR spectrum of NBC4 in $CDCl_3$

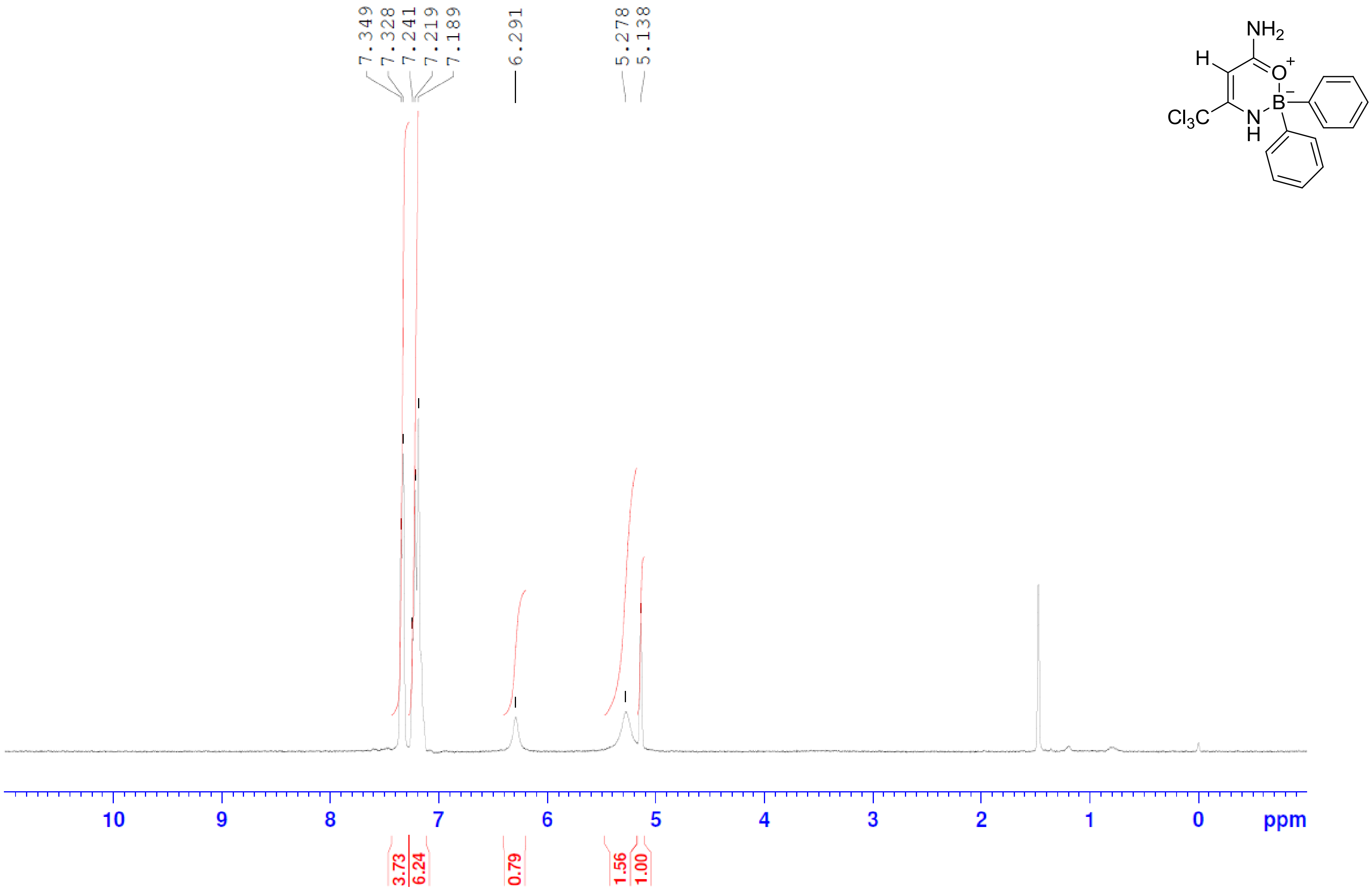

^{1}H NMR spectrum of NBC5 in $CDCl_3$

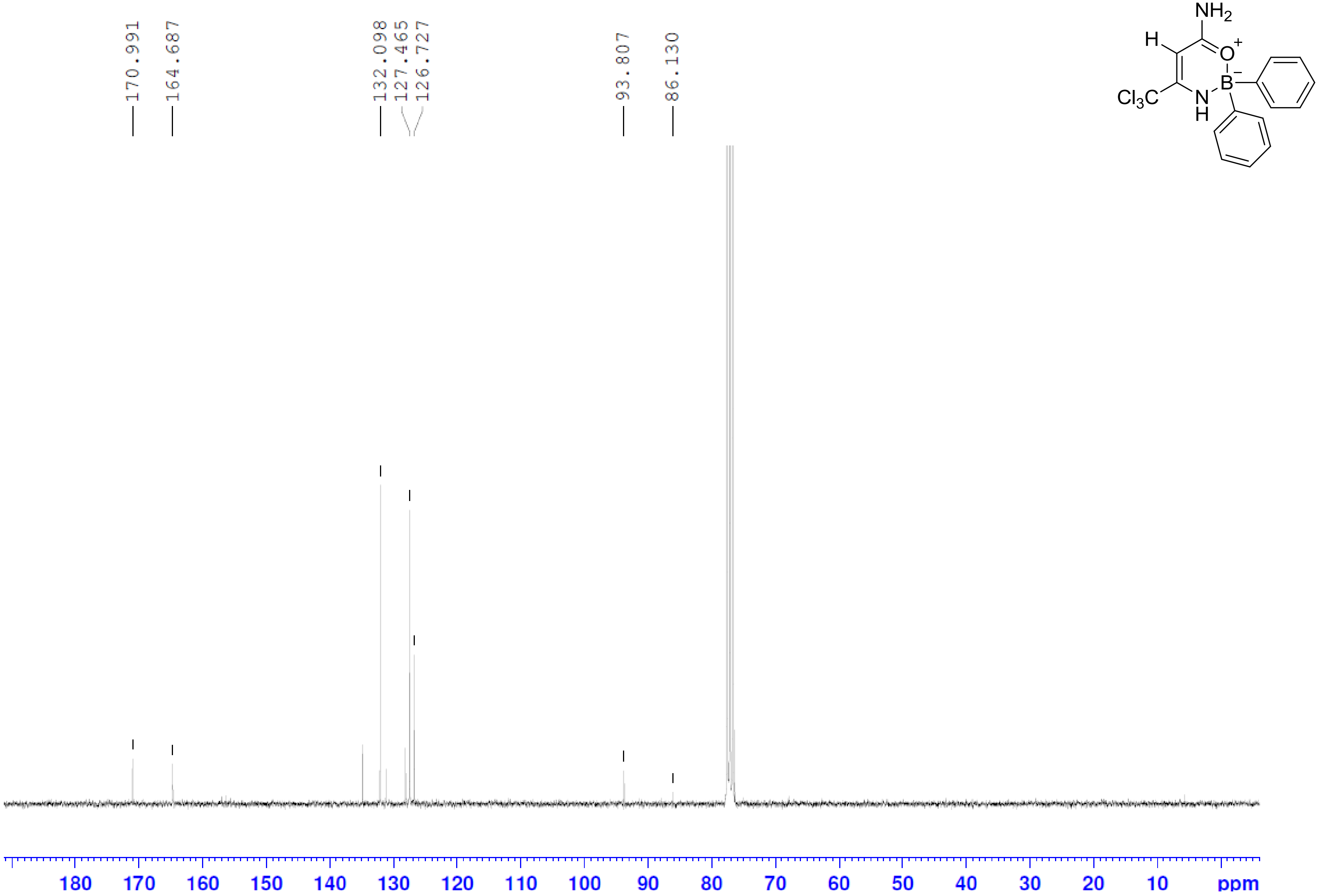

^{13}C NMR spectrum of NBC5 in $CDCl_3$ (3 x small singlet peaks ~ 127-135 ppm due to formation of BPh_2OH hydrolysis product)

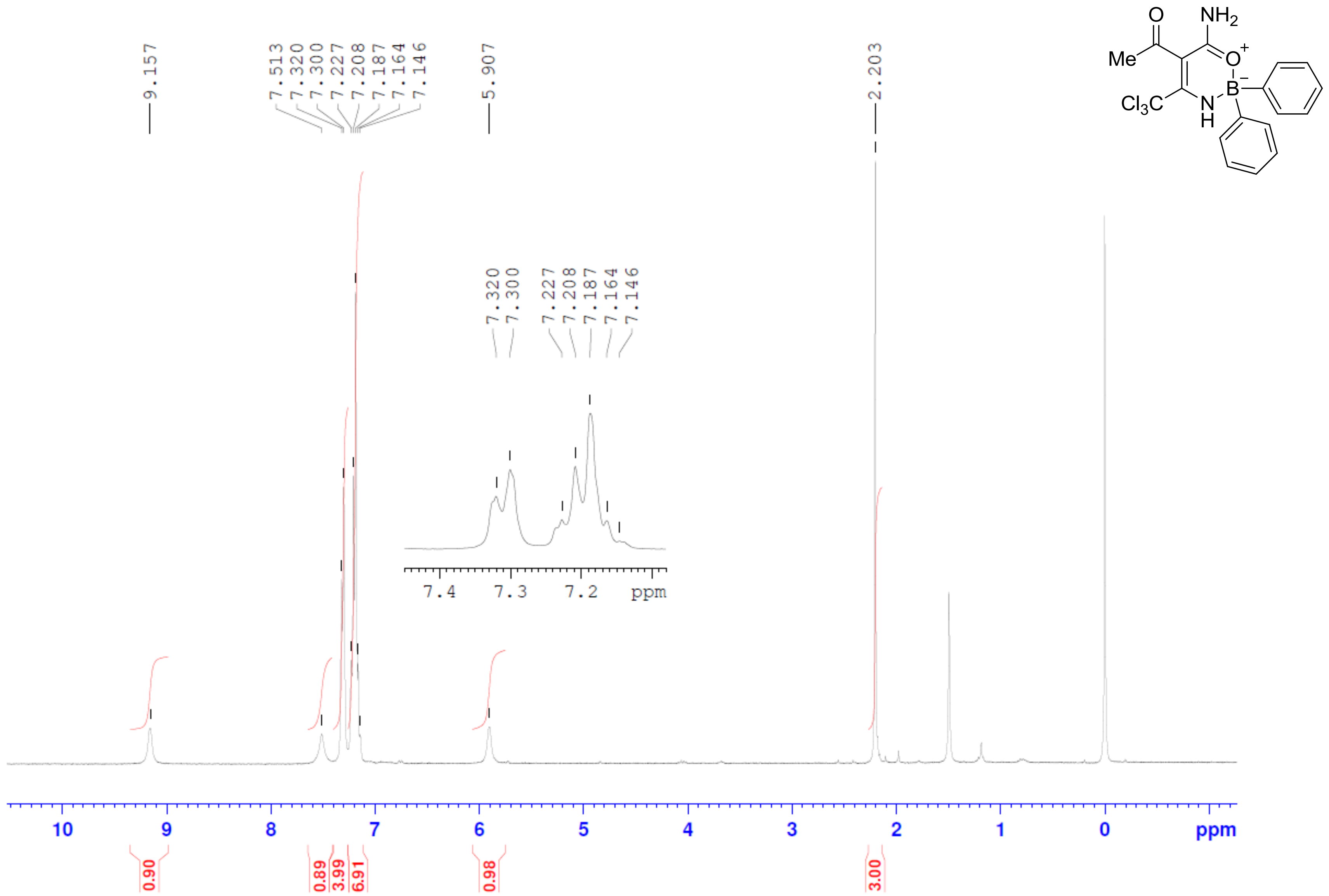

^{1}H NMR spectrum of NBC6 (**15**) in $CDCl_3$

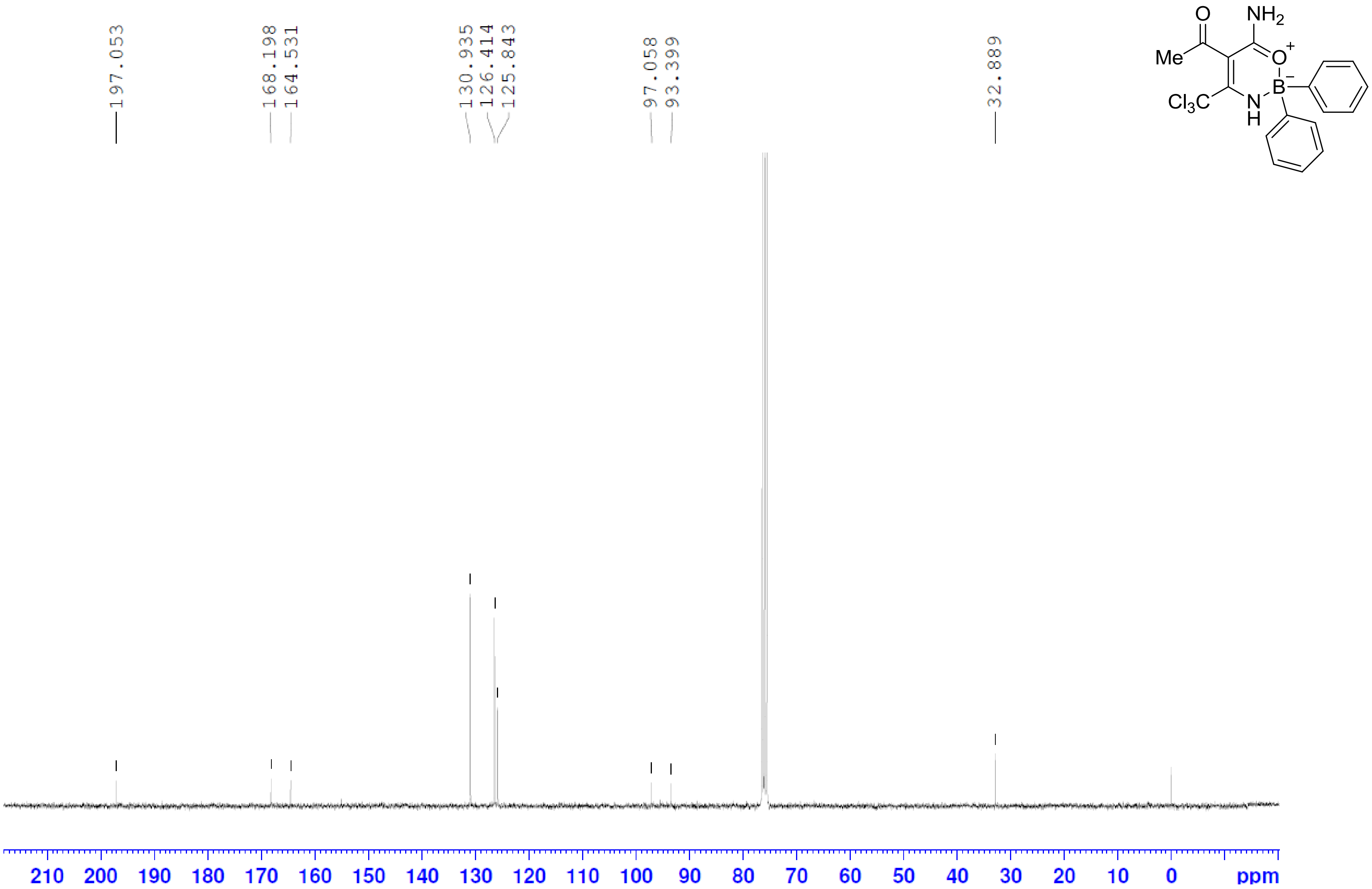

^{13}C NMR spectrum of NBC6 (**15**) in $CDCl_3$

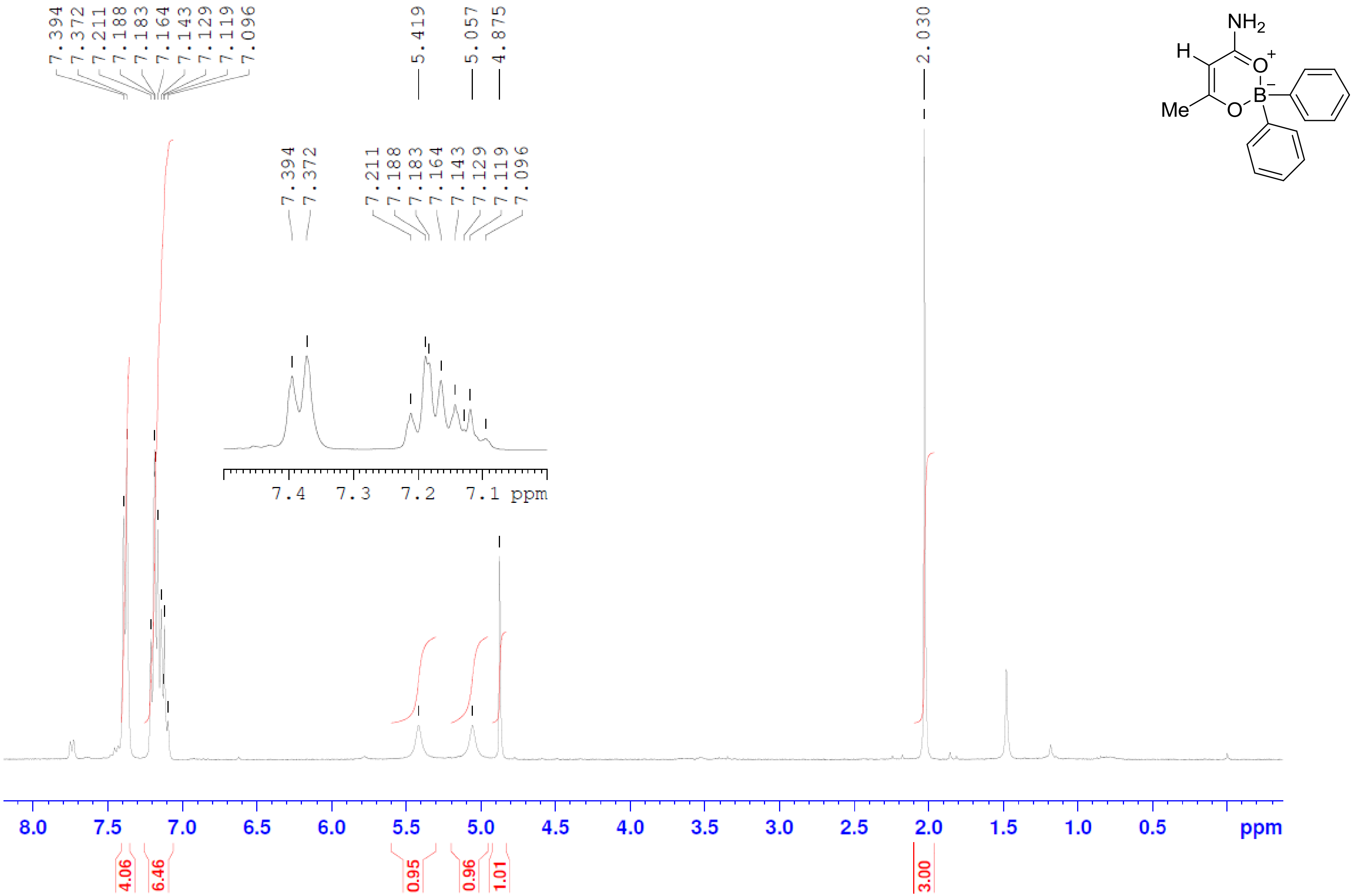

^{1}H NMR spectrum of NBC7 in $CDCl_3$

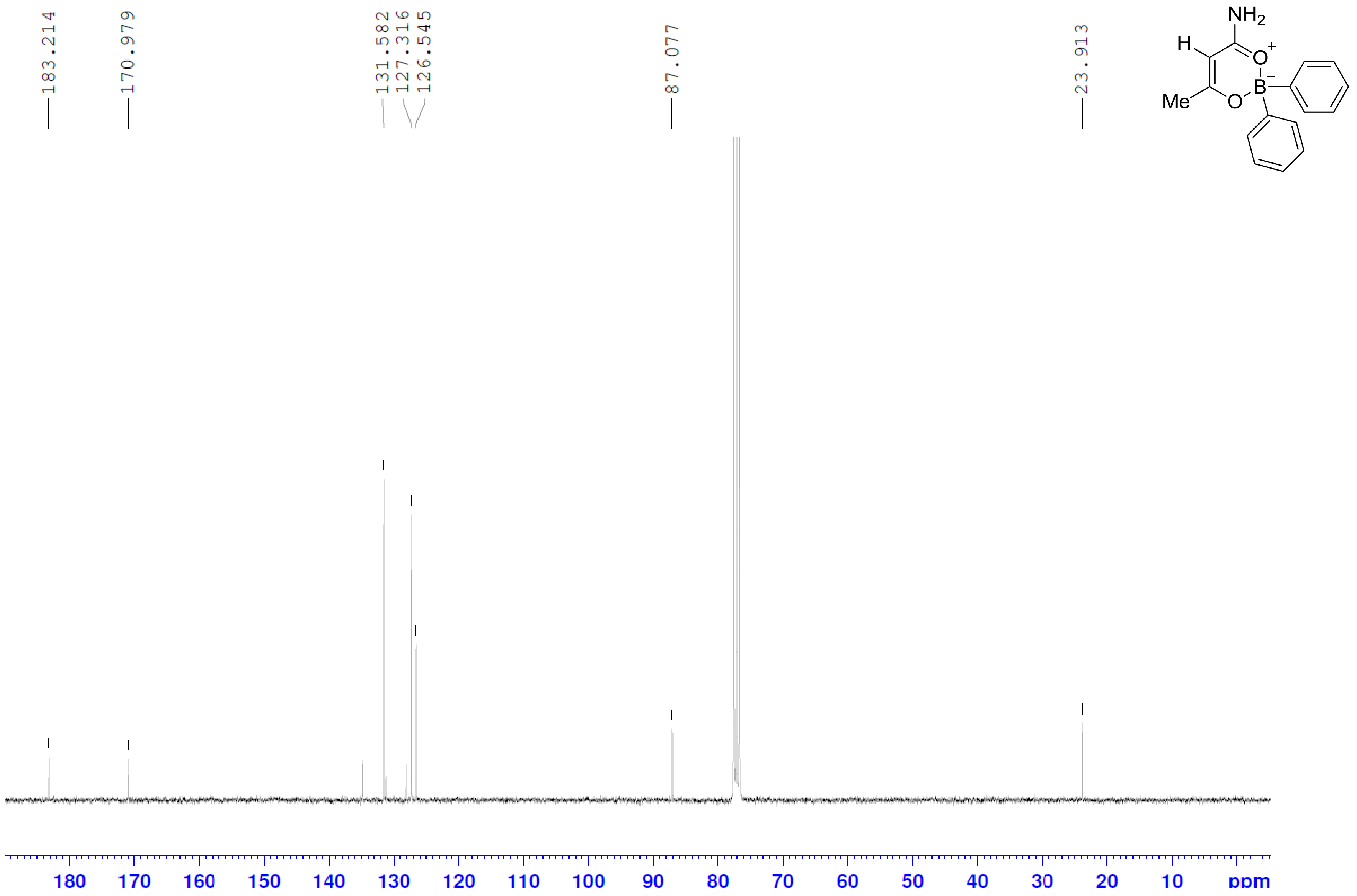

^{13}C NMR spectrum of NBC7 in $CDCl_3$

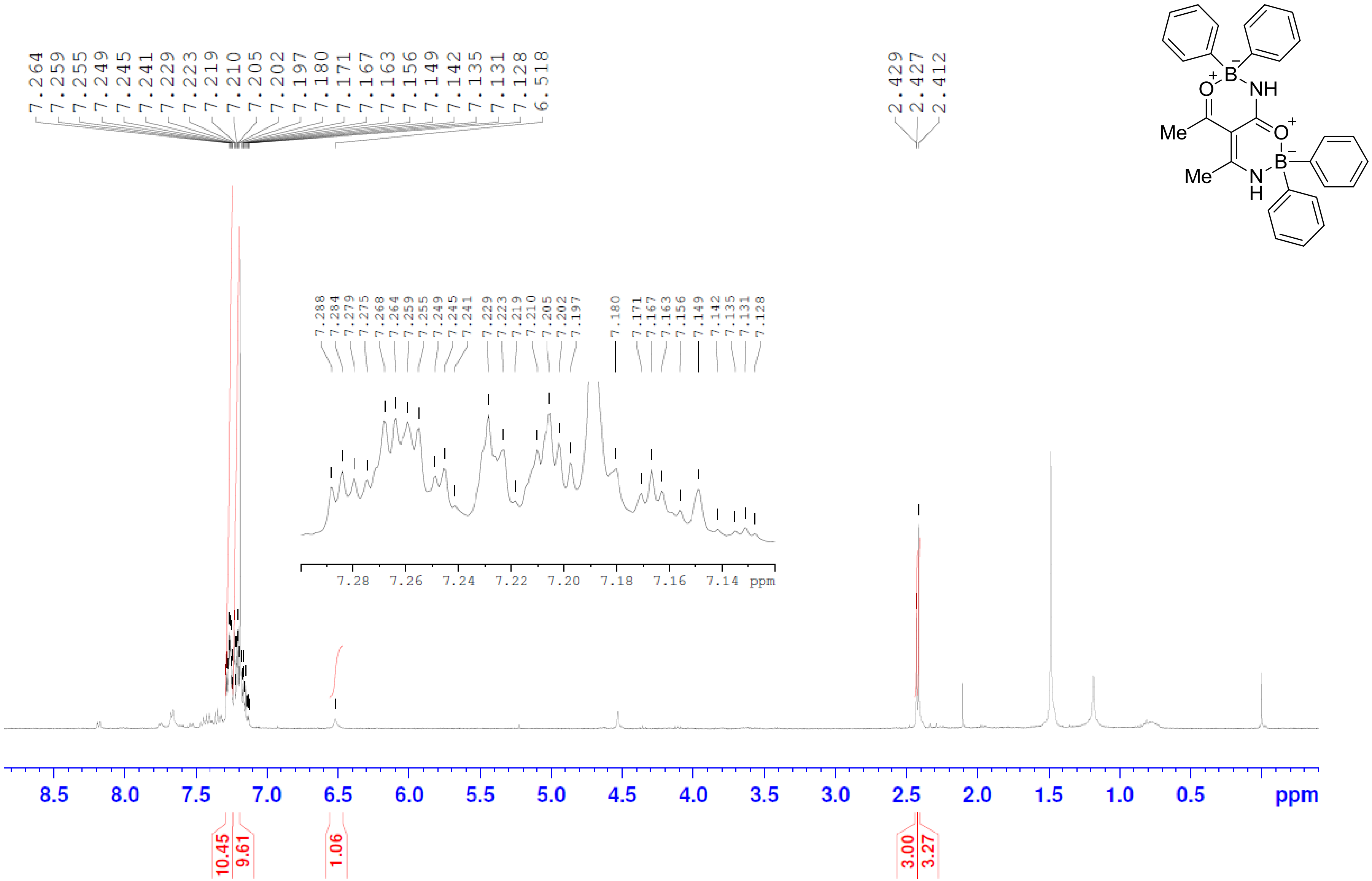

^{1}H NMR spectrum of NBC8 in $CDCl_3$

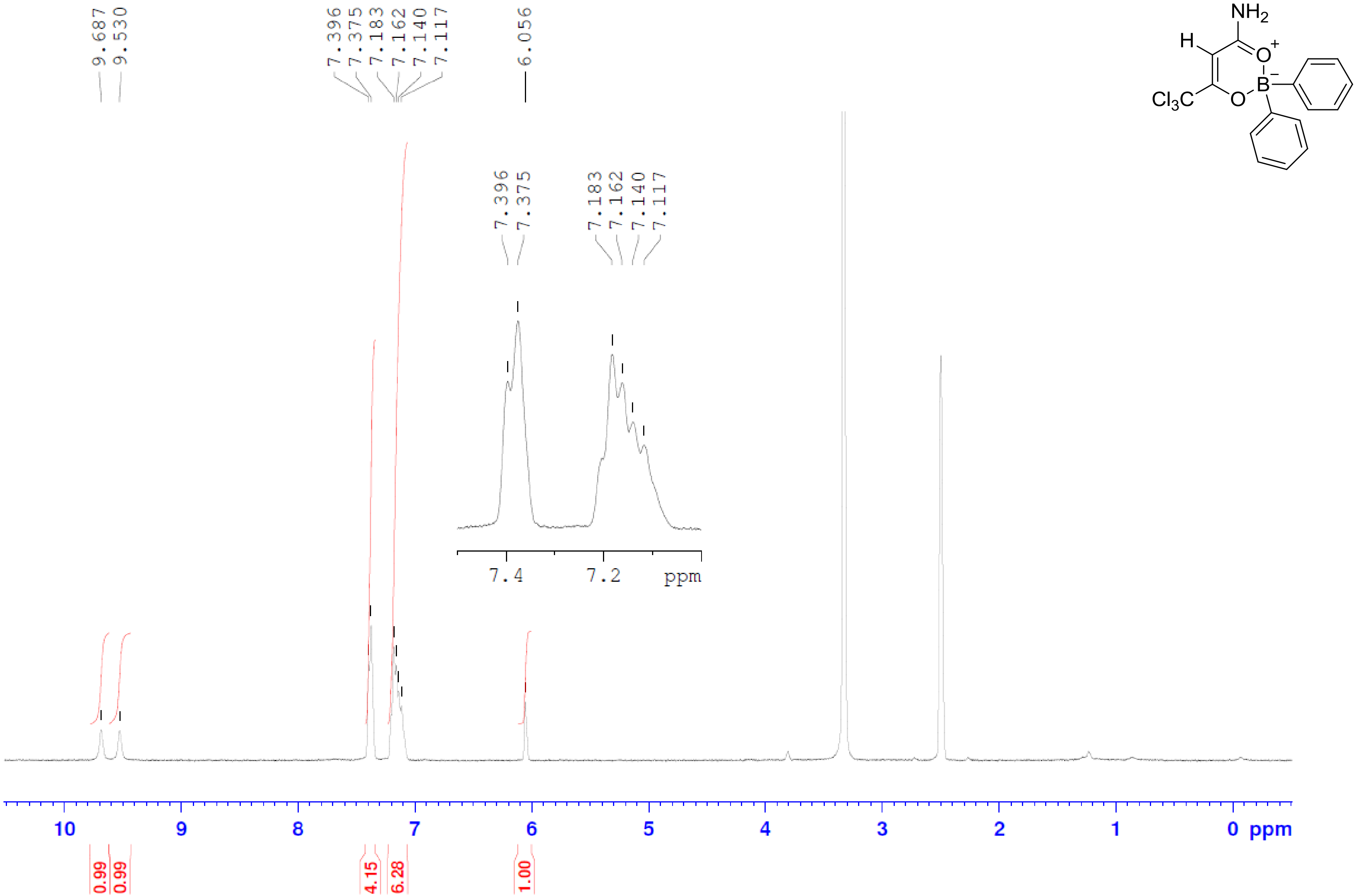

^{1}H NMR spectrum of NBC9 in DMSO-d_6

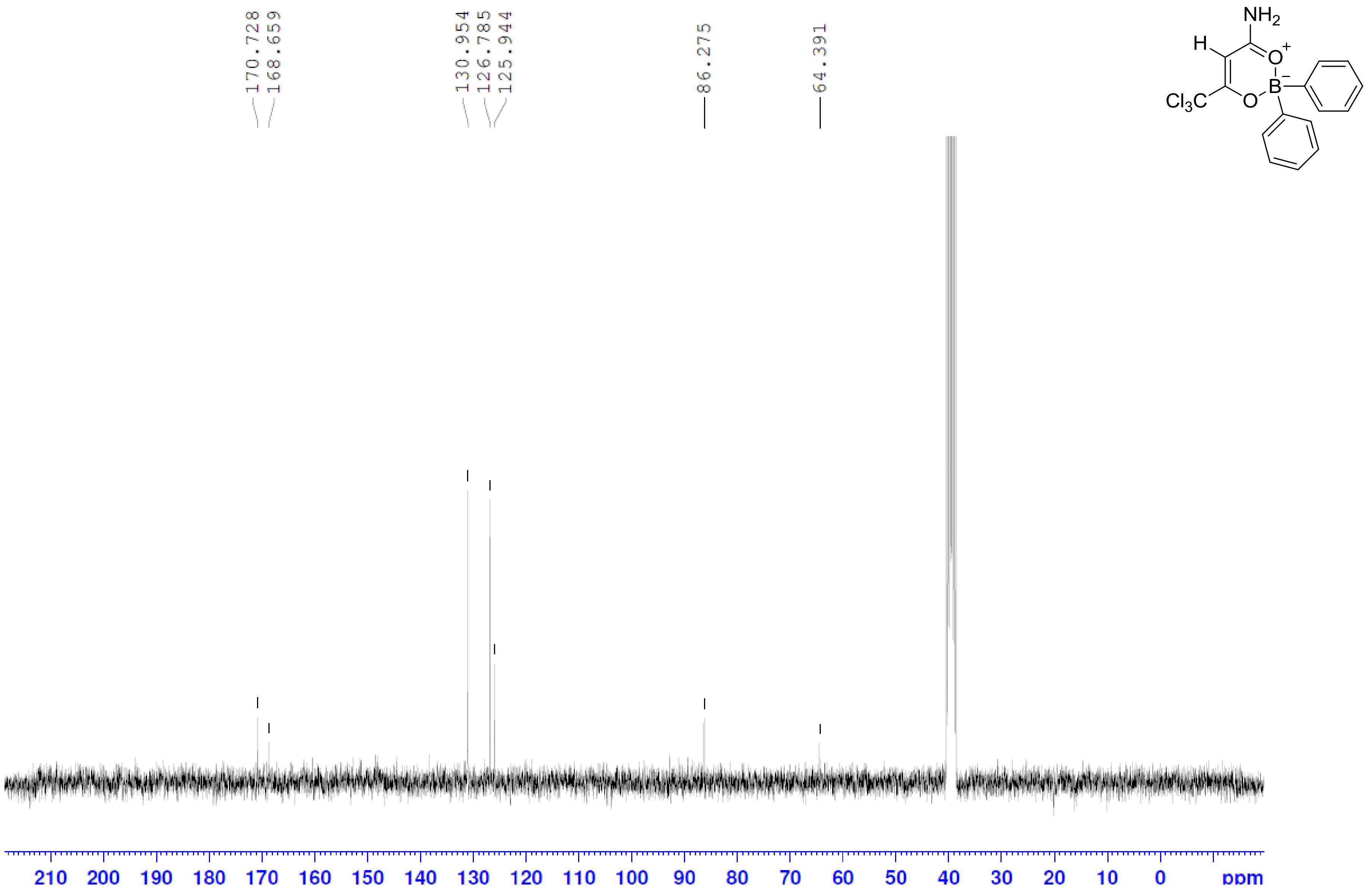

^{13}C NMR spectrum of NBC9 in DMSO-d_6

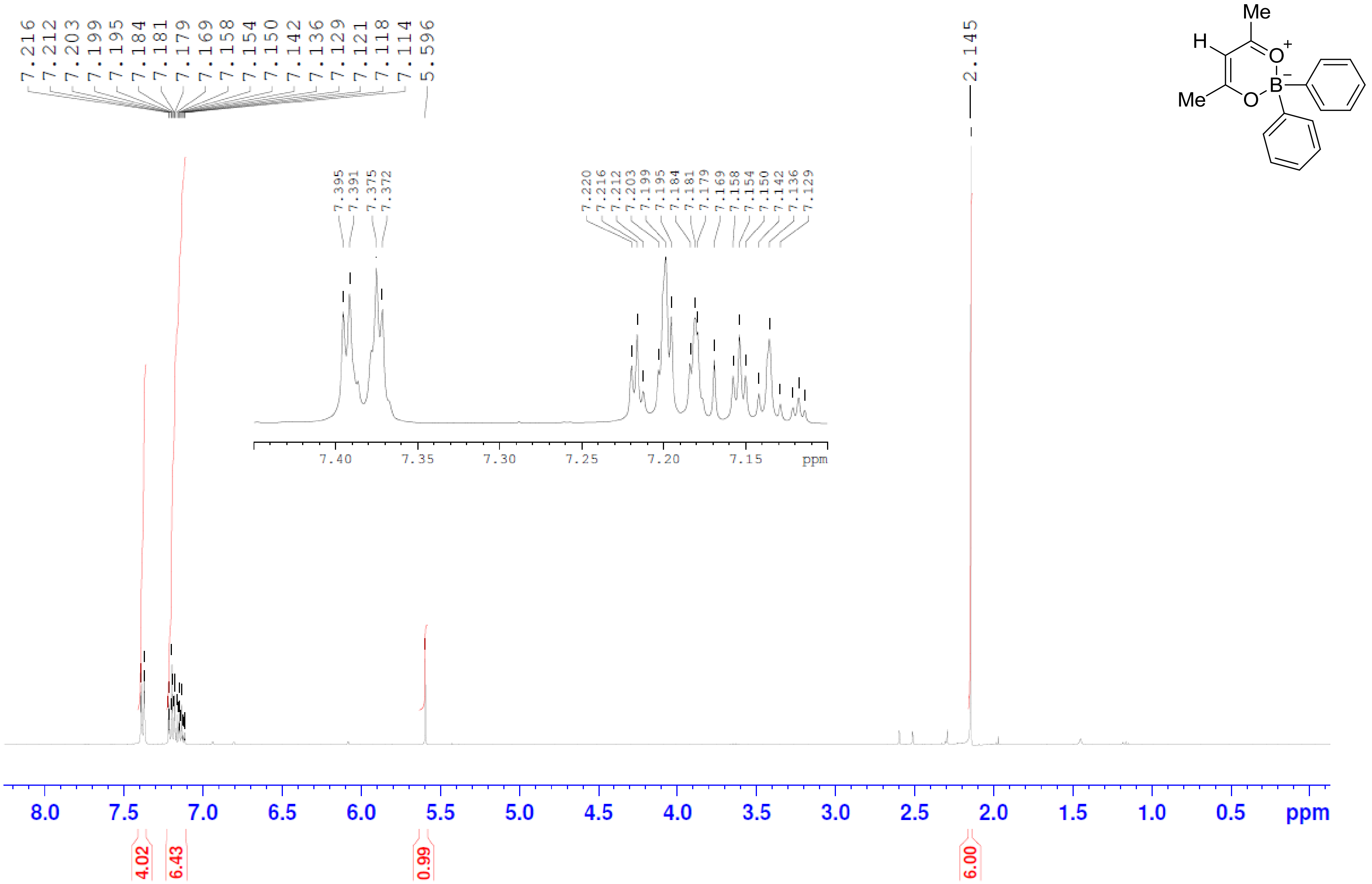

^{1}H NMR spectrum of NBC10 in $CDCl_3$

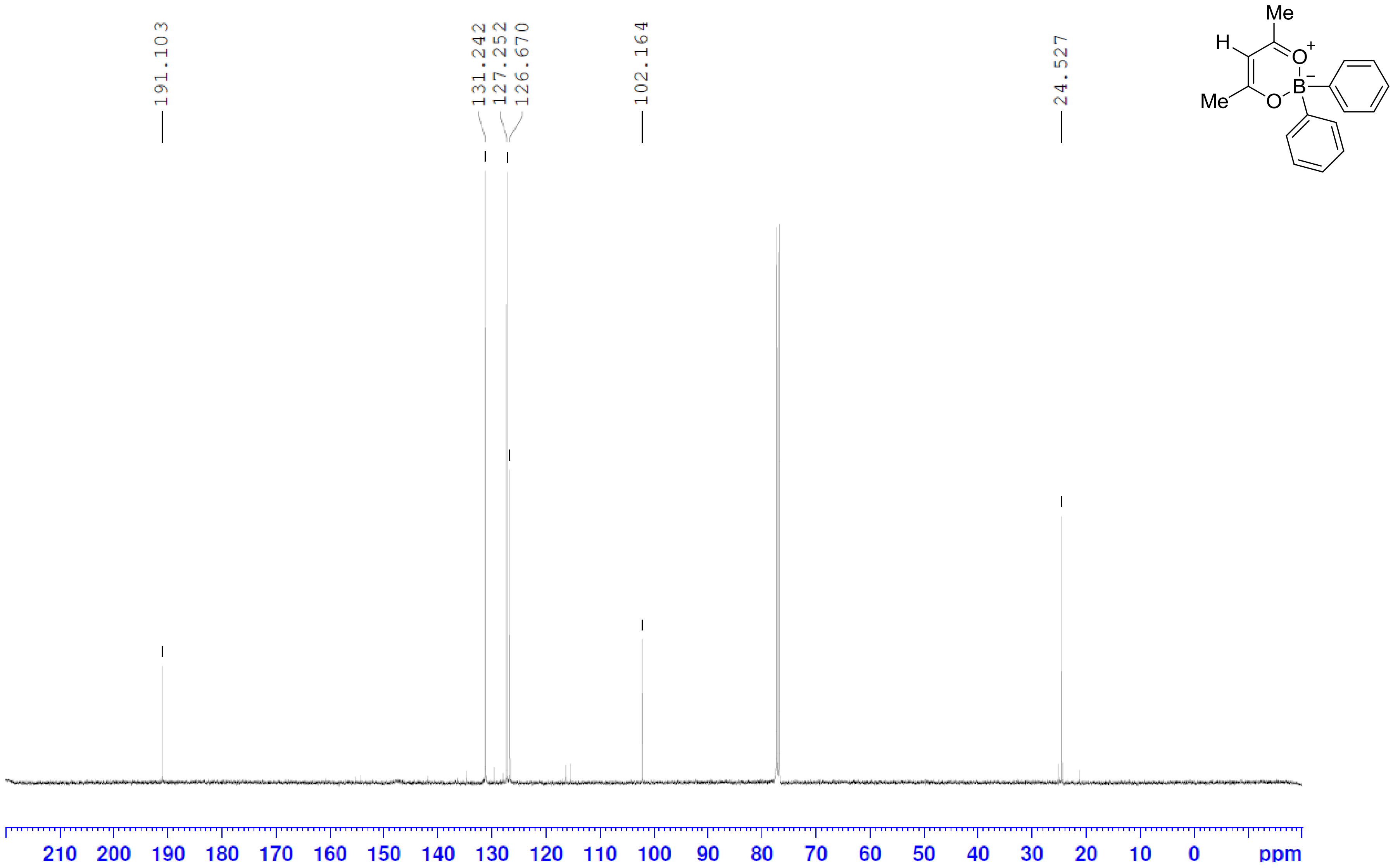

^{13}C NMR spectrum of NBC10 in $CDCl_3$

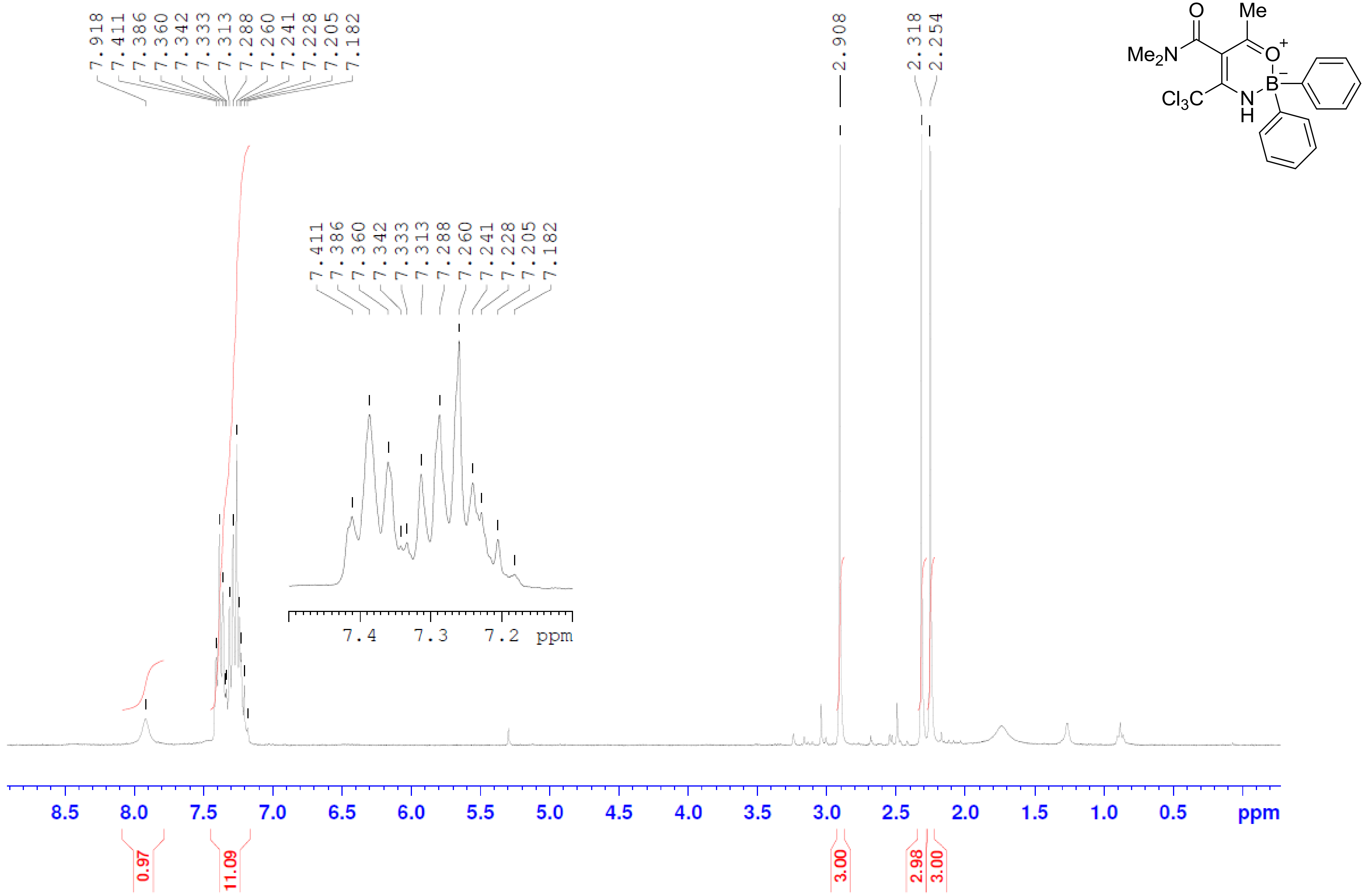

^{1}H NMR spectrum of NBC11 (**16**) in $CDCl_3$

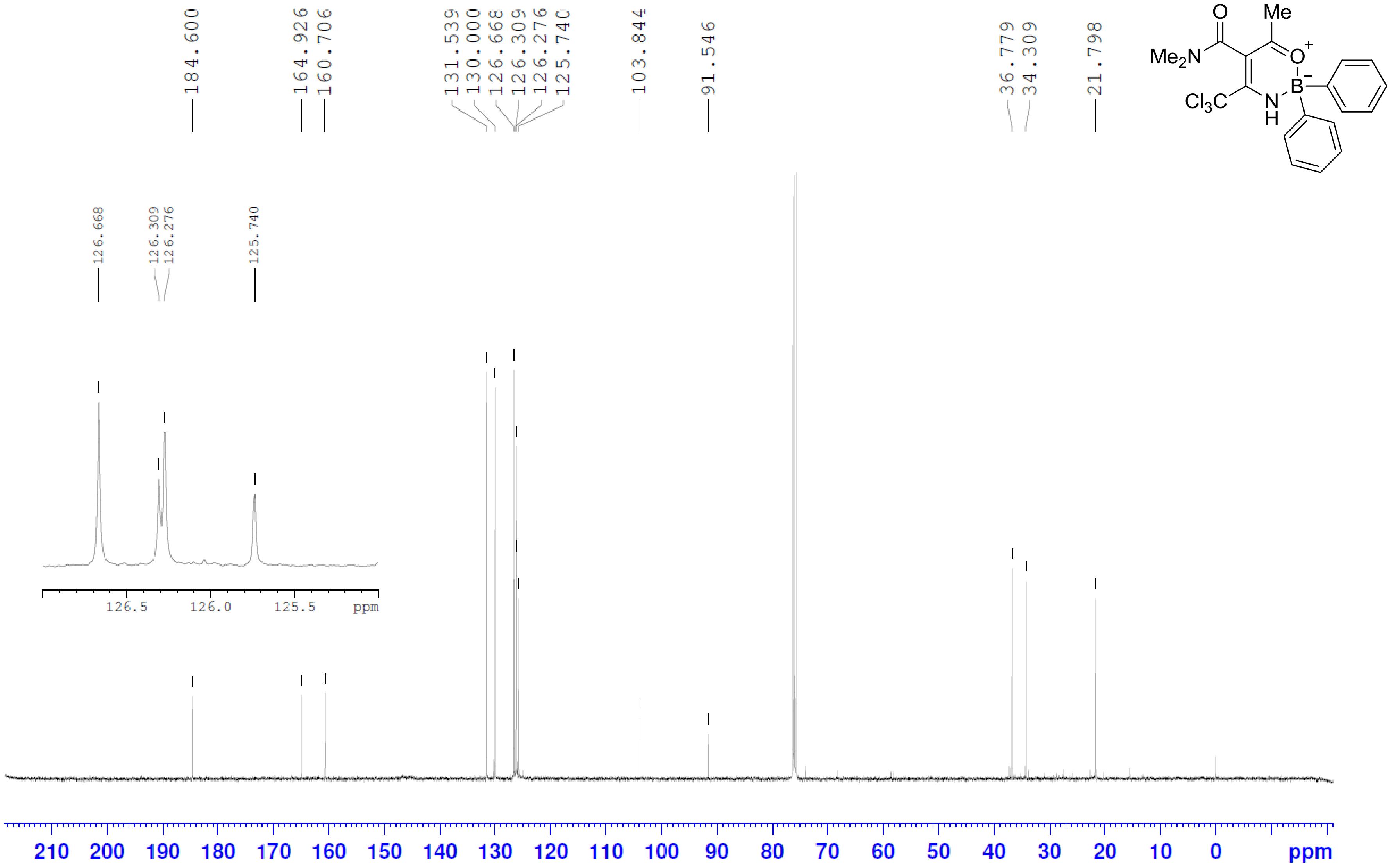

^{13}C NMR spectrum of NBC11 (**16**) in $CDCl_3$

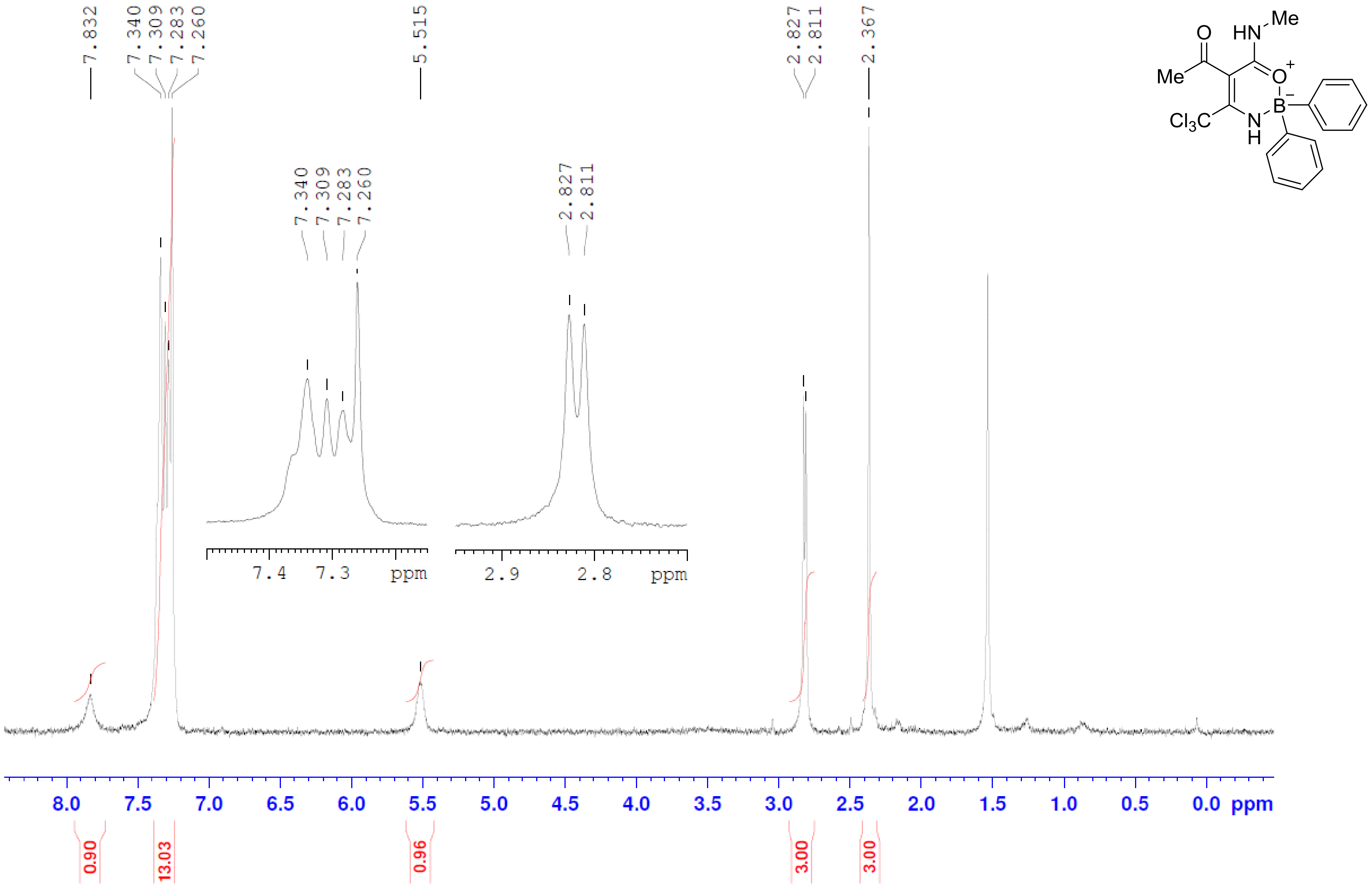

^{1}H NMR spectrum of NBC12 in $CDCl_3$

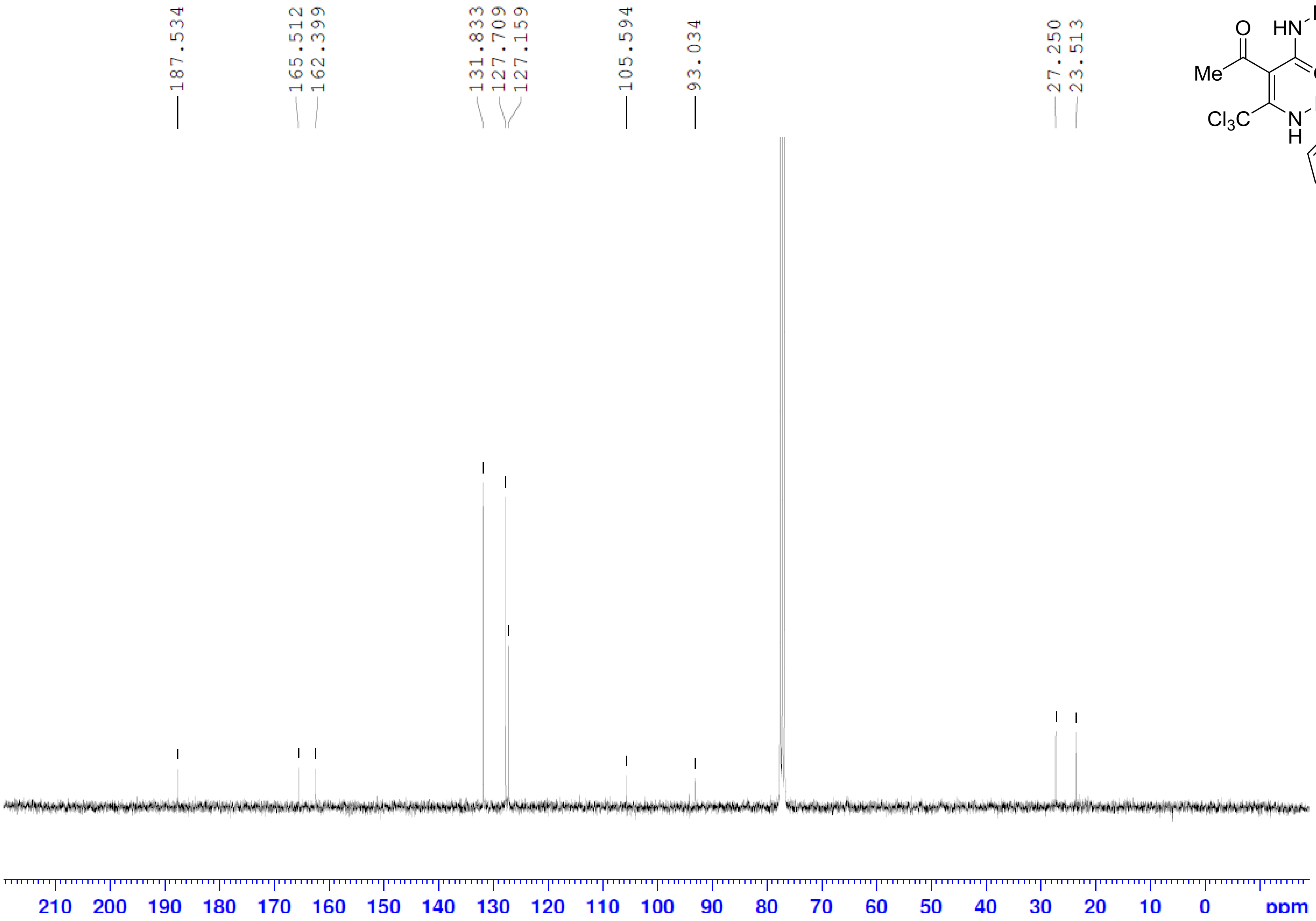

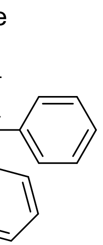

^{13}C NMR spectrum of NBC12 in $CDCl_3$

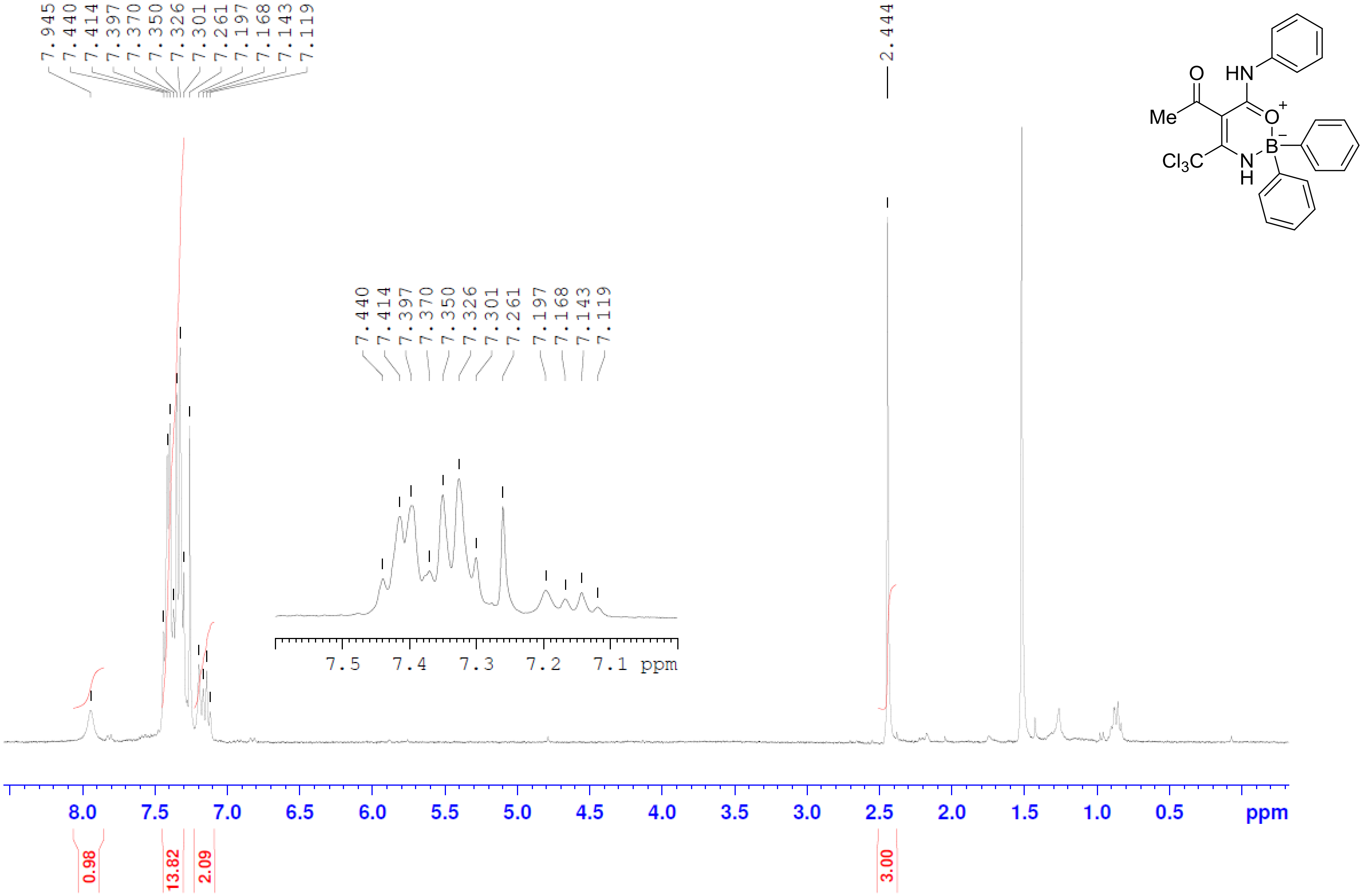

^{1}H NMR spectrum of NBC13 in $CDCl_3$

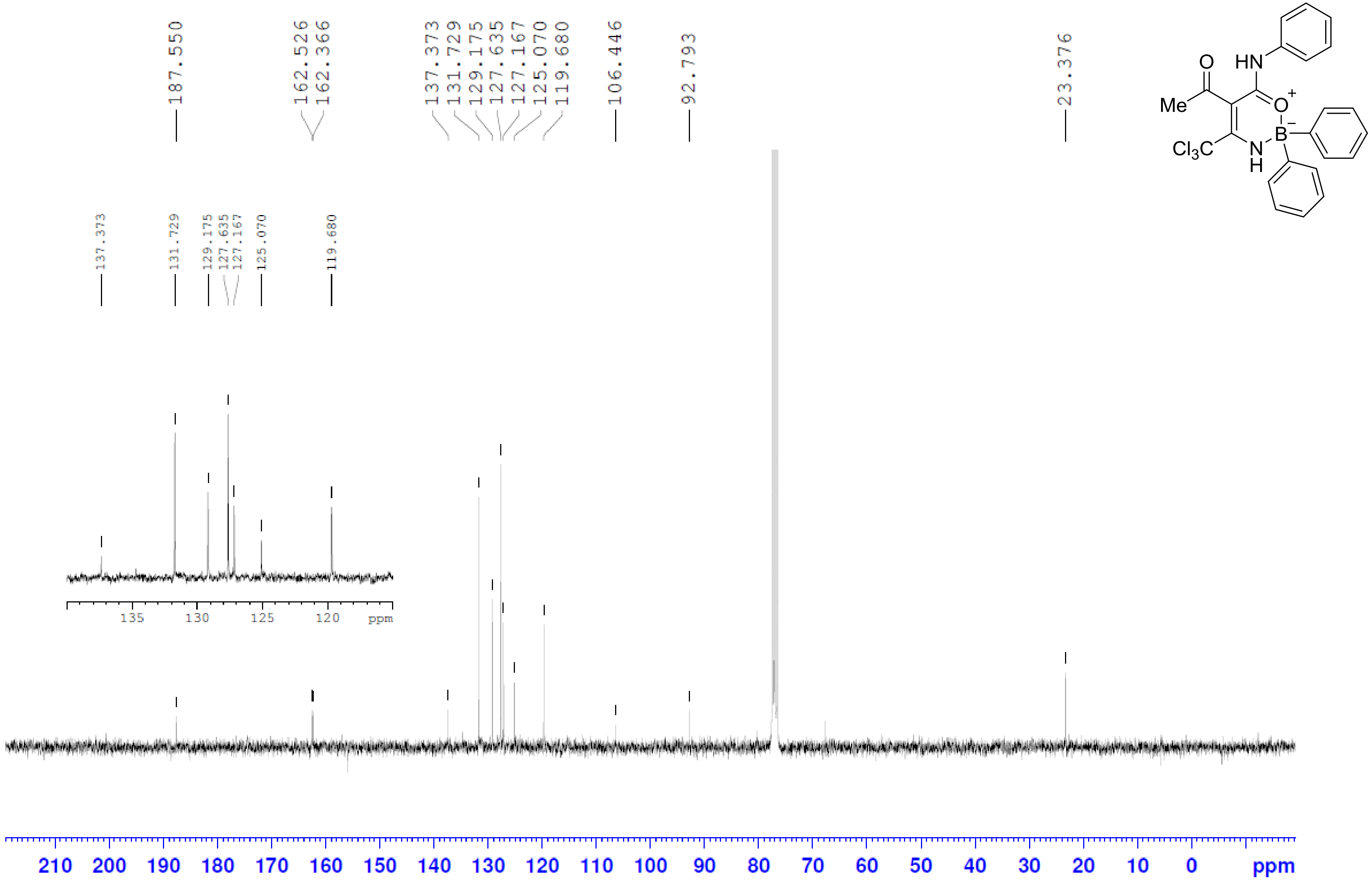

^{13}C NMR spectrum of NBC13 in $CDCl_3$

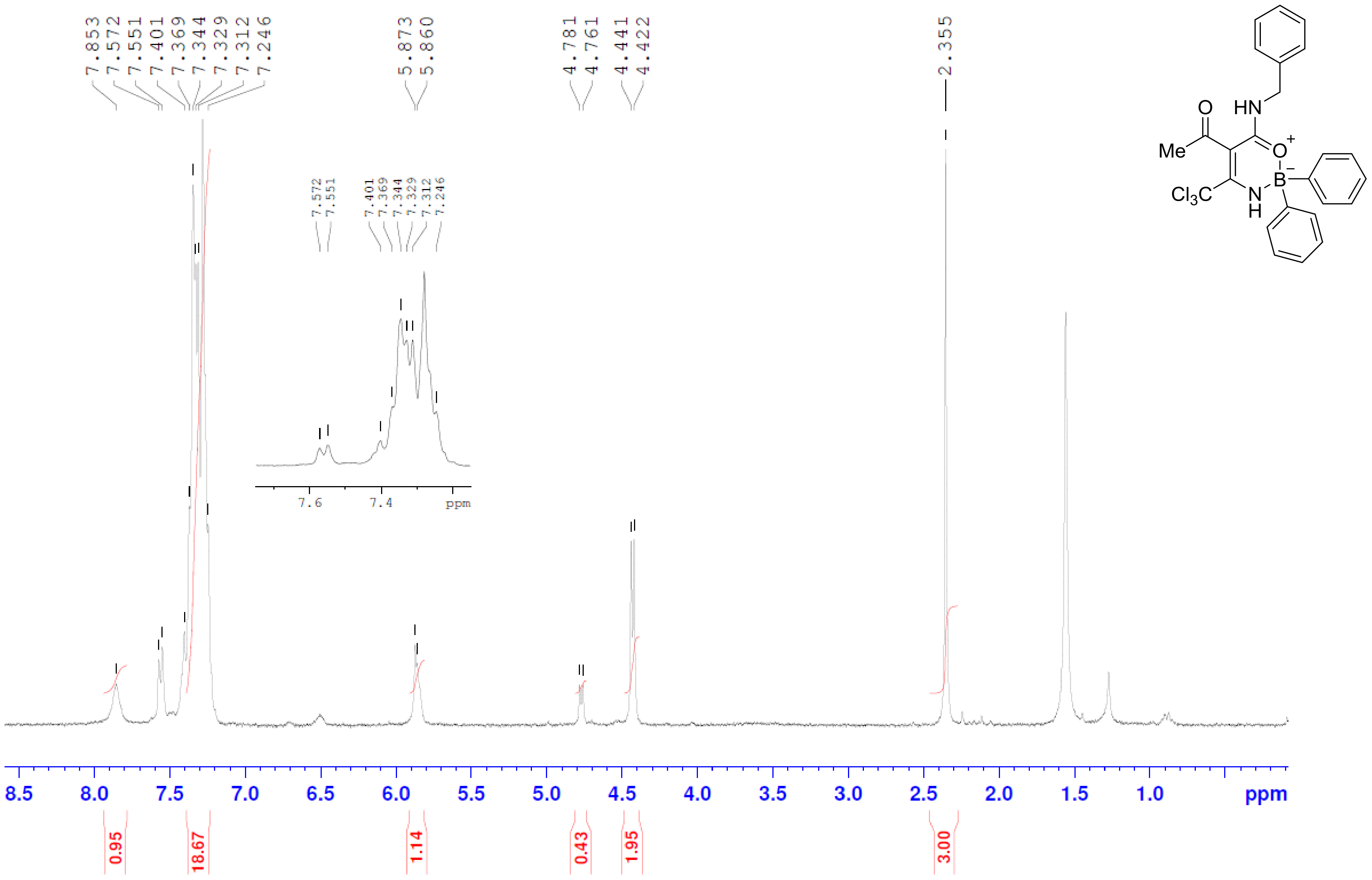

^{1}H NMR spectrum of NBC14 in $CDCl_3$

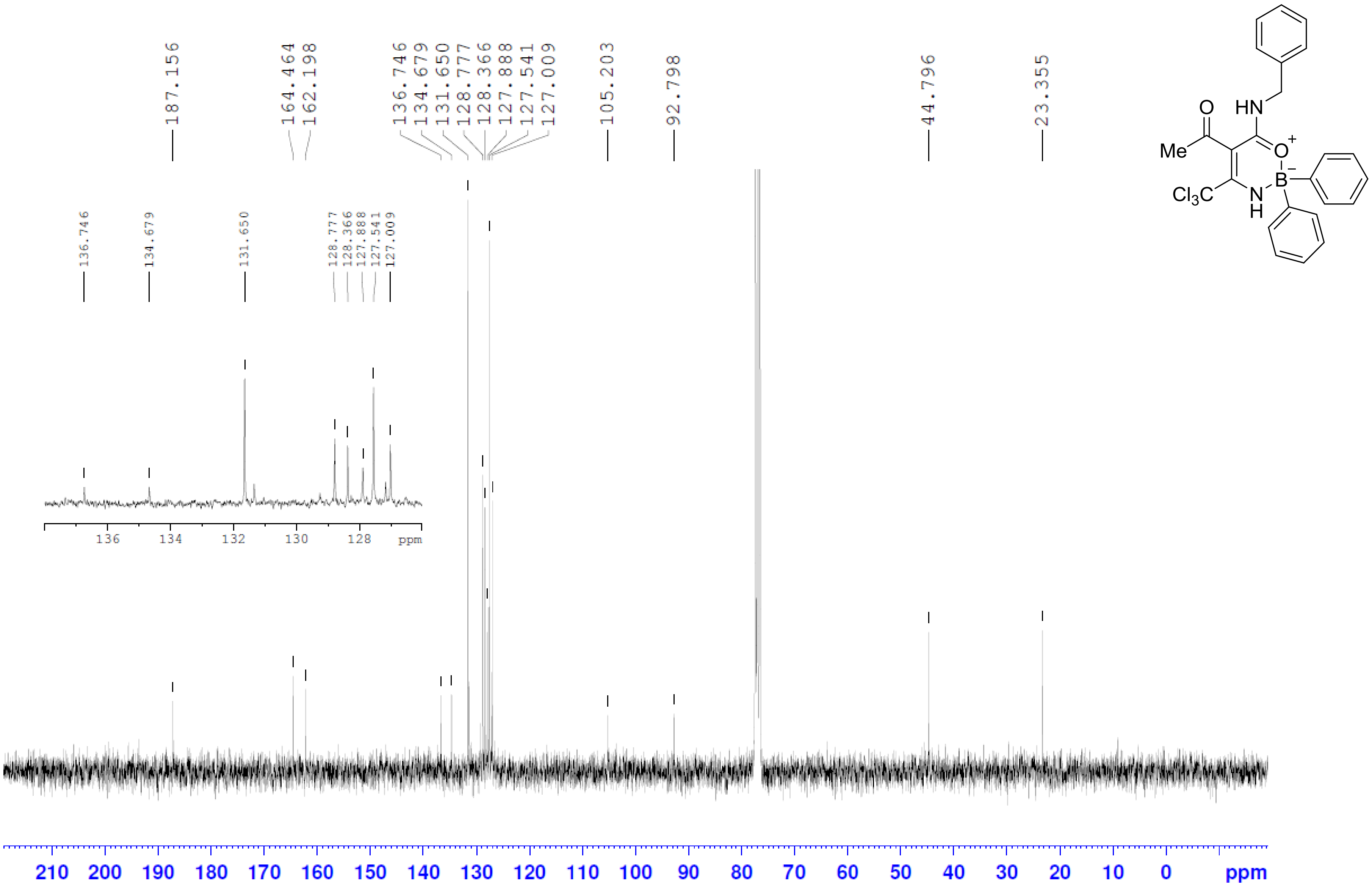

^{13}C NMR spectrum of NBC14 in $CDCl_3$

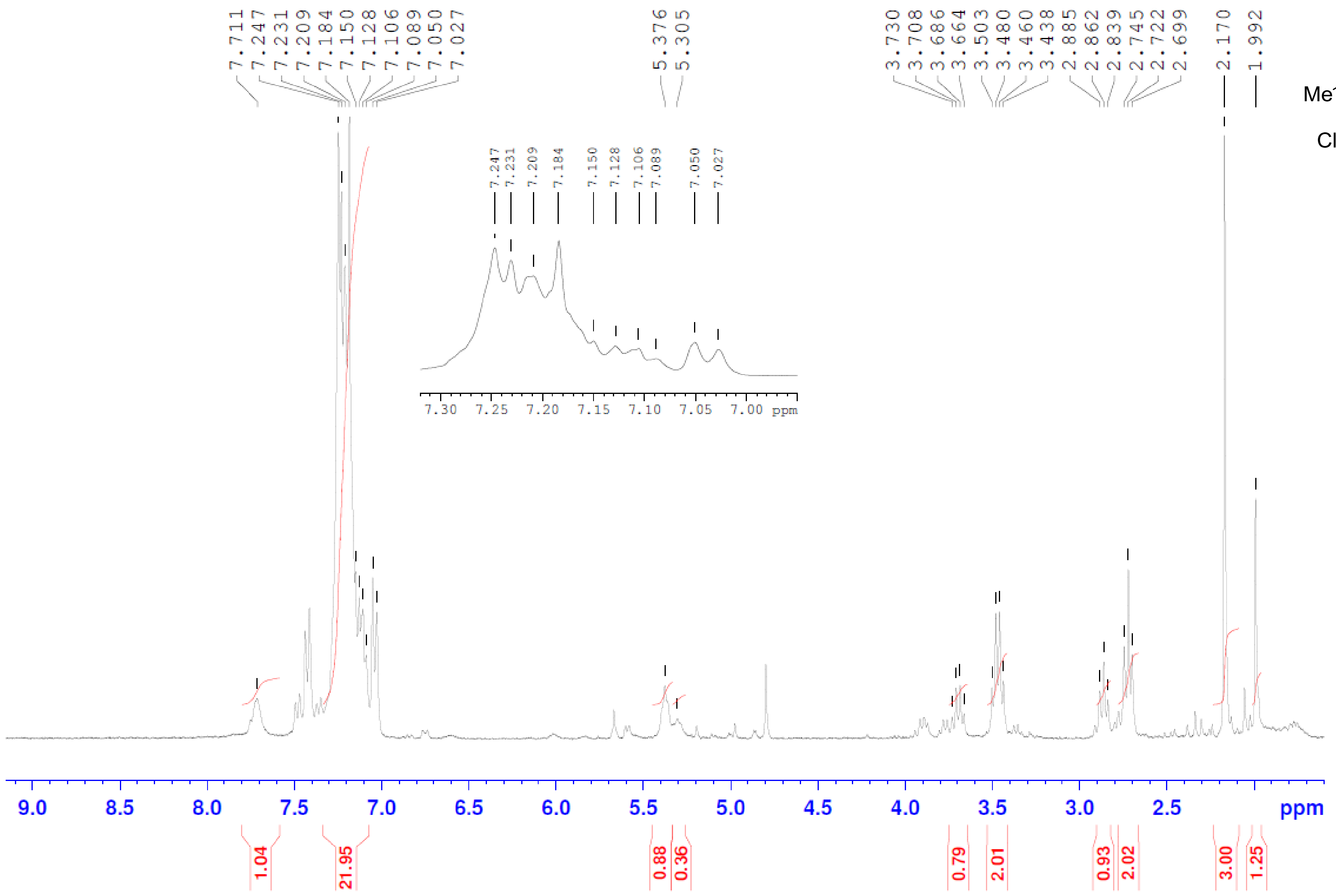

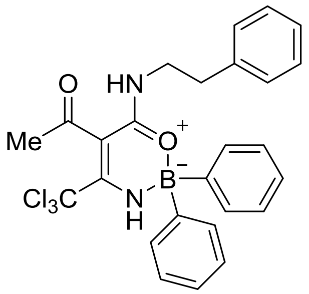

^{1}H NMR spectrum of NBC15 in $CDCl_3$

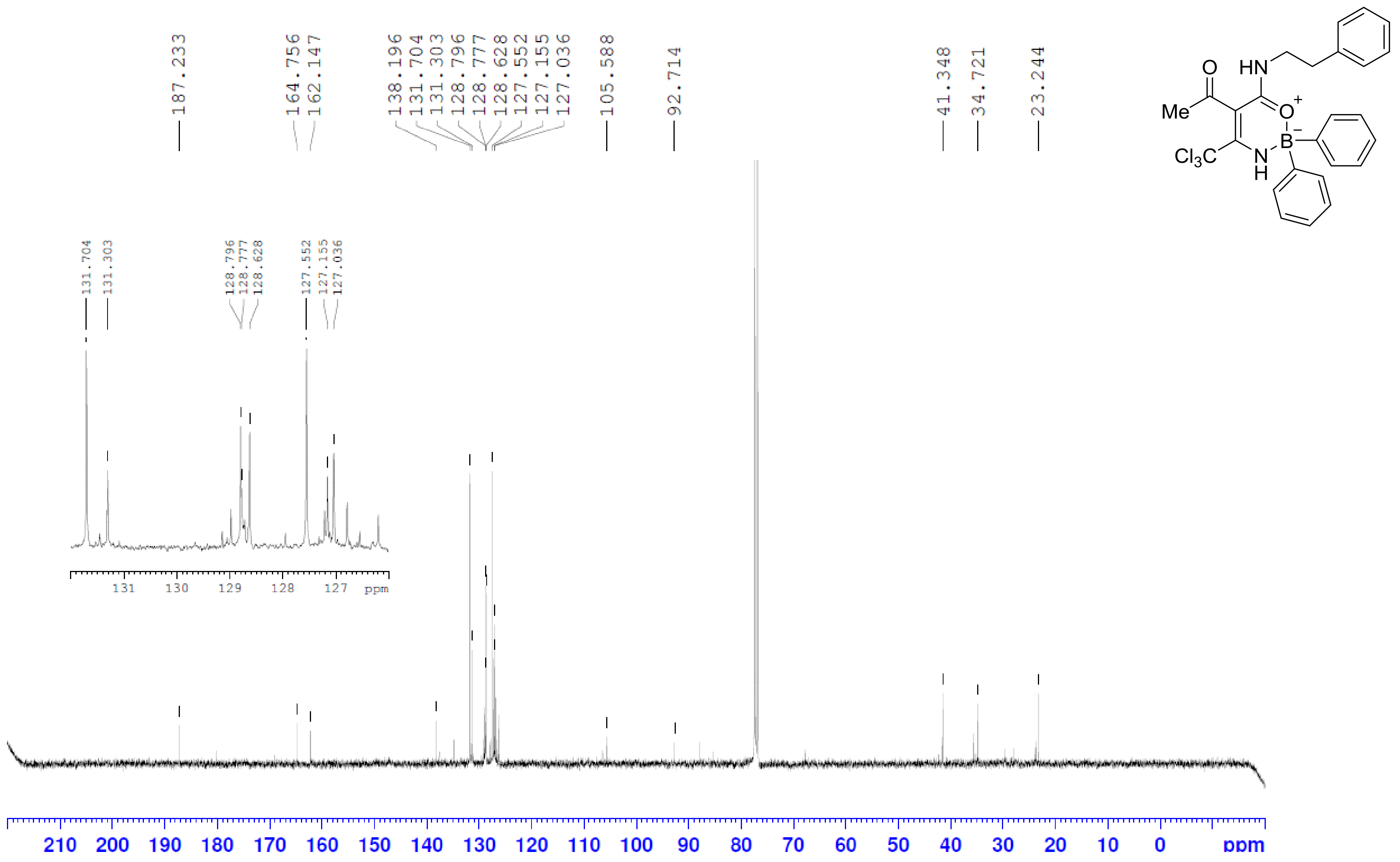

^{13}C NMR spectrum of NBC15 in $CDCl_3$

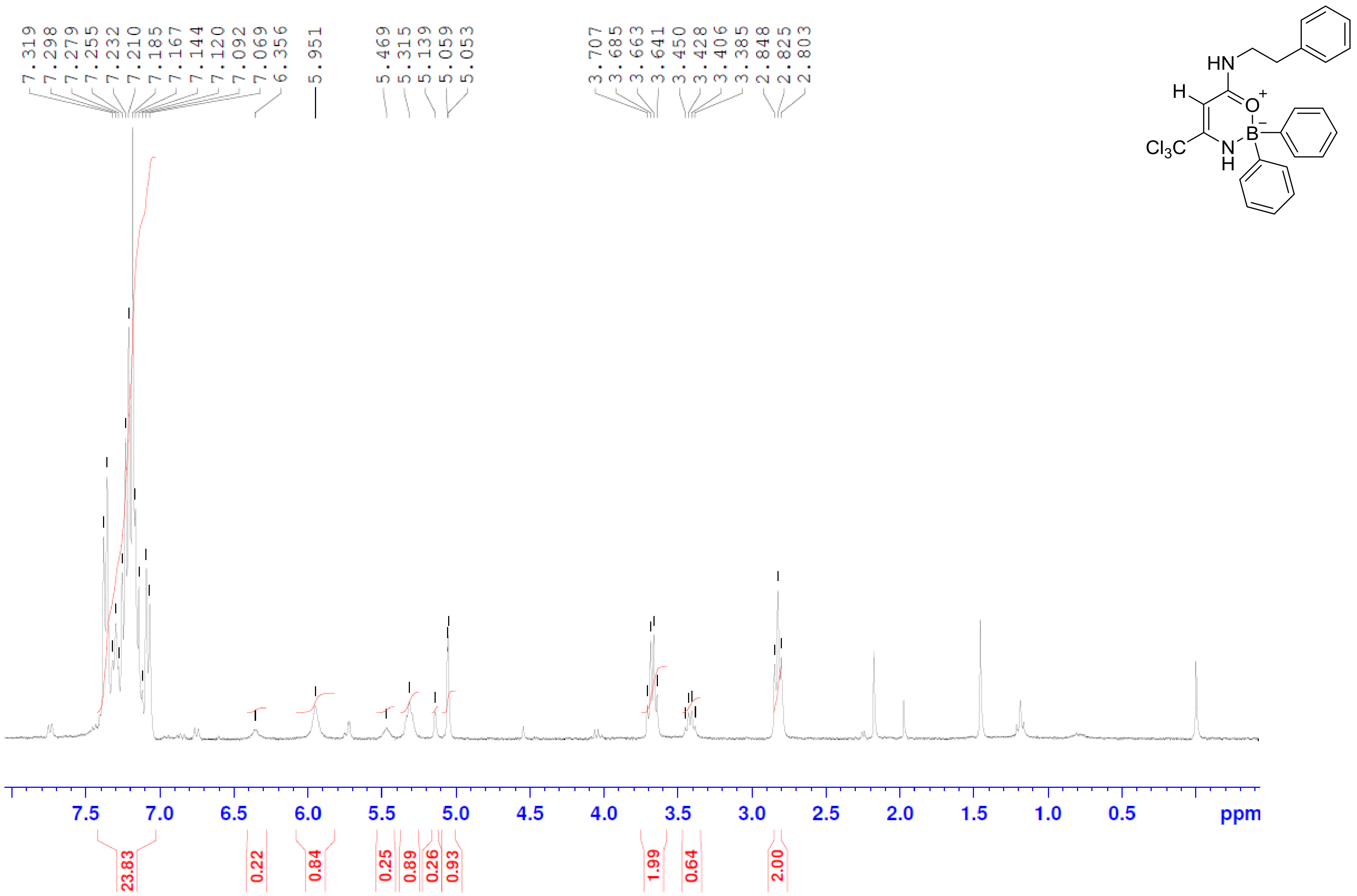

^{1}H NMR spectrum of NBC16 in $CDCl_3$

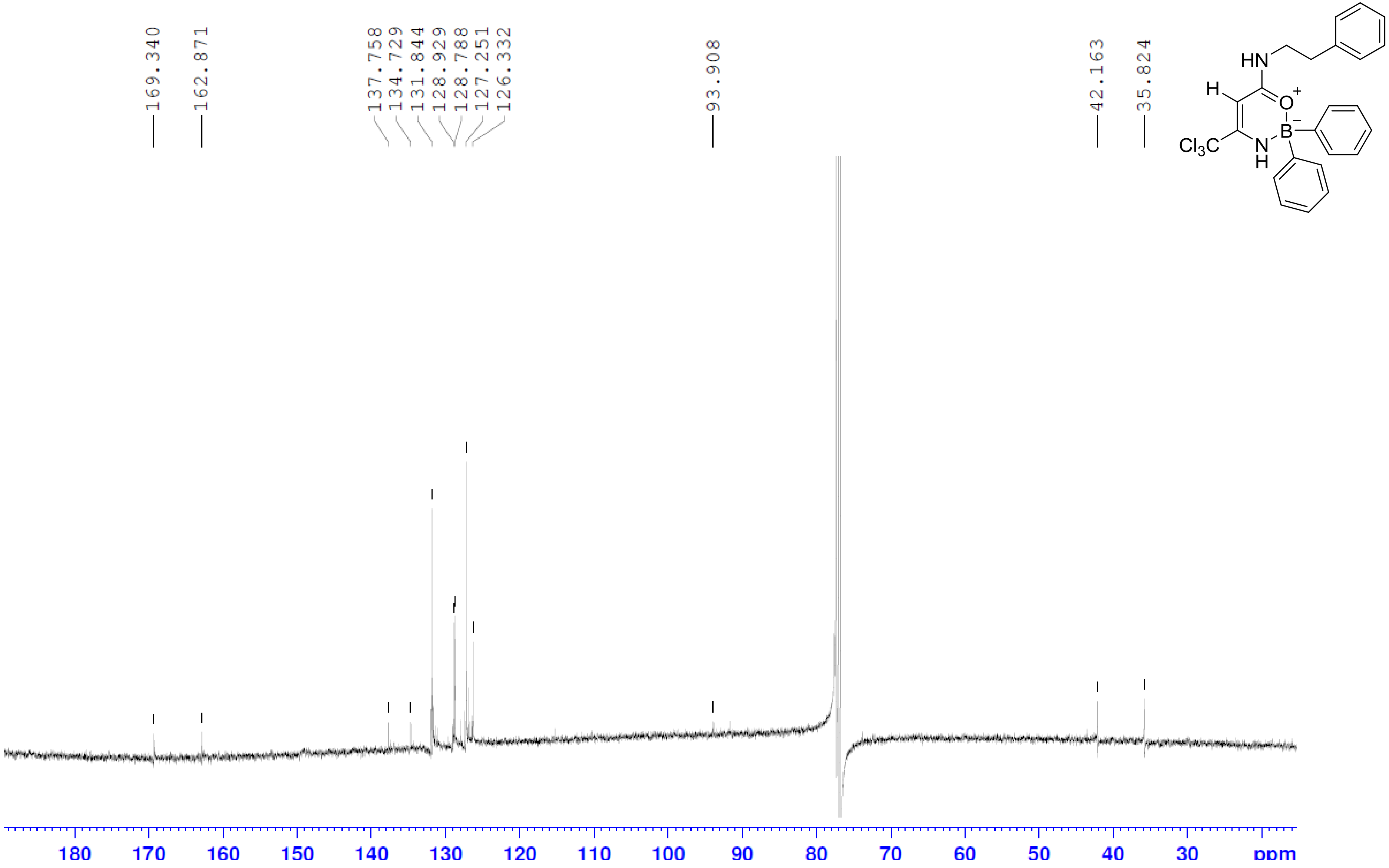

^{13}C NMR spectrum of NBC16 in $CDCl_3$

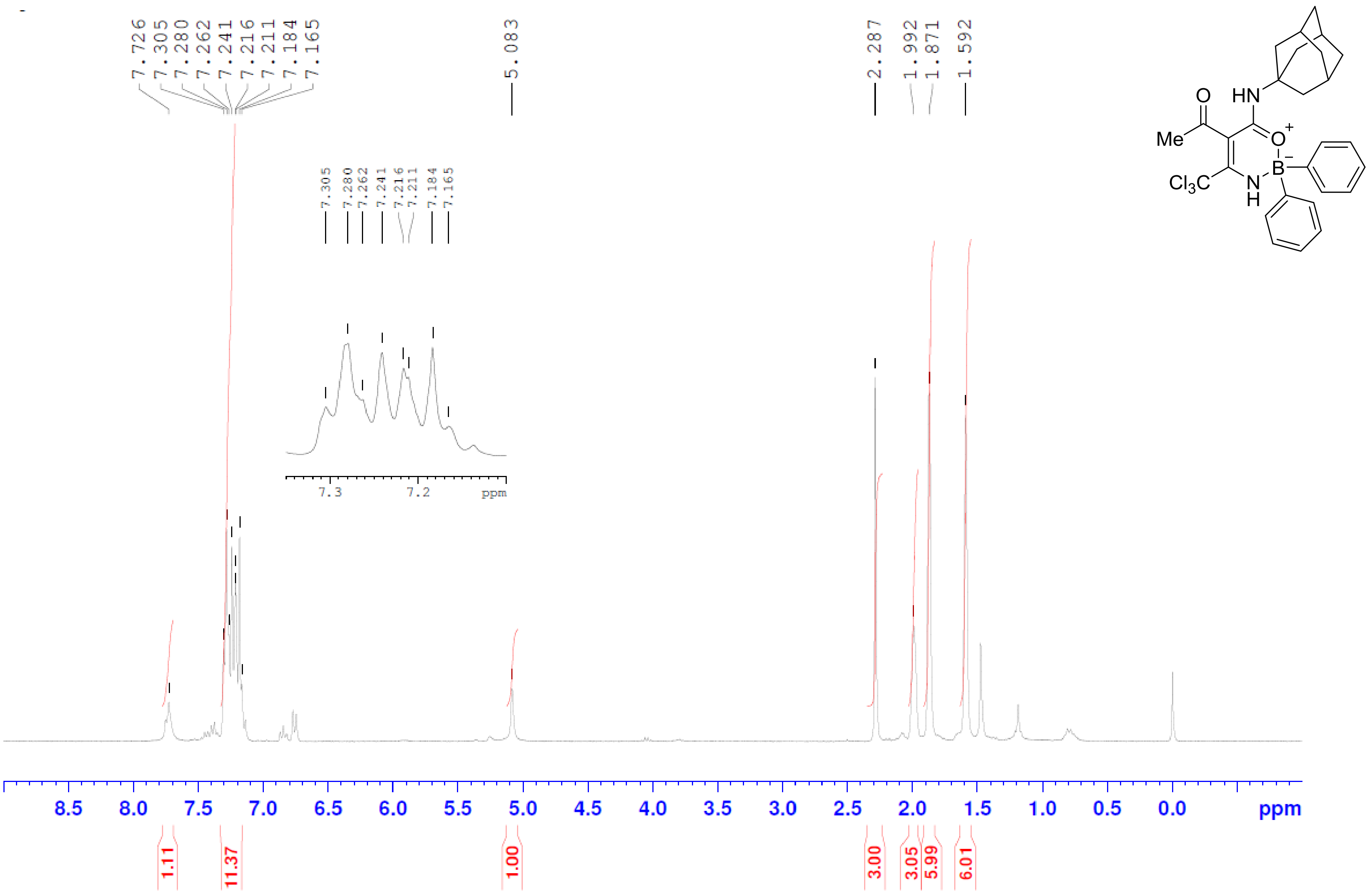

^{1}H NMR spectrum of NBC17 in $CDCl_3$

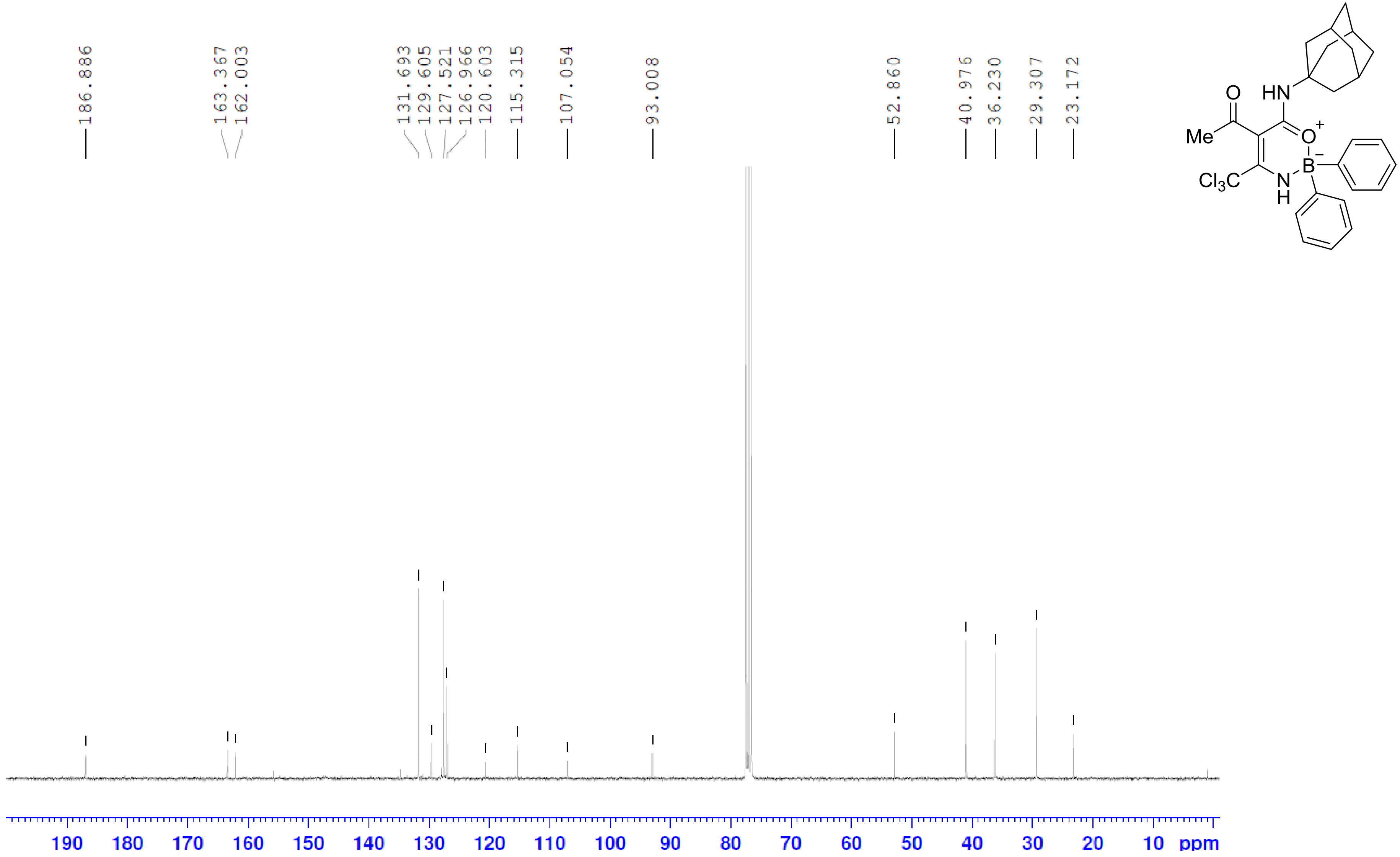

^{13}C NMR spectrum of NBC17 in $CDCl_3$

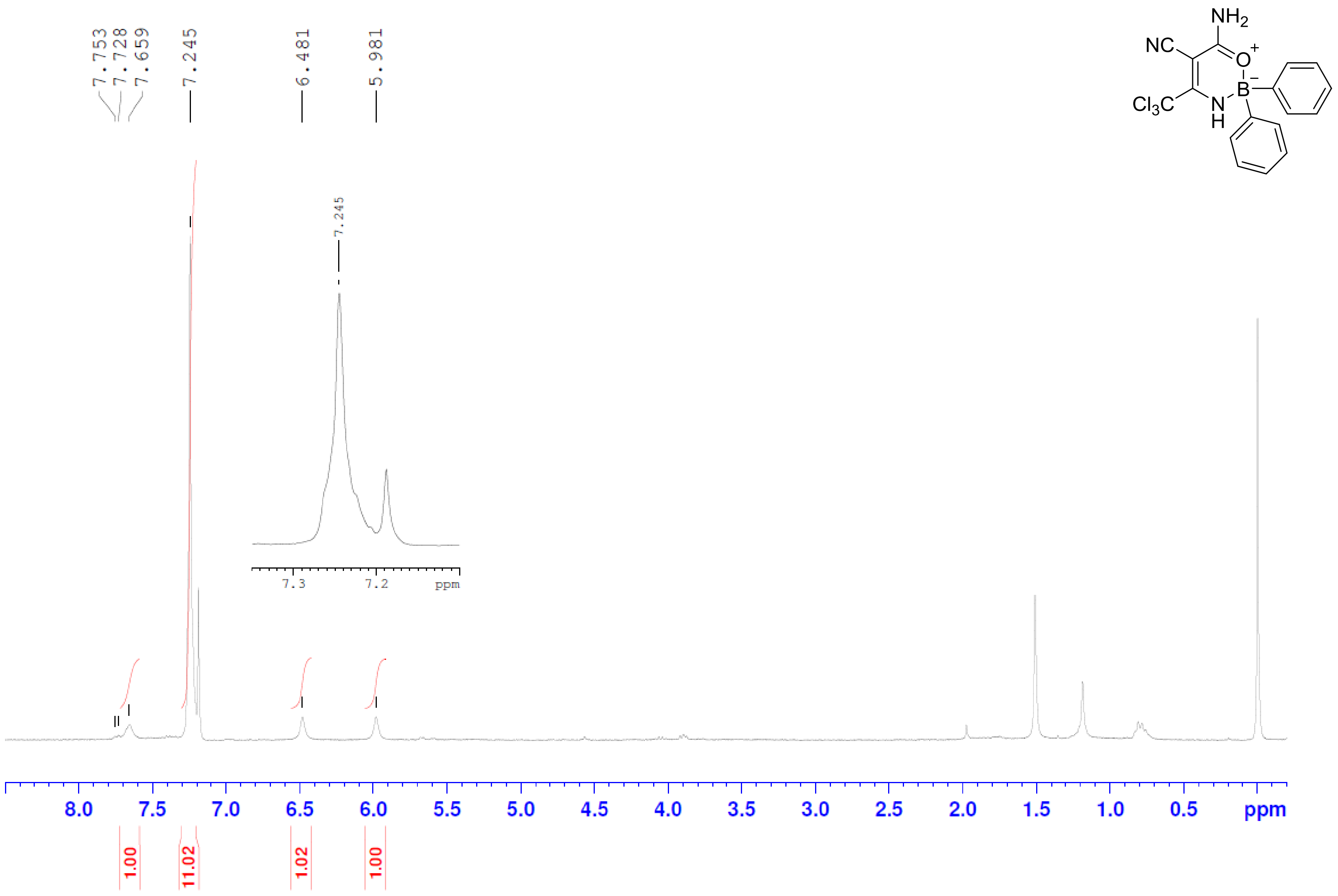

^{1}H NMR spectrum of NBC18 in $CDCl_3$

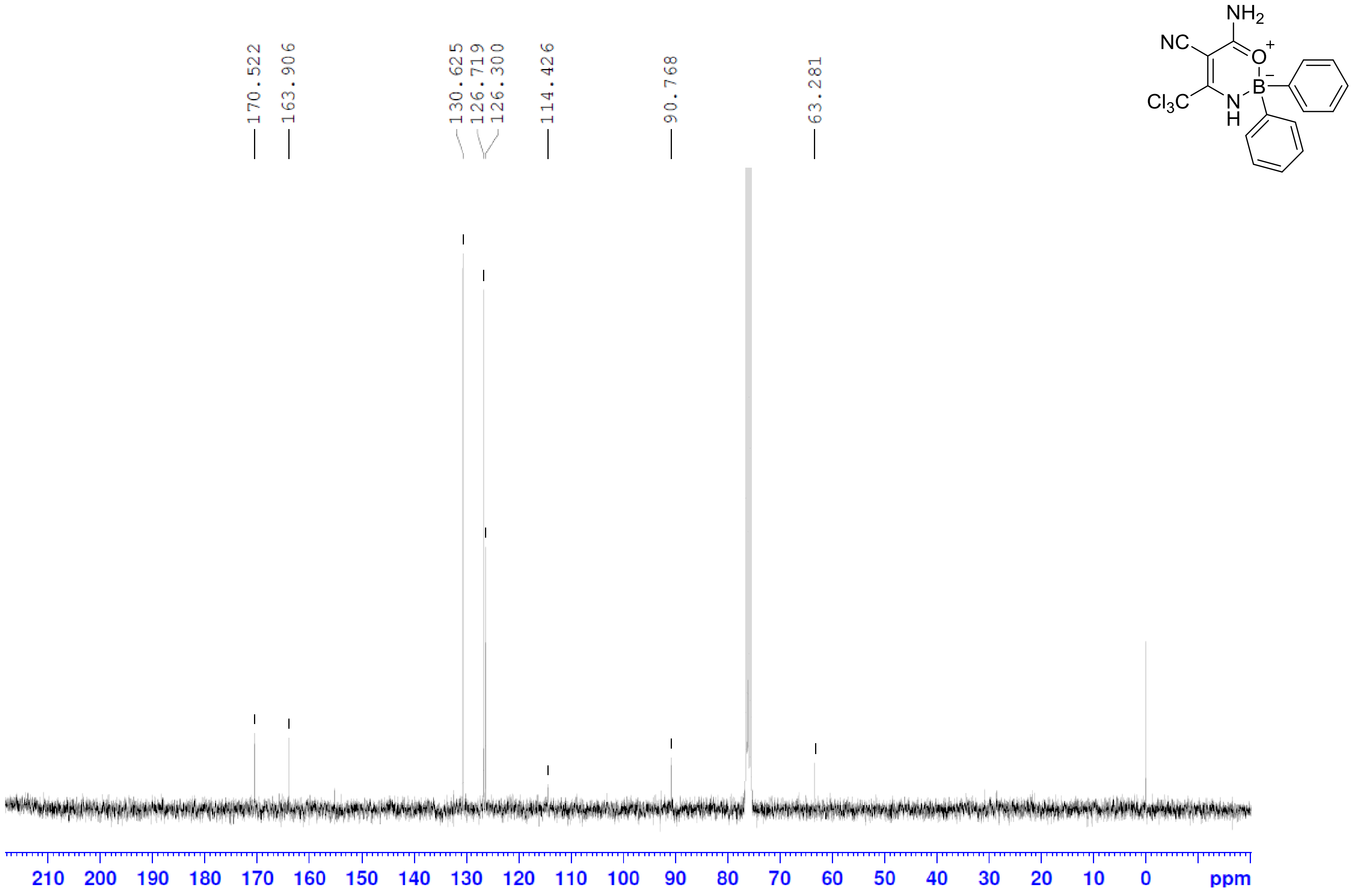

^{13}C NMR spectrum of NBC18 in $CDCl_3$

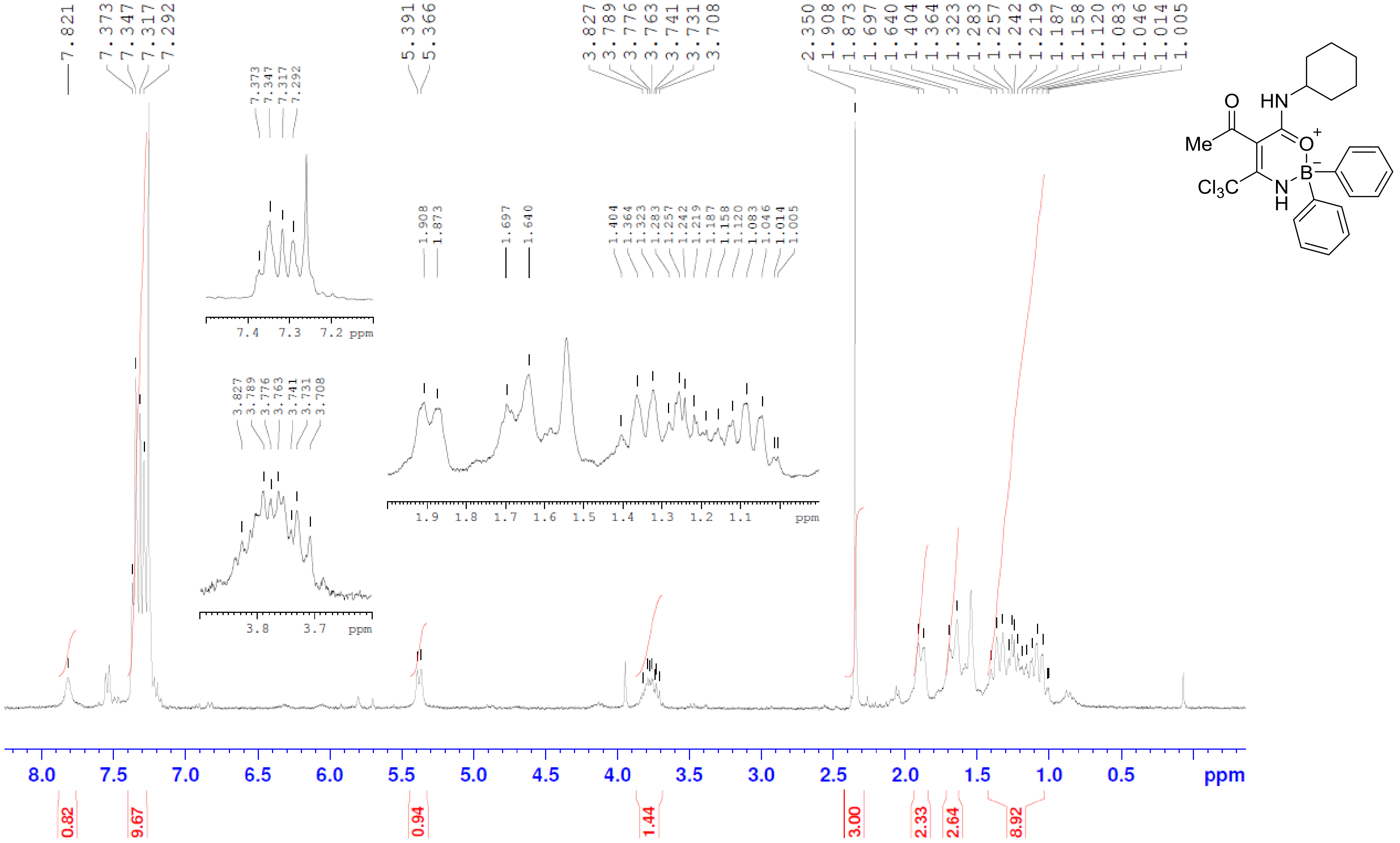

^{1}H NMR spectrum of NBC19 (**17**) in $CDCl_3$

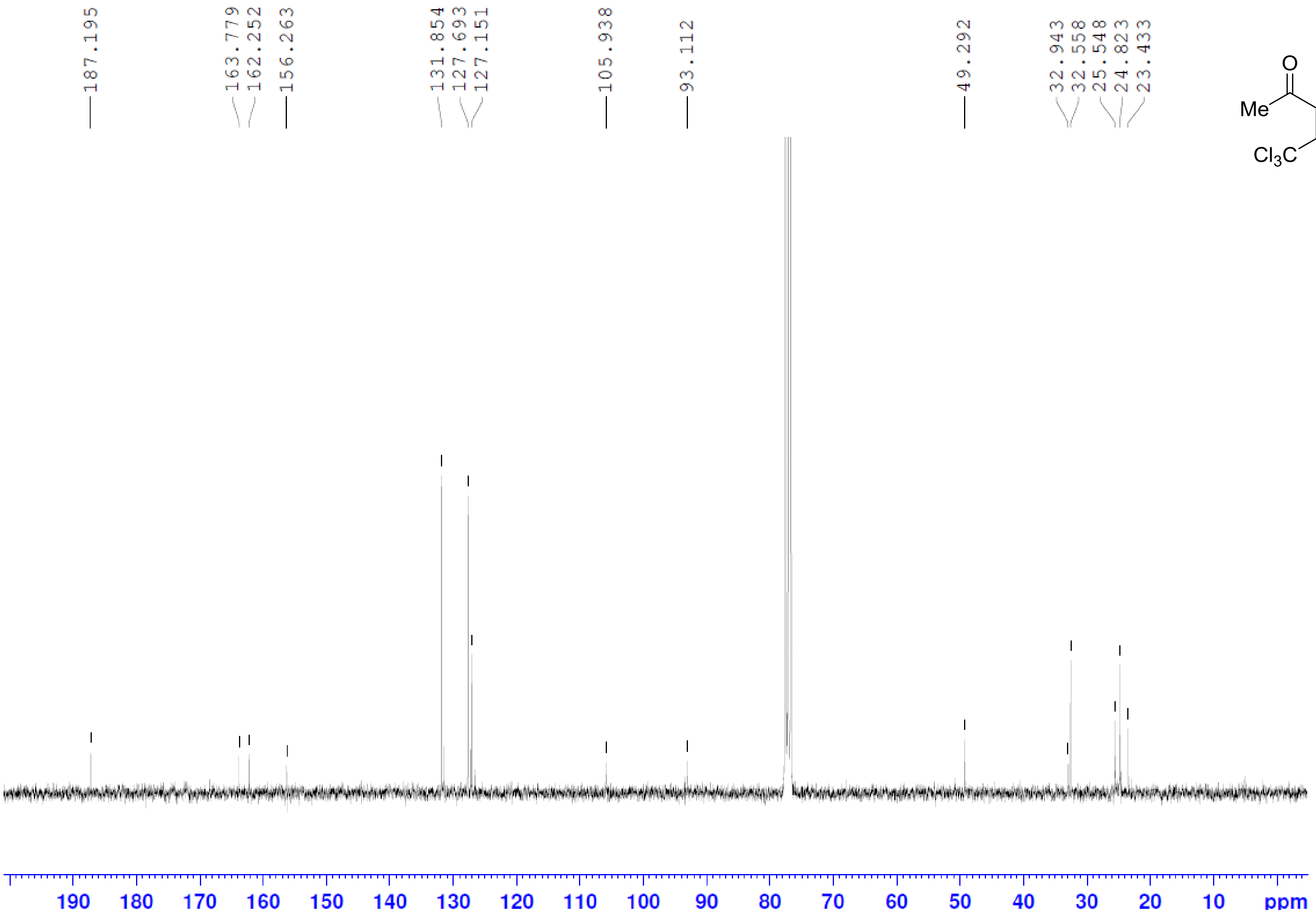

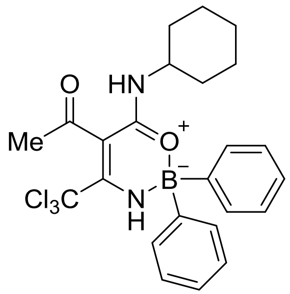

^{13}C NMR spectrum of NBC19 (**17**) in $CDCl_3$

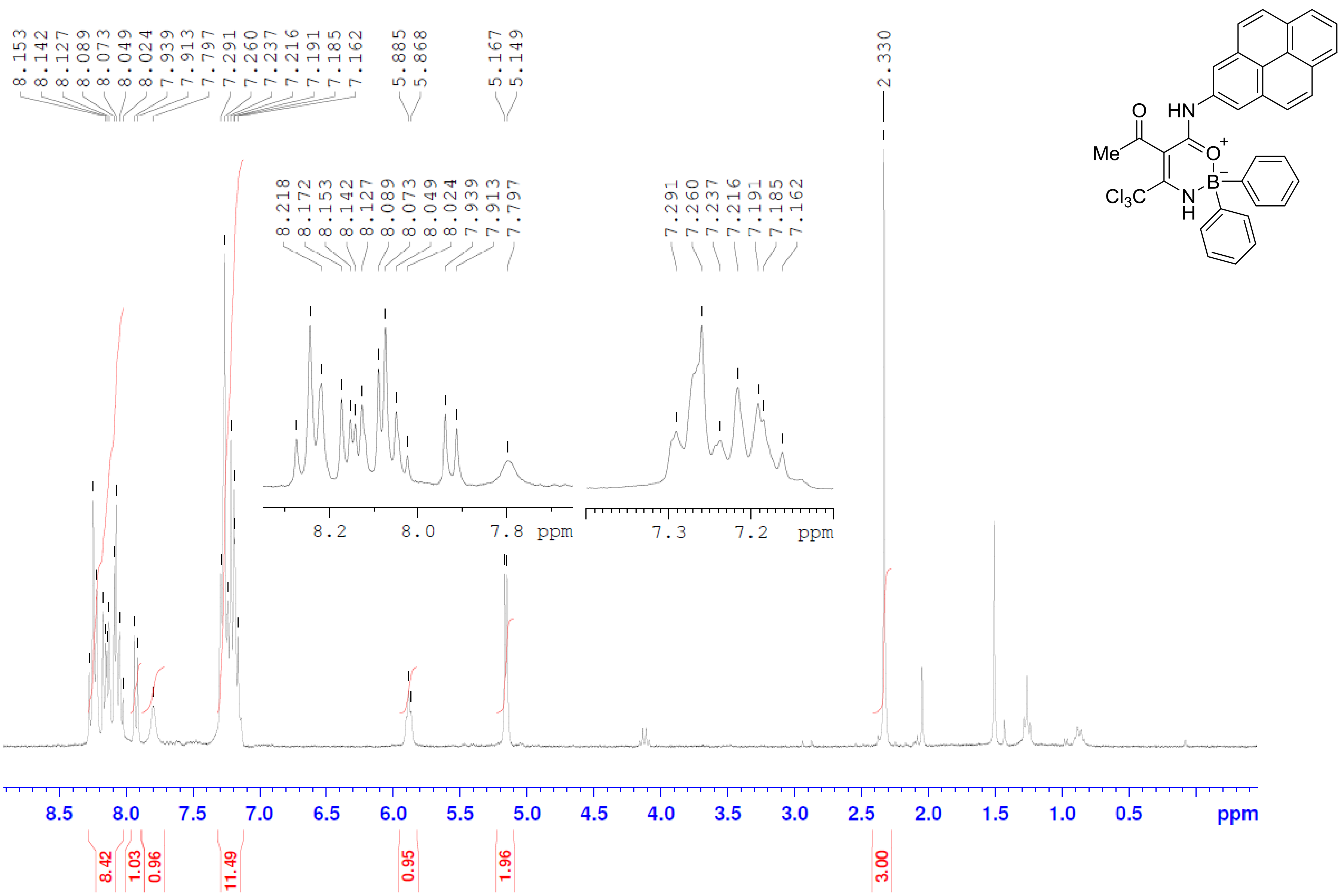

^{1}H NMR spectrum of NBC20 in $CDCl_3$

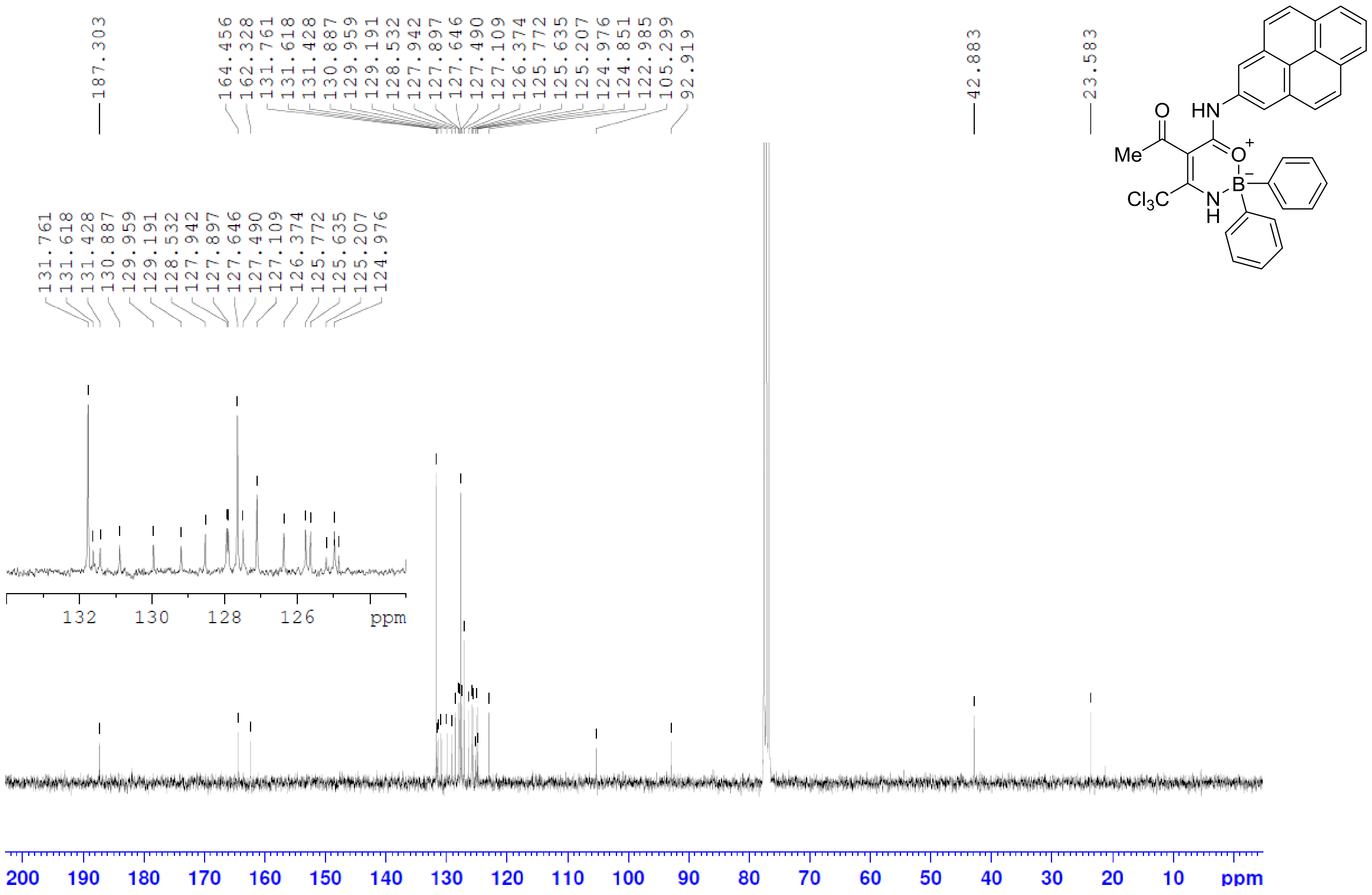

^{13}C NMR spectrum of NBC20 in $CDCl_3$

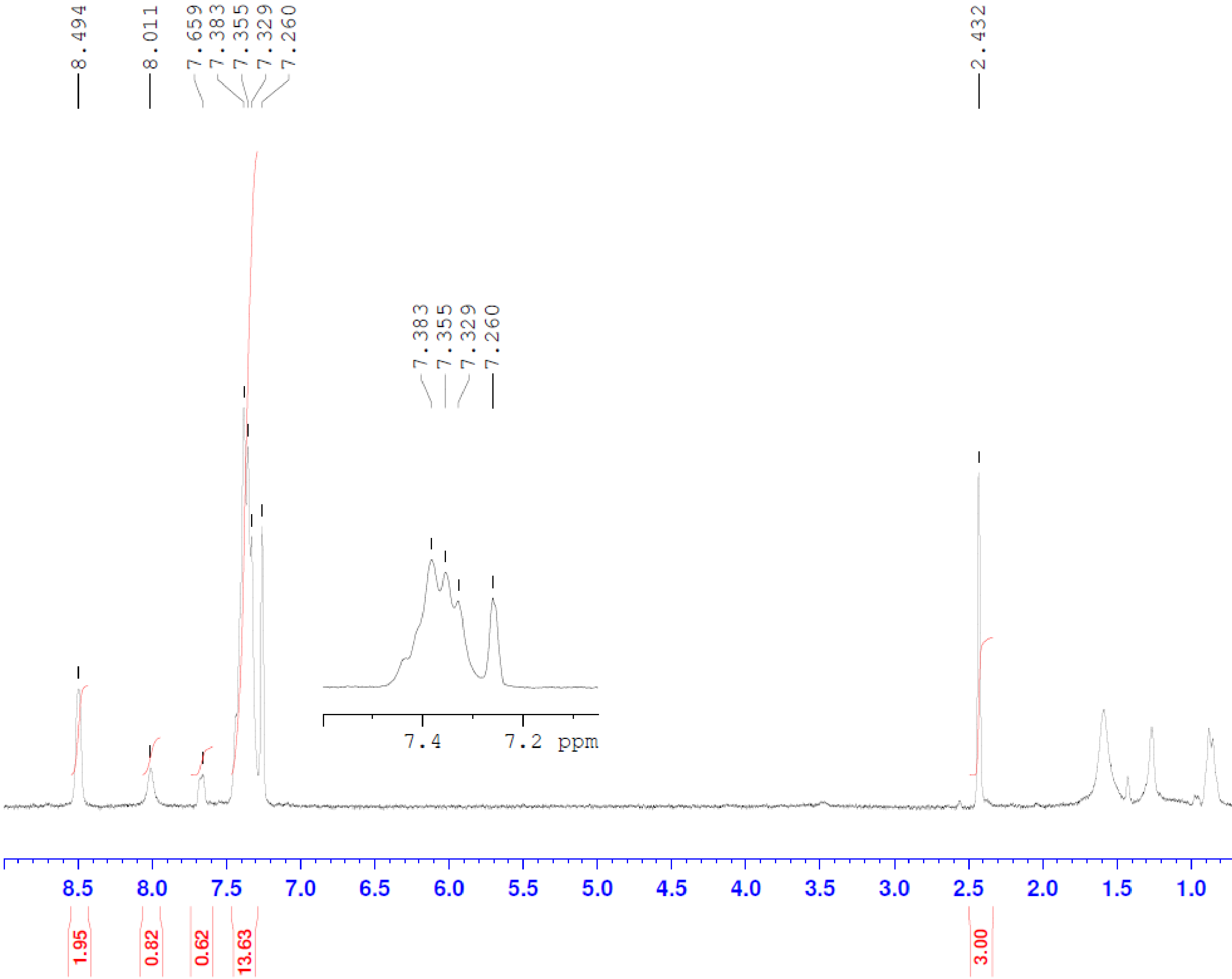

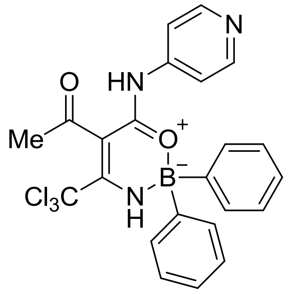

^{1}H NMR spectrum of NBC22 in $CDCl_3$

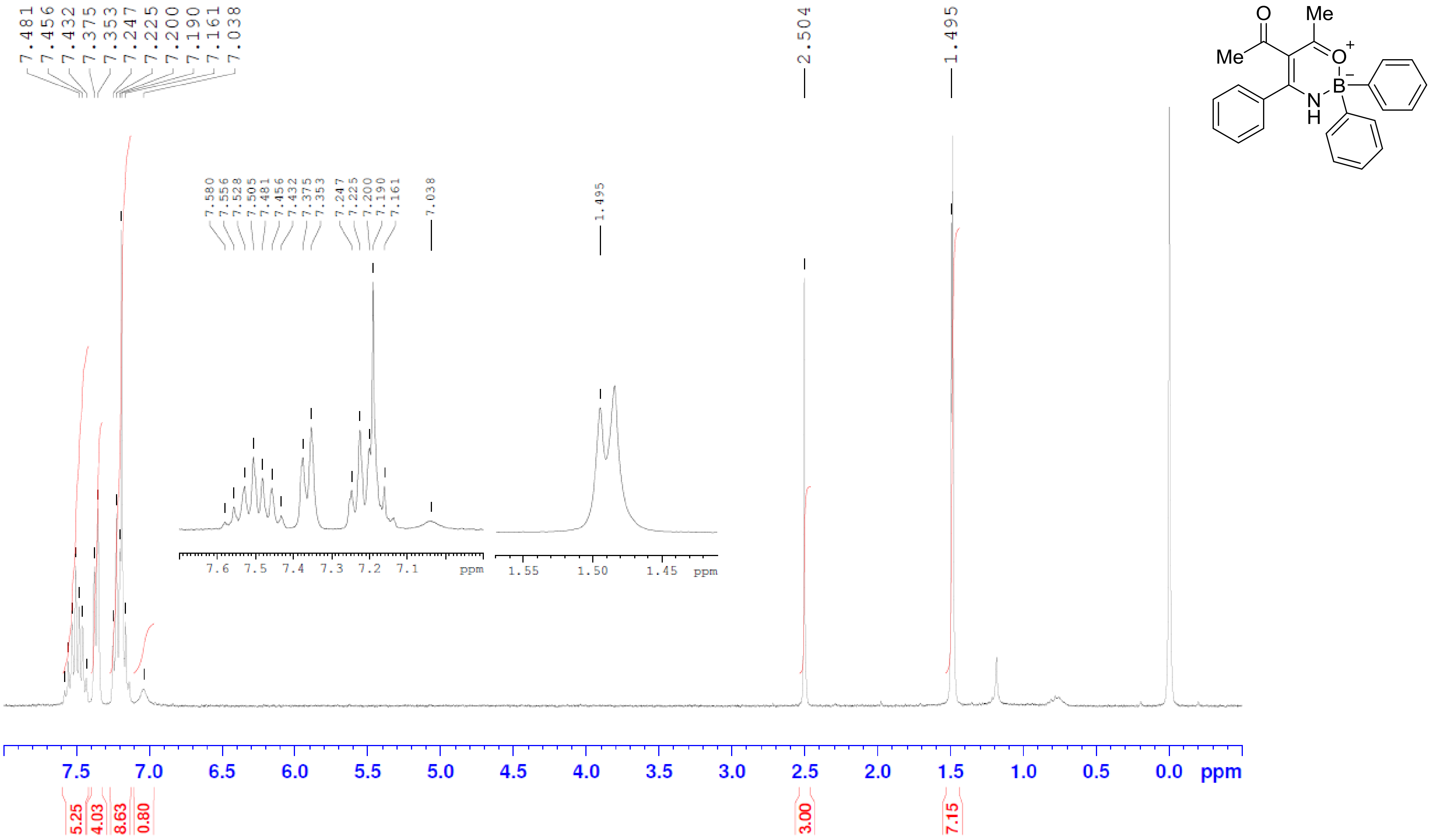

^{1}H NMR spectrum of NBC23 in $CDCl_3$

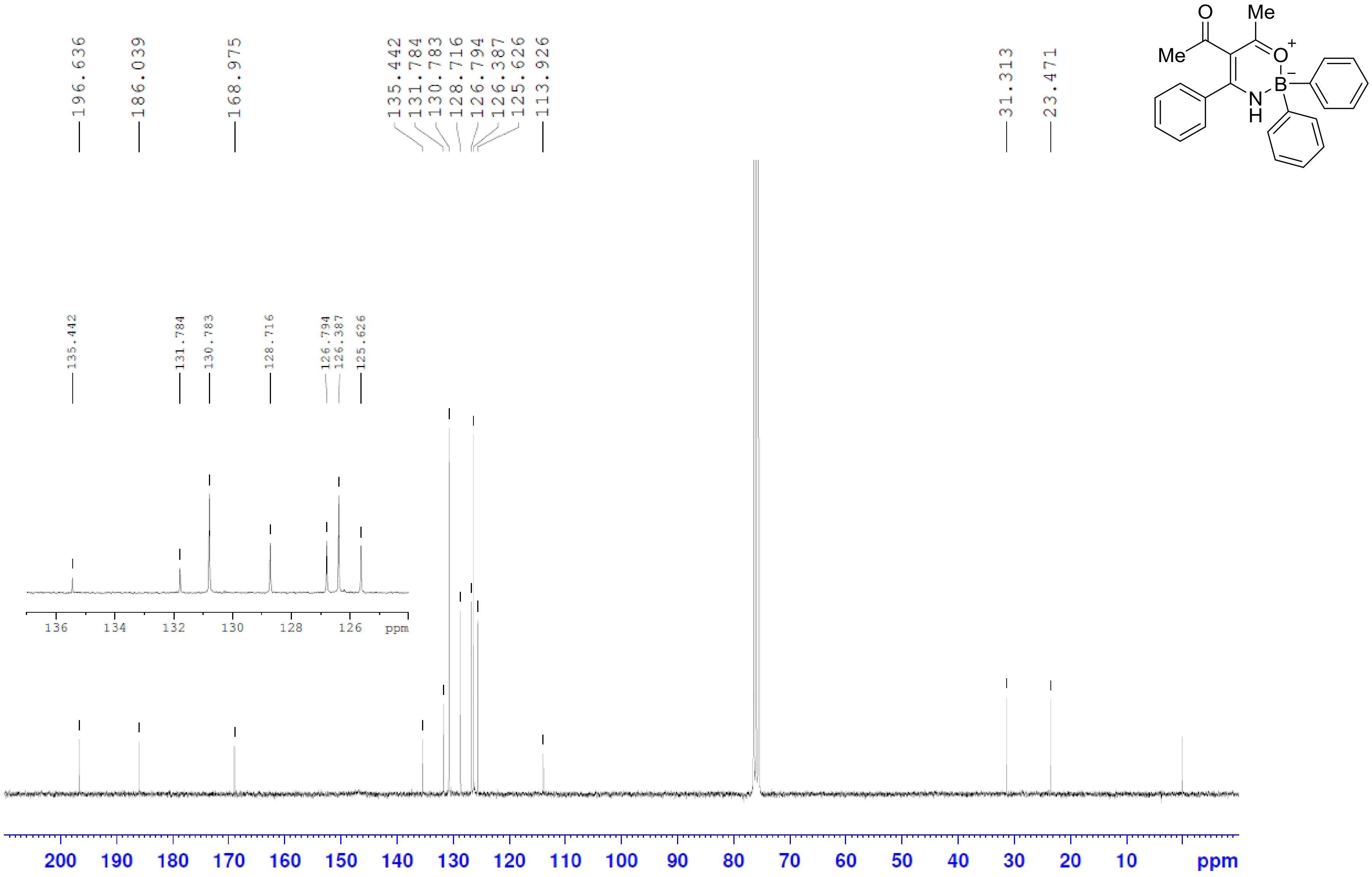

^{13}C NMR spectrum of NBC23 in $CDCl_3$

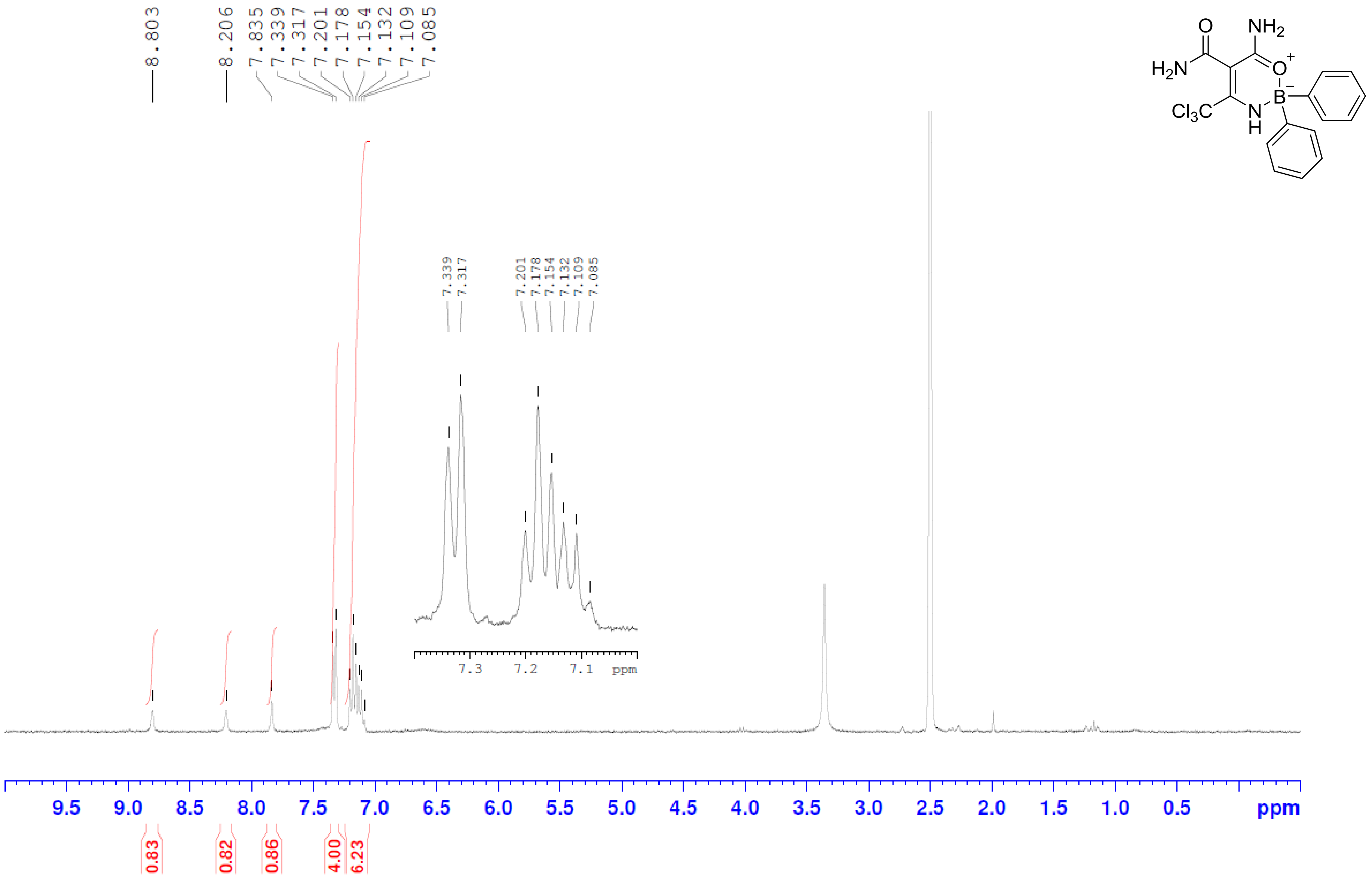

^{1}H NMR spectrum of NBC24 in $CDCl_3$

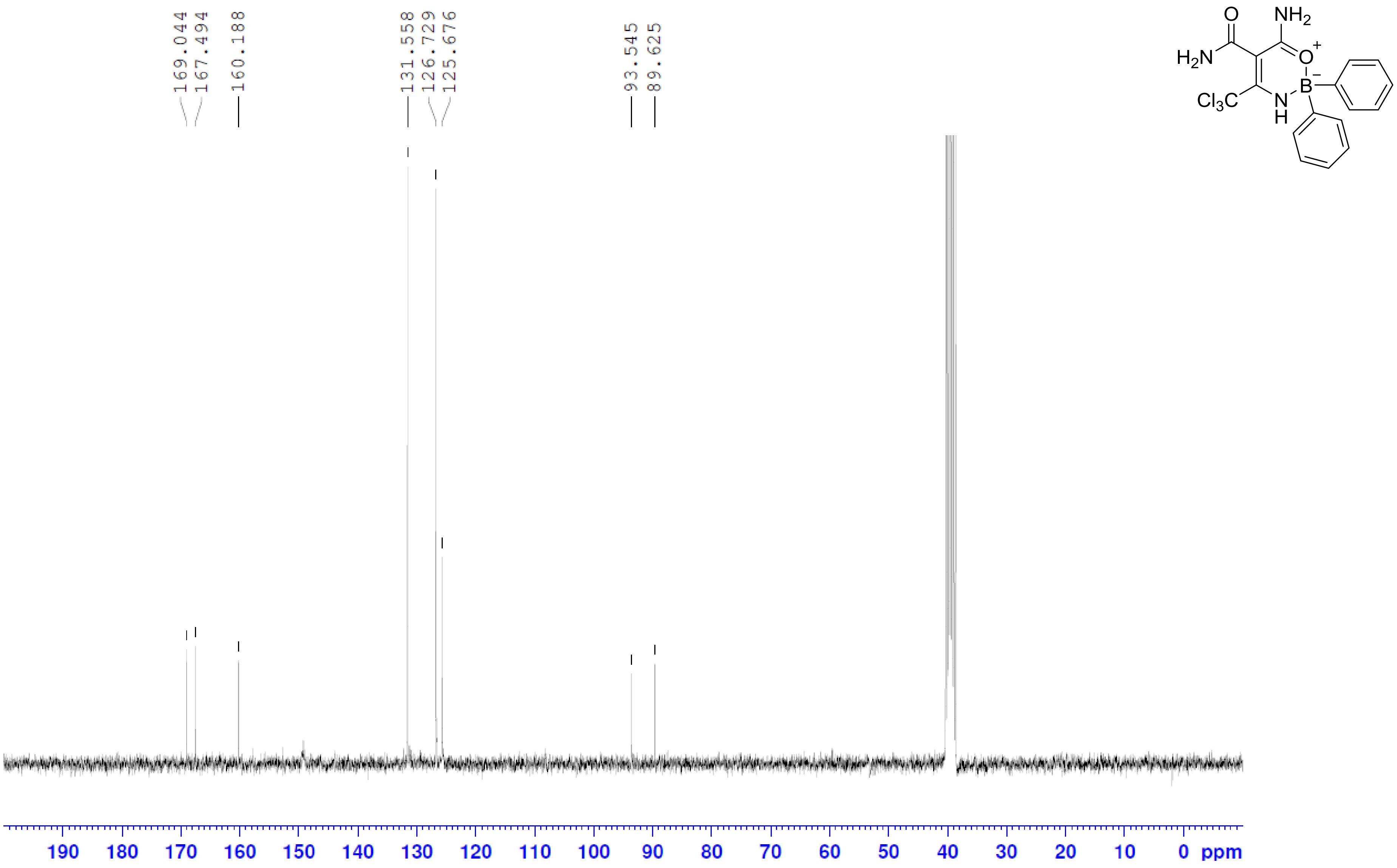

^{13}C NMR spectrum of NBC24 in $CDCl_3$

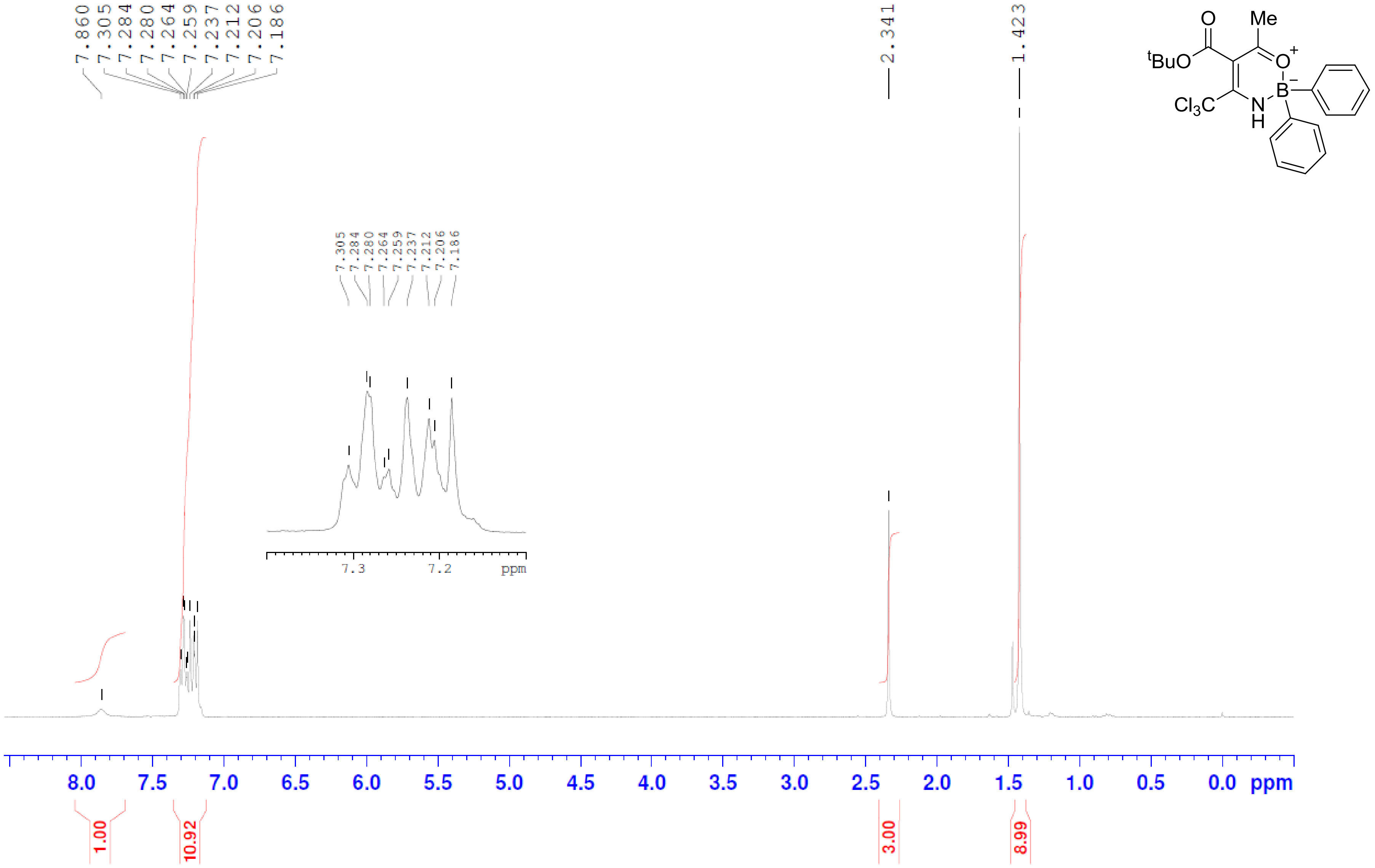

^{1}H NMR spectrum of NBC25 in $CDCl_3$

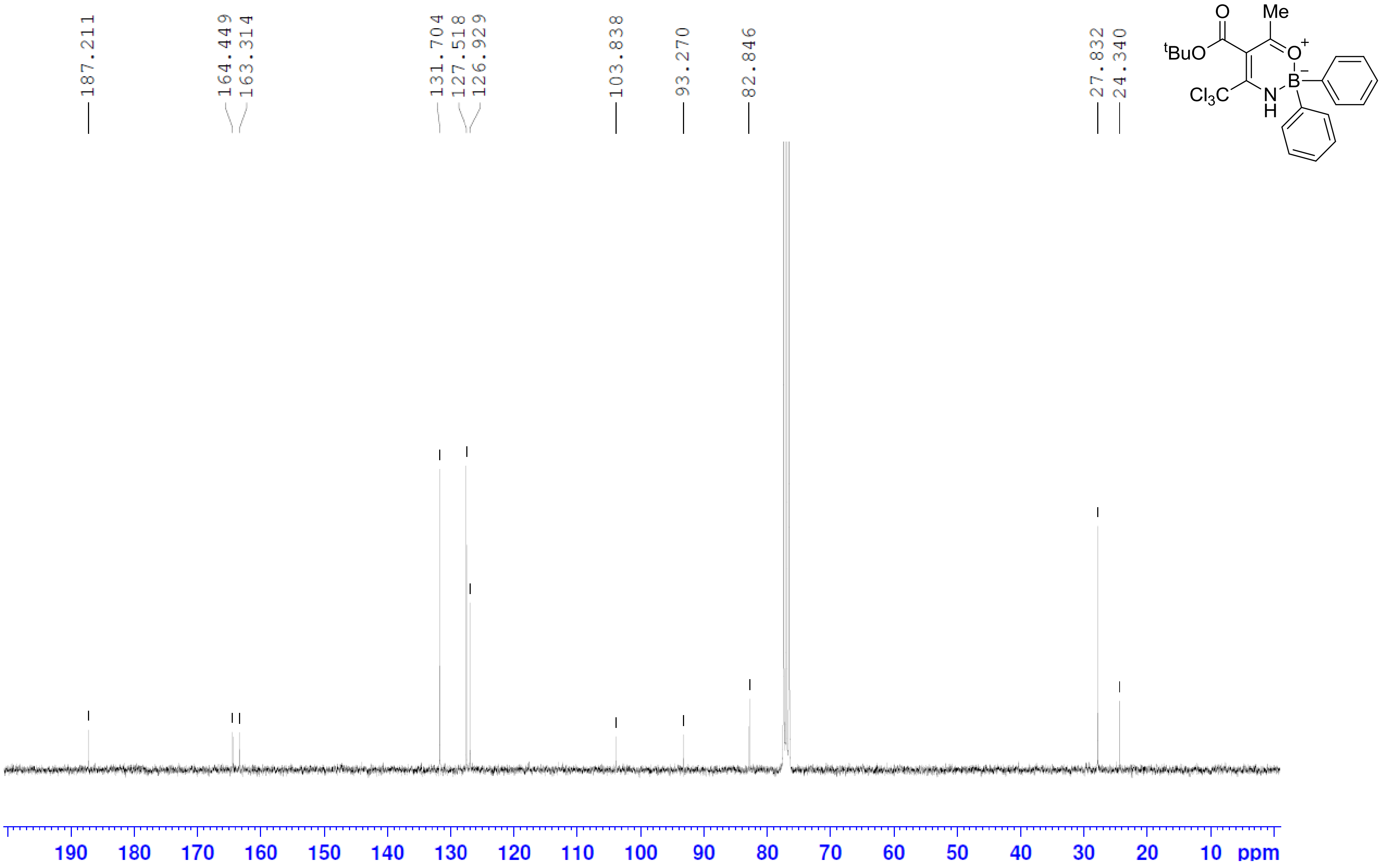

^{13}C NMR spectrum of NBC25 in $CDCl_3$

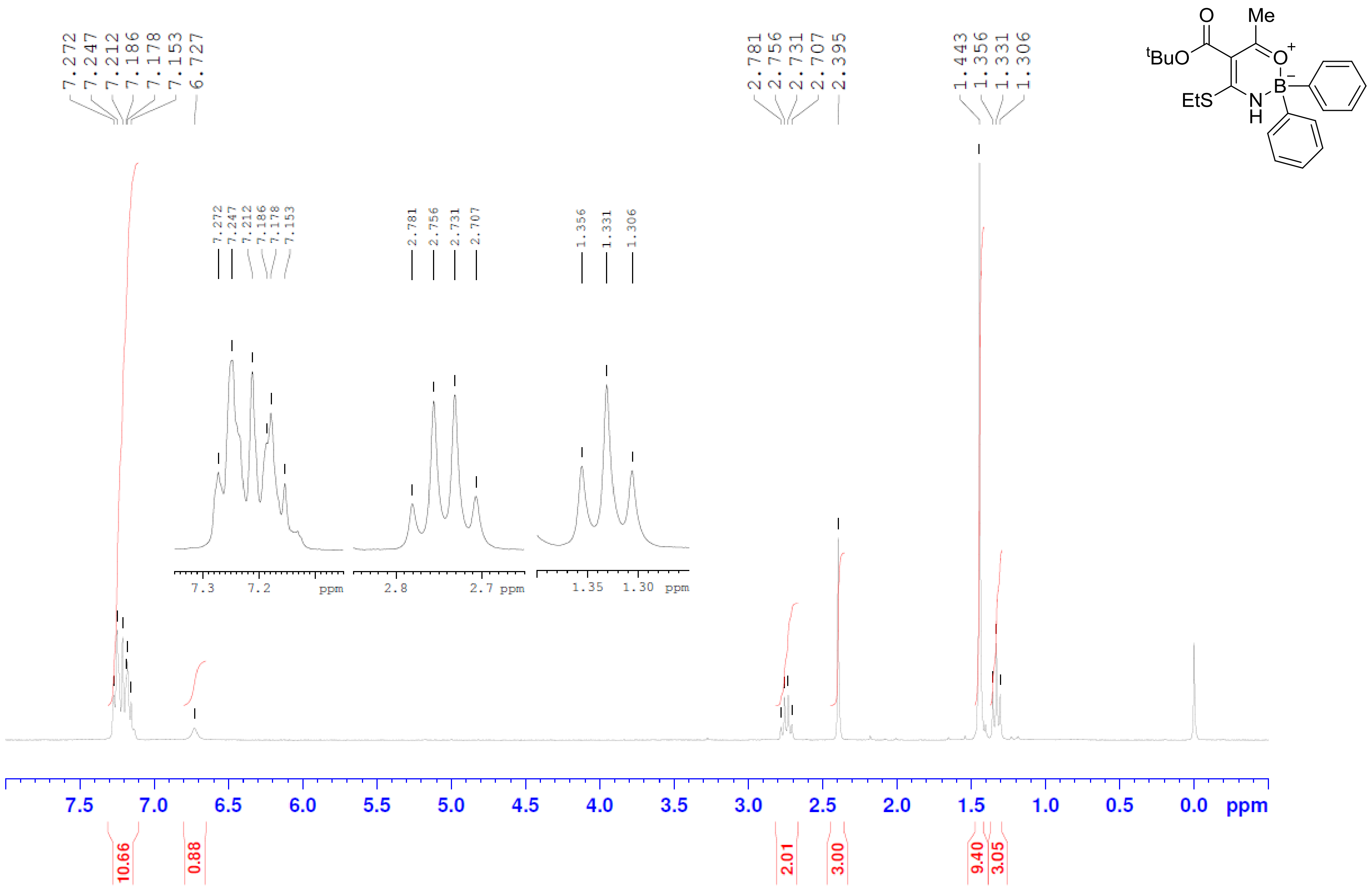

^{1}H NMR spectrum of NBC26 in $CDCl_3$

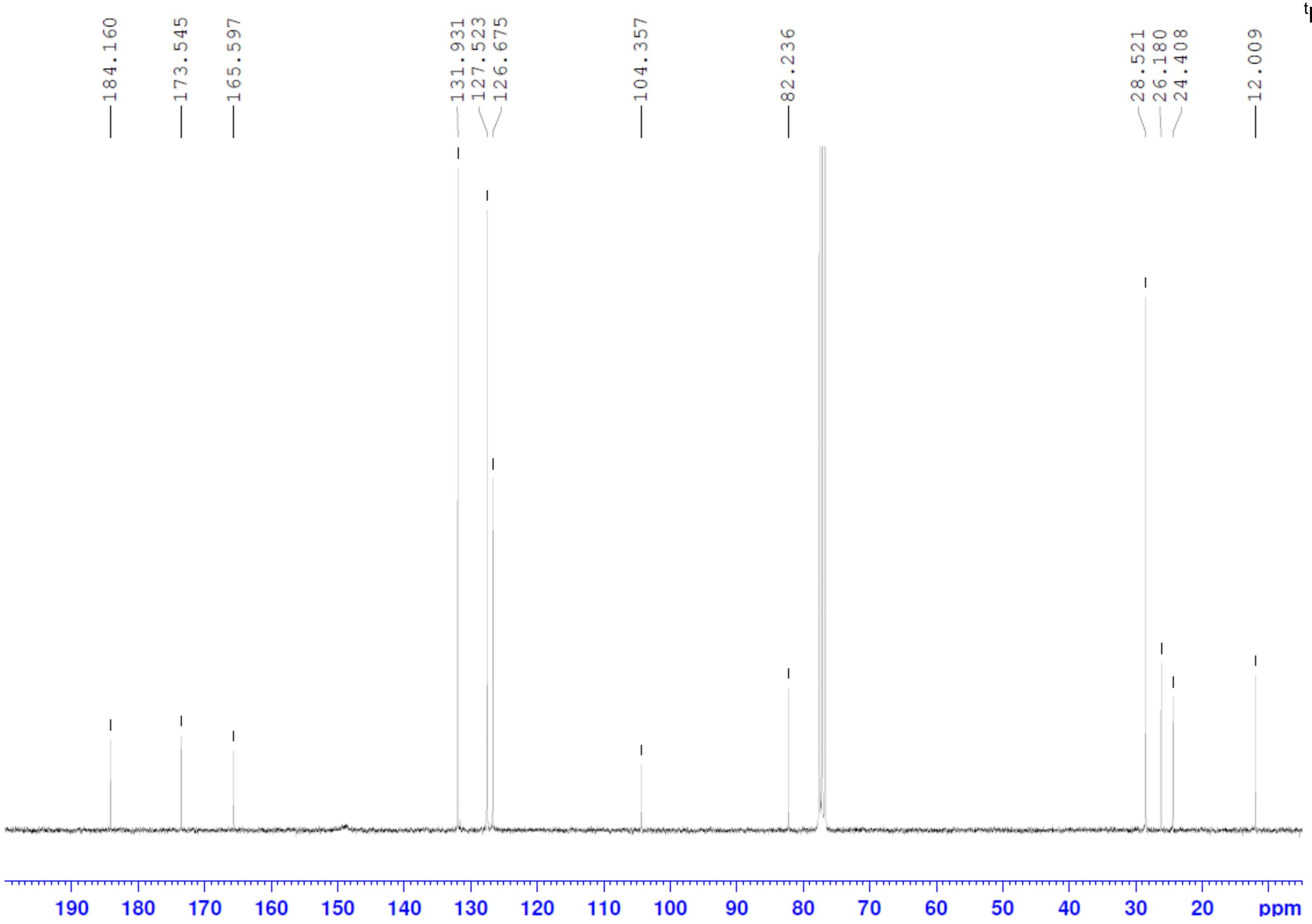

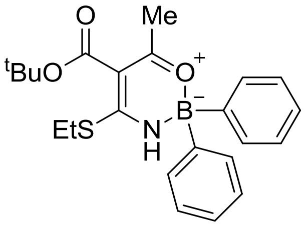

^{13}C NMR spectrum of NBC26 in $CDCl_3$

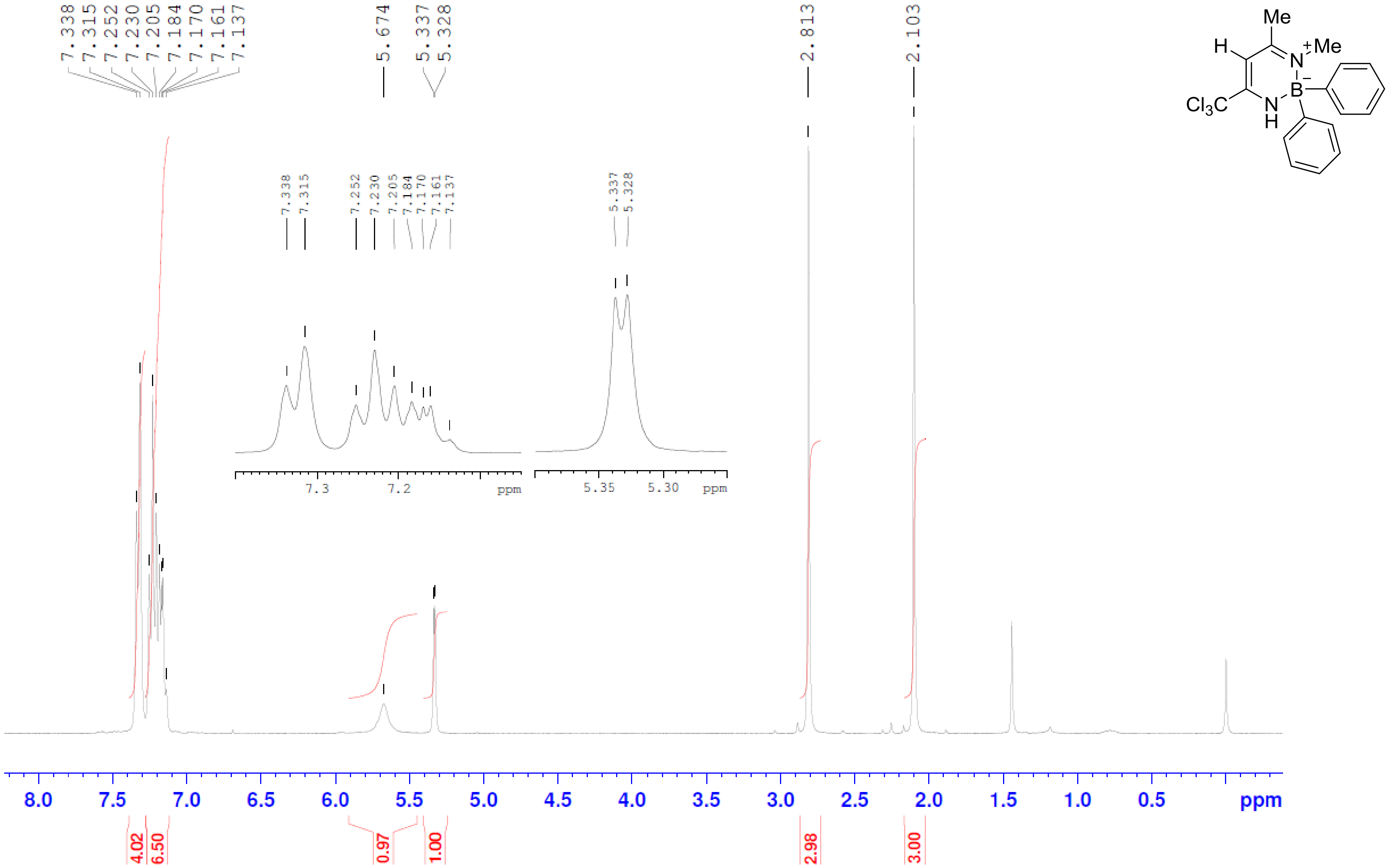

^{1}H NMR spectrum of NBC27 (**18**) in $CDCl_3$

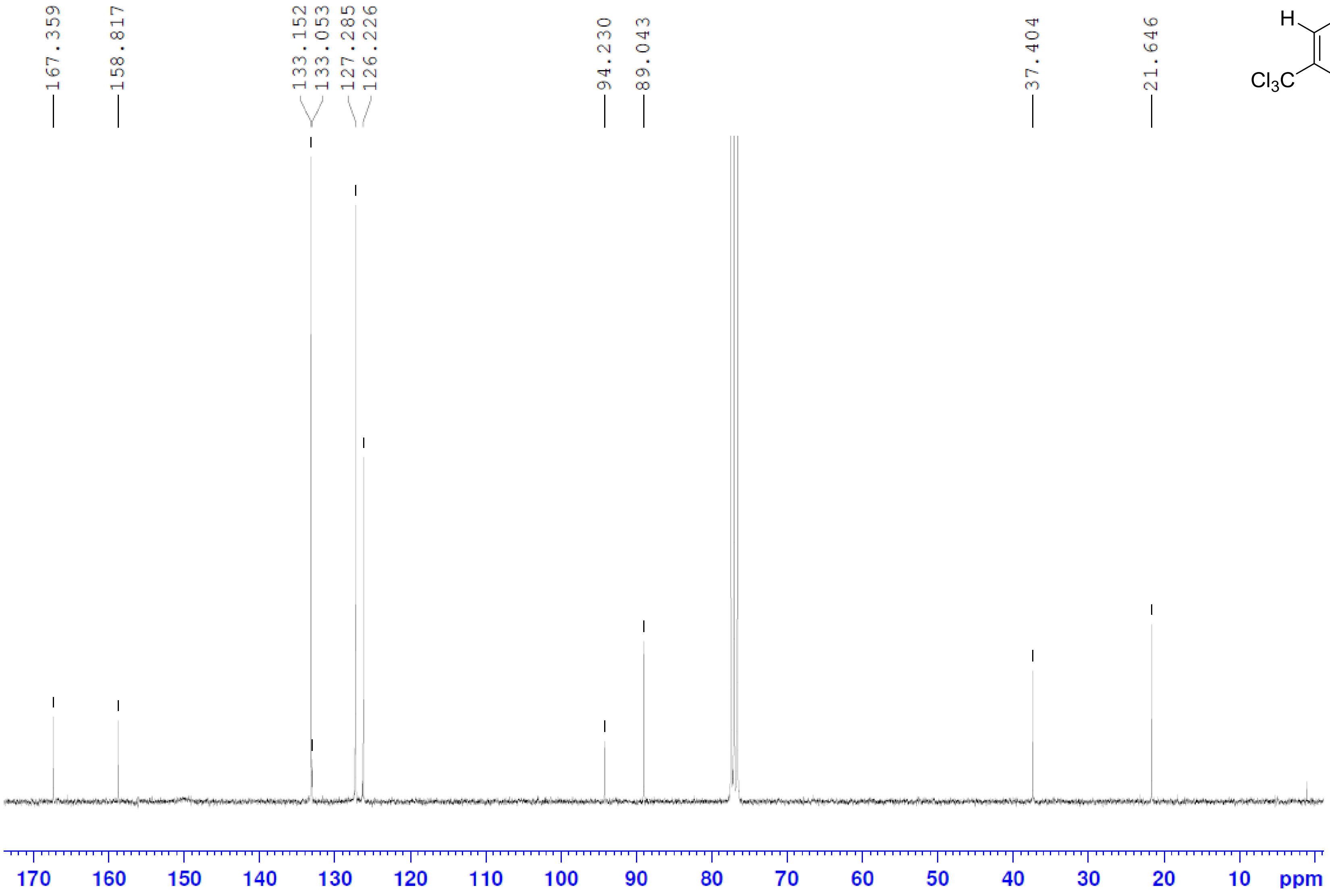

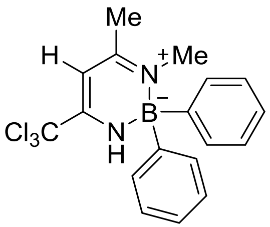

^{13}C NMR spectrum of NBC27 (**18**) in $CDCl_3$

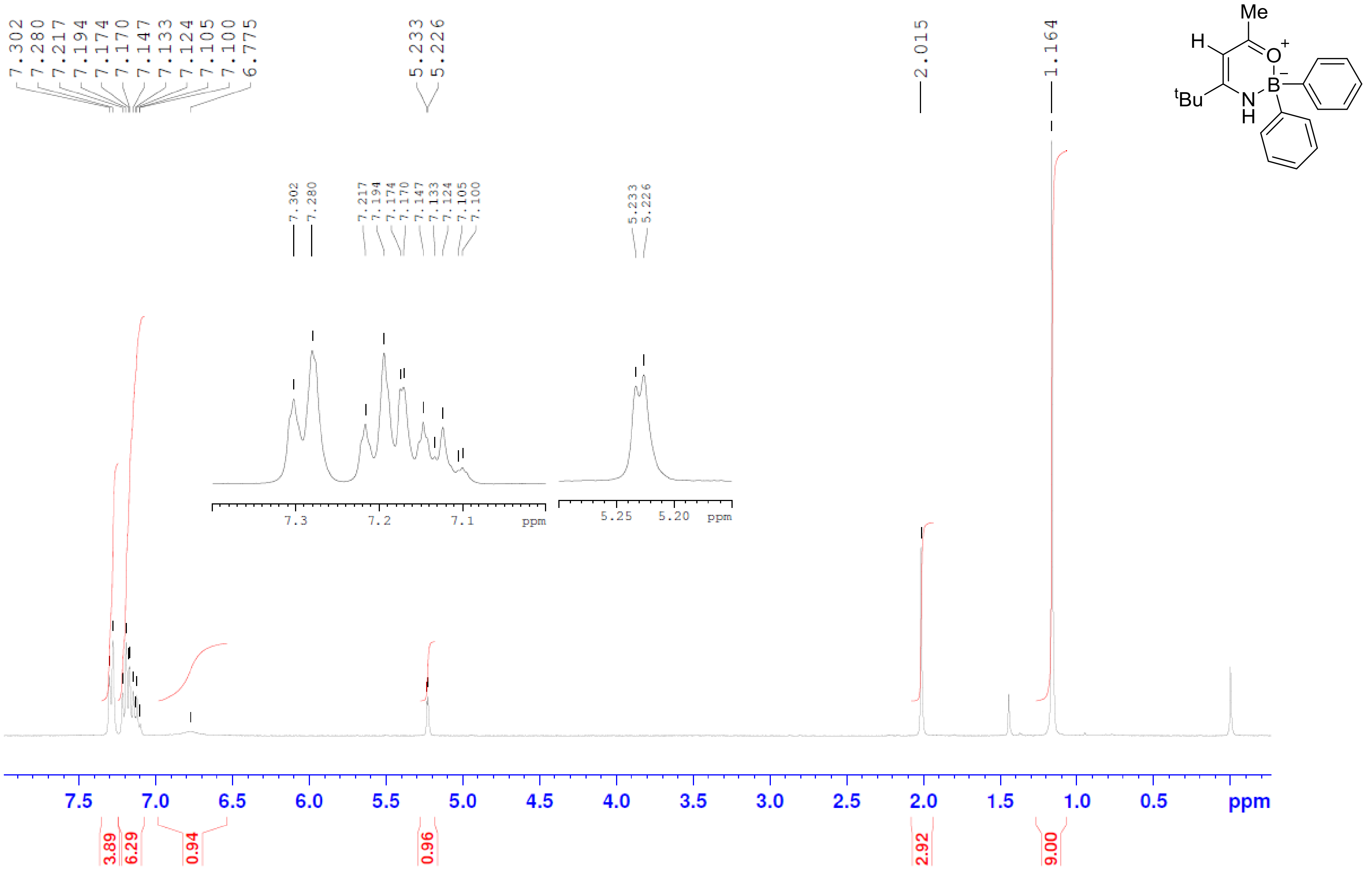

^{1}H NMR spectrum of NBC28 in $CDCl_3$

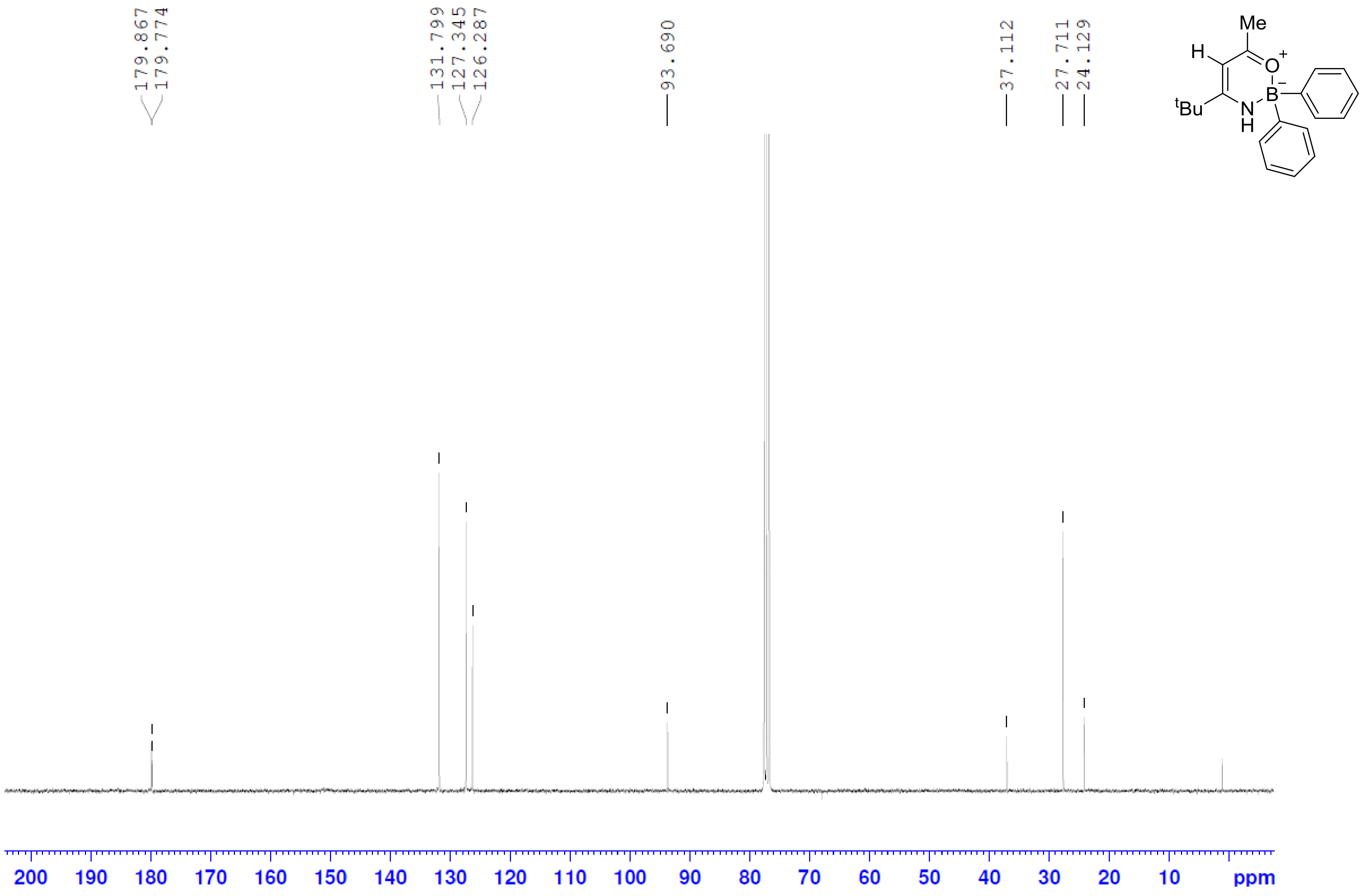

^{13}C NMR spectrum of NBC28 in $CDCl_3$

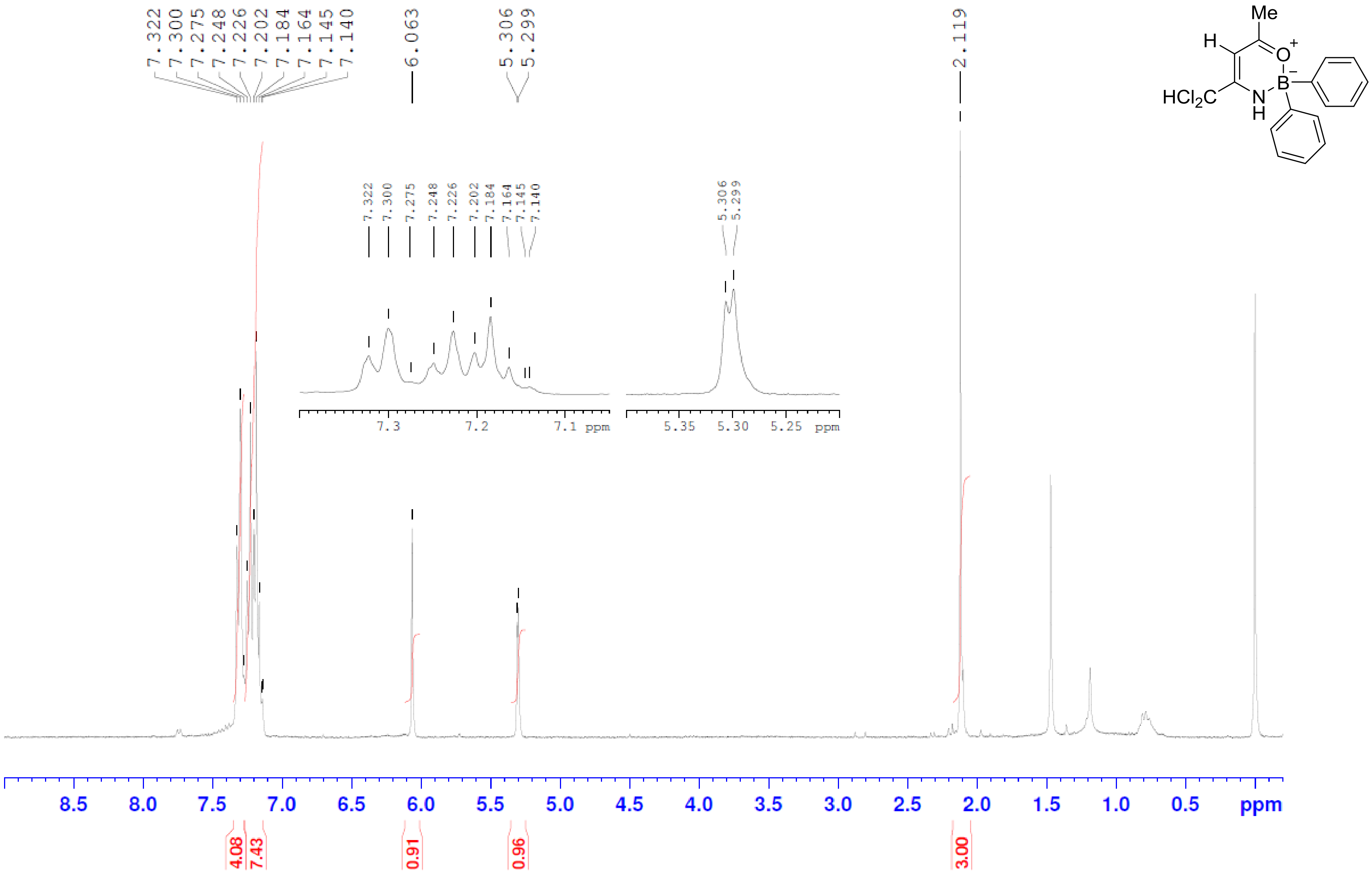

^{1}H NMR spectrum of NBC29 in $CDCl_3$

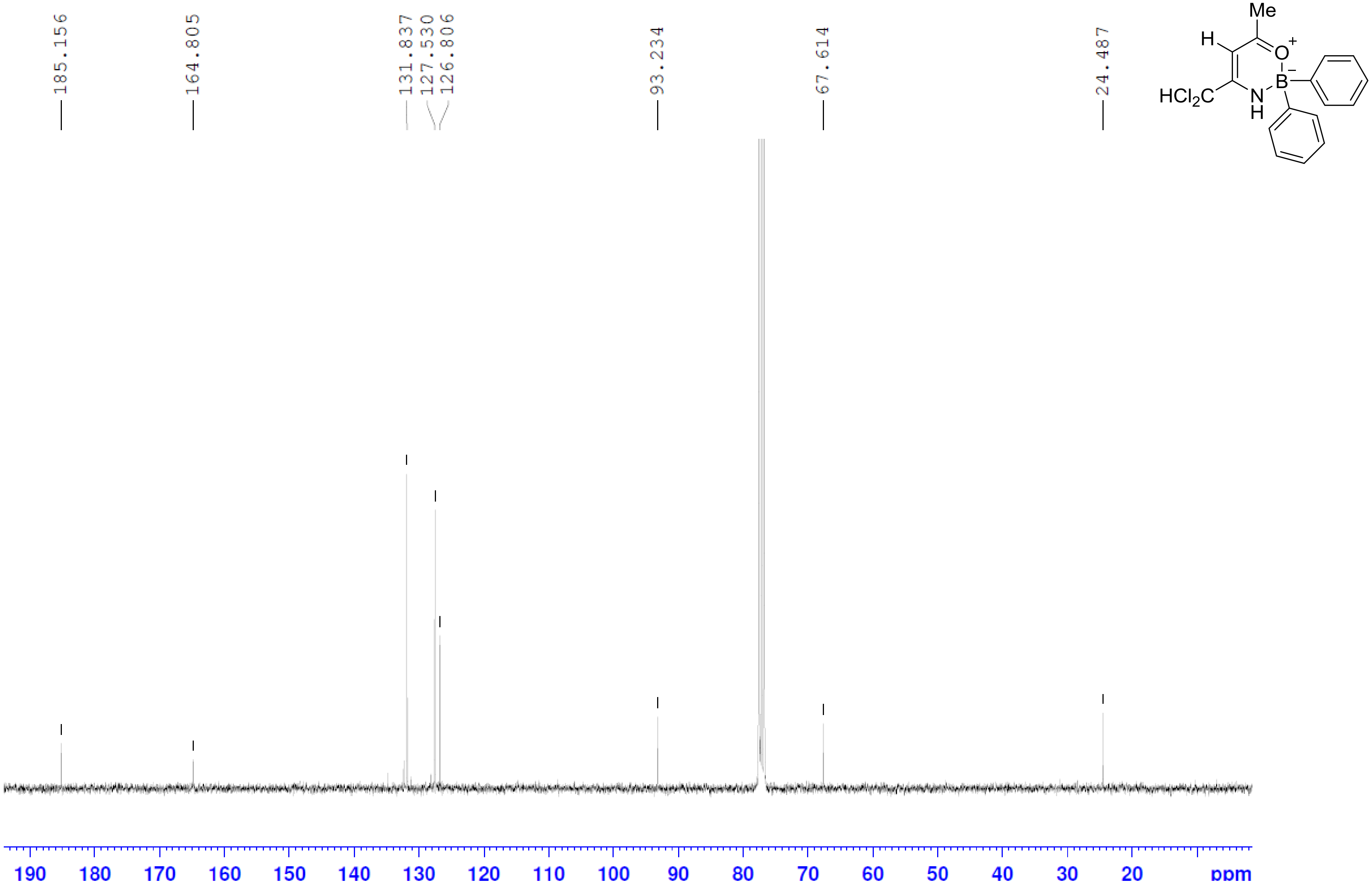

^{13}C NMR spectrum of NBC29 in $CDCl_3$

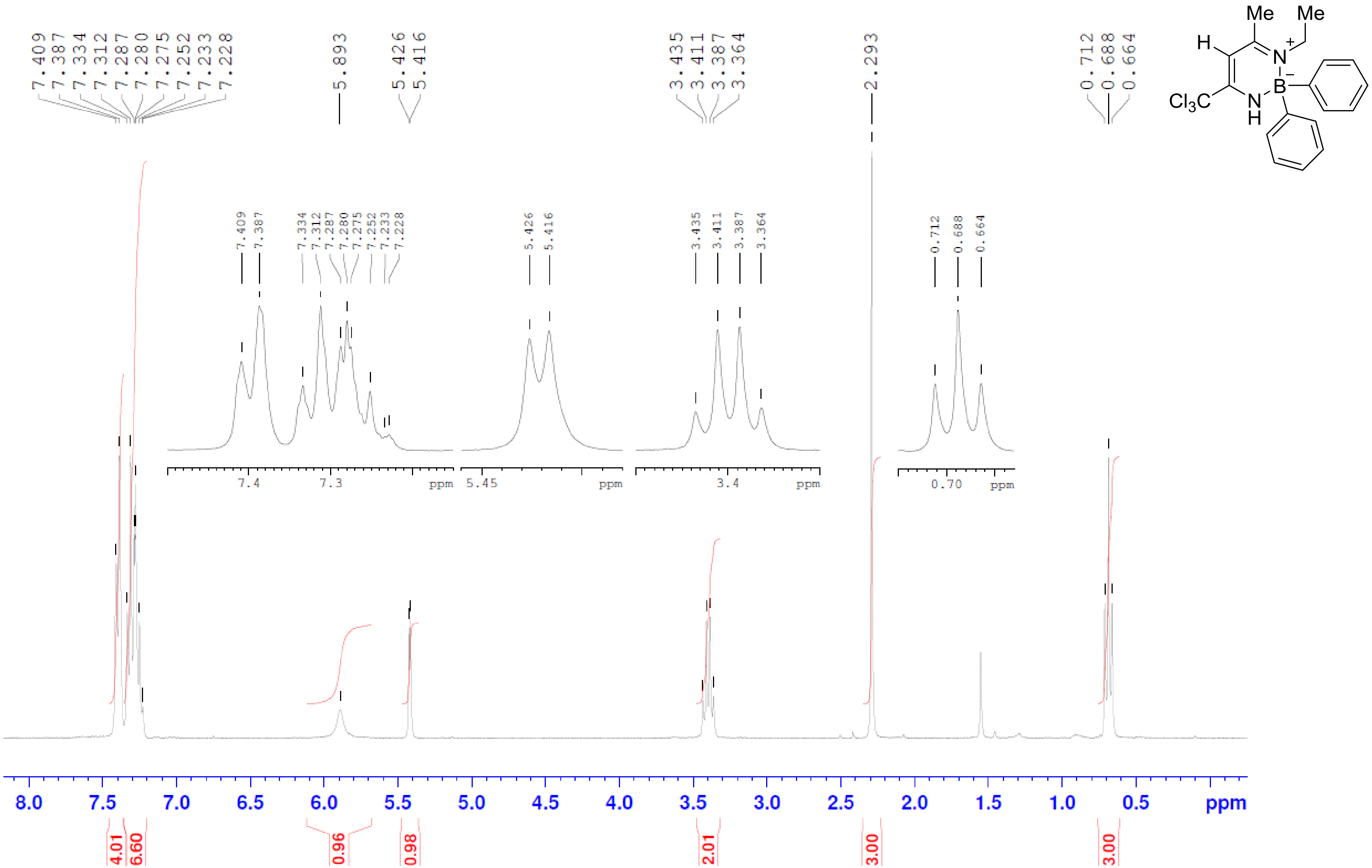

^{1}H NMR spectrum of NBC30 (**19**) in $CDCl_3$

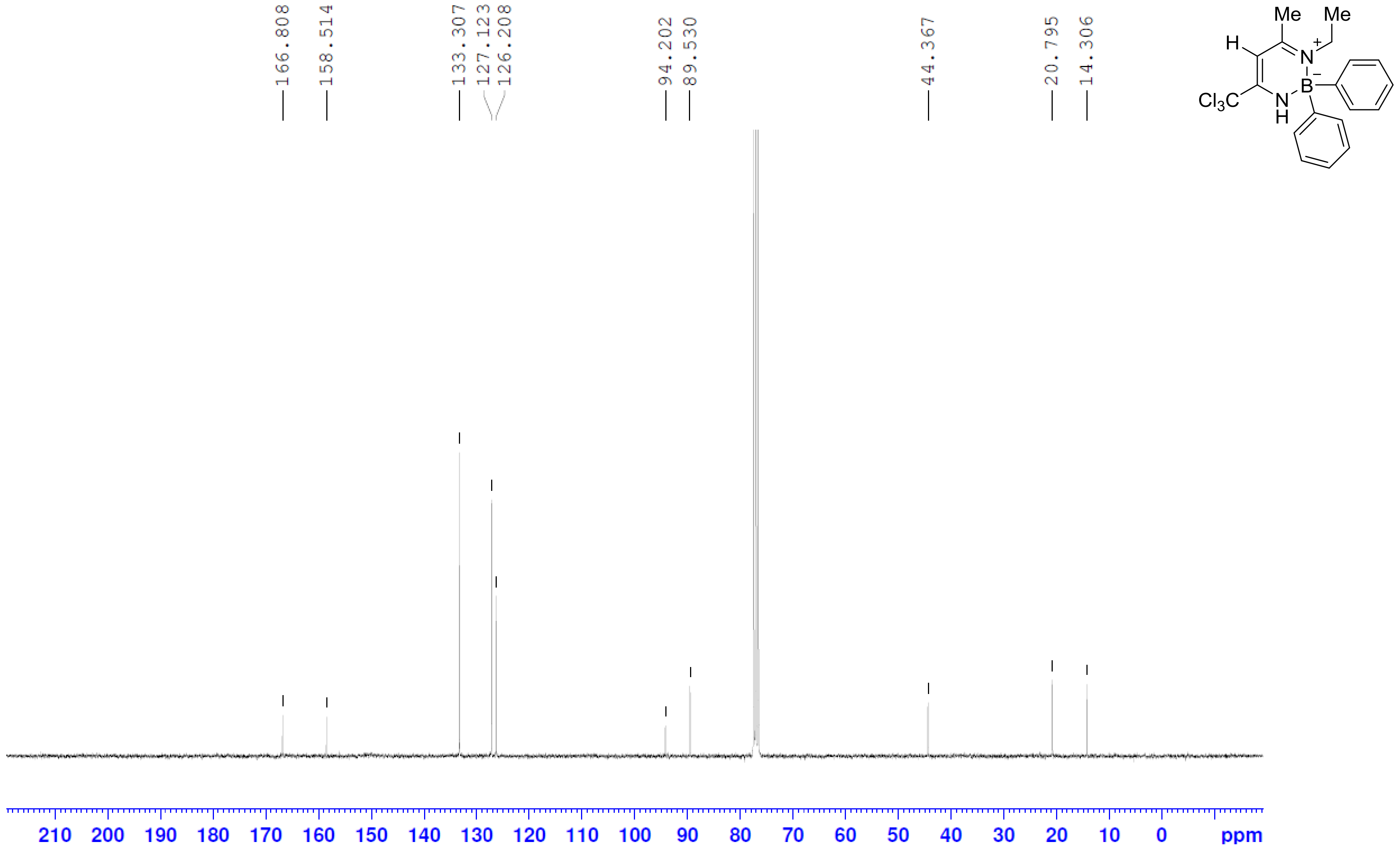

^{13}C NMR spectrum of NBC30 (**19**) in $CDCl_3$

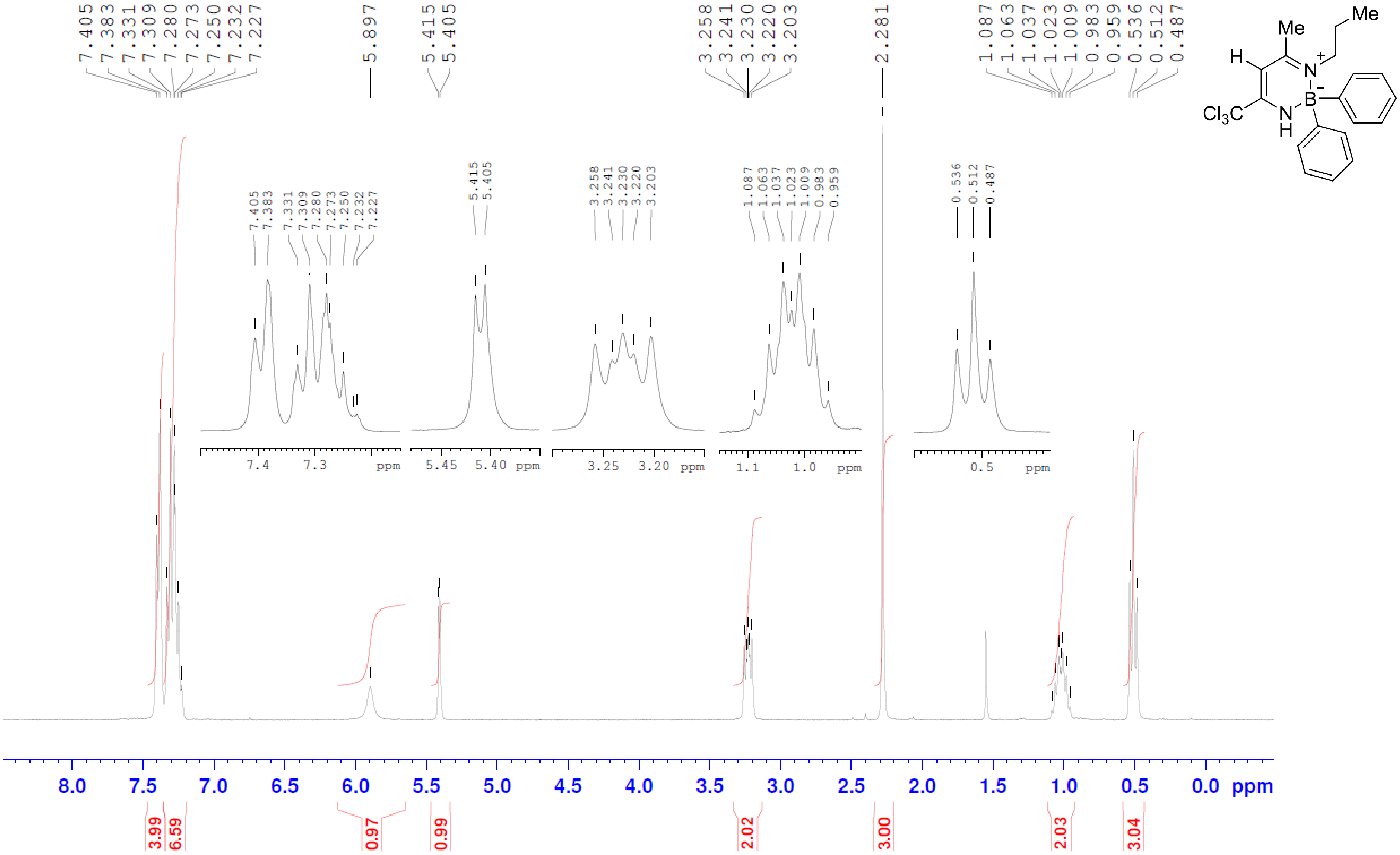

^{1}H NMR spectrum of NBC31 in $CDCl_3$

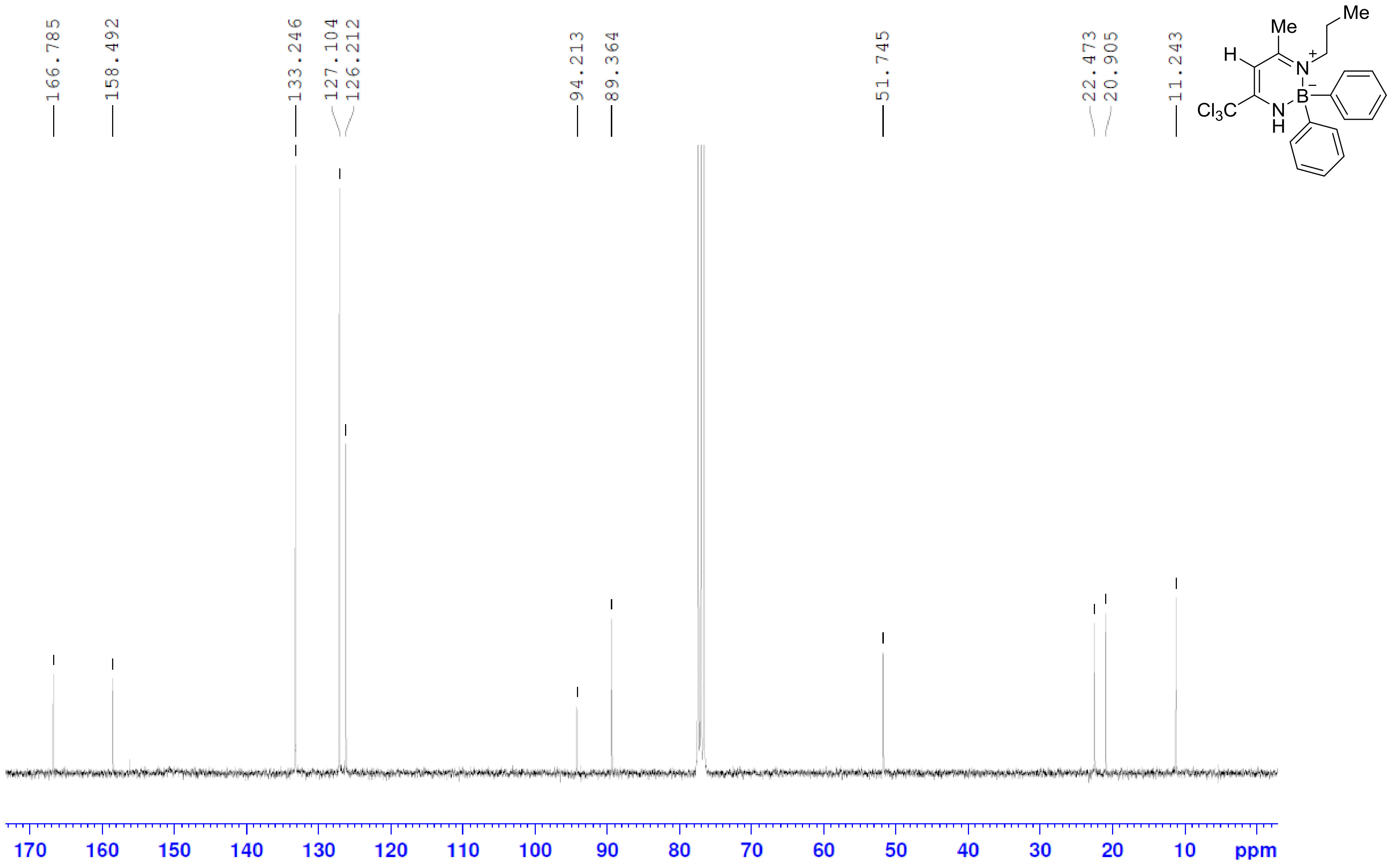

^{13}C NMR spectrum of NBC31 in $CDCl_3$

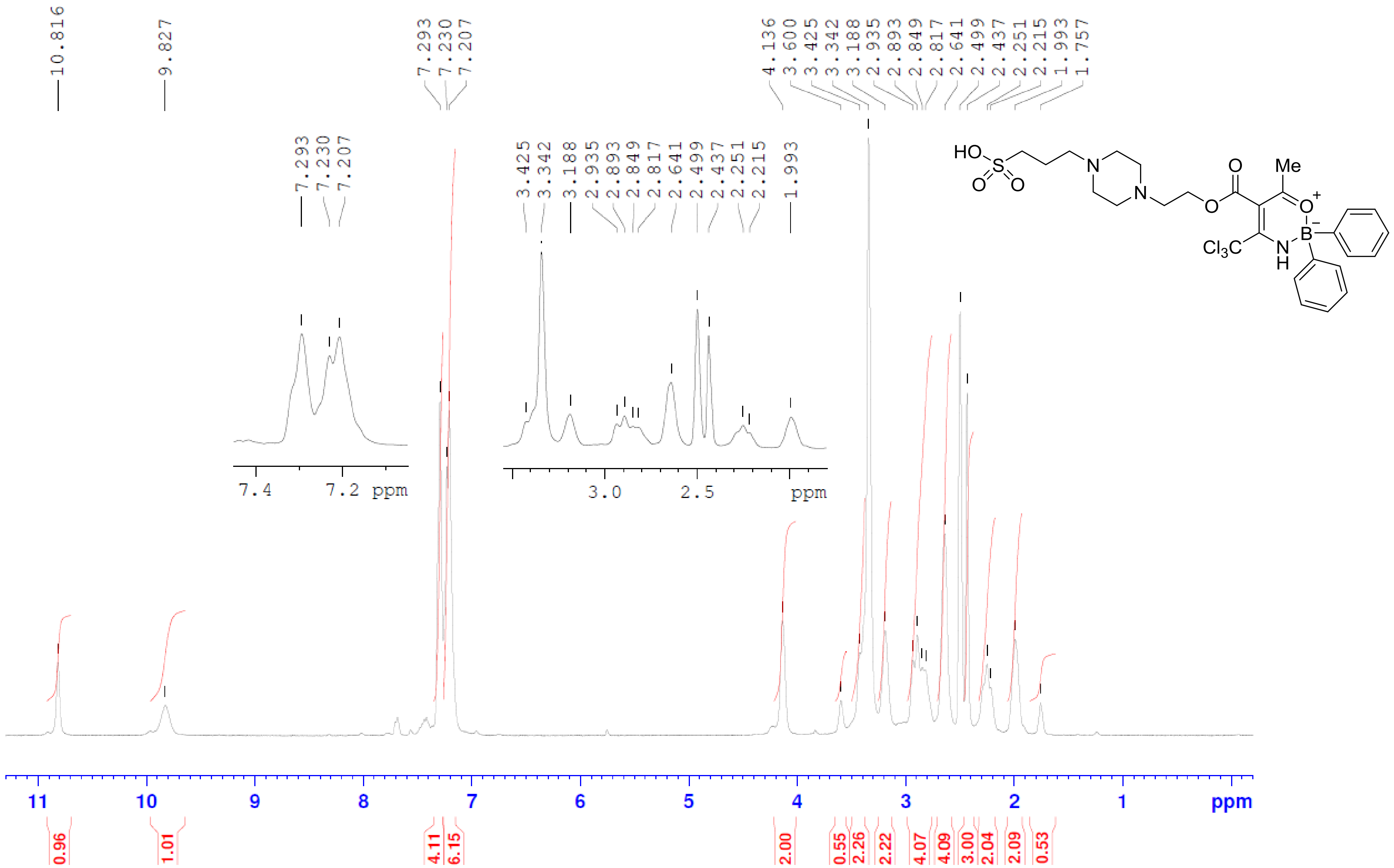

^{1}H NMR spectrum of NBC-EPPS in DMSO-d_6

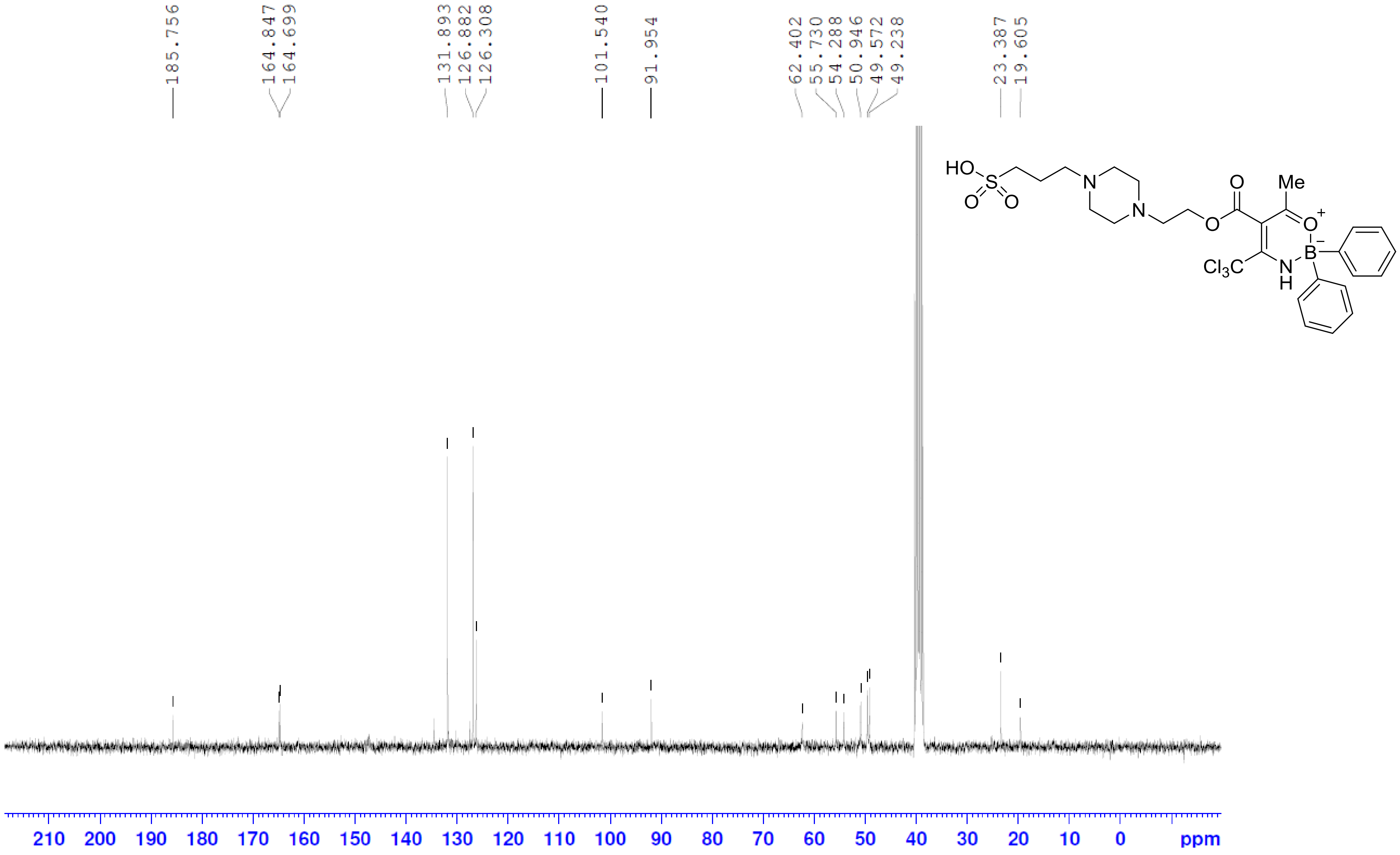

^{13}C NMR spectrum of NBC-EPPS in DMSO-d
